# C3–H methylenephosphonylation of azaarenes enabled by catalyst-controlled regioselective cyclizative rearrangement

**DOI:** 10.1039/d6sc03456j

**Published:** 2026-06-11

**Authors:** Dan Liu, Fang-Zhou Li, Yu-Ping He, Hua Wu

**Affiliations:** a Shanghai Key Laboratory for Molecular Engineering of Chiral Drugs, Shanghai Frontiers Science Center for Drug Target Identification and Delivery, and Laboratory of Innovative Immunotherapy, School of Pharmaceutical Sciences, Shanghai Jiao Tong University Shanghai 200240 China hua.wu@sjtu.edu.cn; b Department of Chemistry, College of Sciences, Shanghai University Shanghai 200444 China

## Abstract

The incorporation of phosphonate moieties into azaarenes *via* C–H functionalization is of paramount importance in pharmaceutical research, yet it remains a formidable synthetic challenge. Herein, we report an intermolecular cyclizative rearrangement approach for the efficient site-selective methylenephosphonylation of diverse azaarenes. This reaction proceeds *via* an orchestrated cascade sequence comprising regioselective 1,3-dipolar cycloaddition, [3,5]-sigmatropic rearrangement, and selective C–C bond cleavages. As a Lewis acid catalyst, ZnBr_2_ is crucial to this transformation: it not only delivers high conversion but also controls the regioselectivity of the initial cyclization, which in turn dictates the chemoselectivity of the entire process. This protocol is applicable to late-stage functionalization of various pharmaceuticals and ligands and facilitates further product diversification for advanced uses. This reaction represents the first example of the C–H methylenephosphonylation of arenes.

## Abstract 2

We report a ZnBr_2_-catalyzed intermolecular cyclizative rearrangement enabling site-selective methylenephosphonylation of azaarenes, applicable to late-stage modification of pharmaceuticals and ligands.

## Graphical abstract

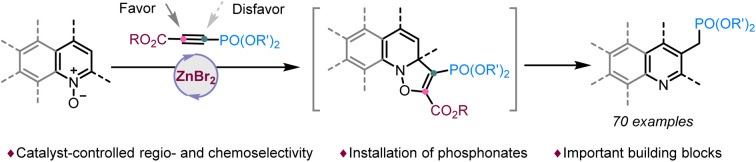

## Introduction

**Fig. 1:**
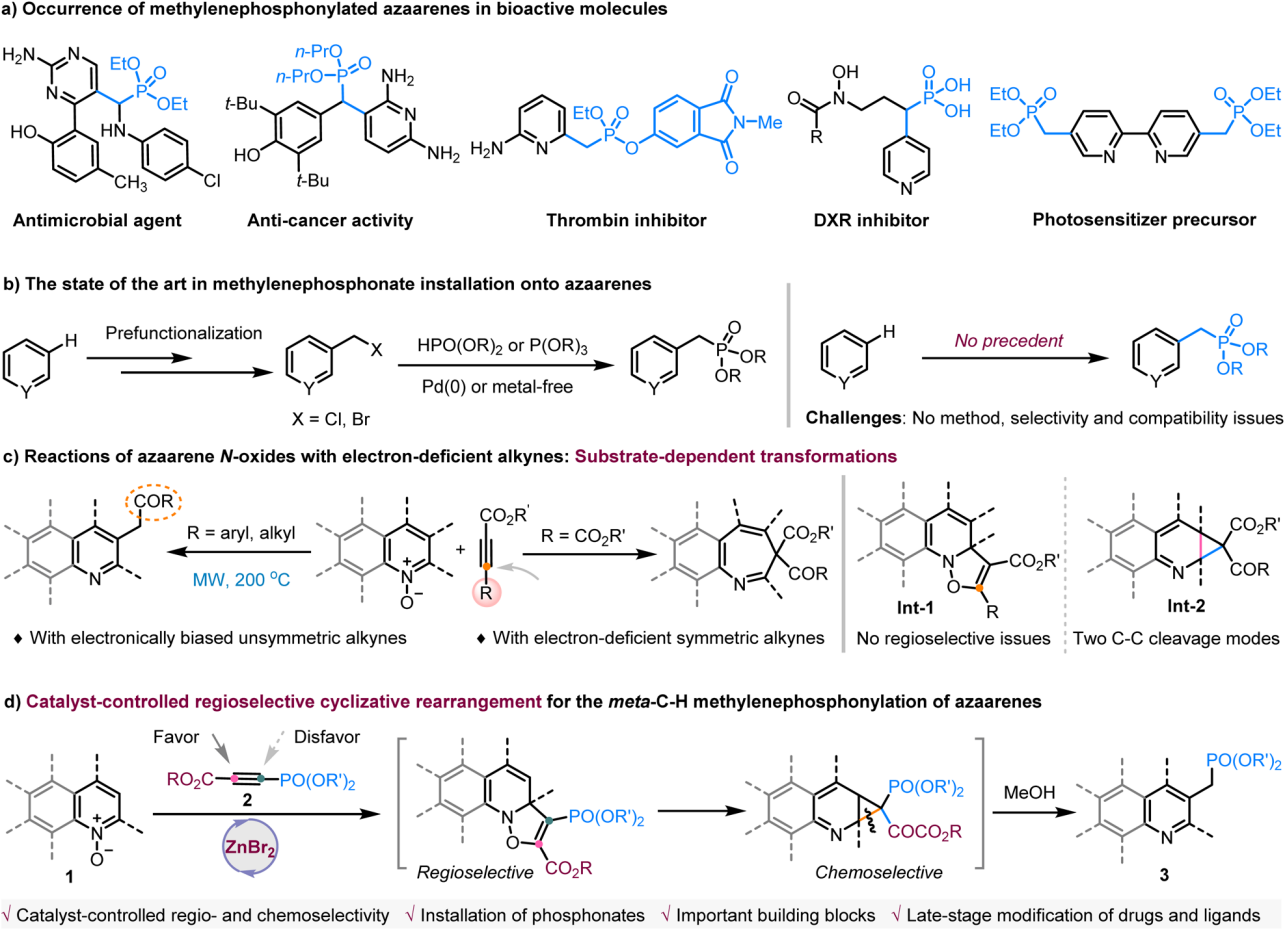
C–H methylenephosphonylation of azaarenes.

Phosphonates represent essential structural motifs in biological systems, where they govern a multitude of metabolic processes.^1,2^ Among them, azaarenes bearing methylenephosphonate moieties have emerged as core scaffolds in a number of pharmaceuticals and functional materials, endowed with diverse bioactivities such as antimicrobial,^3^ anticancer,^4^ thrombin inhibitory,^5^ and 1-deoxy-d-xylulose-5-phosphate reductoisomerase (DXR) inhibitory properties,^6^ alongside their utility as precursors to photosensitizers (Fig. 1a).^7,8^ Accordingly, the functionalization of diverse azaarenes with methylenephosphonate moieties is of paramount importance for the development of novel drug candidates. Conventional methods for the methylenephosphonylation of azaarenes rely heavily on stepwise transformation strategies, which necessitate prefunctionalization of azaarenes at the target site, followed by sequential cross-coupling or S_N_2 reactions (Fig. 1b).^7–10^ Direct and site-selective C–H methylenephosphonylation undoubtedly represents one of the most straightforward and efficient pathways to this goal. Nevertheless, to the best of our knowledge, such transformations remain unexplored, mainly hampered by the scarcity of efficient synthetic protocols, insufficient control of selectivity, and challenging compatibility issues (Fig. 1b).

Azaarene *N*-oxides are readily accessible *via* the oxidation of parent azaarenes and act as versatile 1,3-dipoles in a broad range of reactions, thus offering a straightforward and modular platform for the site-selective functionalization of azaarenes.^11–14^ However, previous reports are predominantly limited to *ortho*-functionalization, and methodologies enabling selective functionalization at other positions remain scarce,^15–17^ particularly for C3–H bond functionalization.^18–23^ In this context, Maulide and coworkers reported an elegant *meta*-selective alkyne oxyarylation of pyridine and quinoline *N*-oxides to afford metyrapone analogues, albeit needing 200 °C under microwave irradiation to achieve sufficient stepwise reactivity (Fig. 1c).^22^ While the use of more electron-deficient alkynes, such as diacetylene dicarboxylate (DAAD), can indeed significantly lower the reaction temperature, it diverts the reaction toward a completely distinct pathway. As documented by Hamana in 1983, such conditions lead exclusively to the formation of benzazepine derivatives (Fig. 1c).^24^ Both reactions proceed through a cascade of 1,3-dipolar cycloaddition and [3,5]-sigmatropic rearrangement, generating Int-1 and Int-2 as the respective intermediates. Notably, in both cases, the regioselectivity in the initial 1,3-dipolar cycloaddition is not a concern (Int-1), since the alkynes employed are either electronically differentiated unsymmetric alkynes or symmetric electron-deficient alkynes. Moreover, the observed chemodivergence arises from divergent C–C bond cleavage in Int-2, a process that is also clearly substrate-controlled. Inspired by these studies, we hypothesized that catalyst regulation of chemoselectivity might allow the realization of C3–H methylenephosphonylation of azaarenes. To this end, we sought a simple, readily available phosphonate-substituted alkyne bearing an electron-withdrawing group (EWG) at the other terminus, and under such circumstances, alkynyl phosphonate bearing an ester moiety represents the optimal choice.^25^ Nevertheless, the realization of this transformation poses substantial challenges. Beyond reaction reactivity, particular attention must be paid to the regioselectivity of the initial cyclization (Int-1) and the chemoselectivity of the subsequent step (Int-2), which directly dictate the product distribution. Achieving catalyst-controlled selectivity in place of substrate control would undoubtedly expand the synthetic scope of this transformation, yet it is clearly a formidable task. In line with our research interest in rearrangement-based heterocyclic chemistry,^23,26–33^ we herein report the successful realization of this endeavor. Specifically, the Lewis acid catalyst ZnBr_2_ not only ensures the reactivity of the reaction but also guarantees its chemoselectivity, thus providing a modular and general synthesis of C3-methylenephosphonylated derivatives 3 from readily available azaarene *N*-oxides 1 and alkynes 2 (Fig. 1d). Notably, this protocol allows for the direct one-pot conversion of unmasked azaarenes to product 3, with its practical utility corroborated by gram-scale synthesis and late-stage modification of diverse drug molecules and ligands. This method also addresses the gap whereby existing *meta*-functionalization strategies, such as transition-metal-catalyzed C–H activation,^34–37^ temporary dearomatization-rearomatization,^38–50^ ring-opening-ring-closing,^51–55^ organocatalytic cyclizative rearrangement,^23^ and radical coupling,^56–62^ cannot directly introduce phosphonyl moieties into azaarenes.

## Results and discussion

### Reaction optimization

Quinoline *N*-oxide 1a and methyl 3-(diethoxyphosphoryl)propiolate 2a were chosen as model substrates to assess the viability of the proposed rearrangement reaction (Table 1). When the reaction was carried out in THF at 80 °C, two compounds (3a’ and 4a) were observed. Notably, 3a’ is derived from our desired reaction pathway *via* intermediates Int-1a and Int-2a, whereas 4a originates from a distinctly different pathway involving reversed regiocyclization (Int-1a’) and internal C–C bond cleavage (Int-2a’). Additionally, we did not observe the corresponding benzazepine derivative formed by the ring expansion of intermediate Int-2a. On treating with MeOH, the unstable compound 3a’ could be efficiently converted into the desired methylenephosphonylated product 3a, while 4a underwent complete decomposition. Indeed, these two reaction pathways are mutually competitive, and the undesired pathway leading to the ring-expanded product 4a is significantly more favorable. To better delineate the selectivity between the two pathways, the relatively stable products 3a and 4a were individually isolated from parallel experiments, affording a ratio of 1 : 2 in THF and an overall low reaction yield of 3a (entry 1, Table 1). Notably, other alkynes 2 with the ester group replaced by alkyl or aryl moieties were found to be inert to this reaction. Subsequently, various solvents such as toluene, 1,2-dichloroethane (DCE), acetonitrile (MeCN) and dioxane all failed to significantly improve the chemoselectivity and yield (entries 2–5). We postulated that employing a Lewis acid catalyst might address this issue, given its ability to increase alkyne electrophilicity by means of the chelation effect. Furthermore, the inherent differences in the coordination behavior of ester and phosphonate groups may further boost reaction chemoselectivity *via* site-selective coordination. Gratifyingly, the addition of a catalytic amount (20 mol%) of Lewis acids indeed significantly improved the yield of 3a while simultaneously suppressing the formation of byproduct 4a (entries 6–12), and ZnBr_2_ gave the best reaction outcomes in terms of both chemoselectivity and yield (3a/4a > 20/1, 71% yield, entry 12). Ultimately, elevating ZnBr_2_ loading further improved reaction efficiency (entries 13–15), with 40 mol% ZnBr_2_ affording the desired product in 83% yield (entry 14). Moreover, ZnBr_2_ is a commercially available and extremely inexpensive reagent ($23 kg^−1^, https://www.leyan.com/). The enhanced chemoselectivity should originate from the much stronger coordination ability of the phosphate moiety relative to the ester group toward the zinc complex. In addition, other alkynes are less effective than the ester-substituted phosphonylated alkynes (listed in Table S2).

**Table 1:** Optimization of reaction conditions^a^

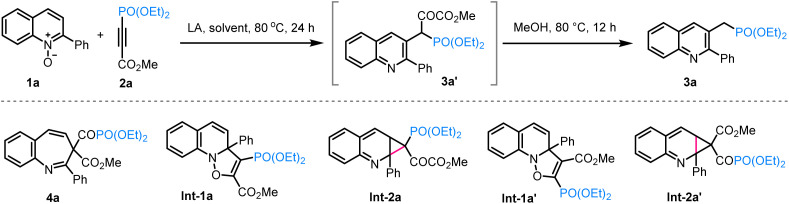				
Entry	Solvent	LA (20 mol%)	Yield of 3a (%)^a^	Ratio of 3a/4a^b^
1	THF	—	10	1 : 2
2	Toluene	—	23	1 : 1
3	DCE	—	16	1 : 2
4	MeCN	—	9	1 : 4
5	Dioxane	—	25	1 : 1
6	Dioxane	Cu(OTf)_2_	34	>20 : 1
7	Dioxane	Ni(OTf)_2_	35	10 : 1
8	Dioxane	Mg(OTf)_2_	40	7 : 1
9	Dioxane	Sc(OTf)_3_	40	>20 : 1
10	Dioxane	Zn(OTf)_2_	50	>20 : 1
11	Dioxane	ZnCl_2_	65	>20 : 1
12	Dioxane	ZnBr_2_	71	>20 : 1
13^c^	Dioxane	ZnBr_2_	78	>20 : 1
14^d^	Dioxane	ZnBr_2_	83	>20 : 1
15^e^	Dioxane	ZnBr_2_	82	>20 : 1

a Reaction conditions for 3a: 1a (0.1 mmol, 1 equiv), 2a (0.15 mmol, 1.5 equiv), LA (0.02 mmol, 20 mol%), solvent (c 0.1 M), 80 °C, 24 h, then MeOH (0.5 mL) was added, 80 °C, 12 h.

b Reaction conditions for 4a: 1a (0.1 mmol, 1 equiv), 2a (0.15 mmol, 1.5 equiv), LA (0.02 mmol, 20 mol%), solvent (1 mL), 80 °C, 24 h.

c ZnBr_2_ (0.03 mmol, 30 mol%).

d ZnBr_2_ (0.04 mmol, 40 mol%).

e ZnBr_2_ (0.05 mmol, 50 mol%). LA: Lewis acid.

### Substrate scope investigation

**Fig. 2:**
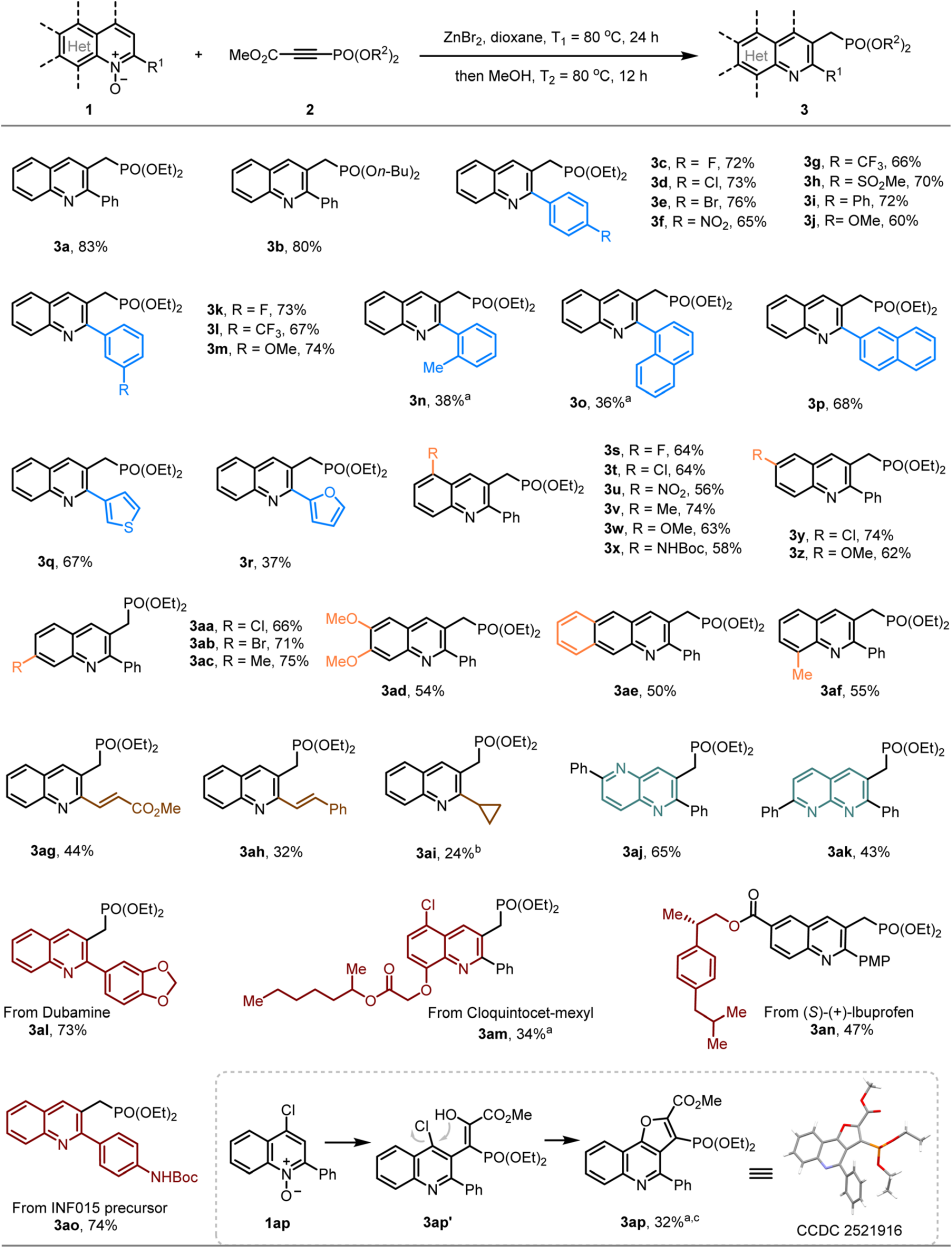
The substrate scope of quinolines. Standard conditions: *N*-oxides 1 (0.1 mmol), alkynes 2 (0.15 mmol), ZnBr_2_ (0.4 equiv), dioxane (*c* 0.1 M), 80 °C, 24 h; then MeOH (0.5 mL), 80 °C, 12 h. ^a^*T*_1_ = 120 °C. ^b^MeOH (0.5 mL) and CH_3_CO_2_H (0.1 mmol) were added. ^c^Without addition of MeOH.

With the optimized reaction conditions in hand, the substrate scope of this C3-methylenephosphonylation reaction was then investigated (Fig. 2). Unfortunately, the unsubstituted quinoline *N*-oxide afforded no target product (listed in Table S2); instead it underwent decomposition possibly due to the instability of the dearomatized intermediates involved in this cyclizative rearrangement. With respect to the ester moieties in substrate 2, modification of the alkyl substituent from ethyl to *n*-butyl resulted in the formation of product 3b with an 80% yield. Various C2-aryl substituents bearing halogens (F, Cl, and Br), electron-withdrawing groups (NO_2_, CF_3_, and SO_2_Me) and electron-donating groups (OMe and Ph) were well compatible with the optimized conditions, affording the desired products (3c–3m and 3p) in good yields (60–76%). Additionally, *ortho*-methylphenyl- and 1-naphthyl-substituted quinoline *N*-oxides underwent conversion to the expected products (3n and 3o) with diminished yields (38% and 36%, respectively), presumably due to steric hindrance. Heterocyclic substituents such as 2-thienyl and 2-furyl could undergo this reaction smoothly (3q–3r). Moreover, a wide range of substituents including functional groups (fluoro, chloro, methoxy, nitro, and *tert*-butyl carbamate) located at the C5, C6, C7, and C8 positions of the benzocyclic moiety were found to be well-tolerated (3s-3af). In addition to aryl groups, 2-alkenyl- and 2-alkyl-substituted azaarenes were also compatible with the reaction, albeit with slightly reduced yields (3ag–3ai). Other bicyclic azaarenes, such as 1,5-naphthyridine (1aj) and 1,8-naphthyridine (1ak), also underwent this C3-methylenephosphonylation reaction without event. Remarkably, the incorporation of methylenephosphonate into biologically active quinoline-containing drugs, such as dubamine, an INF015 precursor, a cloquintocet-mexyl derivative and an (*S*)–(+)-ibuprofen derivative, also resulted in moderate to good yields (3al–3ao, 34–74%), demonstrating the great potential of this protocol in site-selective late-stage modification of structurally complex molecules. Interestingly, the use of 4-chloro-substituted quinoline *N*-oxide led to the formation of the synthetically useful furo[3,2-*c*]quinoline scaffold (3ap),^63,64^ which is proposed to arise from the intramolecular oxo-cyclization of the enol intermediate 3ap’. This result implies the reaction pathway of this C3–H methylenephosphonylation.

**Fig. 3:**
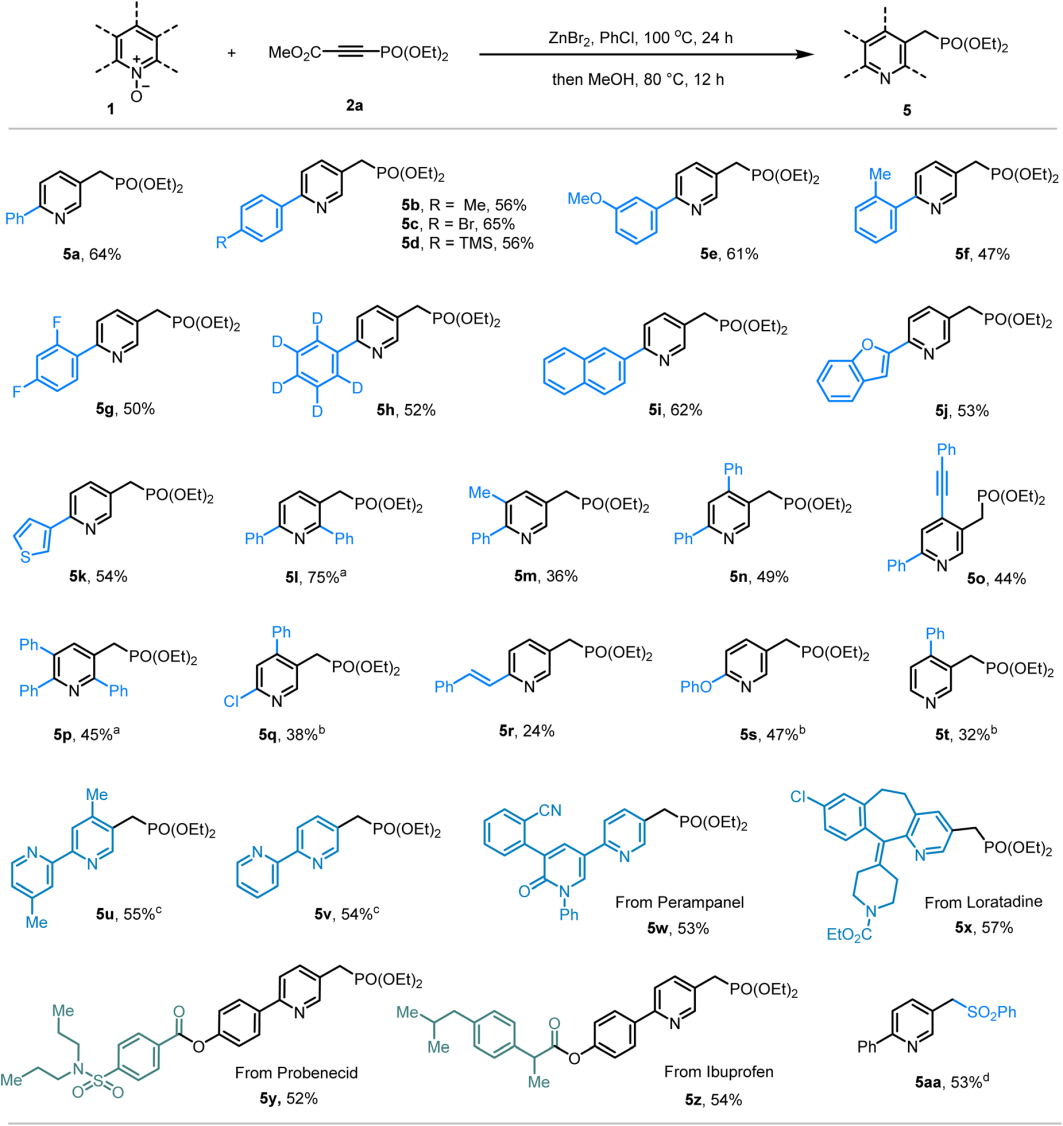
The substrate scope of pyridines. Standard conditions: *N*-oxide 1 (0.1 mmol), alkyne 2 (0.2 mmol), ZnBr_2_ (0.2 equiv), PhCl (*c* 0.1 M), 100 °C, 24 h; then MeOH (0.5 mL), 80 °C, 12 h. ^a^120 °C for the first step. ^b^Et_3_N (0.15 mmol) was added in the second step. ^c^Without ZnBr_2_. ^d^Using 2d instead of 2a.

The pyridine derivatives were further investigated as substrates in this reaction. They were found to exhibit relatively lower reactivity toward this transformation; accordingly, the reaction conditions were slightly modified to afford good results (see SI Table S1). Under optimal conditions (Fig. 3), 2-phenylpyridine *N*-oxide could react with alkyne 2a to give desired product 5a in 64% yield. And no regioisomeric C3-functionalized product was detected, which is likely attributed to steric hindrance during the initial 1,3-dipolar cycloaddition step. We also examined the unsubstituted pyridine *N*-oxide substrate under standard conditions and no desired product was produced (listed in Table S2). Other 2-aryl substituents bearing electron-donating, electron-withdrawing, and electron-neutral groups at different positions were also viable, affording products 5b–5f in moderate to good yields. Moreover, good yields were achieved when 2,4-difluorophenyl, deuterated phenyl (*d*_5_-phenyl), or aromatic heterocyclic substituents (2-benzofuranyl and 3-thienyl) were employed (5g–5k). 2,6-Diphenyl pyridine gave even higher yield (5l, 75%). Additionally, both disubstituted and trisubstituted pyridines with diverse functional groups were well-tolerated under the optimized conditions (5m–5q). Moreover, 2-alkenyl- and 2-phenoxy-substituted pyridines were also viable in this cyclizative rearrangement protocol, affording the desired products (5r and 5s) in moderate yields. To our delight, bipyridines, which serve as versatile ligands in various catalytic reactions,^65^ were smoothly converted to C3-methylenephosphonated derivatives in good yields (5u, 5v). Alternatively, 4-phenylpyridine *N*-oxide afforded 5t in 32% yield, and 3-phenylpyridine-derived *N*-oxide was also evaluated and only a trace amount of desired product was detected (listed in Table S2). Furthermore, late-stage C–H functionalization of complex drugs bearing diverse sensitive functional groups was achieved. Specifically, perampanel-, loratadine-, probenecid- and ibuprofen-derived *N*-oxides delivered the corresponding products efficiently (5w–5z). To further verify the generality of this cyclizative rearrangement strategy, we investigated a sulfur-containing alkyne analogue bearing a benzenesulfonyl group. Under standard conditions, the reaction with 2-phenylpyridine *N*-oxide successfully delivered the target methylenesulfonated product 5aa in 53% yield.

### Synthetic transformations

**Fig. 4:**
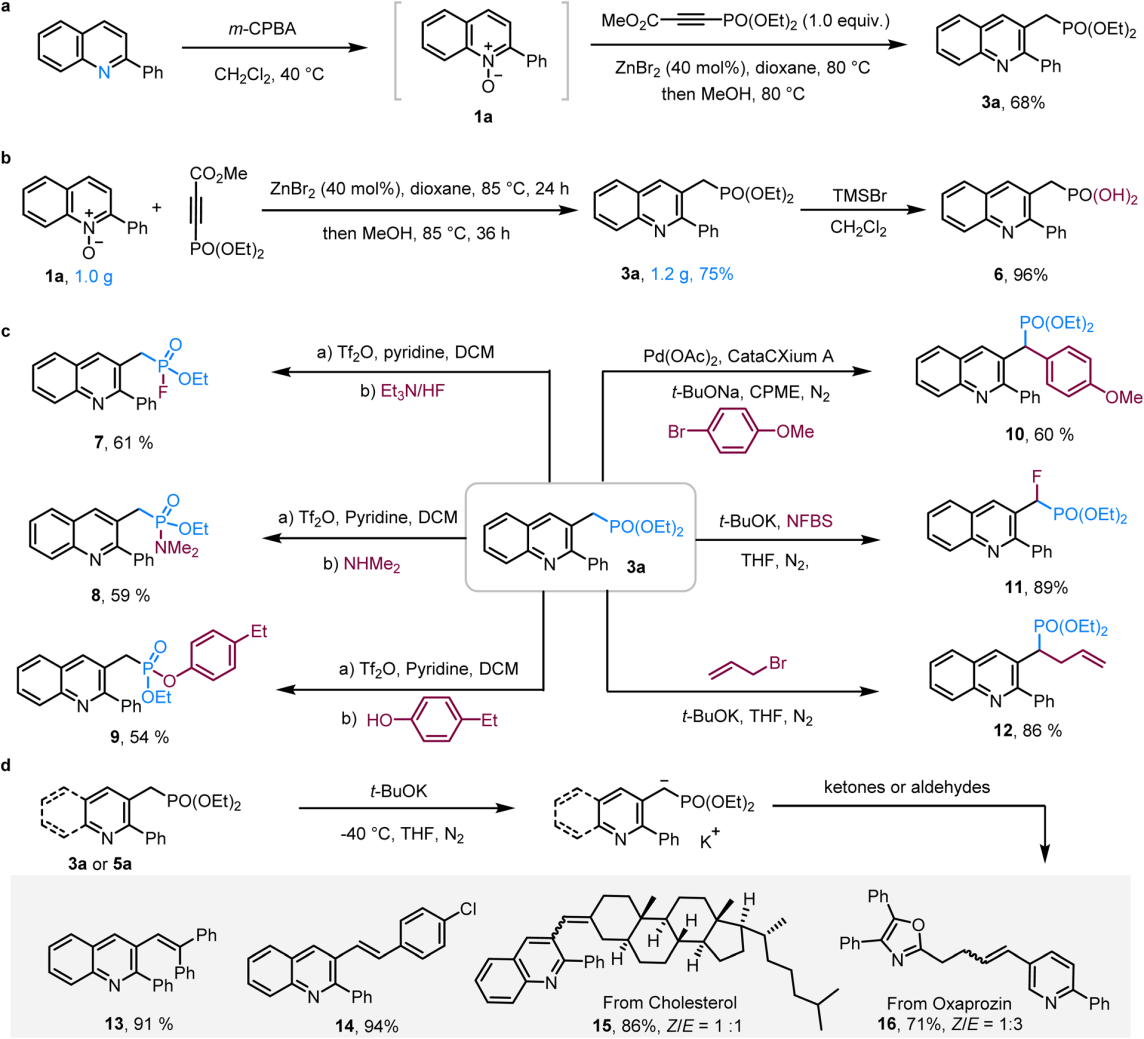
Synthetic utility. (a) One-pot synthesis of 3a from 2-phenylquinoline. (b) Gram-scale synthesis of 3a. (c) Synthetic transformations of 3a. (d) Horner–Wadsworth–Emmons reactions of methylenephosphonated azaarenes 3a and 5a. CataCxium A: butyldi-1-adamantylphosphine; CPME: cyclopentyl methyl ether; NFBS: *N*-fluorobenzenesulfonimide.

To further expand the utility of this protocol, the one-pot synthesis of 3a directly from 2-phenylquinoline was investigated. This approach proved feasible, affording desired product 3a with high efficiency (68% yield, Fig. 4a). Moreover, the gram-scale reaction of 1a with 2a afforded 3a in 75% yield, demonstrating the scalability of this method (Fig. 4b).

Hydrolysis of 3a with TMSBr gave its phosphoric acid 6 in excellent yield. Subsequently, a series of synthetic transformations were conducted to explore the synthetic versatility of product 3a (Fig. 4c). Fluorinated phosphonate 7 was obtained *via* Tf_2_O activation followed by nucleophilic substitution with Et_3_N·HF as the fluoride source. Likewise, the reaction of 3a with dimethylamine and 4-ethylphenol afforded the corresponding products 8 and 9, respectively. In addition, phosphonate 3a underwent a palladium-catalyzed cross-coupling reaction, affording α-aryl phosphonate 10 in 60% yield. Furthermore, fluoro and allyl groups were efficiently installed at the α-position of methylenephosphonate 3a to afford products 11 and 12 in high yields (Fig. 4c). Finally, the potential of *meta*-methylenephosphonated azaarenes as Horner–Wadsworth–Emmons (HWE) reagents was evaluated.^66^ Both benzophenone and 4-chlorobenzaldehyde were smoothly converted to C3-alkenylquinoline derivatives 13 and 14, respectively, with good efficiency. Compound 3a could also react with a cholesterol-derived cyclic ketone, furnishing alkenyl product 15 in high yield. When the biologically active oxaprozin-derived alkyl aldehyde was engaged in the HWE reaction, the corresponding alkenyl pyridine 16 was likewise obtained efficiently (Fig. 4d). Collectively, these transformations showcase the practicality of our protocol as well as the great potential of the products to serve as important building blocks for further diversifications.

### Mechanism studies

**Fig. 5:**
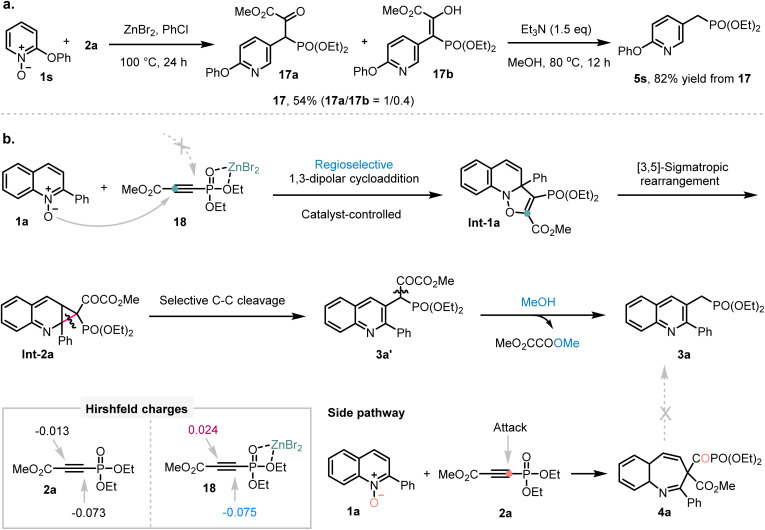
(a) Control experiments. (b) The possible reaction pathway.

Control experiments were performed to provide insights into the reaction mechanism. Using 2-phenoxypyridine *N*-oxide 1s as the substrate to react with alkyne 2a under standard conditions, the key intermediate 17 was isolated in 54% yield. ^1^H and ^31^P NMR analysis revealed a mixture of ketone and enolate tautomers with a ratio of 1 : 0.4. Furthermore, treating 17 with Et_3_N in MeOH at 80 °C afforded desired 5s in high yield (Fig. 5a). These results are consistent with the observations from the reaction of quinoline 1a in Table 1. Moreover, the formation of 3ap*via* the *in situ* trapping of the key enol intermediate (3ap’) from 4-Cl-substituted quinoline 1ap (Fig. 2) is also consistent with the above experimental results.

On the basis of experimental results (Table 1 and Fig. 2, 3 and 5a) and literature precedents,^22,24,26^ a plausible reaction pathway is proposed in Fig. 5b. ZnBr_2_ exhibits a stronger coordination with the phosphonate moiety, which not only activates alkyne 2a but also enhances the electron density difference between the two Csp centers of the alkyne. Coordination of ZnBr_2_ to the phosphonate group activates the alkyne moiety, as evidenced by changes in the Hirshfeld charges at the α and β positions of 2a before and after coordination. This further promotes the selective nucleophilic attack of the oxyanion from 1a, enabling a highly regioselective 1,3-dipolar cycloaddition process (Int-1a). A sequential cascade involving [3,5]-sigmatropic rearrangement and external C–C bond cleavage *via* intermediate Int-2 afforded key intermediate 3a’ bearing a reactive ketoester moiety. Finally, GC-MS analysis of the crude reaction mixture detected a signal assigned to dimethyl oxalate (MeO_2_CCO_2_Me). This result reveals that the ketoester group was removed with the aid of excess methanol, affording the desired product 3a. On the other hand, in the absence of the Lewis acid catalyst (ZnBr_2_), a side reaction pathway occurred, arising from the α-attack of phosphonated alkyne 2a and furnishing the benzazepine product 4a, which could not be further converted to 3a. The regioselective cycloaddition controlled by the Lewis acid catalyst, along with the subsequent two selective C–C bond cleavages, collectively ensures the chemoselectivity of the entire reaction.

## Conclusions

In conclusion, we report the first *meta*-C–H methylenephosphonylation of azaarenes *via* Lewis acid-catalyzed regioselective intermolecular cyclizative rearrangement, using readily available azaarene *N*-oxides and phosphonate-substituted alkynes as substrates. ZnBr_2_ is indispensable for this transformation, ensuring both high reactivity and excellent chemoselectivity. This protocol features broad substrate scope, remarkable functional group compatibility, and high efficiency in the late-stage functionalization of drugs and ligands. The resulting products serve as versatile synthetic linchpins for divergent synthesis of phosphorus-containing compounds and alkenylated azaarenes. This method provides a valuable perspective for the introduction of other heteroatoms onto azaarenes.

## Author contributions

H. W. and Y.-P. H. conceived and designed the project. D. L. conducted all experimental investigations and data collection. F.-Z. L. performed the DFT theoretical calculations. D. L. drafted the original manuscript. H. W. and Y.-P. H. supervised the whole project and revised the manuscript. All authors discussed the results and commented on the final manuscript.

## Conflicts of interest

There are no conflicts to declare.

## Supplementary Material

SC-OLF-D6SC03456J-s001

SC-OLF-D6SC03456J-s002

## Data Availability

Supplementary information (SI): further details of the experimental procedure, copies of ^1^H, ^13^C ^19^F and ^31^P NMR spectra, X-ray crystallographic data and computational details. See DOI: https://doi.org/10.1039/d6sc03456j. CCDC 2521916 contains the supplementary crystallographic data for this paper.^67^
