# Supplementary material for: C3–H methylenephosphonylation of azaarenes enabled by catalyst-controlled regioselective cyclizative rearrangement
Source: Chem Sci. 2026 Jun 11;17(29):14432–41. doi: 10.1039/d6sc03456j (PMC13280453; doi:10.1039/d6sc03456j)
Supplement: SC-017-D6SC03456J-s001 [file SC-017-D6SC03456J-s001.pdf]

Supplementary Information (SI) for Chemical Science.
This journal is © The Royal Society of Chemistry 2026

C3-H Methylenephosphonylation of Azaarenes Enabled by Catalyst-controlled Regioselective Cyclizative Rearrangement

Table of Contents

1. General information....................2
2. Optimization of the reaction conditions....................3
3. General procedure for *meta*-C–H methylenephosphonylation of azaarenes....................5
4. One-pot synthesis of **3a** from 2-phenylquinoline and gram-scale reaction....................42
5. Synthetic applications....................43
6. Mechanistic studies....................53
7. Density functional theory (DFT) studies....................56
8. Substrates preparation and characterization....................59
9. References....................61
10. Crystallographic data....................63
11. Copies of NMR spectra....................65

1. General information

NMR spectra were recorded on Brucker AV 400 MHz spectrometer. Chemical shifts are given in ppm. ^{19}F NMR and ^{31}P NMR spectra are calibrated using absolute referencing to the ^{1}H NMR spectrum, as suggested by IUPAC. (Harris et al., 2001). The spectra are calibrated to the residual ^{1}H and ^{13}C signals of the solvents. Signal multiplicities are reported as follows: s (singlet), d (doublet), t (triplet), q (quartet), quint (quintet), m (multiplet), and combinations thereof (e.g., dd, dt). Mass spectrometry analysis was carried out using an electrospray spectrometer Waters Micromass Q-TOF Premier Mass Spectrometer. Melting points were measured with SGW X-4 micro melting point apparatus.

Materials and Methods: Unless otherwise noted, starting materials were purchased from commercial sources Adamas-Beta®, Leyan, Shanghai Bide pharmatech Co. Ltd., Shanghai Haohong Scientific Co. Ltd, J&K®, Macklin, Aladdin®, Energy chemical, and used without any purification. More sensitive compounds were stored in a desiccator or in a glove box if required. Solvents were purchased in HPLC quality, degassed by purging thoroughly with nitrogen and dried over activated molecular sieves of appropriate size. Alternatively, they were purged with argon and passed through alumina columns in a solvent purification system (Innovative Technology). Reactions were monitored by thin layer chromatography (TLC) using Xinnuo TLC silica gel 60 F254. Compounds were visualized by UV-light at 254 nm and by dipping the plates in an aqueous potassium permanganate solution followed by heating. Flash column chromatography was performed over silica gel (230- 400 mesh). Zinc bromide (Cat No. 1042696) was purchased from Leyan, Shanghai, China.

2. Optimization of the reaction conditions

Table S1: Condition optimization for *meta*-C-H methylenephosphonylation of pyridines[a]

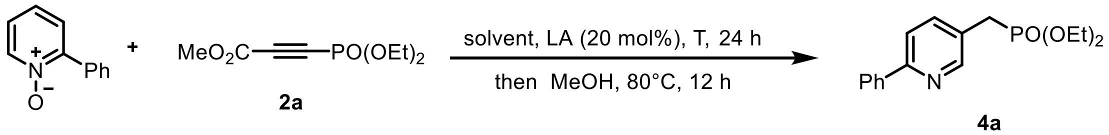

Entry	T (ºC)	Solvent	LA	Yield (%)[b]
1	90	dioxane	$ZnBr_2$	56
2	90	THF	$ZnBr_2$	56
3	90	EtOH	$ZnBr_2$	45
4	90	DCE	$ZnBr_2$	48
5	90	MeCN	$ZnBr_2$	46
6	90	MTBE	$ZnBr_2$	37
7	90	DMF	$ZnBr_2$	trace
8	90	DMSO	$ZnBr_2$	trace
9	90	$PhCF_3$	$ZnBr_2$	50
10	90	$PhCH_3$	$ZnBr_2$	53
11	90	PhCl	$ZnBr_2$	60
12	90	PhCl	$Mg(OTf)_2$	56
13	90	PhCl	$Sc(OTf)_3$	52
14	90	PhCl	$Ni(OTf)_2$	47
15	90	PhCl	$Cu(OTf)_2$	10
**16**	**100**	**PhCl**	**$ZnBr_2$**	**64**
17[c]	100	PhCl	$ZnBr_2$	62

[a]Reaction conditions: 2-phenylpyridine *N*-oxide (0.1 mmol, 1 equiv), **2a** (0.2 mmol, 2 equiv), LA (0.02 mmol, 20 mol%), solvent (1 mL), 90 ºC, 24 h, then add MeOH (0.5 mL), 80 ºC, 12 h. [b]Isolated yield. [c]$ZnBr_2$ (0.04 mmol, 40 mol%).

Table S2: Failed substrates

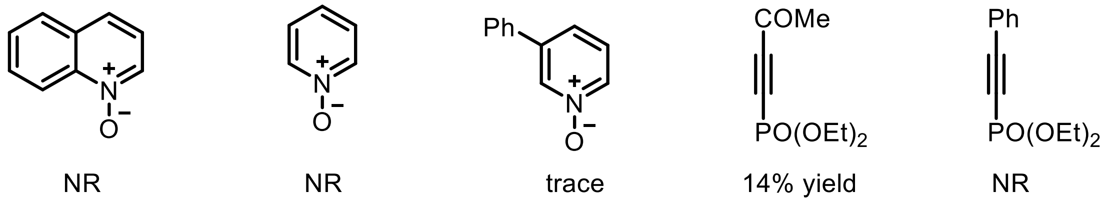

3. General procedure for *meta*-C–H methylenephosphonylation of azaarenes

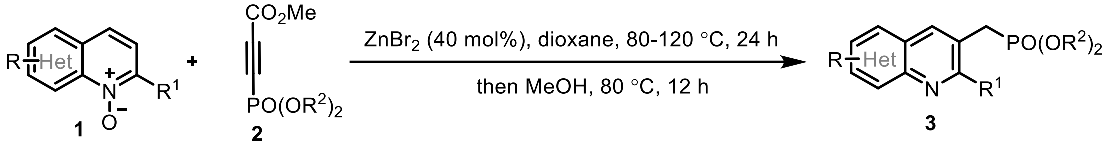

Procedure A: A mixture of azaarene **1** (0.1 mmol, 1.0 equiv), alkyne **2** (0.15 mmol, 1.5 equiv) and $ZnBr_2$ (0.04 mmol, 40 mol%) was dissolved in dioxane (1 mL) and stirred at indicated temperature (T_1) for 24 h. After cooling to room temperature, MeOH (0.5 mL) was added to the above reaction mixture, and the reaction was stirred at 80 °C for 12 h. When the reaction was completed and cooled to room temperature, the solvent was removed under reduced pressure, and the residue was purified by column chromatography on silica gel using PE/EtOAc as eluent to afford product **3**.

Procedure B: An oven-dried vial was charged with azaarene **1ap** (0.1 mmol, 1.0 equiv), alkyne **2a** (0.15 mmol, 1.5 equiv), and $ZnBr_2$ (0.04 mmol, 40 mol%), then the mixture was stirred in dioxane (1 mL) at 120 ºC for 24 h. Upon completion of the reaction, the solvent was removed under reduced pressure, and the residue was purified by column chromatography on silica gel using PE/EtOAc as eluent to afford product **3ap**.

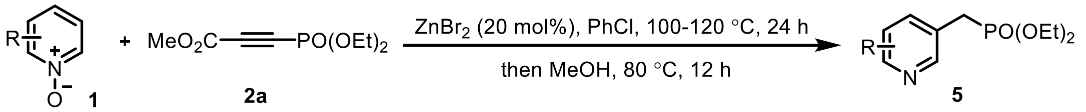

Procedure C: A mixture of azaarene **1** (0.1 mmol, 1.0 equiv), alkyne **2a** (0.2 mmol, 2.0 equiv) and $ZnBr_2$ (0.02 mmol, 20 mol%) was dissolved in PhCl (1 mL) and stirred at indicated temperature (T_2) for 24 h. After cooling to room temperature, MeOH (0.5 mL) was added to the above reaction mixture, and the reaction was stirred at 80 °C for 12 h. When the reaction was completed and cooled to room temperature, the solvent was removed under reduced pressure, and the residue was purified by column chromatography on silica gel using PE/EtOAc as eluent to afford product **5**.

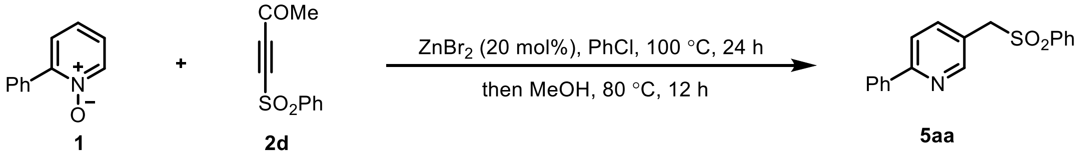

Procedure D: A mixture of azaarene **1** (0.1 mmol, 1.0 equiv), alkyne **2d** (0.2 mmol, 2.0 equiv) and $ZnBr_2$ (0.02 mmol, 20 mol%) was dissolved in PhCl (1 mL) and stirred at 100 ºC for 24 h. After cooling to room temperature, MeOH (0.5 mL) was added to the above reaction mixture, and the reaction was stirred at 80 °C for 12 h. When the reaction was completed and cooled to room temperature, the solvent was removed under reduced pressure, and the residue was purified by column chromatography on silica gel using PE/EtOAc as eluent to afford product **5aa**.

diethyl ((2-phenylquinolin-3-yl)methyl)phosphonate (3a)

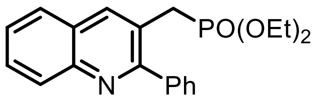

Following procedure A: T_1 = 80 ºC, 29.6 mg, yellow oil, 83% yield.

^{1}H NMR (400 MHz, Chloroform-*d*) 8.41 (d, *J* = 3.4 Hz, 1H), 8.12 (d, *J* = 8.5 Hz, 1H), 7.85 (d, *J* = 8.2 Hz, 1H), 7.70 (t, *J* = 6.8, 1H), 7.66 – 7.60 (m, 2H), 7.57 – 7.42 (m, 4H), 4.02 – 3.92 (m, 4H), 3.37 (d, *J* = 22.2 Hz, 2H), 1.21 (t, *J* = 7.1 Hz, 6H).

^{13}C NMR (101 MHz, Chloroform-*d*) δ 160.4 (d, *J* = 7.4 Hz), 146.9 (d, *J* = 2.5 Hz), 140.3, 138.1 (d, *J* = 6.1 Hz), 129.7 (d, *J* = 1.5 Hz), 129.4 (d, *J* = 1.5 Hz), 129.3, 128.6, 128.5, 127.4 (d, *J* = 1.3 Hz), 127.3 (d, *J* = 3.2 Hz), 126.9, 123.6 (d, *J* = 8,7 Hz), 62.3 (d, *J* = 6.7 Hz), 30.0 (d, *J* = 139.2 Hz), 16.5 (d, *J* = 6.1 Hz).

^{31}P NMR (162 MHz, Chloroform-*d*) δ 25.9.

HRMS (ESI) m/z: $[M + H]^+$ Calcd for $C_{20}H_{23}NO_3P^+$ 356.1410; Found 356.1422.

dibutyl ((2-phenylquinolin-3-yl)methyl)phosphonate (3b)

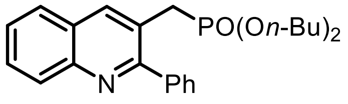

Following procedure A: T_1 = 80 ºC, 32.9 mg, colorless oil, 80% yield.

^{1}H NMR (400 MHz, Chloroform-*d*) δ 8.40 (d, *J* = 3.4 Hz, 1H), 8.12 (d, *J* = 8.5 Hz, 1H), 7.83 (d, *J* = 8.2 Hz, 1H), 7.73 – 7.66 (m, 1H), 7.63 (dd, *J* = 7.9, 1.6 Hz, 2H), 7.56 – 7.40 (m, 4H), 3.94 – 3.85 (m, 4H), 3.37 (d, *J* = 22.2 Hz, 2H), 1.59 – 1.47 (m, 4H), 1.31 – 1.22 (m, 4H), 0.84 (t, *J* = 7.4 Hz, 6H).

^{13}C NMR (101 MHz, Chloroform-*d*) δ 160.3 (d, *J* = 7.5 Hz), 146.9 (d, *J* = 2.5 Hz), 140.3, 138.1 (d, *J* = 6.0 Hz), 129.7 (d, *J* = 1.6 Hz), 129.4 (d, *J* = 1.4 Hz), 129.3, 128.6, 128.5, 127.3 (d, *J* = 1.3 Hz), 127.2 (d, *J* = 3,2 Hz), 126.8, 123.6 (d, *J* = 8.5 Hz), 66.0 (d, *J* = 7.0 Hz), 32.5 (d, *J* = 6.0 Hz), 29.8 (d, *J* = 139.3 Hz), 18.7, 13.6.

^{31}P NMR (162 MHz, Chloroform-*d*) δ 25.9.

HRMS (ESI) m/z: $[M + H]^+$ Calcd for $C_{24}H_{31}NO_3P^+$ 412.2042; Found: 412.2042.

diethyl ((2-(4-fluorophenyl)quinolin-3-yl)methyl)phosphonate (3c)

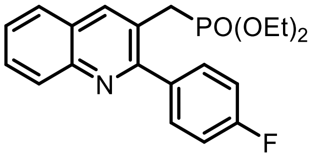

Following procedure A: T_1 = 80 ºC, 26.8 mg, yellow oil, 72% yield.

^{1}H NMR (400 MHz, Chloroform-*d*) δ 8.38 (d, *J* = 3.5 Hz, 1H), 8.10 (d, *J* = 8.5 Hz, 1H), 7.87 – 7.80 (m, 1H), 7.75 – 7.60 (m, 3H), 7.54 (ddd, *J* = 8.1, 6.8, 1.2 Hz, 1H), 7.24 – 7.13 (m, 2H), 4.04 – 3.96 (m, 4H), 3.33 (d, *J* = 22.2 Hz, 2H), 1.22 (t, *J* = 7.1 Hz, 6H).

^{13}C NMR (101 MHz, Chloroform-*d*) δ 163.0 (d, *J* = 248.7 Hz), 159.3 (d, *J* = 7.1 Hz), 146.9 (d, *J* = 2.4 Hz), 138.2 (d, *J* = 5.9 Hz), 136.3 (d, *J* = 3.5 Hz), 131.3 (d, *J* = 8.3 Hz), 129.9 (d, *J* = 1.5 Hz), 129.3 (d, *J* = 1.5 Hz), 127.4 (d, *J* = 1.4 Hz), 127.3 (d, *J* = 3.2 Hz), 127.0, 123.5 (d, *J* = 8.7 Hz), 115.5 (d, *J* = 21.6 Hz), 62.4 (d, *J* = 6.8 Hz), 30.1 (d, *J* = 139.5 Hz), 16.5 (d, *J* = 5.9 Hz).

^{19}F NMR (376 MHz, Chloroform-*d*) δ -113.4.

^{31}P NMR (162 MHz, Chloroform-*d*) δ 25.8.

HRMS (ESI) m/z: $[M + H]^+$ Calcd for $C_{20}H_{22}FNO_3P^+$ 374.1316; Found: 374.1323.

diethyl ((2-(4-chlorophenyl)quinolin-3-yl)methyl)phosphonate (3d)

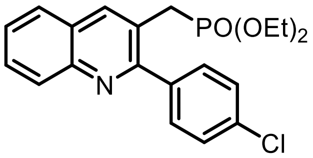

Following procedure A: T_1 = 80 ºC, 28.5 mg, yellow oil, 73% yield.

^{1}H NMR (400 MHz, Chloroform-*d*) δ 8.39 (d, *J* = 3.5 Hz, 1H), 8.10 (d, *J* = 8.5 Hz, 1H), 7.85 (s, 1H), 7.73 – 7.68 (m, 1H), 7.64 – 7.58 (m, 2H), 7.57 – 7.52 (m, 1H), 7.51 – 7.45

(m, 2H), 4.05 – 3.95 (m, 4H), 3.32 (d, J = 22.2 Hz, 2H), 1.22 (t, J = 7.1 Hz, 6H).

^{13}C NMR (101 MHz, Chloroform-*d*) δ 159.1 (d, J = 7.2 Hz), 146.9 (d, J = 2.5 Hz), 138.7, 138.3 (d, J = 6.1 Hz), 134.7, 130.9, 129.9 (d, J = 1.5 Hz), 129.4 (d, J = 1.6 Hz), 128.8, 127.4 (d, J = 1.4 Hz), 127.3 (d, J = 3.2 Hz), 127.1, 123.4 (d, J = 8.6 Hz), 62.4 (d, J = 6.9 Hz), 30.1 (d, J = 139.6 Hz), 16.5 (d, J = 6.0 Hz).

^{31}P NMR (162 MHz, Chloroform-*d*) δ 25.7.

HRMS (ESI) m/z: $[M + H]^+$ Calcd for $C_{20}H_{22}ClNO_3P^+$ 390.1020; Found: 390.1025.

diethyl ((2-(4-bromophenyl)quinolin-3-yl)methyl)phosphonate (3e)

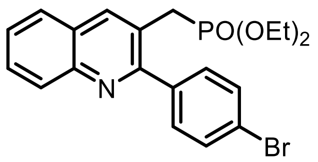

Following procedure A: T_1 = 80 ºC, 33.3 mg, yellow oil, 76% yield.

^{1}H NMR (400 MHz, Chloroform-*d*) δ 8.39 (d, J = 3.5 Hz, 1H), 8.09 (d, J = 8.5 Hz, 1H), 7.84 (d, J = 8.2 Hz, 1H), 7.70 (t, J = 7.7 Hz, 1H), 7.63 (d, J = 8.1 Hz, 2H), 7.58 – 7.50 (m, 3H), 4.04 – 3.96 (m, 4H), 3.32 (d, J = 22.2 Hz, 2H), 1.22 (t, J = 7.1 Hz, 6H).

^{13}C NMR (101 MHz, Chloroform-*d*) δ 159.1 (d, J = 7.3 Hz), 146.9 (d, J = 2.5 Hz), 139.2, 138.3 (d, J = 6.1 Hz), 131.7, 131.1, 129.9 (d, J = 1.5 Hz), 129.3 (d, J = 1.5 Hz), 127.4 (d, J = 1.3 Hz), 127.3 (d, J = 3.2 Hz), 127.1, 123.3 (d, J = 8.6 Hz), 123.0, 62.4 (d, J = 6.8 Hz), 30.0 (d, J = 139.5 Hz), 16.5 (d, J = 6.0 Hz).

^{31}P NMR (162 MHz, Chloroform-*d*) δ 25.7.

HRMS (ESI) m/z: $[M + H]^+$ Calcd for $C_{20}H_{22}BrNO_3P^+$ 434.0515; Found: 434.0513.

diethyl ((2-(4-nitrophenyl)quinolin-3-yl)methyl)phosphonate (3f)

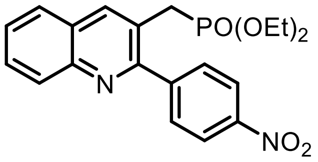

Following procedure A: T_1 = 80 ºC, 26.3 mg, white solid, 65% yield. M.p = 90 – 92 ºC.

^{1}H NMR (400 MHz, Chloroform-*d*) δ 8.41 (d, J = 3.5 Hz, 1H), 8.39 – 8.30 (m, 2H), 8.10 (d, J = 8.5 Hz, 1H), 7.90 – 7.86 (m, 3H), 7.74 (t, J = 7.7 Hz, 1H), 7.59 (t, J = 7.6 Hz, 1H), 4.06 – 3.97 (m, 4H), 3.29 (d, J = 22.2 Hz, 2H), 1.23 (t, J = 7.1 Hz, 6H).

¹³C NMR (101 MHz, Chloroform-*d*) δ 157.9 (d, *J* = 7.0 Hz), 147.9, 146.9 (d, *J* = 2.5 Hz), 146.7, 138.7 (d, *J* = 6.0 Hz), 130.7, 130.3 (d, *J* = 1.5 Hz), 129.4 (d, *J* = 1.6 Hz), 127.6, 127.5, 127.47 (d, *J* = 1.4 Hz), 123.7, 123.1 (d, *J* = 8.6 Hz), 62.5 (d, *J* = 6.8 Hz), 30.1 (d, *J* = 140.0 Hz), 16.5 (d, *J* = 5.9 Hz).

³¹P NMR (162 MHz, Chloroform-*d*) δ 25.3.

HRMS (ESI) m/z: $[M + H]^+$ Calcd for $C_{20}H_{22}N_2O_5P^+$ 401.1261; Found: 401.1262.

diethyl ((2-(4-(trifluoromethyl)phenyl)quinolin-3-yl)methyl)phosphonate (3g)

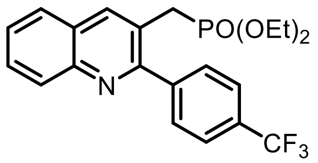

Following procedure A: T_1 = 80 ºC, 27.9 mg, yellow oil, 66% yield.

¹H NMR (400 MHz, Chloroform-*d*) δ 8.42 (d, *J* = 3.5 Hz, 1H), 8.11 (d, *J* = 8.5 Hz, 1H), 7.86 (d, *J* = 8.1 Hz, 1H), 7.81 – 7.70 (m, 5H), 7.57 (t, *J* = 7.5 Hz, 1H), 4.05 – 3.97 (m, 4H), 3.32 (d, *J* = 22.2 Hz, 2H), 1.23 (t, *J* = 7.1 Hz, 6H).

¹³C NMR (101 MHz, Chloroform-*d*) δ 158.8 (d, *J* = 7.2 Hz), 146.9 (d, *J* = 2.5 Hz), 143.9, 138.4 (d, *J* = 6.1 Hz), 130.6 (q, *J* = 32.7 Hz), 130.1, 129.9, 129.4, 127.5, 127.4, 127.3, 125.6 (q, *J* = 3.5 Hz), 124.2 (q, *J* = 271.1 Hz), 123.3 (d, *J* = 8.7 Hz), 62.4 (d, *J* = 6.9 Hz), 30.1 (d, *J* = 139.8 Hz), 16.5 (d, *J* = 5.9 Hz).

¹⁹F NMR (376 MHz, Chloroform-*d*) δ -62.6.

³¹P NMR (162 MHz, Chloroform-*d*) δ 25.5.

HRMS (ESI) m/z: $[M + H]^+$ Calcd for $C_{21}H_{22}F_3NO_3P^+$ 424.1284; Found: 424.1281.

diethyl ((2-(4-(methylsulfonyl)phenyl)quinolin-3-yl)methyl)phosphonate (3h)

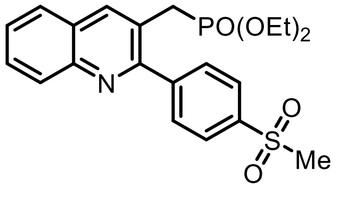

Following procedure A: T_1 = 80 ºC, 30.6 mg, white solid, 70% yield. M.p = 134 – 135 ºC.

¹H NMR (400 MHz, Chloroform-*d*) δ 8.42 (d, *J* = 3.4 Hz, 1H), 8.10 – 8.07 (m, 3H),

7.95 – 7.88 (m, 2H), 7.86 (d, *J* = 8.2 Hz, 1H), 7.76 – 7.70 (m, 1H), 7.60 – 7.56 (m, 1H), 4.06 – 3.95 (m, 4H), 3.28 (d, *J* = 22.2 Hz, 2H), 3.09 (s, 3H), 1.23 (t, *J* = 7.1 Hz, 6H).

^{13}C NMR (101 MHz, Chloroform-*d*) δ 158.2 (d, *J* = 7.2 Hz), 146.9 (d, *J* = 2.5 Hz), 145.8, 140.4, 138.6 (d, *J* = 6.0 Hz), 130.6, 130.2 (d, *J* = 1.5 Hz), 129.4 (d, *J* = 1.5 Hz), 127.7, 127.5, 127.48, 127.47, 123.2 (d, *J* = 8.6 Hz), 62.5 (d, *J* = 6.7 Hz), 44.7, 30.0 (d, *J* = 139.9 Hz), 16.5 (d, *J* = 6.0 Hz).

^{31}P NMR (162 MHz, Chloroform-*d*) δ 25.3.

HRMS (ESI) m/z: $[M + H]^+$ Calcd for $C_{21}H_{25}NO_5PS^+$ 434.1186; Found: 434.1187.

diethyl ((2-([1,1'-biphenyl]-4-yl)quinolin-3-yl)methyl)phosphonate (3i)

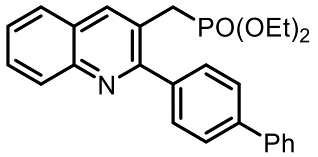

Following procedure A: T_1 = 80 ºC, 31.2 mg, yellow oil, 72% yield.

^{1}H NMR (400 MHz, Chloroform-*d*) δ 8.43 (d, *J* = 3.5 Hz, 1H), 8.14 (d, *J* = 8.5 Hz, 1H), 7.86 (d, *J* = 8.2 Hz, 1H), 7.69 (d, *J* = 33.1 Hz, 7H), 7.56 (t, *J* = 7.5 Hz, 1H), 7.48 (t, *J* = 7.5 Hz, 2H), 7.38 (t, *J* = 7.3 Hz, 1H), 4.05 – 3.97 (m, 4H), 3.44 (d, *J* = 22.2 Hz, 2H), 1.23 (t, *J* = 7.1 Hz, 6H).

^{13}C NMR (101 MHz, Chloroform-*d*) δ 160.0 (d, *J* = 7.3 Hz), 147.0 (d, *J* = 2.5 Hz), 141.4, 140.8, 139.2, 138.2 (d, *J* = 6.0 Hz), 129.8, 129.76, 129.4, 129.0, 127.6, 127.4, 127.36, 127.3, 127.2, 126.9, 123.6 (d, *J* = 8.7 Hz), 62.3 (d, *J* = 6.8 Hz), 30.1 (d, *J* = 139.2 Hz), 16.5 (d, *J* = 6.1 Hz).

^{31}P NMR (162 MHz, Chloroform-*d*) δ 25.9.

HRMS (ESI) m/z: $[M + H]^+$ Calcd for $C_{26}H_{27}NO_3P^+$ 432.1723; Found: 432.1722.

diethyl ((2-(4-methoxyphenyl)quinolin-3-yl)methyl)phosphonate (3j)

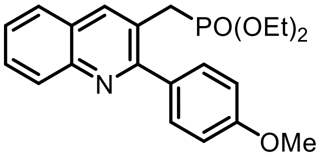

Following procedure A: T_1 = 80 ºC, 23.1 mg, yellow oil, 60% yield.

^{1}H NMR (400 MHz, Chloroform-*d*) δ 8.38 (d, *J* = 3.4 Hz, 1H), 8.10 (d, *J* = 8.5 Hz, 1H),

7.82 (d, J = 8.1 Hz, 1H), 7.68 (ddt, J = 8.3, 7.0, 1.2 Hz, 1H), 7.62 – 7.57 (m, 2H), 7.52 (ddd, J = 8.1, 6.8, 1.2 Hz, 1H), 7.05 – 6.99 (m, 2H), 4.03 – 3.87 (m, 4H), 3.87 (s, 3H), 3.39 (d, J = 22.2 Hz, 2H), 1.22 (t, J = 7.1 Hz, 6H).

^{13}C NMR (101 MHz, Chloroform-*d*) δ 160.1 (d, J = 7.3 Hz), 159.9, 147.0 (d, J = 2.5 Hz), 138.1 (d, J = 6.0 Hz), 132.8, 130.8, 129.6 (d, J = 1.5 Hz), 129.3 (d, J = 1.5 Hz), 127.4 (d, J = 1.4 Hz), 127.1 (d, J = 3.2 Hz), 126.7, 123.6 (d, J = 8.5 Hz), 114.0, 62.3 (d, J = 6.8 Hz), 55.5, 30.0 (d, J = 139.2 Hz), 16.5 (d, J = 6.1 Hz).

^{31}P NMR (162 MHz, Chloroform-*d*) δ 26.1.

HRMS (ESI) m/z: $[M + H]^+$ Calcd for $C_{21}H_{25}NO_4P^+$ 386.1516; Found: 386.1515.

diethyl ((2-(3-fluorophenyl)quinolin-3-yl)methyl)phosphonate (3k)

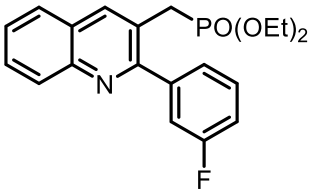

Following procedure A: T_1 = 80 ºC, 27.5 mg, yellow oil, 73% yield.

^{1}H NMR (400 MHz, Chloroform-*d*) δ 8.40 (d, J = 3.5 Hz, 1H), 8.11 (d, J = 8.5 Hz, 1H), 7.85 (d, J = 8.1 Hz, 1H), 7.71 (ddt, J = 8.3, 7.0, 1.3 Hz, 1H), 7.56 (ddd, J = 8.1, 6.9, 1.2 Hz, 1H), 7.50 – 7.34 (m, 3H), 7.20 – 7.08 (m, 1H), 4.07 – 3.94 (m, 4H), 3.34 (d, J = 22.2 Hz, 2H), 1.22 (t, J = 7.1 Hz, 6H).

^{13}C NMR (101 MHz, Chloroform-*d*) δ 162.8 (d, J = 246.9 Hz), 158.9 (dd, J = 7.1, 2.3 Hz), 146.8 (d, J = 2.5 Hz), 142.4 (d, J = 7.4 Hz), 138.4 (d, J = 6.0 Hz), 130.2 (d, J = 8.3 Hz), 129.9 (d, J = 1.5 Hz), 129.4 (d, J = 1.5 Hz), 127.4 (d, J = 1.3 Hz), 127.38 (d, J = 3.1 Hz), 127.1, 125.1 (d, J = 2.9 Hz), 123.4 (d, J = 8.7 Hz), 116.7 (d, J = 22.2 Hz), 115.5 (d, J = 21.0 Hz), 62.4 (d, J = 6.8 Hz), 30.0 (d, J = 139.6 Hz), 16.5 (d, J = 6.1 Hz).

^{19}F NMR (376 MHz, Chloroform-*d*) δ -112.7.

^{31}P NMR (162 MHz, Chloroform-*d*) δ 25.6.

HRMS (ESI) m/z: $[M + H]^+$ Calcd for $C_{20}H_{22}FNO_3P^+$ 374.1316; Found: 374.1320.

diethyl ((2-(3-(trifluoromethyl)phenyl)quinolin-3-yl)methyl)phosphonate (3l)

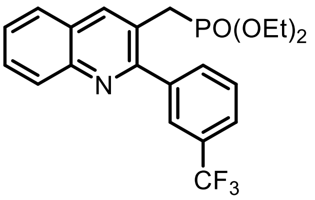

Following procedure A: T_1 = 80 ºC, 28.4 mg, yellow oil, 67% yield.

^{1}H NMR (400 MHz, Chloroform-*d*) δ 8.41 (d, *J* = 3.5 Hz, 1H), 8.11 (d, *J* = 8.5 Hz, 1H), 7.99 (s, 1H), 7.89 – 7.83 (m, 2H), 7.76 – 7.70 (m, 2H), 7.63 (t, *J* = 7.7 Hz, 1H), 7.57 (ddd, *J* = 8.1, 6.8, 1.2 Hz, 1H), 4.09 – 3.94 (m, 4H), 3.30 (d, *J* = 22.2 Hz, 2H), 1.22 (t, *J* = 7.1 Hz, 6H).

^{13}C NMR (101 MHz, Chloroform-*d*) δ 158.7 (d, *J* = 7.1 Hz), 146.9 (d, *J* = 2.6 Hz), 141.0, 138.5 (d, *J* = 6.1 Hz), 132.8, 131.0 (q, *J* = 32.5 Hz), 130.1, 129.4, 129.1, 127.5, 127.4, 127.3, 126.5 (q, *J* = 3.9 Hz), 125.3 (d, *J* = 3.8 Hz), 124.2 (q, *J* = 273.4 Hz), 123.4 (d, *J* = 8.7 Hz), 62.4 (d, *J* = 6.8 Hz), 30.1 (d, *J* = 139.6 Hz), 16.4 (d, *J* = 6.0 Hz).

^{19}F NMR (376 MHz, Chloroform-*d*) δ -62.5.

^{31}P NMR (162 MHz, Chloroform-*d*) δ 25.5.

HRMS (ESI) m/z: $[M + H]^+$ Calcd for $C_{21}H_{22}F_3NO_3P^+$ 424.1284; Found: 424.1289.

diethyl ((2-(3-methoxyphenyl)quinolin-3-yl)methyl)phosphonate (3m)

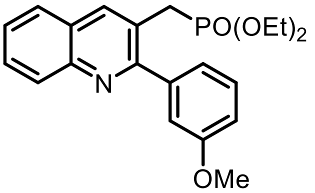

Following procedure A: T_1 = 80 ºC, 28.7 mg, yellow oil, 74% yield.

^{1}H NMR (400 MHz, Chloroform-*d*) δ 8.40 (d, *J* = 3.5 Hz, 1H), 8.12 (d, *J* = 8.5 Hz, 1H), 7.84 (d, *J* = 8.1 Hz, 1H), 7.70 (ddd, *J* = 8.3, 7.0, 1.3 Hz, 1H), 7.54 (ddd, *J* = 8.0, 6.9, 1.2 Hz, 1H), 7.40 (t, *J* = 7.8 Hz, 1H), 7.22 – 7.16 (m, 2H), 6.99 (ddd, *J* = 8.3, 2.6, 1.1 Hz, 1H), 4.06 – 3.93 (m, 4H), 3.86 (s, 3H), 3.37 (d, *J* = 22.2 Hz, 2H), 1.22 (t, *J* = 7.1 Hz, 6H).

^{13}C NMR (101 MHz, Chloroform-*d*) δ 160.2 (d, *J* = 7.4 Hz), 159.7, 146.8 (d, *J* = 2.5 Hz), 141.5, 138.1 (d, *J* = 6.0 Hz), 129.8 (d, *J* = 1.5 Hz), 129.7, 129.4 (d, *J* = 1.7 Hz), 127.4, 127.3 (d, *J* = 3.2 Hz), 126.9, 123.5 (d, *J* = 8.5 Hz), 121.7, 114.7, 114.61, 62.3 (d,

J = 6.8 Hz), 55.5, 29.9 (d, *J* = 139.3 Hz), 16.5 (d, *J* = 6.0 Hz).

^{31}P NMR (162 MHz, Chloroform-*d*) δ 26.0.

HRMS (ESI) m/z: $[M + H]^+$ Calcd for $C_{21}H_{25}NO_4P^+$ 386.1516; Found: 386.1525.

diethyl ((2-(o-tolyl)quinolin-3-yl)methyl)phosphonate (3n)

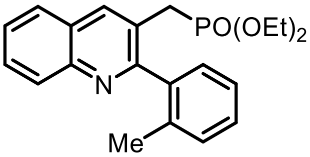

Following procedure A: T_1 = 120 ºC, 14.2 mg, yellow oil, 38% yield.

^{1}H NMR (400 MHz, Chloroform-*d*) δ 8.44 (d, *J* = 3.3 Hz, 1H), 8.11 (d, *J* = 8.5 Hz, 1H), 7.86 (d, *J* = 8.1 Hz, 1H), 7.70 (t, *J* = 7.7 Hz, 1H), 7.56 (t, *J* = 7.5 Hz, 1H), 7.37 – 7.28 (m, 4H), 4.00 – 3.90 (m, 4H), 3.31 – 3.01 (m, 2H), 2.09 (s, 3H), 1.26 – 1.18 (m, 6H).

^{13}C NMR (101 MHz, Chloroform-*d*) δ 160.8 (d, *J* = 8.0 Hz), 146.9 (d, *J* = 2.3 Hz), 139.5, 137.5 (d, *J* = 5.9 Hz), 136.0, 130.6, 129.7 (d, *J* = 1.4 Hz), 129.3 (d, *J* = 1.3 Hz), 129.2, 128.6, 127.5 (d, *J* = 1.3 Hz), 127.4 (d, *J* = 3.0 Hz), 126.8, 126.0, 124.3 (d, *J* = 8.4 Hz), 62.2, 29.4 (d, *J* = 139.9 Hz), 19.7, 16.5 (d, *J* = 6.1 Hz).

^{31}P NMR (162 MHz, Chloroform-*d*) δ 25.9.

HRMS (ESI) m/z: $[M + H]^+$ Calcd for $C_{21}H_{25}NO_3P^+$ 370.1567; Found: 370.1566.

diethyl ((2-(naphthalen-1-yl)quinolin-3-yl)methyl)phosphonate (3o)

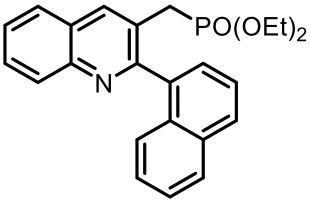

Following procedure A: T_1 = 120 ºC, 14.8 mg, yellow oil, 36% yield.

^{1}H NMR (400 MHz, Chloroform-*d*) δ 8.52 (d, *J* = 3.3 Hz, 1H), 8.14 (d, *J* = 8.4 Hz, 1H), 7.97 – 7.92 (m, 3H), 7.74 (t, *J* = 7.6 Hz, 1H), 7.62 – 7.58 (m, 3H), 7.50 – 7.46 (m, 1H), 7.41 – 7.33 (m, 2H), 3.95 – 3.74 (m, 4H), 3.19 (dd, *J* = 22.6, 15.6 Hz, 1H), 3.02 (dd, *J* = 21.5, 15.6 Hz, 1H), 1.18 – 1.11 (m, 6H).

^{13}C NMR (101 MHz, Chloroform-*d*) δ 159.7 (d, *J* = 8.0 Hz), 147.0 (d, *J* = 2.4 Hz), 137.7 (d, *J* = 5.9 Hz), 137.3, 133.8, 131.8, 129.8, 129.5, 129.0, 128.5, 127.6, 127.5 (d,

J = 2.9 Hz), 127.4, 127.1, 126.7, 126.2, 125.5, 125.2 (d, *J* = 8.3 Hz), 62.2 (d, *J* = 6.8 Hz), 62.15 (d, *J* = 6.8 Hz), 29.8 (d, *J* = 139.6 Hz), 16.4 (d, *J* = 6.1 Hz), 16.39 (d, *J* = 6.1 Hz).

^{31}P NMR (162 MHz, Chloroform-*d*) δ 25.8.

HRMS (ESI) m/z: $[M + H]^+$ Calcd for $C_{24}H_{25}NO_3P^+$ 406.1567; Found: 406.1568.

diethyl ((2-(naphthalen-2-yl)quinolin-3-yl)methyl)phosphonate (3p)

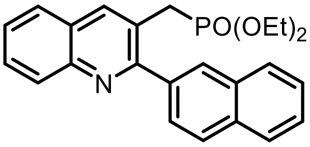

Following procedure A: T_1 = 80 ºC, 27.9 mg, yellow oil, 68% yield.

^{1}H NMR (400 MHz, Chloroform-*d*) δ 8.45 (d, *J* = 3.4 Hz, 1H), 8.16 – 8.14 (m, 2H), 7.98 (d, *J* = 8.4 Hz, 1H), 7.95 – 7.87 (m, 3H), 7.80 – 7.68 (m, 2H), 7.61 – 7.49 (m, 3H), 4.06 – 3.88 (m, 4H), 3.42 (d, *J* = 22.2 Hz, 2H), 1.20 (t, *J* = 7.1 Hz, 6H).

^{13}C NMR (101 MHz, Chloroform-*d*) δ 160.3 (d, *J* = 7.3 Hz), 147.0 (d, *J* = 2.5 Hz), 138.2 (d, *J* = 6.1 Hz), 137.7, 133.2, 133.18, 129.8 (d, *J* = 1.5 Hz), 129.4 (d, *J* = 1.5 Hz), 128.8, 128.5, 128.3, 127.8, 127.4 (d, *J* = 1.3 Hz), 127.3 (d, *J* = 3.2 Hz), 127.0, 126.9, 126.6, 126.5, 123.7 (d, *J* = 8.5 Hz), 62.3 (d, *J* = 6.7 Hz), 30.1 (d, *J* = 139.3 Hz), 16.4 (d, *J* = 6.1 Hz).

^{31}P NMR (162 MHz, Chloroform-*d*) δ 25.9.

HRMS (ESI) m/z: $[M + H]^+$ Calcd for $C_{24}H_{25}NO_3P^+$ 406.1567; Found: 406.1564.

diethyl ((2-(thiophen-3-yl)quinolin-3-yl)methyl)phosphonate (3q)

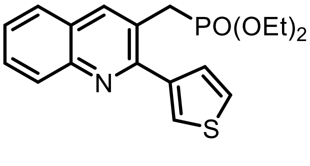

Following procedure A: T_1 = 80 ºC, 24.5 mg, yellow oil, 67% yield.

^{1}H NMR (400 MHz, Chloroform-*d*) δ 8.34 (d, *J* = 3.5 Hz, 1H), 8.09 (d, *J* = 8.5 Hz, 1H), 7.86 – 7.78 (m, 2H), 7.73 – 7.65 (m, 1H), 7.56 – 7.50 (m, 2H), 7.44 (dd, *J* = 5.0, 3.0 Hz, 1H), 4.11 – 3.95 (m, 4H), 3.45 (d, *J* = 22.2 Hz, 2H), 1.23 (t, *J* = 7.0 Hz, 6H).

^{13}C NMR (101 MHz, Chloroform-*d*) δ 155.6 (d, *J* = 7.1 Hz), 147.0 (d, *J* = 2.6 Hz),

141.1, 138.3 (d, J = 6.1 Hz), 129.7 (d, J = 1.6 Hz), 129.32, 129.3, 127.3 (d, J = 1.4 Hz), 127.1 (d, J = 3.3 Hz), 126.8, 125.9, 125.83, 123.6 (d, J = 8.7 Hz), 62.4 (d, J = 6.8 Hz), 30.2 (d, J = 139.4 Hz), 16.5 (d, J = 6.0 Hz).

^{31}P NMR (162 MHz, Chloroform-*d*) δ 26.0.

HRMS (ESI) m/z: $[M + H]^+$ Calcd for $C_{18}H_{21}NO_3PS^+$ 362.0974; Found: 362.0978.

diethyl ((2-(furan-2-yl)quinolin-3-yl)methyl)phosphonate (3r)

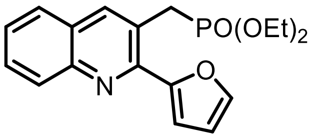

Following procedure A: T_1 = 80 ºC, 13.1 mg, yellow oil, 37% yield.

^{1}H NMR (400 MHz, Chloroform-*d*) δ 8.22 (d, J = 3.6 Hz, 1H), 8.08 (d, J = 8.5 Hz, 1H), 7.77 (d, J = 8.1 Hz, 1H), 7.71 – 7.62 (m, 2H), 7.50 (t, J = 7.5 Hz, 1H), 7.25 (s, 1H), 6.60 (dd, J = 3.5, 1.8 Hz, 1H), 4.05 – 3.91 (m, 4H), 3.80 (d, J = 22.2 Hz, 2H), 1.17 (t, J = 7.1 Hz, 6H).

^{13}C NMR (101 MHz, Chloroform-*d*) δ 154.0, 148.6 (d, J = 5.9 Hz), 147.0 (d, J = 2.5 Hz), 143.6, 139.4 (d, J = 6.6 Hz), 129.9 (d, J = 1.6 Hz), 129.3 (d, J = 1.5 Hz), 127.3 (d, J = 1.5 Hz), 127.0 (d, J = 3.2 Hz), 126.9, 122.8 (d, J = 9.8 Hz), 112.6, 112.0, 62.3 (d, J = 6.9 Hz), 30.7 (d, J = 139.0 Hz), 16.4 (d, J = 6.1 Hz).

^{31}P NMR (162 MHz, Chloroform-*d*) δ 25.7.

HRMS (ESI) m/z: $[M + H]^+$ Calcd for $C_{18}H_{21}NO_4P^+$ 346.1203; Found: 346.1205.

diethyl ((5-fluoro-2-phenylquinolin-3-yl)methyl)phosphonate (3s)

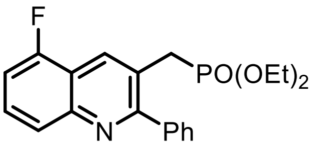

Following procedure A: T_1 = 80 ºC, 23.9 mg, colorless oil, 64% yield.

^{1}H NMR (400 MHz, Chloroform-*d*) δ 8.61 (d, J = 3.5 Hz, 1H), 7.92 (d, J = 8.5 Hz, 1H), 7.67 – 7.58 (m, 3H), 7.53 – 7.43 (m, 3H), 7.20 – 7.17 (m, 1H), 4.06 – 3.94 (m, 4H), 3.40 (d, J = 22.3 Hz, 2H), 1.24 (t, J = 7.1 Hz, 6H).

^{13}**C NMR** (101 MHz, Chloroform-*d*) δ 161.3 (d, *J* = 6.9 Hz), 157.6 (d, *J* = 255.8 Hz), 147.6 (t, *J* = 2.7 Hz), 139.9, 131.4 (dd, *J* = 6.1, 4.1 Hz), 129.3, 129.1 (d, *J* = 9.0 Hz), 128.7, 128.6, 125.3 (dd, *J* = 4.2, 1.5 Hz), 124.2 (dd, *J* = 9.0, 2.5 Hz), 118.1 (dd, *J* = 16.0, 3.4 Hz), 110.3 (d, *J* = 19.2 Hz), 62.3 (d, *J* = 6.8 Hz), 30.2 (d, *J* = 139.2 Hz), 16.4 (d, *J* = 6.2 Hz).

^{19}**F NMR** (376 MHz, Chloroform-*d*) δ -122.9.

^{31}**P NMR** (162 MHz, Chloroform-*d*) δ 25.3.

HRMS (ESI) m/z: $[M + H]^+$ Calcd for $C_{20}H_{22}FNO_3P^+$ 374.1316; Found: 374.1315.

diethyl ((5-chloro-2-phenylquinolin-3-yl)methyl)phosphonate (3t)

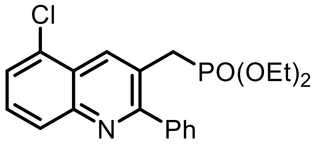

Following procedure A: T_1 = 80 ºC, 25 mg, colorless oil, 64% yield.

^{1}**H NMR** (400 MHz, Chloroform-*d*) δ 8.75 (d, *J* = 3.5 Hz, 1H), 8.06 – 8.02 (m, 1H), 7.66 (dt, *J* = 6.1, 1.5 Hz, 2H), 7.65 – 7.56 (m, 2H), 7.55 – 7.41 (m, 3H), 4.05 – 3.98 (m, 4H), 3.42 (d, *J* = 22.3 Hz, 2H), 1.26 (t, *J* = 7.1 Hz, 6H).

^{13}**C NMR** (101 MHz, Chloroform-*d*) δ 161.2 (d, *J* = 7.0 Hz), 147.5 (d, *J* = 2.6 Hz), 139.7, 135.0 (d, *J* = 6.0 Hz), 130.9 (d, *J* = 1.7 Hz), 129.4, 129.3, 128.8, 128.7, 128.65, 126.8, 125.4 (d, *J* = 3.3 Hz), 125.0 (d, *J* = 9.1 Hz), 62.4 (d, *J* = 6.7 Hz), 30.3 (d, *J* = 139.1 Hz), 16.5 (d, *J* = 6.2 Hz).

^{31}**P NMR** (162 MHz, Chloroform-*d*) δ 25.2.

HRMS (ESI) m/z: $[M + H]^+$ Calcd for $C_{20}H_{22}ClNO_3P^+$ 390.1020; Found: 390.1025.

diethyl ((5-nitro-2-phenylquinolin-3-yl)methyl)phosphonate (3u)

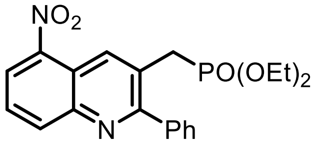

Following procedure A: T_1 = 80 ºC, 22.7 mg, yellow solid, 56% yield. M.p = 76 – 77 ºC.

^{1}H NMR (400 MHz, Chloroform-*d*) δ 9.18 (d, *J* = 3.5 Hz, 1H), 8.41 (dd, *J* = 15.2, 8.1 Hz, 2H), 7.77 (t, *J* = 8.1 Hz, 1H), 7.69 (d, *J* = 7.4 Hz, 2H), 7.55 – 7.47 (m, 3H), 4.08 – 4.01 (m, 4H), 3.45 (d, *J* = 22.4 Hz, 2H), 1.27 (t, *J* = 7.1 Hz, 6H).

^{13}C NMR (101 MHz, Chloroform-*d*) δ 161.8 (d, *J* = 6.8 Hz), 147.0 (d, *J* = 2.5 Hz), 145.0, 139.2, 136.7 (d, *J* = 1.6 Hz), 134.0 (d, *J* = 6.1 Hz), 129.3, 129.1, 128.8, 127.8 (d, *J* = 9.2 Hz), 127.6 (d, *J* = 1.5 Hz), 124.9, 120.2 (d, *J* = 3.5 Hz), 62.5 (d, *J* = 6.7 Hz), 30.7 (d, *J* = 138.5 Hz), 16.4 (d, *J* = 6.2 Hz).

^{31}P NMR (162 MHz, Chloroform-*d*) δ 24.6.

HRMS (ESI) m/z: $[M + H]^+$ Calcd for $C_{20}H_{22}N_2O_5P^+$ 401.1261; Found: 401.1264.

diethyl ((5-methyl-2-phenylquinolin-3-yl)methyl)phosphonate (3v)

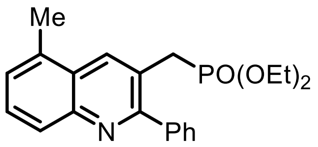

Following procedure A: T_1 = 80 ºC, 27.3 mg, yellow oil, 74% yield.

^{1}H NMR (400 MHz, Chloroform-*d*) δ 8.56 (d, *J* = 3.4 Hz, 1H), 7.97 (d, *J* = 8.5 Hz, 1H), 7.63 (d, *J* = 8.0 Hz, 2H), 7.58 (t, *J* = 7.7 Hz, 1H), 7.49 (dd, *J* = 8.0, 6.6 Hz, 2H), 7.48 – 7.39 (m, 1H), 7.36 (d, *J* = 7.0 Hz, 1H), 4.04 – 3.89 (m, 4H), 3.40 (d, *J* = 22.2 Hz, 2H), 2.73 (s, 3H), 1.22 (t, *J* = 7.0 Hz, 6H).

^{13}C NMR (101 MHz, Chloroform-*d*) δ 159.8 (d, *J* = 7.3 Hz), 147.2 (d, *J* = 2.6 Hz), 140.3, 134.8 (d, *J* = 6.0 Hz), 134.3 (d, *J* = 1.4 Hz), 129.4 (d, *J* = 1.5 Hz), 129.36, 128.6, 128.4, 127.7 (d, *J* = 1.5 Hz), 127.2, 126.6 (d, *J* = 3.2 Hz), 123.0 (d, *J* = 8.8 Hz), 62.2 (d, *J* = 6.8 Hz), 30.2 (d, *J* = 139.1 Hz), 18.8, 16.5 (d, *J* = 6.1 Hz).

^{31}P NMR (162 MHz, Chloroform-*d*) δ 25.9.

HRMS (ESI) m/z: $[M + H]^+$ Calcd for $C_{21}H_{25}NO_3P^+$ 370.1567; Found: 370.1571.

diethyl ((5-methoxy-2-phenylquinolin-3-yl)methyl)phosphonate (3w)

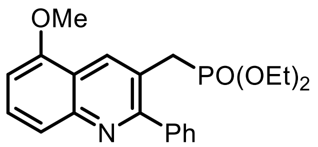

Following procedure A: T_1 = 80 ºC, 24.5 mg, yellow oil, 63% yield.

^{1}H NMR (400 MHz, Chloroform-*d*) δ 8.77 (d, *J* = 3.5 Hz, 1H), 7.70 (d, *J* = 8.5 Hz, 1H), 7.66 – 7.60 (m, 2H), 7.63 – 7.54 (m, 1H), 7.53 – 7.44 (m, 2H), 7.47 – 7.39 (m, 1H), 6.84 (d, *J* = 7.7 Hz, 1H), 4.01 (s, 3H), 4.01 – 3.91 (m, 4H), 3.38 (d, *J* = 22.1 Hz, 2H), 1.22 (t, *J* = 7.1 Hz, 6H).

^{13}C NMR (101 MHz, Chloroform-*d*) δ 160.5 (d, *J* = 7.2 Hz), 155.0, 147.7 (d, *J* = 2.6 Hz), 140.3, 133.1 (d, *J* = 5.8 Hz), 129.7 (d, *J* = 1.5 Hz), 129.4, 128.5, 128.4, 122.6 (d, *J* = 8.9 Hz), 121.5 (d, *J* = 1.5 Hz), 119.8 (d, *J* = 3.2 Hz), 104.3, 62.3 (d, *J* = 6.8 Hz), 55.9, 30.1 (d, *J* = 139.1 Hz), 16.4 (d, *J* = 6.3 Hz).

^{31}P NMR (162 MHz, Chloroform-*d*) δ 25.9.

HRMS (ESI) m/z: $[M + H]^+$ Calcd for $C_{21}H_{25}NO_4P^+$ 386.1516; Found: 386.1515.

***tert*-butyl (3-((diethoxyphosphoryl)methyl)-2-phenylquinolin-5-yl)carbamate (3x)**

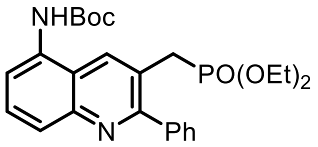

Following procedure A: T_1 = 80 ºC, 27.6 mg, white solid, 58% yield. M.p = 186 – 187 ºC.

^{1}H NMR (400 MHz, Chloroform-*d*) δ 8.48 (d, *J* = 3.4 Hz, 1H), 7.93 (d, *J* = 7.5 Hz, 1H), 7.89 (d, *J* = 8.4 Hz, 1H), 7.66 (t, *J* = 8.1 Hz, 1H), 7.61 (d, *J* = 7.2 Hz, 2H), 7.53 – 7.37 (m, 4H), 4.03 – 3.89 (m, 4H), 3.39 (d, *J* = 22.3 Hz, 2H), 1.56 (s, 9H), 1.21 (t, *J* = 7.1 Hz, 6H).

^{13}C NMR (101 MHz, Chloroform-*d*) δ 160.1 (d, *J* = 7.3 Hz), 153.6, 147.3 (d, *J* = 2.5 Hz), 140.0, 133.0, 131.7 (d, *J* = 5.9 Hz), 129.8, 129.3, 128.6, 125.6, 123.0 (d, *J* = 8.8 Hz), 120.8, 119.2, 81.0, 62.4 (d, *J* = 6.8 Hz), 30.0 (d, *J* = 139.1 Hz), 28.5, 16.4 (d, *J* = 6.2 Hz).

^{31}P NMR (162 MHz, Chloroform-*d*) δ 25.9.

HRMS (ESI) m/z: $[M + H]^+$ Calcd for $C_{25}H_{32}N_2O_5P^+$ 471.2043; Found: 471.2051.

diethyl ((6-chloro-2-phenylquinolin-3-yl)methyl)phosphonate (3y)

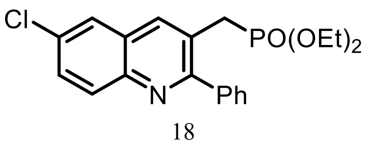

Following procedure A: T_1 = 80 ºC, 29 mg, yellow oil, 74% yield.

^{1}H NMR (400 MHz, Chloroform-*d*) δ 8.31 (d, *J* = 3.4 Hz, 1H), 8.04 (d, *J* = 9.0 Hz, 1H), 7.82 (d, *J* = 2.3 Hz, 1H), 7.66 – 7.57 (m, 3H), 7.54 – 7.40 (m, 3H), 4.01 – 3.91 (m, 4H), 3.36 (d, *J* = 22.3 Hz, 2H)., 1.21 (t, *J* = 7.1 Hz, 6H).

^{13}C NMR (101 MHz, Chloroform-*d*) δ 160.6 (d, *J* = 7.3 Hz), 145.2 (d, *J* = 2.3 Hz), 139.9, 137.1 (d, *J* = 6.1 Hz), 132.5 (d, *J* = 1.4 Hz), 131.1 (d, *J* = 1.5 Hz), 130.7 (d, *J* = 1.5 Hz), 129.3, 128.7, 128.6, 127.8 (d, *J* = 3.1 Hz), 126.0 (d, *J* = 1.4 Hz), 124.8 (d, *J* = 8.7 Hz), 62.4 (d, *J* = 6.8 Hz), 30.0 (d, *J* = 139.1 Hz), 16.5 (d, *J* = 6.1 Hz).

^{31}P NMR (162 MHz, Chloroform-*d*) δ 25.5.

HRMS (ESI) m/z: $[M + H]^+$ Calcd for $C_{20}H_{22}ClNO_3P^+$ 390.1020; Found: 390.1019.

diethyl ((6-methoxy-2-phenylquinolin-3-yl)methyl)phosphonate (3z)

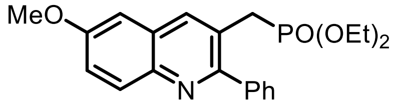

Following procedure A: T_1 = 80 ºC, 24.1 mg, white solid, 62% yield. M.p = 72 – 73 ºC.

^{1}H NMR (400 MHz, Chloroform-*d*) δ 8.31 (d, *J* = 3.4 Hz, 1H), 8.00 (d, *J* = 9.2 Hz, 1H), 7.60 (d, *J* = 7.4 Hz, 2H), 7.48 (t, *J* = 7.4 Hz, 2H), 7.46 – 7.38 (m, 1H), 7.34 (dd, *J* = 9.3, 2.8 Hz, 1H), 7.09 (d, *J* = 2.8 Hz, 1H), 4.01 – 3.93 (m, 4H), 3.93 (s, 3H), δ 3.34 (d, *J* = 22.2 Hz, 2H), 1.20 (t, *J* = 7.1 Hz, 6H).

^{13}C NMR (101 MHz, Chloroform-*d*) δ 158.1, 157.8 (d, *J* = 7.5 Hz), 143.1 (d, *J* = 2.5 Hz), 140.3, 136.8 (d, *J* = 5.9 Hz), 130.8 (d, *J* = 1.5 Hz), 129.4, 128.5, 128.3, 128.2, 123.7 (d, *J* = 8.5 Hz), 122.6 (d, *J* = 1.5 Hz), 104.6, 62.3 (d, *J* = 6.8 Hz), 55.7, 29.9 (d, *J* = 139.0 Hz), 16.5 (d, *J* = 5.9 Hz).

^{31}P NMR (162 MHz, Chloroform-*d*) δ 26.1.

HRMS (ESI) m/z: $[M + H]^+$ Calcd for $C_{21}H_{25}NO_4P^+$ 386.1516; Found: 386.1518.

diethyl ((7-chloro-2-phenylquinolin-3-yl)methyl)phosphonate (3aa)

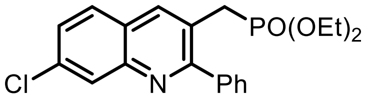

Following procedure A: T_1 = 80 ºC, 25.8 mg, yellow oil, 66% yield.

^{1}H NMR (400 MHz, Chloroform-*d*) δ 8.38 (d, *J* = 3.4 Hz, 1H), 8.11 (d, *J* = 2.1 Hz, 1H), 7.77 (d, *J* = 8.7 Hz, 1H), 7.64 – 7.58 (m, 2H), 7.54 – 7.41 (m, 4H), 4.02 – 3.93 (m, 4H), 3.35 (d, *J* = 22.2 Hz, 2H), 1.20 (t, *J* = 7.1 Hz, 6H).

^{13}C NMR (101 MHz, Chloroform-*d*) δ 161.3 (d, *J* = 7.3 Hz), 147.2 (d, *J* = 2.5 Hz), 139.9, 137.9 (d, *J* = 6.1 Hz), 135.5 (d, *J* = 1.8 Hz), 129.3, 128.7, 128.65, 128.6, 128.4 (d, *J* = 1.5 Hz), 127.9, 125.6 (d, *J* = 3.2 Hz), 123.9 (d, *J* = 8.6 Hz), 62.4 (d, *J* = 6.8 Hz), 29.9 (d, *J* = 139.2 Hz), 16.5 (d, *J* = 6.1 Hz).

^{31}P NMR (162 MHz, Chloroform-*d*) δ 25.6.

HRMS (ESI) m/z: $[M + H]^+$ Calcd for $C_{20}H_{22}ClNO_3P^+$ 390.1020; Found: 390.1030.

diethyl ((7-bromo-2-phenylquinolin-3-yl)methyl)phosphonate (3ab)

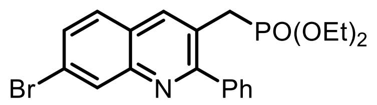

Following procedure A: T_1 = 80 ºC, 30.9 mg, yellow oil, 71% yield.

^{1}H NMR (400 MHz, Chloroform-*d*) δ 8.38 (d, *J* = 3.4 Hz, 1H), 8.31 (d, *J* = 1.9 Hz, 1H), 7.71 (d, *J* = 8.7 Hz, 1H), 7.63 – 7.59 (m, 3H), 7.54 – 7.41 (m, 3H), 4.06 – 3.88 (m, 4H), 3.35 (d, *J* = 22.3 Hz, 2H), 1.20 (t, *J* = 7.1 Hz, 6H).

^{13}C NMR (101 MHz, Chloroform-*d*) δ 161.3 (d, *J* = 7.3 Hz), 147.4 (d, *J* = 2.5 Hz), 139.8, 138.0 (d, *J* = 6.0 Hz), 131.7 (d, *J* = 1.5 Hz), 130.4, 129.3, 128.7, 128.68 (d, *J* = 1.3 Hz), 128.6, 125.8 (d, *J* = 3.2 Hz), 124.1 (d, *J* = 8.5 Hz), 123.7 (d, *J* = 1.9 Hz), 62.4 (d, *J* = 6.8 Hz), 30.0 (d, *J* = 139.2 Hz), 16.5 (d, *J* = 5.9 Hz).

^{31}P NMR (162 MHz, Chloroform-*d*) δ 25.5.

HRMS (ESI) m/z: $[M + H]^+$ Calcd for $C_{20}H_{22}BrNO_3P^+$ 434.0515; Found: 434.0521.

diethyl ((7-methyl-2-phenylquinolin-3-yl)methyl)phosphonate (3ac)

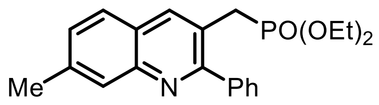

Following procedure A: T_1 = 80 ºC, 27.8 mg, yellow oil, 75% yield.

^{1}H NMR (400 MHz, Chloroform-*d*) δ 8.35 (d, *J* = 3.4 Hz, 1H), 7.90 (s, 1H), 7.73 (d, *J* = 8.3 Hz, 1H), 7.65 – 7.57 (m, 2H), 7.53 – 7.34 (m, 4H), 4.05 – 3.87 (m, 4H), 3.35 (d,

J = 22.1 Hz, 2H), 2.55 (s, 3H), 1.20 (t, J = 7.1 Hz, 6H).

^{13}C NMR (101 MHz, Chloroform-*d*) δ 160.2 (d, J = 7.3 Hz), 147.1 (d, J = 2.4 Hz), 140.4, 140.0 (d, J = 1.5 Hz), 137.8 (d, J = 5.9 Hz), 129.3, 129.1, 128.5, 128.4, 128.35, 127.0 (d, J = 1.4 Hz), 125.3 (d, J = 3.2 Hz), 122.5 (d, J = 8.7 Hz), 62.2 (d, J = 6.7 Hz), 29.9 (d, J = 139.3 Hz), 22.0, 16.5 (d, J = 6.0 Hz).

^{31}P NMR (162 MHz, Chloroform-*d*) δ 26.1.

HRMS (ESI) m/z: $[M + H]^+$ Calcd for $C_{21}H_{25}NO_3P^+$ 370.1567; Found: 370.1562.

diethyl ((6,7-dimethoxy-2-phenylquinolin-3-yl)methyl)phosphonate (3ad)

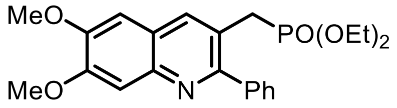

Following procedure A: T_1 = 80 ºC, 22.7 mg, yellow oil, 54% yield.

^{1}H NMR (400 MHz, Chloroform-*d*) δ 8.27 (d, J = 3.2 Hz, 1H), 7.63 – 7.55 (m, 2H), 7.52 – 7.37 (m, 4H), 7.08 (s, 1H), 4.03 (s, 3H), 4.00 (s, 3H), 4.00 – 3.90 (m, 4H), 3.32 (d, J = 22.0 Hz, 2H), 1.20 (t, J = 7.1 Hz, 6H).

^{13}C NMR (101 MHz, Chloroform-*d*) δ 158.0 (d, J = 7.8 Hz), 152.8, 150.2, 144.0 (d, J = 2.4 Hz), 140.6, 136.3 (d, J = 5.7 Hz), 129.4, 128.5, 128.2, 123.0 (d, J = 3.1 Hz), 121.4 (d, J = 8.4 Hz), 108.0, 104.7, 62.3 (d, J = 6.8 Hz), 56.3, 56.2, 29.8 (d, J = 139.3 Hz), 16.5 (d, J = 6.0 Hz).

^{31}P NMR (162 MHz, Chloroform-*d*) δ 26.4.

HRMS (ESI) m/z: $[M + H]^+$ Calcd for $C_{22}H_{27}NO_5P^+$ 416.1621; Found: 416.1627.

diethyl ((2-phenylbenzo[*g*]quinolin-3-yl)methyl)phosphonate (3ae)

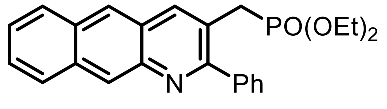

Following procedure A: T_1 = 80 ºC, 20.6 mg, yellow oil, 50% yield.

^{1}H NMR (400 MHz, Chloroform-*d*) δ 8.72 (s, 1H), 8.56 (d, J = 3.9 Hz, 1H), 8.42 (s, 1H), 8.10 – 8.00 (m, 2H), 7.72 – 7.64 (m, 2H), 7.60 – 7.34 (m, 5H), 4.05 – 3.89 (m, 4H), 3.42 (d, J = 22.4 Hz, 2H), 1.21 (t, J = 7.1 Hz, 6H).

^{13}C NMR (101 MHz, Chloroform-*d*) δ 161.4 (d, J = 7.2 Hz), 143.4 (d, J = 2.3 Hz),

140.3, 138.2 (d, J = 7.0 Hz), 134.2 (d, J = 1.7 Hz), 132.1 (d, J = 1.4 Hz), 129.3, 128.7, 128.65, 128.62, 128.3, 127.4 (d, J = 1.5 Hz), 126.3, 126.2, 126.18, 125.7 (d, J = 3.4 Hz), 123.2 (d, J = 9.1 Hz), 62.4 (d, J = 6.8 Hz), 30.1 (d, J = 139.4 Hz), 16.5 (d, J = 6.1 Hz).

^{31}P NMR (162 MHz, Chloroform-*d*) δ 25.9.

HRMS (ESI) m/z: $[M + H]^+$ Calcd for $C_{24}H_{25}NO_3P^+$ 406.1567; Found: 406.1566.

diethyl ((8-methyl-2-phenylquinolin-3-yl)methyl)phosphonate (3af)

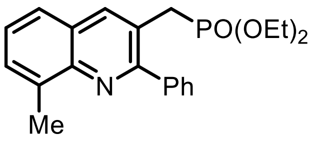

Following procedure A: T_1 = 80 ºC, 20.5 mg, yellow oil, 55% yield.

^{1}H NMR (400 MHz, Chloroform-*d*) δ 8.35 (d, J = 3.4 Hz, 1H), 7.75 – 7.64 (m, 3H), 7.58 – 7.39 (m, 5H), 4.06 – 3.95 (m, 2H), 3.99 – 3.88 (m, 2H), 3.42 (d, J = 22.2 Hz, 2H), 2.79 (s, 3H), 1.21 (t, J = 7.0 Hz, 6H).

^{13}C NMR (101 MHz, Chloroform-*d*) δ 158.7 (d, J = 7.3 Hz), 146.1 (d, J = 2.4 Hz), 140.8, 138.3 (d, J = 6.0 Hz), 137.5 (d, J = 1.5 Hz), 129.8, 129.6 (d, J = 1.5 Hz), 128.4, 128.3, 127.1 (d, J = 3.1 Hz), 126.6, 125.3 (d, J = 1.4 Hz), 123.0 (d, J = 8.6 Hz), 62.3 (d, J = 6.7 Hz), 30.0 (d, J = 139.0 Hz), 18.0, 16.5 (d, J = 6.1 Hz).

^{31}P NMR (162 MHz, Chloroform-*d*) δ 26.1.

HRMS (ESI) m/z: $[M + H]^+$ Calcd for $C_{21}H_{25}NO_3P^+$ 370.1567; Found: 370.1567.

methyl (*E*)-3-(3-((diethoxyphosphoryl)methyl)quinolin-2-yl)acrylate (3ag)

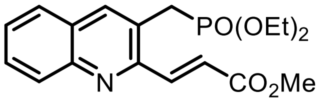

Following procedure A: T_1 = 80 ºC, 18.2 mg, white solid, 44% yield. M.p = 68 – 70 ºC.

^{1}H NMR (400 MHz, Chloroform-*d*) δ 8.19 – 8.10 (m, 2H), 8.06 (d, J = 8.5 Hz, 1H), 7.77 (d, J = 8.1 Hz, 1H), 7.69 (ddd, J = 8.3, 7.0, 1.3 Hz, 1H), 7.53 (ddd, J = 8.1, 6.9, 1.2 Hz, 1H), 7.27 (d, J = 15.1 Hz, 1H), 4.05 – 4.02 (m, 4H), 3.85 (s, 3H), 3.45 (d, J = 21.9 Hz, 2H), 1.24 (t, J = 7.1 Hz, 6H).

^{13}C NMR (101 MHz, Chloroform-*d*) δ 167.3, 151.7 (d, J = 5.3 Hz), 147.3 (d, J = 2.7

Hz), 140.3, 138.6 (d, J = 6.8 Hz), 130.0 (d, J = 1.6 Hz), 129.8 (d, J = 1.6 Hz), 128.3 (d, J = 3.2 Hz), 127.6, 127.3, 124.9 (d, J = 9.6 Hz), 124.86, 62.6 (d, J = 6.9 Hz), 52.0, 30.4 (d, J = 139.8 Hz), 16.5 (d, J = 6.1 Hz).

^{31}P NMR (162 MHz, Chloroform-*d*) δ 24.3.

HRMS (ESI) m/z: $[M + H]^+$ Calcd for $C_{18}H_{23}NO_5P^+$ 364.1308, Found: 364.1308.

diethyl (*E*)-((2-styrylquinolin-3-yl)methyl)phosphonate (3ah)

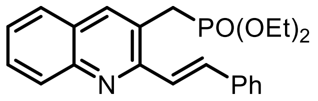

Following procedure A: T_1 = 80 ºC, 12.5 mg, yellow oil, 32% yield.

^{1}H NMR (400 MHz, Chloroform-*d*) δ 8.12 – 8.06 (m, 2H), 8.00 (d, J = 15.5 Hz, 1H), 7.75 (d, J = 8.1 Hz, 1H), 7.70 – 7.61 (m, 4H), 7.48 (t, J = 7.5 Hz, 1H), 7.40 (dd, J = 8.3, 6.7 Hz, 2H), 7.33 (dd, J = 8.3, 6.2 Hz, 1H), 4.09 – 4.02 (m, 4H), 3.49 (d, J = 21.8 Hz, 2H), 1.23 (t, J = 7.1 Hz, 6H).

^{13}C NMR (101 MHz, Chloroform-*d*) δ 154.5 (d, J = 5.0 Hz), 147.4 (d, J = 2.6 Hz), 138.3 (d, J = 7.0 Hz), 137.1, 136.2, 129.7 (d, J = 1.7 Hz), 129.2 (d, J = 1.6 Hz), 128.9, 128.7, 127.6, 127.5 (d, J = 3.4 Hz), 127.3, 126.4, 124.5, 123.9 (d, J = 9.7 Hz), 62.6 (d, J = 6.9 Hz), 30.8 (d, J = 139.6 Hz), 16.5 (d, J = 6.1 Hz).

^{31}P NMR (162 MHz, Chloroform-*d*) δ 25.2.

HRMS (ESI) m/z: $[M + H]^+$ Calcd for $C_{22}H_{25}NO_3P^+$ 382.1567, Found: 382.1570.

diethyl ((2-cyclopropylquinolin-3-yl)methyl)phosphonate (3ai)

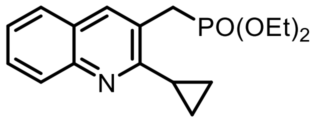

Following procedure A: T_1 = 80 ºC, MeOH (0.5 mL) and CH_3COOH (6.0 mg, 0.1 mmol) were added, 7.9 mg, yellow oil, 24% yield.

^{1}H NMR (400 MHz, Chloroform-*d*) δ 8.02 (d, J = 3.7 Hz, 1H), 7.92 (d, J = 8.5 Hz, 1H), 7.72 (d, J = 8.1 Hz, 1H), 7.60 (dd, J = 8.4, 6.8 Hz, 1H), 7.42 (t, J = 7.3 Hz, 1H), 4.15 – 3.97 (m, 4H), 3.52 (d, J = 21.9 Hz, 2H), 2.46 – 2.39 (m, 1H), 1.25 (t, J = 7.0 Hz, 8H), 1.08 – 1.03 (m, 2H).

^{13}C NMR (101 MHz, Chloroform-*d*) δ 161.4 (d, *J* = 5.3 Hz), 147.0, 137.0 (d, *J* = 6.9 Hz), 129.1, 128.7, 127.2 (d, *J* = 1.5 Hz), 126.7 (d, *J* = 3.3 Hz), 125.7, 124.6 (d, *J* = 9.7 Hz), 62.5 (d, *J* = 6.9 Hz), 30.3 (d, *J* = 139.9 Hz), 16.6 (d, *J* = 6.1 Hz), 15.0, 9.5.

^{31}P NMR (162 MHz, Chloroform-*d*) δ 25.8.

HRMS (ESI) m/z: $[M + H]^+$ Calcd for $C_{17}H_{23}NO_3P^+$ 320.1410, Found: 320.1421.

diethyl ((2,6-diphenyl-1,5-naphthyridin-3-yl)methyl)phosphonate (3aj)

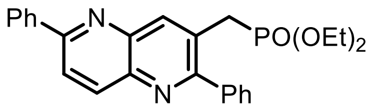

Following procedure A: T_1 = 80 ºC, 28.5 mg, yellow solid, 65% yield. M.p = 124 – 126 ºC.

^{1}H NMR (400 MHz, Chloroform-*d*) δ 8.63 (d, *J* = 3.4 Hz, 1H), 8.46 (d, *J* = 8.9, 1H), 8.21 – 8.17 (m, 2H), 8.10 (d, *J* = 8.8 Hz, 1H), 7.70 – 7.66 (m, 2H), 7.57 – 7.45 (m, 6H), 4.09 – 3.95 (m, 4H), 3.44 (d, *J* = 22.4 Hz, 2H), 1.25 (t, *J* = 7.1 Hz, 6H).

^{13}C NMR (101 MHz, Chloroform-*d*) δ 160.8 (d, *J* = 6.9 Hz), 158.3, 142.7 (d, *J* = 3.3 Hz), 141.6 (d, *J* = 2.5 Hz), 139.8, 139.3 (d, *J* = 5.9 Hz), 139.1, 137.8, 129.9, 129.4, 129.1, 128.8, 128.7, 127.9 (d, *J* = 9.0 Hz), 127.8, 122.6, 62.4 (d, *J* = 6.6 Hz), 30.5 (d, *J* = 138.7 Hz), 16.5 (d, *J* = 5.9 Hz).

^{31}P NMR (162 MHz, Chloroform-*d*) δ 25.2.

HRMS (ESI) m/z: $[M + H]^+$ Calcd for $C_{25}H_{26}N_2O_3P^+$ 433.1676, Found: 433.1675.

diethyl ((2,7-diphenyl-1,8-naphthyridin-3-yl)methyl)phosphonate (3ak)

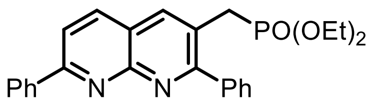

Following procedure A: T_1 = 80 ºC, 18.6 mg, yellow solid, 43% yield. M.p = 92 – 94 ºC.

^{1}H NMR (400 MHz, Chloroform-*d*) δ 8.48 (d, *J* = 3.4 Hz, 1H), 8.35 – 8.25 (m, 3H), 8.02 (d, *J* = 8.5 Hz, 1H), 7.73 – 7.66 (m, 2H), 7.71 – 7.43 (m, 6H), 4.04 – 3.88 (m, 4H), 3.43 (d, *J* = 22.3 Hz, 2H), 1.21 (t, *J* = 7.0 Hz, 6H).

^{13}C NMR (101 MHz, Chloroform-*d*) δ 163.9 (d, *J* = 7.5 Hz), 160.8 (d, *J* = 1.4 Hz),

154.8 (d, J = 2.6 Hz), 139.7, 138.8 (d, J = 5.9 Hz), 138.7, 137.4, 130.2, 129.5, 128.9, 128.7, 128.4, 128.1, 124.6 (d, J = 8.5 Hz), 120.6 (d, J = 3.2 Hz), 120.1, 62.4 (d, J = 6.9 Hz), 29.9 (d, J = 139.1 Hz), 16.5 (d, J = 6.1 Hz).

^{31}P NMR (162 MHz, Chloroform-*d*) δ 25.6.

HRMS (ESI) m/z: $[M + H]^+$ Calcd for $C_{25}H_{26}N_2O_3P^+$ 433.1676, Found: 433.1682.

diethyl ((2-(benzo[*d*][1,3]dioxol-5-yl)quinolin-3-yl)methyl)phosphonate (3al)

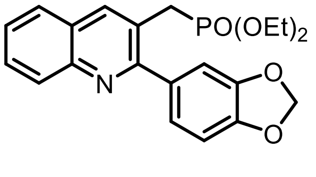

Following procedure A: T_1 = 80 ºC, 29.2 mg, yellow oil, 73% yield.

^{1}H NMR (400 MHz, Chloroform-*d*) δ 8.38 (d, J = 3.5 Hz, 1H), 8.09 (d, J = 8.5 Hz, 1H), 7.82 (d, J = 8.1 Hz, 1H), 7.69 (t, J = 7.7 Hz, 1H), 7.53 (t, J = 7.5 Hz, 1H), 7.12 (d, J = 9.5 Hz, 2H), 6.92 (d, J = 7.8 Hz, 1H), 6.02 (s, 2H), 4.04 – 3.97 (m, 4H), 3.39 (d, J = 22.2 Hz, 2H), 1.23 (t, J = 7.0 Hz, 6H).

^{13}C NMR (101 MHz, Chloroform-*d*) δ 159.8 (d, J = 7.4 Hz), 147.9, 147.8, 146.8 (d, J = 2.5 Hz), 138.1 (d, J = 5.9 Hz), 134.2, 129.7, 129.3, 127.4, 127.2 (d, J = 3.2 Hz), 126.8, 123.5 (d, J = 8.4 Hz), 123.3, 110.1, 108.4, 101.3, 62.3 (d, J = 6.8 Hz), 30.0 (d, J = 139.3 Hz), 16.5 (d, J = 6.1 Hz).

^{31}P NMR (162 MHz, Chloroform-*d*) δ 26.0.

HRMS (ESI) m/z: $[M + H]^+$ Calcd for $C_{21}H_{23}NO_5P^+$ 400.1308; Found: 400.1308.

heptan-2-yl 2-((5-chloro-3-((diethoxyphosphoryl)methyl)-2-phenylquinolin-8-yl)oxy)acetate (3am)

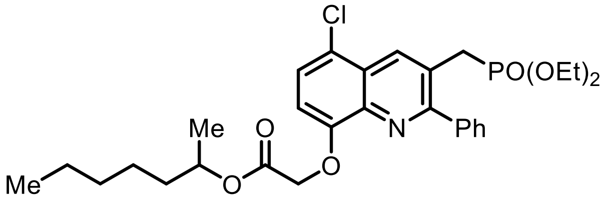

Following procedure A: T_1 = 120 ºC, 19.2 mg, yellow oil, 34% yield.

^{1}H NMR (400 MHz, Chloroform-*d*) δ 8.70 (d, J = 3.5 Hz, 1H), 7.73 – 7.65 (m, 2H), 7.52 – 7.41 (m, 4H), 6.90 (d, J = 8.4 Hz, 1H), 5.02 – 4.96 (m, 1H), 4.93 (s, 2H), 4.07 –

3.96 (m, 4H), 3.44 (d, *J* = 22.3 Hz, 2H), 1.61 – 1.40 (m, 2H), 1.27 – 1.18 (m, 15H), 0.87 – 0.83 (m, 3H).

^{13}C NMR (101 MHz, Chloroform-*d*) δ 168.6, 159.9 (d, *J* = 7.0 Hz), 153.2, 139.8, 139.5 (d, *J* = 2.8 Hz), 135.3 (d, *J* = 6.0 Hz), 129.8, 128.7, 128.5, 126.3, 126.2, 125.6 (d, *J* = 9.1 Hz), 123.3, 110.8, 72.7, 67.0, 62.4 (d, *J* = 6.8 Hz), 35.8, 31.7, 30.3 (d, *J* = 138.8 Hz), 25.1, 22.6, 20.0, 16.5 (d, *J* = 6.2 Hz), 14.1.

^{31}P NMR (162 MHz, Chloroform-*d*) δ 25.1.

HRMS (ESI) m/z: $[M + H]^+$ Calcd for $C_{29}H_{38}ClNO_6P^+$ 562.2120, Found: 562.2123.

(*R*)-2-(4-isobutylphenyl)propyl 3-((diethoxyphosphoryl)methyl)-2-(4-methoxyphenyl)quinoline-6-carboxylate (3an)

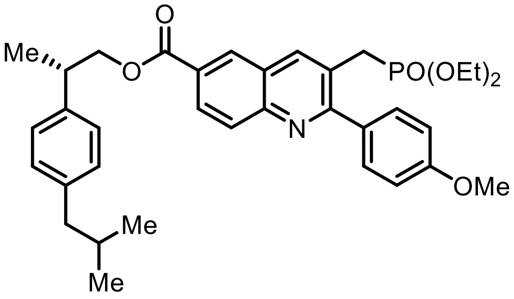

Following procedure A: T_1 = 80 ºC, 28.4 mg, yellow oil, 47% yield.

^{1}H NMR (400 MHz, Chloroform-*d*) δ 8.54 (d, *J* = 1.9 Hz, 1H), 8.45 (d, *J* = 3.4 Hz, 1H), 8.25 – 8.16 (m, 1H), 8.11 (d, *J* = 8.9 Hz, 1H), 7.63 (d, *J* = 8.4 Hz, 2H), 7.23 (d, *J* = 7.7 Hz, 2H), 7.12 (d, *J* = 7.7 Hz, 2H), 7.04 (d, *J* = 8.3 Hz, 2H), 4.54 – 4.37 (m, 2H), 4.51 – 4.40 (m, 4H), 3.88 (s, 3H), 3.42 (d, *J* = 22.3 Hz, 2H), 3.31 – 3.25 (m, 1H), 2.45 (d, *J* = 7.2 Hz, 2H), 1.92 – 1.78 (m, 1H), 1.43 (d, *J* = 7.0 Hz, 3H), 1.23 (t, *J* = 7.1 Hz, 6H), 0.89 (d, *J* = 6.6 Hz, 6H).

^{13}C NMR (101 MHz, Chloroform-*d*) δ 166.2, 162.2 (d, *J* = 7.1 Hz), 160.2, 148.7 (d, *J* = 2.5 Hz), 140.3, 140.27, 139.3 (d, *J* = 6.1 Hz), 132.3, 130.8, 130.5, 129.6, 129.4, 129.1, 128.3, 127.2, 126.2 (d, *J* = 3.2 Hz), 124.7 (d, *J* = 8.6 Hz), 114.1, 70.5, 62.4 (d, *J* = 6.8 Hz), 55.5, 45.1, 38.8, 30.3, 30.1 (d, *J* = 139.1 Hz), 22.5, 22.49, 18.2, 16.5 (d, *J* = 6.1 Hz).

^{31}P NMR (162 MHz, Chloroform-*d*) δ 25.7.

HRMS (ESI) m/z: $[M + H]^+$ Calcd for $C_{35}H_{43}NO_6P^+$ 604.2823, Found: 604.2825.

diethyl ((2-(p-tolyl)quinolin-3-yl)methyl)phosphonate (3ao)

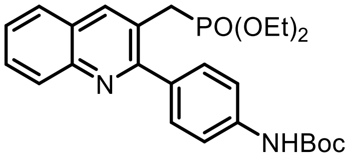

Following procedure A: T_1 = 80 ºC, 35.2 mg, yellow solid, 74% yield. M.p = 193 – 195 ºC.

^{1}H NMR (400 MHz, Chloroform-*d*) δ 8.41 (d, *J* = 3.4 Hz, 1H), 8.10 (d, *J* = 8.5 Hz, 1H), 7.82 (d, *J* = 8.1 Hz, 1H), 7.68 (t, *J* = 7.7 Hz, 1H), 7.59 (d, *J* = 8.5 Hz, 2H), 7.55 – 7.46 (m, 3H), 6.86 (s, 1H), 4.03 – 3.93 (m, 4H), 3.37 (d, *J* = 22.2 Hz, 2H), 1.52 (s, 9H), 1.21 (t, *J* = 7.1 Hz, 6H).

^{13}C NMR (101 MHz, Chloroform-*d*) δ 159.9 (d, *J* = 7.5 Hz), 152.8, 146.9, 138.8, 138.2 (d, *J* = 6.1 Hz), 134.7, 130.2, 129.7, 129.3, 127.4, 127.2 (d, *J* = 3.2 Hz), 126.7, 123.6 (d, *J* = 8.3 Hz), 118.3, 80.8, 62.3 (d, *J* = 6.8 Hz), 29.9 (d, *J* = 139.3 Hz), 28.5, 16.5 (d, *J* = 5.9 Hz).

^{31}P NMR (162 MHz, Chloroform-*d*) δ 26.0.

HRMS (ESI) m/z: $[M + H]^+$ Calcd for $C_{25}H_{32}N_2O_5P^+$ 471.2043; Found: 471.2045.

methyl 3-(diethoxyphosphoryl)-4-phenylfuro[3,2-*c*]quinoline-2-carboxylate (3ap)

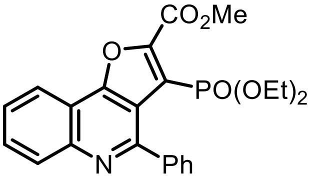

Following procedure B, 14.2 mg, white solid, 32% yield. M.p = 104 – 106 ºC.

^{1}H NMR (400 MHz, Chloroform-*d*) δ 8.39 (d, *J* = 8.1 Hz, 1H), 8.26 (d, *J* = 8.4 Hz, 1H), 7.85 – 7.78 (m, 3H), 7.68 (t, *J* = 7.6 Hz, 1H), 7.56 – 7.45 (m, 3H), 4.08 (s, 3H), 3.94 – 3.78 (m, 4H), 1.14 (t, *J* = 7.1 Hz, 6H).

^{13}C NMR (101 MHz, Chloroform-*d*) δ 159.7, 156.8 (d, *J* = 13.8 Hz), 156.6, 152.0 (d, *J* = 22.6 Hz), 146.2, 140.7, 130.3, 129.8, 129.4, 129.2, 128.3, 127.5, 120.5, 118.2 (d, *J* = 9.9 Hz), 115.4, 113.8 (d, *J* = 211.4 Hz), 62.7 (d, *J* = 6.0 Hz), 53.5, 16.2 (d, *J* = 6.8 Hz).

^{31}P NMR (162 MHz, Chloroform-*d*) δ 8.0.

HRMS (ESI) m/z: $[M + H]^+$ Calcd for $C_{23}H_{23}NO_6P^+$ 440.1258, Found: 440.1267.

diethyl ((6-phenylpyridin-3-yl)methyl)phosphonate (5a)

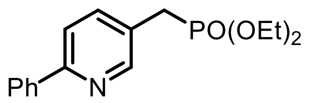

Following procedure C: T_2 = 100 ºC, 19.6 mg, white solid, 64% yield.

^{1}H NMR (400 MHz, Chloroform-*d*) δ 8.59 – 8.54 (m, 1H), 8.02 – 7.94 (m, 2H), 7.77 – 7.67 (m, 2H), 7.50 – 7.43 (m, 2H), 7.43 – 7.37 (m, 1H), 4.10 – 4.03 (m, 4H), 3.17 (d, *J* = 21.6 Hz, 2H), 1.27 (t, *J* = 7.1 Hz, 6H).

^{13}C NMR (101 MHz, Chloroform-*d*) δ 156.2 (d, *J* = 3.9 Hz), 150.5 (d, *J* = 7.6 Hz), 139.1 (d, *J* = 1.5 Hz), 138.1 (d, *J* = 5.7 Hz), 129.1, 128.9, 126.9, 126.3 (d, *J* = 9.3 Hz), 120.4 (d, *J* = 3.1 Hz), 62.5 (d, *J* = 6.7 Hz), 30.9 (d, *J* = 140.0 Hz), 16.5 (d, *J* = 5.9 Hz).

^{31}P NMR (162 MHz, Chloroform-*d*) δ 25.2.

HRMS (ESI) m/z: $[M + H]^+$ Calcd for $C_{16}H_{21}NO_3P^+$ 306.1254, Found: 306.1248.

diethyl ((6-(*p*-tolyl)pyridin-3-yl)methyl)phosphonate (5b)

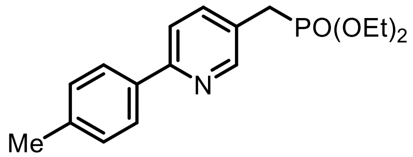

Following procedure C: T_2 = 100 ºC, 17.9 mg, yellow oil, 56% yield.

^{1}H NMR (400 MHz, Chloroform-*d*) δ 8.56 – 8.52 (m, 1H), 7.91 – 7.84 (m, 2H), 7.72 (dt, *J* = 8.3, 2.4 Hz, 1H), 7.66 (d, *J* = 8.2 Hz, 1H), 7.27 (d, *J* = 8.4 Hz, 2H), 4.10 – 4.02 (m, 4H), 3.15 (d, *J* = 21.5 Hz, 2H), 2.40 (s, 3H), 1.27 (t, *J* = 7.1 Hz, 6H).

^{13}C NMR (101 MHz, Chloroform-*d*) δ 156.2 (d, *J* = 3.8 Hz), 150.4 (d, *J* = 7.6 Hz), 139.1, 138.0 (d, *J* = 5.7 Hz), 136.3 (d, *J* = 1.5 Hz), 129.6, 126.8, 126.0 (d, *J* = 9.3 Hz), 120.1 (d, *J* = 3.1 Hz), 62.5 (d, *J* = 6.7 Hz), 30.8 (d, *J* = 140.0 Hz), 21.4, 16.5 (d, *J* = 6.1 Hz).

^{31}P NMR (162 MHz, Chloroform-*d*) δ 25.3.

HRMS (ESI) m/z: $[M + H]^+$ Calcd for $C_{17}H_{23}NO_3P^+$ 320.1410, Found: 320.1411.

diethyl ((6-(4-bromophenyl)pyridin-3-yl)methyl)phosphonate (5c)

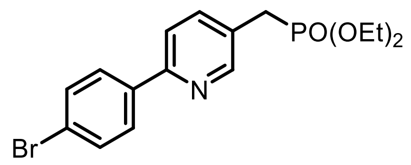

Following procedure C: T_2 = 100 ºC, 25.3 mg, yellow oil, 65% yield.

^{1}H NMR (400 MHz, Chloroform-*d*) δ 8.55 (t, *J* = 2.5 Hz, 1H), 7.88 – 7.83 (m, 2H), 7.74 (dt, *J* = 8.3, 2.4 Hz, 1H), 7.66 (d, *J* = 8.2 Hz, 1H), 7.60 – 7.56 (m, 2H), 4.12 – 4.00 (m, 4H), 3.16 (d, *J* = 21.6 Hz, 2H), 1.27 (t, *J* = 7.1 Hz, 6H).

^{13}C NMR (101 MHz, Chloroform-*d*) δ 155.0 (d, *J* = 3.9 Hz), 150.6 (d, *J* = 7.4 Hz), 138.2 (d, *J* = 5.7 Hz), 138.0 (d, *J* = 1.7 Hz), 132.0, 128.5, 126.8 (d, *J* = 9.2 Hz), 123.5, 120.1 (d, *J* = 3.0 Hz), 62.5 (d, *J* = 6.8 Hz), 30.9 (d, *J* = 139.9 Hz), 16.5 (d, *J* = 5.9 Hz).

^{31}P NMR (162 MHz, Chloroform-*d*) δ 25.0.

HRMS (ESI) m/z: $[M + H]^+$ Calcd for $C_{16}H_{20}BrNO_3P^+$ 384.0359, Found: 384.0360.

diethyl ((6-(4-silylphenyl)pyridin-3-yl)methyl)phosphonate (5d)

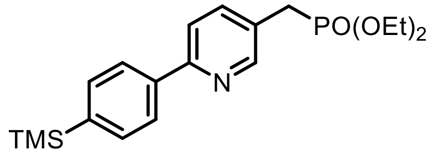

Following procedure C: T_2 = 100 ºC, 21.4 mg, white solid, 56% yield. M.p = 62 – 64 ºC.

^{1}H NMR (400 MHz, Chloroform-*d*) δ 8.57 (t, *J* = 2.5 Hz, 1H), 7.99 – 7.93 (m, 2H), 7.74 (dt, *J* = 8.2, 2.3 Hz, 1H), 7.70 (d, *J* = 8.2 Hz, 1H), 7.64 – 7.60 (m, 2H), 4.10 – 4.03 (m, 4H), 3.16 (d, *J* = 21.6 Hz, 2H), 1.27 (t, *J* = 7.1 Hz, 6H), 0.29 (s, 9H).

^{13}C NMR (101 MHz, Chloroform-*d*) δ 156.2 (d, *J* = 3.8 Hz), 150.5 (d, *J* = 7.5 Hz), 141.6, 139.3 (d, *J* = 1.5 Hz), 138.0 (d, *J* = 5.7 Hz), 133.9, 126.4 (d, *J* = 9.1 Hz), 126.1, 120.4 (d, *J* = 3.1 Hz), 62.5 (d, *J* = 6.8 Hz), 30.9 (d, *J* = 140.0 Hz), 16.5 (d, *J* = 6.0 Hz), -1.0.

^{31}P NMR (162 MHz, Chloroform-*d*) δ 25.2.

HRMS (ESI) m/z: $[M + H]^+$ Calcd for $C_{19}H_{29}NO_3PSi^+$ 378.1649, Found: 378.1654.

diethyl ((6-(3-methoxyphenyl)pyridin-3-yl)methyl)phosphonate (5e)

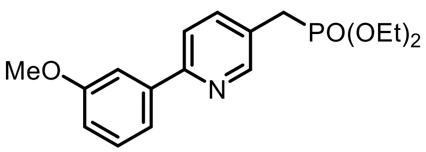

Following procedure C: T_2 = 100 ºC, 20.6 mg, yellow oil, 61% yield.

^{1}H NMR (400 MHz, Chloroform-*d*) δ 8.56 (t, *J* = 2.5 Hz, 1H), 7.74 (dt, *J* = 8.3, 2.4 Hz, 1H), 7.68 (d, *J* = 8.2 Hz, 1H), 7.56 (t, *J* = 2.1 Hz, 1H), 7.52 (dt, *J* = 7.7, 1.3 Hz, 1H), 7.37 (t, *J* = 7.9 Hz, 1H), 6.98 – 6.93 (m, 1H), 4.10 – 4.02 (m, 4H), 3.88 (s, 3H), 3.16 (d, *J* = 21.6 Hz, 2H), 1.27 (t, *J* = 7.1 Hz, 6H).

^{13}C NMR (101 MHz, Chloroform-*d*) δ 160.2, 156.0 (d, *J* = 3.9 Hz), 150.4 (d, *J* = 7.5 Hz), 140.6 (d, *J* = 1.5 Hz), 138.0 (d, *J* = 5.7 Hz), 129.9, 126.5 (d, *J* = 9.2 Hz), 120.5 (d, *J* = 3.0 Hz), 119.3, 115.2, 112.0, 62.5 (d, *J* = 6.8 Hz), 55.5, 30.9 (d, *J* = 140.0 Hz), 16.5 (d, *J* = 6.0 Hz).

^{31}P NMR (162 MHz, Chloroform-*d*) δ 25.2.

HRMS (ESI) m/z: $[M + H]^+$ Calcd for $C_{17}H_{23}NO_4P^+$ 336.1359, Found: 336.1360.

diethyl ((6-(o-tolyl)pyridin-3-yl)methyl)phosphonate (5f)

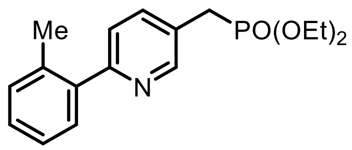

Following procedure C: T_2 = 100 ºC, 15.1 mg, yellow oil, 47% yield.

^{1}H NMR (400 MHz, Chloroform-*d*) δ 8.57 (t, *J* = 2.5 Hz, 1H), 7.74 (dt, *J* = 8.1, 2.4 Hz, 1H), 7.38 (td, *J* = 7.0, 6.2, 1.7 Hz, 2H), 7.30 – 7.25 (m, 3H), 4.12 – 4.04 (m, 4H), 3.18 (d, *J* = 21.6 Hz, 2H), 2.35 (s, 3H), 1.27 (t, *J* = 7.1 Hz, 6H).

^{13}C NMR (101 MHz, Chloroform-*d*) δ 158.7 (d, *J* = 3.8 Hz), 150.0 (d, *J* = 7.5 Hz), 140.1, 137.5 (d, *J* = 5.6 Hz), 135.9, 130.9, 129.7, 128.4, 126.0, 125.8 (d, *J* = 9.1 Hz), 123.9 (d, *J* = 3.0 Hz), 62.5 (d, *J* = 6.7 Hz), 30.9 (d, *J* = 140.0 Hz), 20.4, 16.5 (d, *J* = 6.0 Hz).

^{31}P NMR (162 MHz, Chloroform-*d*) δ 25.3.

HRMS (ESI) m/z: $[M + H]^+$ Calcd for $C_{17}H_{23}NO_3P^+$ 320.1410, Found: 320.1410.

diethyl ((6-(2,4-difluorophenyl)pyridin-3-yl)methyl)phosphonate (5g)

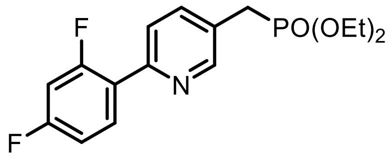

Following procedure C: T_2 = 100 ºC, 17.2 mg, yellow oil, 50% yield.

^{1}H NMR (400 MHz, Chloroform-*d*) δ 8.58 (d, *J* = 2.4 Hz, 1H), 8.02 – 7.96 (m, 1H), 7.77 – 7.67 (m, 2H), 6.99 (td, *J* = 8.2, 2.4 Hz, 1H), 6.90 (ddd, *J* = 11.3, 8.8, 2.5 Hz, 1H), 4.12 – 4.03 (m, 4H), 3.17 (d, *J* = 21.6 Hz, 2H), 1.28 (t, *J* = 7.0 Hz, 6H).

^{13}C NMR (101 MHz, Chloroform-*d*) δ 163.4 (dd, *J* = 251.9, 12.2 Hz), 160.8 (dd, *J* = 252.7, 11.3 Hz), 151.2 (t, *J* = 2.8 Hz), 150.6 (d, *J* = 7.3 Hz), 137.8 (d, *J* = 5.8 Hz), 132.1 (dd, *J* = 9.5, 4.5 Hz), 126.8 (d, *J* = 9.2 Hz), 124.0 (dd, *J* = 9.6, 3.0 Hz), 123.6 – 123.3 (m), 112.0 (dd, *J* = 21.1, 3.6 Hz), 104.5 (t, *J* = 26.3 Hz), 62.5 (d, *J* = 6.8 Hz), 30.9 (d, *J* = 140.0 Hz), 16.5 (d, *J* = 5.9 Hz).

^{19}F NMR (376 MHz, Chloroform-*d*) δ -109.2 (d, *J* = 8.4 Hz, 1F), -112.7 (d, *J* = 8.5 Hz, 1F).

^{31}P NMR (162 MHz, Chloroform-*d*) δ 25.0.

HRMS (ESI) m/z: $[M + H]^+$ Calcd for $C_{16}H_{19}F_2NO_3P^+$ 342.1065, Found: 342.1069.

diethyl ((6-(phenyl-*d*5)pyridin-3-yl)methyl)phosphonate (5h)

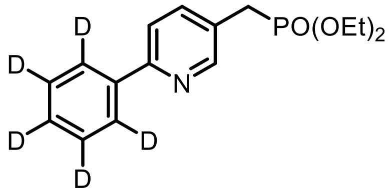

Following procedure C: T_2 = 100 ºC, 16.4 mg, yellow oil, 52% yield.

^{1}H NMR (400 MHz, Chloroform-*d*) δ 8.57 (t, *J* = 2.5 Hz, 1H), 7.75 (dt, *J* = 8.2, 2.4 Hz, 1H), 7.69 (d, *J* = 8.2 Hz, 1H), 4.13 – 4.00 (m, 4H), 3.17 (d, *J* = 21.6 Hz, 2H), 1.27 (t, *J* = 7.1 Hz, 6H).

^{13}C NMR (101 MHz, Chloroform-*d*) δ 156.2 (d, *J* = 3.8 Hz), 150.5 (d, *J* = 7.5 Hz), 138.9, 138.1 (d, *J* = 5.7 Hz), 128.6, 128.3, 126.8, 126.3 (d, *J* = 9.2 Hz), 120.4 (d, *J* = 3.0 Hz), 62.5 (d, *J* = 6.7 Hz), 30.9 (d, *J* = 140.0 Hz), 16.5 (d, *J* = 6.0 Hz).

^{31}P NMR (162 MHz, Chloroform-*d*) δ 25.2.

HRMS (ESI) m/z: $[M + H]^+$ Calcd for $C_{16}H_{16}D_5NO_3P^+$ 311.1567, Found: 311.1570.

diethyl ((6-(naphthalen-2-yl)pyridin-3-yl)methyl)phosphonate (5i)

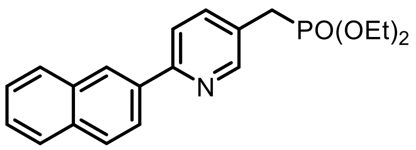

Following procedure C: T_2 = 100 ºC, 22.2 mg, white solid, 62% yield. M.p = 71 – 73 ºC.

^{1}H NMR (400 MHz, Chloroform-*d*) δ 8.62 (d, *J* = 2.5 Hz, 1H), 8.47 (d, *J* = 1.8 Hz, 1H), 8.12 (dd, *J* = 8.6, 1.8 Hz, 1H), 7.94 (d, *J* = 6.8, 2H), 7.88 – 7.83 (m, 2H), 7.79 (dt, *J* = 8.2, 2.4 Hz, 1H), 7.52 – 7.48 (m, 2H), 4.15 – 4.03 (m, 4H), 3.19 (d, *J* = 21.6 Hz, 2H), 1.29 (t, *J* = 7.1 Hz, 6H).

^{13}C NMR (101 MHz, Chloroform-*d*) δ 156.0 (d, *J* = 3.9 Hz), 150.6 (d, *J* = 7.5 Hz), 138.1 (d, *J* = 5.8 Hz), 136.3 (d, *J* = 1.6 Hz), 133.7, 133.6, 128.8, 128.6, 127.8, 126.7, 126.44, 126.4 (d, *J* = 9.2 Hz), 126.3, 124.6, 120.6 (d, *J* = 3.1 Hz), 62.5 (d, *J* = 6.7 Hz), 30.9 (d, *J* = 140.0 Hz), 16.5 (d, *J* = 6.0 Hz).

^{31}P NMR (162 MHz, Chloroform-*d*) δ 25.2.

HRMS (ESI) m/z: $[M + H]^+$ Calcd for $C_{20}H_{23}NO_3P^+$ 356.1410, Found: 356.1412.

diethyl ((6-(benzofuran-2-yl)pyridin-3-yl)methyl)phosphonate (5j)

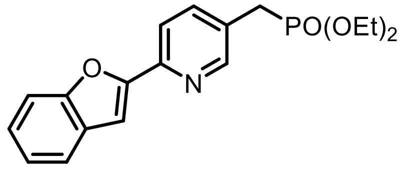

Following procedure C: T_2 = 100 ºC, 18.5 mg, yellow solid, 53% yield. M.p = 50 – 52 ºC.

^{1}H NMR (400 MHz, Chloroform-*d*) δ 8.55 (d, *J* = 2.5 Hz, 1H), 7.86 (d, *J* = 8.2 Hz, 1H), 7.77 (dt, *J* = 8.2, 2.5 Hz, 1H), 7.63 (d, *J* = 7.7 Hz, 1H), 7.56 (d, *J* = 8.2 Hz, 1H), 7.40 (s, 1H), 7.35 – 7.30 (m, 1H), 7.27 – 7.22 (m, 1H), 4.13 – 3.99 (m, 4H), 3.17 (d, *J* = 21.7 Hz, 2H), 1.27 (t, *J* = 7.1 Hz, 6H).

^{13}C NMR (101 MHz, Chloroform-*d*) δ 155.4, 154.9 (d, *J* = 2.2 Hz), 150.7 (d, *J* = 7.5 Hz), 148.0 (d, *J* = 3.9 Hz), 138.0 (d, *J* = 5.9 Hz), 128.9, 127.3 (d, *J* = 9.3 Hz), 125.3,

123.3, 121.8, 119.7 (d, J = 3.1 Hz), 111.6, 104.9, 62.5 (d, J = 6.8 Hz), 31.1 (d, J = 139.9 Hz), 16.5 (d, J = 5.9 Hz).

^{31}P NMR (162 MHz, Chloroform-*d*) δ 24.8.

HRMS (ESI) m/z: $[M + H]^+$ Calcd for $C_{18}H_{21}NO_4P^+$ 346.1203, Found: 346.1207.

diethyl ((6-(thiophen-3-yl)pyridin-3-yl)methyl)phosphonate (5k)

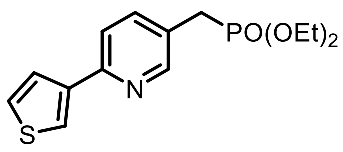

Following procedure C: T_2 = 100 ºC, 16.8 mg, yellow oil, 54% yield.

^{1}H NMR (400 MHz, Chloroform-*d*) δ 8.49 (t, J = 2.6 Hz, 1H), 7.87 (dd, J = 3.2, 1.3 Hz, 1H), 7.70 (dt, J = 8.2, 2.4 Hz, 1H), 7.64 (dd, J = 5.0, 1.3 Hz, 1H), 7.58 (d, J = 8.1 Hz, 1H), 7.39 (dd, J = 5.1, 3.0 Hz, 1H), 4.09 – 4.02 (m, 4H), 3.14 (d, J = 21.6 Hz, 2H), 1.26 (t, J = 7.1 Hz, 6H).

^{13}C NMR (101 MHz, Chloroform-*d*) δ 152.3 (d, J = 3.9 Hz), 150.4 (d, J = 7.6 Hz), 141.9 (d, J = 1.6 Hz), 138.0 (d, J = 5.8 Hz), 126.5, 126.3, 126.0 (d, J = 9.4 Hz), 123.6, 120.1 (d, J = 3.2 Hz), 62.5 (d, J = 6.7 Hz), 30.9 (d, J = 140.1 Hz), 16.5 (d, J = 5.9 Hz).

^{31}P NMR (162 MHz, Chloroform-*d*) δ 25.2.

HRMS (ESI) m/z: $[M + H]^+$ Calcd for $C_{14}H_{19}NO_3PS^+$ 312.0818, Found: 312.0822.

diethyl ((2,6-diphenylpyridin-3-yl)methyl)phosphonate (5l)

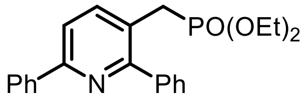

Following procedure C: T_2 = 120 ºC, 28.6 mg, colorless oil, 75% yield.

^{1}H NMR (400 MHz, Chloroform-*d*) δ 8.10 – 8.04 (m, 2H), 7.99 (dd, J = 8.2, 2.5 Hz, 1H), 7.73 – 7.65 (m, 3H), 7.52 – 7.36 (m, 6H), 4.07 – 3.94 (m, 4H), 3.29 (d, J = 22.2 Hz, 2H), 1.24 (t, J = 7.1 Hz, 6H).

^{13}C NMR (101 MHz, Chloroform-*d*) δ 159.0 (d, J = 8.5 Hz), 155.5 (d, J = 3.6 Hz), 140.3, 139.5 (d, J = 4.6 Hz), 139.1 (d, J = 1.5 Hz), 129.6, 129.0, 128.8, 128.4, 128.3, 127.1, 123.8 (d, J = 8.7 Hz), 118.9 (d, J = 3.2 Hz), 62.3 (d, J = 6.8 Hz), 29.8 (d, J = 138.9 Hz), 16.5 (d, J = 6.0 Hz).

^{31}P NMR (162 MHz, Chloroform-*d*) δ 26.1.

HRMS (ESI) m/z: $[M + H]^+$ Calcd for $C_{22}H_{25}NO_3P^+$ 382.1567, Found: 382.1571.

diethyl ((5-methyl-6-phenylpyridin-3-yl)methyl)phosphonate (5m)

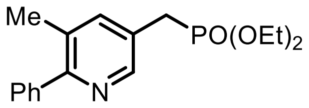

Following procedure C: T_2 = 100 ºC, 11.6 mg, yellow oil, 36% yield.

^{1}H NMR (400 MHz, Chloroform-*d*) δ 8.40 (t, *J* = 2.4 Hz, 1H), 7.60 – 7.56 (m, 1H), 7.53 – 7.48 (m, 2H), 7.44 (ddt, *J* = 8.0, 6.3, 1.1 Hz, 2H), 7.41 – 7.35 (m, 1H), 4.13 – 4.05 (m, 4H), 3.14 (d, *J* = 21.5 Hz, 2H), 2.35 (d, *J* = 0.9 Hz, 3H), 1.29 (t, *J* = 7.1 Hz, 6H).

^{13}C NMR (101 MHz, Chloroform-*d*) δ 157.4 (d, *J* = 3.8 Hz), 147.7 (d, *J* = 7.3 Hz), 140.3, 139.9 (d, *J* = 5.8 Hz), 130.8 (d, *J* = 3.0 Hz), 129.1, 128.3, 128.1, 126.2 (d, *J* = 9.0 Hz), 62.5 (d, *J* = 6.8 Hz), 30.6 (d, *J* = 139.9 Hz), 20.1, 16.5 (d, *J* = 6.1 Hz).

^{31}P NMR (162 MHz, Chloroform-*d*) δ 25.5.

HRMS (ESI) m/z: $[M + H]^+$ Calcd for $C_{17}H_{23}NO_3P^+$ 320.1410, Found: 320.1415.

diethyl ((4,6-diphenylpyridin-3-yl)methyl)phosphonate (5n)

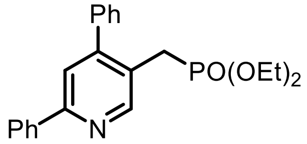

Following procedure C: T_2 = 100 ºC, 18.9 mg, colorless oil, 49% yield.

^{1}H NMR (400 MHz, Chloroform-*d*) δ 8.78 (d, *J* = 2.6 Hz, 1H), 8.04 – 7.96 (m, 2H), 7.63 (s, 1H), 7.51 – 7.39 (m, 8H), 4.08 – 3.95 (m, 4H), 3.23 (d, *J* = 22.2 Hz, 2H), 1.25 (t, *J* = 7.1 Hz, 6H).

^{13}C NMR (101 MHz, Chloroform-*d*) δ 155.9 (d, *J* = 3.8 Hz), 151.6 (d, *J* = 5.0 Hz), 150.9 (d, *J* = 7.4 Hz), 139.0 (d, *J* = 1.6 Hz), 138.8, 129.1, 128.9, 128.7, 128.4, 127.0, 124.0 (d, *J* = 9.1 Hz), 121.8 (d, *J* = 2.9 Hz), 62.3 (d, *J* = 6.8 Hz), 27.9 (d, *J* = 139.8 Hz), 16.5 (d, *J* = 6.1 Hz).

^{31}P NMR (162 MHz, Chloroform-*d*) δ 25.5.

HRMS (ESI) m/z: $[M + H]^+$ Calcd for $C_{22}H_{25}NO_3P^+$ 382.1567, Found: 382.1560.

diethyl ((6-phenyl-4-(phenylethynyl)pyridin-3-yl)methyl)phosphonate (5o)

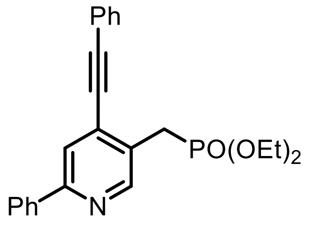

Following procedure C: T_2 = 100 ºC, 18.1 mg, yellow oil, 44% yield.

^{1}H NMR (400 MHz, Chloroform-*d*) δ 8.71 (d, *J* = 2.7 Hz, 1H), 8.03 – 7.98 (m, 2H), 7.84 (s, 1H), 7.63 – 7.57 (m, 2H), 7.52 – 7.46 (m, 2H), 7.45 – 7.37 (m, 4H), 4.16 – 4.06 (m, 4H), 3.50 (d, *J* = 22.0 Hz, 2H), 1.27 (t, *J* = 7.1 Hz, 6H).

^{13}C NMR (101 MHz, Chloroform-*d*) δ 155.9 (d, *J* = 3.9 Hz), 151.1 (d, *J* = 5.5 Hz), 138.6 (d, *J* = 1.8 Hz), 132.4 (d, *J* = 7.0 Hz), 132.0, 129.5, 129.3, 128.9, 128.7, 127.3 (d, *J* = 9.9 Hz), 127.0, 122.6 (d, *J* = 3.1 Hz), 122.2, 97.7, 85.8 (d, *J* = 2.5 Hz), 62.5 (d, *J* = 6.7 Hz), 29.5 (d, *J* = 139.4 Hz), 16.5 (d, *J* = 6.1 Hz).

^{31}P NMR (162 MHz, Chloroform-*d*) δ 24.5.

HRMS (ESI) m/z: $[M + H]^+$ Calcd for $C_{24}H_{25}NO_3P^+$ 406.1567, Found: 406.1573.

diethyl ((2,5,6-triphenylpyridin-3-yl)methyl)phosphonate (5p)

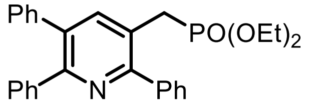

Following procedure C: T_2 = 120 ºC, 20.6 mg, white solid, 45% yield. M.p = 107 – 109 ºC.

^{1}H NMR (400 MHz, Chloroform-*d*) δ 7.93 (d, *J* = 2.6 Hz, 1H), 7.74 – 7.69 (m, 2H), 7.48 (dd, *J* = 8.2, 6.6 Hz, 2H), 7.43 – 7.40 (m, 3H), 7.29 – 7.25 (m, 3H), 7.25 – 7.19 (m, 5H), 4.08 – 3.96 (m, 4H), 3.33 (d, *J* = 22.1 Hz, 2H), 1.26 (t, *J* = 7.1 Hz, 6H).

^{13}C NMR (101 MHz, Chloroform-*d*) δ 157.7 (d, *J* = 8.4 Hz), 155.3 (d, *J* = 3.6 Hz), 141.4 (d, *J* = 4.7 Hz), 140.0, 139.92, 139.6, 134.5 (d, *J* = 3.1 Hz), 130.2, 129.7, 129.66, 128.44, 128.4, 128.3, 127.9, 127.87, 127.3, 123.8 (d, *J* = 8.7 Hz), 62.3 (d, *J* = 6.7 Hz), 29.7 (d, *J* = 139.0 Hz), 16.5 (d, *J* = 6.1 Hz).

^{31}P NMR (162 MHz, Chloroform-*d*) δ 26.0.

HRMS (ESI) m/z: $[M + H]^+$ Calcd for $C_{28}H_{29}NO_3P^+$ 458.1880, Found: 458.1883.

diethyl ((6-chloro-4-phenylpyridin-3-yl)methyl)phosphonate (5q)

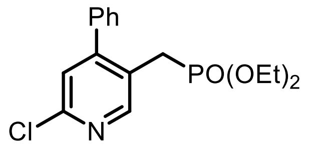

Following procedure C: T_2 = 100 ºC, MeOH (0.5 mL) and Et_3N (0.15 mmol) were added, 12.9 mg, yellow oil, 38% yield.

^{1}H NMR (400 MHz, Chloroform-*d*) δ 8.49 (d, *J* = 2.7 Hz, 1H), 7.51 – 7.38 (m, 5H), 7.24 (s, 1H), 4.06 – 3.93 (m, 4H), 3.14 (d, *J* = 22.1 Hz, 2H), 1.24 (t, *J* = 7.0 Hz, 6H).

^{13}C NMR (101 MHz, Chloroform-*d*) δ 153.1 (d, *J* = 7.3 Hz), 151.5 (d, *J* = 5.1 Hz), 150.0 (d, *J* = 4.0 Hz), 137.3, 128.87, 128.9, 125.0 (d, *J* = 2.7 Hz), 124.8 (d, *J* = 8.8 Hz), 62.4 (d, *J* = 6.8 Hz), 27.5 (d, *J* = 140.2 Hz), 16.5 (d, *J* = 6.1 Hz).

^{31}P NMR (162 MHz, Chloroform-*d*) δ 24.8.

HRMS (ESI) m/z: $[M + H]^+$ Calcd for $C_{16}H_{20}ClNO_3P^+$ 340.0864, Found: 340.0864.

diethyl (*E*)-((6-styrylpyridin-3-yl)methyl)phosphonate (5r)

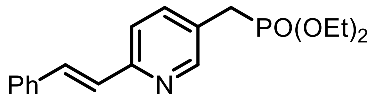

Following procedure C: T_2 = 100 ºC, 8.2 mg, yellow oil, 24% yield.

^{1}H NMR (400 MHz, Chloroform-*d*) δ 8.48 (d, *J* = 2.6 Hz, 1H), 7.66 (dt, *J* = 8.1, 2.5 Hz, 1H), 7.62 (d, *J* = 16.4 Hz, 1H), 7.57 – 7.56 (m, 2H), 7.39 – 7.35 (m, 3H), 7.32 – 7.27 (m, 1H), 7.16 (d, *J* = 16.1 Hz, 1H), 4.13 – 4.00 (m, 4H), 3.14 (d, *J* = 21.6 Hz, 2H), 1.27 (t, *J* = 7.0 Hz, 6H).

^{13}C NMR (101 MHz, Chloroform-*d*) δ 154.4 (d, *J* = 4.0 Hz), 150.4 (d, *J* = 7.6 Hz), 137.9 (d, *J* = 5.8 Hz), 136.7, 132.9 (d, *J* = 1.8 Hz), 128.8, 128.5, 127.6 (d, *J* = 2.3 Hz), 127.2, 126.3 (d, *J* = 9.4 Hz), 121.9 (d, *J* = 3.1 Hz), 62.5 (d, *J* = 6.9 Hz), 31.0 (d, *J* = 140.0 Hz), 16.5 (d, *J* = 5.9 Hz).

^{31}P NMR (162 MHz, Chloroform-*d*) δ 25.1.

HRMS (ESI) m/z: $[M + H]^+$ Calcd for $C_{18}H_{23}NO_3P^+$ 332.1410, Found: 332.1418.

diethyl ((6-phenoxypyridin-3-yl)methyl)phosphonate (5s)

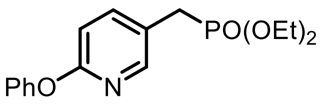

Following procedure C: T_2 = 100 ºC, MeOH (0.5 mL) and Et_3N (0.15 mmol) were added, 15.4 mg, yellow oil, 47% yield.

^{1}H NMR (400 MHz, Chloroform-*d*) δ 8.06 (d, *J* = 2.8 Hz, 1H), 7.68 (dt, *J* = 8.5, 2.4 Hz, 1H), 7.42 – 7.34 (m, 2H), 7.22 – 7.17 (m, 1H), 7.15 – 7.05 (m, 2H), 6.86 (d, *J* = 8.5 Hz, 1H), 4.09 – 4.02 (m, 4H), 3.07 (d, *J* = 21.1 Hz, 2H), 1.27 (t, *J* = 7.1 Hz, 6H).

^{13}C NMR (101 MHz, Chloroform-*d*) δ 163.0 (d, *J* = 3.2 Hz), 154.3, 148.2 (d, *J* = 8.0 Hz), 140.9 (d, *J* = 5.1 Hz), 129.8, 124.8, 122.4 (d, *J* = 9.1 Hz), 121.2, 111.4 (d, *J* = 2.8 Hz), 62.4 (d, *J* = 6.8 Hz), 30.1 (d, *J* = 141.0 Hz), 16.5 (d, *J* = 6.0 Hz).

^{31}P NMR (162 MHz, Chloroform-*d*) δ 25.5.

HRMS (ESI) m/z: $[M + H]^+$ Calcd for $C_{16}H_{21}NO_4P^+$ 322.1203, Found: 322.1212.

diethyl ((4-phenylpyridin-3-yl)methyl)phosphonate (5t)

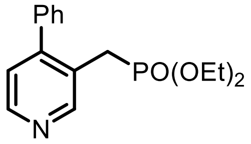

Following procedure C: T_2 = 100 ºC, MeOH (0.5 mL) and Et_3N (0.15 mmol) were added, 9.8 mg, yellow oil, 32% yield.

^{1}H NMR (400 MHz, Chloroform-*d*) δ 8.76 – 8.69 (m, 1H), 8.52 (d, *J* = 4.4 Hz, 1H), 7.50 – 7.38 (m, 5H), 7.21 (d, *J* = 5.0 Hz, 1H), 4.05 – 3.92 (m, 4H), 3.20 (d, *J* = 22.2 Hz, 2H), 1.24 (t, *J* = 7.1 Hz, 6H).

^{13}C NMR (101 MHz, Chloroform-*d*) δ 151.7 (d, *J* = 4.8 Hz), 150.2 (d, *J* = 7.5 Hz), 148.0 (d, *J* = 3.7 Hz), 138.4, 129.1, 128.7, 128.4, 125.6 (d, *J* = 8.9 Hz), 124.9 (d, *J* = 2.7 Hz), 62.3 (d, *J* = 6.7 Hz), 28.0 (d, *J* = 140.2 Hz), 16.5 (d, *J* = 6.1 Hz).

^{31}P NMR (162 MHz, Chloroform-*d*) δ 25.4.

HRMS (ESI) m/z: $[M + H]^+$ Calcd for $C_{16}H_{21}NO_3P^+$ 306.1254, Found: 306.1255.

diethyl ((4,4'-dimethyl-[2,2'-bipyridin]-5-yl)methyl)phosphonate (5u)

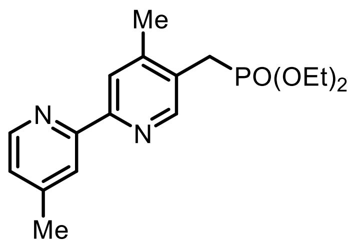

Following procedure C: T_2 = 100 ºC, no $ZnBr_2$ was added, 18.4 mg, yellow oil, 55% yield.

^{1}H NMR (400 MHz, Chloroform-*d*) δ 8.50 – 8.45 (m, 2H), 8.20 (s, 2H), 7.12 (d, J = 5.0 Hz, 1H), 4.08 – 4.01 (m, 4H), 3.22 (d, J = 22.0 Hz, 2H), 2.51 – 2.47 (m, 3H), 2.43 (s, 3H), 1.26 (t, J = 7.0 Hz, 6H).

^{13}C NMR (101 MHz, Chloroform-*d*) δ 155.8, 154.7, 150.6 (d, J = 5.7 Hz), 149.0, 148.4, 147.5 (d, J = 5.9 Hz), 127.2 (d, J = 9.6 Hz), 124.8, 122.8 (d, J = 3.3 Hz), 122.0, 62.4 (d, J = 6.9 Hz), 28.7 (d, J = 140.2 Hz), 21.3, 19.7, 16.5 (d, J = 6.0 Hz).

^{31}P NMR (162 MHz, Chloroform-*d*) δ 25.3.

HRMS (ESI) m/z: $[M + H]^+$ Calcd for $C_{17}H_{24}N_2O_3P^+$ 335.1519, Found: 335.1516.

diethyl ([2,2'-bipyridin]-5-ylmethyl)phosphonate (5v)

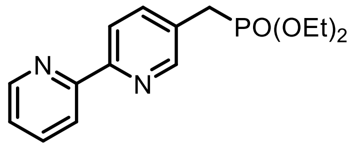

Following procedure C: T_2 = 100 ºC, no $ZnBr_2$ was added, 16.7 mg, yellow oil, 54% yield.

^{1}H NMR (400 MHz, Chloroform-*d*) δ 8.69 – 8.64 (m, 1H), 8.55 (d, J = 2.6 Hz, 1H), 8.34 (t, J = 7.3 Hz, 2H), 7.82 – 7.76 (m, 2H), 7.29 (ddd, J = 7.5, 4.8, 1.1 Hz, 1H), 4.10 – 3.99 (m, 4H), 3.18 (d, J = 21.7 Hz, 2H), 1.25 (t, J = 7.1 Hz, 6H).

^{13}C NMR (101 MHz, Chloroform-*d*) δ 155.8, 154.8 (d, J = 3.8 Hz), 150.0 (d, J = 7.4 Hz), 149.2, 138.1 (d, J = 5.8 Hz), 137.0, 128.1 (d, J = 9.1 Hz), 123.7, 121.1, 120.8 (d, J = 3.1 Hz), 62.4 (d, J = 6.8 Hz), 31.0 (d, J = 139.8 Hz), 16.4 (d, J = 6.1 Hz).

^{31}P NMR (162 MHz, Chloroform-*d*) δ 24.9.

HRMS (ESI) m/z: $[M + H]^+$ Calcd for $C_{15}H_{20}N_2O_3P^+$ 307.1206, Found: 307.1210.

diethyl ((5'-(2-cyanophenyl)-6'-oxo-1'-phenyl-1',6'-dihydro-[2,3'-bipyridin]-5-

yl)methyl)phosphonate (5w)

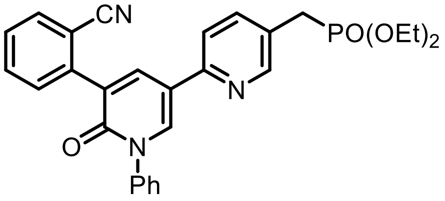

Following procedure C: T_2 = 100 ºC, 26.5 mg, yellow solid, 53% yield. M.p = 70 – 72 ºC.

^{1}H NMR (400 MHz, Chloroform-*d*) δ 8.46 (d, *J* = 2.4 Hz, 1H), 8.32 – 8.26 (m, 2H), 7.80 – 7.70 (m, 3H), 7.62 (t, *J* = 7.8 Hz, 1H), 7.58 – 7.49 (m, 5H), 7.44 (t, *J* = 7.6 Hz, 2H), 4.09 – 4.02 (m, 4H), 3.14 (d, *J* = 21.6 Hz, 2H), 1.27 (t, *J* = 7.1 Hz, 6H).

^{13}C NMR (101 MHz, Chloroform-*d*) δ 160.5, 151.7 (d, *J* = 3.7 Hz), 150.5 (d, *J* = 7.5 Hz), 141.0, 140.4, 138.9, 138.4 (d, *J* = 5.8 Hz), 138.0, 133.3, 132.4, 131.2, 129.5, 129.1, 128.9, 128.4, 126.8, 126.5 (d, *J* = 9.4 Hz), 118.7, 118.6 (d, *J* = 3.0 Hz), 118.0, 112.6, 62.5 (d, *J* = 6.8 Hz), 30.8 (d, *J* = 140.0 Hz), 16.5 (d, *J* = 6.0 Hz).

^{31}P NMR (162 MHz, Chloroform-*d*) δ 24.9.

HRMS (ESI) m/z: $[M + H]^+$ Calcd for $C_{28}H_{27}N_3O_4P^+$ 500.1734, Found: 500.1733.

ethyl 4-(8-chloro-3-((diethoxyphosphoryl)methyl)-5,6-dihydro-11H-benzo[5,6]cyclohepta[1,2-*b*]pyridin-11-ylidene)piperidine-1-carboxylate (5x)

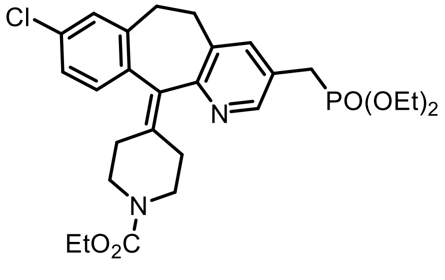

Following procedure C: T_2 = 100 ºC, 30.8 mg, yellow solid, 57% yield. M.p = 126 – 127 ºC.

^{1}H NMR (400 MHz, Chloroform-*d*) δ 8.28 (t, *J* = 2.3 Hz, 1H), 7.44 (s, 1H), 7.18 – 7.06 (m, 3H), 4.16 – 4.01 (m, 6H), 3.80 (s, 2H), 3.41 – 3.27 (m, 2H), 3.16 – 3.09 (m, 2H), 3.05 (d, *J* = 21.5 Hz, 2H), 2.90 – 2.73 (m, 2H), 2.50 – 2.43 (m, 1H), 2.34 – 2.27 (m, 3H), 1.27 – 1.23(m, 9H).

^{13}C NMR (101 MHz, Chloroform-*d*) δ 155.6, 155.4 (d, *J* = 3.7 Hz), 147.4 (d, *J* = 7.3 Hz), 139.7, 139.0 (d, *J* = 5.9 Hz), 137.9 (d, *J* = 5.1 Hz), 133.9, 133.3 (d, *J* = 3.0 Hz), 133.0, 130.5, 129.0, 126.4 (d, *J* = 8.8 Hz), 126.3, 62.4 (d, *J* = 6.8 Hz), 62.3 (d, *J* = 6.8 Hz), 61.4, 44.9, 44.86, 31.6 (d, *J* = 2.8 Hz), 30.9, 30.6, 30.56 (d, *J* = 140.1 Hz), 29.8, 16.5 (d, *J* = 6.0 Hz), 14.8.

^{31}P NMR (162 MHz, Chloroform-*d*) δ 25.2.

HRMS (ESI) m/z: $[M + H]^+$ Calcd for $C_{27}H_{35}ClN_2O_5P^+$ 533.1967, Found: 533.1963.

4-(5-((diethoxyphosphoryl)methyl)pyridin-2-yl)phenyl 4-(*N*,*N*-dipropylsulfamoyl)benzoate (5y)

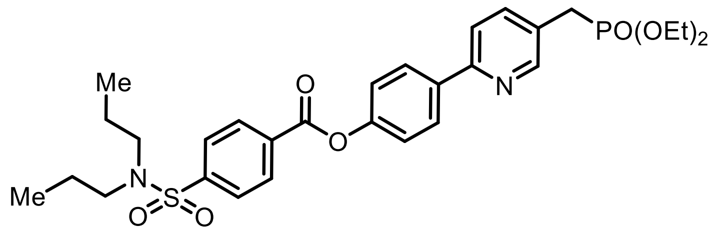

Following procedure C: T_2 = 100 ºC, 31.1 mg, white solid, 52% yield. M.p = 69 – 71 ºC.

^{1}H NMR (400 MHz, Chloroform-*d*) δ 8.57 (d, *J* = 2.6 Hz, 1H), 8.37 – 8.30 (m, 2H), 8.09 – 8.04 (m, 2H), 7.98 – 7.92 (m, 2H), 7.76 (dt, *J* = 8.3, 2.4 Hz, 1H), 7.70 (d, *J* = 8.2 Hz, 1H), 7.36 – 7.30 (m, 2H), 4.11 – 4.03 (m, 4H), 3.17 (d, *J* = 22.1 Hz, 2H), 3.14 – 3.08 (m, 4H), 1.62 – 1.50 (m, 4H), 1.27 (t, *J* = 7.1 Hz, 6H), 0.88 (t, *J* = 7.4 Hz, 6H).

^{13}C NMR (101 MHz, Chloroform-*d*) δ 163.9, 155.1 (d, *J* = 3.8 Hz), 151.4, 150.6 (d, *J* = 7.5 Hz), 145.1, 138.2 (d, *J* = 5.8 Hz), 137.2, 132.8, 130.9, 128.2, 127.3, 126.6 (d, *J* = 9.3 Hz), 121.9, 120.3 (d, *J* = 3.0 Hz), 62.5 (d, *J* = 6.8 Hz), 50.0, 30.8 (d, *J* = 140.0 Hz), 22.0, 16.5 (d, *J* = 6.0 Hz), 11.3.

^{31}P NMR (162 MHz, Chloroform-*d*) δ 25.1.

HRMS (ESI) m/z: $[M + H]^+$ Calcd for $C_{29}H_{38}N_2O_7PS^+$ 589.2132, Found: 589.2132.

4-(5-((diethoxyphosphoryl)methyl)pyridin-2-yl)phenyl 2-(4-isobutylphenyl)propanoate (5z)

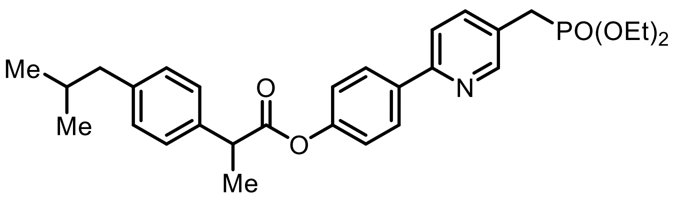

Following procedure C: T_2 = 100 ºC, 27.9 mg, white solid, 54% yield. M.p = 89 – 91 ºC.

^{1}H NMR (400 MHz, Chloroform-*d*) δ 8.56 – 8.52 (m, 1H), 7.98 – 7.93 (m, 2H), 7.73 (dt, *J* = 8.2, 2.4 Hz, 1H), 7.64 (d, *J* = 8.2 Hz, 1H), 7.33 – 7.29 (m, 2H), 7.17 – 7.13 (m, 2H), 7.12 – 7.08 (m, 2H), 4.10 – 4.02 (m, 4H), 3.95 (q, *J* = 7.1 Hz, 1H), 3.15 (d, *J* = 21.6 Hz, 2H), 2.47 (d, *J* = 7.2 Hz, 2H), 1.92 – 1.85 (m, 1H), 1.61 (d, *J* = 7.1 Hz, 3H), 1.26 (t, *J* = 7.1 Hz, 6H), 0.91 (d, *J* = 6.6 Hz, 6H).

^{13}C NMR (101 MHz, Chloroform-*d*) δ 173.2, 155.3 (d, *J* = 3.9 Hz), 151.7, 150.5 (d, *J* = 7.4 Hz), 141.0, 138.1 (d, *J* = 5.8 Hz), 137.3, 136.6 (d, *J* = 1.5 Hz), 129.7, 127.9, 127.3, 126.4 (d, *J* = 9.1 Hz), 121.8, 120.2 (d, *J* = 3.0 Hz), 62.5 (d, *J* = 6.7 Hz), 45.4, 45.2, 30.8 (d, *J* = 140.0 Hz), 30.3, 22.5, 18.7, 16.5 (d, *J* = 5.9 Hz).

^{31}P NMR (162 MHz, Chloroform-*d*) δ 25.1.

HRMS (ESI) m/z: $[M + H]^+$ Calcd for $C_{29}H_{37}NO_5P^+$ 510.2404, Found: 510.2406.

2-phenyl-5-((phenylsulfonyl)methyl)pyridine (5aa)

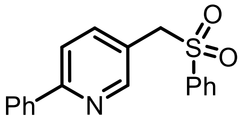

Following procedure D, 16.5 mg, white solid, 53% yield. M.p = 181 – 183 ºC.

^{1}H NMR (400 MHz, Chloroform-*d*) δ 8.16 (d, *J* = 2.2 Hz, 1H), 7.92 – 7.86 (m, 2H), 7.65 – 7.52 (m, 5H), 7.45 – 7.34 (m, 5H), 4.28 (s, 2H).

^{13}C NMR (101 MHz, Chloroform-*d*) δ 157.7, 151.1, 139.0, 138.4, 137.5, 134.2, 129.5, 129.3, 128.9, 128.6, 127.0, 122.7, 120.3, 59.9.

HRMS (ESI) m/z: $[M + H]^+$ Calcd for $C_{18}H_{16}NO_2S^+$ 310.0896, Found: 310.0910.

4. One-pot synthesis of 3a from 2-phenylquinoline and gram-scale reaction.

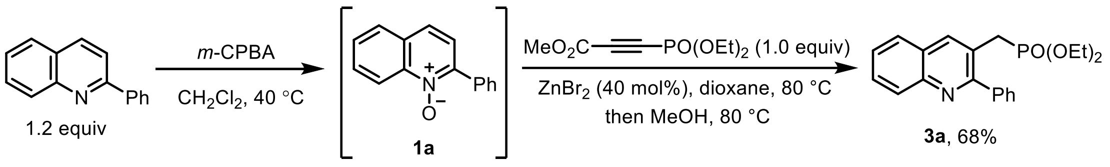

To a solution of 2-phenylquinoline (24.6 mg, 0.12 mmol) in CH_2Cl_2 (1 mL) was added *m*-CPBA (31.1 mg, 0.36 mmol), and the mixture was stirred at 40 ºC for 36 h.

The solvent was then removed in vacuo, to the residue were added alkyne **2a** (22.1 mg, 0.10 mmol), $ZnBr_2$ (9.0 mg, 0.04 mmol), and dioxane (1 mL). The reaction mixture was stirred at 80 ºC for 24 h, followed by the addition of MeOH (0.5 mL) and stirring for another 12 h. After the reaction was completed, the solvent was removed in vacuo and the crude product was purified by column chromatography on silica gel using PE/EtOAc (v/v = 2:1) as eluent to provide **3a** as yellow oil (24.2 mg, 68% yield).

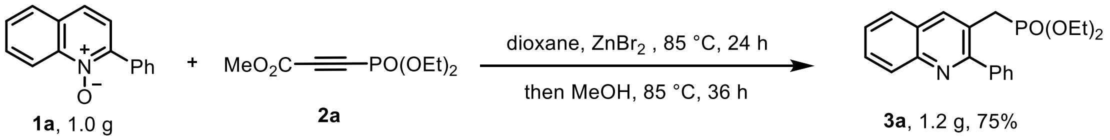

An oven-dried flask was charged with **1a** (1.0 g, 4.52 mmol), **2a** (1.5 g, 6.78 mmol), $ZnBr_2$ (0.4 g, 1.8 mmol) and dioxane (45 mL). The mixture was stirred at 85 ºC for 24 h. Then MeOH (20 mL) was added and the reaction was allowed to proceed for an additional 36 h. Upon completion of the reaction, the solvent was removed and product **3a** was obtained as yellow oil (1.2 g, 75% yield) by column chromatography on silica gel using PE/EtOAc (v/v = 2:1) as eluent.

5. Synthetic applications

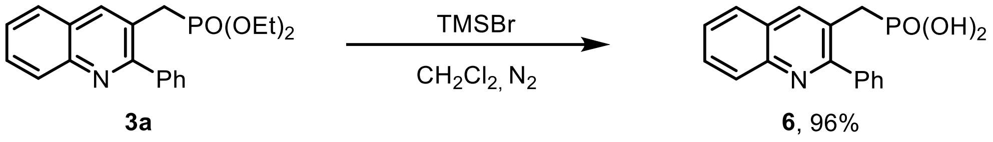

Prepared according to the reported method.[1] To a solution of **3a** (36.0 mg, 0.1 mmol) in CH_2Cl_2 (1 mL) was added dropwise a solution of bromotrimethylsilane (TMSBr, 76.5 mg, 0.5 mmol) in CH_2Cl_2 (1 mL) under a nitrogen atmosphere. The mixture was stirred at room temperature for 12 h. The solvent was then removed under reduced pressure, and the residue was treated with MeOH (1 mL) and stirred for an additional 1 h. After solvent removal under reduced pressure, the resulting solid was washed with cold EtOAc to afford the desired product **6**.

((2-phenylquinolin-3-yl)methyl)phosphonic acid (6)

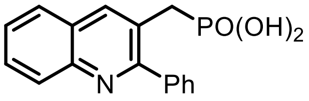

28.8 mg, white solid, 96% yield. M.p = 275 -278 ºC.

^{1}H NMR (400 MHz, Methanol-*d*4) δ 9.28 (d, *J* = 3.1 Hz, 1H), 8.38 (d, *J* = 8.3 Hz, 1H), 8.27 (d, *J* = 8.6 Hz, 1H), 8.19 (t, *J* = 7.8 Hz, 1H), 8.02 (t, *J* = 7.7 Hz, 1H), 7.92 – 7.85 (m, 2H), 7.80 – 7.70 (m, 3H), 3.46 (d, *J* = 21.7 Hz, 2H).

^{13}C NMR (101 MHz, Methanol-*d*4) δ 156.4 (d, *J* = 6.6 Hz), 148.0 (d, *J* = 5.2 Hz), 137.2 (d, *J* = 1.9 Hz), 135.0, 131.5, 131.0, 130.0, 129.6, 129.0, 128.6, 128.2 (d, *J* = 8.1 Hz), 128.0 (d, *J* = 3.0 Hz), 119.7, 30.1 (d, *J* = 135.7 Hz).

^{31}P NMR (162 MHz, Methanol-*d*4) δ 20.4.

HRMS (ESI) m/z: $[M + H]^+$ Calcd for $C_{16}H_{15}NO_3P^+$ 300.0784, Found: 300.0789.

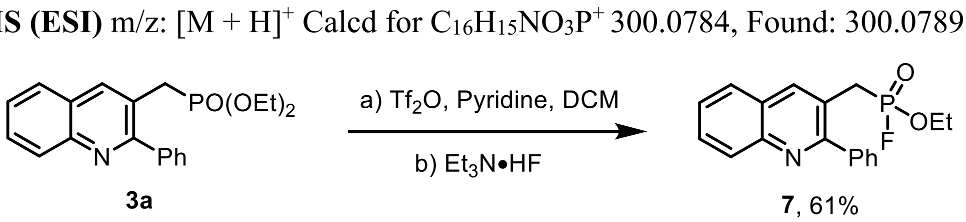

Prepared according to the reported method.[2] Trifluoromethanesulfonic anhydride (Tf_2O, 93.1 mg, 0.33 mmol) was added to a stirred solution of compound **3a** (39.1 mg, 0.11 mmol) in CH_2Cl_2 (1 mL) at room temperature, and the resulting mixture was stirred

for 5 minutes. Subsequently, pyridine (34.8 mg, 0.44 mmol) was introduced to the reaction system, and stirring was continued for an additional 5 minutes. Upon the addition of triethylamine trihydrofluoride ($Et_3N{\cdot}3HF$, 17.7 mg, 0.11 mmol), the reaction mixture was allowed to react for 15 minutes at room temperature. Thereafter, Na_2CO_3 (35.0 mg, 0.33 mmol) and methanol (MeOH, 1 mL) were added sequentially, and the resulting solution was stirred for a further 15 minutes at room temperature to complete the reaction. Finally, the crude product was purified by column chromatography on silica gel using PE/EtOAc (v/v = 1:2) as the eluent, affording the target product **7**.

ethyl ((2-phenylquinolin-3-yl)methyl)phosphonofluoridate (7)

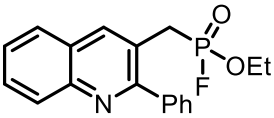

22.9 mg, yellow oil, 61% yield.

^{1}H NMR (400 MHz, Chloroform-*d*) δ 8.37 (d, *J* = 3.6 Hz, 1H), 8.14 (d, *J* = 8.5 Hz, 1H), 7.86 (d, *J* = 8.2 Hz, 1H), 7.74 (dd, *J* = 8.5, 6.9 Hz, 1H), 7.60 – 7.45 (m, 6H), 4.22 – 4.14 (m, 2H), 3.59 – 3.49 (m, 2H), 1.27 (t, *J* = 7.1 Hz, 3H).

^{13}C NMR (101 MHz, Chloroform-*d*) δ 160.2 (d, *J* = 7.9 Hz), 147.1 (d, *J* = 2.7 Hz), 139.9, 138.2 (d, *J* = 6.6 Hz), 130.2 (d, *J* = 1.7 Hz), 129.5 (d, *J* = 1.6 Hz), 129.1, 128.8, 128.7, 127.4 (d, *J* = 1.4 Hz), 127.2 (d, *J* = 3.3 Hz), 127.15 (d, *J* = 1.3 Hz), 121.5 (d, *J* = 9.2 Hz), 64.2 (d, *J* = 7.3 Hz), 28.8 (dd, *J* = 143.9, 24.8 Hz), 16.4 (d, *J* = 5.7 Hz).

^{19}F NMR (376 MHz, Chloroform-*d*) δ -62.6 (d, *J* = 1078.6 Hz, 1F).

^{31}P NMR (162 MHz, Chloroform-*d*) δ 24.3 (d, *J* = 1078.4 Hz, 1P).

HRMS (ESI) m/z: $[M + H]^+$ Calcd for $C_{18}H_{18}FNO_2P^+$ 330.1054, Found: 330.1053.

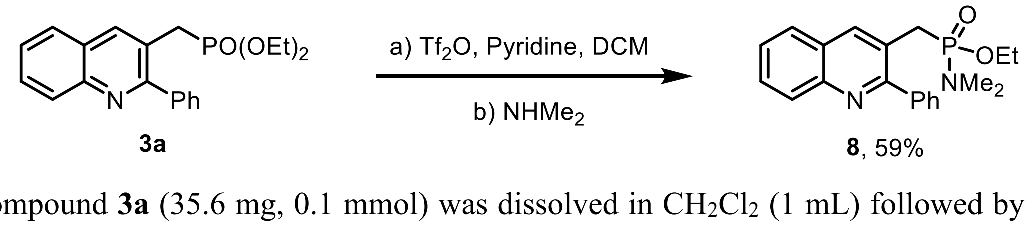

3a **8**, 59%

Compound **3a** (35.6 mg, 0.1 mmol) was dissolved in CH_2Cl_2 (1 mL) followed by the addition of Tf_2O (84.6 mg, 0.3 mmol), the reaction was stirred for 10 min at room temperature. After adding pyridine (31.6 mg, 0.4 mmol), the mixture was stirred for a

further 10 min. Then a 2M solution of dimethylamine in THF (0.25 mL, 0.5 mmol) was added and the resulting solution was stirred at 50 ºC for 1 h. Upon completion of the reaction, the solvent was removed under reduced pressure. The resulting residue was then purified by column chromatography on silica gel using CH_2Cl_2/MeOH (v/v =100:1) as the eluent to give the target product **8**.

ethyl *N*,*N*-dimethyl-*P*-((2-phenylquinolin-3-yl)methyl)phosphonamidite (8)

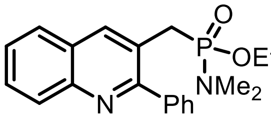

21.0 mg, yellow oil, 59% yield.

^{1}H NMR (400 MHz, Chloroform-*d*) δ 8.40 (d, *J* = 3.3 Hz, 1H), 8.12 (d, *J* = 8.5 Hz, 1H), 7.85 (d, *J* = 8.1 Hz, 1H), 7.70 (t, *J* = 7.7 Hz, 1H), 7.64 – 7.59 (m, 2H), 7.57 – 7.41 (m, 4H), 3.99 – 3.91 (m, 1H), 3.85 – 3.76 (m, 1H), 3.42 – 3.22 (m, 2H), 2.39 (s, 3H), 2.37 (s, 3H), 1.22 (t, *J* = 7.1 Hz, 3H).

^{13}C NMR (101 MHz, Chloroform-*d*) δ 160.2 (d, *J* = 6.7 Hz), 146.8 (d, *J* = 2.5 Hz), 140.5, 138.3 (d, *J* = 5.8 Hz), 129.5, 129.4, 129.35, 128.5, 128.4, 127.5, 127.4 (d, *J* = 3.1 Hz), 126.7, 124.4 (d, *J* = 7.9 Hz), 59.9 (d, *J* = 6.9 Hz), 36.1 (d, *J* = 4.3 Hz), 29.7 (d, *J* = 126.6 Hz), 16.3 (d, *J* = 6.8 Hz).

^{31}P NMR (162 MHz, Chloroform-*d*) δ 30.7.

HRMS (ESI) m/z: $[M + H]^+$ Calcd for $C_{20}H_{24}N_2O_2P^+$ 355.1570, Found: 355.1572.

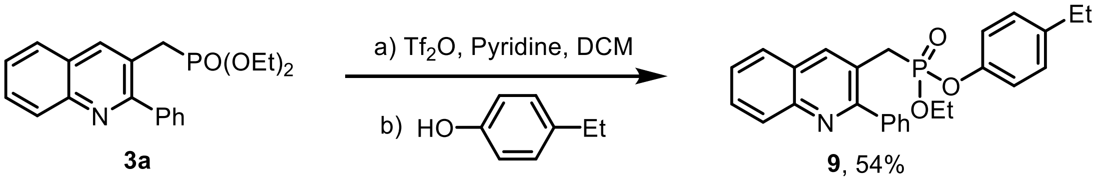

To a stirring solution of compound **3a** (35.6 mg, 0.1 mmol) in CH_2Cl_2 (1 mL) was added Tf_2O (84.6 mg, 0.3 mmol). After stirring for 10 min at room temperature, pyridine (31.6 mg, 0.4 mmol) was added, and the mixture was stirred for an additional 10 min. Subsequently, 4-ethylphenol was added, and the reaction was allowed to proceed for 15 min. Thereafter, Na_2CO_3 (35.0 mg, 0.33 mmol) and MeOH (1 mL) were added sequentially, and the mixture was heated at 50 °C for 3 h. After completion of the

reaction, the crude product was purified by column chromatography on silica gel (PE/EtOAc, v/v = 3:1) to afford product **9**.

ethyl (4-ethylphenyl) ((2-phenylquinolin-3-yl)methyl)phosphonate (9)

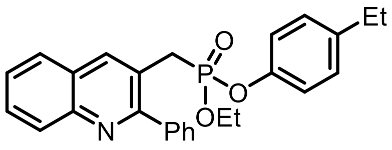

23.6 mg, yellow oil, 54% yield.

^{1}H NMR (400 MHz, Chloroform-*d*) δ 8.45 (d, *J* = 3.5 Hz, 1H), 8.14 (d, *J* = 8.5 Hz, 1H), 7.84 (d, *J* = 8.2 Hz, 1H), 7.71 (t, *J* = 7.6 Hz, 1H), 7.64 – 7.52 (m, 3H), 7.47 (q, *J* = 7.2, 6.7 Hz, 3H), 7.07 (d, *J* = 8.2 Hz, 2H), 6.97 – 6.90 (m, 2H), 4.10 – 4.01 (m, 2H), 3.53 (d, *J* = 22.2 Hz, 2H), 2.58 (q, *J* = 7.6 Hz, 2H), 1.22 – 1.14 (m, 6H).

^{13}C NMR (101 MHz, Chloroform-*d*) δ 160.4 (d, *J* = 7.6 Hz), 148.4 (d, *J* = 8.8 Hz), 146.9 (d, *J* = 2.5 Hz), 141.0 (d, *J* = 1.4 Hz), 140.1, 138.2 (d, *J* = 6.2 Hz), 129.8, 129.4, 129.3, 129.1, 128.6, 128.5, 127.4 (d, *J* = 1.5 Hz), 127.3 (d, *J* = 3.2 Hz), 126.9, 123.1 (d, *J* = 8.7 Hz), 120.2 (d, *J* = 4.3 Hz), 63.2 (d, *J* = 7.1 Hz), 30.0 (d, *J* = 139.8 Hz), 28.2, 16.4 (d, *J* = 5.9 Hz), 15.7.

^{31}P NMR (162 MHz, Chloroform-*d*) δ 22.7.

HRMS (ESI) m/z: $[M + H]^+$ Calcd for $C_{26}H_{27}NO_3P^+$ 432.1723, Found: 432.1729.

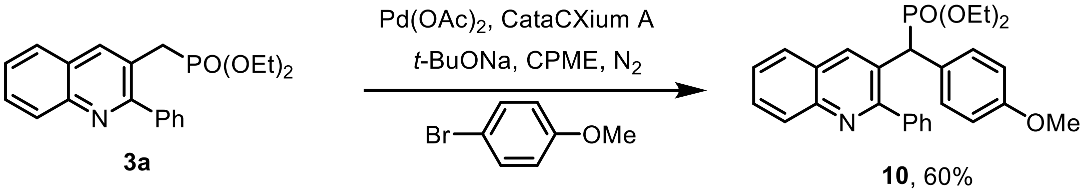

3a **10**, 60%

Prepared according to the reported method.[3] $Pd(OAc)_2$ (2.3 mg, 0.01 mmol) and CataCXium A (7.2 mg, 0.02 mmol) were dissolved in methoxycyclopentane (CPME 1 mL) and stirred for 1 h at room temperature under N_2. *t*-BuONa (28.8 mg, 0.3 mmol), **3a** (35.5 mg, 0.1 mmol), and 1-bromo-4-methoxybenzene (37.4 mg, 0.2 mmol) were added, and the mixture was heated at 80 °C for 12 h. The mixture was extracted with EtOAc (3 × 5 mL), dried over anhydrous Na_2SO_4, and concentrated. The residue was purified by column chromatography (PE/EtOAc, v/v =1:1) afforded the product **10**.

diethyl ((4-methoxyphenyl)(2-phenylquinolin-3-yl)methyl)phosphonate (10)

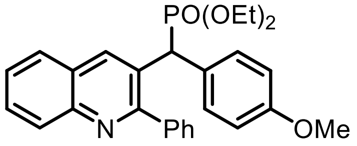

27.8 mg, white solid, 60% yield. M.p = 120 – 121 ºC.

^{1}H NMR (400 MHz, Chloroform-*d*) δ 9.03 (d, *J* = 2.3 Hz, 1H), 8.10 (d, *J* = 8.4 Hz, 1H), 7.97 (d, *J* = 8.2 Hz, 1H), 7.71 (t, *J* = 7.7 Hz, 1H), 7.57 (t, *J* = 7.5 Hz, 1H), 7.49 – 7.42 (m, 3H), 7.38 – 7.35 (m, 2H), 7.20 – 7.13 (m, 2H), 6.78 (d, *J* = 8.4 Hz, 2H), 4.69 (d, *J* = 25.6 Hz, 1H), 4.01 – 3.94 (m, 1H), 3.94 – 3.82 (m, 2H), 3.75 (s, 3H), 3.73 – 3.62 (m, 1H), 1.11 (t, *J* = 7.1 Hz, 3H), 1.06 (t, *J* = 7.1 Hz, 3H).

^{13}C NMR (101 MHz, Chloroform-*d*) δ 161.2 (d, *J* = 12.1 Hz), 159.2 (d, *J* = 2.5 Hz), 146.9 (d, *J* = 1.4 Hz), 140.6, 137.5 (d, *J* = 5.7 Hz), 131.0, 130.9, 130.2, 129.6, 129.59, 129.3, 128.8, 128.4, 128.1 (d, *J* = 6.1 Hz), 127.7 (d, *J* = 1.5 Hz), 127.1, 114.4 (d, *J* = 2.0 Hz), 63.5 (d, *J* = 7.1 Hz), 62.9 (d, *J* = 7.3 Hz), 55.6, 45.7 (d, *J* = 140.1 Hz), 16.7 (d, *J* = 5.9 Hz), 16.7 (d, *J* = 5.7 Hz).

^{31}P NMR (162 MHz, Chloroform-*d*) δ 25.4.

HRMS (ESI) m/z: $[M + H]^+$ Calcd for $C_{27}H_{29}NO_4P^+$ 462.1829, Found: 462.1834.

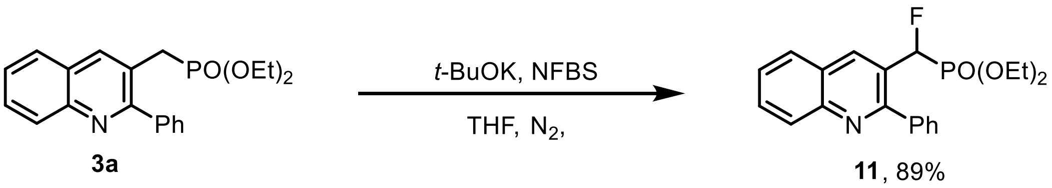

Prepared according to the reported method[4]. To a solution of **3a** (35.6 mg, 0.1 mmol) in THF (1 mL) was added *t*-BuOK (22.4 mg, 0.2 mmol), and the mixture was stirred at -40 °C for 0.5 h under N_2. Subsequently, *N*-fluorobis(phenylsulfonyl)amine (NFBS, 78.8 mg, 0.25 mmol) was added. After stirring for an additional 15 min, the reaction mixture was quenched with water, and extracted with EtOAc (3 × 5 mL). The combined organic layers were dried over anhydrous Na_2SO_4 and concentrated in vacuo. The residue was purified by column chromatography on silica gel (PE/EtOAc, v/v = 4:1) to afford product **11**.

diethyl (fluoro(2-phenylquinolin-3-yl)methyl)phosphonate (11)

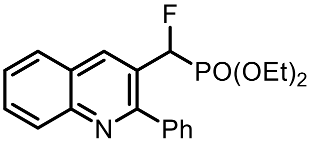

34.9 mg, yellow oil, 89% yield.

^{1}H NMR (400 MHz, Chloroform-*d*) δ 8.82 (t, *J* = 1.9 Hz, 1H), 8.16 (d, *J* = 8.5 Hz, 1H), 7.95 (d, *J* = 8.2 Hz, 1H), 7.78 (t, *J* = 7.7 Hz, 1H), 7.71 – 7.64 (m, 2H), 7.59 (t, *J* = 7.5 Hz, 1H), 7.54 – 7.46 (m, 3H), 5.98 (dd, *J* = 44.3, 7.9 Hz, 1H), 4.27 – 4.18 (m, 2H), 4.13 – 3.93 (m, 2H), 1.35 (t, *J* = 7.1 Hz, 3H), 1.20 (t, *J* = 7.1 Hz, 3H).

^{13}C NMR (101 MHz, Chloroform-*d*) δ 159.2 (dd, *J* = 8.8, 3.2 Hz), 148.1 (t, *J* = 2.2 Hz), 139.2, 138.6 (t, *J* = 4.8 Hz), 131.1, 129.5, 128.9, 128.7, 128.4, 127.2, 126.9 (d, *J* = 2.1 Hz), 124.9 (d, *J* = 17.6 Hz), 85.8 (dd, *J* = 180.4, 173.7 Hz), 64.0 (d, *J* = 6.7 Hz), 63.4 (d, *J* = 6.8 Hz), 16.6 (d, *J* = 5.6 Hz), 16.4 (d, *J* = 5.7 Hz).

^{19}F NMR (377 MHz, Chloroform-*d*) δ -188.1 (d, *J* = 93.2 Hz).

^{31}P NMR (162 MHz, Chloroform-*d*) δ 15.4 (d, *J* = 93.3 Hz).

HRMS (ESI) m/z: $[M + H]^+$ Calcd for $C_{20}H_{22}FNO_3P^+$ 374.1316, Found: 374.1313.

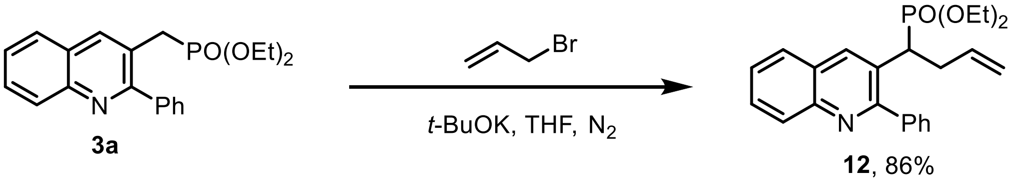

To a solution of **3a** (35.6 mg, 0.1 mmol) in THF was added *t*-BuOK (22.4 mg, 0.2 mmol) at -40 °C. After stirring for 30 min under N_2, 3-bromoprop-1-ene (24.2 mg, 0.2 mmol) was added. The mixture was stirred for a further 15 min, then extracted with EtOAc three times. The organic layer was dried over Na_2SO_4 and concentrated. Purification by column chromatography (PE/EtOAc, v/v = 1:1) afforded the product **12**.

diethyl (1-(2-phenylquinolin-3-yl)but-3-en-1-yl)phosphonate (12)

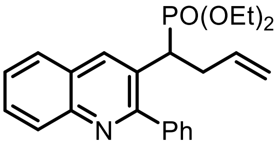

34.2 mg, colorless oil, 86% yield.

^{1}**H NMR** (400 MHz, Chloroform-*d*) δ 8.49 (d, *J* = 3.2 Hz, 1H), 8.12 (d, *J* = 8.5 Hz, 1H), 7.88 (d, *J* = 8.2 Hz, 1H), 7.73 – 7.67 (m, 1H), 7.58 – 7.51 (m, 3H), 7.49 – 7.40 (m, 3H), 5.54 – 5.44 (m, 1H), 4.90 – 4.82 (m, 2H), 4.08 – 3.99 (m, 2H), 3.96 – 3.86 (m, 1H), 3.85 – 3.75 (m, 1H), 3.60 (ddd, *J* = 22.4, 10.9, 4.3 Hz, 1H), 2.92 – 2.61 (m, 2H), 1.28 (t, *J* = 7.1 Hz, 3H), 1.09 (t, *J* = 7.1 Hz, 3H).

^{13}**C NMR** (101 MHz, Chloroform-*d*) δ 161.2 (d, *J* = 8.3 Hz), 146.6 (d, *J* = 2.1 Hz), 140.3, 136.5 (d, *J* = 5.4 Hz), 134.5, 134.4, 129.8 (d, *J* = 1.4 Hz), 129.4 (d, *J* = 1.2 Hz), 129.3, 128.5, 128.3, 127.7, 127.4 (d, *J* = 2.8 Hz), 126.7, 117.8, 62.7 (d, *J* = 7.2 Hz), 62.2 (d, *J* = 7.2 Hz), 39.0 (d, *J* = 139.1 Hz), 36.0 (d, *J* = 2.7 Hz), 16.5 (d, *J* = 6.0 Hz), 16.4 (d, *J* = 5.7 Hz).

^{31}**P NMR** (162 MHz, Chloroform-*d*) δ 28.1.

HRMS (ESI) m/z: $[M + H]^+$ Calcd for $C_{23}H_{27}NO_3P^+$ 396.1723, Found: 396.1721.

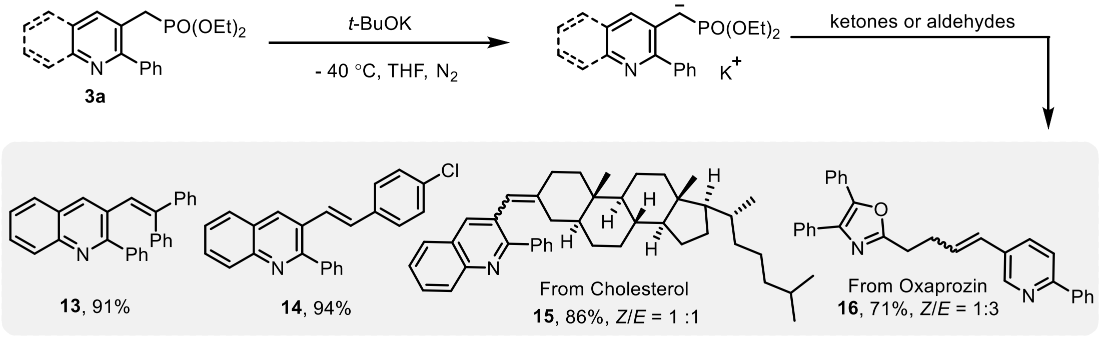

General procedure: *t*-BuOK (22.4 mg, 0.2 mmol) was added to a solution of **3a** (42.6 mg, 0.12 mmol) in THF (1 mL) at -40 °C. After stirring for 1 h under N_2, the corresponding ketone or aldehyde (0.1 mmol) was added. The mixture was warmed to room temperature and stirred for 3 h. Water was added, and the mixture was extracted with EtOAc. The combined organic layers were concentrated and purified by column chromatography (PE/EtOAc, 40:1 to 10:1) to give the *meta*-alkenyl azaarene.

3-(2,2-diphenylvinyl)-2-phenylquinoline (13)

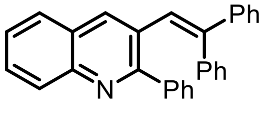

35 mg, white solid, 91% yield. M.p = 190 – 192 ºC.

^{1}**H NMR** (400 MHz, Chloroform-*d*) δ 8.05 (d, *J* = 8.5 Hz, 1H), 7.77 – 7.72 (m, 2H),

7.68 (s, 1H), 7.59 (t, J = 6.92 Hz, 1H), 7.44 – 7.33 (m, 5H), 7.24 – 7.22 (m, 8H), 7.16 – 7.14 (m, 2H), 6.91 (s, 1H).

^{13}C NMR (101 MHz, Chloroform-*d*) δ 159.5, 146.8, 144.1, 142.9, 140.4, 139.7, 137.9, 130.7, 130.0, 129.7, 129.5, 129.3, 128.7, 128.6, 128.4, 128.37, 128.1, 127.9, 127.6, 127.59, 126.9, 126.4, 125.9.

HRMS (ESI) m/z: $[M + H]^+$ Calcd for $C_{29}H_{22}N^+$ 384.1747, Found: 384.1745.

(*E*)-3-(4-chlorostyryl)-2-phenylquinoline (14)

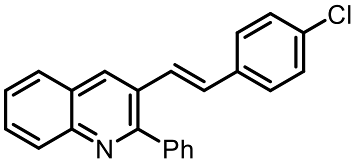

32.2 mg, white solid, 94% yield. M.p = 165 – 166 ºC.

^{1}H NMR (400 MHz, Chloroform-*d*) δ 8.42 (s, 1H), 8.16 (d, J = 8.5 Hz, 1H), 7.87 (d, J = 8.1 Hz, 1H), 7.73 – 7.69 (m, 3H), 7.57 – 7.46 (m, 4H), 7.33 (q, J = 8.4 Hz, 4H), 7.15 (q, J = 16.2 Hz, 2H).

^{13}C NMR (101 MHz, Chloroform-*d*) δ 158.9, 147.5, 140.0, 135.7, 133.7, 133.1, 129.9, 129.87, 129.8, 129.76, 129.5, 129.1, 128.8, 128.5, 128.0, 127.6, 127.5, 127.3, 126.9.

HRMS (ESI) m/z: $[M + H]^+$ Calcd for $C_{23}H_{17}ClN^+$ 342.1044, Found: 342.1047.

3-(((5*S*,8*R*,9*S*,10*S*,13*R*,14*S*,17*R*)-10,13-dimethyl-17-((*R*)-6-methylheptan-2-yl)hexadecahydro-3H-cyclopenta[*a*]phenanthren-3-ylidene)methyl)-2-phenylquinoline (15)

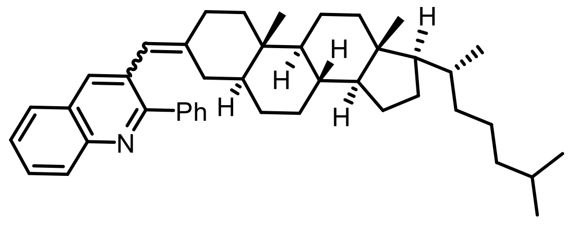

50.6 mg, a mixture of isomers (Z/E = 1:1, determined by ^{1}H NMR analysis of the mixture), white solid, 86% yield. M.p = 133 – 136 ºC.

^{1}H NMR (400 MHz, Chloroform-*d*) δ 8.16 (s, 1H), 8.14 (s, 1H), 7.97 (d, J = 3.4 Hz, 2H), 7.80 (d, J = 8.2 Hz, 2H), 7.77 – 7.70 (m, 4H), 7.67 (t, J = 7.6 Hz, 2H), 7.53 – 7.40 (m, 8H), 6.15 (s, 1H), 6.12 (s, 1H), 2.65 (d, J = 14.3 Hz, 1H), 2.40 – 2.28 (m, 2H), 2.25

– 2.14 (m, 2H), 2.11 – 1.43 (m, 19H), 1.40 – 0.98 (m, 38H), 0.92 – 0.85 (m, 24H), 0.67 (d, *J* = 2.8 Hz, 6H).

^{13}C NMR (101 MHz, Chloroform-*d*) δ 159.4, 159.37, 146.9, 144.1, 144.0, 140.8, 140.7, 137.1, 137.06, 130.36, 130.3, 130.0, 129.99, 129.4, 129.1, 128.4, 128.36, 128.1, 127.3, 127.26, 127.23, 127.21, 126.5, 120.4, 120.1, 56.6, 56.59, 56.4, 54.5, 54.49, 48.1, 47.1, 42.7, 40.2, 39.9, 39.8, 39.6, 39.3, 36.5, 36.4, 36.3, 35.9, 35.6, 35.5, 33.0, 32.5, 32.1, 32.0, 29.1, 28.9, 28.4, 28.1, 25.6, 24.34, 24.3, 24.0, 23.0, 22.7, 21.3, 21.2, 18.81, 18.8, 12.2, 12.0, 11.9.

HRMS (ESI) m/z: $[M + H]^+$ Calcd for $C_{43}H_{58}N^+$ 588.4564, Found: 588.4569.

(*Z*)-4,5-diphenyl-2-(4-(6-phenylpyridin-3-yl)but-3-en-1-yl)oxazole (16-*Z*)

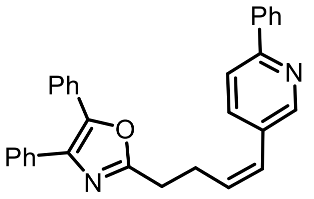

8.0 mg, yellow oil, 18% yield.

^{1}H NMR (400 MHz, Chloroform-*d*) δ 8.63 (d, *J* = 2.0 Hz, 1H), 8.03 – 7.95 (m, 2H), 7.71 – 7.66 (m, 2H), 7.66 – 7.61 (m, 2H), 7.56 – 7.52 (m, 2H), 7.50 – 7.46 (m, 2H), 7.44 – 7.29 (m, 7H), 6.51 (d, *J* = 11.8 Hz, 1H), 5.92 (dt, *J* = 11.7, 7.2 Hz, 1H), 3.07 – 3.00 (m, 2H), 2.95 (dd, *J* = 8.1, 6.4 Hz, 2H).

^{13}C NMR (101 MHz, Chloroform-*d*) δ 162.5, 155.6, 149.8, 145.5, 139.1, 136.7, 135.3, 132.7, 132.6, 131.4, 129.1, 129.01, 128.9, 128.8, 128.7, 128.5, 128.2, 128.1, 126.9, 126.6, 120.1, 28.4, 26.4.

HRMS (ESI) m/z: $[M + H]^+$ Calcd for $C_{30}H_{25}N_2O^+$ 429.1961, Found: 429.1958.

(*E*)-4,5-diphenyl-2-(4-(6-phenylpyridin-3-yl)but-3-en-1-yl)oxazole (16-*E*)

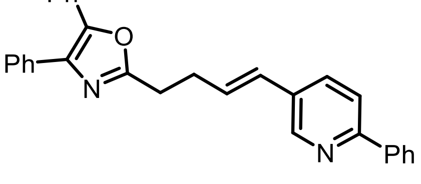

22.8 mg, white solid, 53% yield. M.p = 116 – 118 ºC.

^{1}H NMR (400 MHz, Chloroform-*d*) δ 8.64 (d, *J* = 2.3 Hz, 1H), 7.99 (d, *J* = 7.6 Hz, 2H),

7.76 (dd, J = 8.3, 2.3 Hz, 1H), 7.67 (dd, J = 9.7, 7.7 Hz, 3H), 7.62 – 7.56 (m, 2H), 7.47 (t, J = 7.5 Hz, 2H), 7.43 – 7.30 (m, 7H), 6.55 (d, J = 16.0 Hz, 1H), 6.44 (dt, J = 15.7, 6.6 Hz, 1H), 3.08 (t, J = 7.6 Hz, 2H), 2.84 (q, J = 7.2 Hz, 2H).

^{13}C NMR (101 MHz, Chloroform-*d*) δ 162.7, 156.0, 148.1, 145.4, 139.2, 135.2, 133.5, 132.6, 131.5, 130.7, 129.1, 129.0, 128.9, 128.8, 128.7, 128.6, 128.2, 128.0, 127.8, 126.8, 126.5, 120.4, 30.7, 28.2.

HRMS (ESI) m/z: $[M + H]^+$ Calcd for $C_{30}H_{25}N_2O^+$ 429.1961, Found: 429.1962.

6. Mechanistic studies

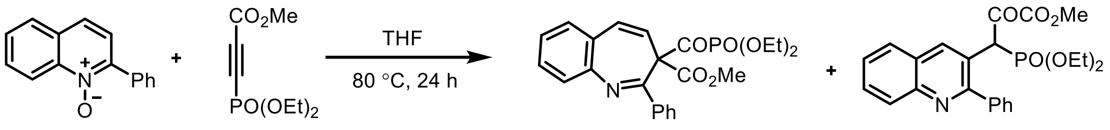

1a **2a** **4a**, 20% **3a'**

A mixture of *N*-oxide **1a** (22.1 mg, 0.1 mmol), alkyne **2a** (33.0 mg, 0.15 mmol) was dissolved in THF (1 mL) and stirred at 80 ºC for 24 h. Upon cooling to room temperature, the volatiles were removed under reduced pressure. Purification of the crude residue by column chromatography on silica gel (eluent: PE/EtOAc = 2:1) yielded product **4a and 3a'**. ^{1}H and ^{31}P NMR analysis revealed **3a'** exists as a mixture of ketone and enolate tautomers with a ratio of 1:0.6.

methyl 3-((diethoxyphosphoryl)carbonyl)-2-phenyl-3H-benzo[*b*]azepine-3-carboxylate (4a)

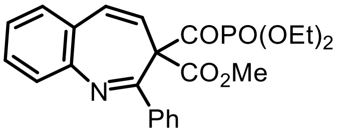

8.8 mg, yellow oil, 20% yield.

^{1}H NMR (400 MHz, Chloroform-*d*) δ 7.53 (d, *J* = 8.1 Hz, 1H), 7.49 – 7.29 (m, 7H), 7.18 (t, *J* = 7.5 Hz, 1H), 7.12 (d, *J* = 10.1 Hz, 1H), 6.57 (d, *J* = 10.1 Hz, 1H), 4.05 – 3.86 (m, 2H), 3.84 – 3.74 (m, 1H), 3.58 – 3.48 (m, 1H), 3.48 (s, 3H), 1.22 (t, *J* = 7.1 Hz, 3H), 1.11 (t, *J* = 7.1 Hz, 3H).

^{13}C NMR (101 MHz, Chloroform-*d*) δ 195.5 (d, *J* = 176.6 Hz), 167.6 (d, *J* = 3.0 Hz), 153.2 (d, *J* = 5.9 Hz), 146.0, 139.7 (d, *J* = 2.9 Hz), 131.7, 130.3, 129.1, 128.4, 128.1, 128.0, 127.9, 125.1, 124.8, 121.2 (d, *J* = 2.3 Hz), 71.9 (d, *J* = 56.6 Hz), 64.3 (d, *J* = 8.0 Hz), 63.8 (d, *J* = 6.6 Hz), 53.3, 16.3 (d, *J* = 6.0 Hz), 16.2 (d, *J* = 6.1 Hz).

^{31}P NMR (162 MHz, Chloroform-*d*) δ -5.3.

HRMS (ESI) m/z: $[M + H]^+$ Calcd for $C_{23}H_{25}NO_6P^+$ 442.1414; Found 442.1424.

3a', a tautomeric mixture of ketone and enolate with a ratio of 1:0.6, 2 mg, yellow oil.

^{1}H NMR (400 MHz, Chloroform-*d*) δ 11.85 (s, 1H, enolate), 8.68 (d, *J* = 3.0 Hz, 1H, ketone), 8.19 – 8.08 (m, 1H, ketone, 1H enolate), 8.00 – 7.90 (m, 1H, ketone, 1H

enolate), 7.81 – 7.69 (m, 2H, ketone, 2H enolate), 7.62 – 7.34 (m, 5H, ketone, 6H enolate), 5.92 (d, J = 25.9 Hz, 1H, ketone), 4.13 – 3.81 (m, 4H, ketone, 4H enolate), 3.80 (s, 3H, ketone), 3.53 (s, 3H, enolate), 1.28 – 1.24 (m, 6H, ketone), 1.19 (t, J = 7.1 Hz, 6H, enolate).

^{31}P NMR (162 MHz, Chloroform-*d*) δ 20.7 (s, enonate), 16.7 (s, ketone)

HRMS (ESI) m/z: $[M + H]^+$ Calcd for $C_{23}H_{25}NO_6P^+$ 442.1414, Found: 442.1413.

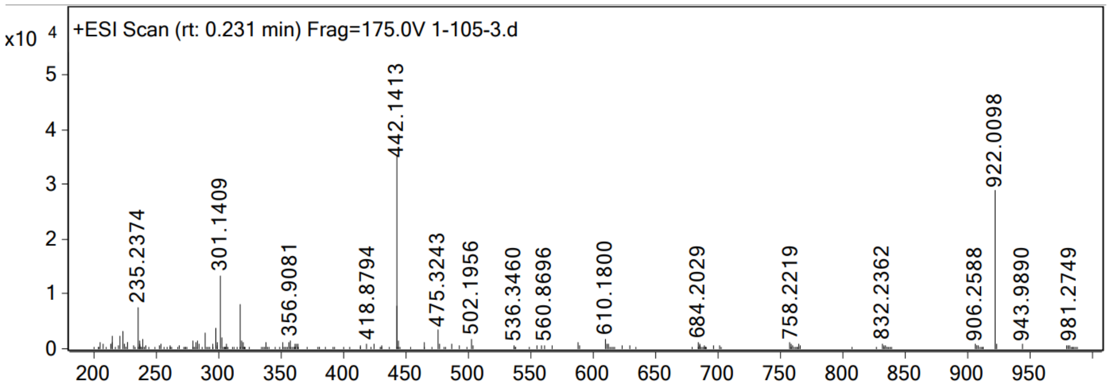

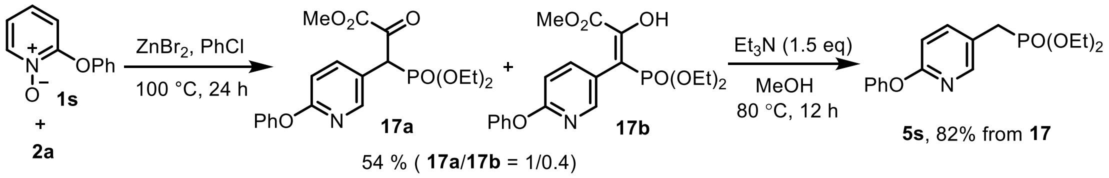

A mixture of *N*-oxide **1s** (0.1 mmol, 18.7 mg), alkyne **2a** (0.2 mmol, 44.0 mg) and $ZnBr_2$ (0.02 mmol, 4.51 mg) was dissolved in PhCl (1 mL), then the mixture was stirred at 100 ºC until **1s** was completely consumed monitored by thin layer chromatography (TLC). After 24 h, the reaction was cooled to room temperature and the solvent was removed under reduced pressure. The residue was purified by column chromatography on silica gel using MeOH/DCM (v/v = 1:50) as eluent to afford **17** as yellow oil (22.0 mg, 54% yield).

An oven-dried vial was charged with the **17** (20.4 mg, 0.05 mmol), Et_3N (7.6 mg, 0.08 mmol) and MeOH (0.5 mL), then the solution was reacted at 80 ºC for 12 h. After the reaction was completed, the solvent was removed and the crude product was purified by column chromatography (PE/EtOAc = 1:4) to yield **5s** as yellow oil (13.3 mg, 82% yield from **17**).

17, a tautomeric mixture of ketone **17a** and enolate **17b** with a ratio of 1:0.4, **^{1}H NMR**

(400 MHz, Chloroform-*d*) δ 11.86 (s, 1H, **17b**), 8.18 (t, *J* = 2.6 Hz, 1H, **17a**), 7.99 – 7.94 (m, 1H, **17a**, 1H, **17b**), 7.53 – 7.48 (m, 1H, **17b**), 7.43 – 7.35 (m, 2H, **17a**, 2H, **17b**), 7.23 – 7.11 (m, 3H, **17a**, 3H, **17b**), 6.92 (d, *J* = 8.6 Hz, 1H, **17a**), 6.86 (d, *J* = 8.5 Hz, 1H, **17b**), 5.46 (d, *J* = 26.1 Hz, 1H, **17a**), 4.14 – 4.05 (m, 4H, **17a**, 4H, **17b**), 3.90 (s, 3H, **17a**), 3.65 (s, 3H, **17b**), 1.29 – 1.22 (m, 6H, **17a**, 6H, **17b**).

^{31}P NMR (162 MHz, Chloroform-*d*) δ 21.7 (s, **17b**), 16.1 (s, **17a**).

HRMS (ESI) m/z: $[M + H]^+$ Calcd for $C_{19}H_{23}NO_7P^+$ 408.1207, Found: 408.1218.

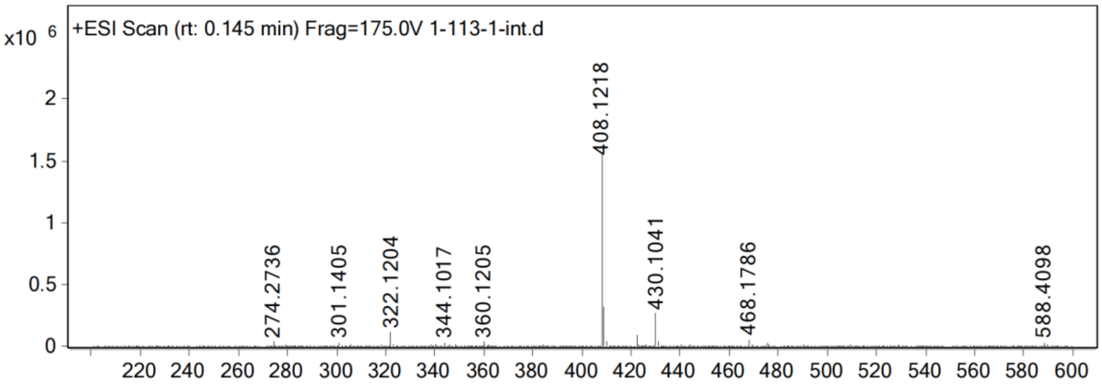

Detection of byproduct MeO_2CCO_2Me by GC-MS analysis of the crude reaction mixture under standard conditions

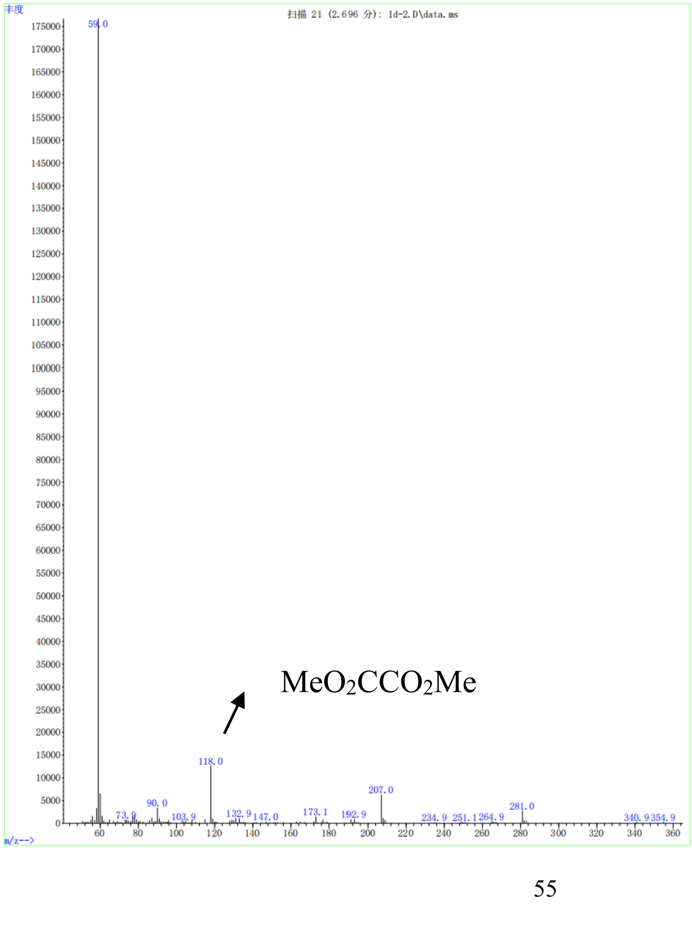

7. Density functional theory (DFT) studies

Computational Methods

All density functional theory (DFT) calculations were performed using the Gaussian 16 software package.[5] For the geometry optimization, the B3LYP functional[6,7] with Grimme's third generation empirical dispersion correction including Becke-Johanson damping was used,[8,9] which is denoted as B3LYP-D3BJ. The def2svp[10] basis sets was used for all geometry optimizations performed. Hessian calculation were performed to confirm the stationary points; minima with no imaginary frequency. Solvent effects were considered based on SMD model in 1,4-dioxane for the geometry optimization and single-point energy calculation.[11] The Hirshfeld charge was used for computing charges on the atoms with Multiwfn 3.8 program.[12-14]

Cartesian coordinates (Å) of optimized structures

2a

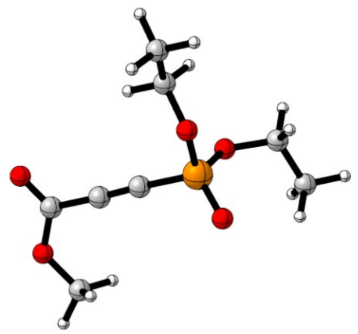

C	-1.87860200	0.20003500	-0.14488300
C	-0.69814600	0.01642300	0.07803800
P	0.99105600	-0.25442000	0.52888000
O	1.19311200	-1.10482000	1.72410600
C	-3.28098300	0.47218700	-0.44795500
O	-3.62202900	1.47205800	-1.02708200
O	-4.16818100	-0.44346500	-0.04974800
C	-3.74187100	-1.62442800	0.64171900
O	1.55323000	-0.79588800	-0.88034400

C	2.81575000	-2.88111000	-0.66156600
C	2.87099800	-1.39686300	-0.95240200
O	1.60140000	1.21936500	0.72038800
C	2.16352500	3.49372600	0.22073000
C	1.56052900	2.21922200	-0.32084200
H	-4.65756400	-2.18420300	0.86912700
H	-3.08444200	-2.24046700	0.00936600
H	-3.22369900	-1.37369700	1.57979500
H	3.81769100	-3.32286000	-0.78546800
H	2.47767000	-3.05988700	0.36940500
H	2.12580400	-3.38775700	-1.35430900
H	3.21885700	-1.19709800	-1.97642500
H	3.55290700	-0.87759500	-0.25874100
H	2.14484800	4.27387700	-0.55651600
H	1.59478400	3.85602400	1.09079700
H	3.20823000	3.33407400	0.52955800
H	2.11873300	1.84725700	-1.19535800
H	0.51289000	2.37547500	-0.63003300

2a-ZnBr$_2$ (18)

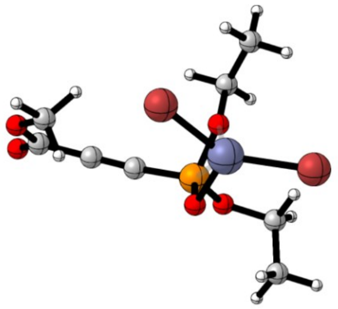

C	-3.32862500	1.07425800	-0.16778700
C	-2.16106500	1.40761800	-0.13420000
P	-0.42238000	1.43751200	-0.13928600

O	0.18395300	0.62779900	-1.26260100
C	-4.71999100	0.61861600	-0.22217900
O	-5.64516700	1.38146800	-0.12237200
O	-4.88240100	-0.69196300	-0.38911500
C	-3.74592100	-1.57274400	-0.48748600
O	-0.03743900	2.96929200	-0.07279900
C	1.82393900	3.58296400	-1.55617400
C	1.37882700	3.36638100	-0.13022000
O	0.06251000	0.70147100	1.19581900
C	0.45300800	0.06182900	3.45790100
C	-0.24070900	1.06443800	2.56913500
H	-4.16062000	-2.57720400	-0.63178500
H	-3.11108200	-1.31067700	-1.34577900
H	-3.14690400	-1.55312600	0.43381600
H	2.85948500	3.95850800	-1.55477800
H	1.80279200	2.64077000	-2.12211300
H	1.18571900	4.32372300	-2.06151300
H	1.42220000	4.28756200	0.46571900
H	1.98565300	2.59388700	0.36766700
H	0.25187500	0.29767700	4.51415600
H	0.09020000	-0.95547000	3.24716900
H	1.54056600	0.08399500	3.29097500
H	0.11437200	2.09231800	2.74198000
H	-1.33481300	1.04291600	2.69752200
Br	3.43175300	-0.24269500	-0.11304500
Br	-0.13823000	-2.77995300	-0.15099800
Zn	1.24515200	-0.94065200	-0.44925400

8. Substrates preparation and characterization.

The *N*-oxide compounds were prepared according to previous literature reported by our group.[15-17] Alkynes **2** were prepared according to literature procedures with some modifications.[18]

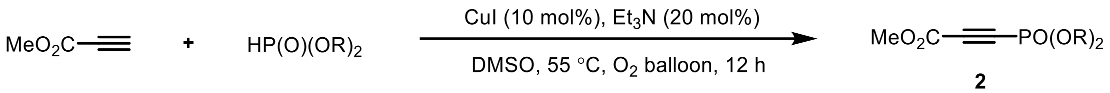

The corresponding phosphite ester (1 mmol, 139 mg), CuI (19 mg, 0.1 mmol), Et_3N (21 mg, 0.2 mmol) were dissolved in anhydrous DMSO (6 mL), then the mixture was purged with O_2 balloon for 3 min. Subsequently, a solution of methyl propiolate (1.2 mmol, 101 mg) in anhydrous DMSO (3 mL) was added dropwise at 55 ºC under an oxygen atmosphere. The resulting system was stirred at 55 ºC for 12 h while maintaining the oxygen atmosphere. Upon completion of the reaction, the mixture was extracted with saturated brine, dried over anhydrous Na_2SO_4, concentrated in vacuo. The residue was purified by column chromatography on silica gel using PE/EtOAc (v/v = 6:1) as eluent to afford alkyne product **2**.

methyl 3-(diethoxyphosphoryl)propiolate (2a)

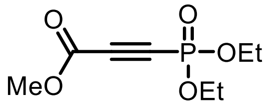

122 mg, yellow oil, 55% yield.

^{1}H NMR (400 MHz, Chloroform-*d*) δ 4.21 – 4.13 (m, 4H), 3.79 (s, 3H), 1.34 (t, *J* = 7.1 Hz, 6H).

^{13}C NMR (101 MHz, Chloroform-*d*) δ 152.1 (d, *J* = 6.0 Hz), 86.5 (d, *J* = 46.6 Hz), 74.1, 64.3 (d, *J* = 5.7 Hz), 53.6, 16.2 (d, *J* = 6.9 Hz).

^{31}P NMR (162 MHz, Chloroform-*d*) δ -9.8.

HRMS (ESI) m/z: $[M + H]^+$ Calcd for $C_8H_{14}O_5P^+$ 221.0573, Found: 221.0577.

methyl 3-(dibutoxyphosphoryl)propiolate (2b)

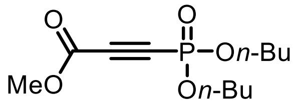

139 mg, yellow oil, 50% yield.

^{1}H NMR (400 MHz, Chloroform-*d*) δ 4.18 – 4.09 (m, 4H), 3.83 (s, 3H), 1.77 – 1.63 (m, 4H), 1.49 – 1.36 (m, 4H), 0.94 (t, *J* = 7.4 Hz, 6H).

^{13}C NMR (101 MHz, Chloroform-*d*) δ 152.1 (d, *J* = 5.9 Hz), 86.5 (d, *J* = 46.4 Hz), 74.0, 67.9 (d, *J* = 6.1 Hz), 53.5, 32.2 (d, *J* = 6.9 Hz), 18.7, 13.6.

^{31}P NMR (162 MHz, Chloroform-*d*) δ -9.4.

HRMS (ESI) m/z: $[M + H]^+$ Calcd for $C_{12}H_{22}O_5P^+$ 277 .1199, Found: 277.1196.

diethyl (3-oxobut-1-yn-1-yl)phosphonate (2c)

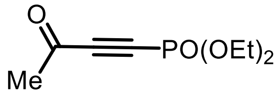

The diethyl (3-oxobut-1-yn-1-yl)phosphonate **2c** was synthesized according to literature procedures[19]. Yellow liquid, **^{1}H NMR** (400 MHz, Chloroform-*d*) δ 4.26 – 4.11 (m, 4H), 2.40 (s, 3H), 1.36 (t, *J* = 7.1 Hz, 6H). **^{13}C NMR** (101 MHz, Chloroform-*d*) δ 182.4 (d, *J* = 4.2 Hz), 92.5 (d, *J* = 43.0 Hz), 77.5 (d, *J* = 277.9 Hz), 64.1 (d, *J* = 5.7 Hz), 32.4, 16.0 (d, *J* = 6.9 Hz). **^{31}P NMR** (162 MHz, Chloroform-*d*) δ -9.1. **HRMS (ESI)** m/z: $[M + H]^+$ Calcd for $C_8H_{14}O_4P^+$ 205.0624, Found: 205.0616.

methyl 3-(phenylsulfonyl)propiolate (2d)

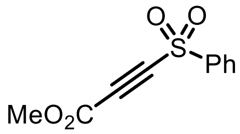

The methyl 3-(phenylsulfonyl)propiolate **2d** was synthesized according to literature procedures[20-21]. Yellow liquid, **^{1}H NMR** (400 MHz, Chloroform-*d*) δ 8.07 – 7.99 (m, 2H), 7.78 – 7.72 (m, 1H), 7.63 (dd, *J* = 8.5, 7.3 Hz, 2H), 3.82 (s, 3H). **^{13}C NMR** (101 MHz, Chloroform-*d*) δ 151.4, 139.7, 135.3, 129.8, 128.1, 79.5, 79.4, 53.9. **HRMS (ESI)** m/z: $[M + H]^+$ Calcd for $C_{10}H_9O_4S^+$ 225.0216, Found: 225.0220.

9. References

[1] H. Wei, H. Chen, J. Chen, I. D. Gridnev, W. Zhang, *Angew. Chem. Int. Ed.* **2023**, *62*, e202214990.

[2] K. Zhang, W. Feng, Z. Mou, L. Zhan, M. Ma, Z. Zhao, X. Liu, X. Chen, Z. Li, *Nat. Commun.* **2024**, *15*, 10338.

[3] S. Montel, L. Raffier, Y. He, and P. J. Walsh, *Org. Lett.* **2014**, *16*, 1446−1449.

[4] S. D. Taylor, A. N. Dinauff, A. N. Thadani, Z. Huang, *Tetrahedron Lett.* **1996**, *37*, 8089-8092.

[5] M. J. Frisch, G. W. Trucks, H. B. Schlegel, G. E. Scuseria, M. A. Robb, J. R. Cheeseman, G. Scalmani, V. Barone, G. A. Petersson, H. Nakatsuji, X. Li, M. Caricato, A. V. Marenich, J. Bloino, B. G. Janesko, R. Gomperts, B. Mennucci, H. P. Hratchian, J. V. Ortiz, A. F. Izmaylov, J. L. Sonnenberg, Ding, F. Williams, F. Lipparini, F. Egidi, J. Goings, B. Peng, A. Petrone, T. Henderson, D. Ranasinghe, V. G. Zakrzewski, J. Gao, N. Rega, G. Zheng, W. Liang, M. Hada, M. Ehara, S18 1 K. Toyota, R. Fukuda, J. Hasegawa, M. Ishida, T. Nakajima, Y. Honda, O. Kitao, H. Nakai, T. Vreven, K. Throssell, Jr. Montgomery, J. E. Peralta, F.O. gliaro, M. J. Bearpark, J. J. Heyd, E. N. Brothers, K. N. Kudin, V. N. Staroverov, T. A. Keith, R. Kobayashi, J. Normand, K. Raghavachari, A. P. Rendell, J. C. Burant, S. S. Iyengar, J. Tomasi, M. Cossi, J. M. Millam, M. Klene, C. Adamo, R. Cammi, J. W. Ochterski, R. L. Martin, K. Morokuma, O. Farkas, J. B. Foresman, D. J. Fox, *Gaussian 16 Revision A.03*, Wallingford, CT, **2016**.

[6] C. Lee, W. Yang, R.G. Parr, *Phys. Rev. B* **1988**, *37*, 785–789.

[7] A. D. Beck, *J. Chem. Phys*. **1993**, *98*, 5648–5652.

[8] S. Grimme, J. Antony, S. Ehrlich, H. Krieg, *J. Chem. Phys.* **2010**, *132*, 154104-154118.

[9] S. Grimme, S. Ehrlich, L. Goerigk, *J. Comput. Chem.* **2011**, *32*, 1456–1465.

[10] F. Weigend, R. Ahlrichs, *Phys. Chem. Chem. Phys*. **2005**, *7*, 3297–3305.

[11] A. V. Marenich, C. J. Cramer, D. G. Truhlar, *J. Phys. Chem. B* **2009**, *113*, 6378–6396.

[12] F. L. Hirshfeld, *Theor. Chim. Acta (Berl.)*, **1977**, *44*, 129.

[13] T. Lu, F. Chen, *J. Comput. Chem*. **2012**, *33*, 580–592.

[14] T. Lu, *J Chem Phys.* **2024**, *161*, 082503.

[15] J. Woo, A. H. Christian, S. A. Burgess, Y. Jiang, U. F. Mansoor, M. D. Levin, *Science*, **2022**, *376*, 527−532.

[16] R. Martinez, D. J. Ramon, M. J. Yus, *Org. Chem*. **2008**, *73*, 9778–9780.

[17] D. Tian, Y.-P. He, L.-S. Yang, Z.-C. Li, H. Wu, *Nat. Chem.* **2025**, *17*, 952–960.

[18] Y. Gao, G. Wang, L. Chen, P. Xu, Y. Zhao, Y. Zhou, L.-B. Han, *J. Am. Chem. Soc.* **2009**, *131*, 7956–7957.

[19] Y. Moglie, E. Mascaro, V. Gutierrez, F. Alonso, G. Radivoy, *J. Org. Chem.* **2016**, *81*, 1813−1818.

[20] S. Okumura, Y. Takeda, K. Kiyokawaa, S. Minakata, *Chem. Commun.* **2013**, *49*, 9266-9268.

[21] P. Sharma, R. R. Singh, S. S. Giri, L.-Y. Chen, M.-J. Cheng, R.-S. Liu, *Org. Lett.* **2019**, *21*, 5475−5479.

10. Crystallographic data

Table 2 Crystallographic data for **3ap**

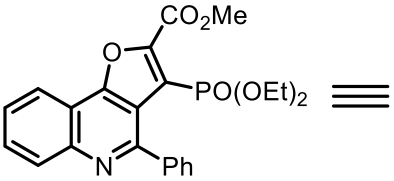

3ap

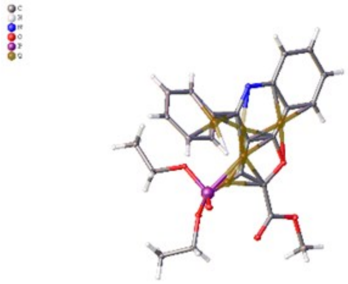

CCDC 2521916

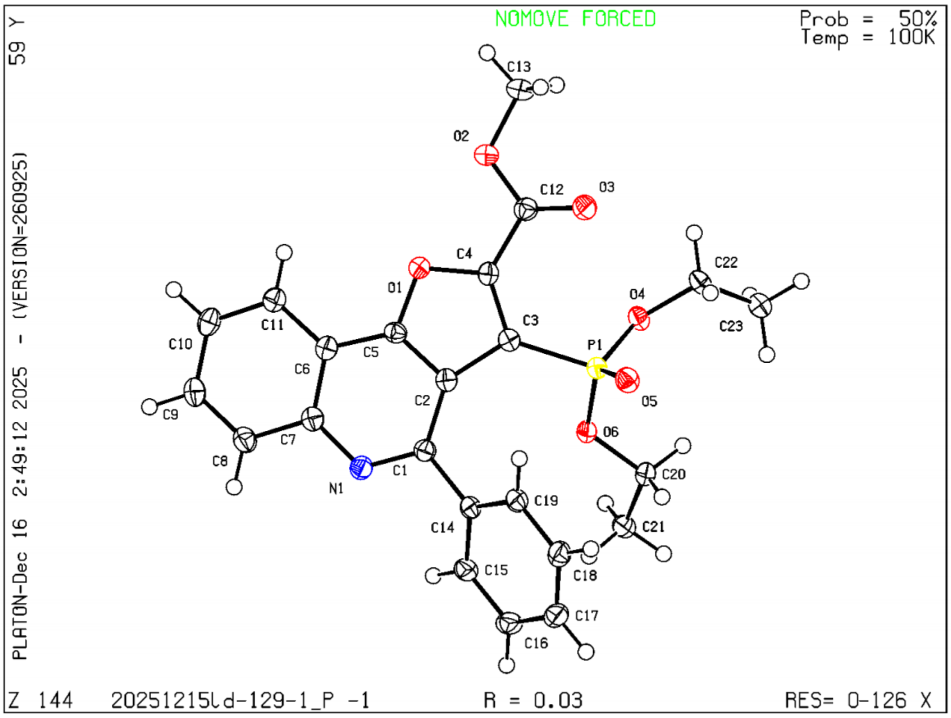

Bond precision:	C-C = 0.0020 Å		Wavelength=1.54184
Cell:	a=7.3493(4)	b=8.8307(5)	c=16.1540(9)
	alpha=76.690(5)	beta=84.882(4)	gamma=86.571(5)
Temperature:	Temperature:		
	Calculated		Reported
Volume	1015.32(10)		1015.32(10)
Space group	P -1		P -1
Hall group	-P 1		-P 1
Moiety formula	C23 H22 N O6 P		C23 H22 N O6 P

Sum formula	C23 H22 N O6 P	C23 H22 N O6 P
Mr	439.39	439.38
Dx ($g{\cdot}cm^{-3}$)	1.437	1.437
Z	2	2
Mu (mm^{-1})	1.568	1.568
F000	460.0	460.0
F000´	462.02	
h,k,lmax	9, 11, 20	9, 11, 20
Nref	4200	3947
Tmin,Tmax	0.778, 0.828	0.922, 1.000
Tmin´	0.778	

Correction method= # Reported T Limits: Tmin=0.922 Tmax=1.000

AbsCorr = MULTI-SCAN

Data completeness= 0.940 Theta(max) = 75.480

R(reflections)= 0.0347(3525) wR2(reflections) = 0.0952(3947)

S = 1.072 Npar= 284

CCDC#2521916 contains the supplementary crystallographic data for this structure. The data can be Cambridge obtained free of charge from the Crystallographic Data Centre via www.ccdc.cam.ac.uk

11. Copies of NMR spectra

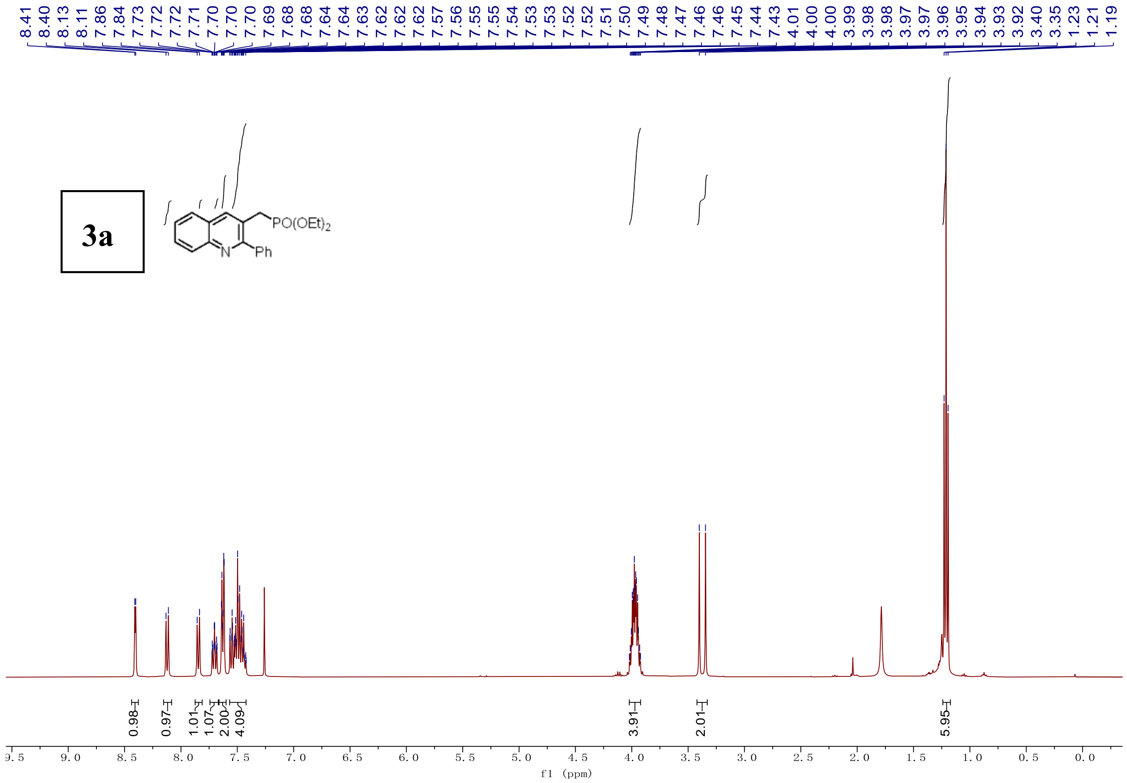

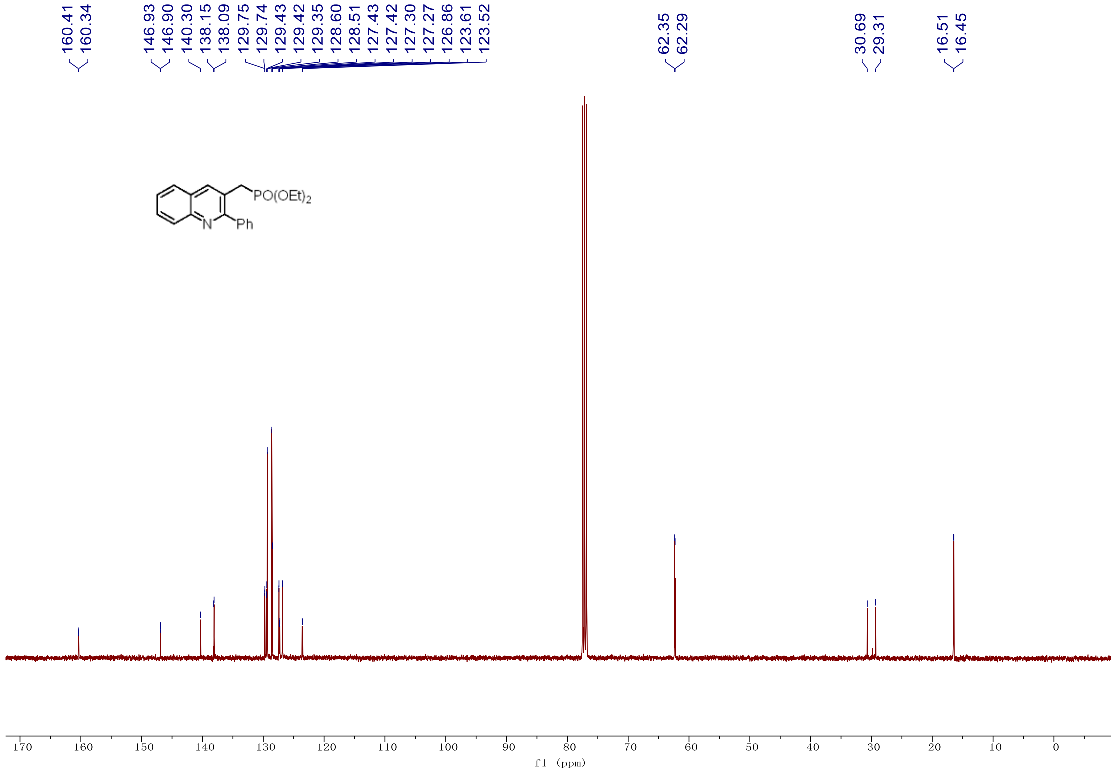

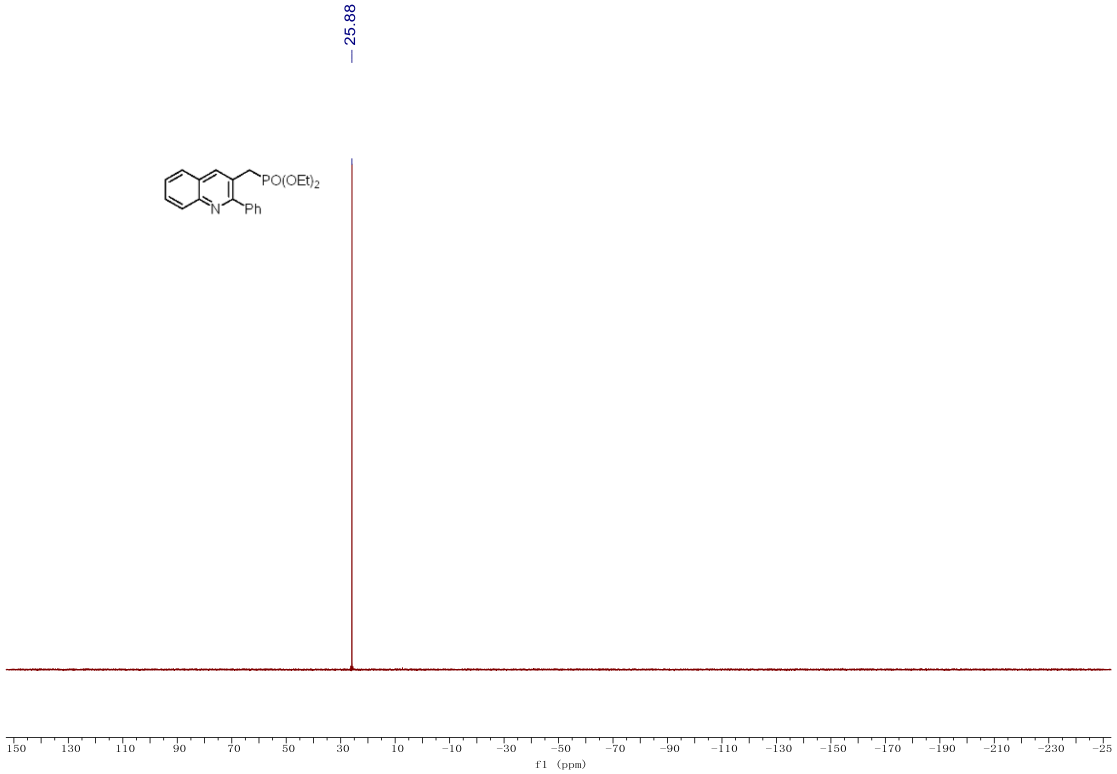
25.88
PO(OEt)2
N
Ph
150 130 110 90 70 50 30 10 −10 −30 −50 −70 −90 −110 −130 −150 −170 −190 −210 −230 −25
f1 (ppm)

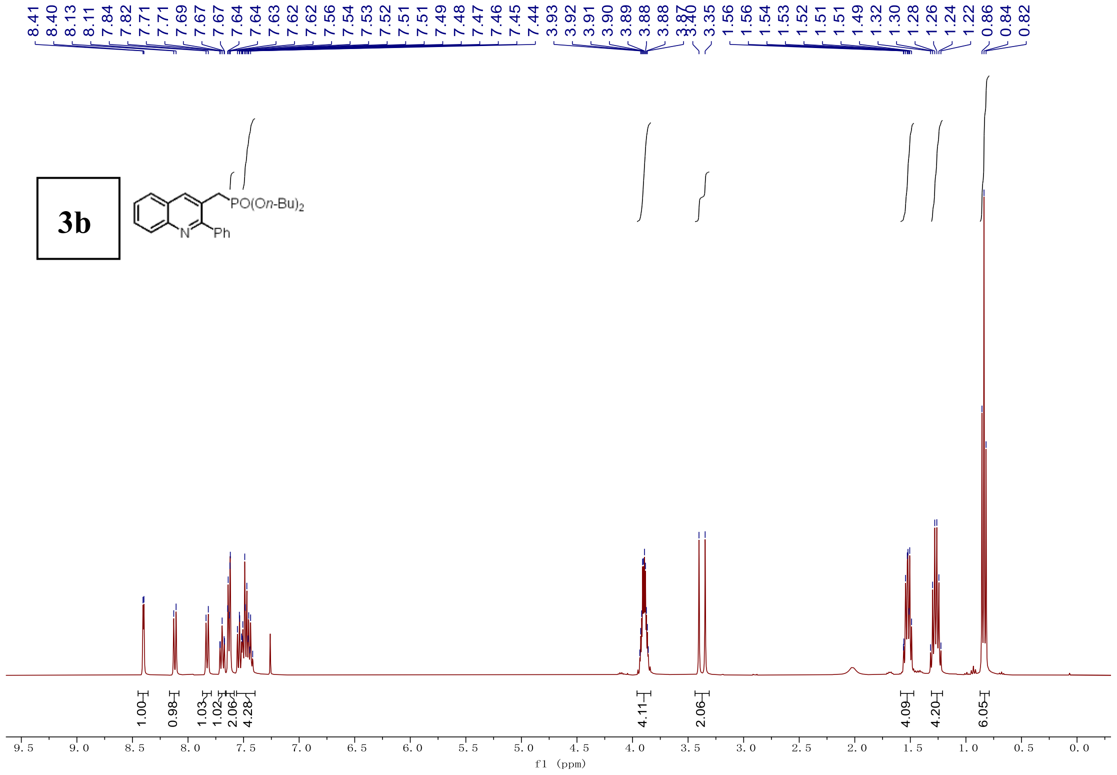
8.41 8.40 8.13 8.11 7.84 7.82 7.71 7.71 7.69 7.67 7.67 7.64 7.64 7.63 7.62 7.62 7.56 7.54 7.53 7.52 7.51 7.51 7.49 7.48 7.47 7.46 7.45 7.44 3.93 3.92 3.91 3.90 3.89 3.88 3.88 3.87 3.40 3.35 1.56 1.56 1.54 1.53 1.52 1.51 1.51 1.49 1.32 1.30 1.28 1.26 1.24 1.22 0.86 0.84 0.82
3b
PO(On-Bu)2
N
Ph
1.00 0.98 1.03 1.02 2.06 4.28 4.11 2.06 4.09 4.20 6.05
9.5 9.0 8.5 8.0 7.5 7.0 6.5 6.0 5.5 5.0 4.5 4.0 3.5 3.0 2.5 2.0 1.5 1.0 0.5 0.0
f1 (ppm)

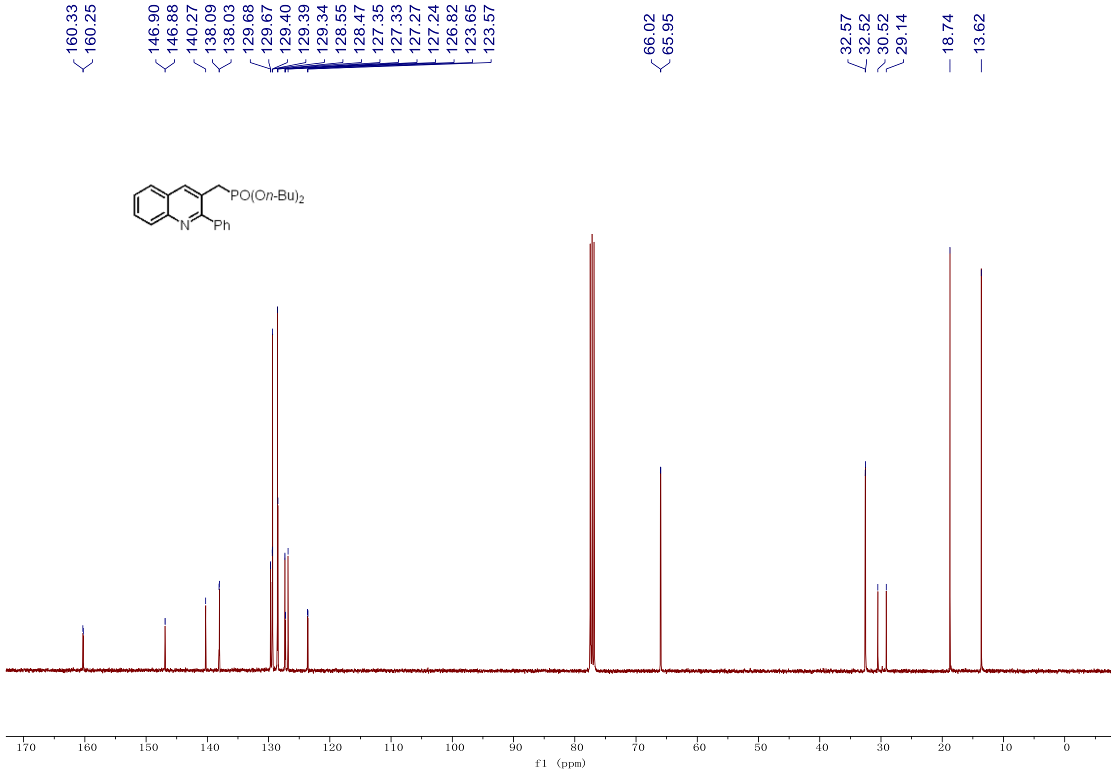
160.33
160.25
146.90
146.88
140.27
138.09
138.03
129.68
129.67
129.40
129.39
129.34
128.55
128.47
127.35
127.33
127.27
127.24
126.82
123.65
123.57
66.02
65.95
32.57
32.52
30.52
29.14
18.74
13.62
PO(On-Bu)2
N
Ph
f1 (ppm)

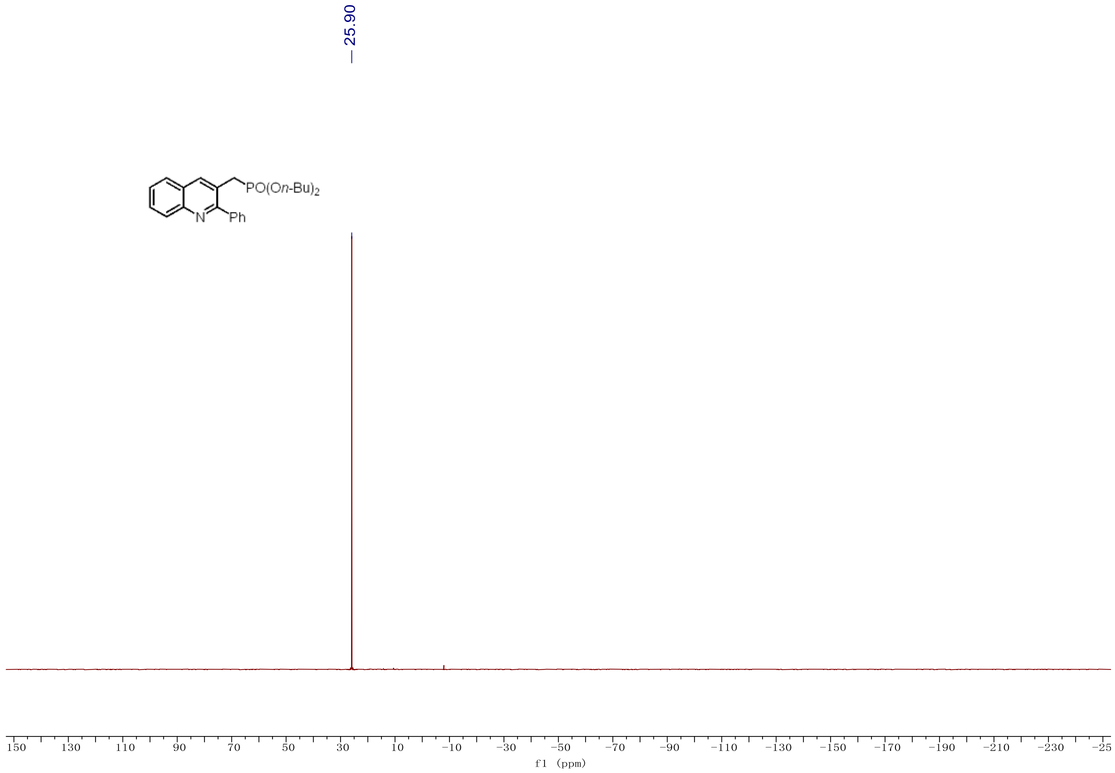
25.90
PO(On-Bu)2
N
Ph
f1 (ppm)

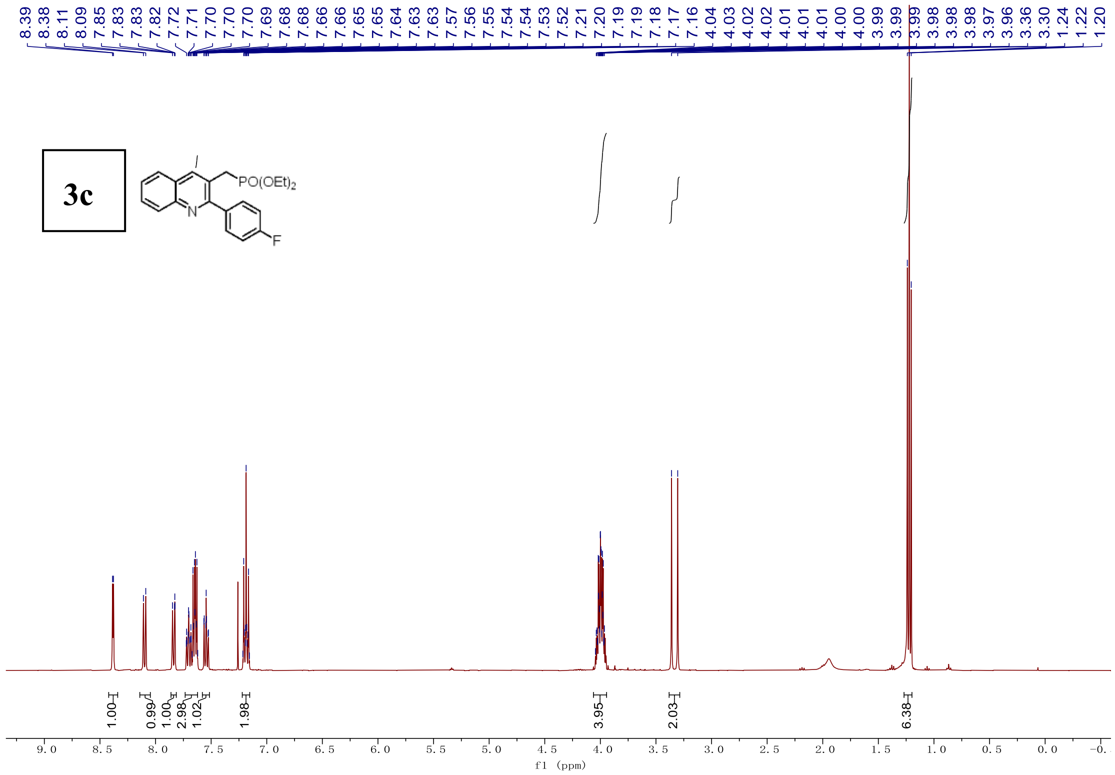

8.39 8.38 8.11 8.09 7.85 7.83 7.83 7.82 7.72 7.71 7.70 7.70 7.69 7.68 7.68 7.66 7.65 7.65 7.64 7.63 7.63 7.57 7.56 7.55 7.54 7.54 7.53 7.52 7.21 7.20 7.19 7.19 7.18 7.17 7.16 4.04 4.03 4.02 4.02 4.01 4.01 4.01 4.00 4.00 3.99 3.99 3.99 3.98 3.98 3.98 3.97 3.96 3.36 3.30 1.24 1.22 1.20
3c
PO(OEt)2
N
F
1.00 0.99 1.00 2.98 1.02 1.98 3.95 2.03 6.38
9.0 8.5 8.0 7.5 7.0 6.5 6.0 5.5 5.0 4.5 4.0 3.5 3.0 2.5 2.0 1.5 1.0 0.5 0.0 -0.
f1 (ppm)

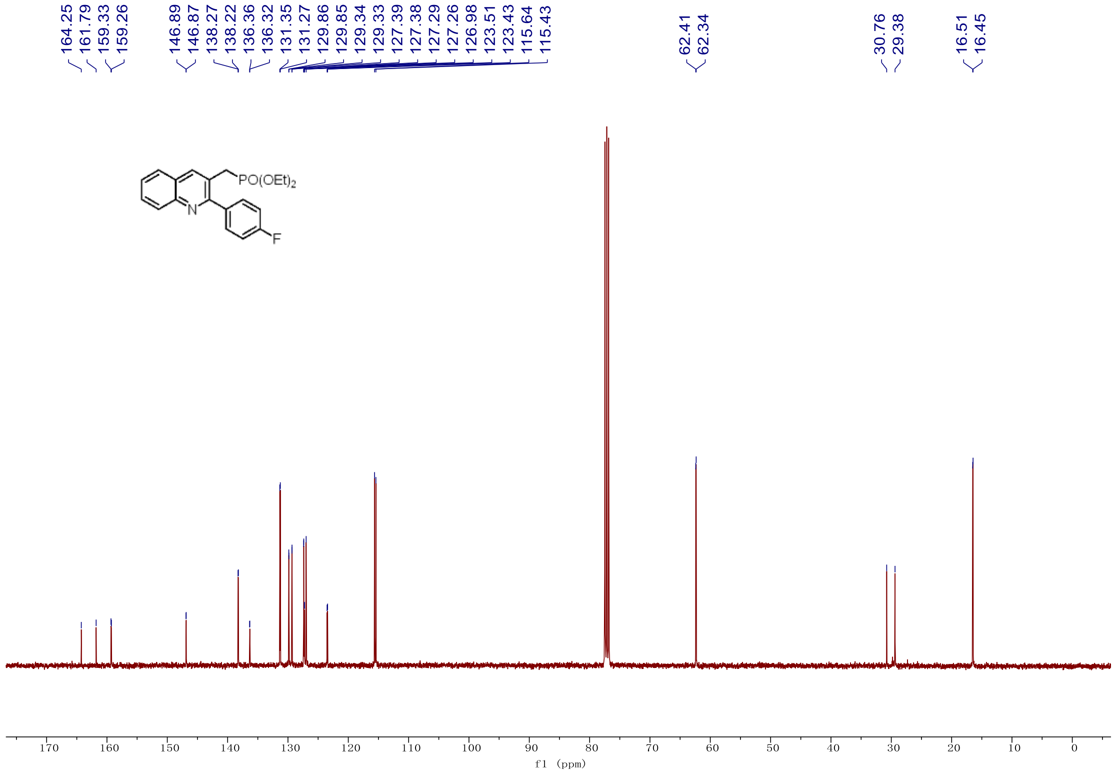

164.25 161.79 159.33 159.26 146.89 146.87 138.27 138.22 136.36 136.32 131.35 131.27 129.86 129.85 129.34 129.33 127.39 127.38 127.29 127.26 126.98 123.51 123.43 115.64 115.43 62.41 62.34 30.76 29.38 16.51 16.45
PO(OEt)2
N
F
170 160 150 140 130 120 110 100 90 80 70 60 50 40 30 20 10 0
f1 (ppm)

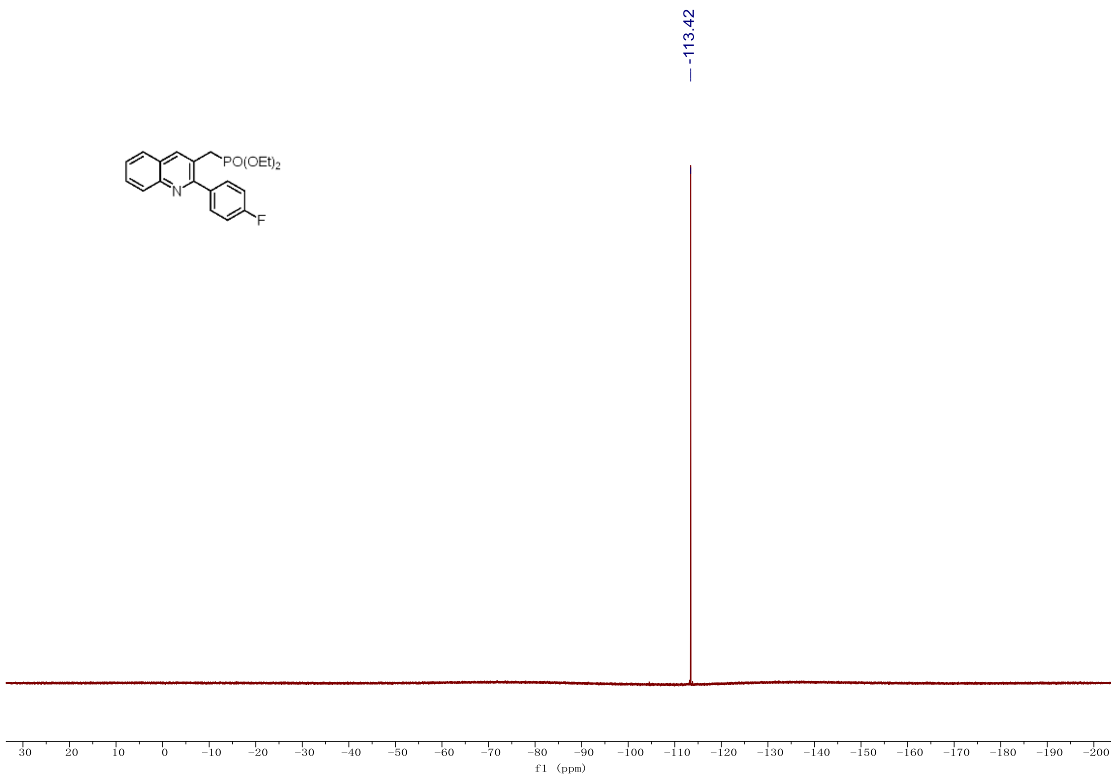
-113.42
PO(OEt)2
N
F
f1 (ppm)

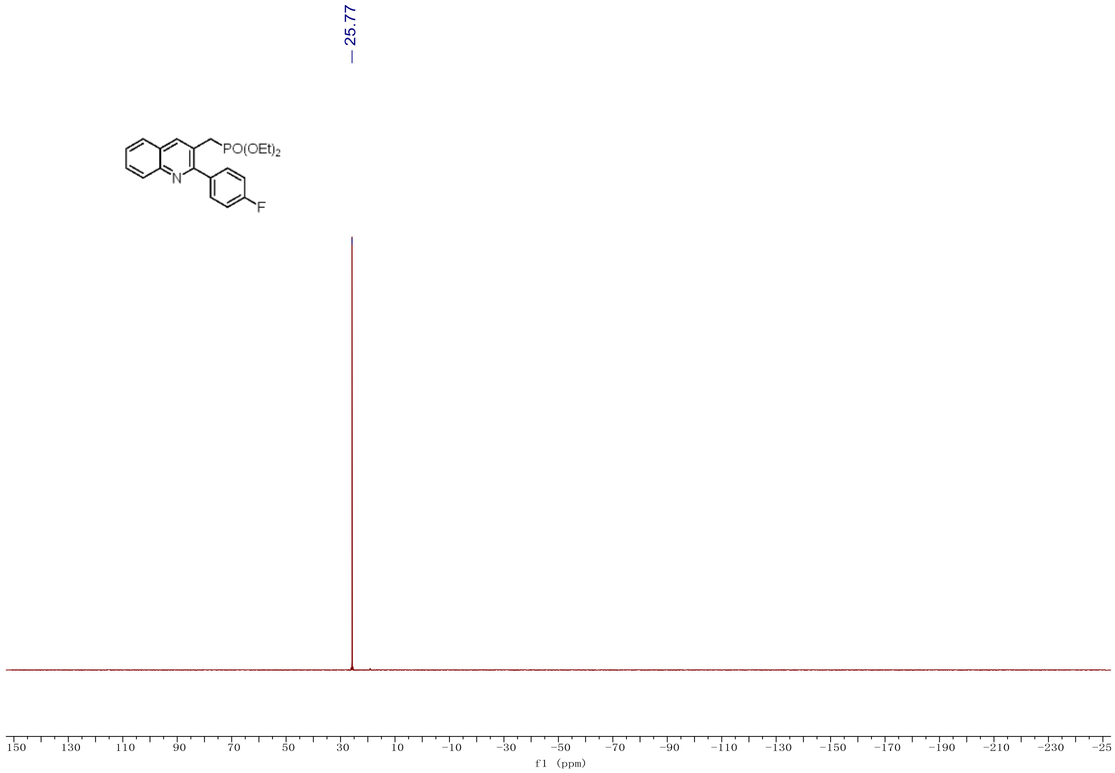
25.77
PO(OEt)2
N
F
f1 (ppm)

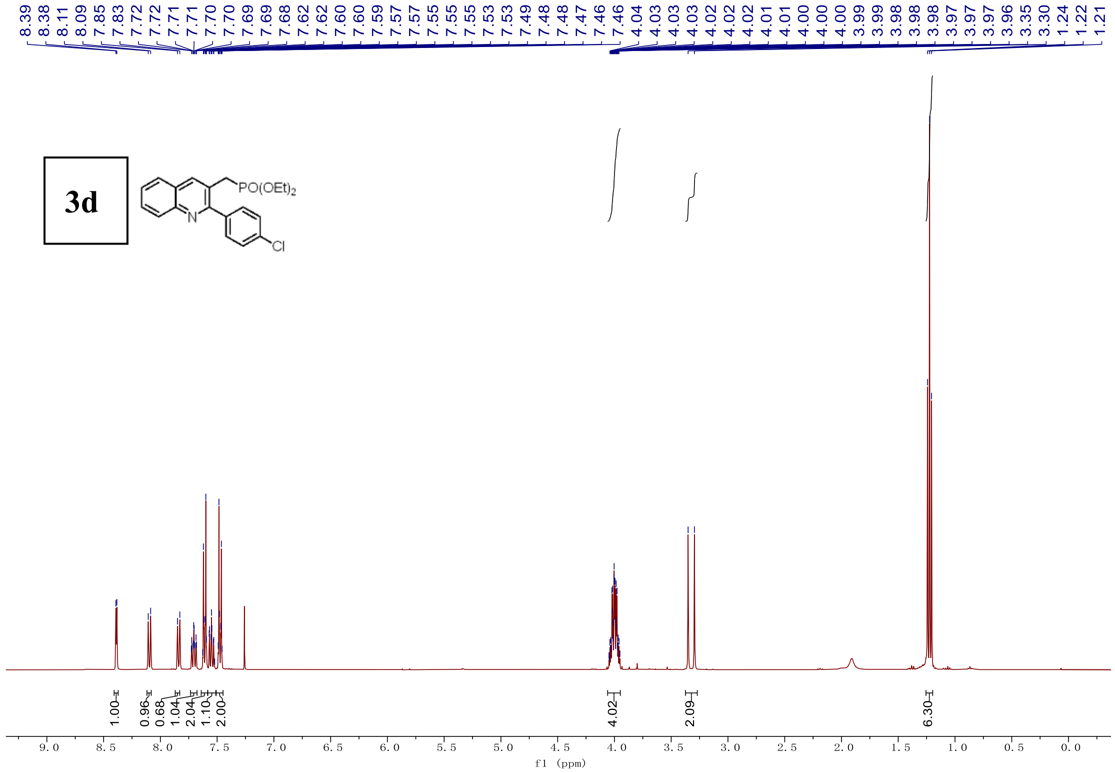
3d
PO(OEt)2
N
Cl
8.39 8.38 8.11 8.09 7.85 7.83 7.72 7.72 7.71 7.71 7.70 7.70 7.69 7.69 7.68 7.62 7.62 7.60 7.59 7.57 7.57 7.55 7.55 7.53 7.53 7.49 7.48 7.48 7.47 7.46 7.46 4.04 4.03 4.03 4.03 4.02 4.02 4.02 4.01 4.01 4.00 4.00 4.00 3.99 3.99 3.98 3.98 3.98 3.97 3.97 3.96 3.35 3.30 1.24 1.22 1.21
1.00 0.96 0.68 1.04 2.04 1.10 2.00 4.02 2.09 6.30
9.0 8.5 8.0 7.5 7.0 6.5 6.0 5.5 5.0 4.5 4.0 3.5 3.0 2.5 2.0 1.5 1.0 0.5 0.0
f1 (ppm)

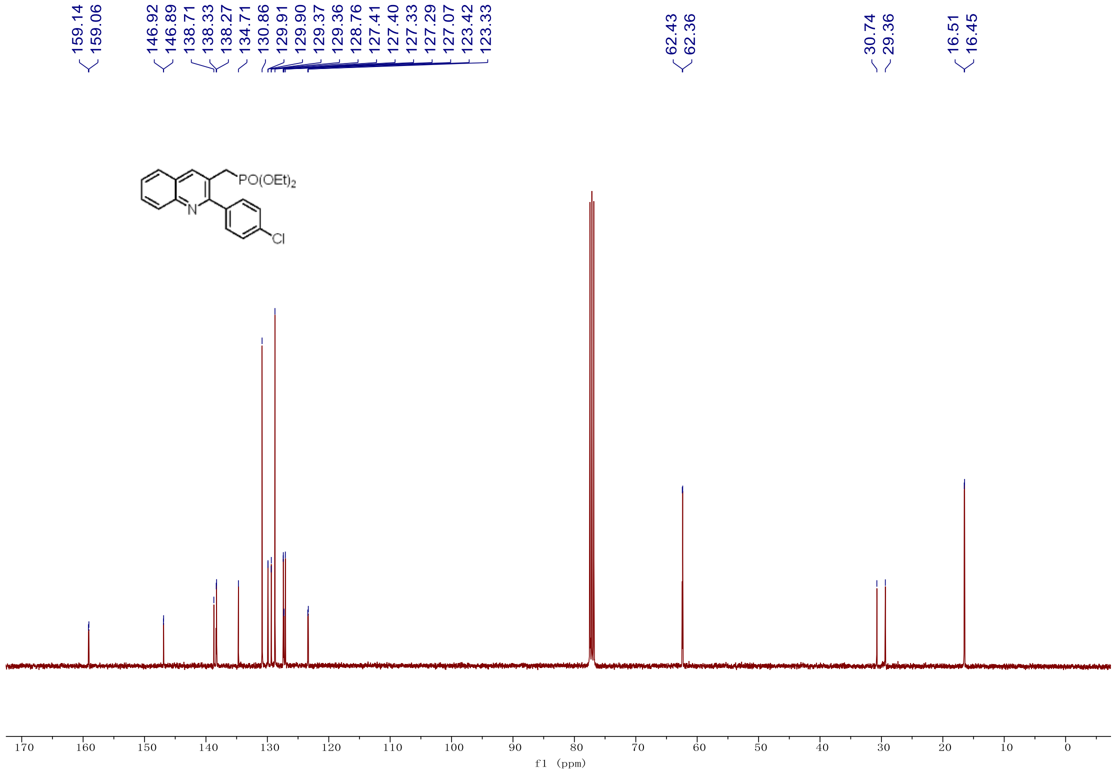
159.14 159.06 146.92 146.89 138.71 138.33 138.27 134.71 130.86 129.91 129.90 129.37 129.36 128.76 127.41 127.40 127.33 127.29 127.07 123.42 123.33 62.43 62.36 30.74 29.36 16.51 16.45
PO(OEt)2
N
Cl
170 160 150 140 130 120 110 100 90 80 70 60 50 40 30 20 10 0
f1 (ppm)

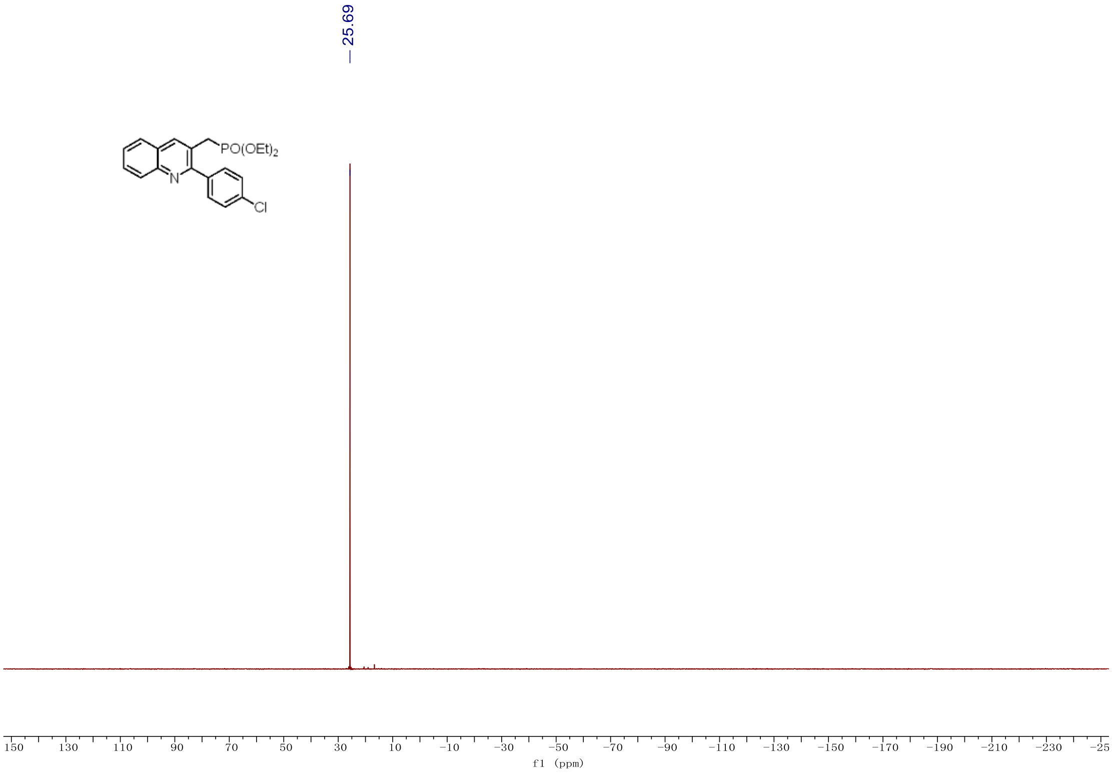
25.69
PO(OEt)2
N
Cl
f1 (ppm)

3e

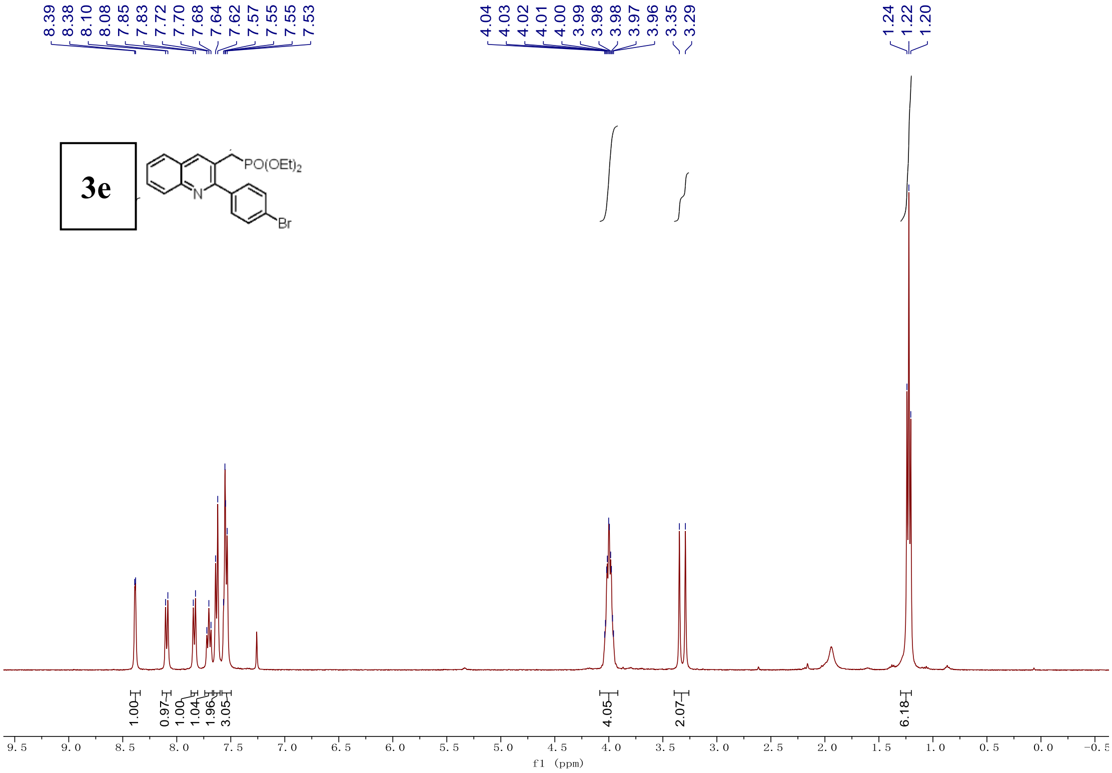
8.39
8.38
8.10
8.08
7.85
7.83
7.72
7.70
7.68
7.64
7.62
7.57
7.55
7.55
7.53
4.04
4.03
4.02
4.01
4.00
3.99
3.98
3.98
3.97
3.96
3.35
3.29
1.24
1.22
1.20
PO(OEt)2
N
Br
1.00
0.97
1.00
1.04
1.96
3.05
4.05
2.07
6.18
f1 (ppm)

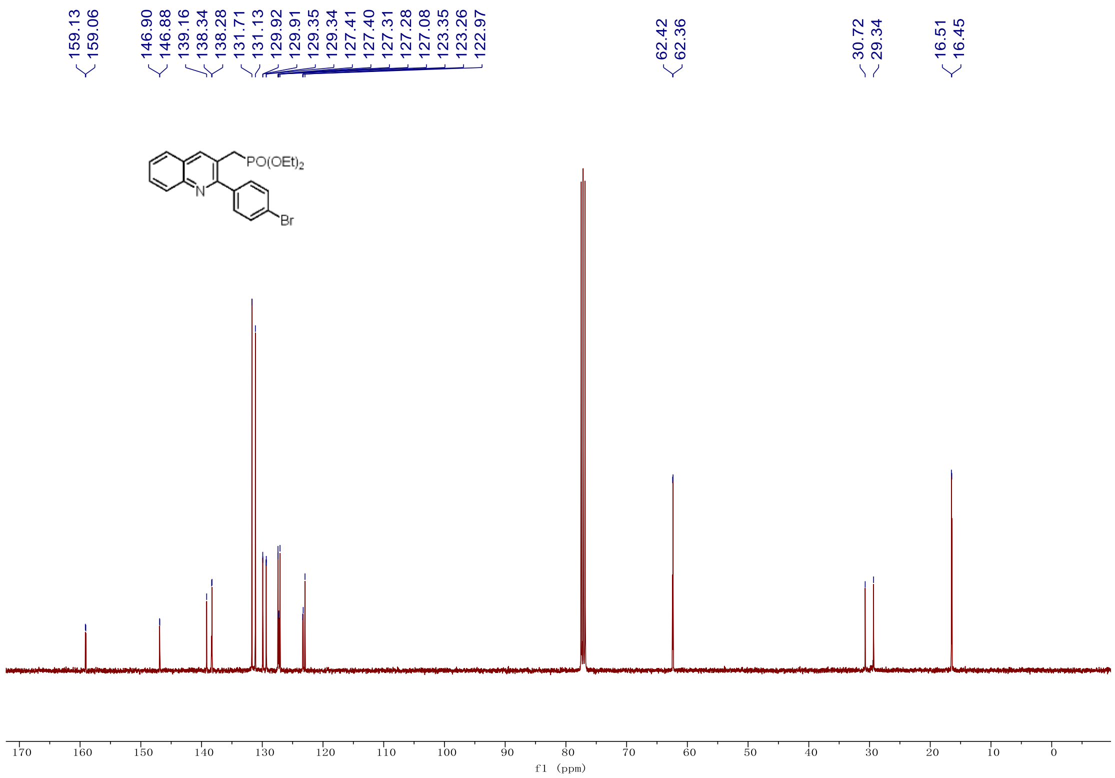
159.13
159.06
146.90
146.88
139.16
138.34
138.28
131.71
131.13
129.92
129.91
129.35
129.34
127.41
127.40
127.31
127.28
127.08
123.35
123.26
122.97
62.42
62.36
30.72
29.34
16.51
16.45
PO(OEt)2
N
Br
f1 (ppm)

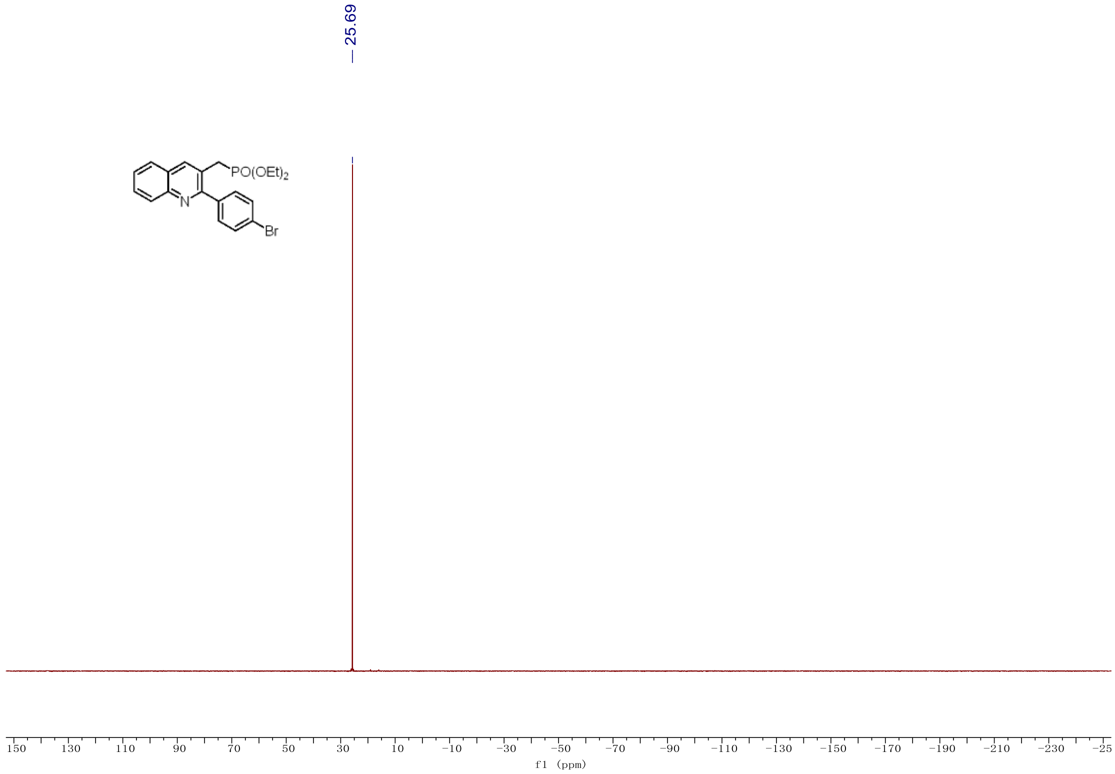
25.69
PO(OEt)2
N
Br
f1 (ppm)

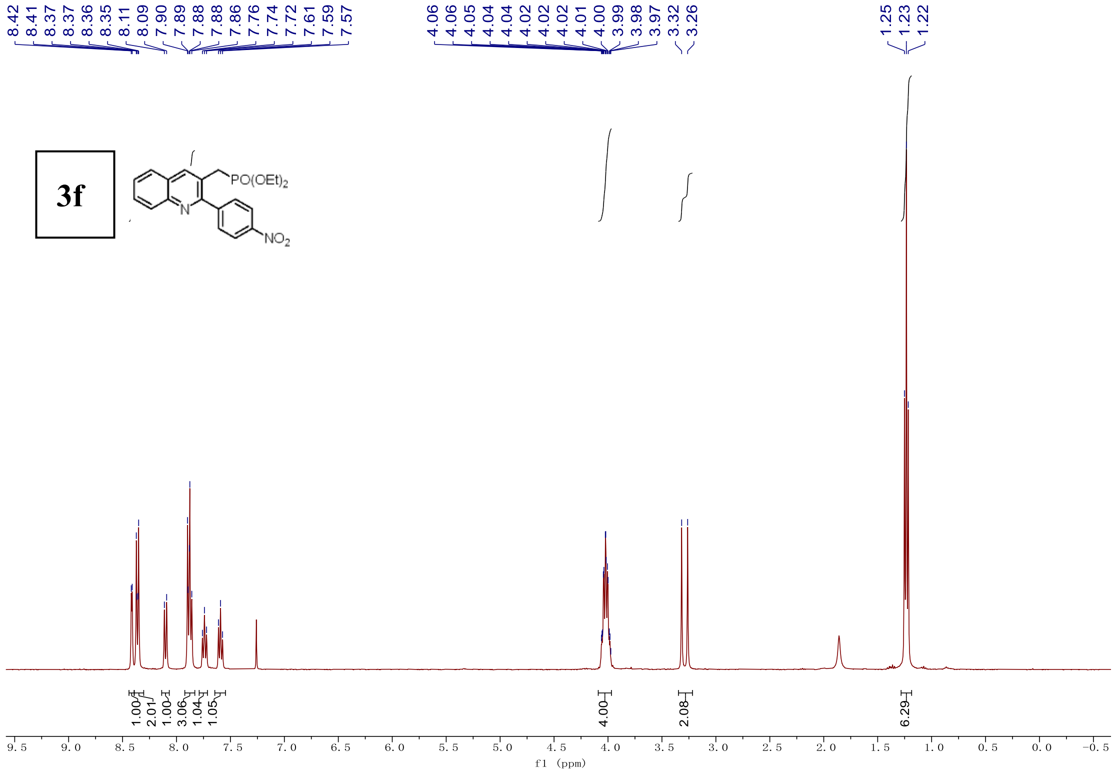
8.42 8.41 8.37 8.37 8.36 8.35 8.11 8.09 7.90 7.89 7.88 7.88 7.86 7.76 7.74 7.72 7.61 7.59 7.57
4.06 4.06 4.05 4.04 4.04 4.02 4.02 4.02 4.01 4.00 3.99 3.98 3.97 3.32 3.26
1.25 1.23 1.22
3f
PO(OEt)2
N
NO2
1.00 2.01 1.00 3.06 1.04 1.05
4.00
2.08
6.29
9.5 9.0 8.5 8.0 7.5 7.0 6.5 6.0 5.5 5.0 4.5 4.0 3.5 3.0 2.5 2.0 1.5 1.0 0.5 0.0 -0.5
f1 (ppm)

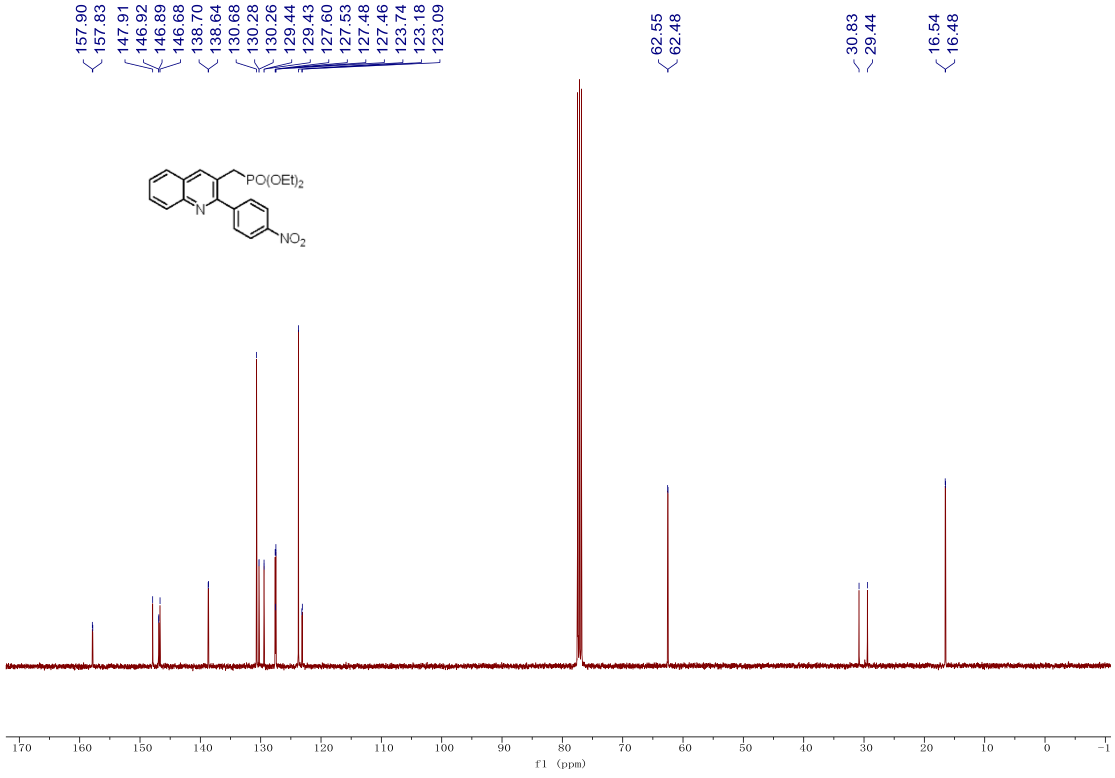
157.90 157.83 147.91 146.92 146.89 146.68 138.70 138.64 130.68 130.28 130.26 129.44 129.43 127.60 127.53 127.48 127.46 123.74 123.18 123.09
62.55 62.48
30.83 29.44
16.54 16.48
PO(OEt)2
N
NO2
170 160 150 140 130 120 110 100 90 80 70 60 50 40 30 20 10 0 -1
f1 (ppm)

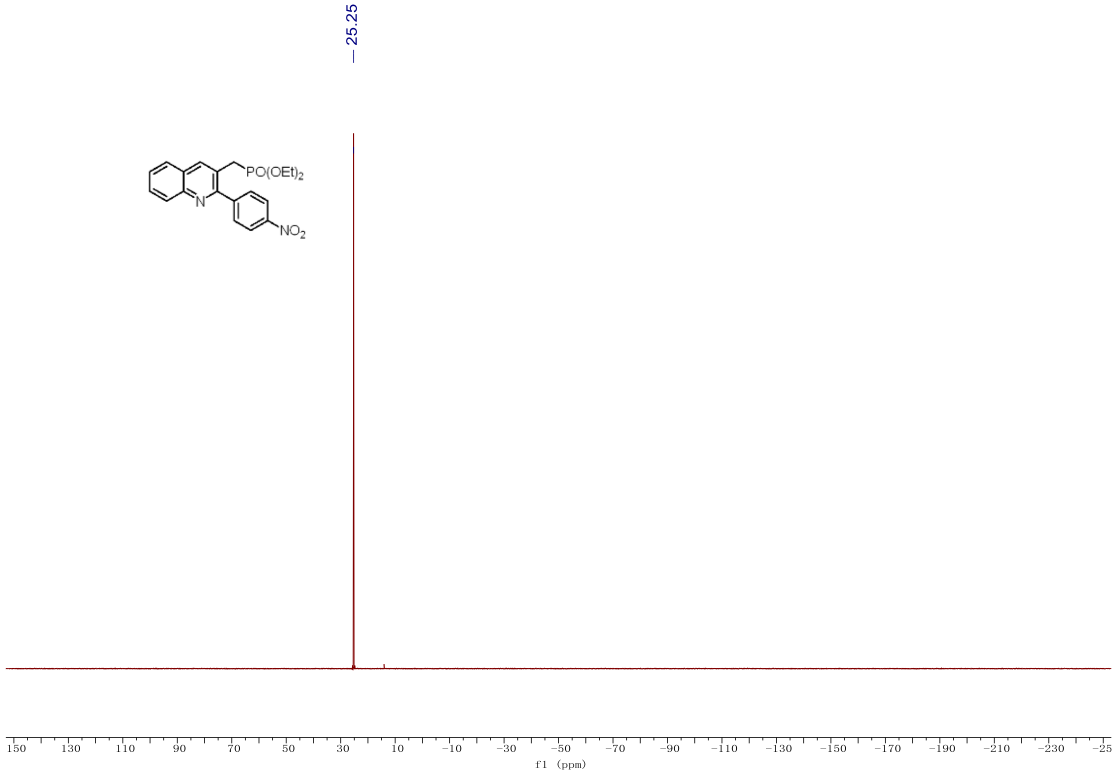

25.25
PO(OEt)2
N
NO2
f1 (ppm)

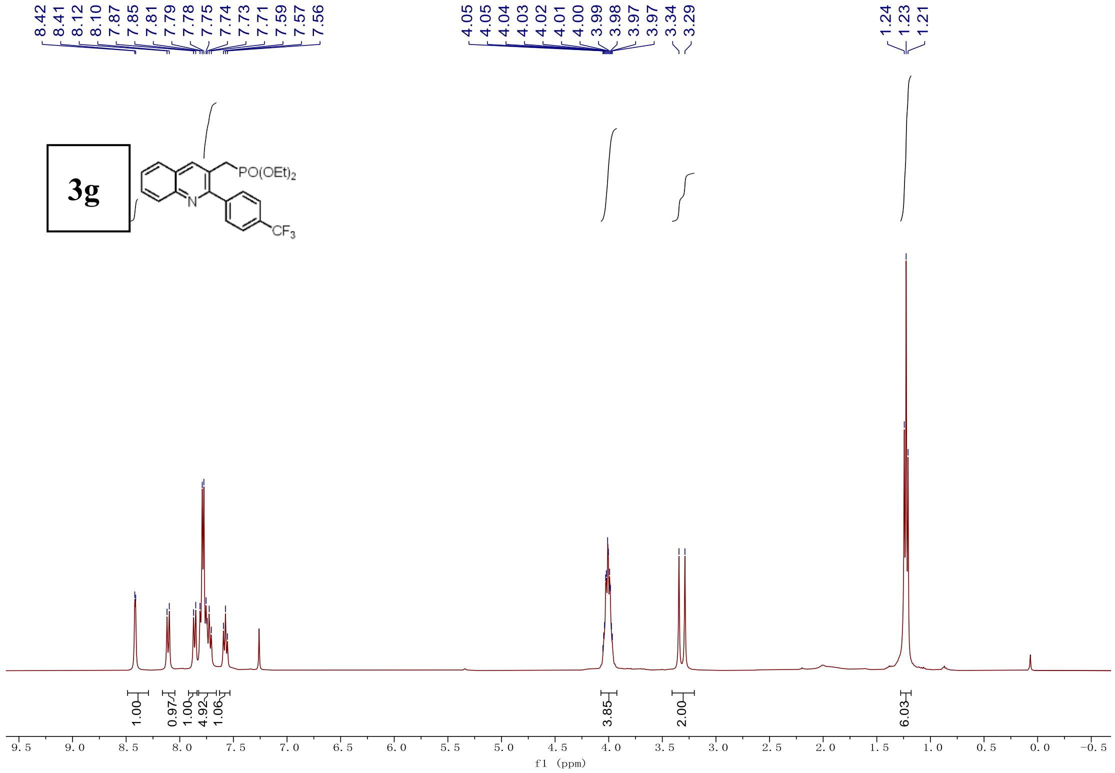

8.42
8.41
8.12
8.10
7.87
7.85
7.81
7.79
7.78
7.75
7.74
7.73
7.71
7.59
7.57
7.56
4.05
4.05
4.04
4.03
4.02
4.01
4.00
3.99
3.98
3.97
3.97
3.34
3.29
1.24
1.23
1.21
3g
PO(OEt)2
N
CF3
1.00
0.97
1.00
4.92
1.06
3.85
2.00
6.03
f1 (ppm)

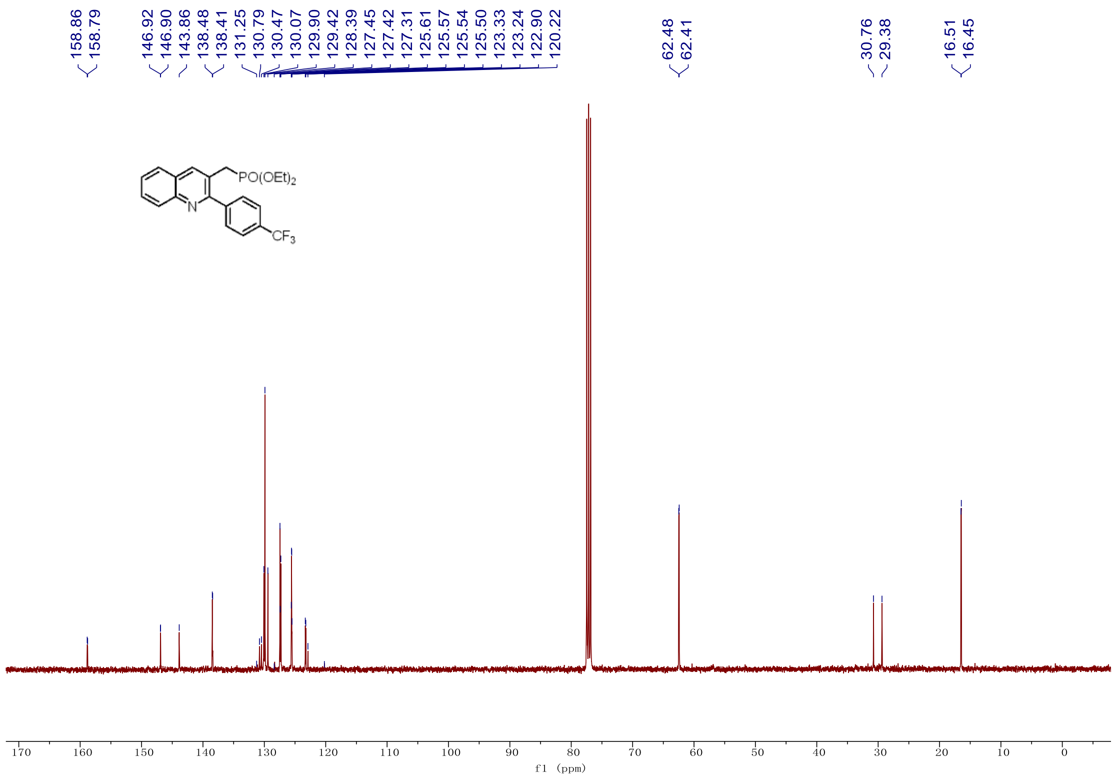

158.86
158.79
146.92
146.90
143.86
138.48
138.41
131.25
130.79
130.47
130.07
129.90
129.42
128.39
127.45
127.42
127.31
125.61
125.57
125.54
125.50
123.33
123.24
122.90
120.22
62.48
62.41
30.76
29.38
16.51
16.45
PO(OEt)2
N
CF3
f1 (ppm)

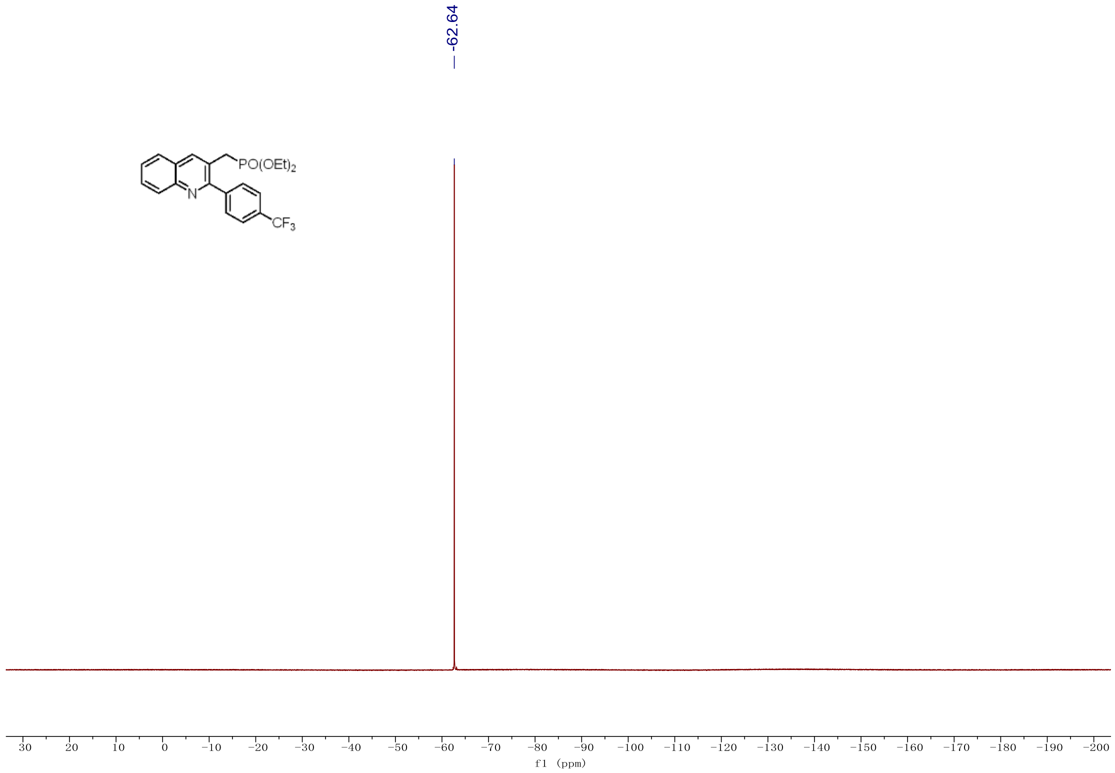

-62.64
PO(OEt)2
N
CF3
f1 (ppm)

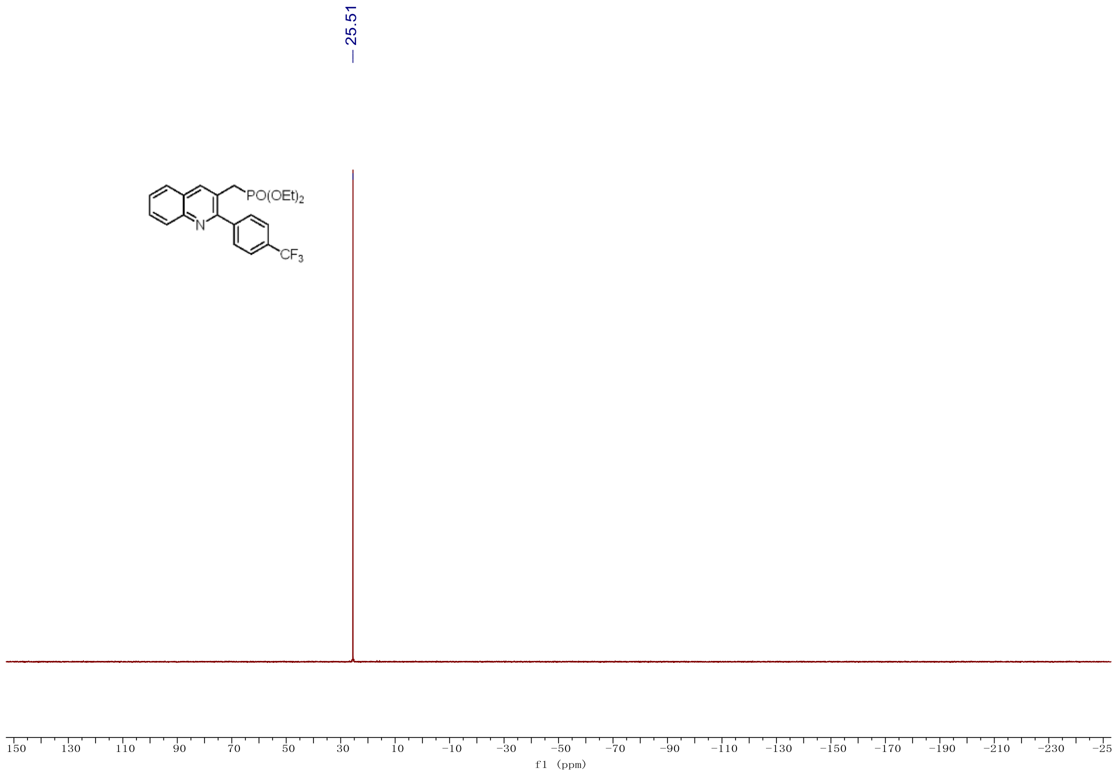

25.51
PO(OEt)2
N
CF3
150 130 110 90 70 50 30 10 −10 −30 −50 −70 −90 −110 −130 −150 −170 −190 −210 −230 −25
f1 (ppm)

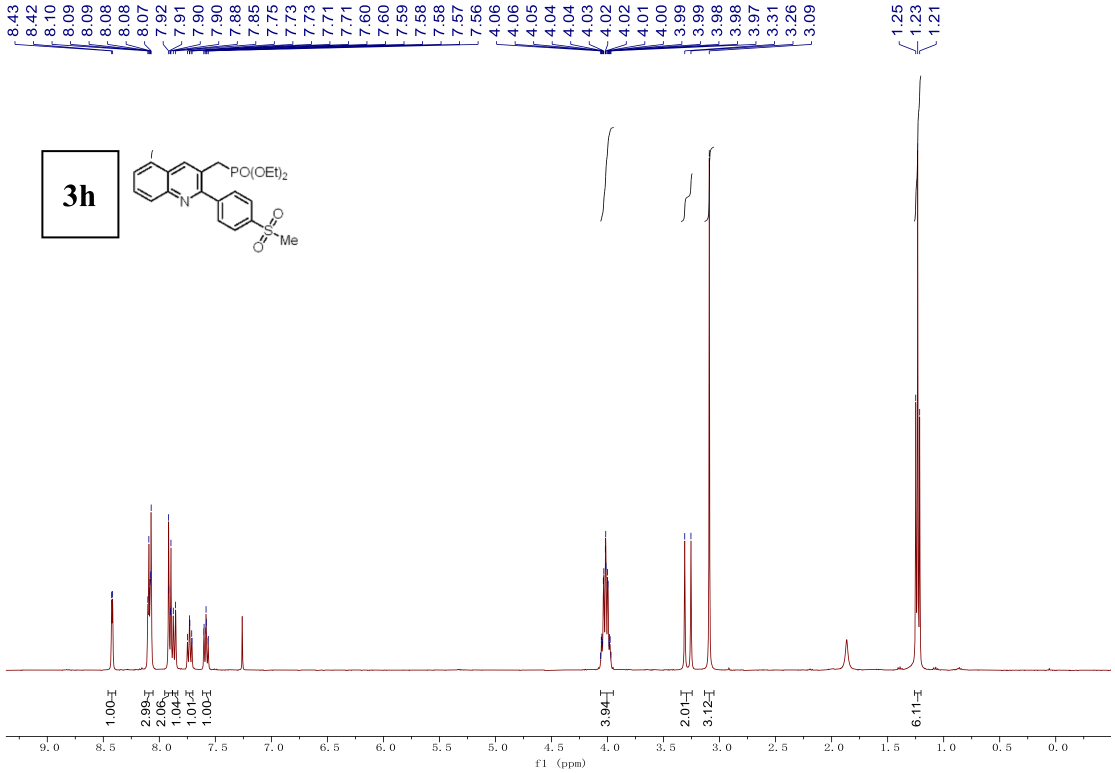

8.43 8.42 8.10 8.09 8.09 8.08 8.08 8.07 7.92 7.91 7.90 7.90 7.88 7.85 7.75 7.73 7.73 7.71 7.71 7.60 7.59 7.58 7.58 7.57 7.56 4.06 4.05 4.04 4.04 4.03 4.02 4.02 4.01 4.00 3.99 3.99 3.98 3.98 3.97 3.31 3.26 3.09
1.25 1.23 1.21
3h
PO(OEt)2
N
O
S
O
Me
1.00 2.99 2.06 1.04 1.01 1.00 3.94 2.01 3.12 6.11
9.0 8.5 8.0 7.5 7.0 6.5 6.0 5.5 5.0 4.5 4.0 3.5 3.0 2.5 2.0 1.5 1.0 0.5 0.0
f1 (ppm)

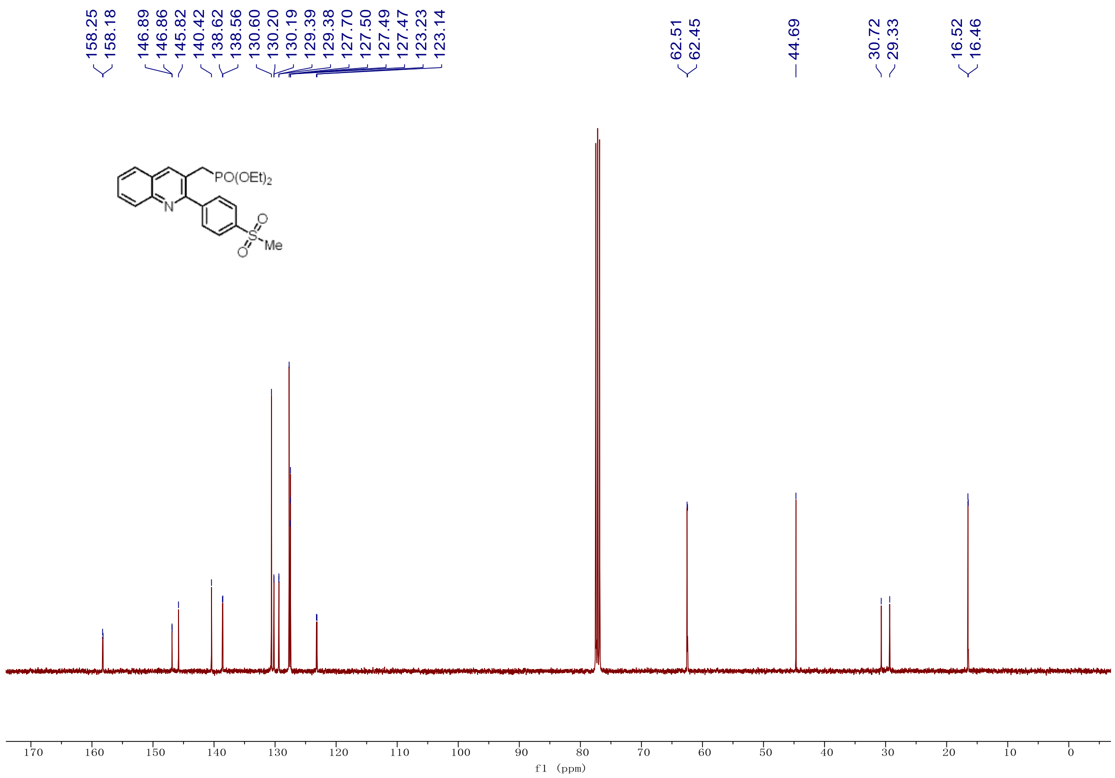
158.25
158.18
146.89
146.86
145.82
140.42
138.62
138.56
130.60
130.20
130.19
129.39
129.38
127.70
127.50
127.49
127.47
123.23
123.14
62.51
62.45
44.69
30.72
29.33
16.52
16.46
PO(OEt)2
N
O
S
Me
O
f1 (ppm)

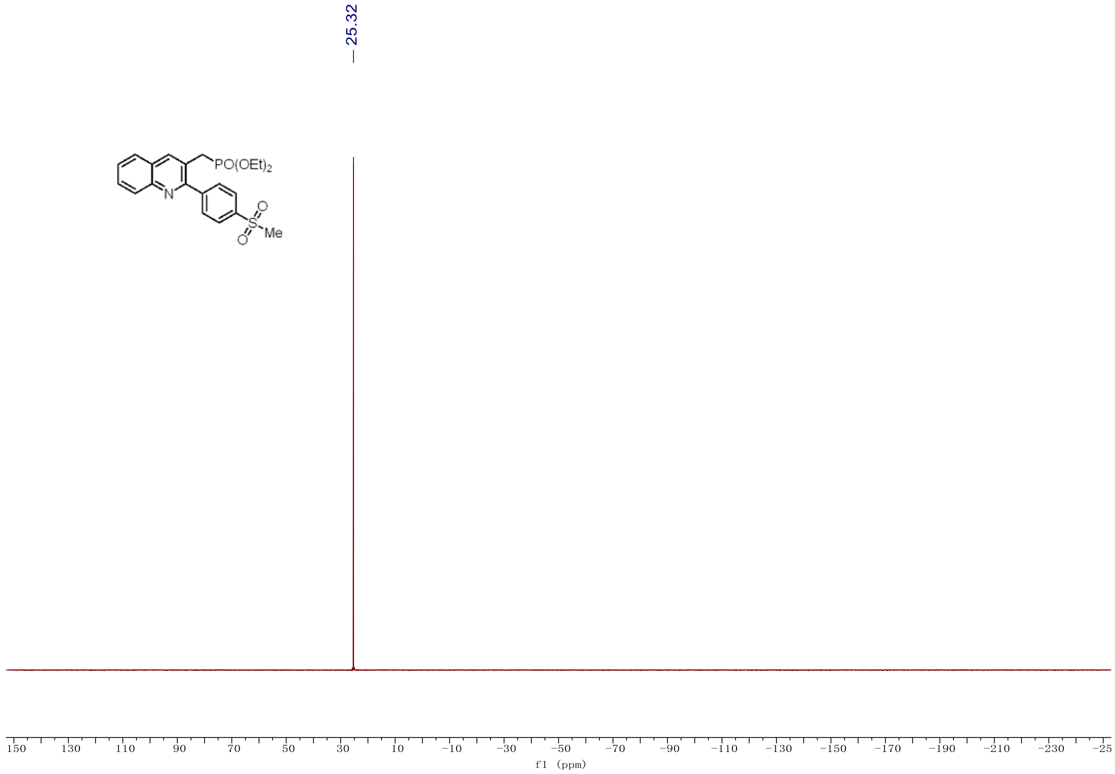
25.32
PO(OEt)2
N
O
S
Me
O
f1 (ppm)

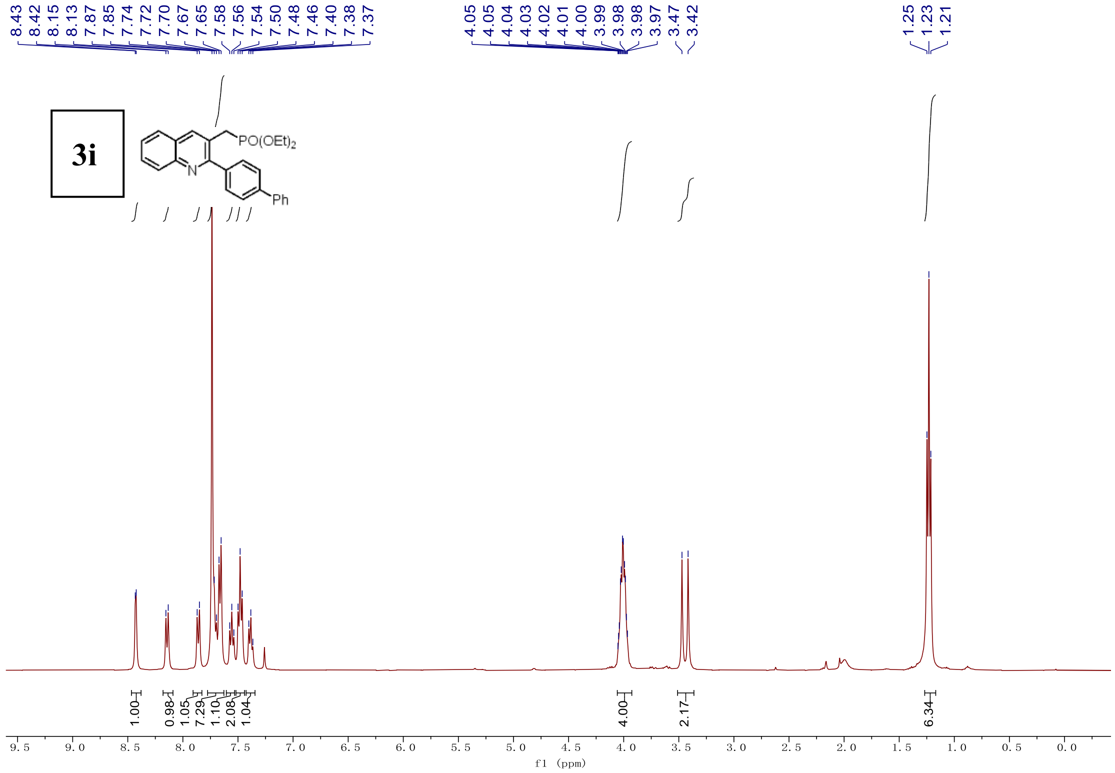
8.43
8.42
8.15
8.13
7.87
7.85
7.74
7.72
7.70
7.67
7.65
7.58
7.56
7.54
7.50
7.48
7.46
7.40
7.38
7.37
4.05
4.05
4.04
4.03
4.02
4.01
4.00
3.99
3.98
3.98
3.97
3.47
3.42
1.25
1.23
1.21
3i
PO(OEt)2
N
Ph
1.00
0.98
1.05
7.29
1.10
2.08
1.04
4.00
2.17
6.34
f1 (ppm)

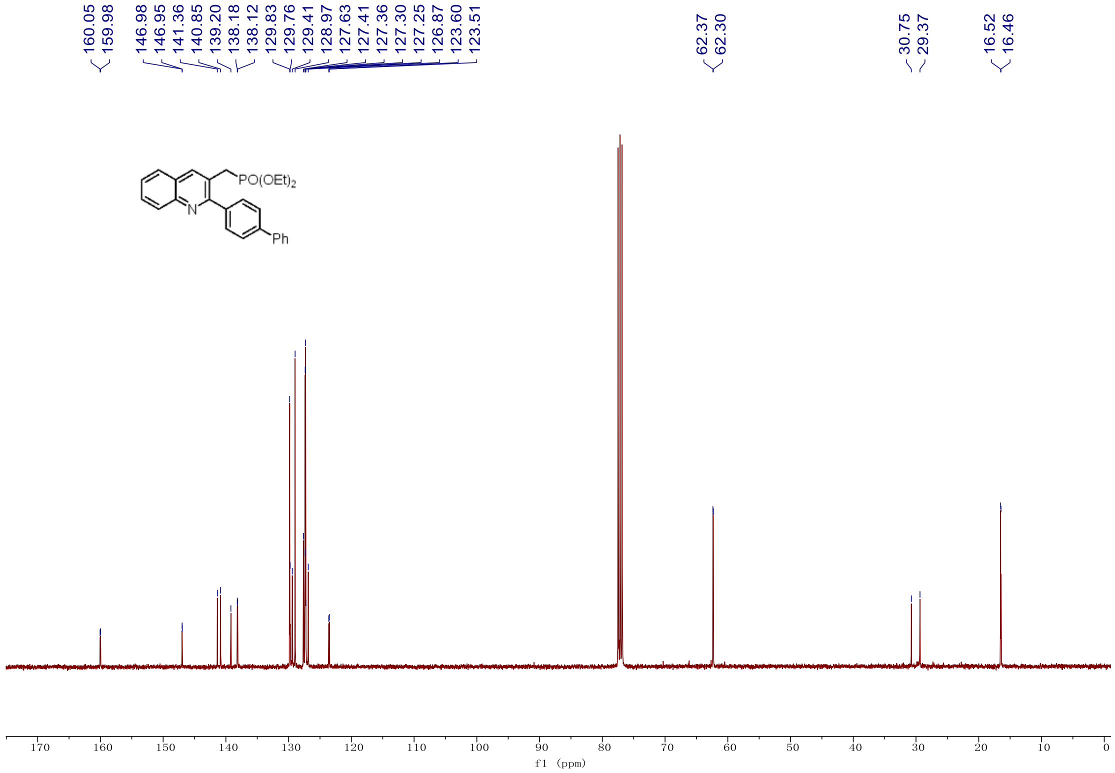
160.05
159.98
146.98
146.95
141.36
140.85
139.20
138.18
138.12
129.83
129.76
129.41
128.97
127.63
127.41
127.36
127.30
127.25
126.87
123.60
123.51
62.37
62.30
30.75
29.37
16.52
16.46
PO(OEt)2
N
Ph
f1 (ppm)

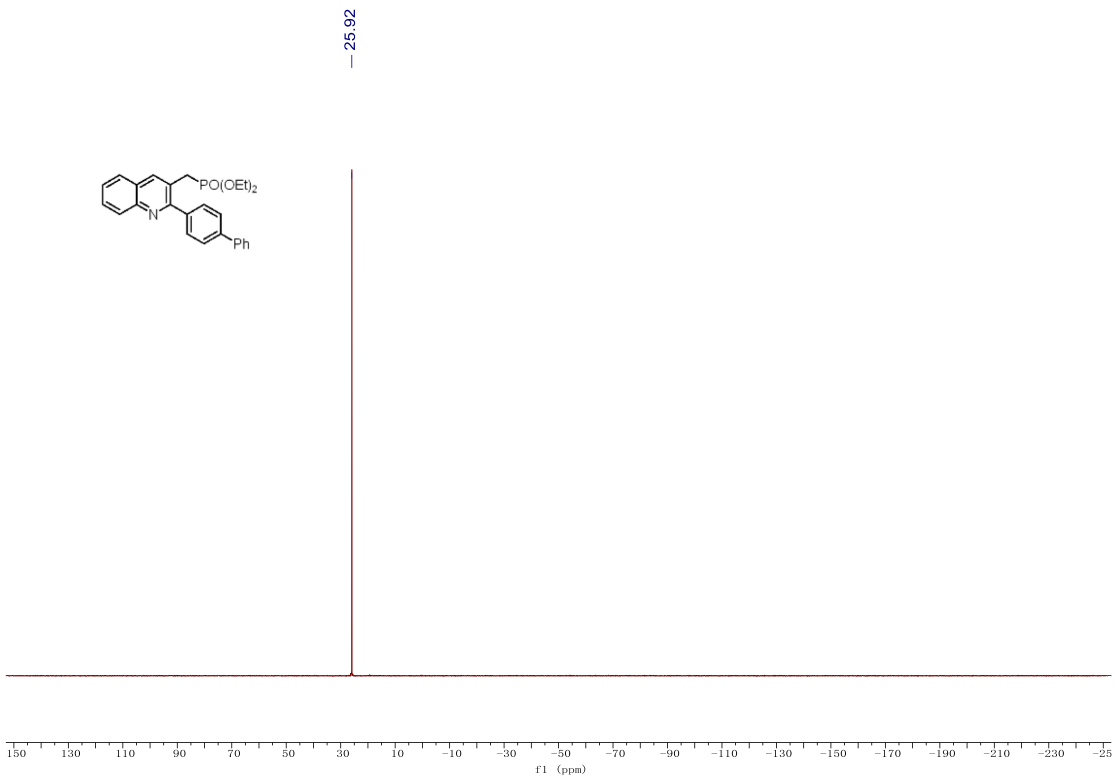

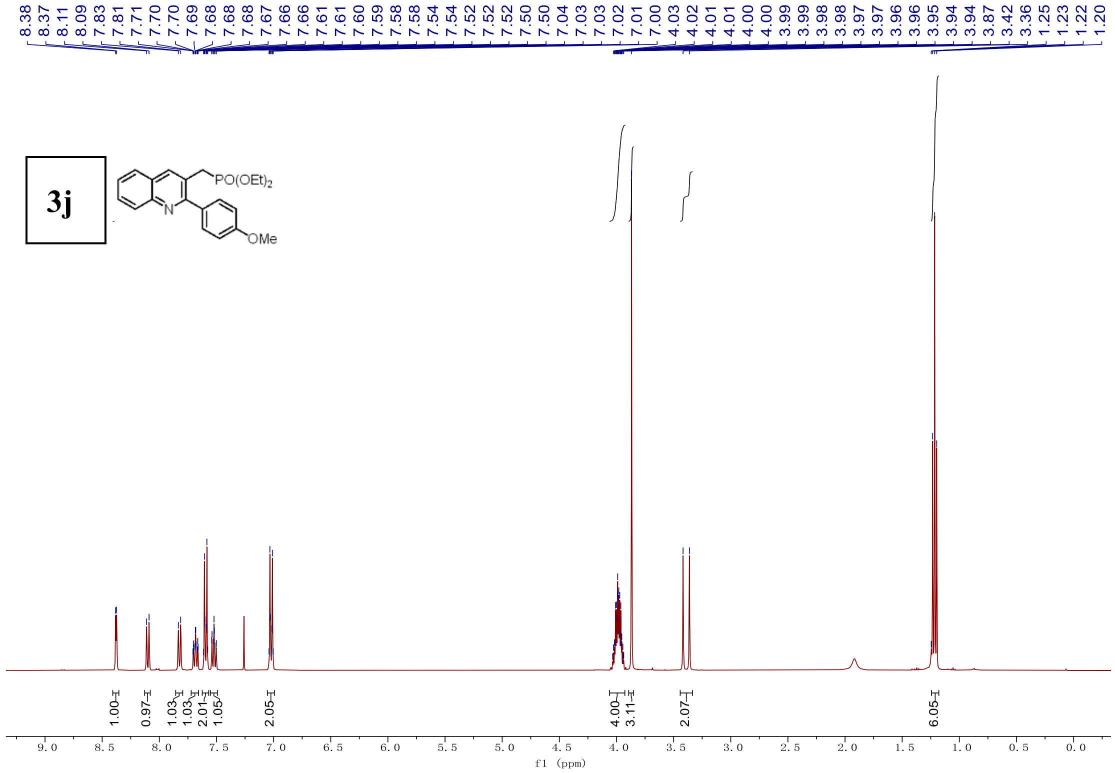

3j

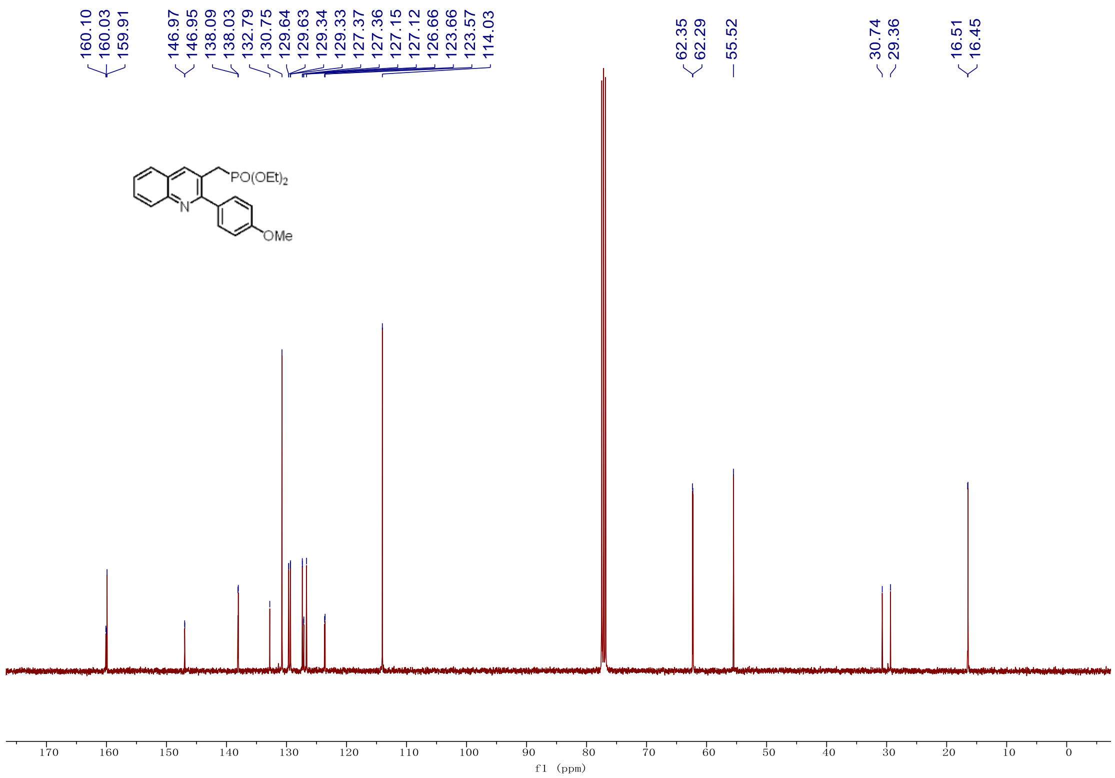
160.10
160.03
159.91
146.97
146.95
138.09
138.03
132.79
130.75
129.64
129.63
129.34
129.33
127.37
127.36
127.15
127.12
126.66
123.66
123.57
114.03
62.35
62.29
55.52
30.74
29.36
16.51
16.45
PO(OEt)2
N
OMe
f1 (ppm)

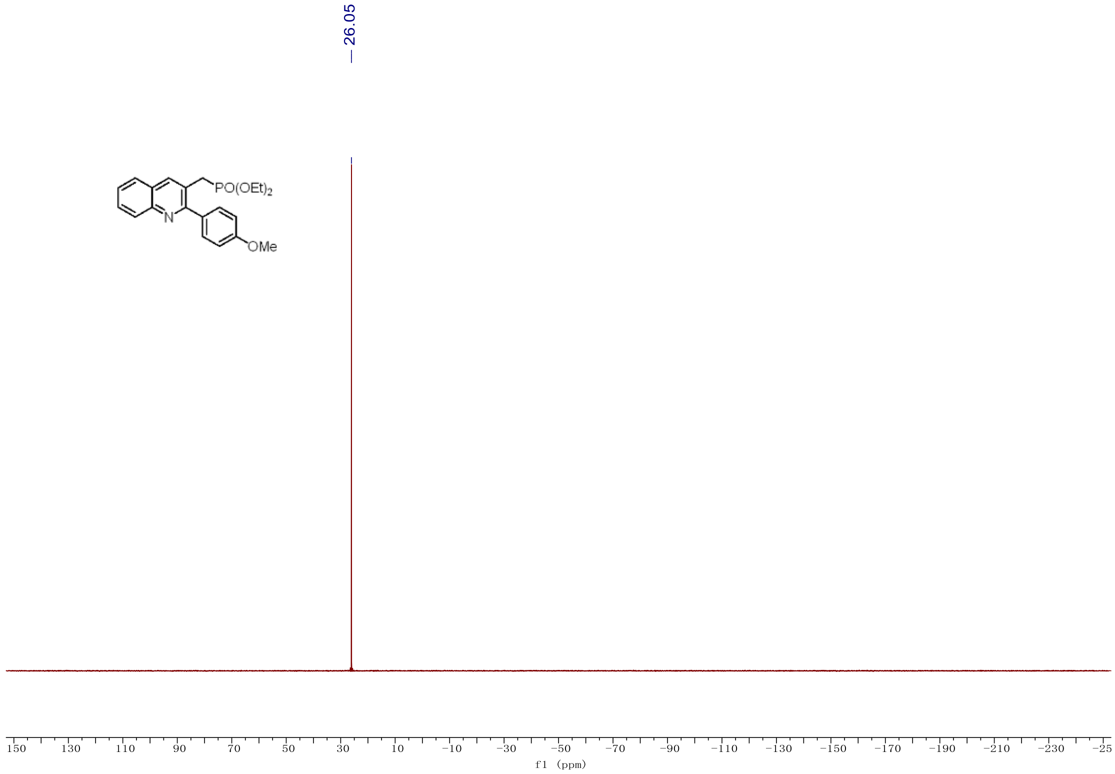
26.05
PO(OEt)2
N
OMe
f1 (ppm)

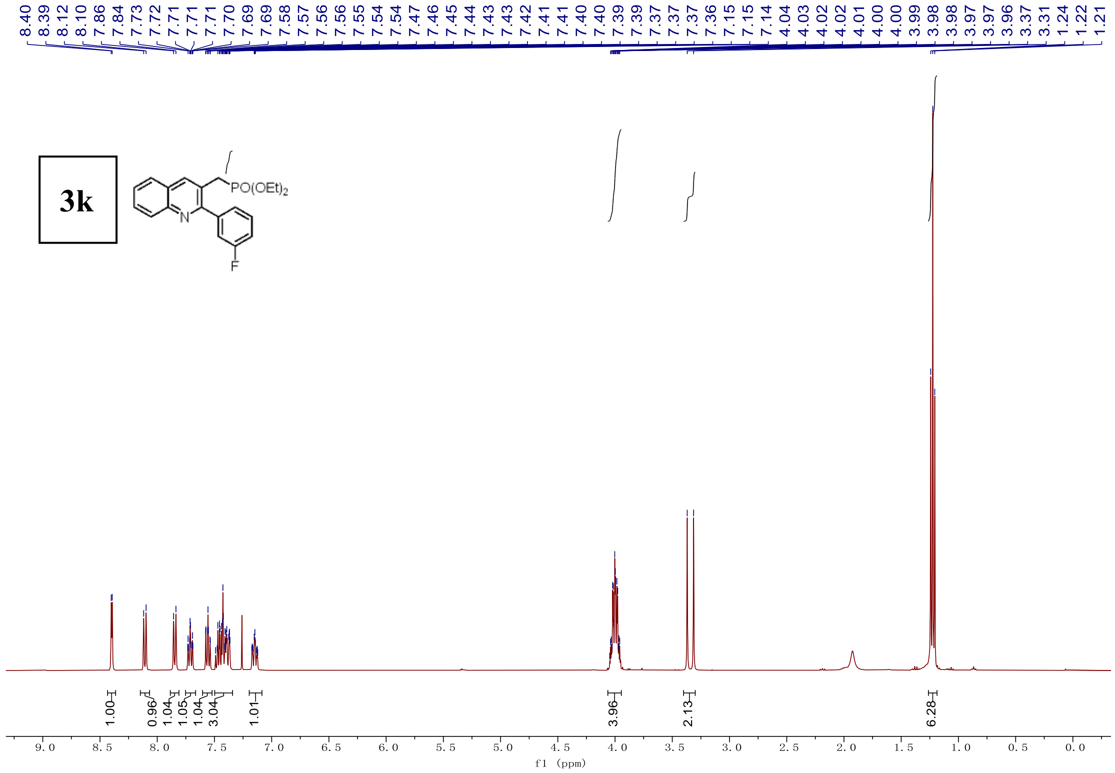
8.40 8.39 8.12 8.10 7.86 7.84 7.73 7.72 7.71 7.71 7.71 7.70 7.69 7.69 7.58 7.57 7.56 7.55 7.54 7.54 7.47 7.46 7.45 7.44 7.43 7.43 7.42 7.41 7.41 7.40 7.40 7.39 7.39 7.37 7.37 7.37 7.36 7.15 7.15 7.14 4.04 4.03 4.02 4.02 4.01 4.00 4.00 3.99 3.98 3.98 3.97 3.96 3.37 3.31 1.24 1.22 1.21
3k
PO(OEt)2
N
F
1.00 0.96 1.04 1.05 1.04 3.04 1.01 3.96 2.13 6.28
f1 (ppm)

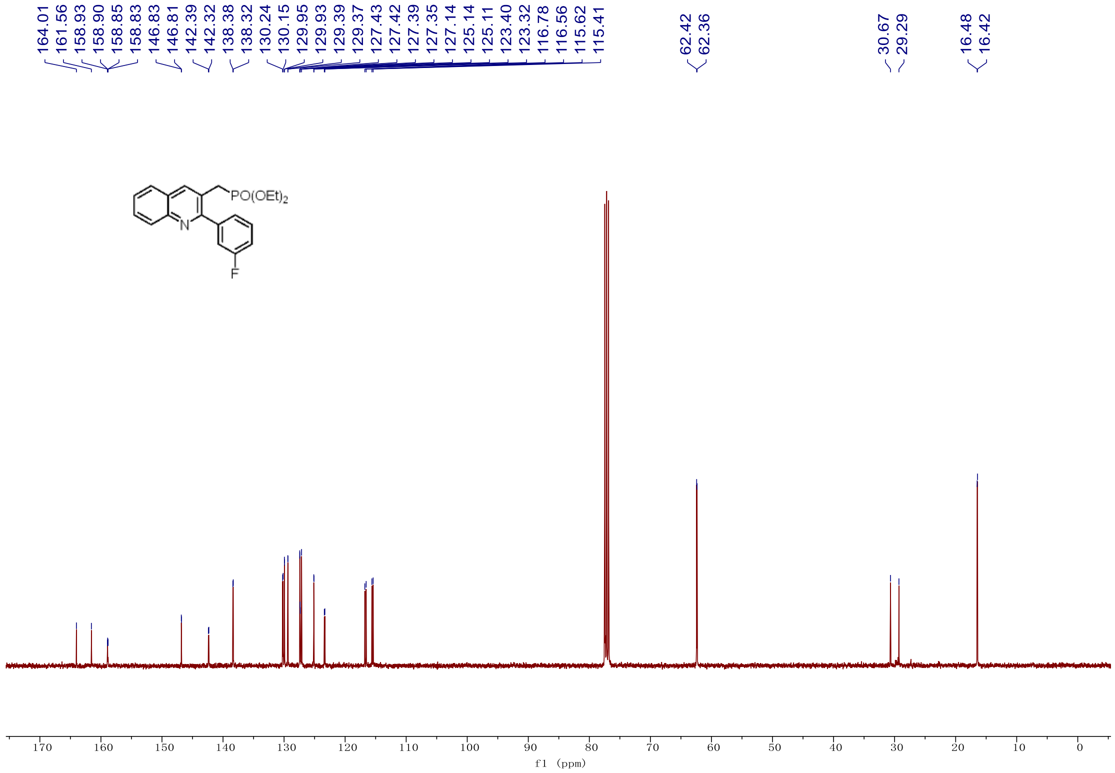
164.01 161.56 158.93 158.90 158.85 158.83 146.83 146.81 142.39 142.32 138.38 138.32 130.24 130.15 129.95 129.93 129.39 129.37 127.43 127.42 127.39 127.35 127.14 125.14 125.11 123.40 123.32 116.78 116.56 115.62 115.41
62.42 62.36
30.67 29.29
16.48 16.42
PO(OEt)2
N
F
f1 (ppm)

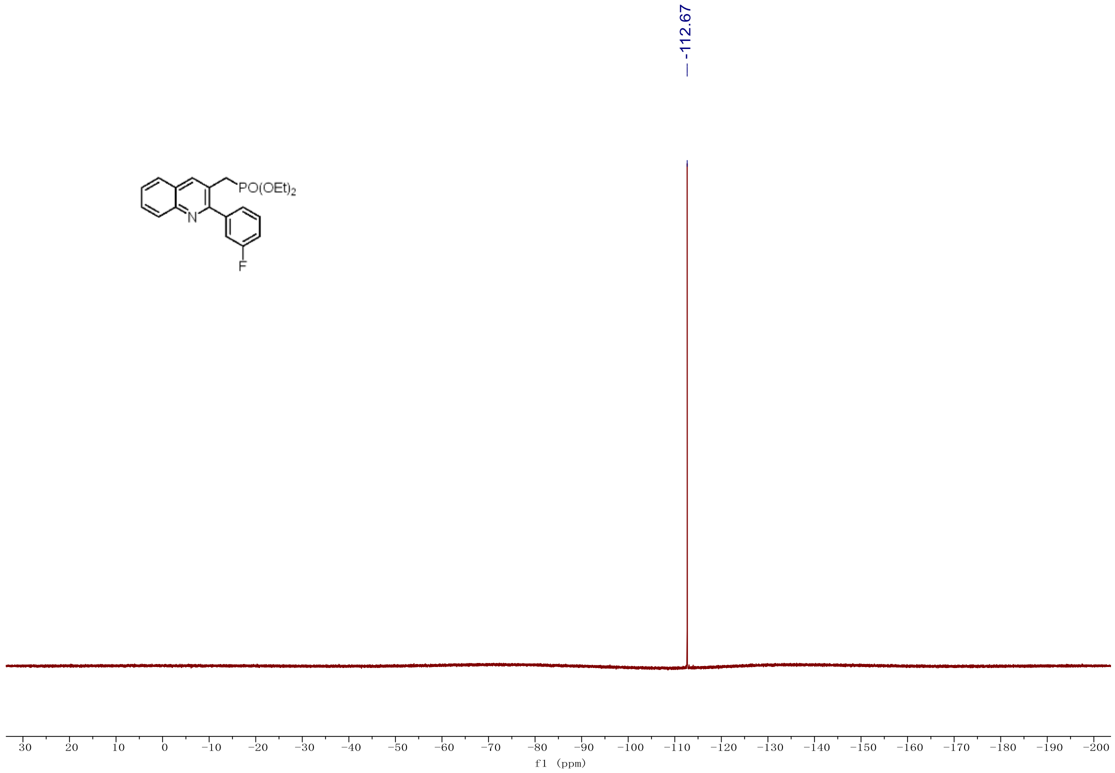

-112.67
PO(OEt)2
N
F
f1 (ppm)

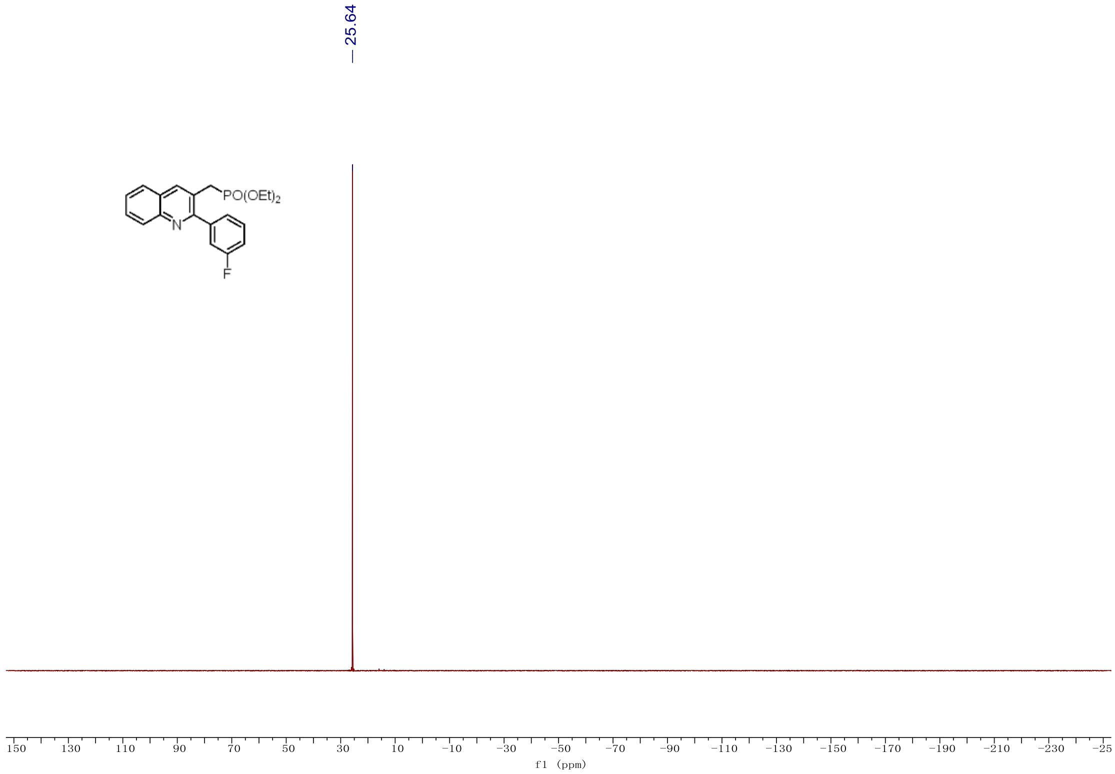

25.64
PO(OEt)2
N
F
f1 (ppm)

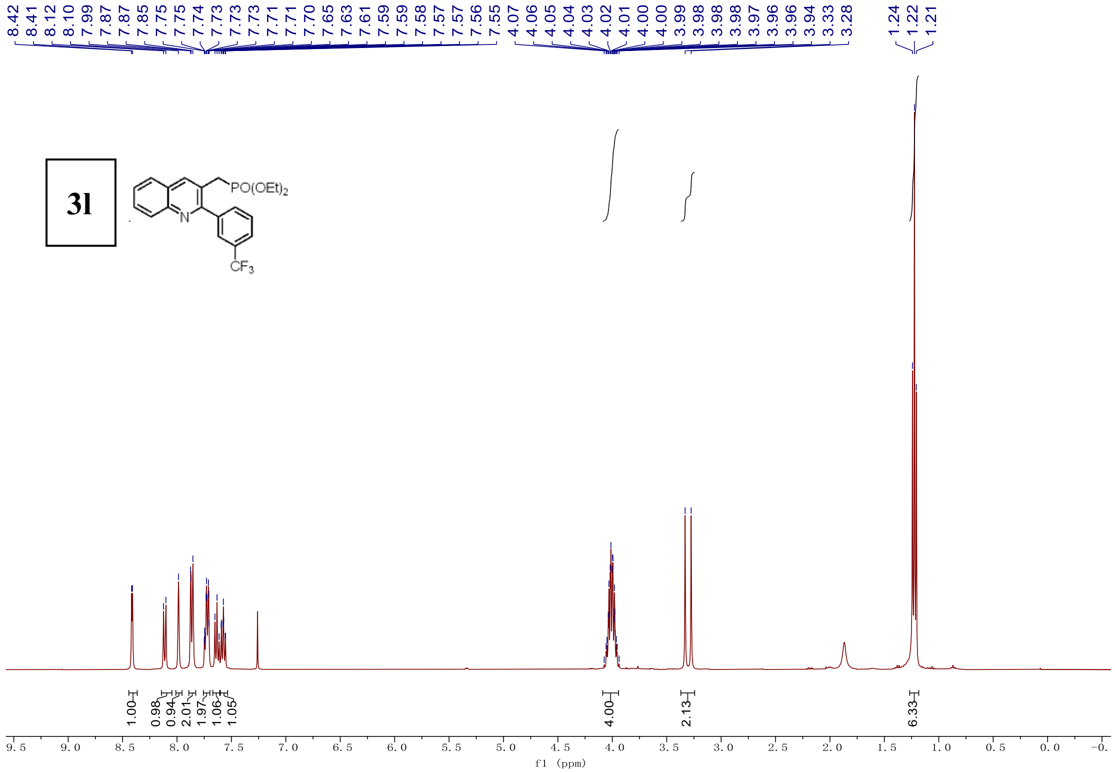

3l
PO(OEt)2
N
CF3
8.42 8.41 8.12 8.10 7.99 7.87 7.87 7.85 7.75 7.75 7.74 7.73 7.73 7.73 7.71 7.71 7.70 7.65 7.63 7.61 7.59 7.59 7.58 7.57 7.57 7.56 7.55 4.07 4.05 4.04 4.03 4.02 4.01 4.00 4.00 3.99 3.98 3.98 3.98 3.97 3.96 3.94 3.33 3.28 1.24 1.22 1.21
1.00 0.98 0.94 2.01 1.97 1.06 1.05 4.00 2.13 6.33
9.5 9.0 8.5 8.0 7.5 7.0 6.5 6.0 5.5 5.0 4.5 4.0 3.5 3.0 2.5 2.0 1.5 1.0 0.5 0.0 -0.
f1 (ppm)

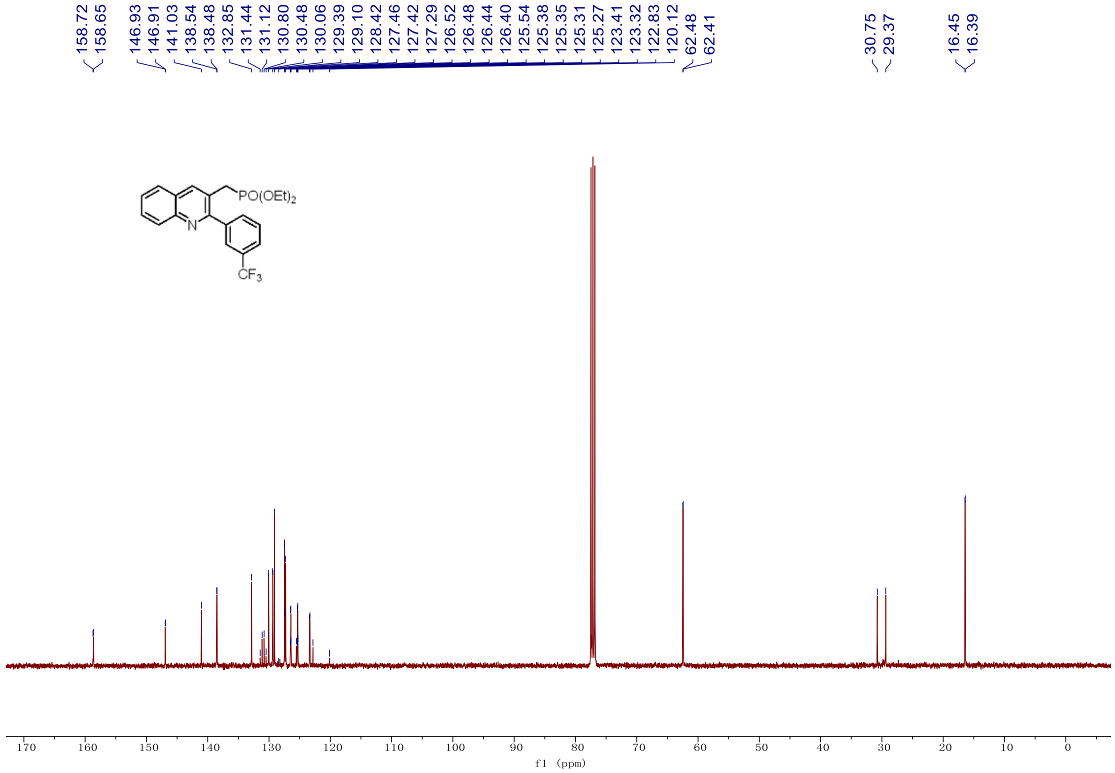

158.72 158.65 146.93 146.91 141.03 138.54 138.48 132.85 131.44 131.12 130.80 130.48 130.06 129.39 129.10 128.42 127.46 127.42 127.29 126.52 126.48 126.44 126.40 125.54 125.38 125.35 125.31 125.27 123.41 123.32 122.83 120.12 62.48 62.41 30.75 29.37 16.45 16.39
PO(OEt)2
N
CF3
170 160 150 140 130 120 110 100 90 80 70 60 50 40 30 20 10 0
f1 (ppm)

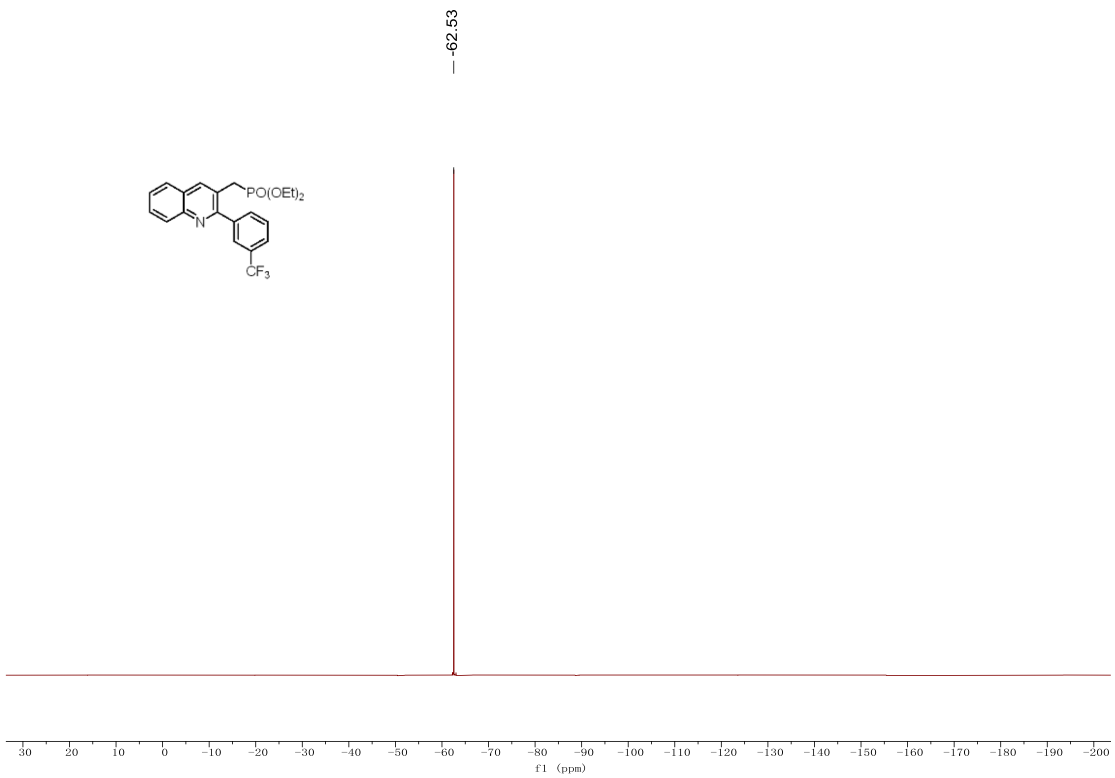
-62.53
PO(OEt)2
N
CF3
30 20 10 0 -10 -20 -30 -40 -50 -60 -70 -80 -90 -100 -110 -120 -130 -140 -150 -160 -170 -180 -190 -200
f1 (ppm)

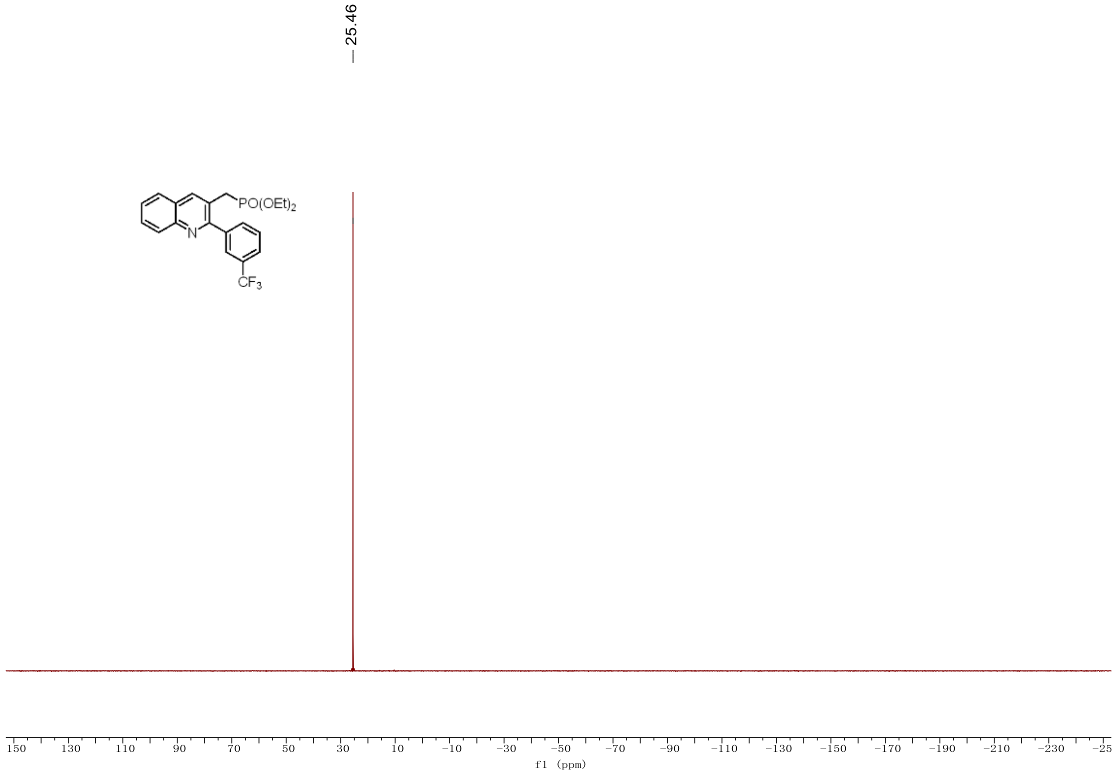
25.46
PO(OEt)2
N
CF3
150 130 110 90 70 50 30 10 -10 -30 -50 -70 -90 -110 -130 -150 -170 -190 -210 -230 -25
f1 (ppm)

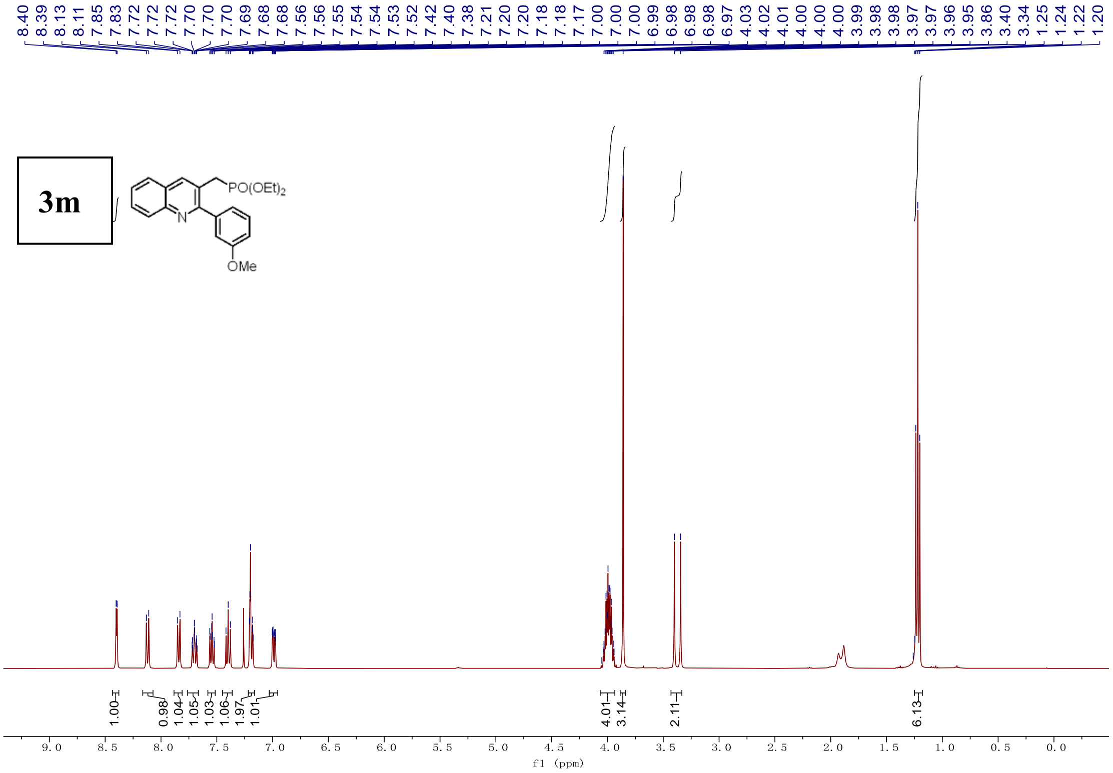
8.40 8.39 8.13 8.11 7.85 7.83 7.72 7.72 7.70 7.70 7.70 7.69 7.68 7.68 7.56 7.56 7.55 7.54 7.54 7.53 7.52 7.42 7.40 7.38 7.21 7.20 7.18 7.18 7.17 7.00 7.00 6.99 6.98 6.98 6.98 6.97 4.03 4.02 4.01 4.00 4.00 3.99 3.98 3.98 3.97 3.97 3.96 3.95 3.86 3.40 3.34 1.25 1.24 1.22 1.20
3m
PO(OEt)2
N
OMe
1.00 0.98 1.04 1.05 1.03 1.06 1.97 1.01 4.01 3.14 2.11 6.13
f1 (ppm)

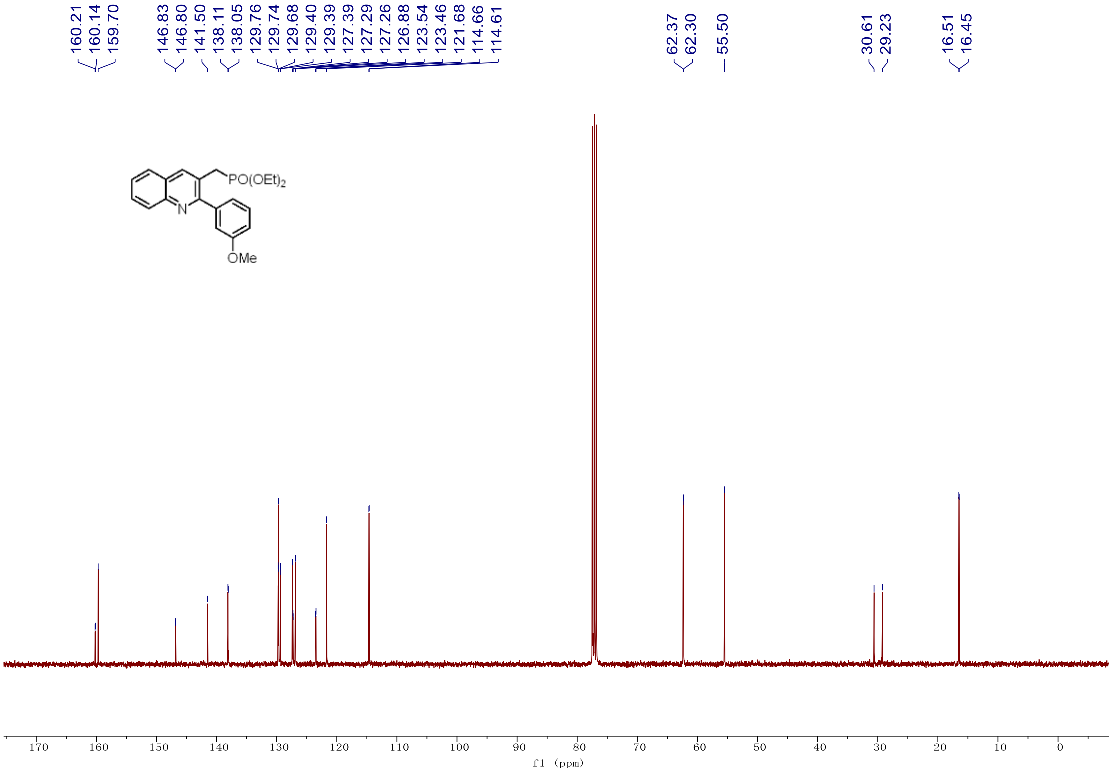
160.21 160.14 159.70 146.83 146.80 141.50 138.11 138.05 129.76 129.74 129.68 129.40 129.39 127.39 127.29 127.26 126.88 123.54 123.46 121.68 114.66 114.61 62.37 62.30 55.50 30.61 29.23 16.51 16.45
PO(OEt)2
N
OMe
f1 (ppm)

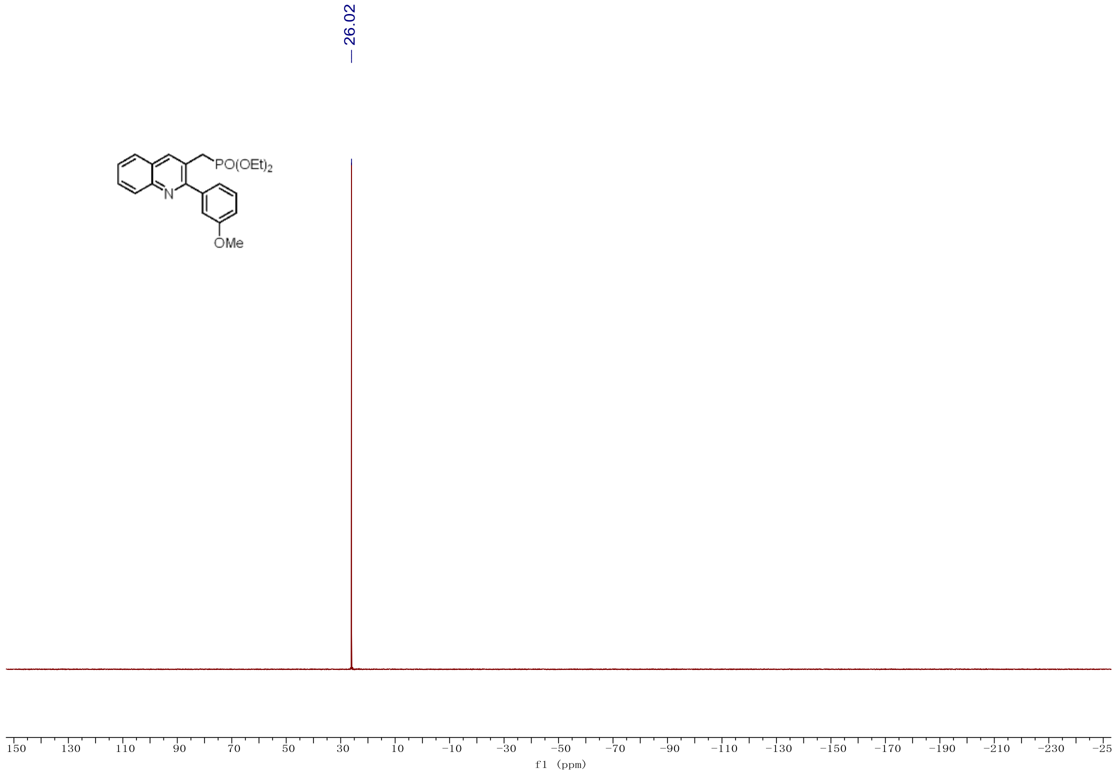
26.02
PO(OEt)2
N
OMe
150 130 110 90 70 50 30 10 −10 −30 −50 −70 −90 −110 −130 −150 −170 −190 −210 −230 −25
f1 (ppm)

3n

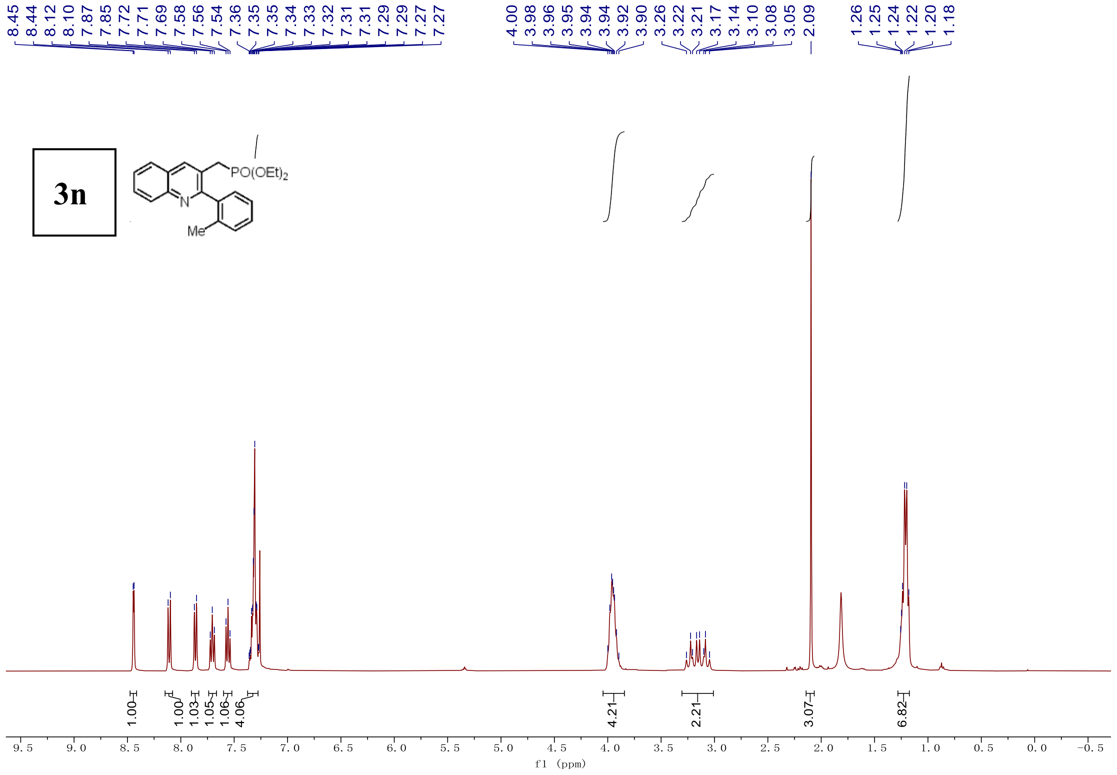
8.45 8.44 8.12 8.10 7.87 7.85 7.72 7.71 7.69 7.58 7.56 7.54 7.36 7.35 7.35 7.34 7.33 7.32 7.31 7.31 7.29 7.29 7.27 7.27
4.00 3.98 3.96 3.95 3.94 3.94 3.92 3.90 3.26 3.22 3.21 3.17 3.14 3.10 3.08 3.05 2.09
1.26 1.25 1.24 1.22 1.20 1.18
PO(OEt)2
N
Me
1.00 1.00 1.03 1.05 1.06 4.06 4.21 2.21 3.07 6.82
9.5 9.0 8.5 8.0 7.5 7.0 6.5 6.0 5.5 5.0 4.5 4.0 3.5 3.0 2.5 2.0 1.5 1.0 0.5 0.0 −0.5
f1 (ppm)

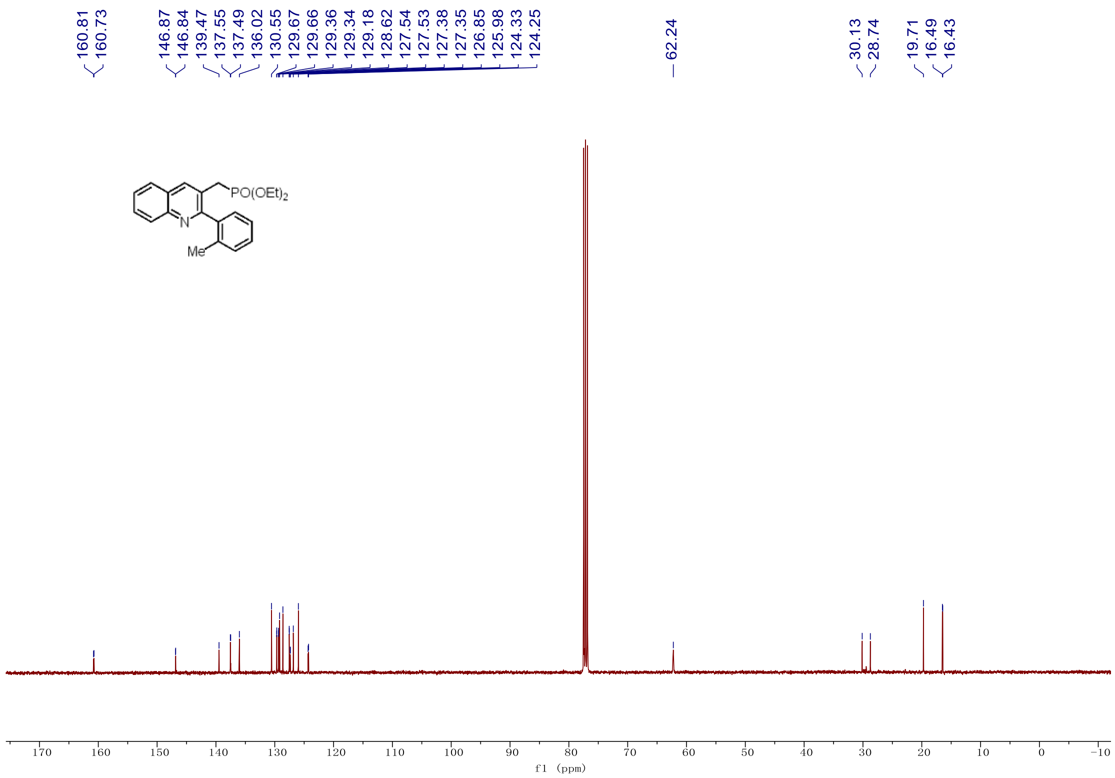
160.81
160.73
146.87
146.84
139.47
137.55
137.49
136.02
130.55
129.67
129.66
129.36
129.34
129.18
128.62
127.54
127.53
127.38
127.35
126.85
125.98
124.33
124.25
62.24
30.13
28.74
19.71
16.49
16.43
PO(OEt)2
N
Me
f1 (ppm)

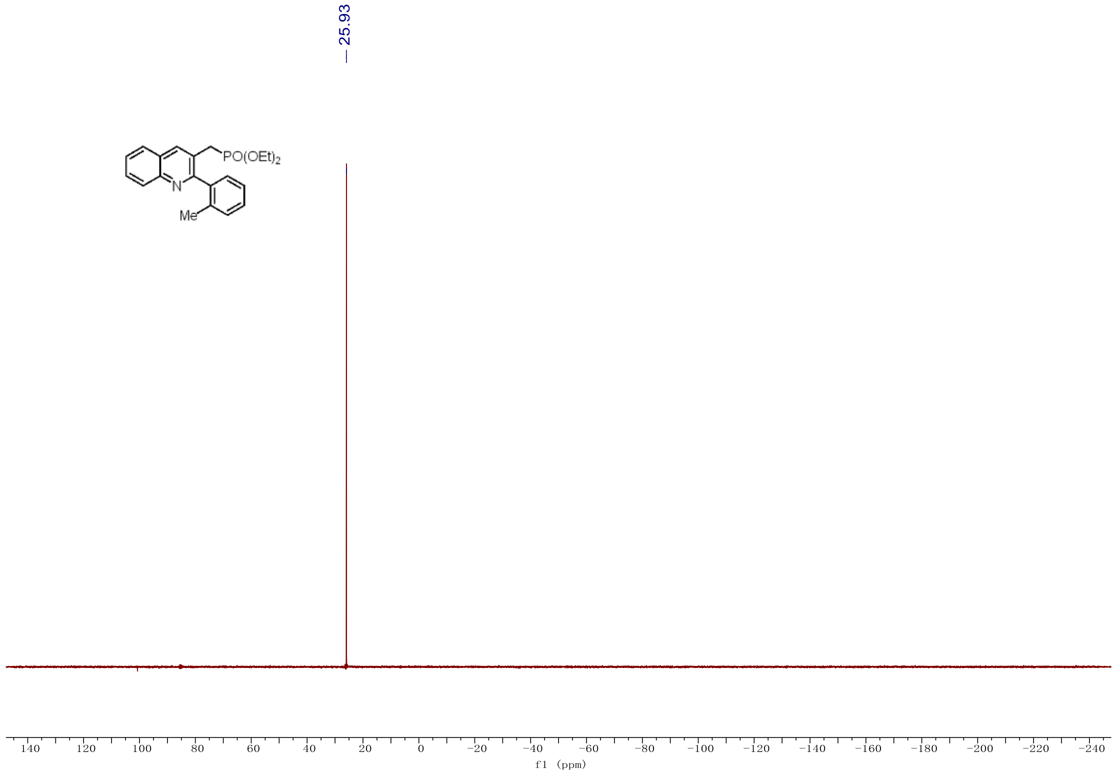
25.93
PO(OEt)2
N
Me
f1 (ppm)

3o

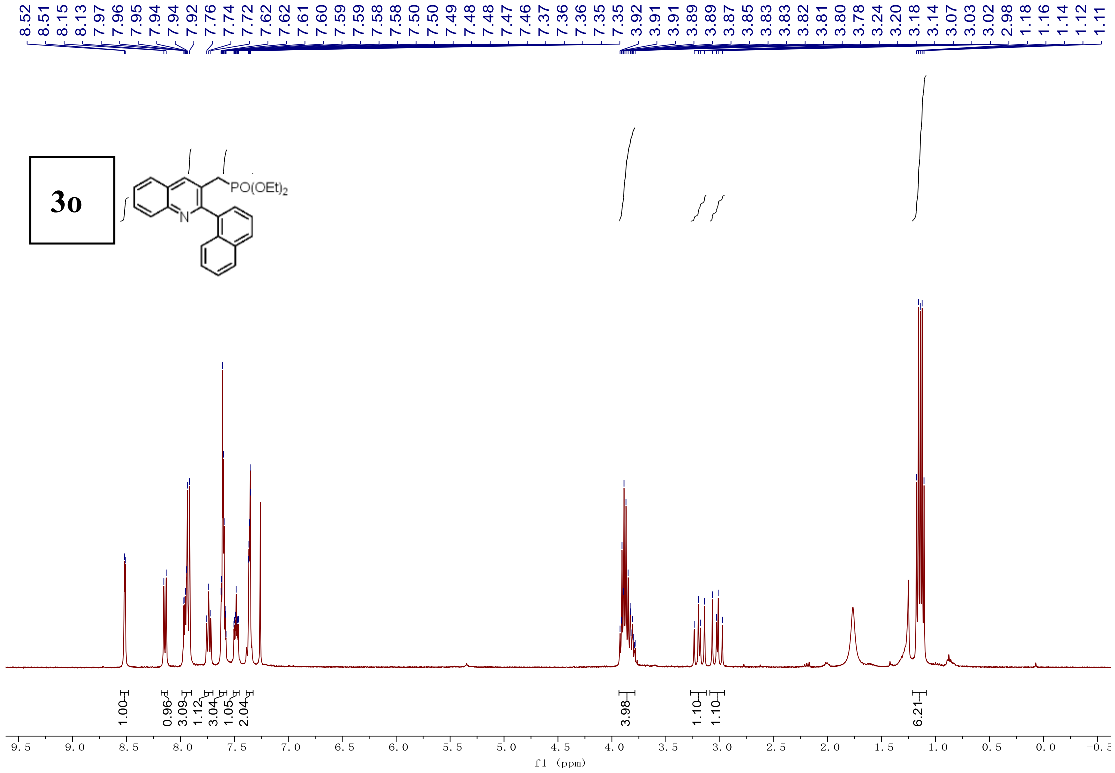
8.52 8.51 8.15 8.13 7.97 7.96 7.95 7.94 7.94 7.92 7.76 7.74 7.72 7.62 7.62 7.61 7.60 7.59 7.59 7.58 7.58 7.50 7.50 7.49 7.48 7.48 7.47 7.46 7.37 7.36 7.36 7.35 7.35 3.92 3.91 3.91 3.89 3.89 3.87 3.85 3.83 3.83 3.82 3.81 3.80 3.78 3.24 3.20 3.18 3.14 3.07 3.03 3.02 2.98 1.18 1.16 1.14 1.12 1.11
PO(OEt)2
N
1.00 0.96 3.09 1.12 3.04 1.05 2.04 3.98 1.10 1.10 6.21
f1 (ppm)

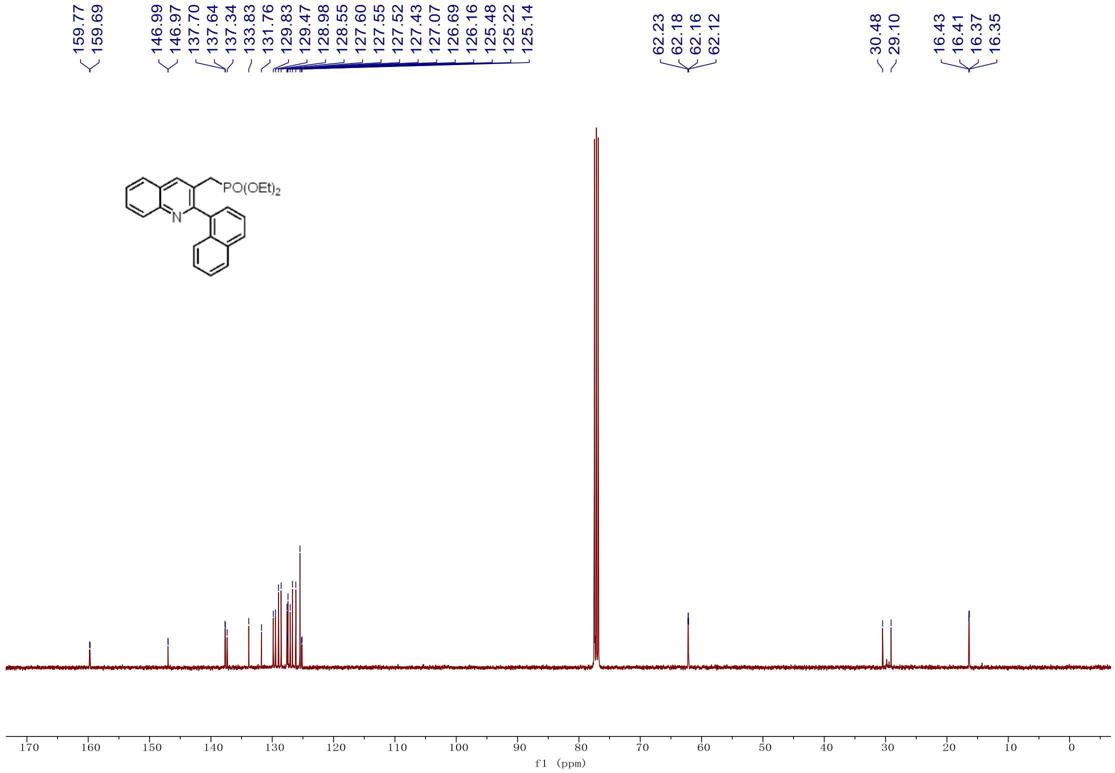
159.77 159.69 146.99 146.97 137.70 137.64 137.34 133.83 131.76 129.83 129.47 128.98 128.55 127.60 127.55 127.52 127.43 127.07 126.69 126.16 125.48 125.22 125.14 62.23 62.18 62.16 62.12 30.48 29.10 16.43 16.41 16.37 16.35
PO(OEt)2
N
f1 (ppm)

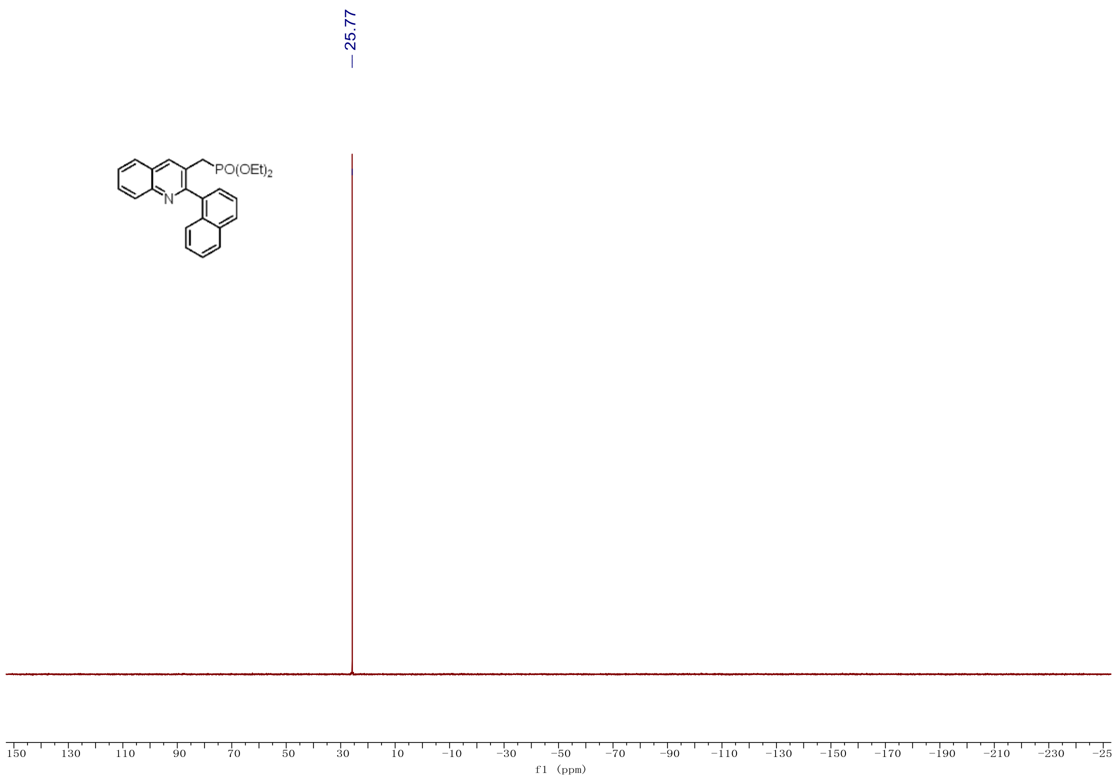
25.77
PO(OEt)2
N
f1 (ppm)

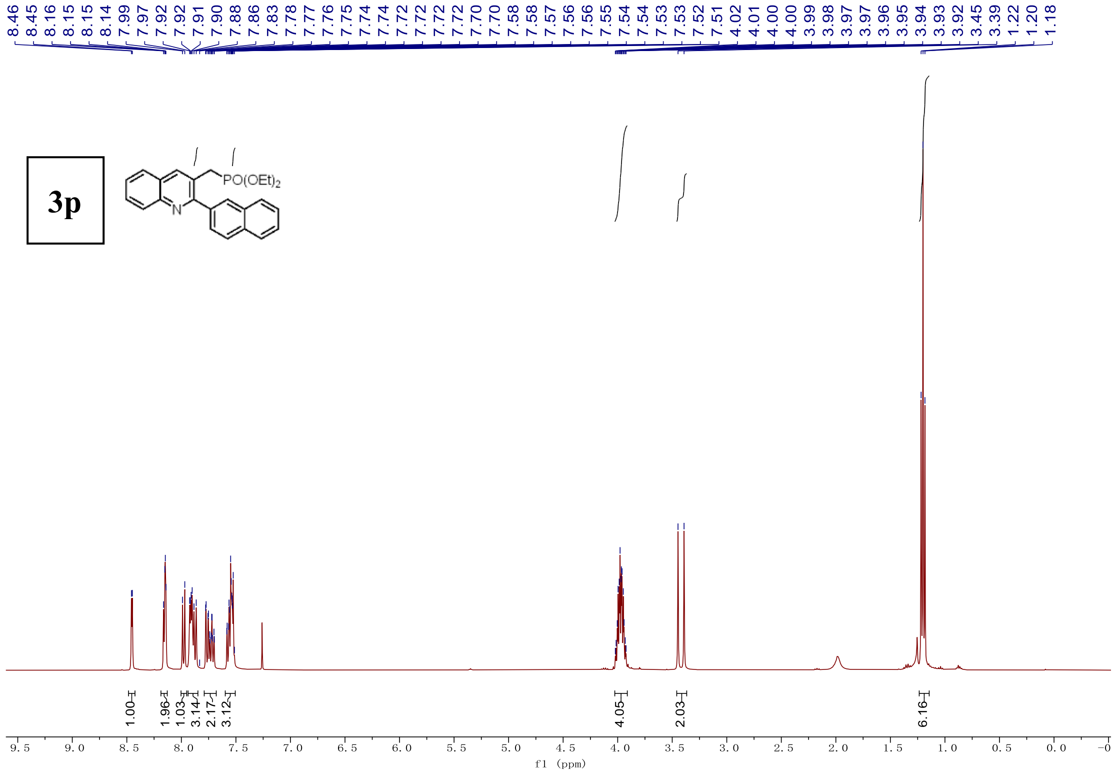
8.46
8.45
8.16
8.15
8.15
8.14
7.99
7.97
7.92
7.92
7.91
7.90
7.88
7.86
7.83
7.78
7.77
7.76
7.75
7.74
7.74
7.72
7.72
7.72
7.72
7.70
7.70
7.58
7.57
7.56
7.56
7.55
7.54
7.54
7.53
7.53
7.52
7.51
4.02
4.01
4.00
4.00
3.99
3.98
3.97
3.97
3.96
3.95
3.94
3.93
3.92
3.45
3.39
1.22
1.20
1.18
3p
PO(OEt)2
N
1.00
1.96
1.03
3.14
2.17
3.12
4.05
2.03
6.16
f1 (ppm)

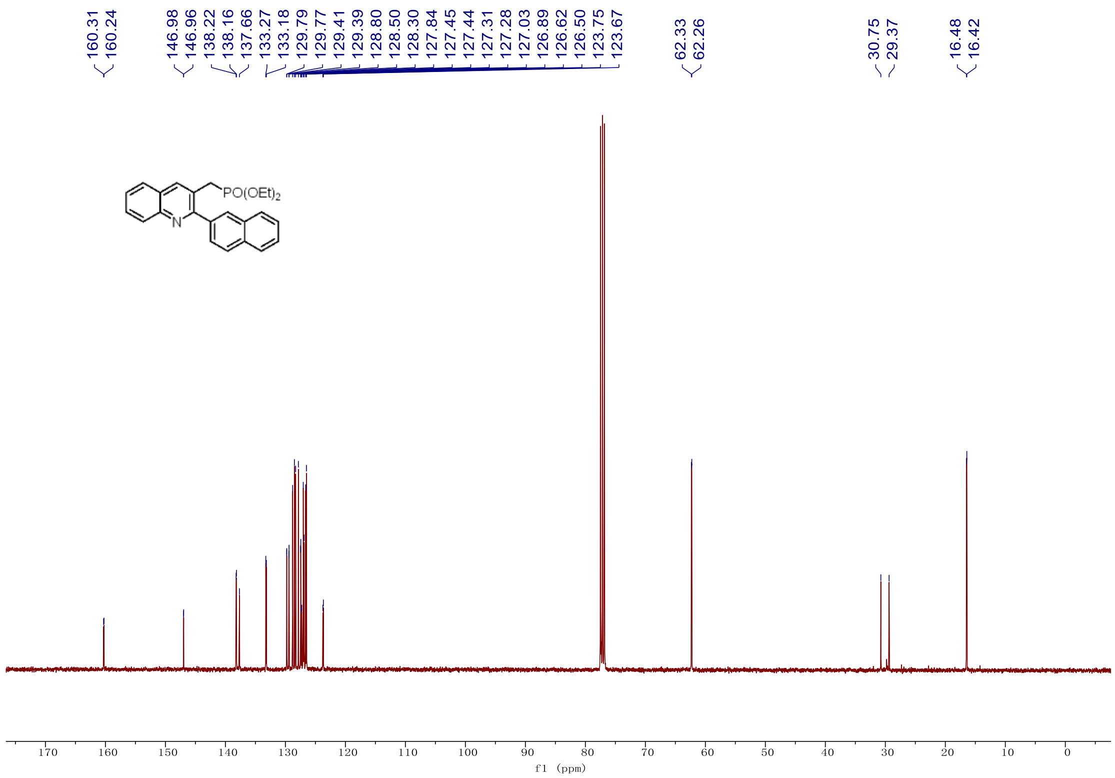
160.31
160.24
146.98
146.96
138.22
138.16
137.66
133.27
133.18
129.79
129.77
129.41
129.39
128.80
128.50
128.30
127.84
127.45
127.44
127.31
127.28
127.03
126.89
126.62
126.50
123.75
123.67
62.33
62.26
30.75
29.37
16.48
16.42
PO(OEt)2
N
f1 (ppm)

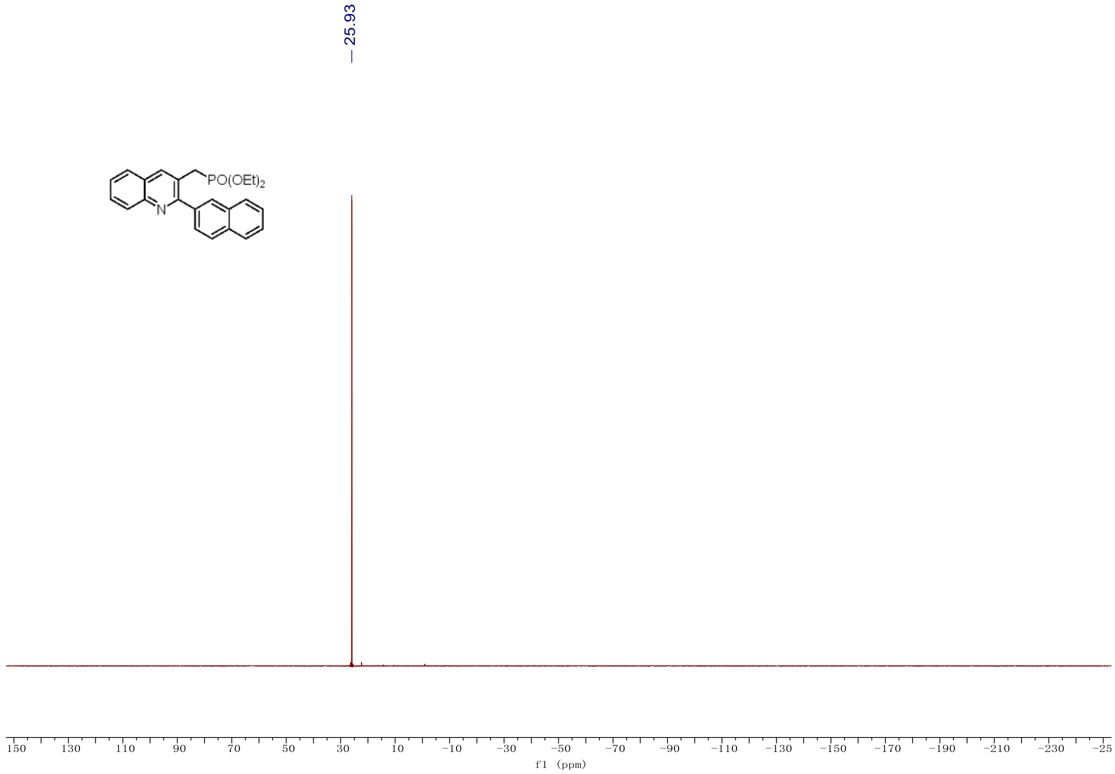
25.93
PO(OEt)2
N
f1 (ppm)

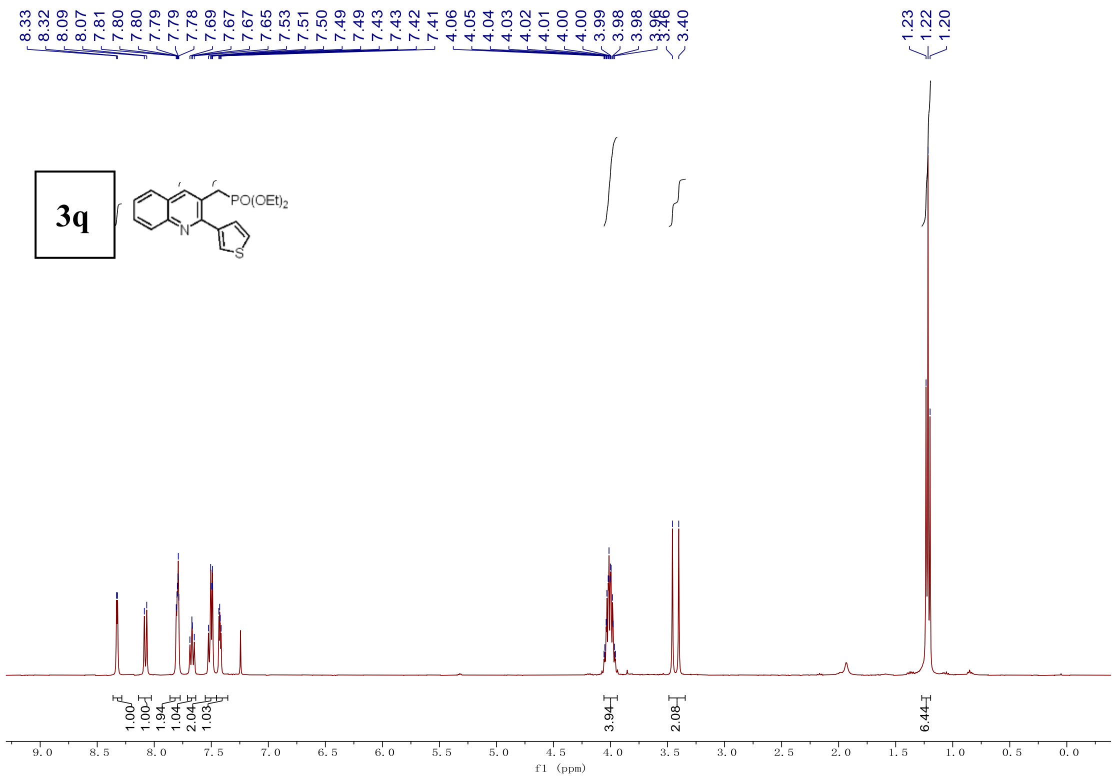
8.33
8.32
8.09
8.07
7.81
7.80
7.80
7.79
7.79
7.78
7.69
7.67
7.67
7.65
7.53
7.51
7.50
7.49
7.49
7.43
7.43
7.42
7.41
4.06
4.05
4.04
4.03
4.02
4.01
4.00
4.00
3.99
3.98
3.98
3.96
3.46
3.40
1.23
1.22
1.20
3q
PO(OEt)2
N
S
1.00
1.00
1.94
1.04
2.04
1.03
3.94
2.08
6.44
f1 (ppm)

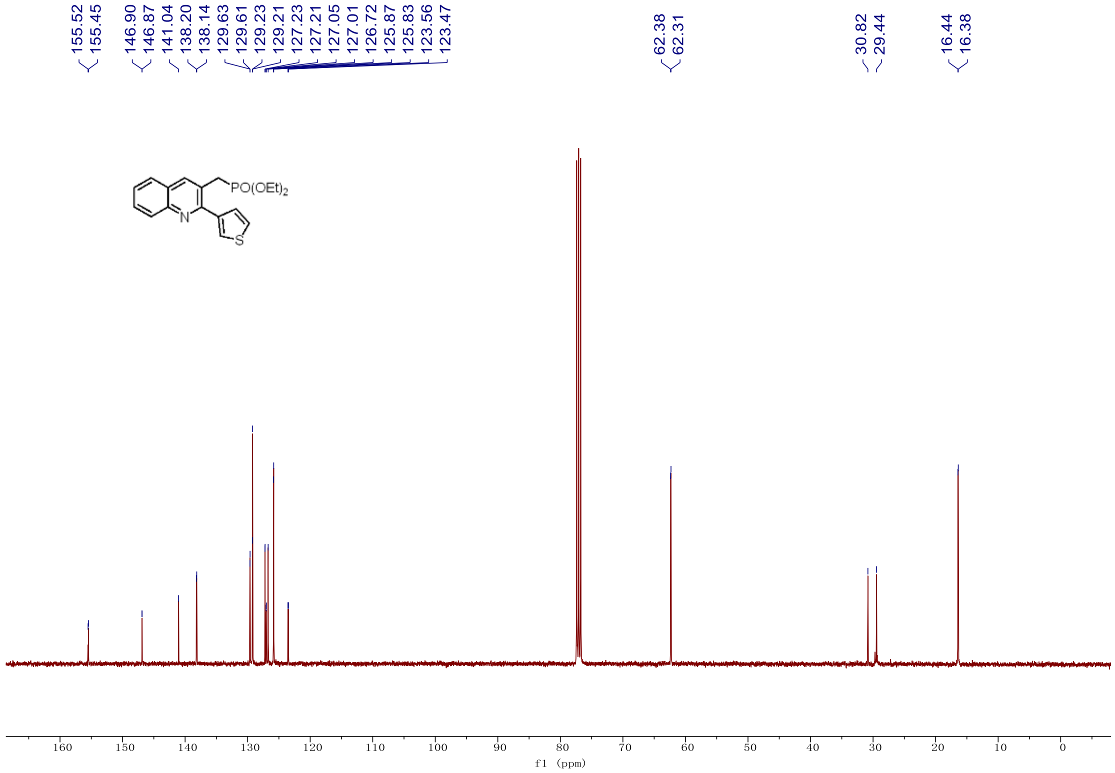
155.52
155.45
146.90
146.87
141.04
138.20
138.14
129.63
129.61
129.23
129.21
127.23
127.21
127.05
127.01
126.72
125.87
125.83
123.56
123.47
62.38
62.31
30.82
29.44
16.44
16.38
PO(OEt)2
N
S
f1 (ppm)

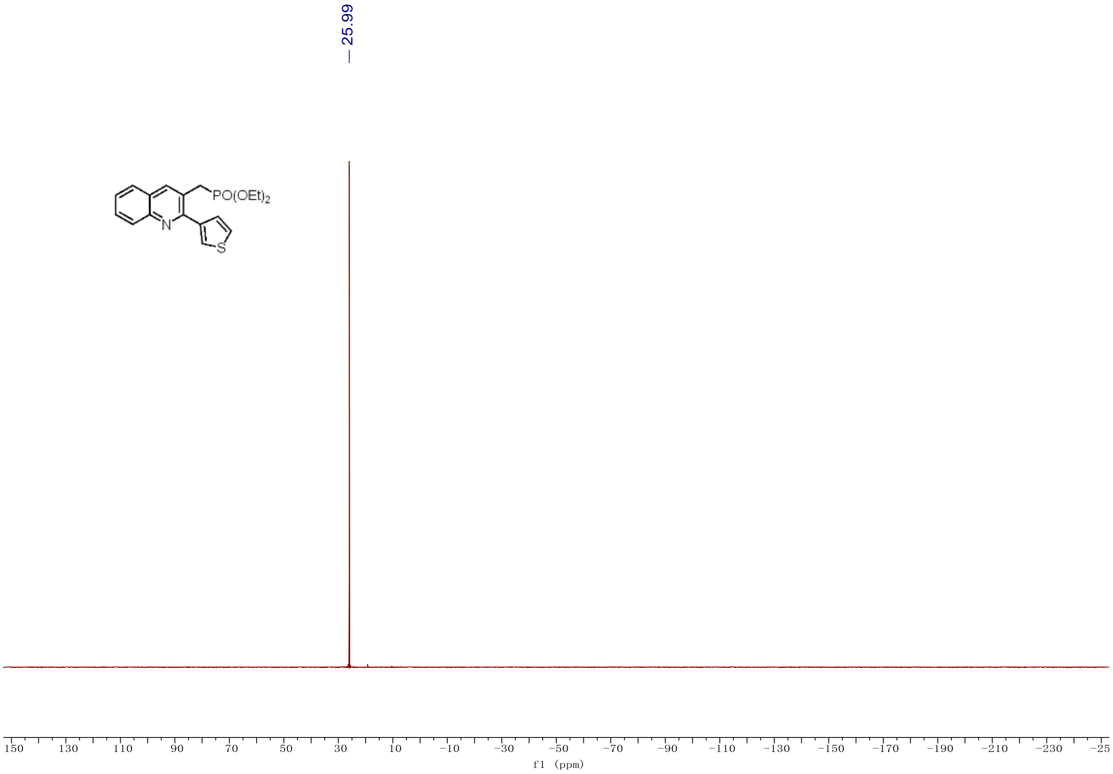
25.99
PO(OEt)2
N
S
f1 (ppm)

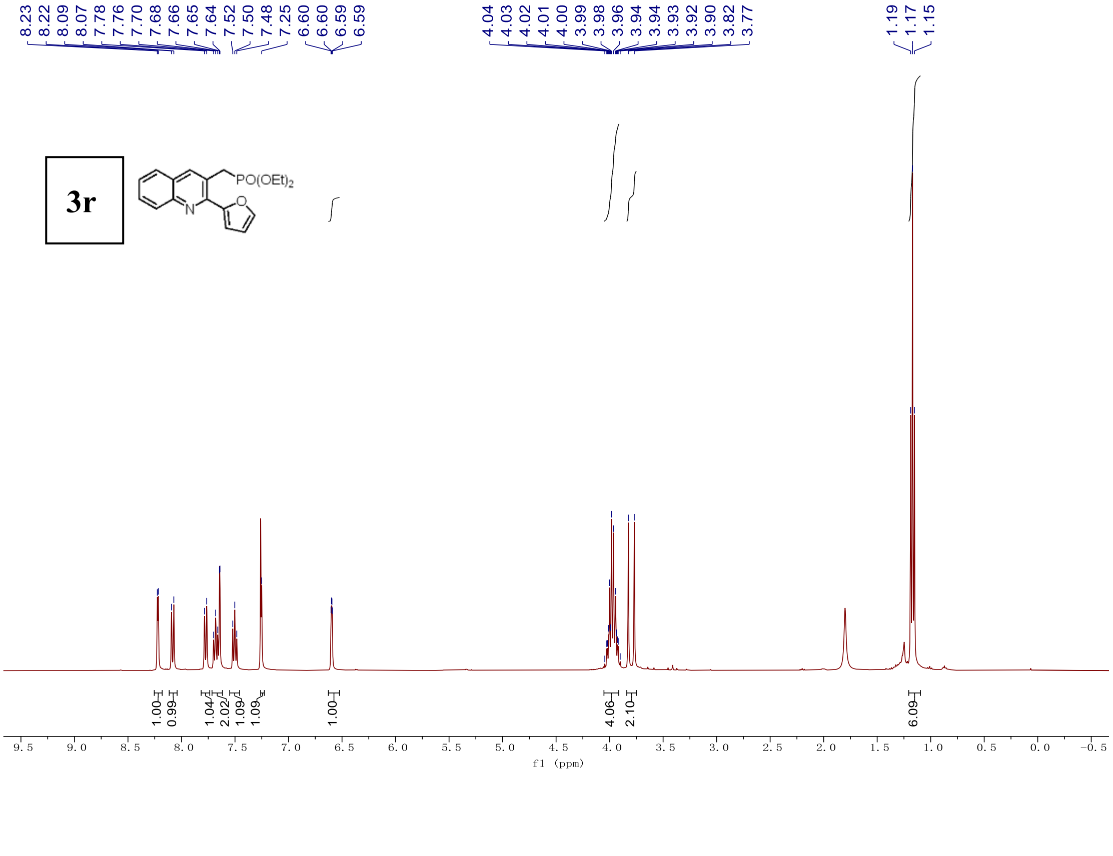
8.23
8.22
8.09
8.07
7.78
7.76
7.70
7.68
7.66
7.65
7.64
7.52
7.50
7.48
7.25
6.60
6.60
6.59
6.59
4.04
4.03
4.02
4.01
4.00
3.99
3.98
3.96
3.94
3.93
3.92
3.90
3.82
3.77
1.19
1.17
1.15
3r
PO(OEt)2
N
O
1.00
0.99
1.04
2.02
1.09
1.09
1.00
4.06
2.10
6.09
f1 (ppm)

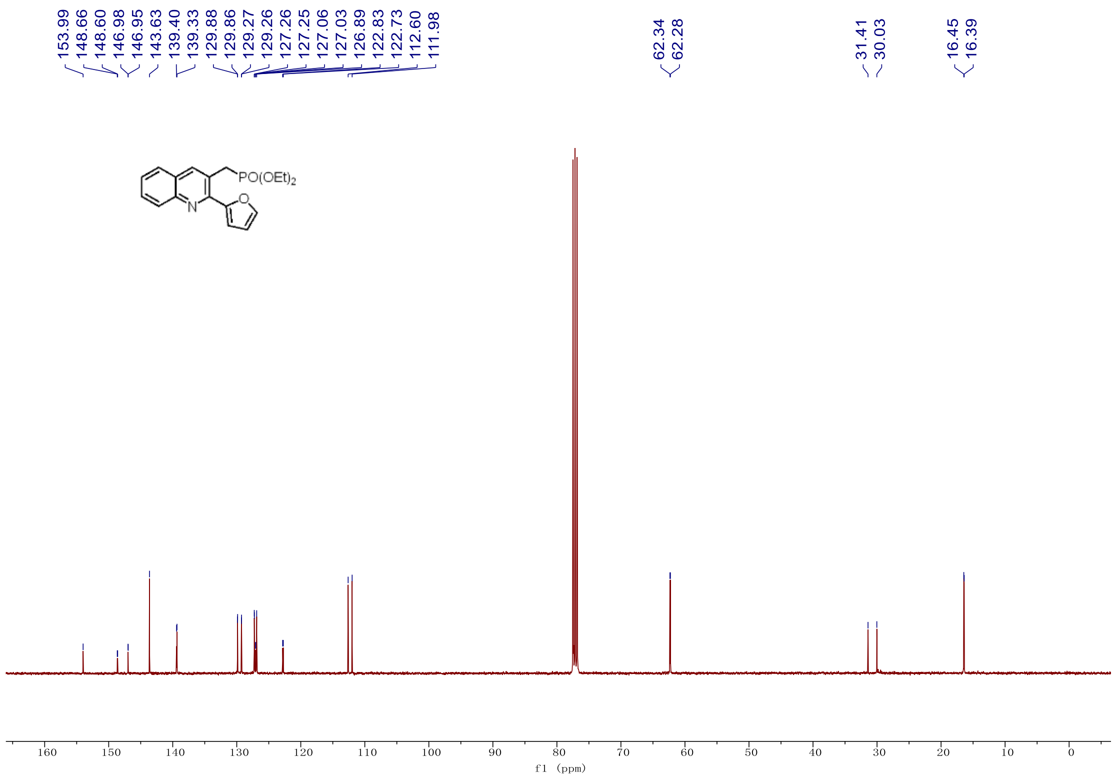
153.99
148.66
148.60
146.98
146.95
143.63
139.40
139.33
129.88
129.86
129.27
129.26
127.26
127.25
127.06
127.03
126.89
122.83
122.73
112.60
111.98
62.34
62.28
31.41
30.03
16.45
16.39
PO(OEt)2
N
O
f1 (ppm)

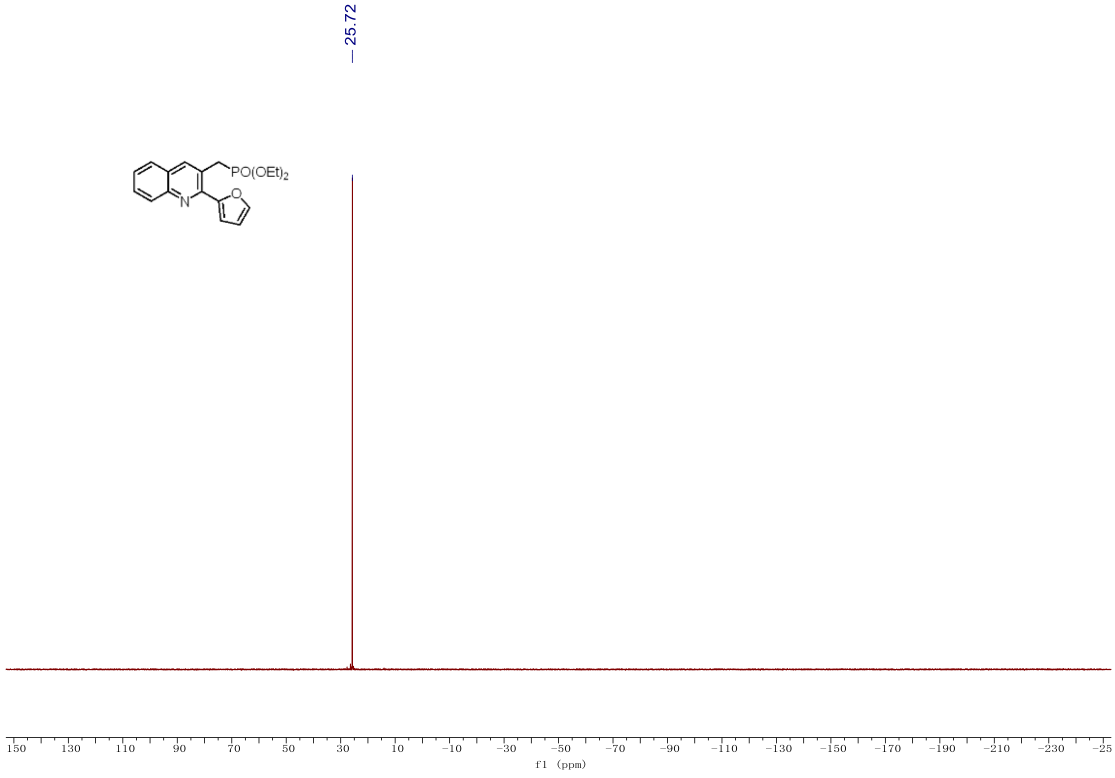
25.72
PO(OEt)2
N
O
f1 (ppm)

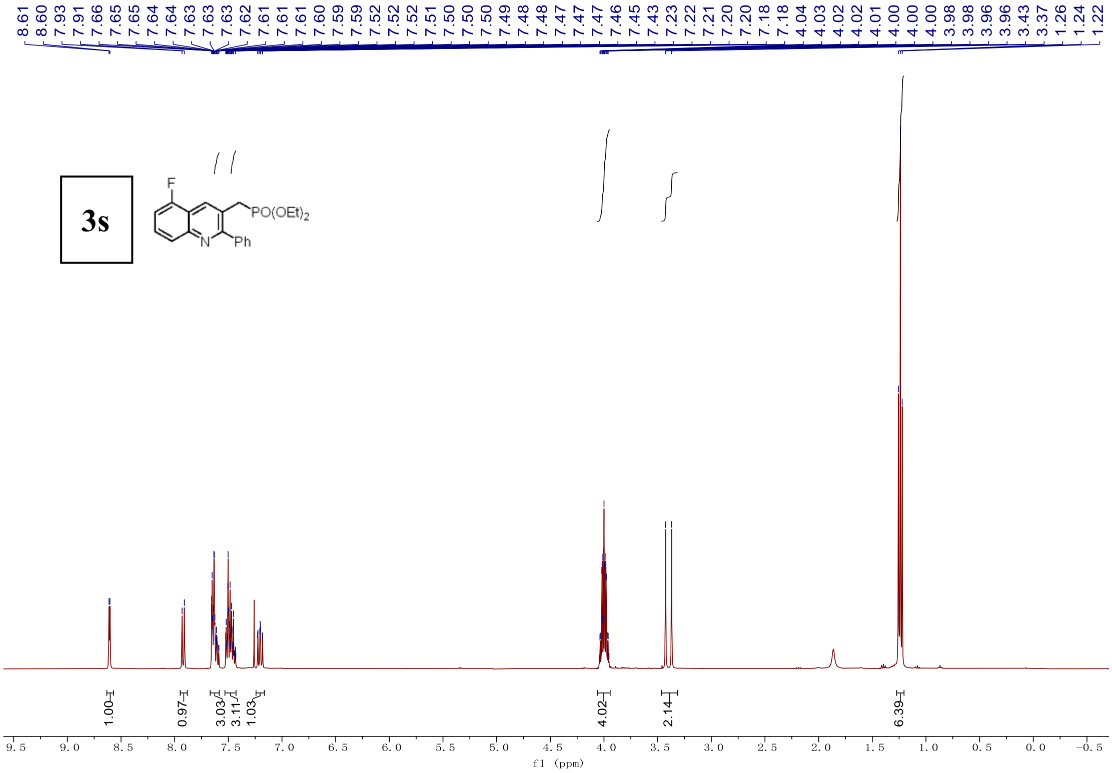
8.61 8.60 7.93 7.91 7.66 7.65 7.65 7.64 7.64 7.63 7.63 7.63 7.62 7.61 7.61 7.60 7.59 7.52 7.52 7.52 7.51 7.50 7.50 7.49 7.48 7.48 7.47 7.47 7.46 7.45 7.43 7.23 7.22 7.21 7.20 7.20 7.18 4.04 4.03 4.02 4.02 4.01 4.00 4.00 4.00 3.98 3.98 3.96 3.96 3.43 3.37 1.26 1.24 1.22
3s
F
PO(OEt)2
N
Ph
1.00
0.97
3.03
3.11
1.03
4.02
2.14
6.39
f1 (ppm)

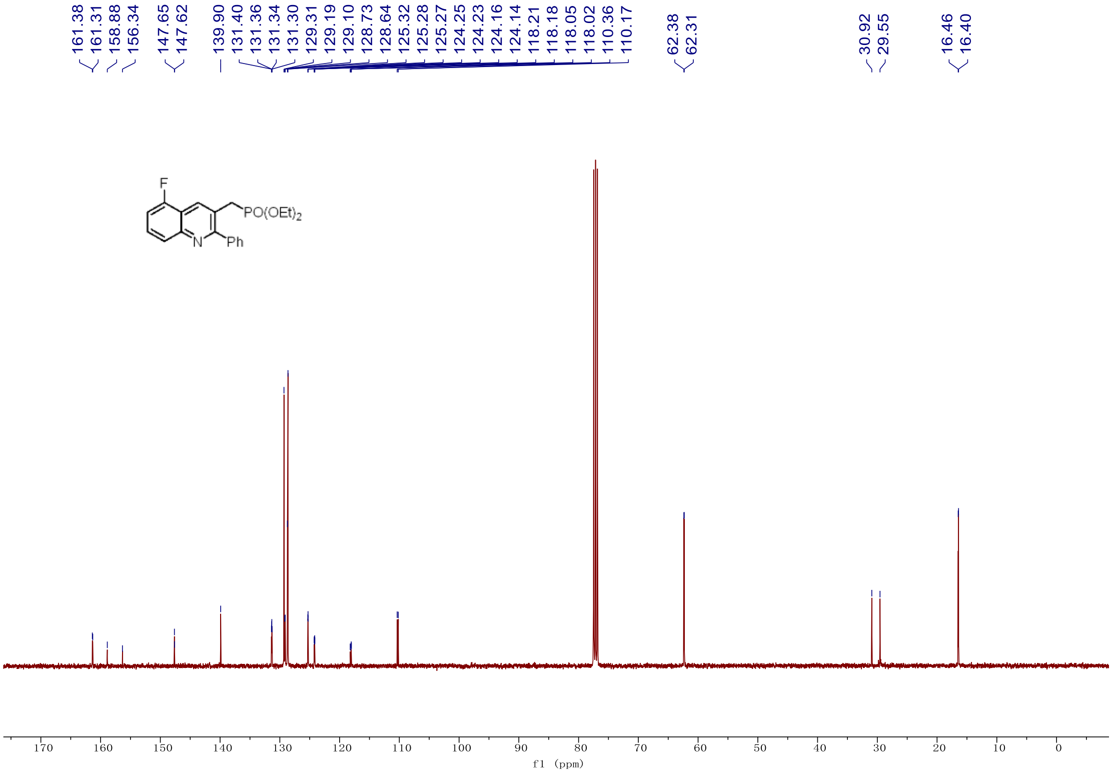
161.38 161.31 158.88 156.34 147.65 147.62 139.90 131.40 131.36 131.34 131.30 129.31 129.19 129.10 128.73 128.64 125.32 125.27 124.25 124.23 124.16 124.14 118.21 118.18 118.05 118.02 110.36 110.17 62.38 62.31 30.92 29.55 16.46 16.40
F
PO(OEt)2
N
Ph
f1 (ppm)

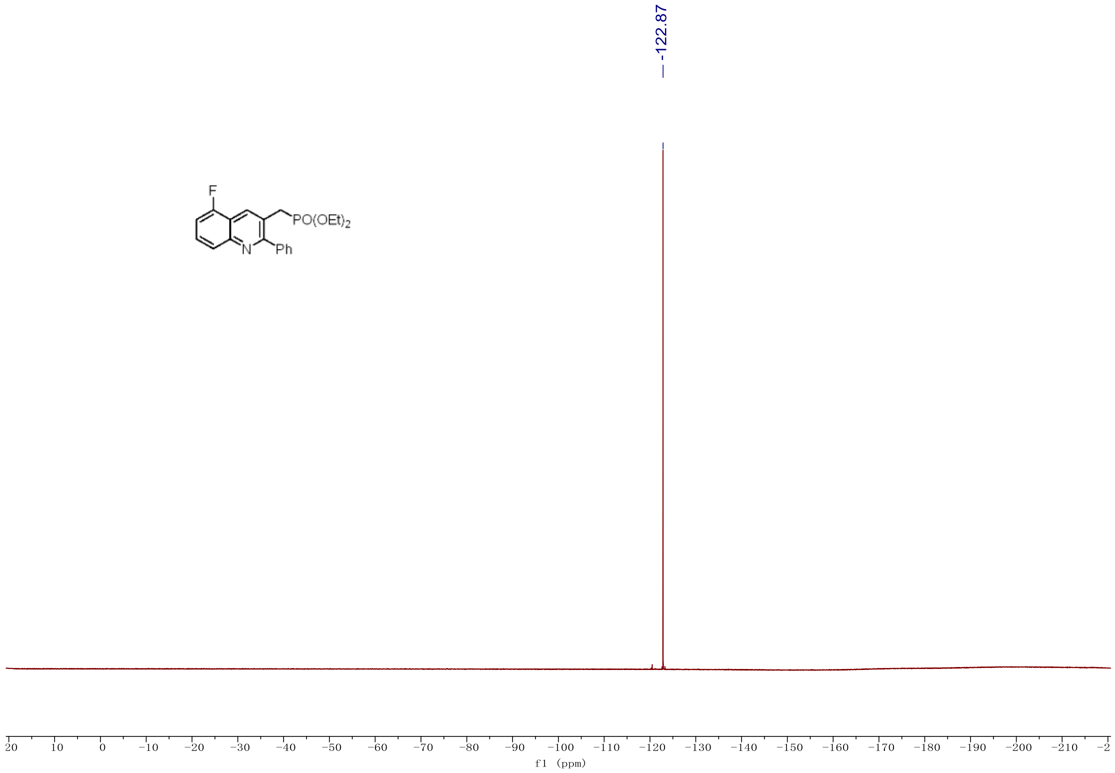
-122.87
F
PO(OEt)2
N
Ph
f1 (ppm)

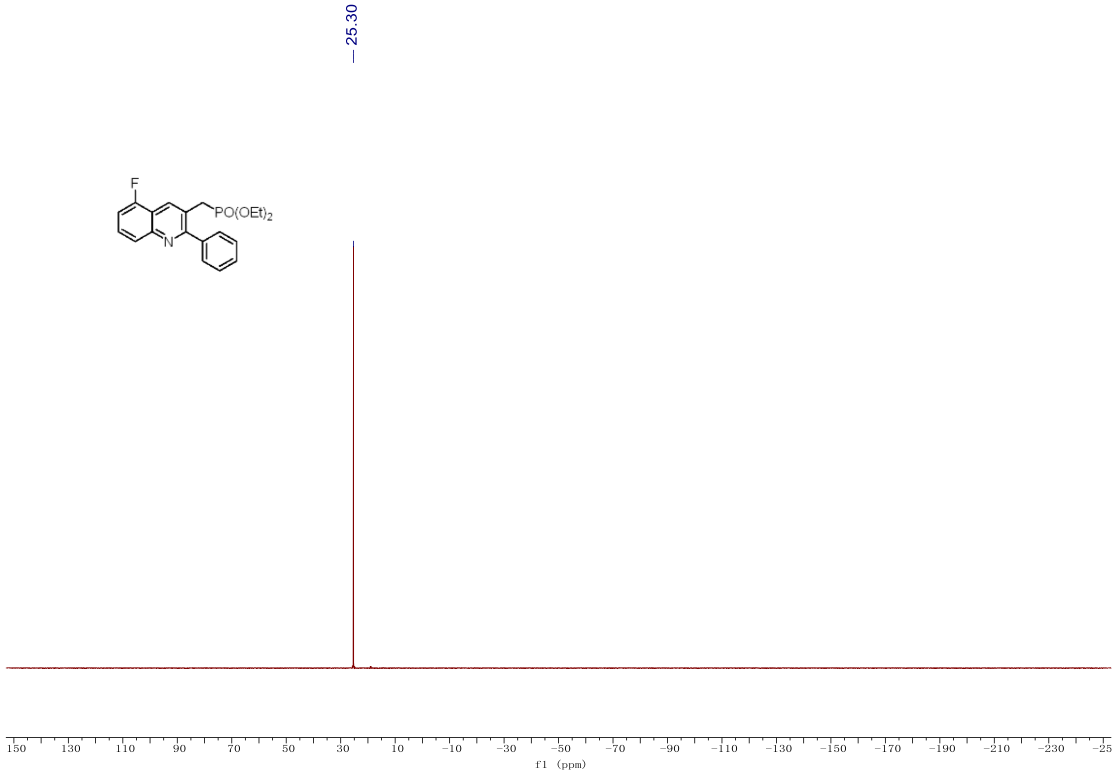
25.30
F
PO(OEt)2
N
f1 (ppm)

3t

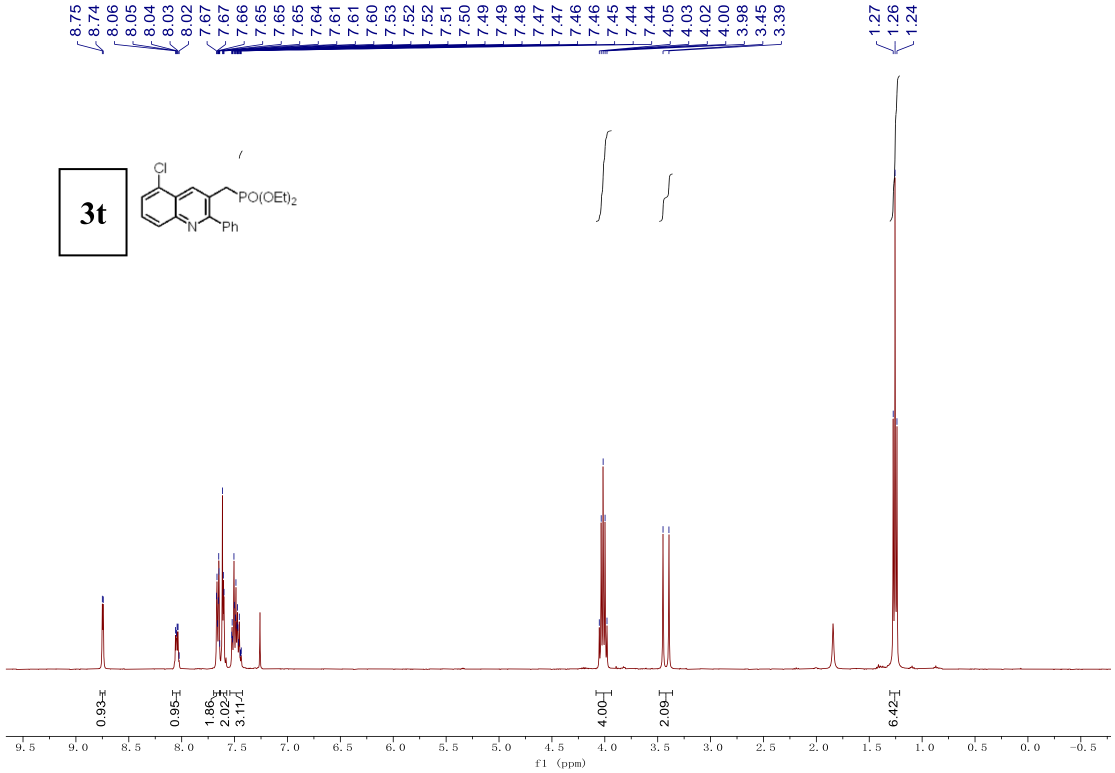

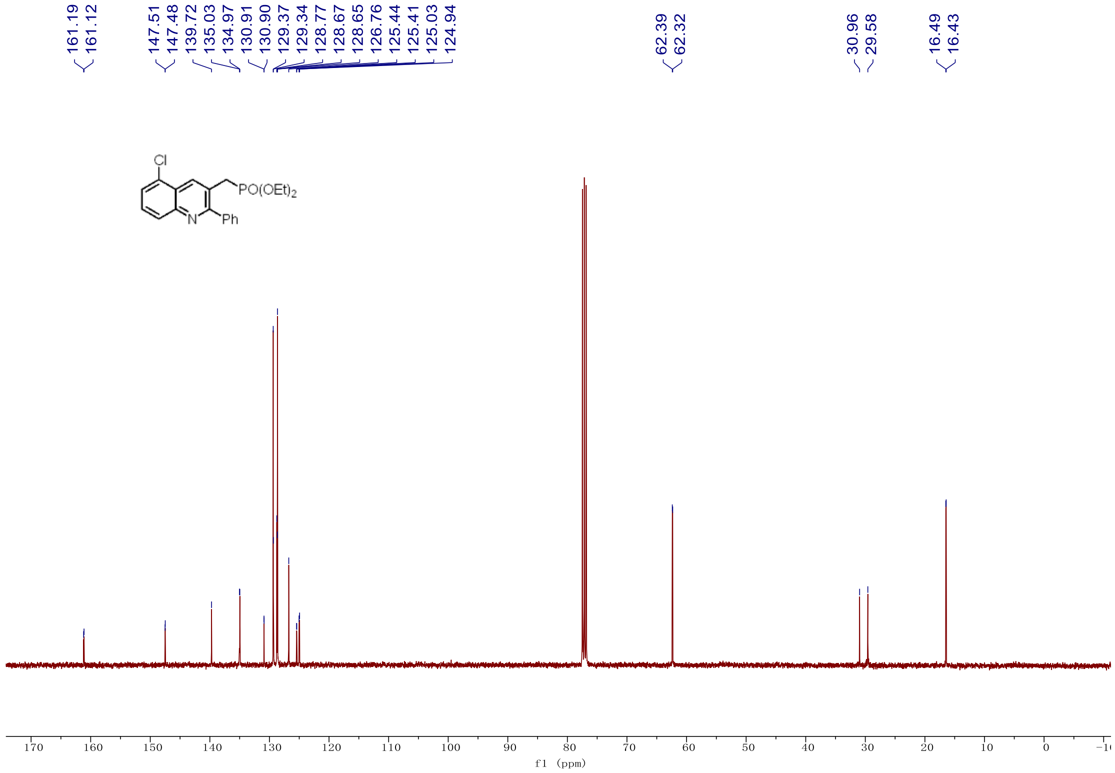

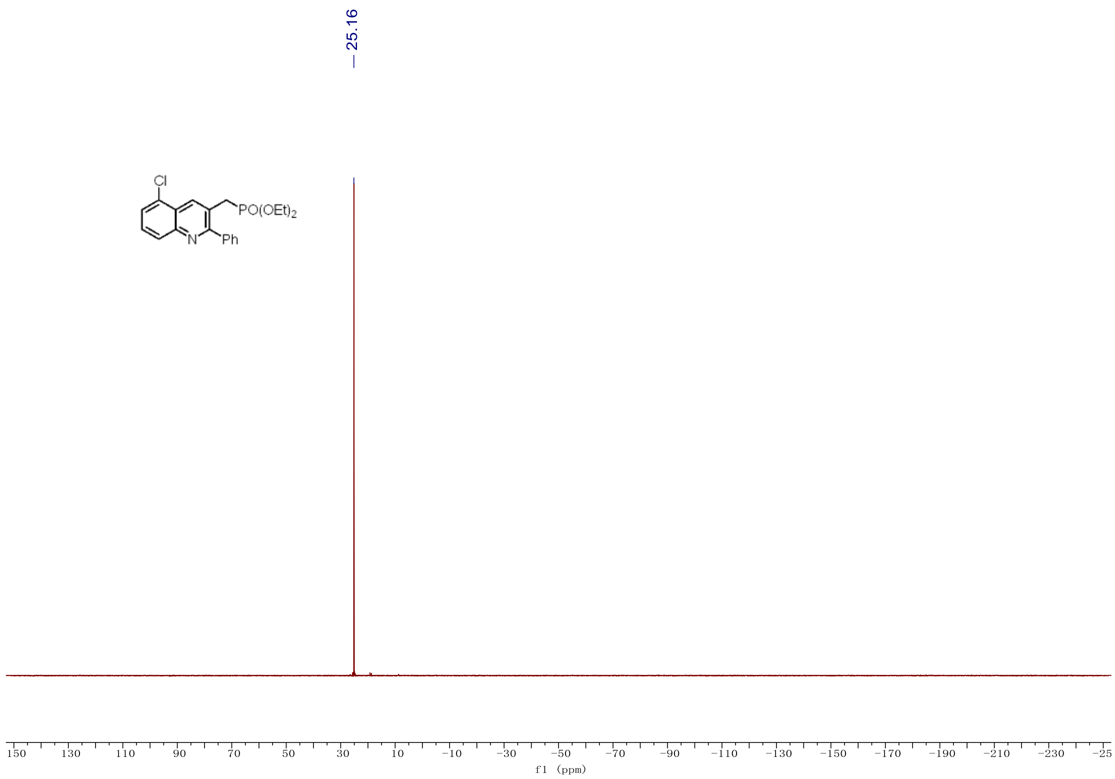
25.16
Cl
PO(OEt)2
N
Ph
f1 (ppm)

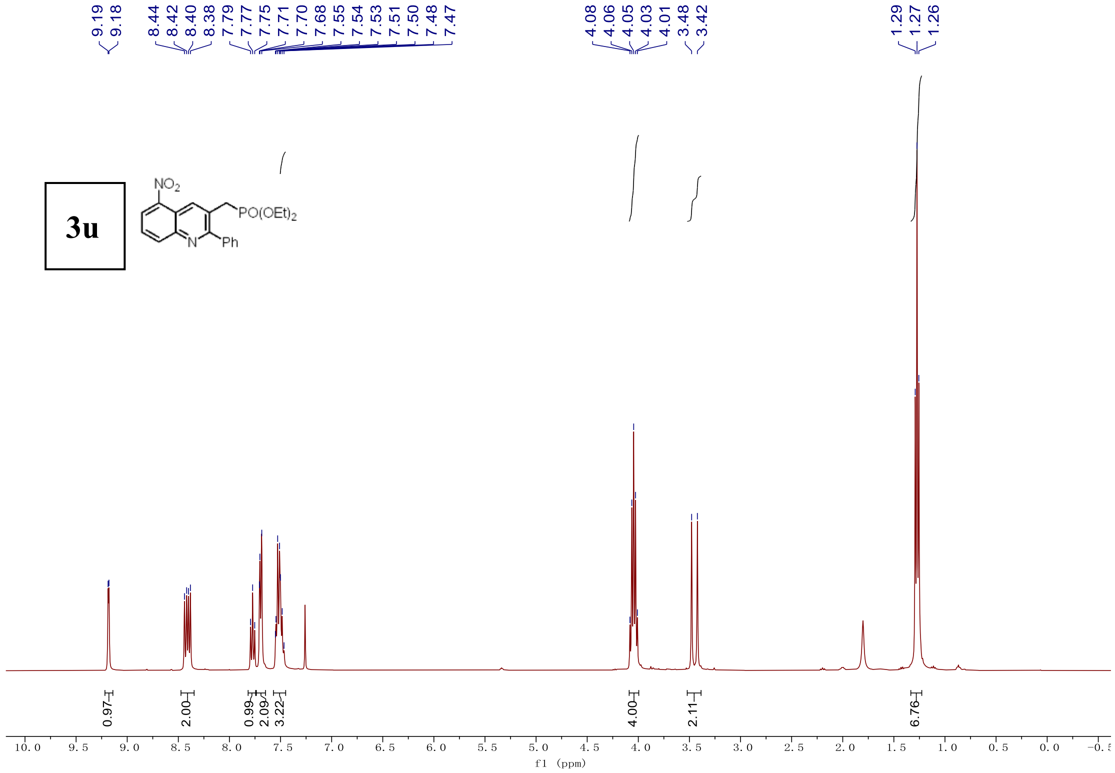
9.19
9.18
8.44
8.42
8.40
8.38
7.79
7.77
7.75
7.71
7.70
7.68
7.55
7.54
7.53
7.51
7.50
7.48
7.47
4.08
4.06
4.05
4.03
4.01
3.48
3.42
1.29
1.27
1.26
3u
NO2
PO(OEt)2
N
Ph
0.97
2.00
0.99
2.09
3.22
4.00
2.11
6.76
f1 (ppm)

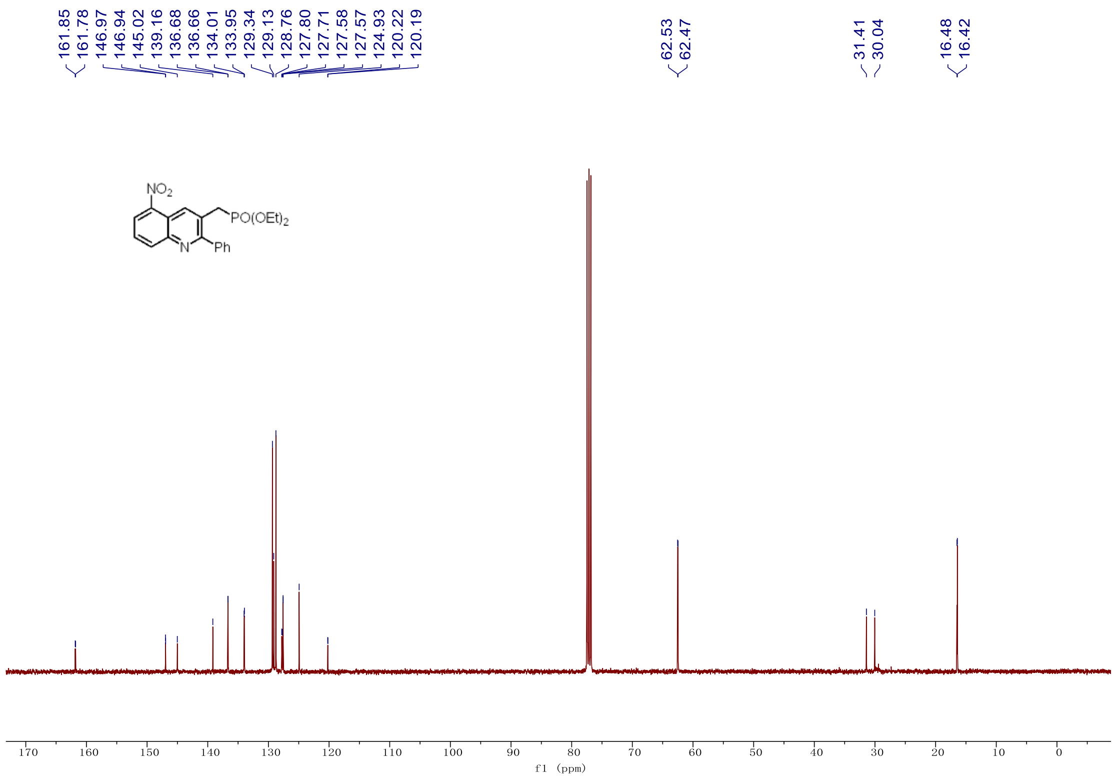
161.85
161.78
146.97
146.94
145.02
139.16
136.68
136.66
134.01
133.95
129.34
129.13
128.76
127.80
127.71
127.58
127.57
124.93
120.22
120.19
62.53
62.47
31.41
30.04
16.48
16.42
NO2
PO(OEt)2
N
Ph
170 160 150 140 130 120 110 100 90 80 70 60 50 40 30 20 10 0
f1 (ppm)

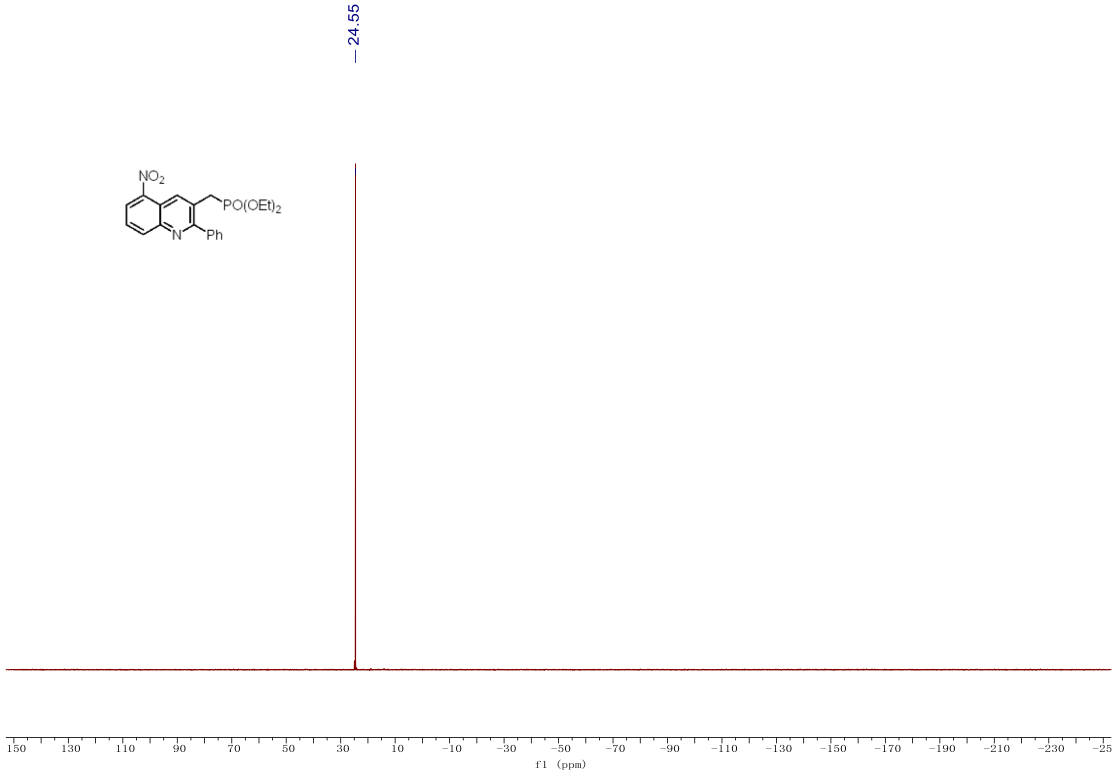
24.55
NO2
PO(OEt)2
N
Ph
150 130 110 90 70 50 30 10 −10 −30 −50 −70 −90 −110 −130 −150 −170 −190 −210 −230 −25
f1 (ppm)

3v

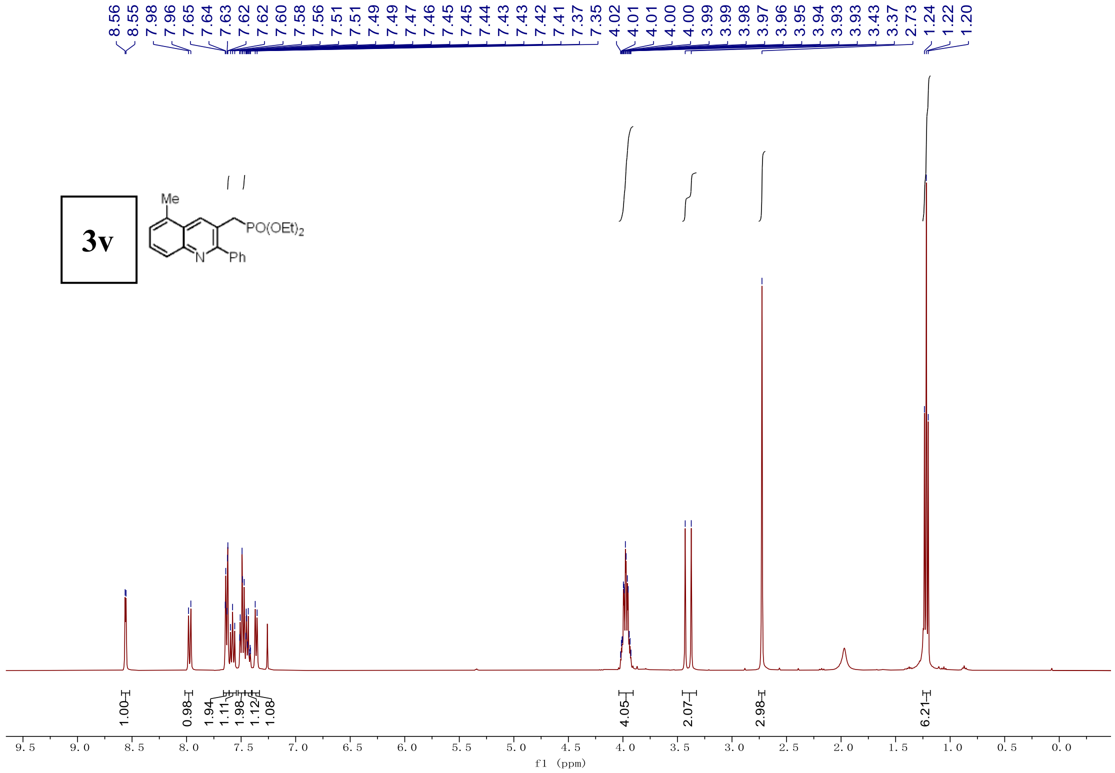
8.56
8.55
7.98
7.96
7.65
7.64
7.63
7.62
7.62
7.60
7.58
7.56
7.51
7.49
7.49
7.47
7.46
7.45
7.45
7.44
7.43
7.43
7.42
7.41
7.37
7.35
4.02
4.01
4.01
4.00
4.00
3.99
3.99
3.98
3.97
3.96
3.95
3.94
3.93
3.93
3.43
3.37
2.73
1.24
1.22
1.20
Me
PO(OEt)2
N
Ph
1.00
0.98
1.94
1.11
1.98
1.12
1.08
4.05
2.07
2.98
6.21
f1 (ppm)

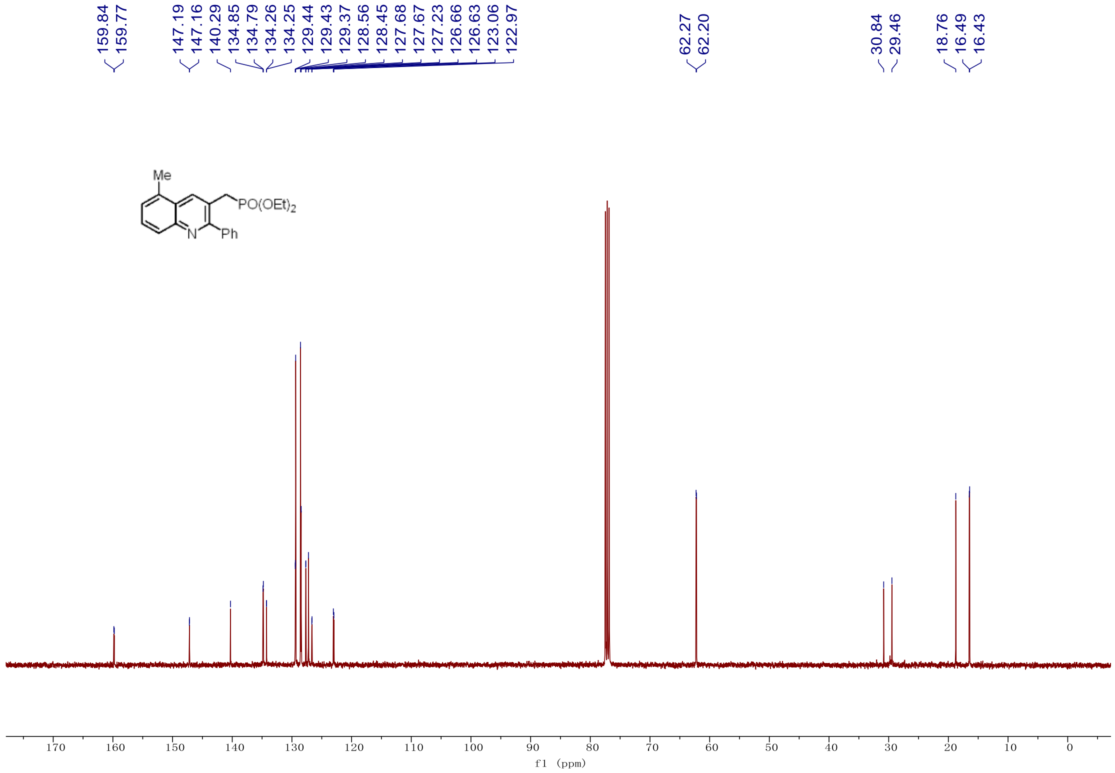
159.84
159.77
147.19
147.16
140.29
134.85
134.79
134.26
134.25
129.44
129.43
129.37
128.56
128.45
127.68
127.67
127.23
126.66
126.63
123.06
122.97
62.27
62.20
30.84
29.46
18.76
16.49
16.43
Me
PO(OEt)2
N
Ph
f1 (ppm)

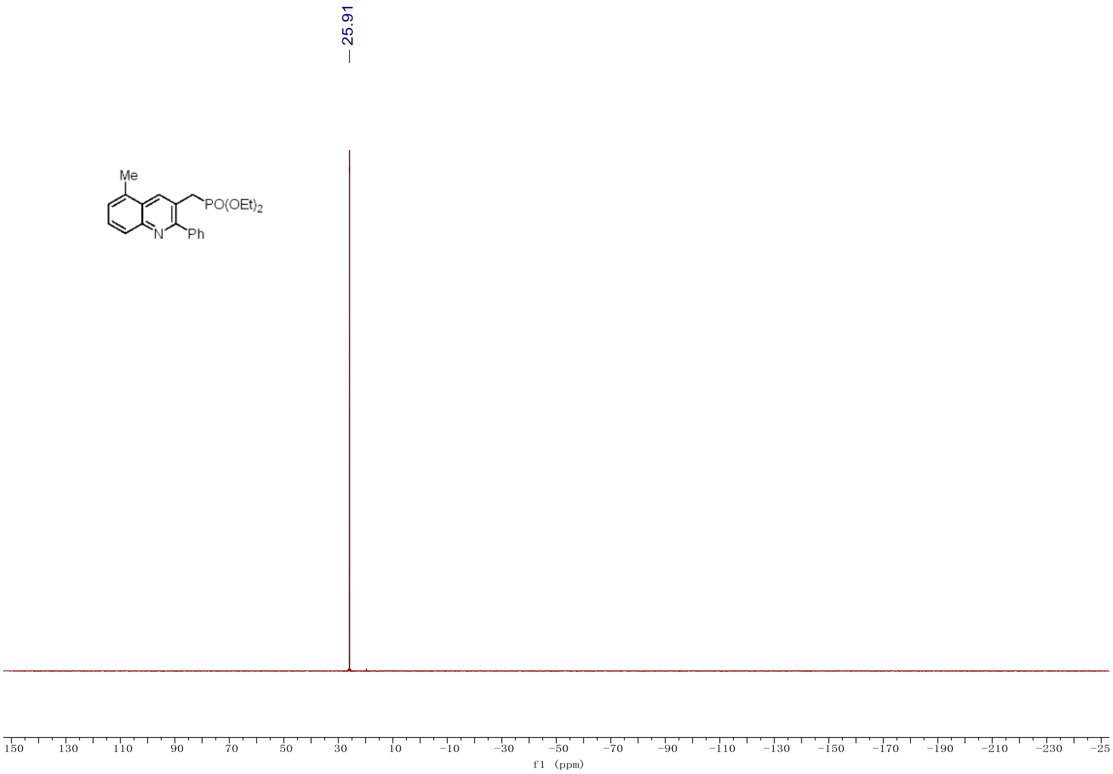
25.91
Me
PO(OEt)2
N
Ph
150 130 110 90 70 50 30 10 −10 −30 −50 −70 −90 −110 −130 −150 −170 −190 −210 −230 −25
f1 (ppm)

3w

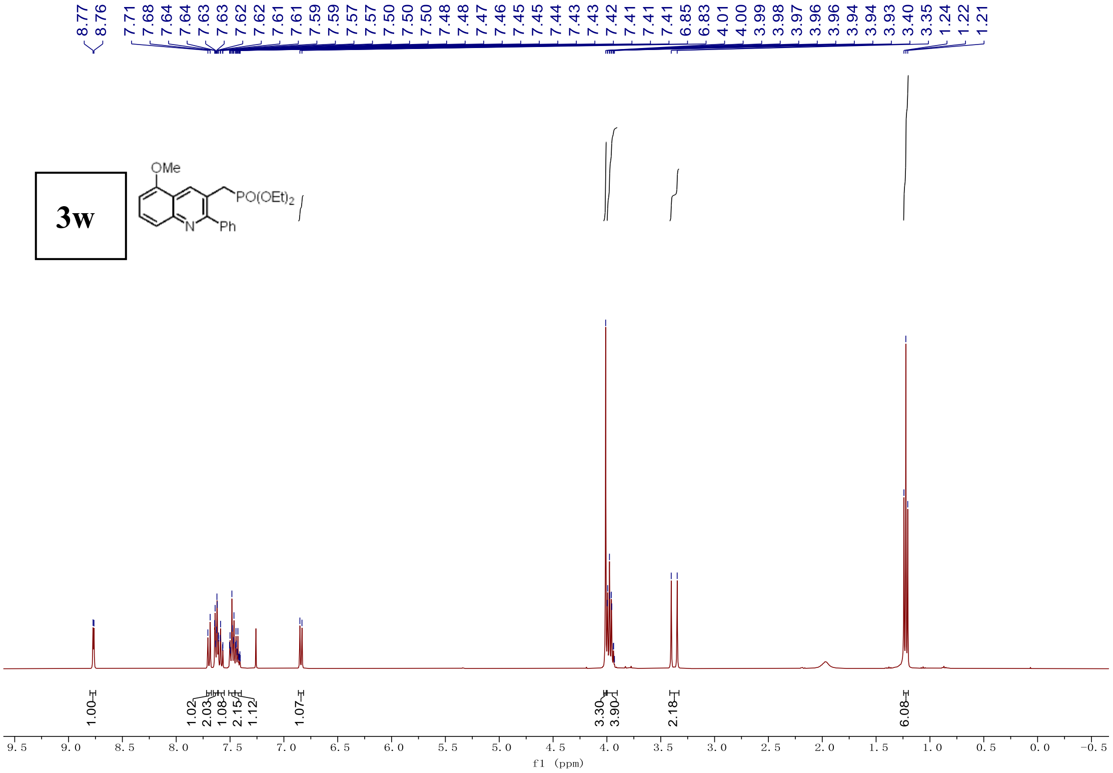
8.77 8.76 7.71 7.68 7.64 7.64 7.63 7.63 7.62 7.62 7.61 7.61 7.59 7.57 7.57 7.50 7.50 7.48 7.48 7.47 7.46 7.45 7.45 7.44 7.43 7.43 7.42 7.41 7.41 7.41 6.85 6.83 4.01 4.00 3.99 3.98 3.97 3.96 3.96 3.94 3.94 3.93 3.40 3.35 1.24 1.22 1.21
OMe
PO(OEt)2
N
Ph
1.00 1.02 2.03 1.08 2.15 1.12 1.07 3.30 3.90 2.18 6.08
9.5 9.0 8.5 8.0 7.5 7.0 6.5 6.0 5.5 5.0 4.5 4.0 3.5 3.0 2.5 2.0 1.5 1.0 0.5 0.0 −0.5
f1 (ppm)

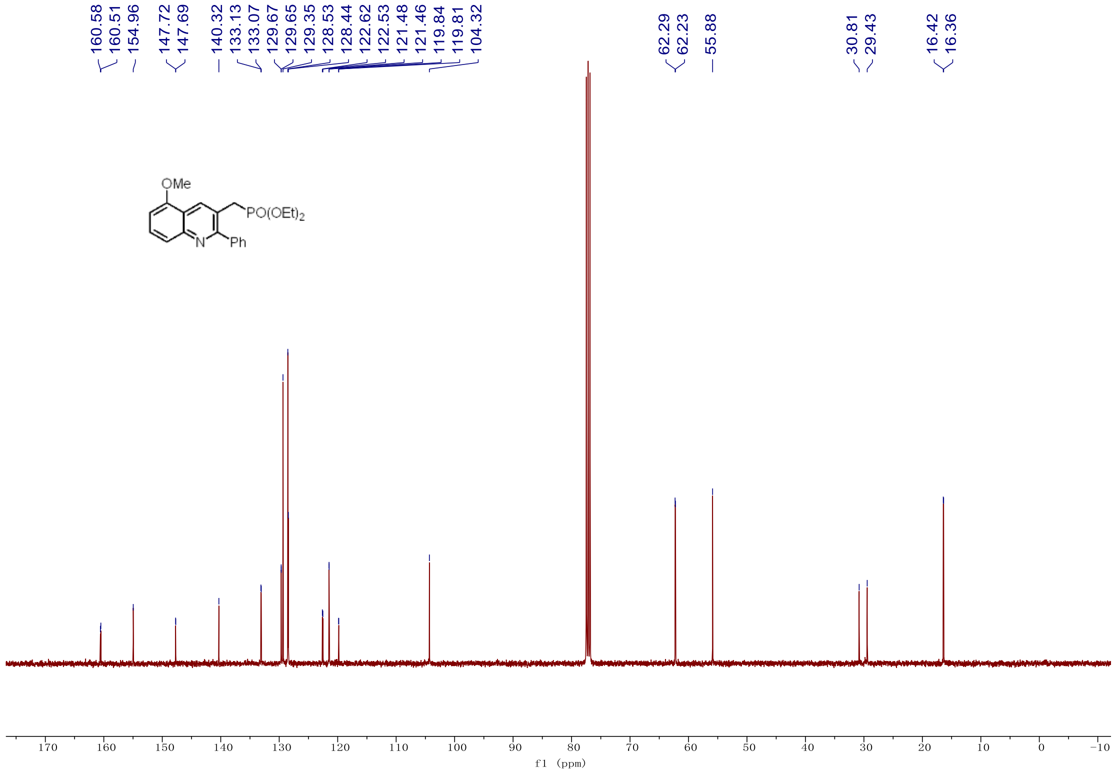
160.58
160.51
154.96
147.72
147.69
140.32
133.13
133.07
129.67
129.65
129.35
128.53
128.44
122.62
122.53
121.48
121.46
119.84
119.81
104.32
62.29
62.23
55.88
30.81
29.43
16.42
16.36
OMe
PO(OEt)2
N
Ph
f1 (ppm)

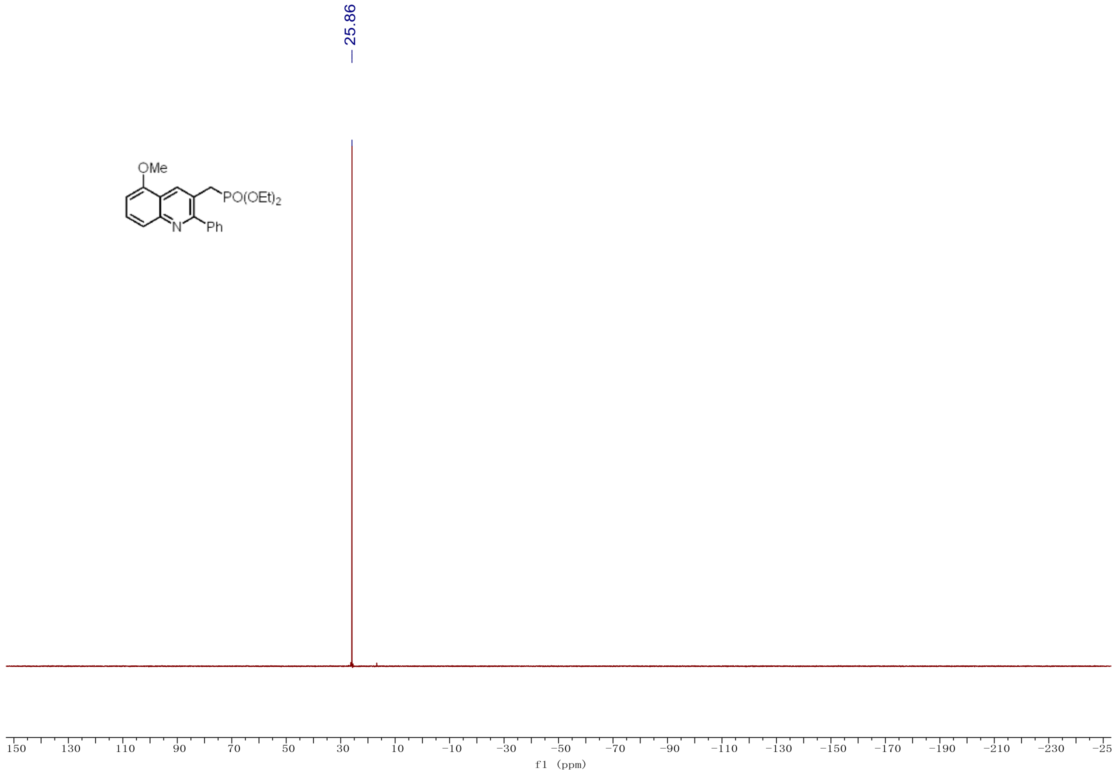
25.86
OMe
PO(OEt)2
N
Ph
f1 (ppm)

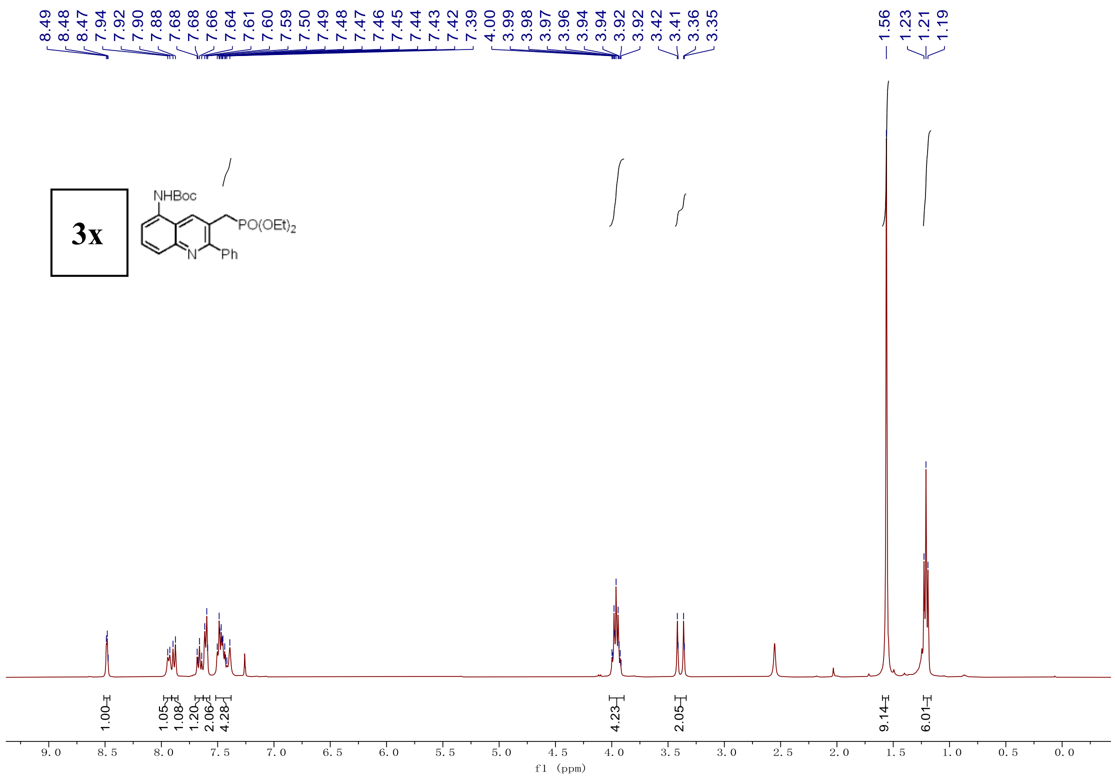
8.49 8.48 8.47 7.94 7.92 7.90 7.88 7.68 7.68 7.66 7.64 7.61 7.60 7.59 7.50 7.49 7.48 7.47 7.46 7.45 7.44 7.43 7.42 7.39 4.00 3.99 3.98 3.97 3.96 3.94 3.94 3.92 3.92 3.42 3.41 3.36 3.35
1.56 1.23 1.21 1.19
3x
NHBoc
PO(OEt)2
N
Ph
1.00 1.05 1.08 1.20 2.06 4.28 4.23 2.05 9.14 6.01
f1 (ppm)

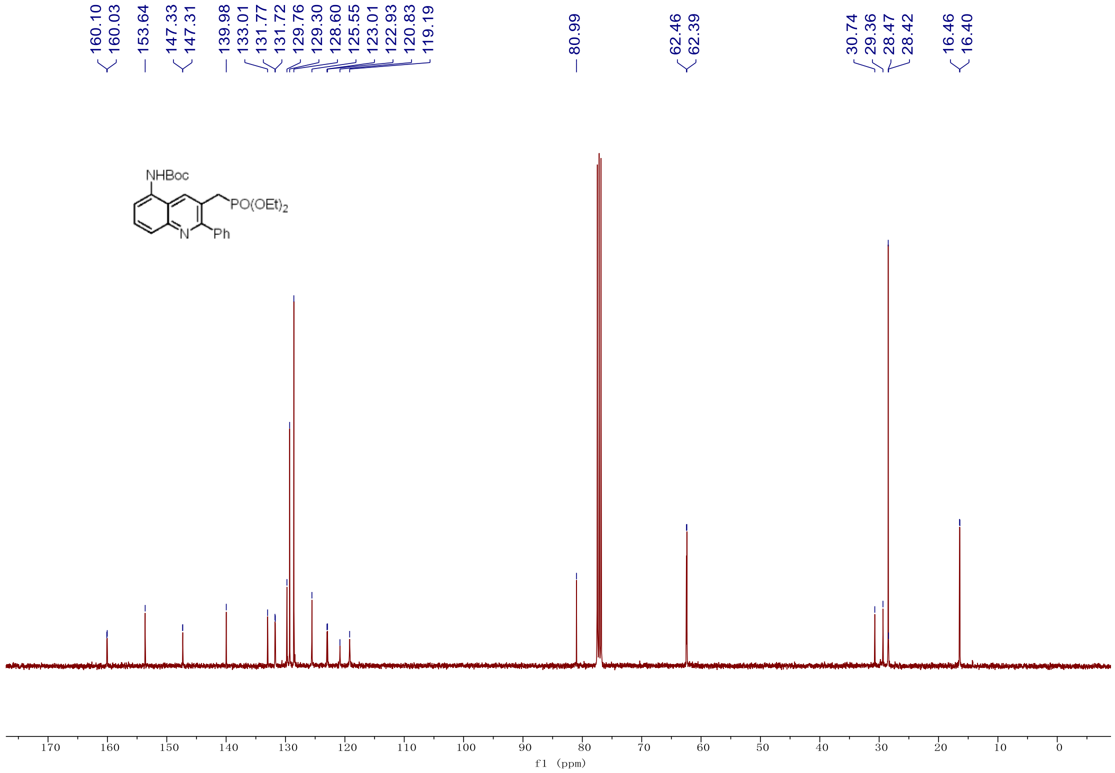
160.10 160.03 153.64 147.33 147.31 139.98 133.01 131.77 131.72 129.76 129.30 128.60 125.55 123.01 122.93 120.83 119.19
80.99
62.46 62.39
30.74 29.36 28.47 28.42 16.46 16.40
NHBoc
PO(OEt)2
N
Ph
f1 (ppm)

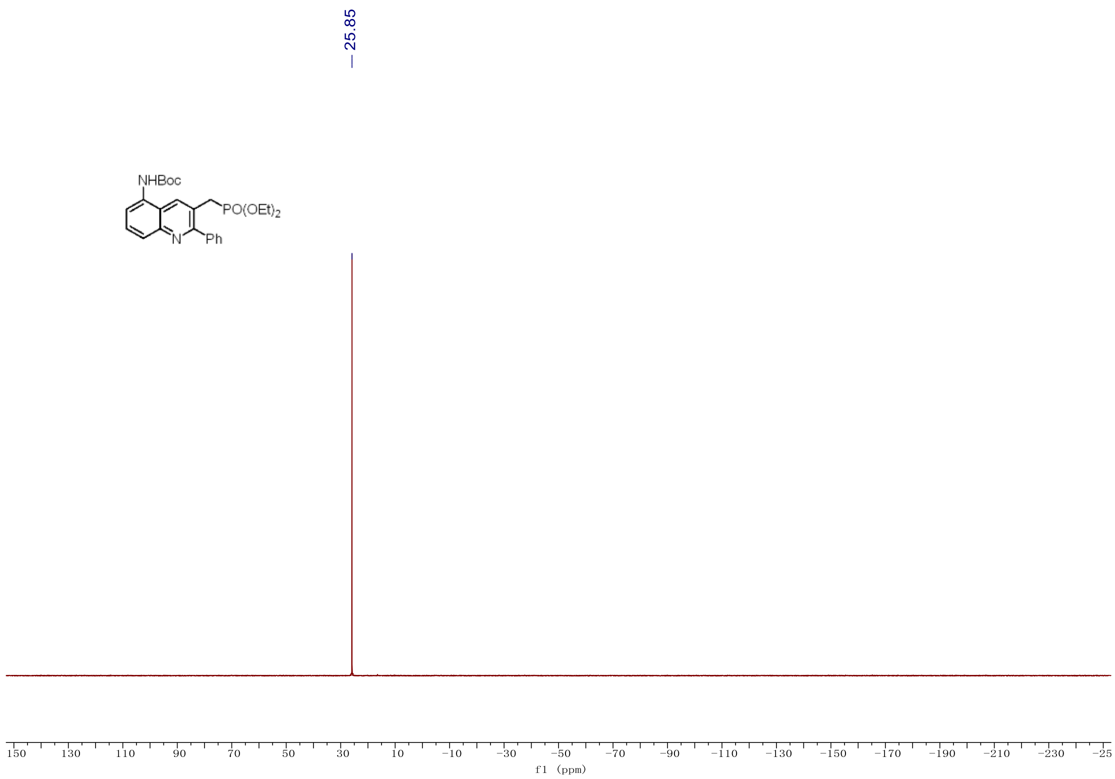

25.85
NHBoc
PO(OEt)2
N
Ph
f1 (ppm)

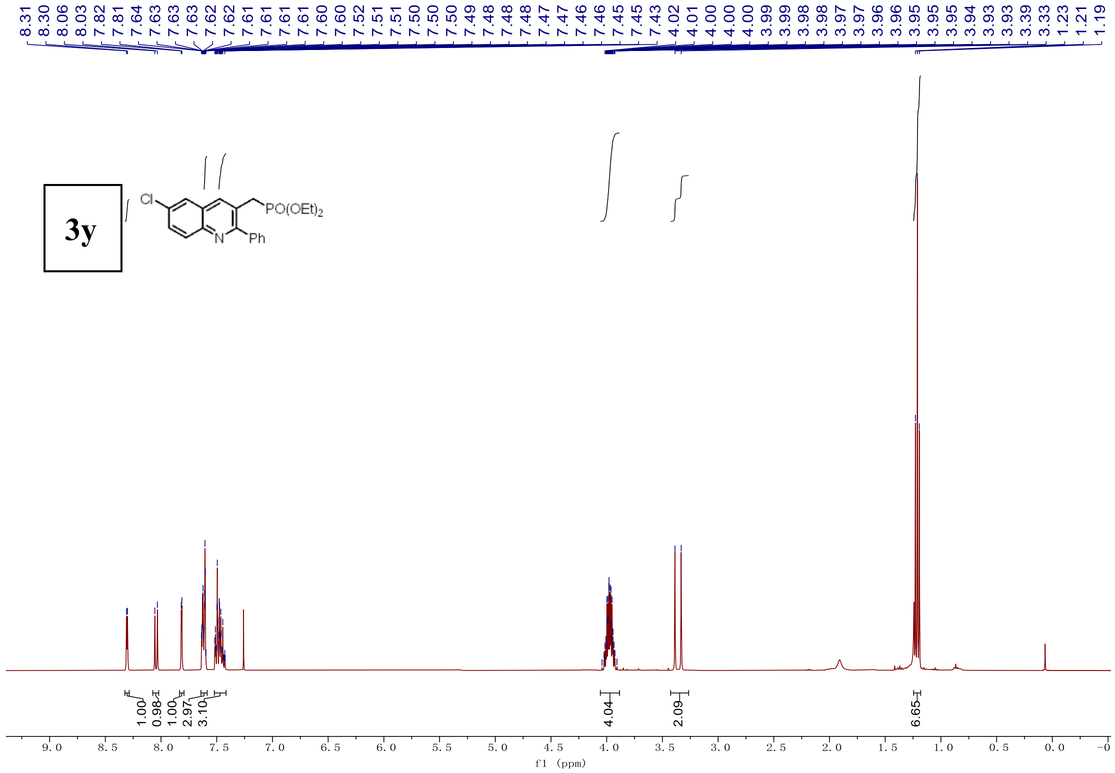

3y
Cl
PO(OEt)2
N
Ph
8.31
8.30
8.06
8.03
7.82
7.81
7.64
7.63
7.63
7.62
7.62
7.61
7.61
7.61
7.61
7.60
7.60
7.52
7.51
7.51
7.50
7.50
7.49
7.48
7.48
7.48
7.47
7.47
7.46
7.46
7.45
7.45
7.43
4.02
4.01
4.00
4.00
3.99
3.99
3.98
3.98
3.97
3.97
3.96
3.96
3.95
3.95
3.94
3.93
3.93
3.39
3.33
1.23
1.21
1.19
1.00
0.98
1.00
2.97
3.10
4.04
2.09
6.65
f1 (ppm)

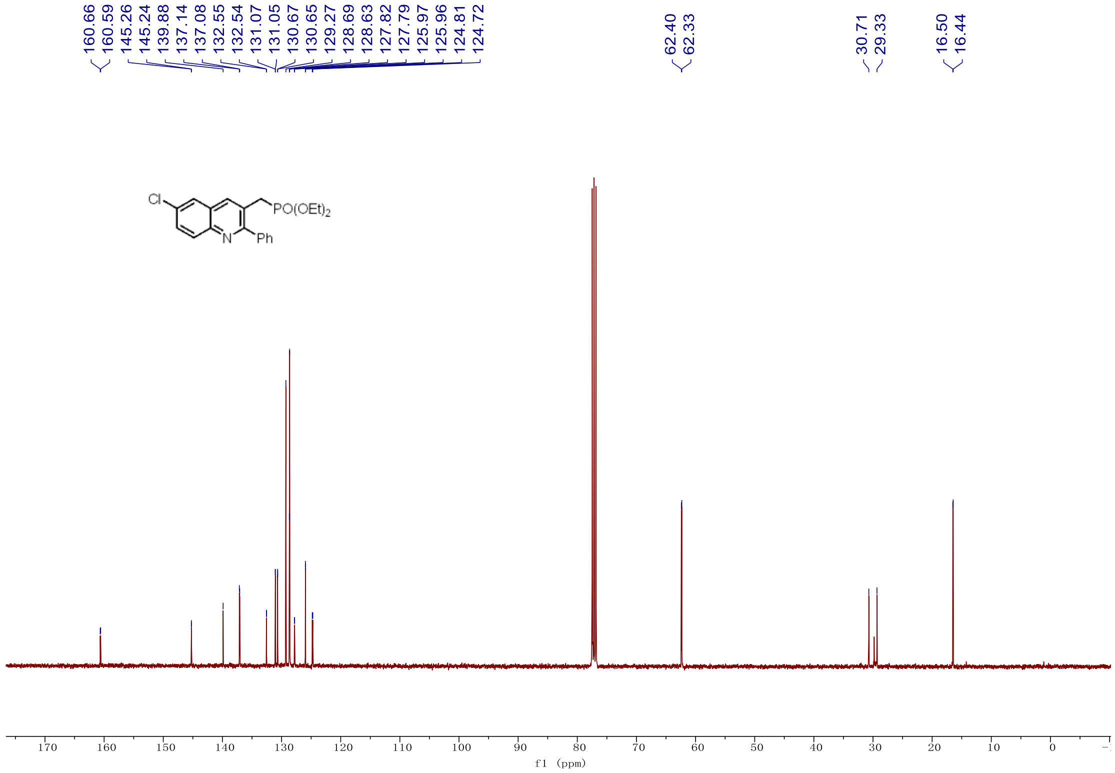
160.66
160.59
145.26
145.24
139.88
137.14
137.08
132.55
132.54
131.07
131.05
130.67
130.65
129.27
128.69
128.63
127.82
127.79
125.97
125.96
124.81
124.72
62.40
62.33
30.71
29.33
16.50
16.44
Cl
PO(OEt)2
N
Ph
f1 (ppm)

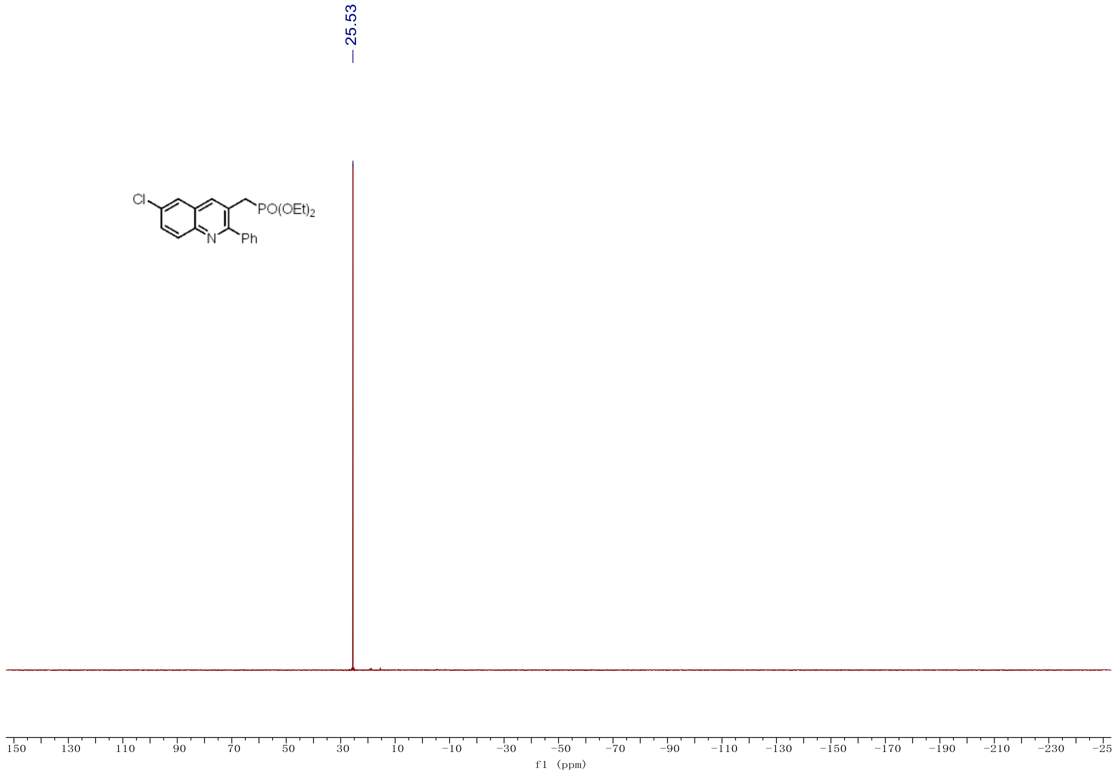
25.53
Cl
PO(OEt)2
N
Ph
f1 (ppm)

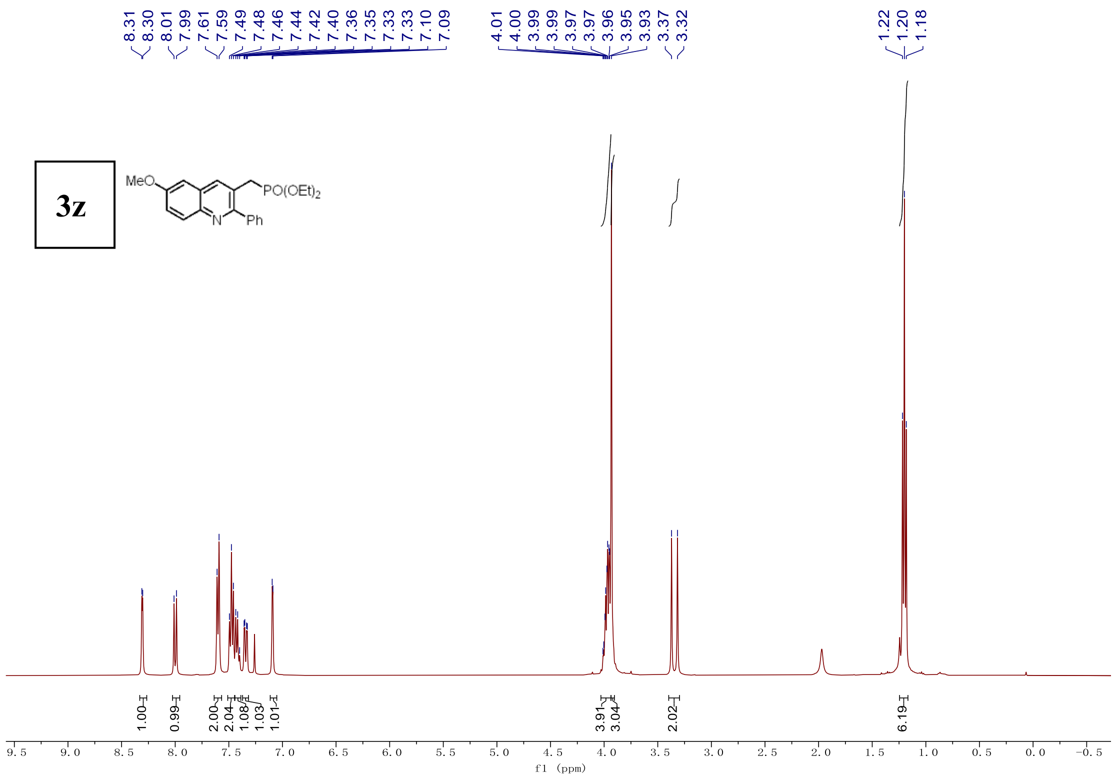
3z
MeO
PO(OEt)2
N
Ph
8.31 8.30 8.01 7.99 7.61 7.59 7.49 7.48 7.46 7.44 7.42 7.40 7.36 7.35 7.33 7.33 7.10 7.09
4.01 4.00 3.99 3.99 3.97 3.97 3.96 3.95 3.93 3.37 3.32
1.22 1.20 1.18
1.00 0.99 2.00 2.04 1.08 1.03 1.01 3.91 3.04 2.02 6.19
f1 (ppm)

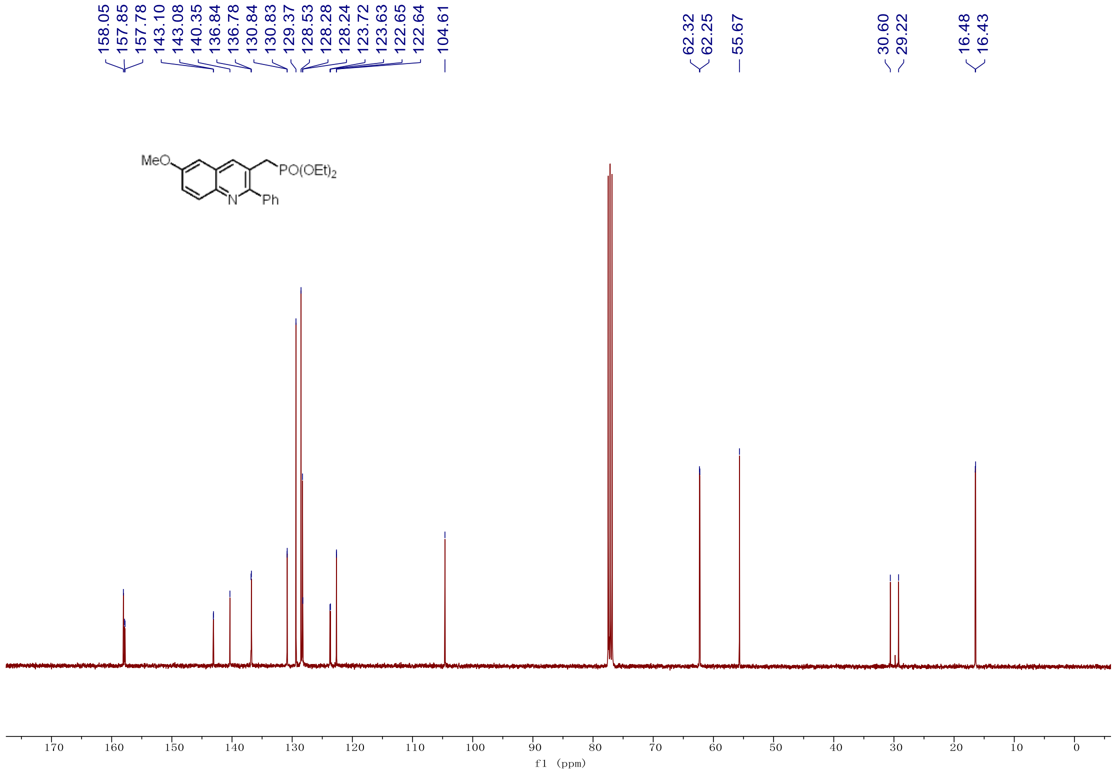
158.05 157.85 157.78 143.10 143.08 140.35 136.84 136.78 130.84 130.83 129.37 128.53 128.28 128.24 123.72 123.63 122.65 122.64 104.61
62.32 62.25 55.67
30.60 29.22
16.48 16.43
MeO
PO(OEt)2
N
Ph
f1 (ppm)

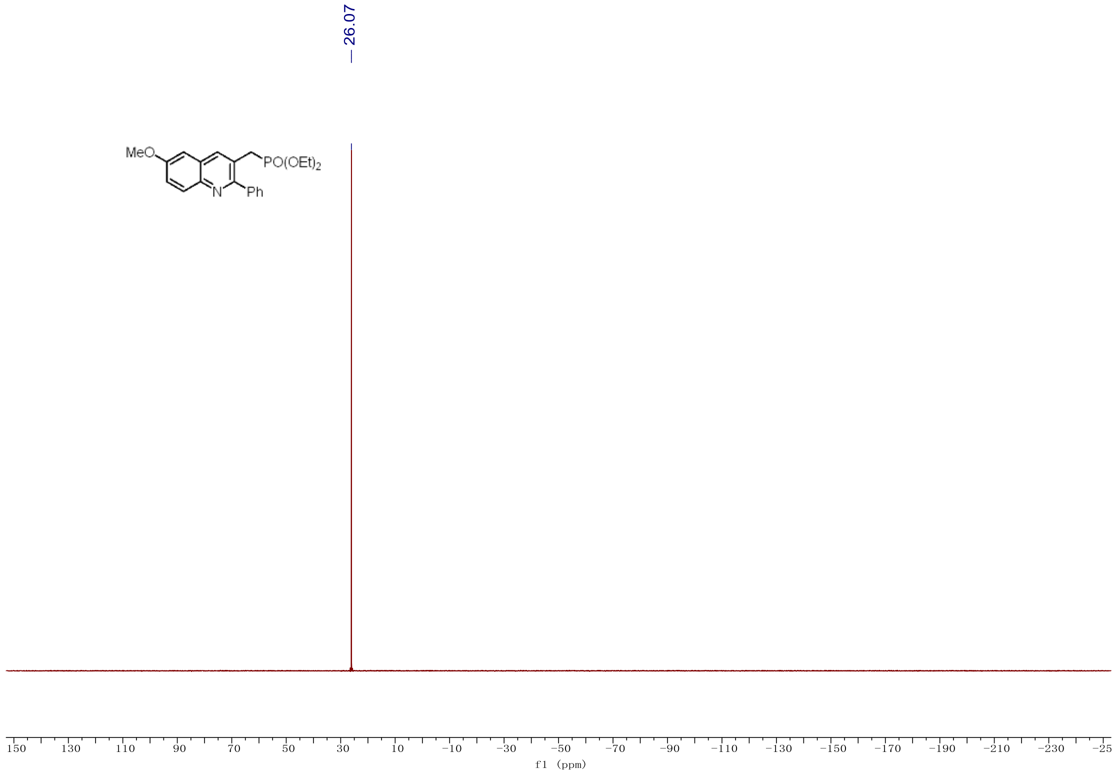

26.07
MeO
PO(OEt)2
N
Ph
f1 (ppm)

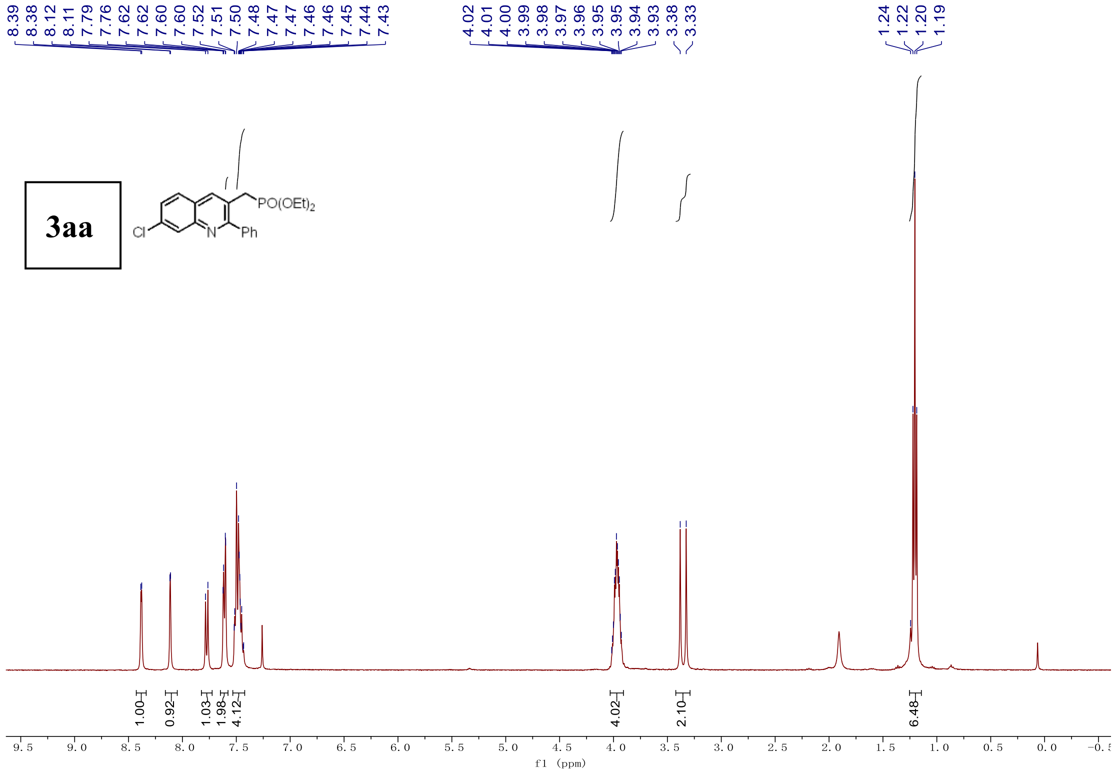

8.39
8.38
8.12
8.11
7.79
7.76
7.62
7.62
7.60
7.60
7.52
7.51
7.50
7.48
7.47
7.47
7.46
7.46
7.45
7.44
7.43
4.02
4.01
4.00
3.99
3.98
3.97
3.96
3.95
3.95
3.94
3.93
3.38
3.33
1.24
1.22
1.20
1.19
3aa
Cl
N
PO(OEt)2
Ph
1.00
0.92
1.03
1.98
4.12
4.02
2.10
6.48
f1 (ppm)

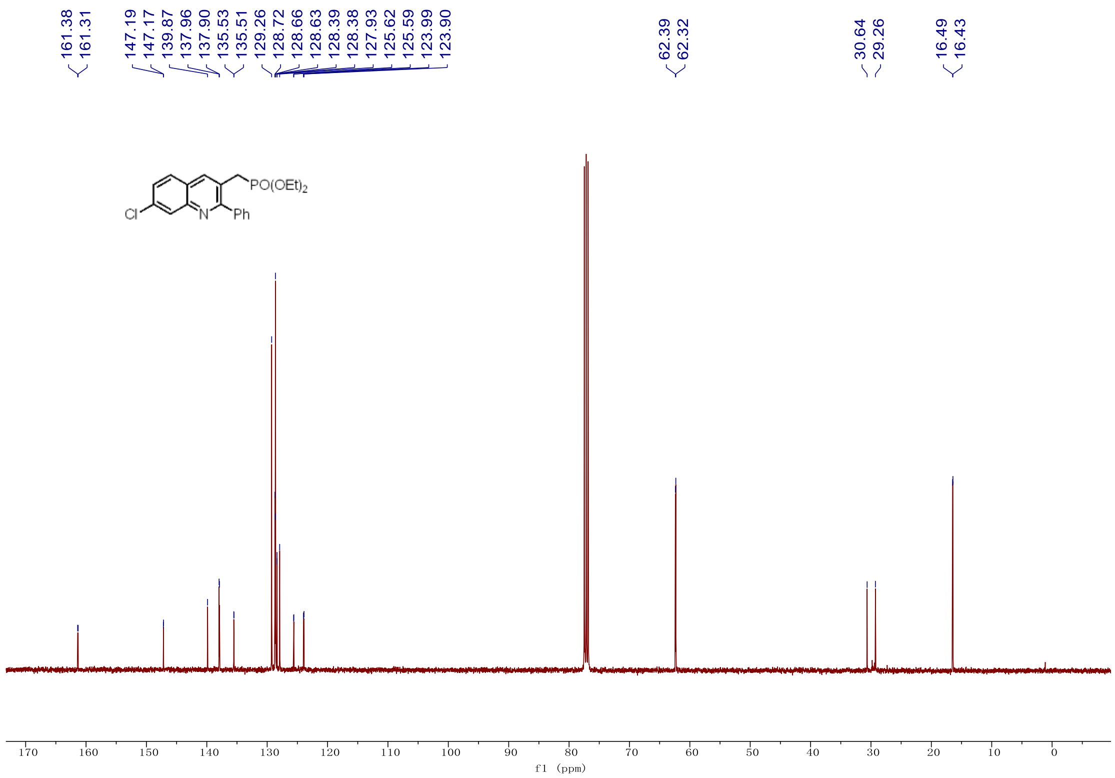
161.38
161.31
147.19
147.17
139.87
137.96
137.90
135.53
135.51
129.26
128.72
128.66
128.63
128.39
128.38
127.93
125.62
125.59
123.99
123.90
62.39
62.32
30.64
29.26
16.49
16.43
PO(OEt)2
Cl
N
Ph
f1 (ppm)

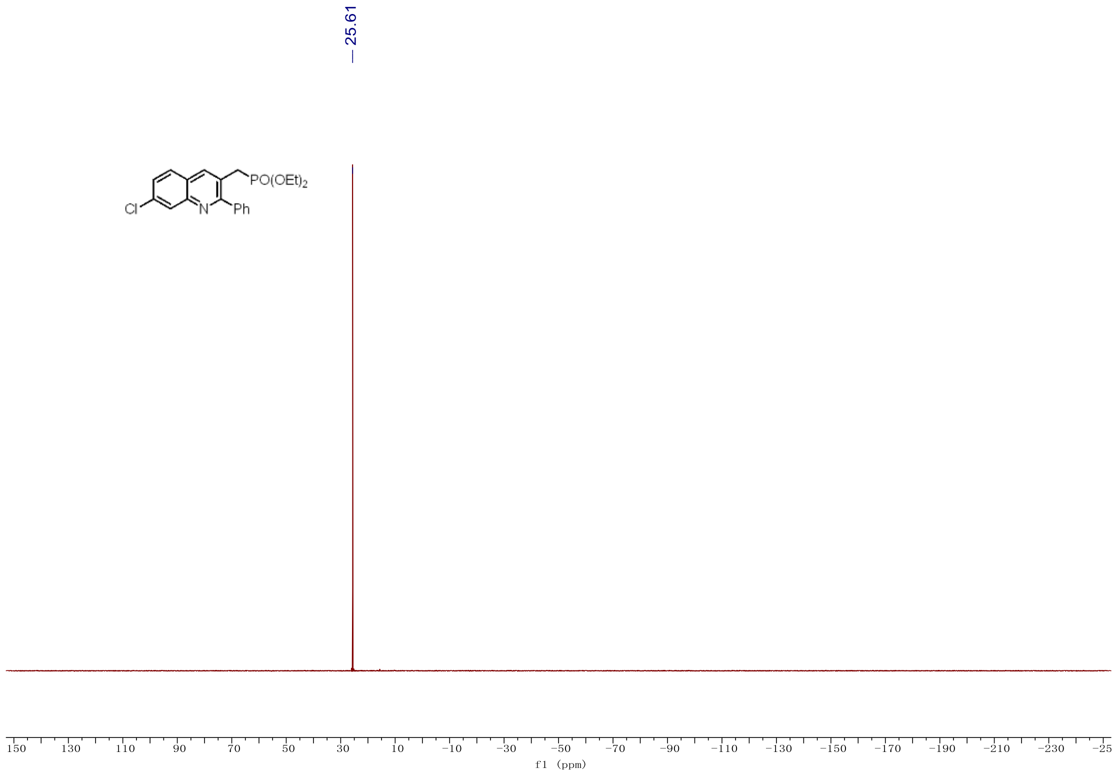
25.61
PO(OEt)2
Cl
N
Ph
f1 (ppm)

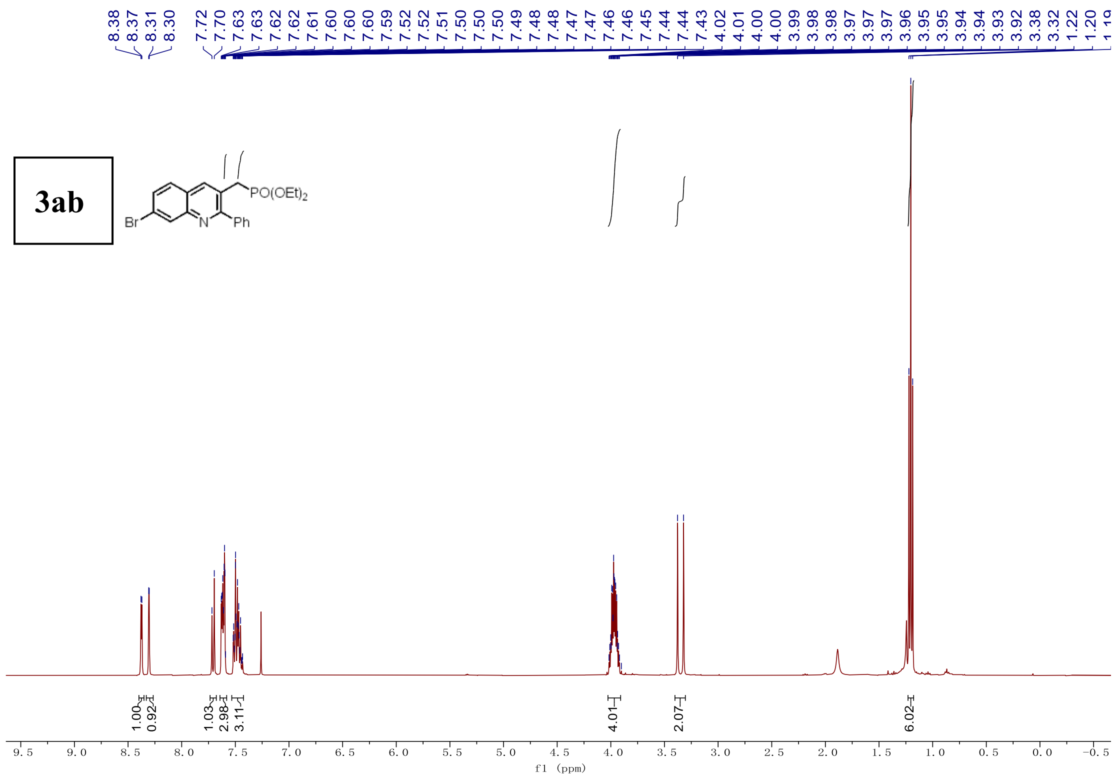
8.38
8.37
8.31
8.30
7.72
7.70
7.63
7.63
7.62
7.62
7.61
7.60
7.60
7.59
7.52
7.52
7.51
7.50
7.50
7.49
7.48
7.47
7.47
7.46
7.46
7.45
7.44
7.44
7.43
4.02
4.01
4.00
3.99
3.98
3.98
3.97
3.97
3.97
3.96
3.95
3.95
3.94
3.94
3.93
3.92
3.38
3.32
1.22
1.20
3ab
PO(OEt)2
Br
N
Ph
1.00
0.92
1.03
2.98
3.11
4.01
2.07
6.02
f1 (ppm)

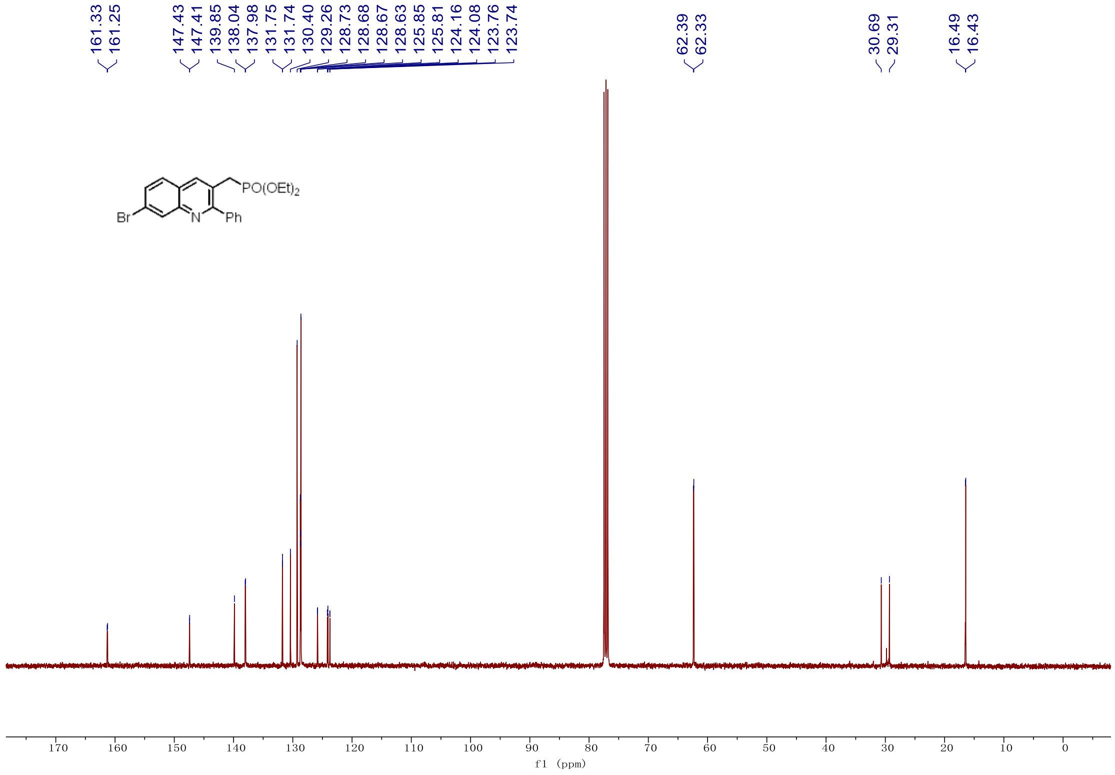
161.33
161.25
147.43
147.41
139.85
138.04
137.98
131.75
131.74
130.40
129.26
128.73
128.68
128.67
128.63
125.85
125.81
124.16
124.08
123.76
123.74
62.39
62.33
30.69
29.31
16.49
16.43
PO(OEt)2
Br
N
Ph
f1 (ppm)

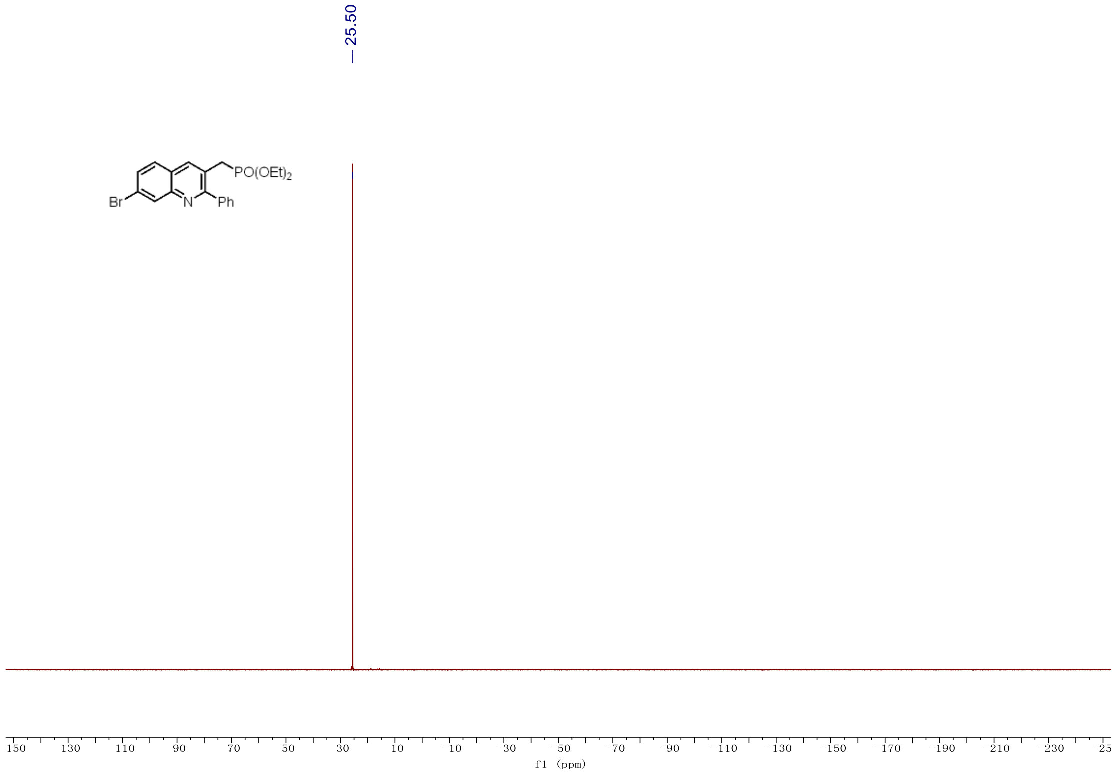
25.50
Br
N
Ph
PO(OEt)2
150
130
110
90
70
50
30
10
−10
−30
−50
−70
−90
−110
−130
−150
−170
−190
−210
−230
f1 (ppm)

3ac

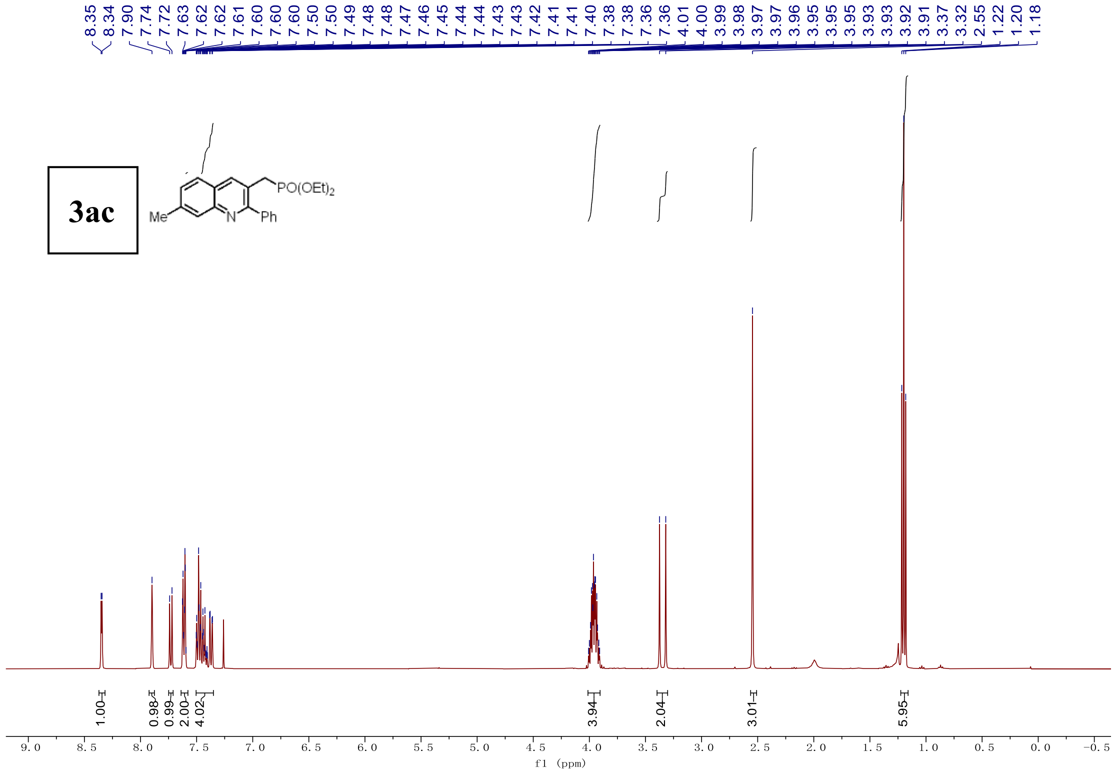
8.35
8.34
7.90
7.74
7.72
7.63
7.62
7.61
7.60
7.60
7.60
7.50
7.49
7.48
7.48
7.47
7.46
7.45
7.44
7.44
7.43
7.43
7.42
7.41
7.41
7.40
7.38
7.38
7.36
7.36
4.01
4.00
3.99
3.98
3.97
3.97
3.96
3.95
3.95
3.93
3.93
3.92
3.91
3.37
3.32
2.55
1.22
1.20
1.18
Me
N
Ph
PO(OEt)2
1.00
0.98
0.99
2.00
4.02
3.94
2.04
3.01
5.95
9.0
8.5
8.0
7.5
7.0
6.5
6.0
5.5
5.0
4.5
4.0
3.5
3.0
2.5
2.0
1.5
1.0
0.5
0.0
−0.5
f1 (ppm)

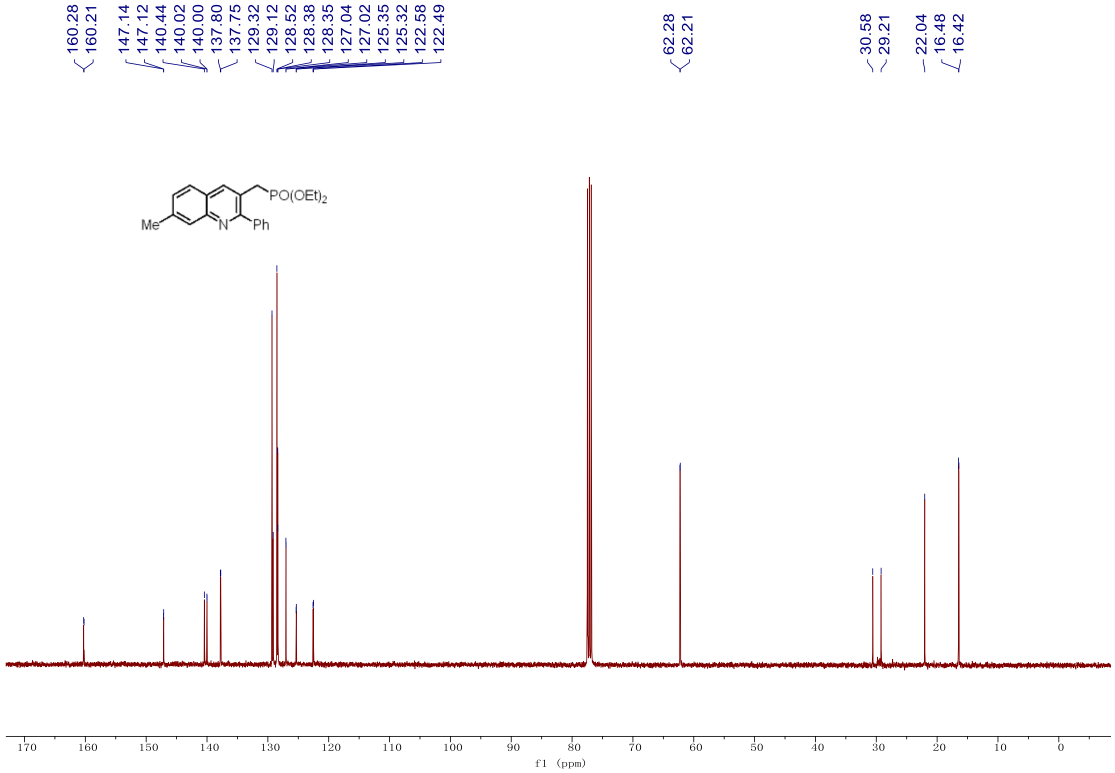
160.28
160.21
147.14
147.12
140.44
140.02
140.00
137.80
137.75
129.32
129.12
128.52
128.38
128.35
127.04
127.02
125.35
125.32
122.58
122.49
62.28
62.21
30.58
29.21
22.04
16.48
16.42
PO(OEt)2
Me
N
Ph
f1 (ppm)

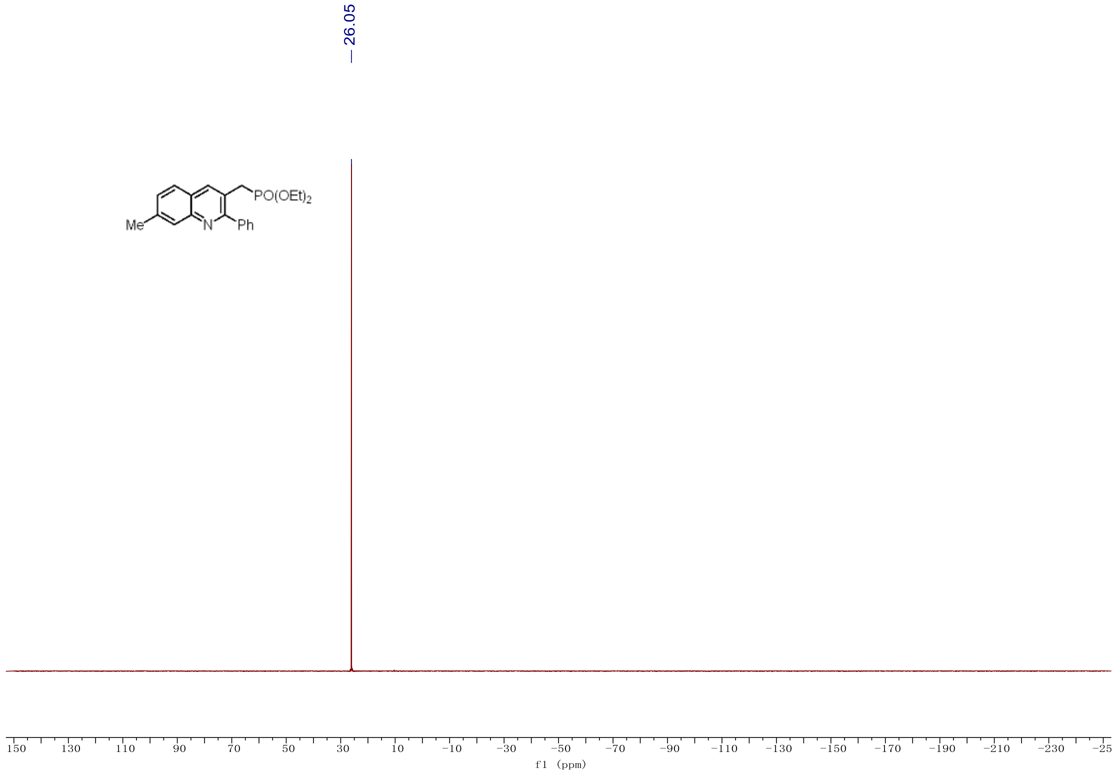
26.05
PO(OEt)2
Me
N
Ph
f1 (ppm)

3ad

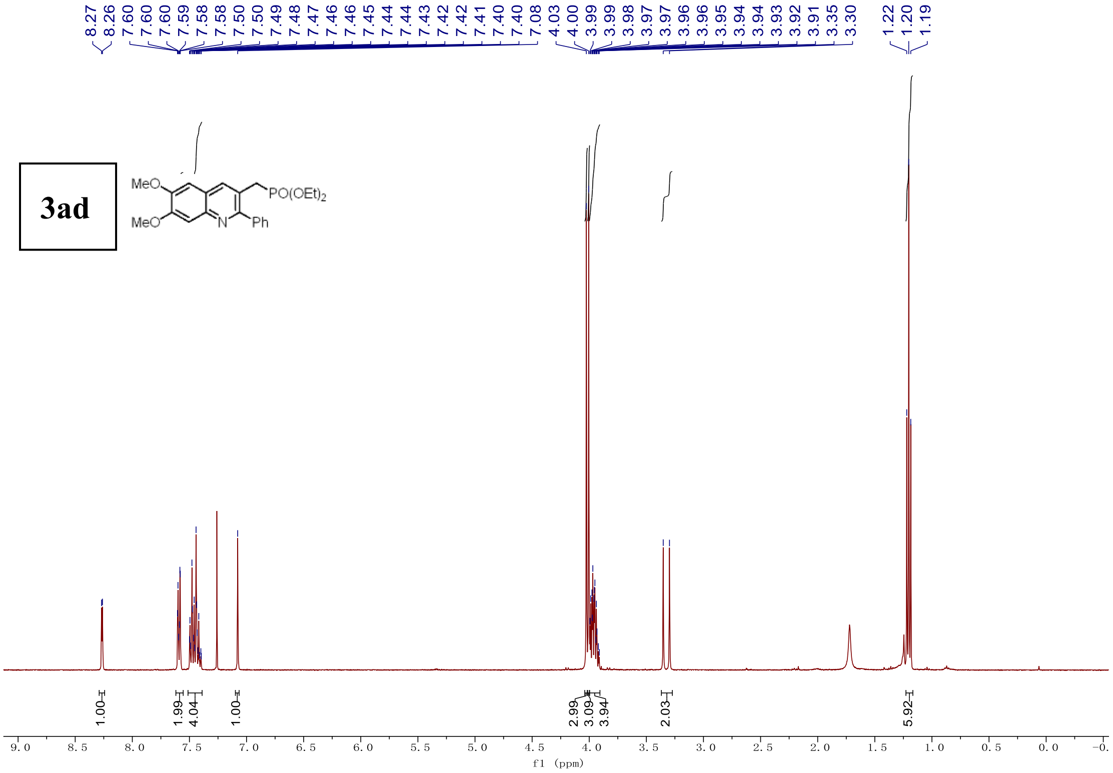
8.27 8.26 7.60 7.60 7.59 7.58 7.58 7.50 7.49 7.48 7.47 7.46 7.46 7.45 7.44 7.44 7.43 7.42 7.42 7.41 7.40 7.40 7.08 4.03 4.00 3.99 3.99 3.98 3.97 3.97 3.96 3.96 3.95 3.94 3.94 3.93 3.92 3.91 3.35 3.30 1.22 1.20 1.19
MeO
MeO
N
Ph
PO(OEt)2
1.00
1.99
4.04
1.00
2.99
3.09
3.94
2.03
5.92
f1 (ppm)

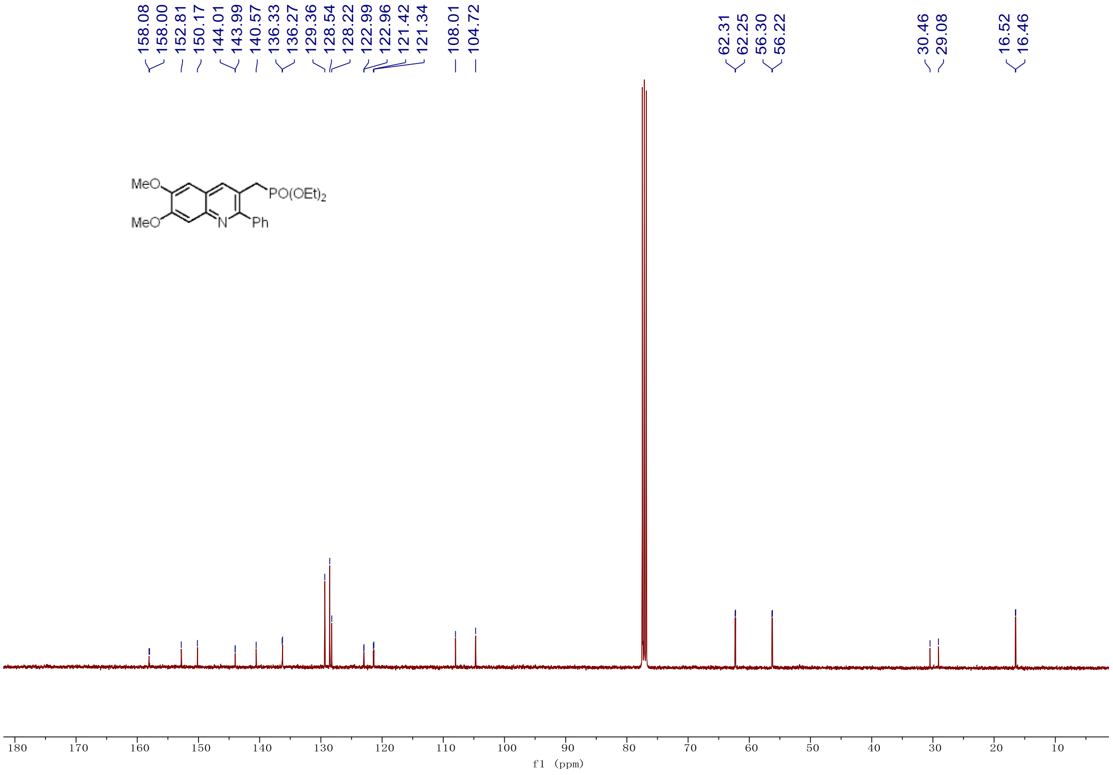
158.08 158.00 152.81 150.17 144.01 143.99 140.57 136.33 136.27 129.36 128.54 128.22 122.99 122.96 121.42 121.34 108.01 104.72
62.31 62.25 56.30 56.22
30.46 29.08
16.52 16.46
MeO
MeO
N
Ph
PO(OEt)2
f1 (ppm)

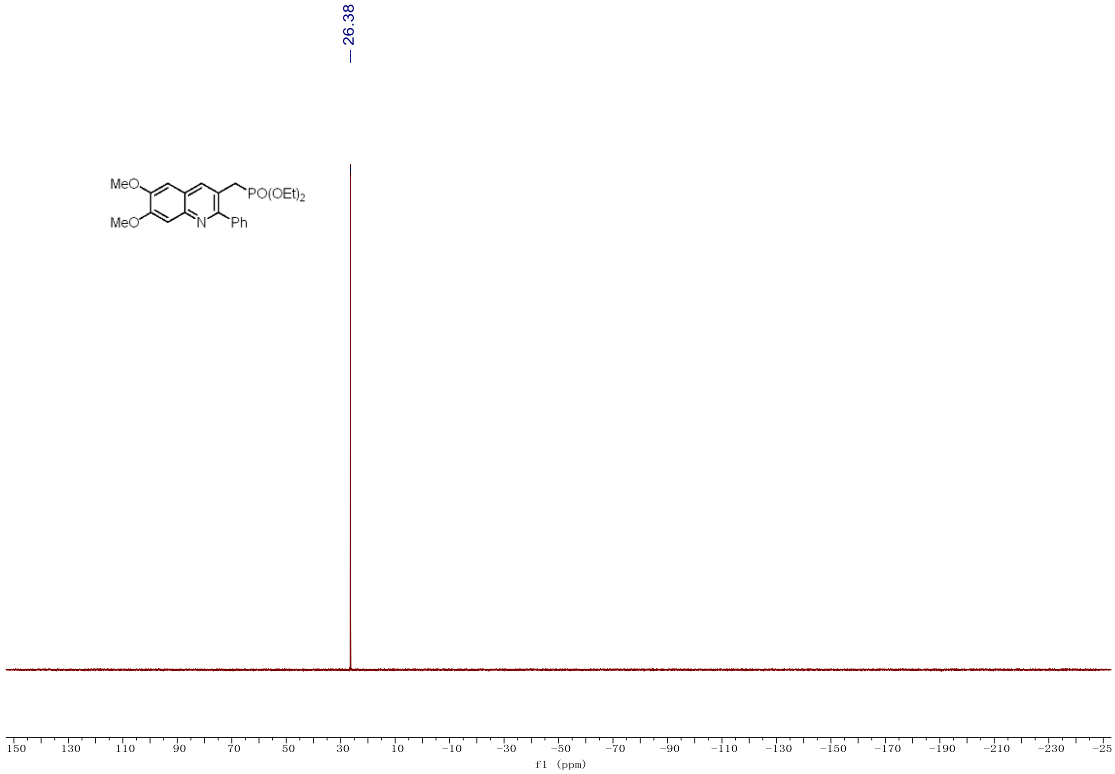
26.38
MeO
MeO
N
PO(OEt)2
Ph
f1 (ppm)

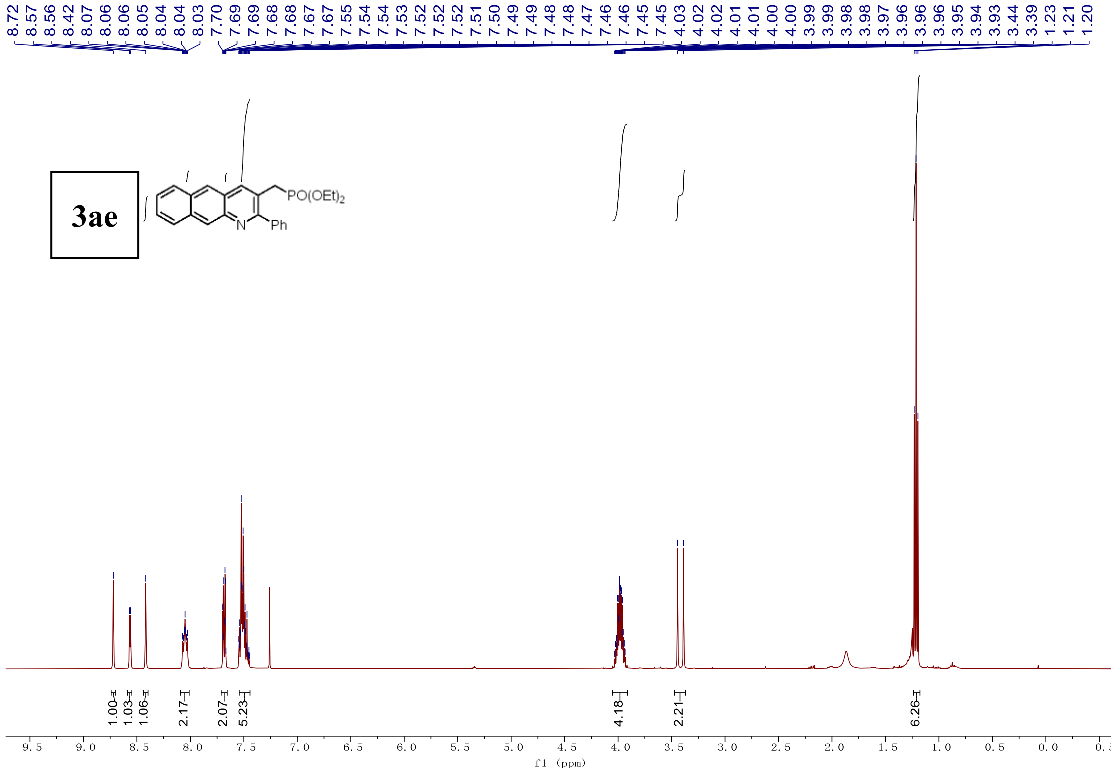
8.72
8.57
8.56
8.42
8.07
8.06
8.06
8.05
8.04
8.04
8.03
7.70
7.69
7.69
7.68
7.68
7.67
7.67
7.55
7.54
7.54
7.53
7.52
7.52
7.52
7.51
7.50
7.49
7.49
7.48
7.48
7.47
7.46
7.46
7.45
7.45
4.03
4.02
4.02
4.01
4.01
4.00
4.00
3.99
3.99
3.98
3.98
3.97
3.96
3.96
3.96
3.95
3.94
3.93
3.44
3.39
1.23
1.21
1.20
3ae
PO(OEt)2
N
Ph
1.00
1.03
1.06
2.17
2.07
5.23
4.18
2.21
6.26
f1 (ppm)

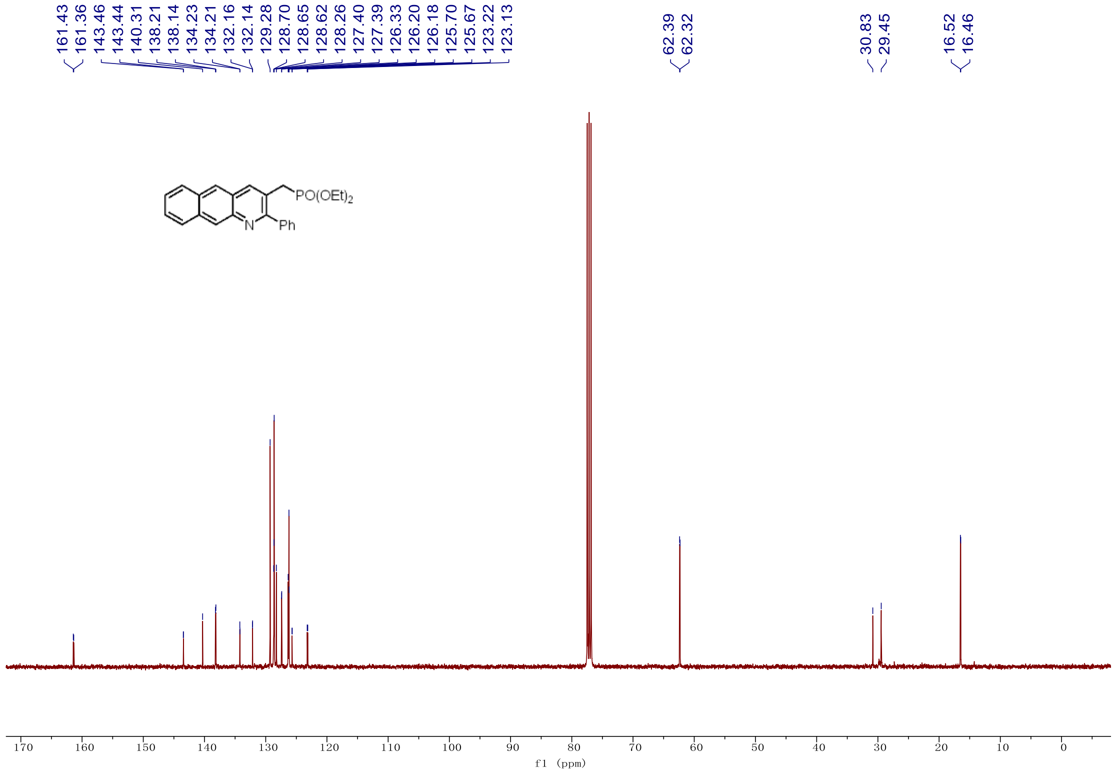
161.43
161.36
143.46
143.44
140.31
138.21
138.14
134.23
134.21
132.16
132.14
129.28
128.70
128.65
128.62
128.26
127.40
127.39
126.33
126.20
126.18
125.70
125.67
123.22
123.13
62.39
62.32
30.83
29.45
16.52
16.46
PO(OEt)2
N
Ph
f1 (ppm)

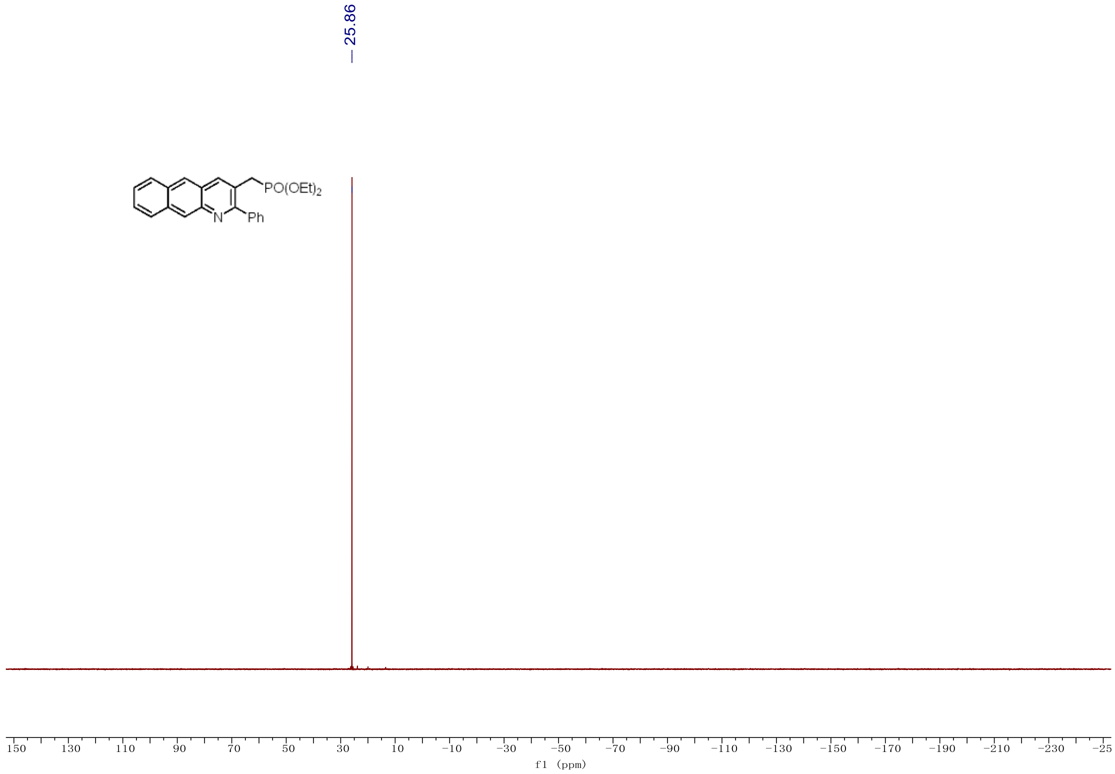
25.86
PO(OEt)2
N
Ph
f1 (ppm)

3af

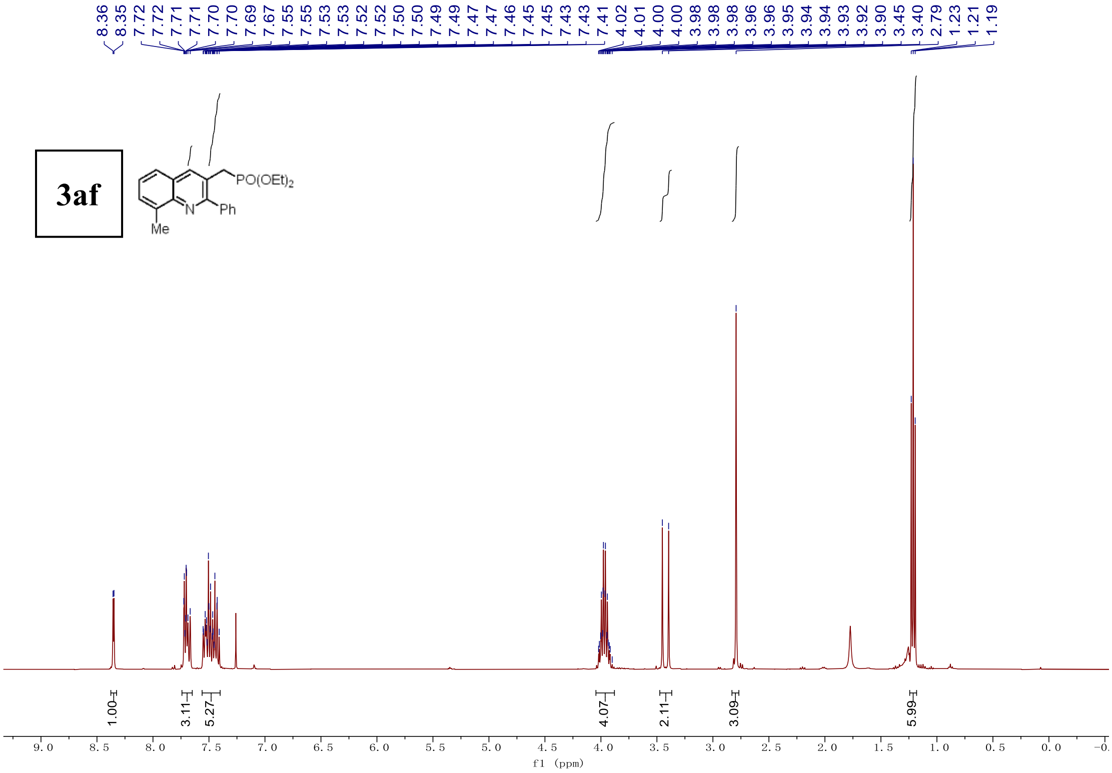
8.36
8.35
7.72
7.72
7.71
7.71
7.70
7.70
7.69
7.67
7.55
7.55
7.53
7.53
7.52
7.52
7.50
7.50
7.49
7.49
7.47
7.47
7.46
7.45
7.45
7.43
7.43
7.41
4.02
4.01
4.00
4.00
3.98
3.98
3.98
3.96
3.96
3.95
3.94
3.94
3.93
3.92
3.90
3.45
3.40
2.79
1.23
1.21
1.19
PO(OEt)2
N
Ph
Me
1.00
3.11
5.27
4.07
2.11
3.09
5.99
f1 (ppm)

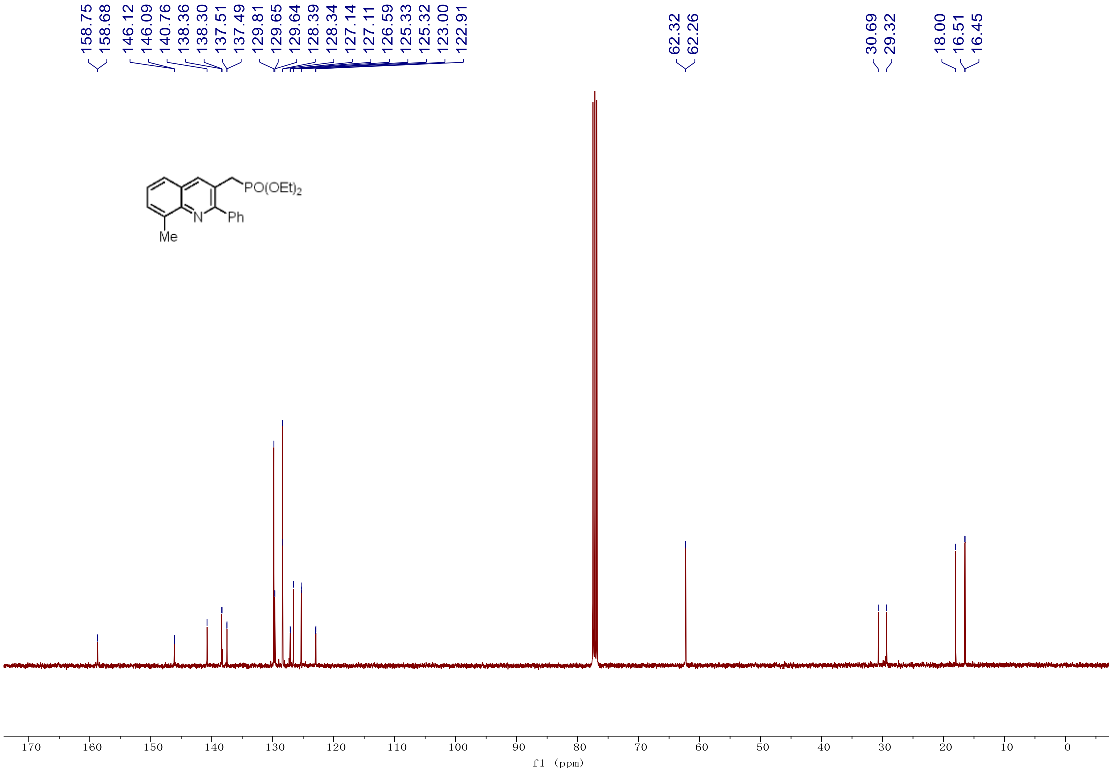
158.75
158.68
146.12
146.09
140.76
138.36
138.30
137.51
137.49
129.81
129.65
129.64
128.39
128.34
127.14
127.11
126.59
125.33
125.32
123.00
122.91
62.32
62.26
30.69
29.32
18.00
16.51
16.45
PO(OEt)2
N
Ph
Me
f1 (ppm)

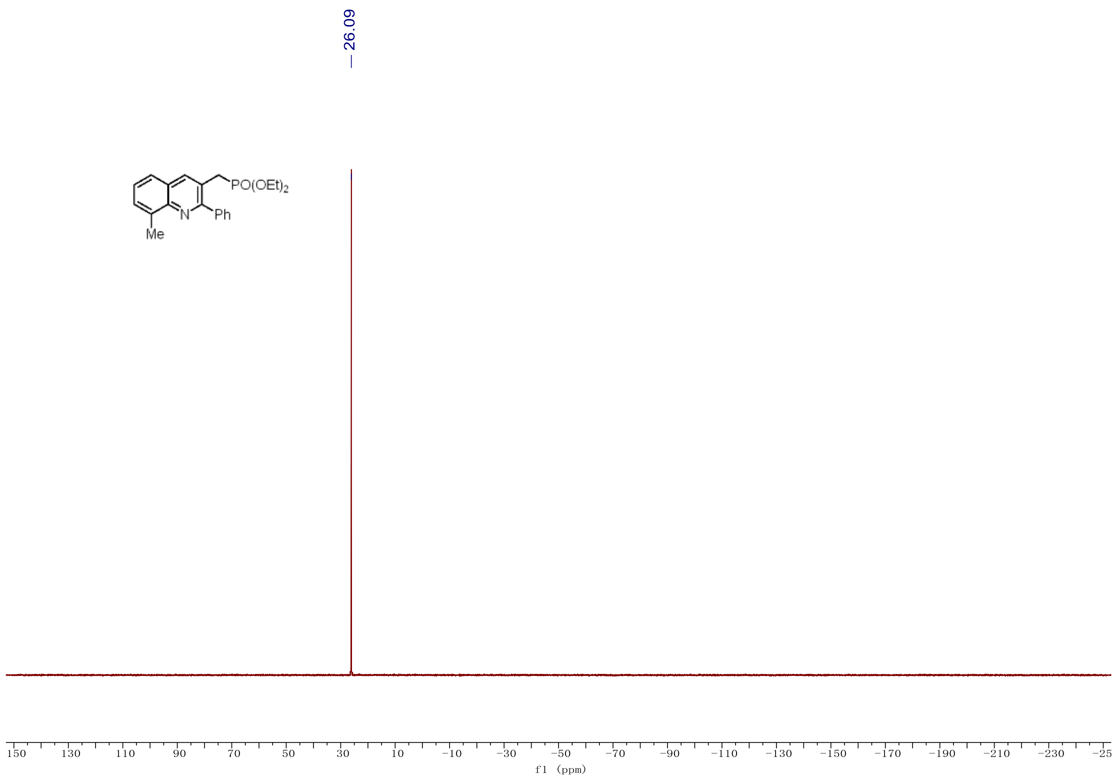
26.09
PO(OEt)2
N
Ph
Me
f1 (ppm)

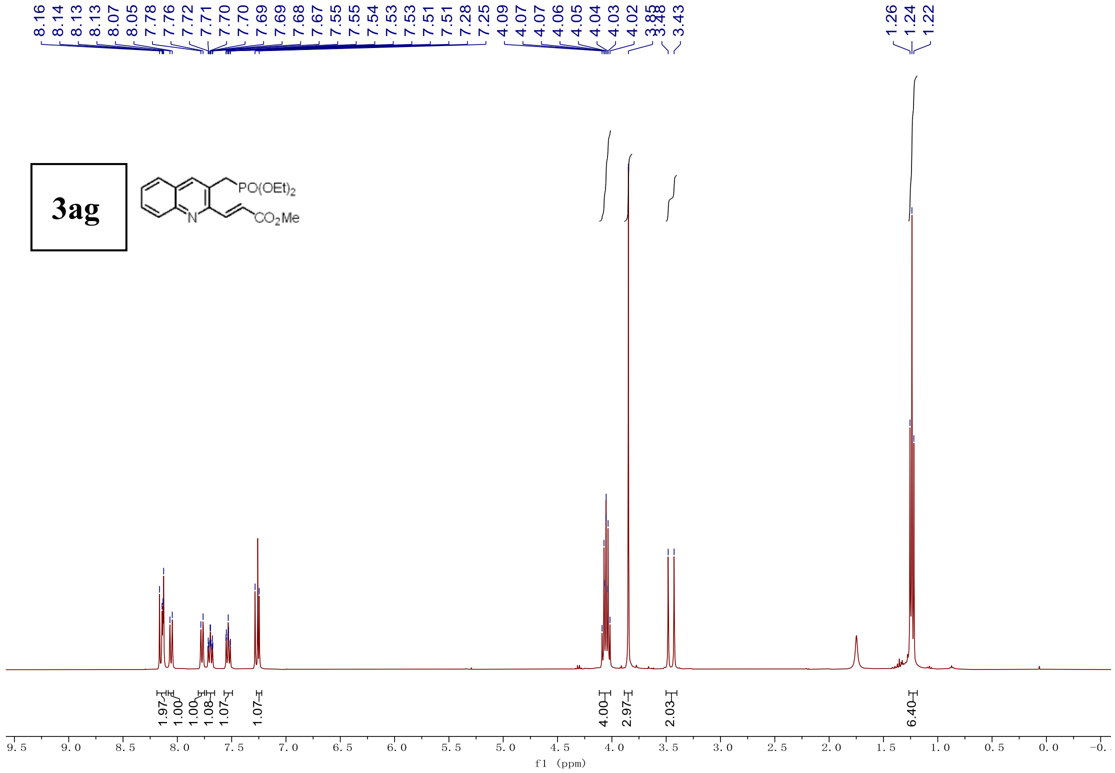
3ag
PO(OEt)2
N
CO2Me
f1 (ppm)

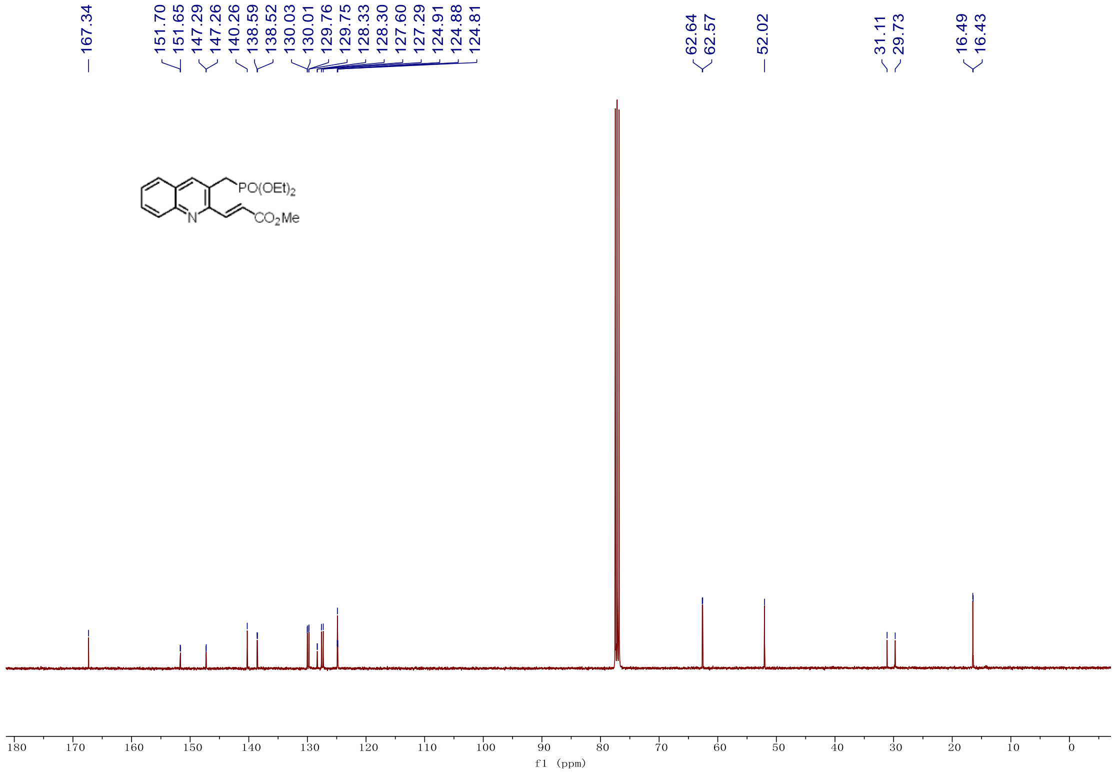
167.34
151.70
151.65
147.29
147.26
140.26
138.59
138.52
130.03
130.01
129.76
129.75
128.33
128.30
127.60
127.29
124.91
124.88
124.81
62.64
62.57
52.02
31.11
29.73
16.49
16.43
PO(OEt)2
N
CO2Me
f1 (ppm)

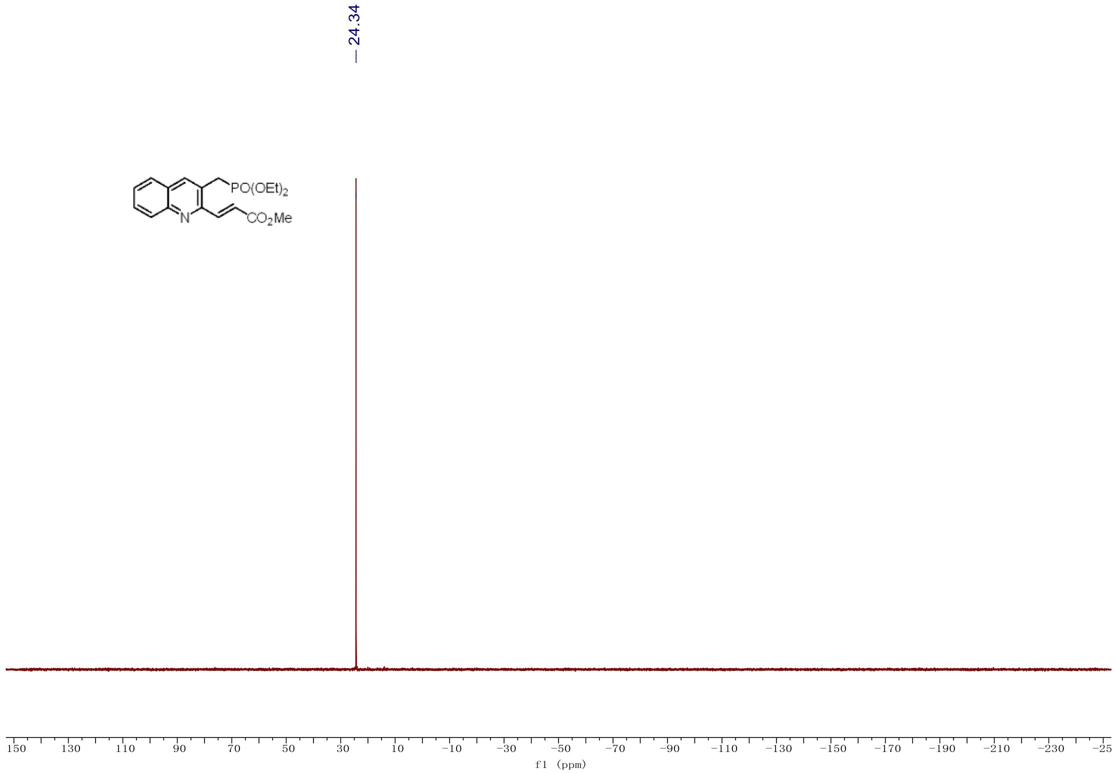
24.34
PO(OEt)2
N
CO2Me
f1 (ppm)

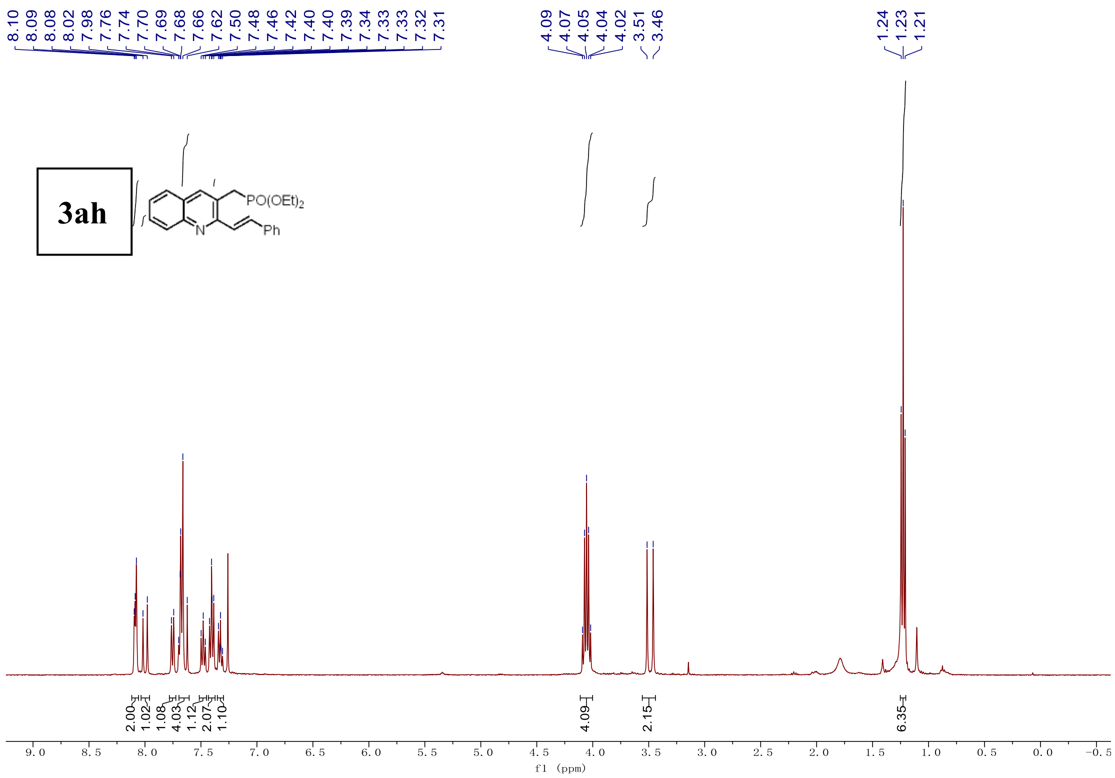
8.10
8.09
8.08
8.02
7.98
7.76
7.74
7.70
7.69
7.68
7.66
7.62
7.50
7.48
7.46
7.42
7.40
7.39
7.34
7.33
7.33
7.32
7.31
4.09
4.07
4.05
4.04
4.02
3.51
3.46
1.24
1.23
1.21
3ah
PO(OEt)2
N
Ph
2.00
1.02
1.08
4.03
1.12
2.07
1.10
4.09
2.15
6.35
f1 (ppm)

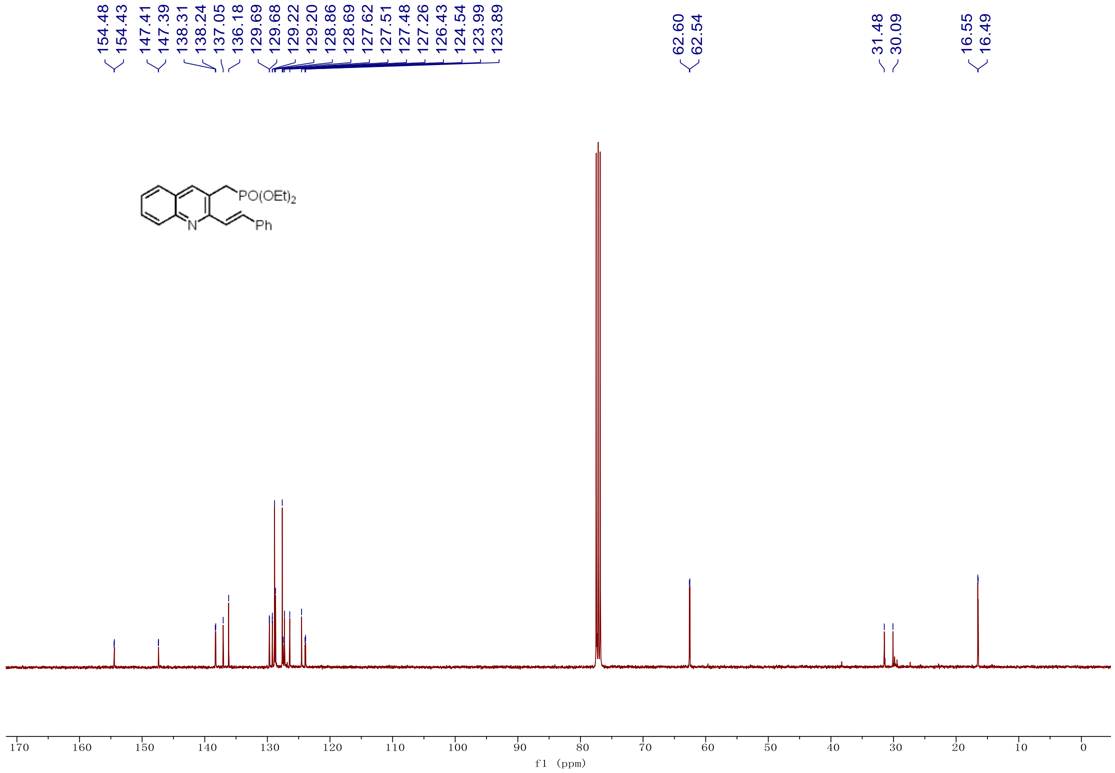
154.48
154.43
147.41
147.39
138.31
138.24
137.05
136.18
129.69
129.68
129.22
129.20
128.86
128.69
127.62
127.51
127.48
127.26
126.43
124.54
123.99
123.89
62.60
62.54
31.48
30.09
16.55
16.49
PO(OEt)2
N
Ph
f1 (ppm)

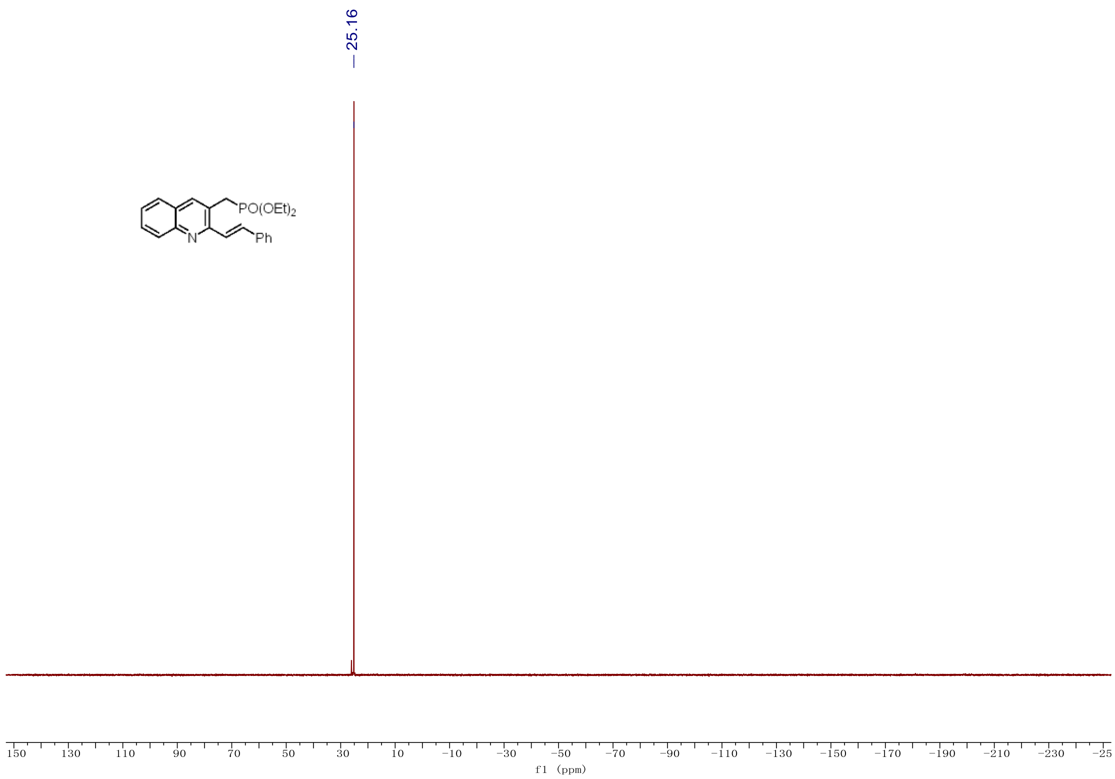
25.16
PO(OEt)2
N
Ph
150 130 110 90 70 50 30 10 −10 −30 −50 −70 −90 −110 −130 −150 −170 −190 −210 −230 −25
f1 (ppm)

3ai

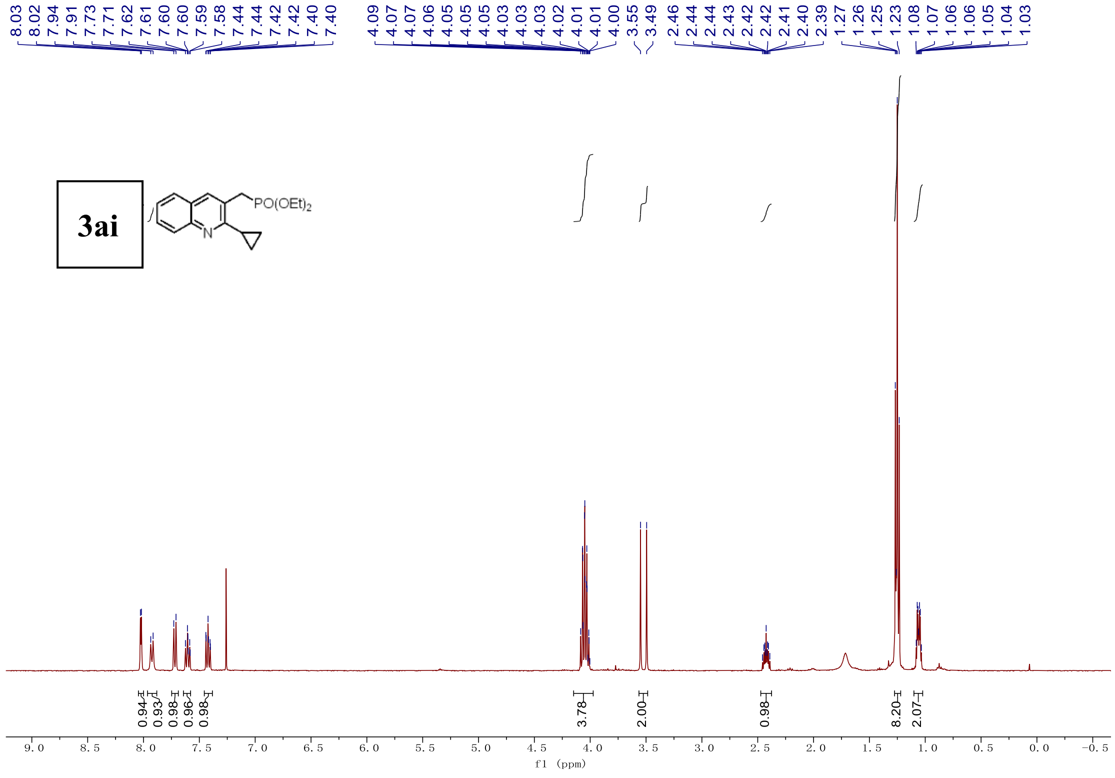
8.03 8.02 7.94 7.91 7.73 7.71 7.62 7.61 7.60 7.60 7.59 7.58 7.44 7.44 7.42 7.42 7.40 7.40
4.09 4.07 4.07 4.06 4.05 4.05 4.05 4.03 4.03 4.03 4.02 4.01 4.01 4.00 3.55 3.49
2.46 2.44 2.43 2.42 2.42 2.42 2.41 2.40 2.39 1.27 1.26 1.25 1.23 1.08 1.07 1.06 1.06 1.05 1.04 1.03
PO(OEt)2
N
0.94 0.93 0.98 0.96 0.98 3.78 2.00 0.98 8.20 2.07
9.0 8.5 8.0 7.5 7.0 6.5 6.0 5.5 5.0 4.5 4.0 3.5 3.0 2.5 2.0 1.5 1.0 0.5 0.0 −0.5
f1 (ppm)

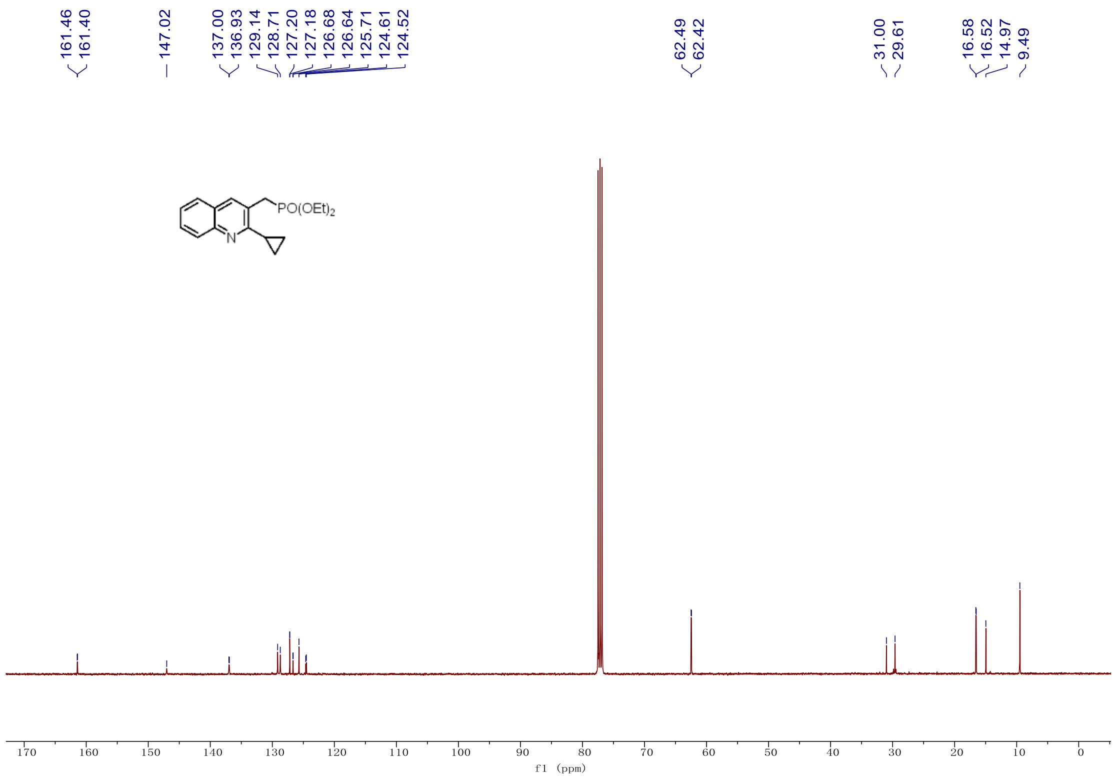
161.46
161.40
147.02
137.00
136.93
129.14
128.71
127.20
127.18
126.68
126.64
125.71
124.61
124.52
62.49
62.42
31.00
29.61
16.58
16.52
14.97
9.49
PO(OEt)2
N
f1 (ppm)

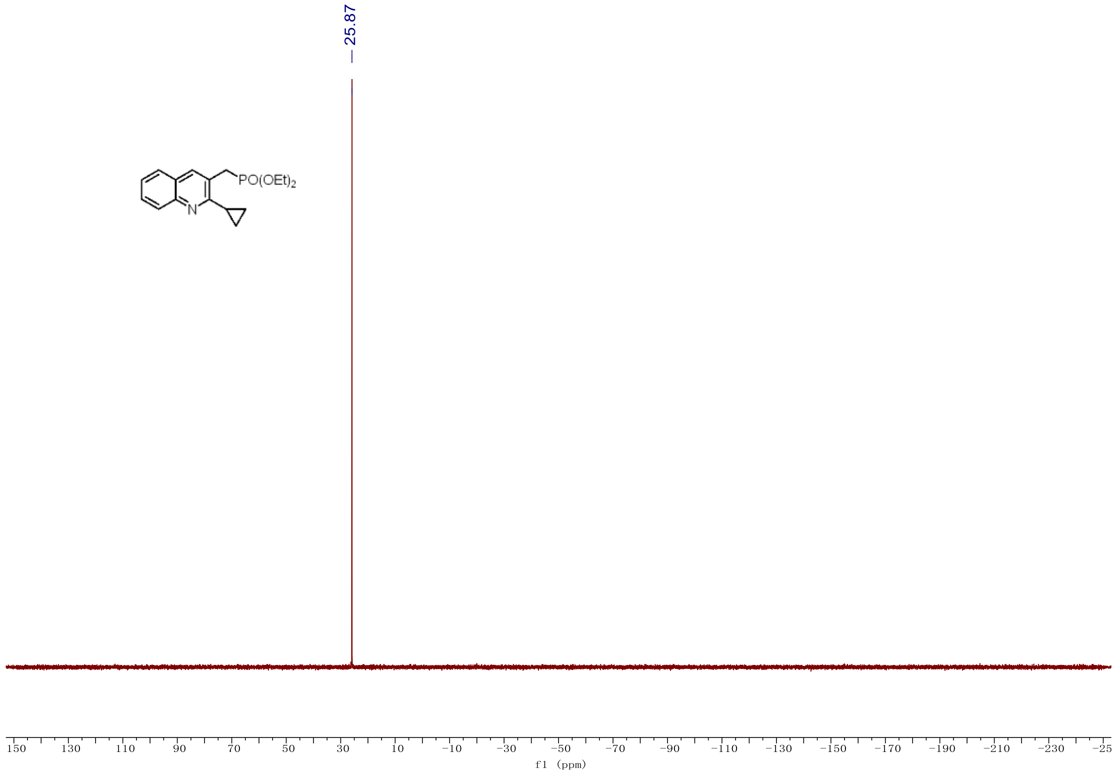
25.87
PO(OEt)2
N
f1 (ppm)

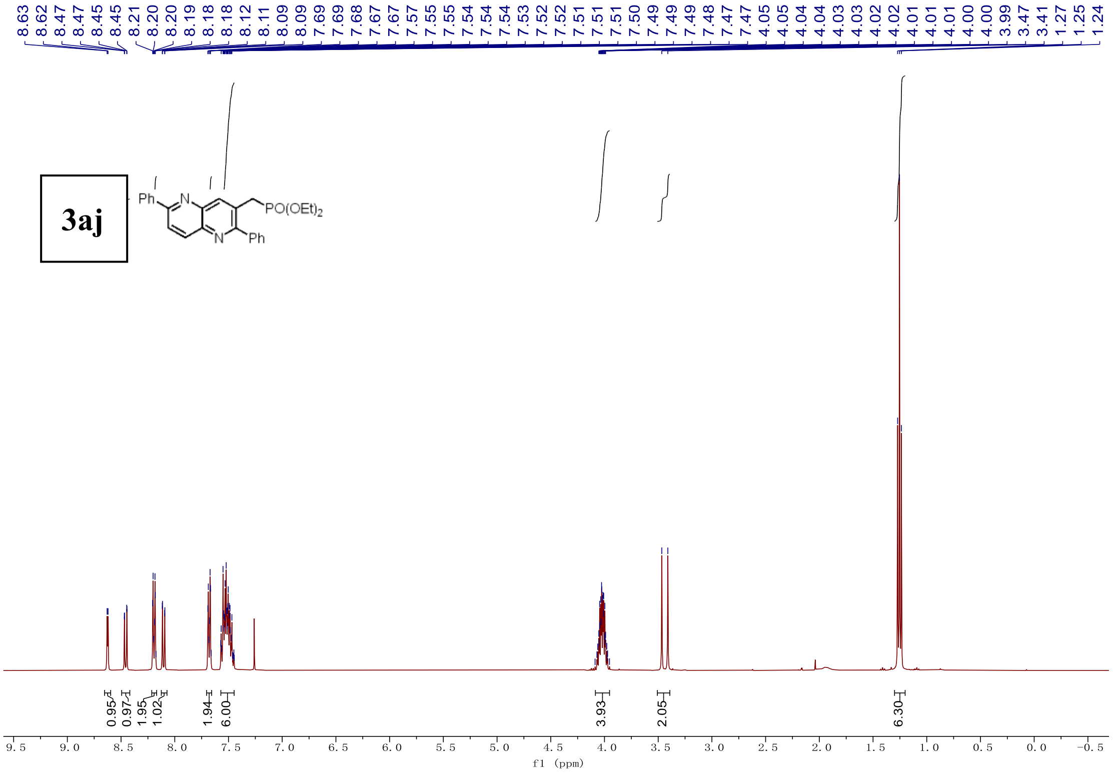
8.63 8.62 8.47 8.45 8.45 8.21 8.20 8.20 8.19 8.18 8.18 8.12 8.11 8.09 8.09 7.69 7.69 7.68 7.67 7.67 7.57 7.55 7.55 7.54 7.54 7.54 7.53 7.52 7.52 7.51 7.51 7.51 7.50 7.49 7.49 7.48 7.47 7.47 4.05 4.05 4.04 4.04 4.03 4.03 4.02 4.02 4.01 4.01 4.01 4.00 3.99 3.47 3.41 1.27 1.25 1.24
3aj
Ph
N
N
Ph
PO(OEt)2
0.95
0.97
1.95
1.02
1.94
6.00
3.93
2.05
6.30
9.5 9.0 8.5 8.0 7.5 7.0 6.5 6.0 5.5 5.0 4.5 4.0 3.5 3.0 2.5 2.0 1.5 1.0 0.5 0.0 -0.5
f1 (ppm)
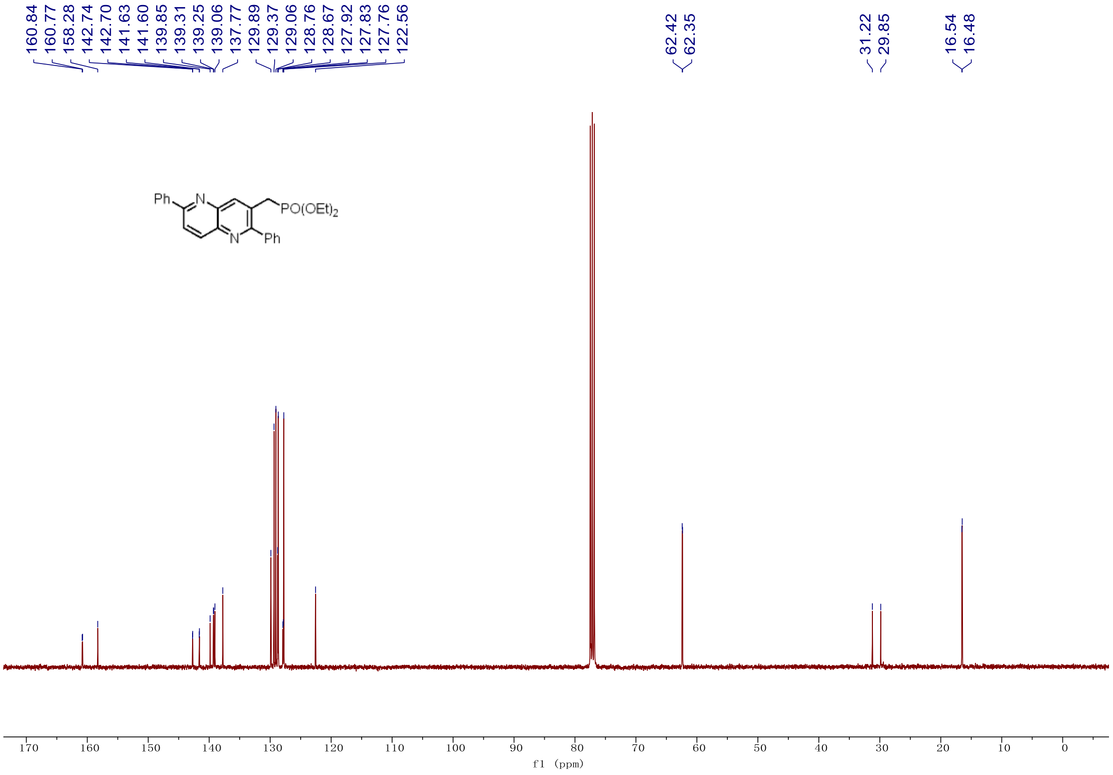
160.84 160.77 158.28 142.74 142.70 141.63 141.60 139.85 139.31 139.25 139.06 137.77 129.89 129.37 129.06 128.76 128.67 127.92 127.83 127.76 122.56
62.42 62.35
31.22 29.85
16.54 16.48
Ph
N
N
Ph
PO(OEt)2
170 160 150 140 130 120 110 100 90 80 70 60 50 40 30 20 10 0
f1 (ppm)

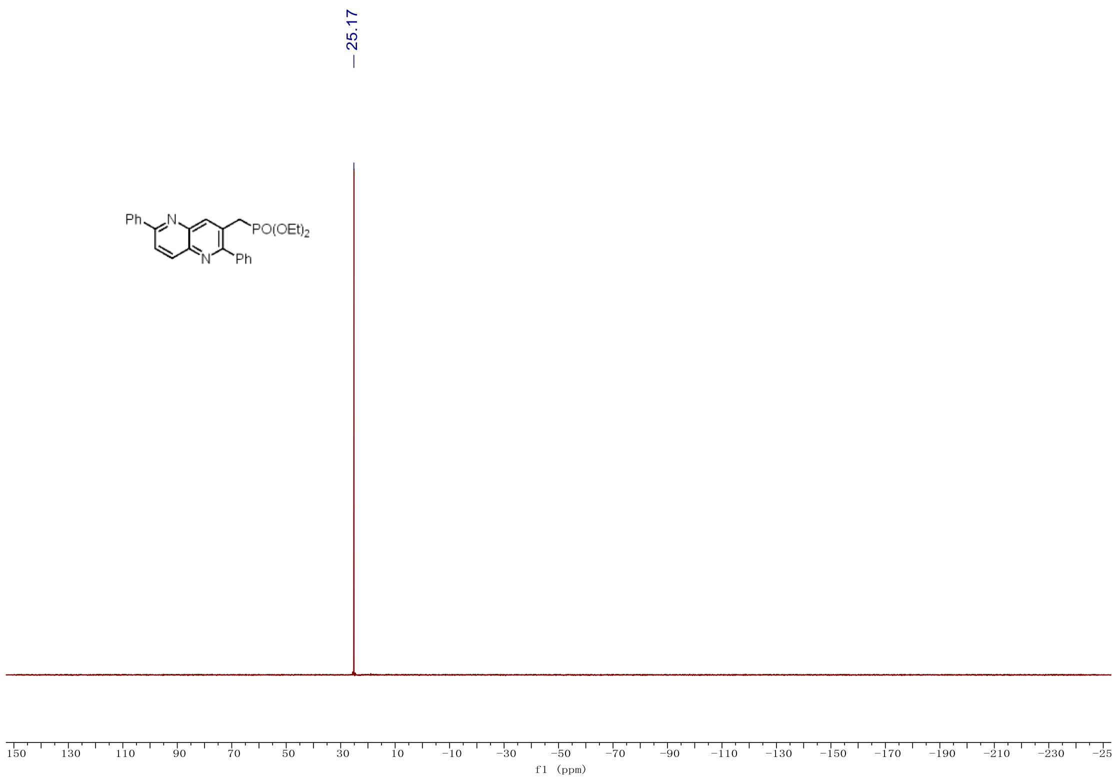

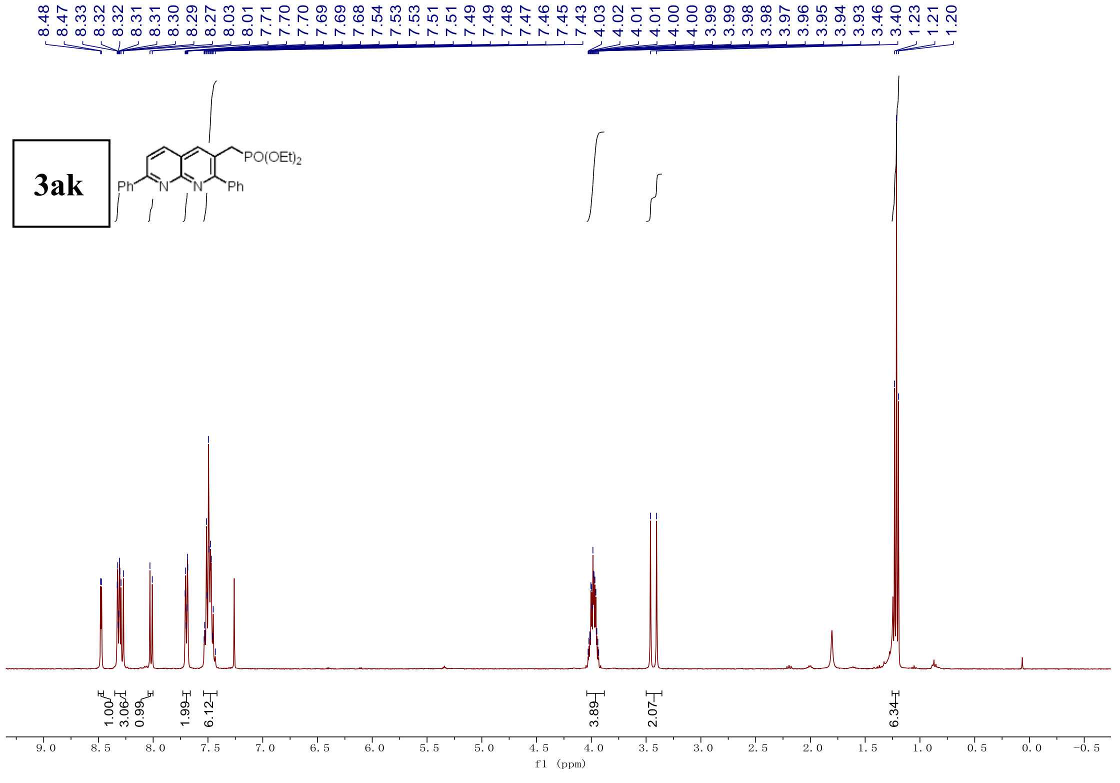

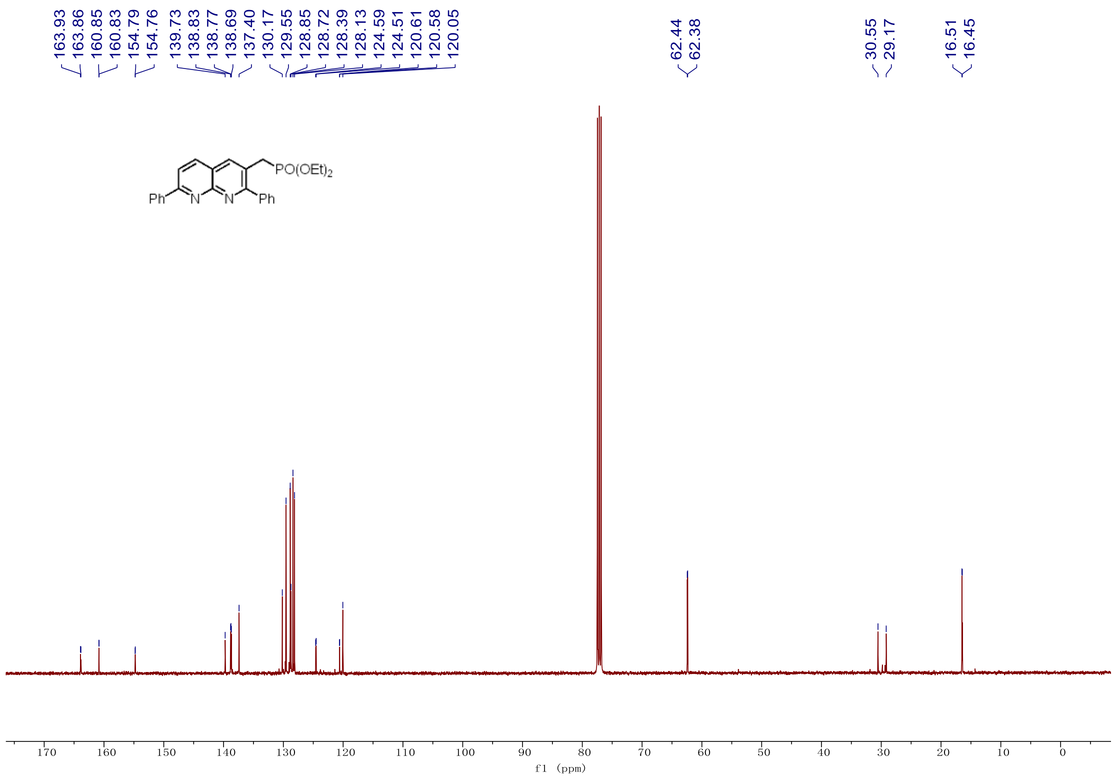

163.93
163.86
160.85
160.83
154.79
154.76
139.73
138.83
138.77
138.69
137.40
130.17
129.55
128.85
128.72
128.39
128.13
124.59
124.51
120.61
120.58
120.05
62.44
62.38
30.55
29.17
16.51
16.45
PO(OEt)2
Ph
N
N
Ph
f1 (ppm)

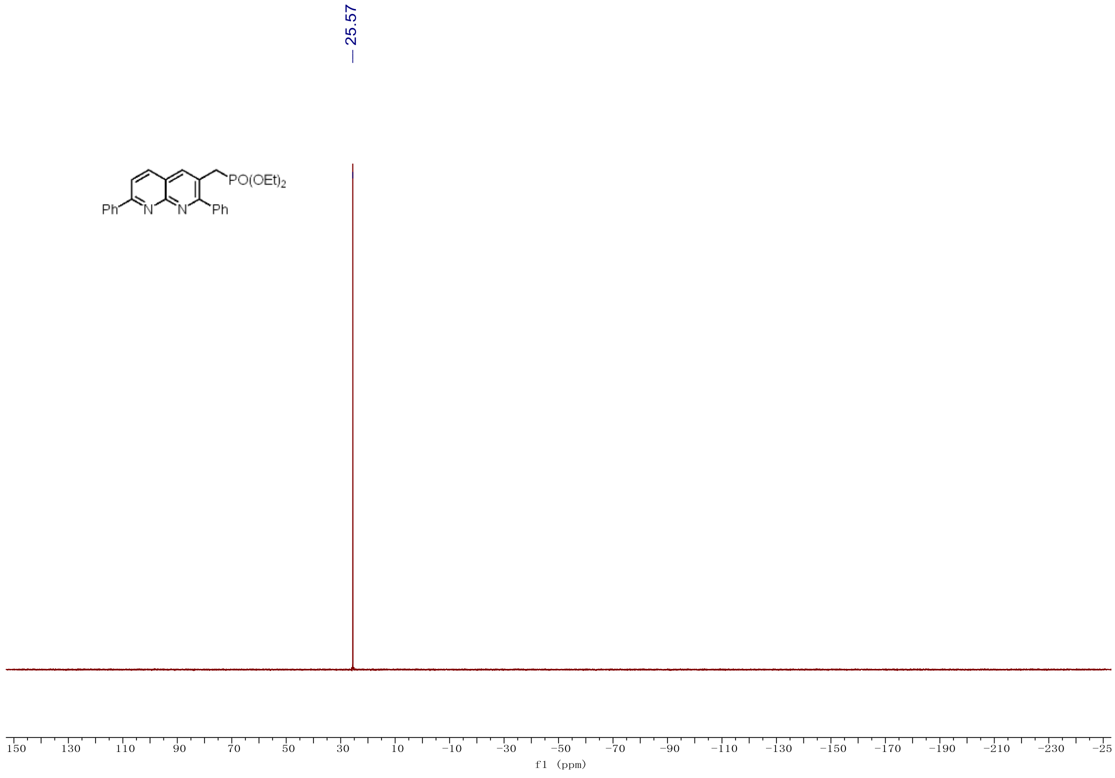

25.57
PO(OEt)2
Ph
N
N
Ph
f1 (ppm)

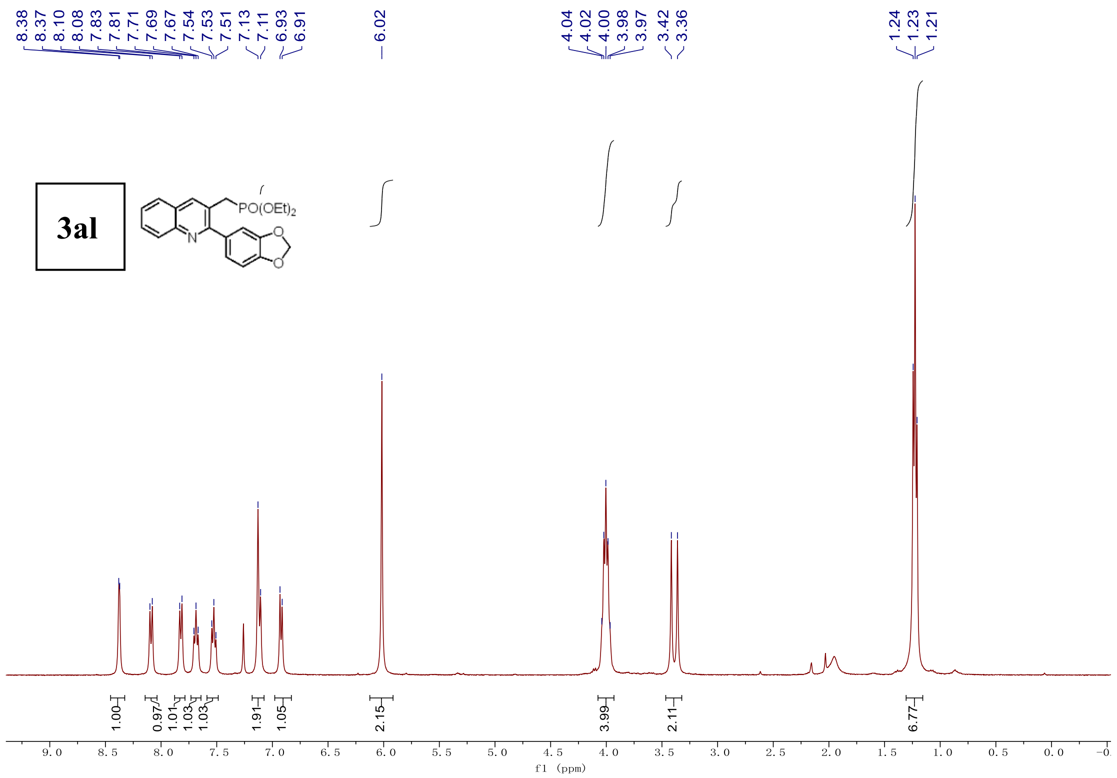
3al
PO(OEt)2
f1 (ppm)

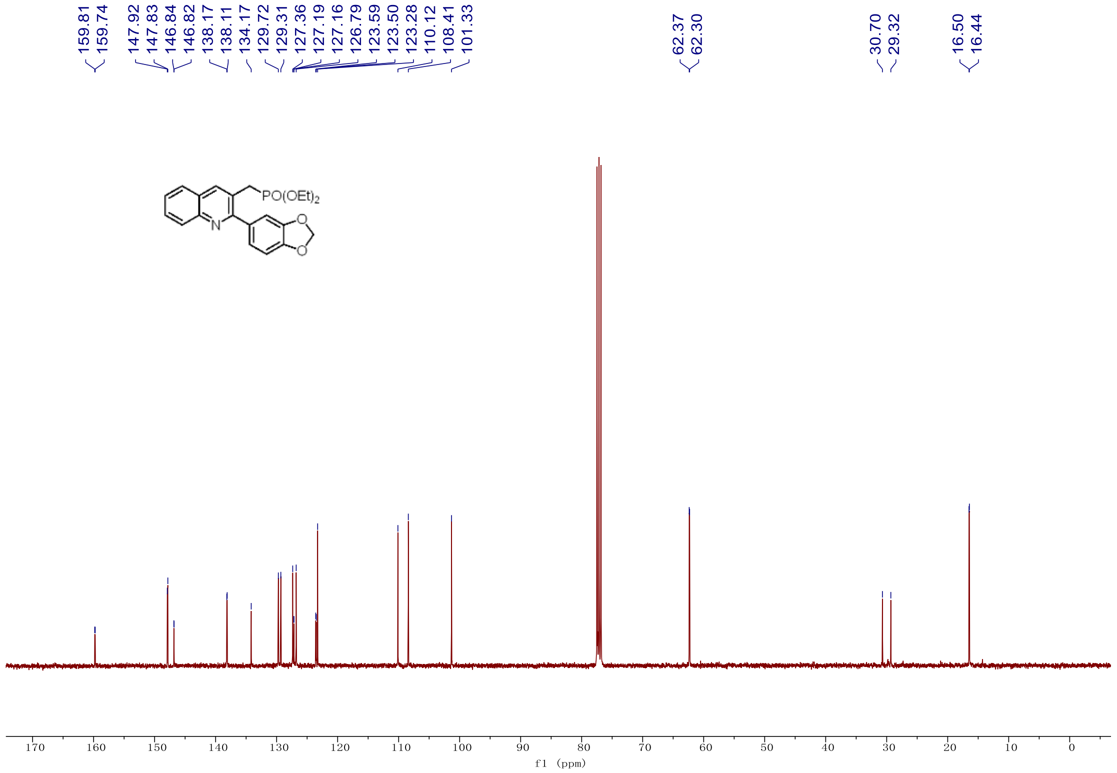
PO(OEt)2
f1 (ppm)

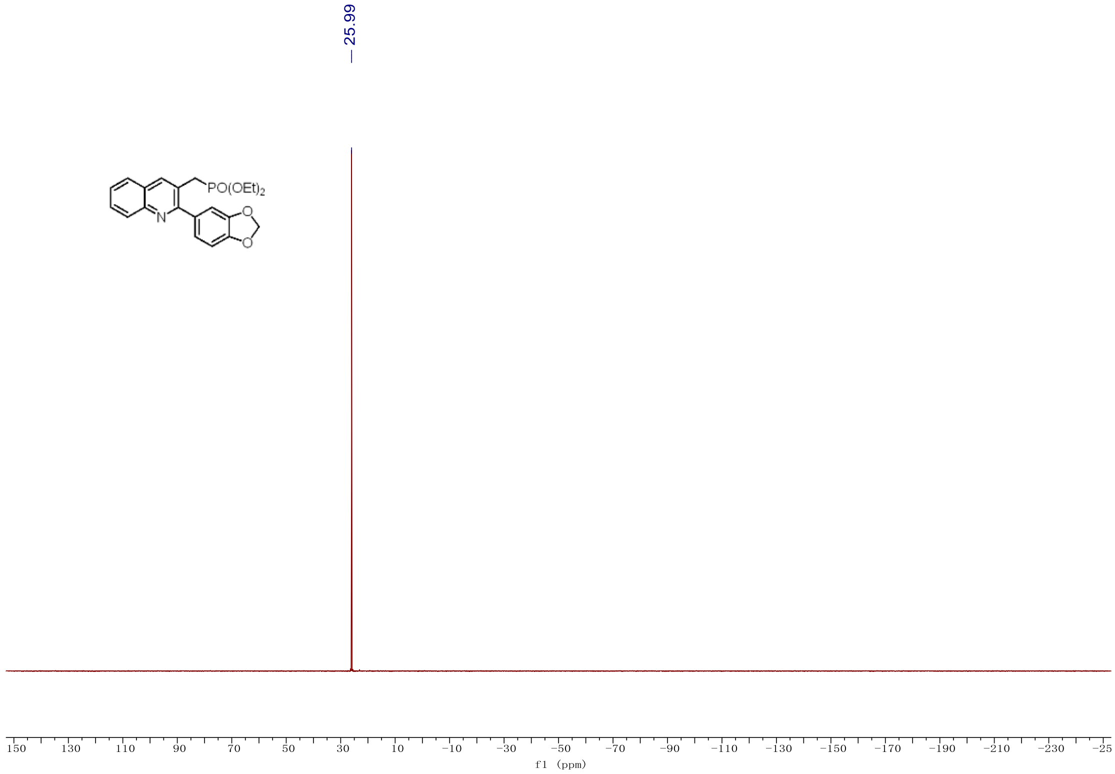
25.99
PO(OEt)2
N
O
O
150 130 110 90 70 50 30 10 −10 −30 −50 −70 −90 −110 −130 −150 −170 −190 −210 −230 −25
f1 (ppm)

3am

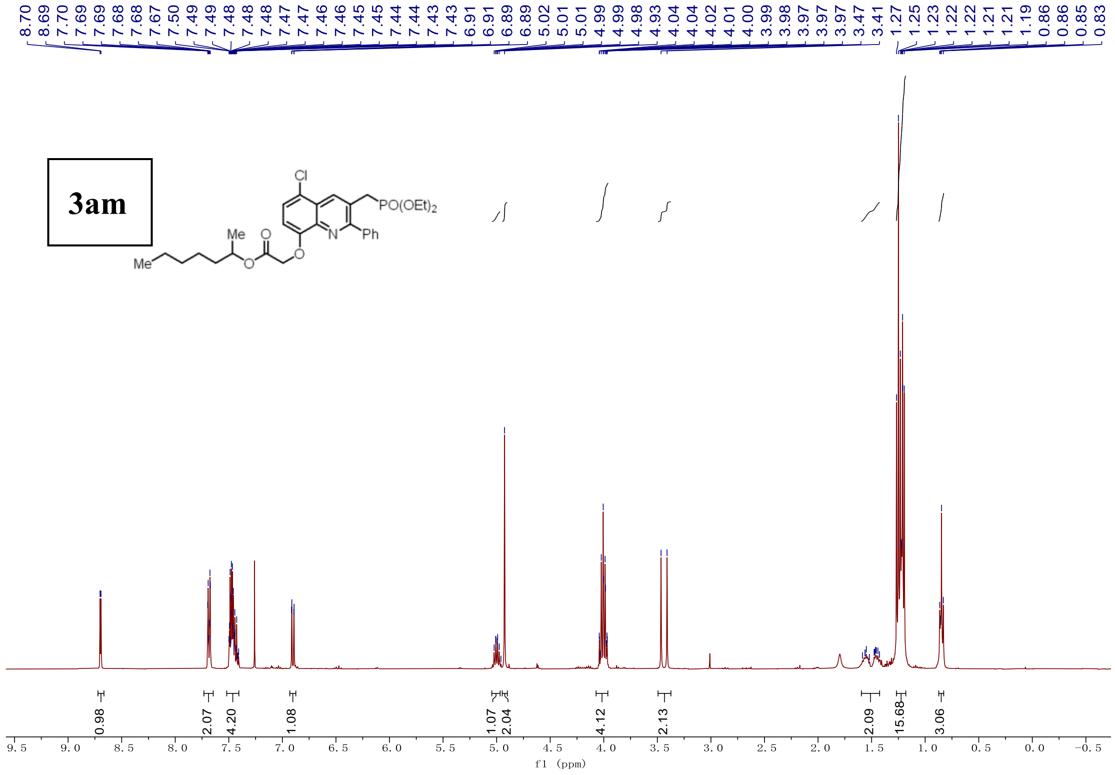
8.70 8.69 7.70 7.69 7.69 7.68 7.68 7.67 7.50 7.49 7.49 7.48 7.48 7.48 7.47 7.47 7.46 7.46 7.45 7.45 7.44 7.44 7.43 7.43 6.91 6.91 6.89 6.89 5.02 5.01 5.01 4.99 4.99 4.98 4.93 4.04 4.04 4.02 4.01 4.00 3.99 3.98 3.97 3.97 3.97 3.47 3.41 1.27 1.25 1.23 1.22 1.22 1.21 1.21 1.19 0.86 0.86 0.85 0.83
Cl
PO(OEt)2
Me
O
Me
O
O
N
Ph
0.98 2.07 4.20 1.08 1.07 2.04 4.12 2.13 2.09 15.68 3.06
9.5 9.0 8.5 8.0 7.5 7.0 6.5 6.0 5.5 5.0 4.5 4.0 3.5 3.0 2.5 2.0 1.5 1.0 0.5 0.0 −0.5
f1 (ppm)

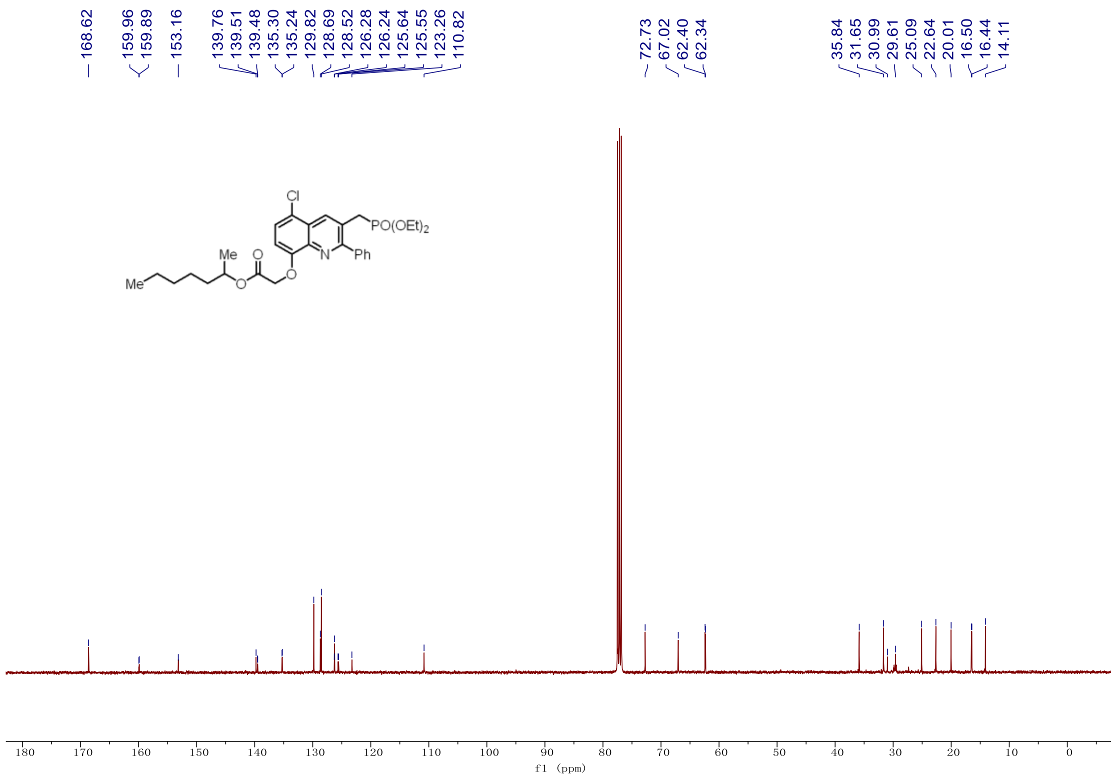
168.62
159.96
159.89
153.16
139.76
139.51
139.48
135.30
135.24
129.82
128.69
128.52
126.28
126.24
125.64
125.55
123.26
110.82
72.73
67.02
62.40
62.34
35.84
31.65
30.99
29.61
25.09
22.64
20.01
16.50
16.44
14.11
Cl
PO(OEt)2
Me
O
N
Ph
Me
O
O
f1 (ppm)

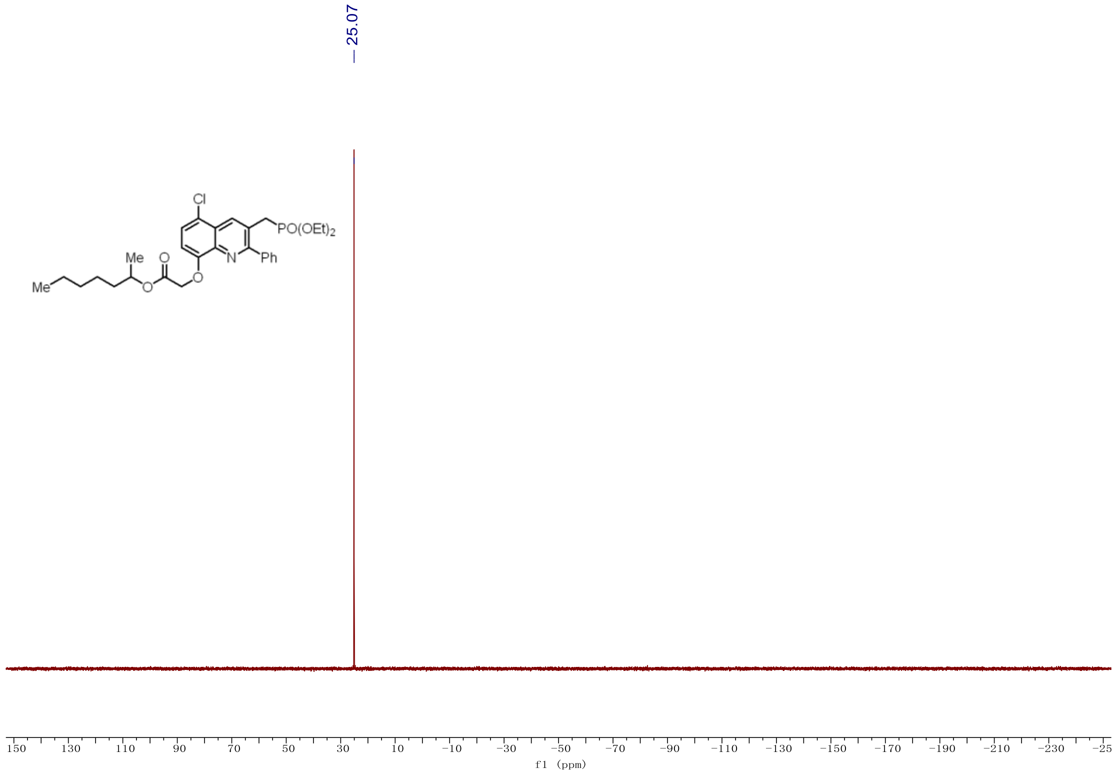
25.07
Cl
PO(OEt)2
Me
O
N
Ph
Me
O
O
f1 (ppm)

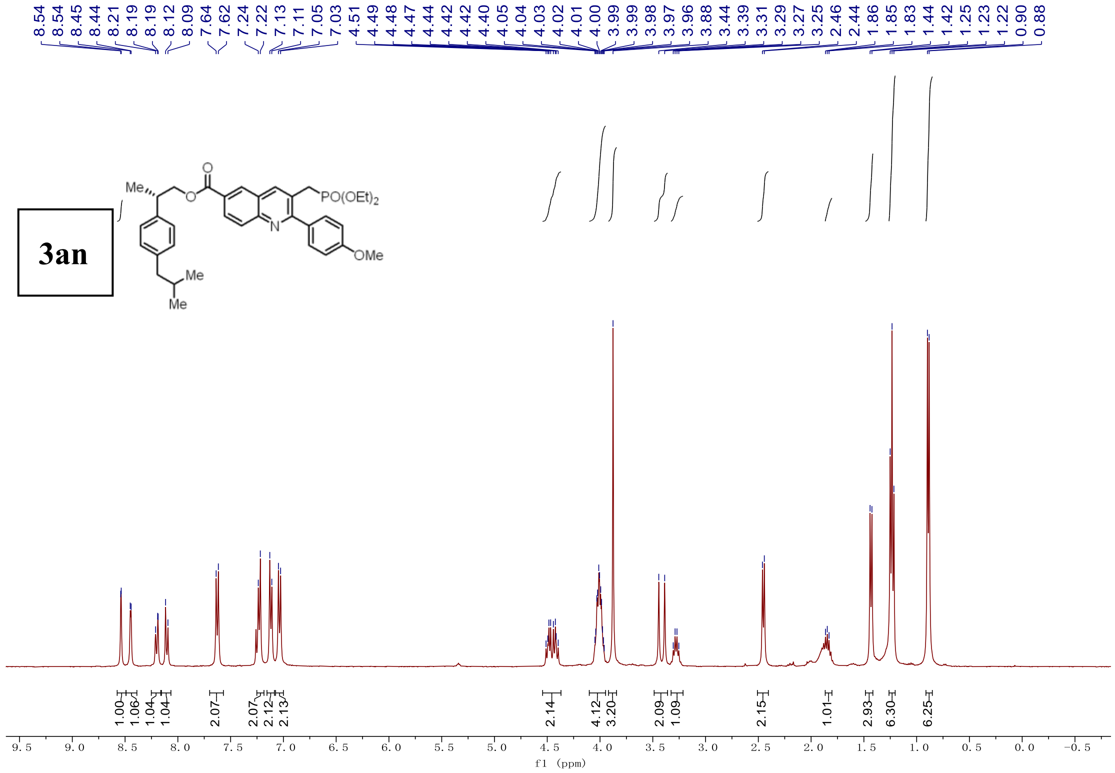
8.54 8.54 8.45 8.44 8.21 8.19 8.19 8.12 8.09 7.64 7.62 7.24 7.22 7.13 7.11 7.05 7.03 4.51 4.49 4.48 4.47 4.44 4.42 4.42 4.40 4.05 4.04 4.03 4.02 4.01 4.00 3.99 3.98 3.97 3.96 3.88 3.44 3.39 3.31 3.29 3.27 3.25 2.46 2.44 1.86 1.85 1.83 1.44 1.42 1.25 1.23 1.22 0.90 0.88
3an
Me
O
PO(OEt)2
N
OMe
Me
Me
1.00 1.06 1.04 1.04 2.07 2.07 2.12 2.13 2.14 4.12 3.20 2.09 1.09 2.15 1.01 2.93 6.30 6.25
f1 (ppm)

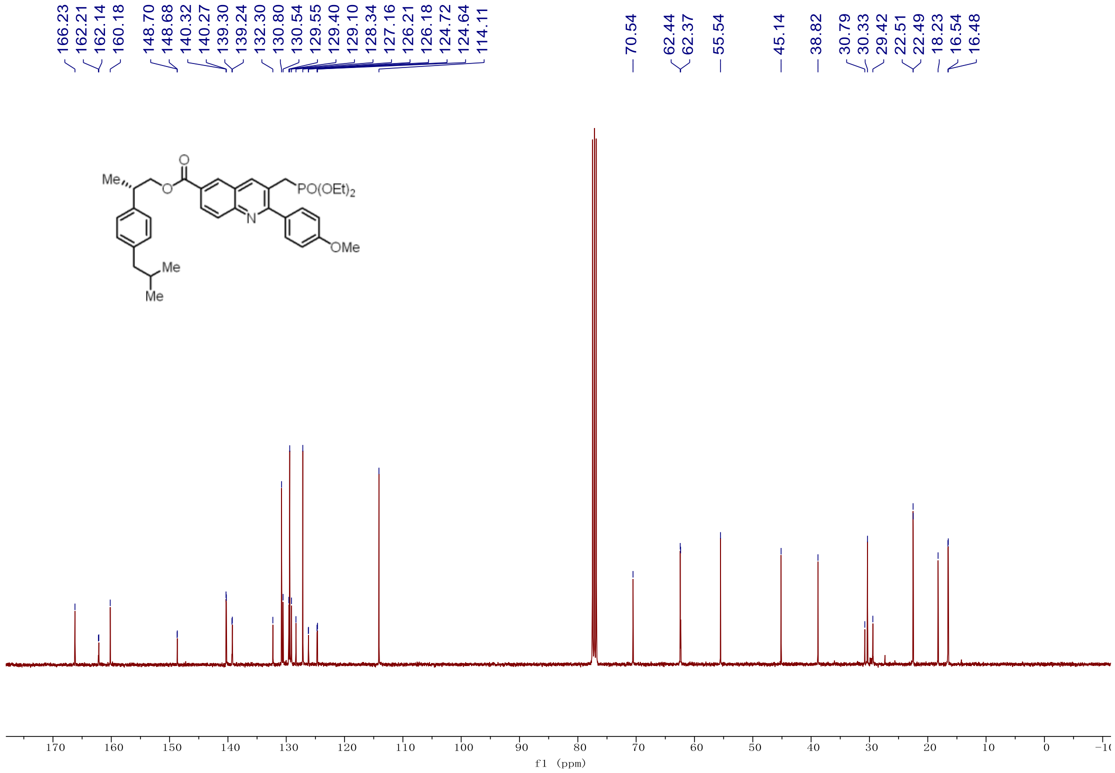
166.23 162.21 162.14 160.18 148.70 148.68 140.32 140.27 139.30 139.24 132.30 130.80 130.54 129.55 129.40 129.10 128.34 127.16 126.21 126.18 124.72 124.64 114.11 70.54 62.44 62.37 55.54 45.14 38.82 30.79 30.33 29.42 22.51 22.49 18.23 16.54 16.48
Me
O
PO(OEt)2
N
OMe
Me
Me
f1 (ppm)

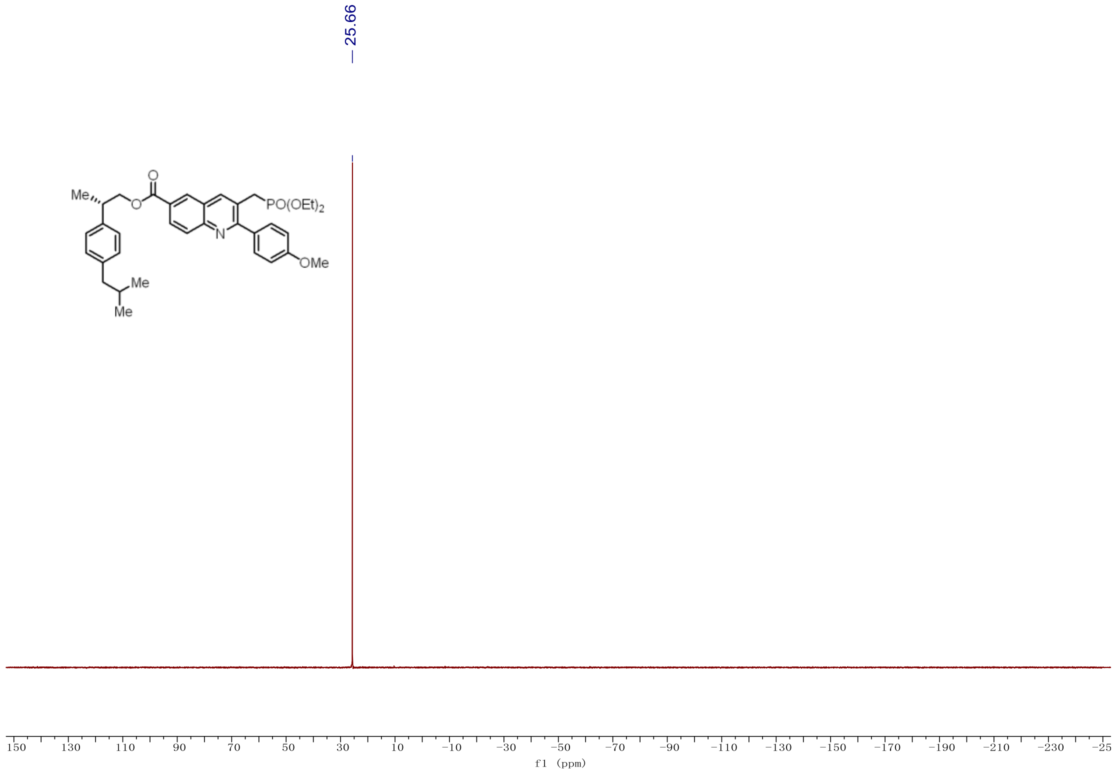

25.66
150 130 110 90 70 50 30 10 −10 −30 −50 −70 −90 −110 −130 −150 −170 −190 −210 −230 −25
f1 (ppm)

3ao

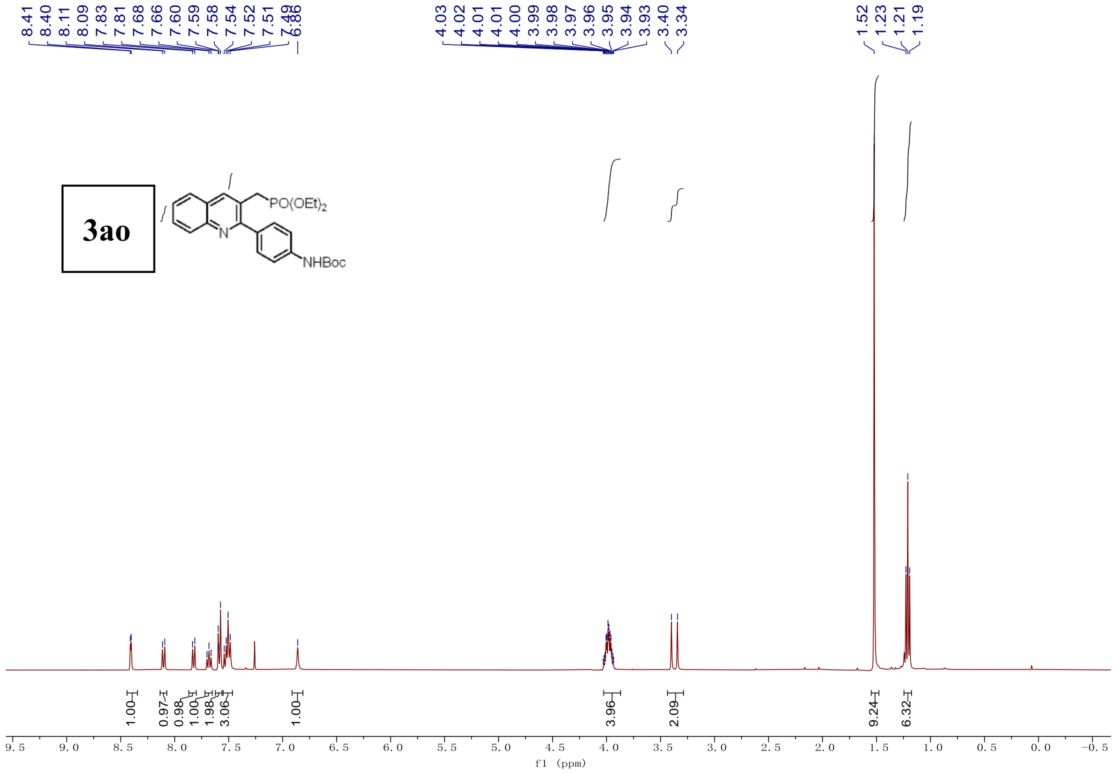

8.41 8.40 8.11 8.09 7.83 7.81 7.68 7.66 7.60 7.59 7.58 7.54 7.52 7.51 7.49 6.86
4.03 4.02 4.01 4.01 4.00 3.99 3.98 3.97 3.96 3.95 3.94 3.93 3.40 3.34
1.52 1.23 1.21 1.19
1.00 0.97 0.98 1.00 1.98 3.06 1.00 3.96 2.09 9.24 6.32
9.5 9.0 8.5 8.0 7.5 7.0 6.5 6.0 5.5 5.0 4.5 4.0 3.5 3.0 2.5 2.0 1.5 1.0 0.5 0.0 -0.5
f1 (ppm)

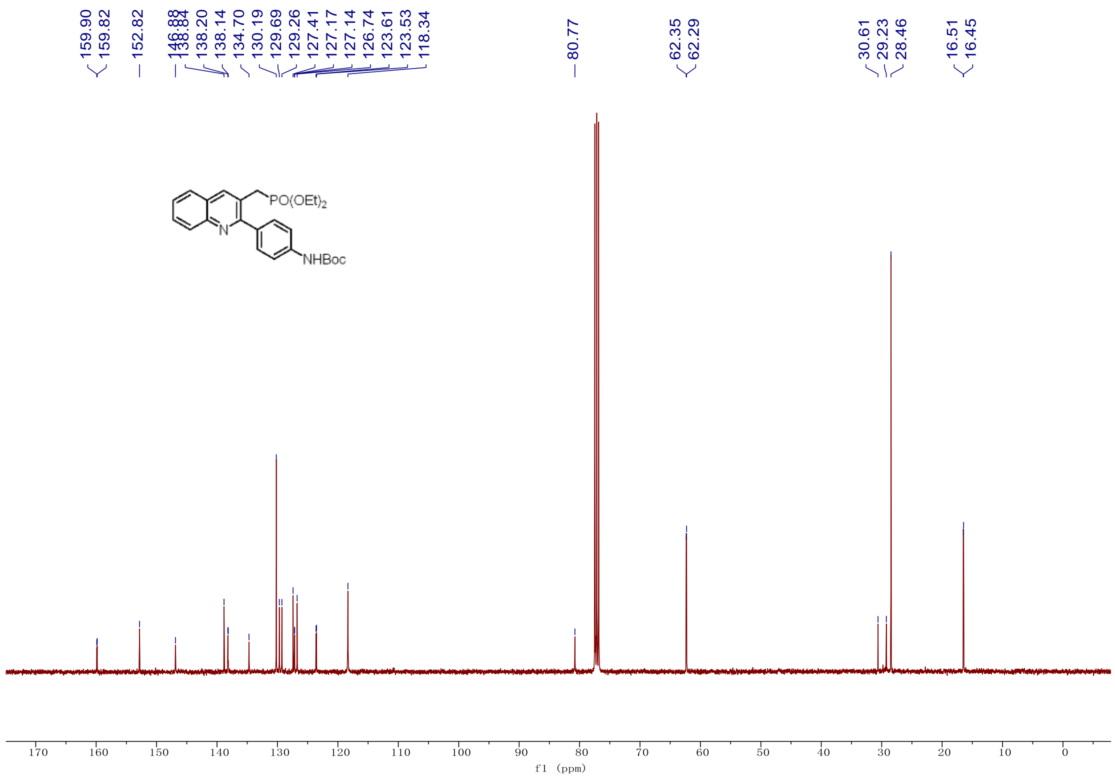
159.90
159.82
152.82
146.88
138.84
138.20
138.14
134.70
130.19
129.69
129.26
127.41
127.17
127.14
126.74
123.61
123.53
118.34
80.77
62.35
62.29
30.61
29.23
28.46
16.51
16.45
PO(OEt)2
N
NHBoc
f1 (ppm)

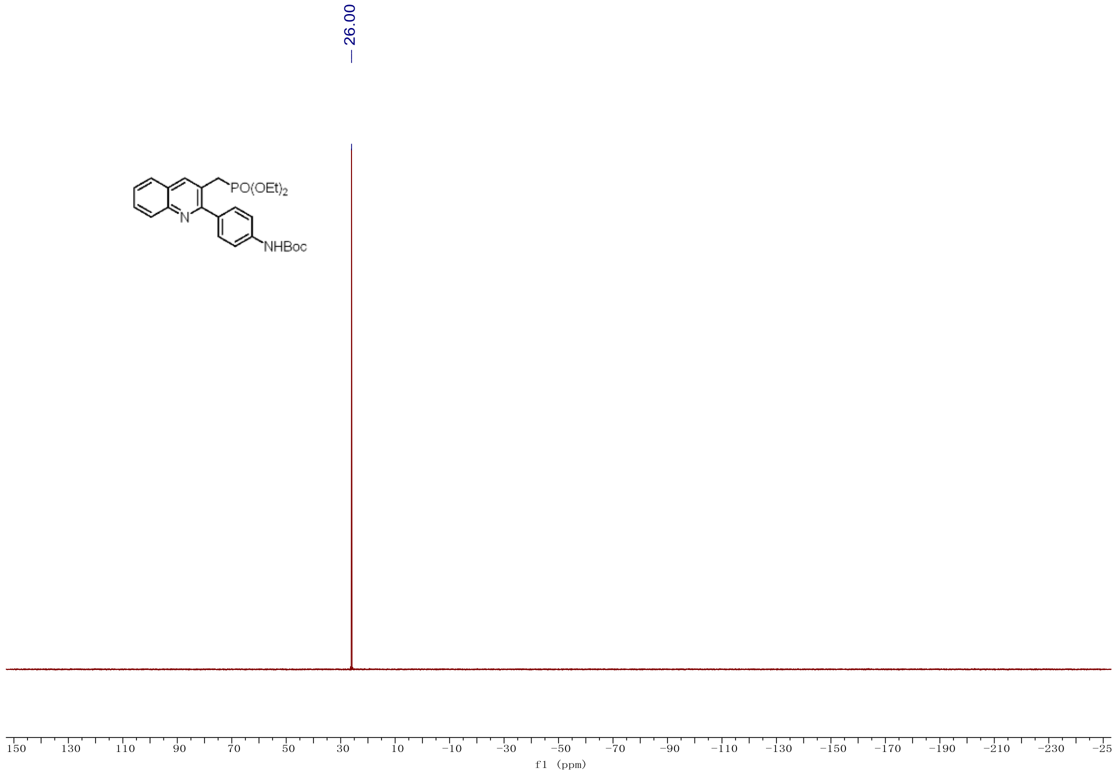
26.00
PO(OEt)2
N
NHBoc
f1 (ppm)

3ap

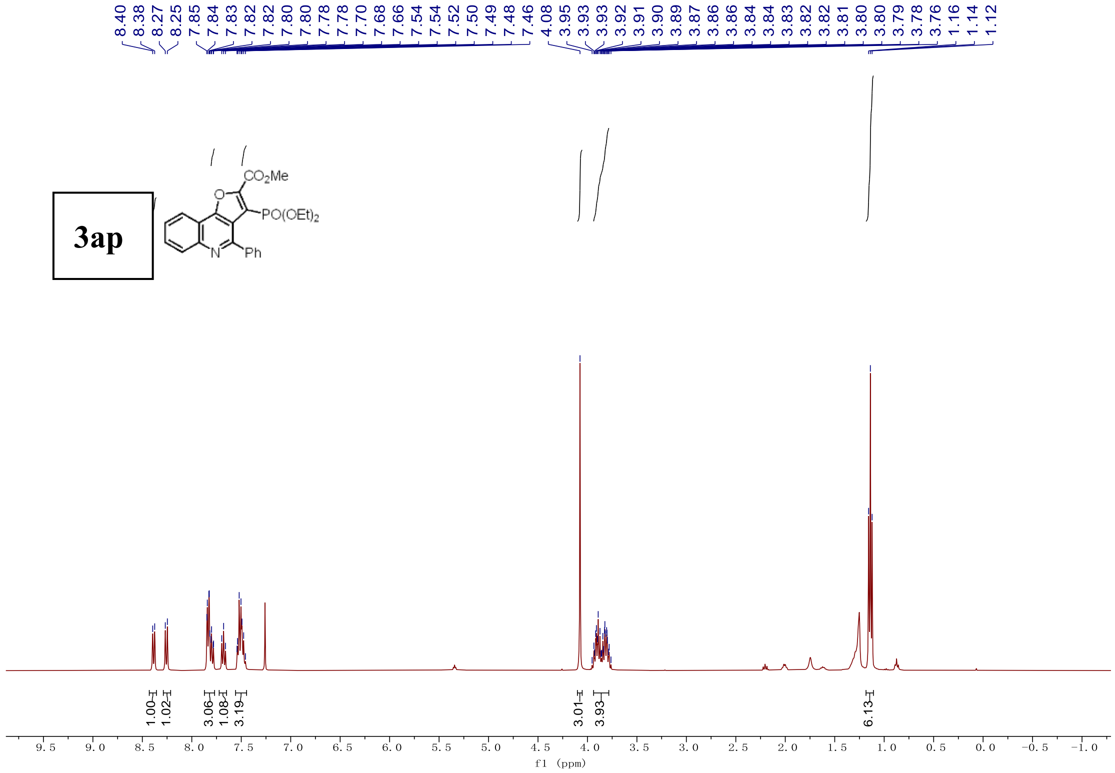

8.40 8.38 8.27 8.25 7.85 7.84 7.83 7.82 7.82 7.80 7.78 7.78 7.70 7.68 7.66 7.54 7.52 7.50 7.49 7.48 7.46 4.08 3.95 3.93 3.93 3.92 3.91 3.90 3.89 3.87 3.86 3.86 3.84 3.84 3.83 3.82 3.82 3.81 3.80 3.80 3.79 3.78 3.76 1.16 1.14 1.12
CO2Me
PO(OEt)2
N
O
Ph
1.00
1.02
3.06
1.08
3.19
3.01
3.93
6.13
f1 (ppm)

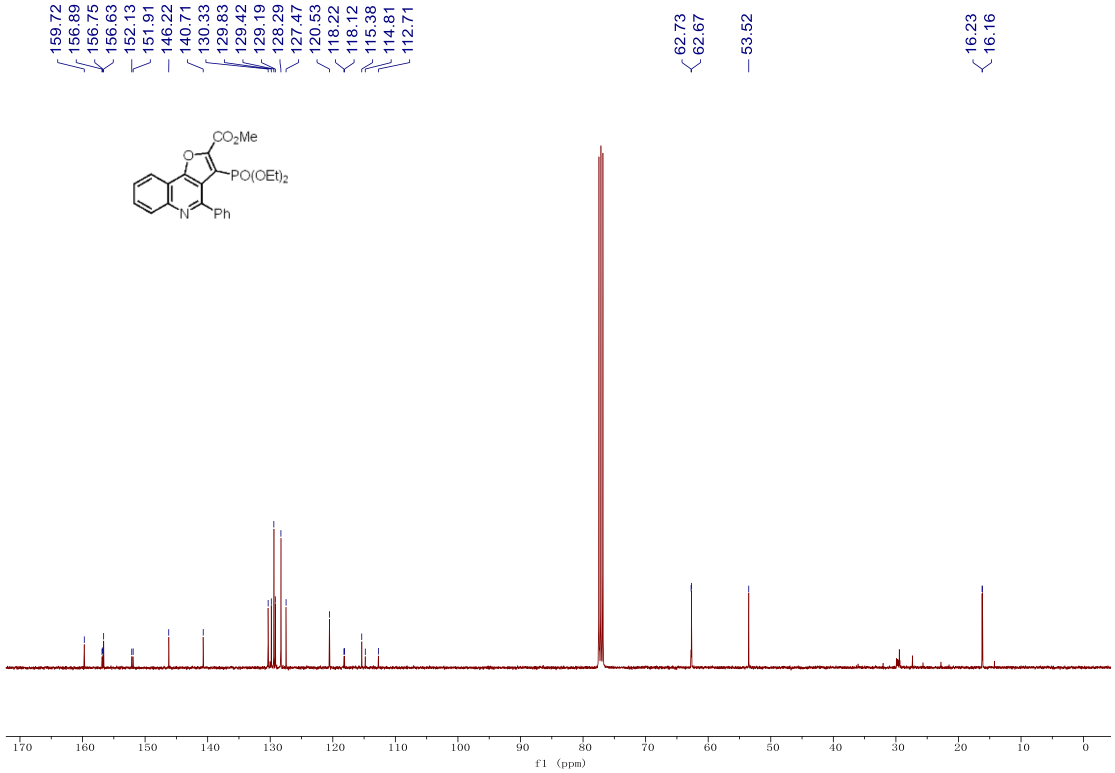

159.72 156.89 156.75 156.63 152.13 151.91 146.22 140.71 130.33 129.83 129.42 129.19 128.29 127.47 120.53 118.22 118.12 115.38 114.81 112.71 62.73 62.67 53.52 16.23 16.16
CO2Me
PO(OEt)2
N
O
Ph
f1 (ppm)

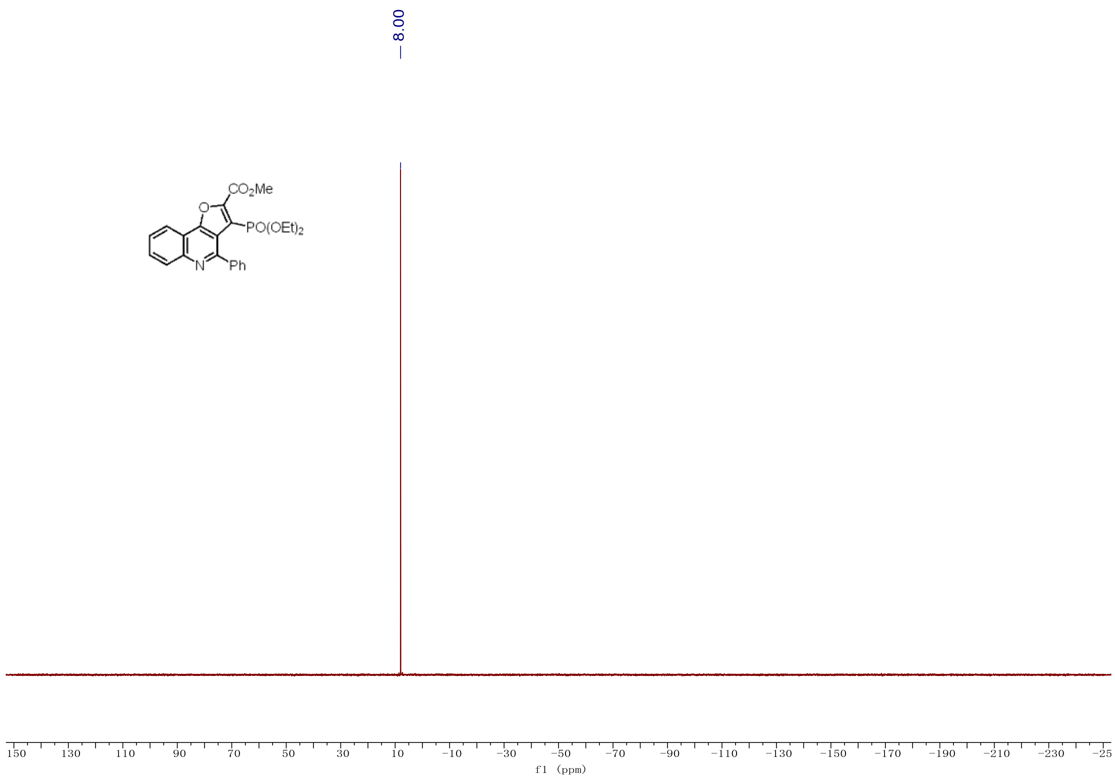

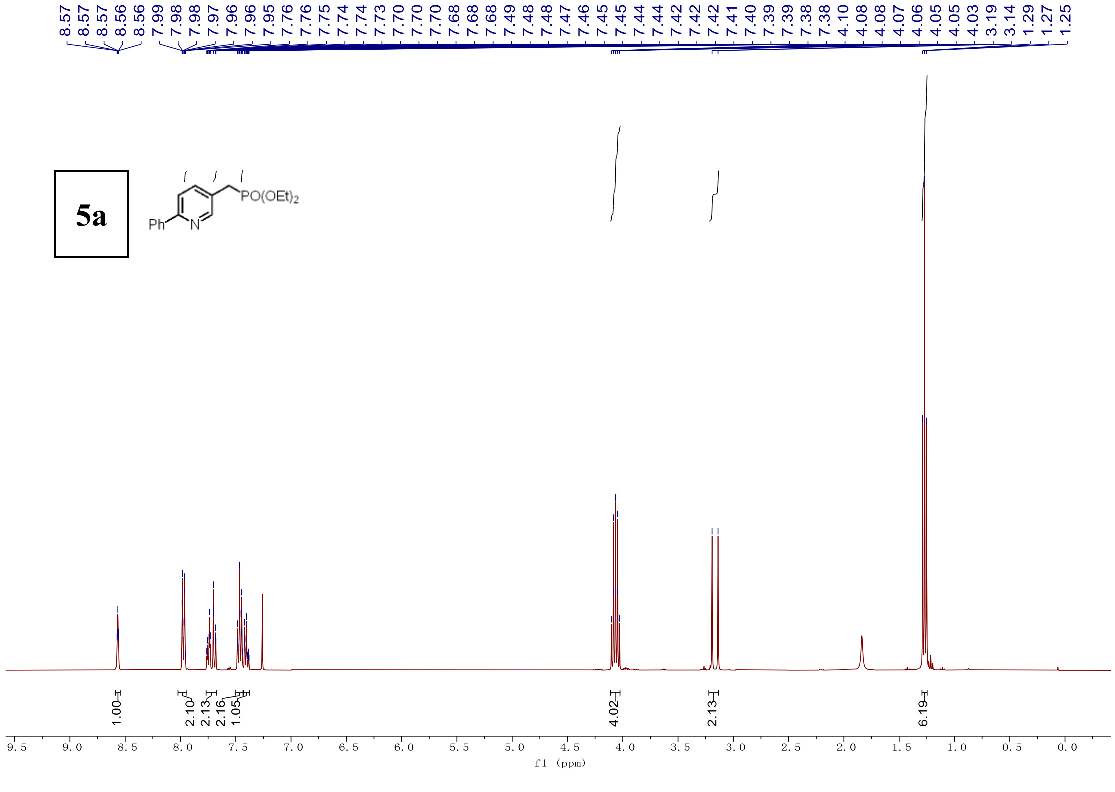

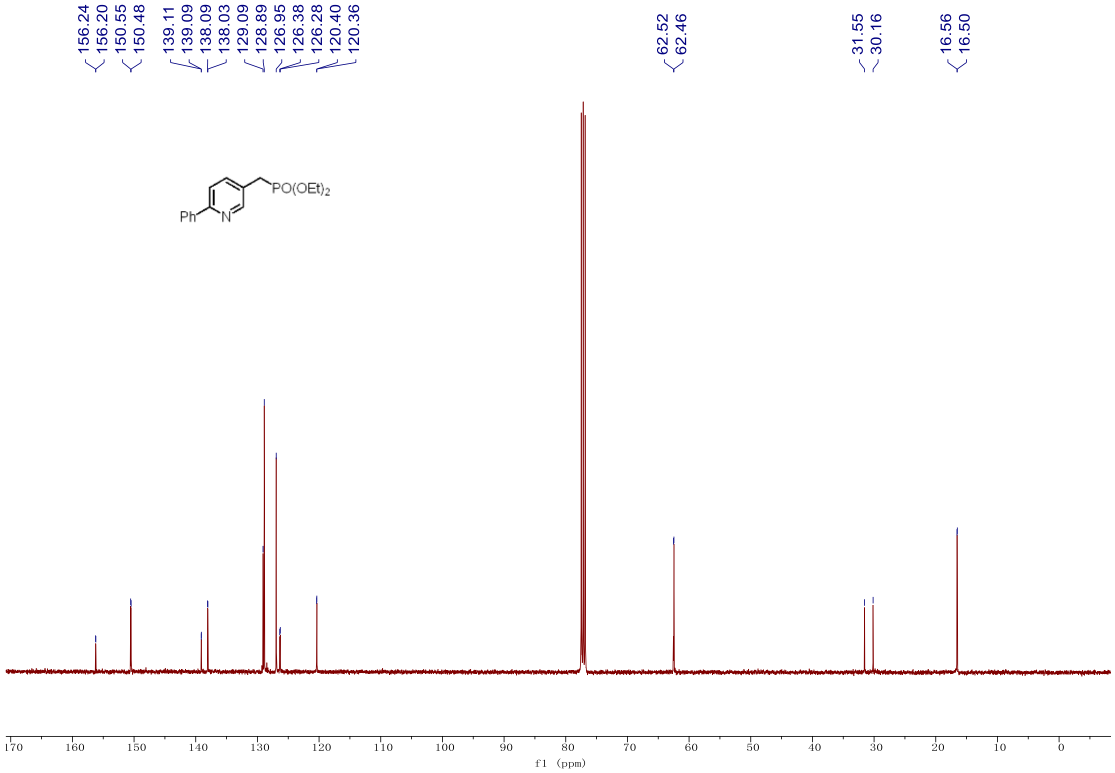
156.24
156.20
150.55
150.48
139.11
139.09
138.09
138.03
129.09
128.89
126.95
126.38
126.28
120.40
120.36
62.52
62.46
31.55
30.16
16.56
16.50
PO(OEt)2
Ph
N
f1 (ppm)

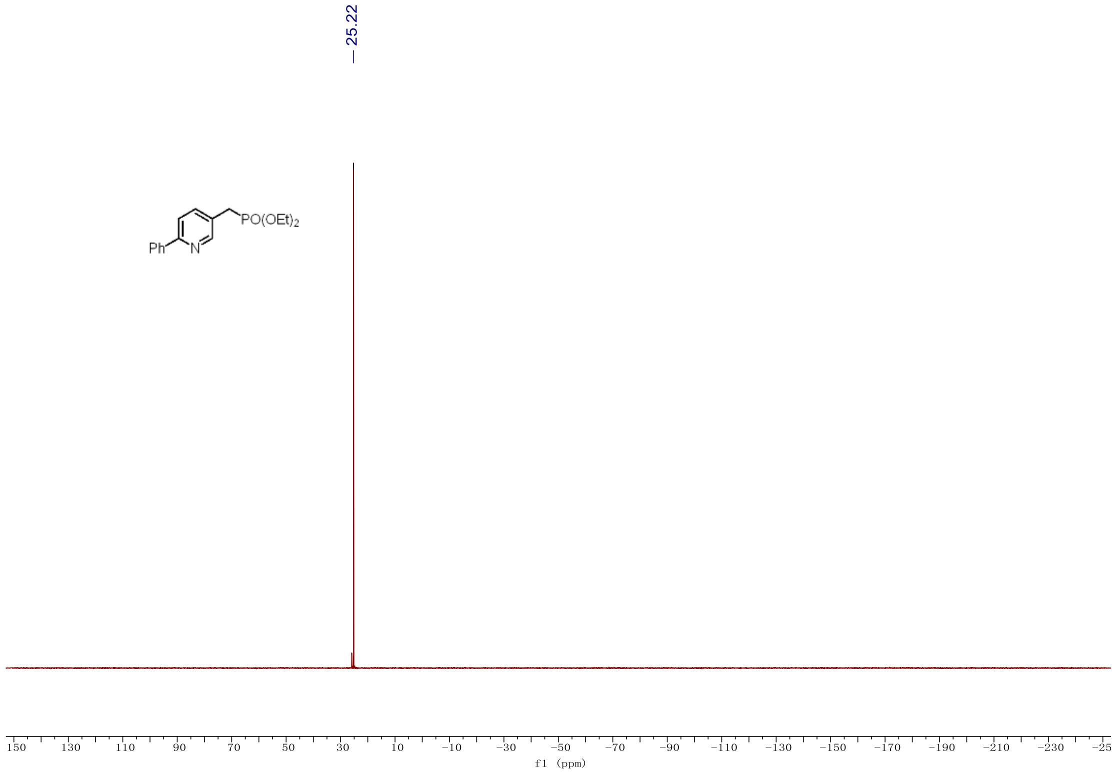
25.22
PO(OEt)2
Ph
N
f1 (ppm)

5b

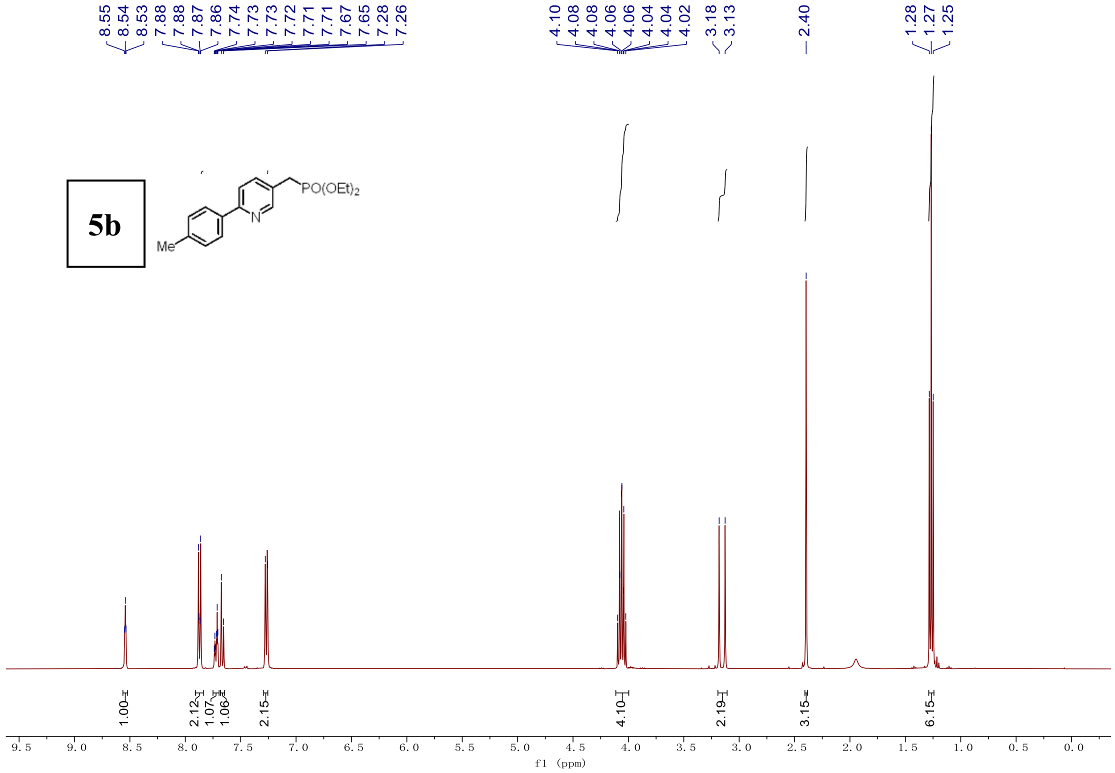
8.55 8.54 8.53 7.88 7.88 7.87 7.86 7.74 7.73 7.73 7.72 7.71 7.71 7.67 7.65 7.28 7.26
4.10 4.08 4.08 4.06 4.06 4.04 4.04 4.02 3.18 3.13
2.40
1.28 1.27 1.25
PO(OEt)2
N
Me
1.00 2.12 1.07 1.06 2.15 4.10 2.19 3.15 6.15
f1 (ppm)

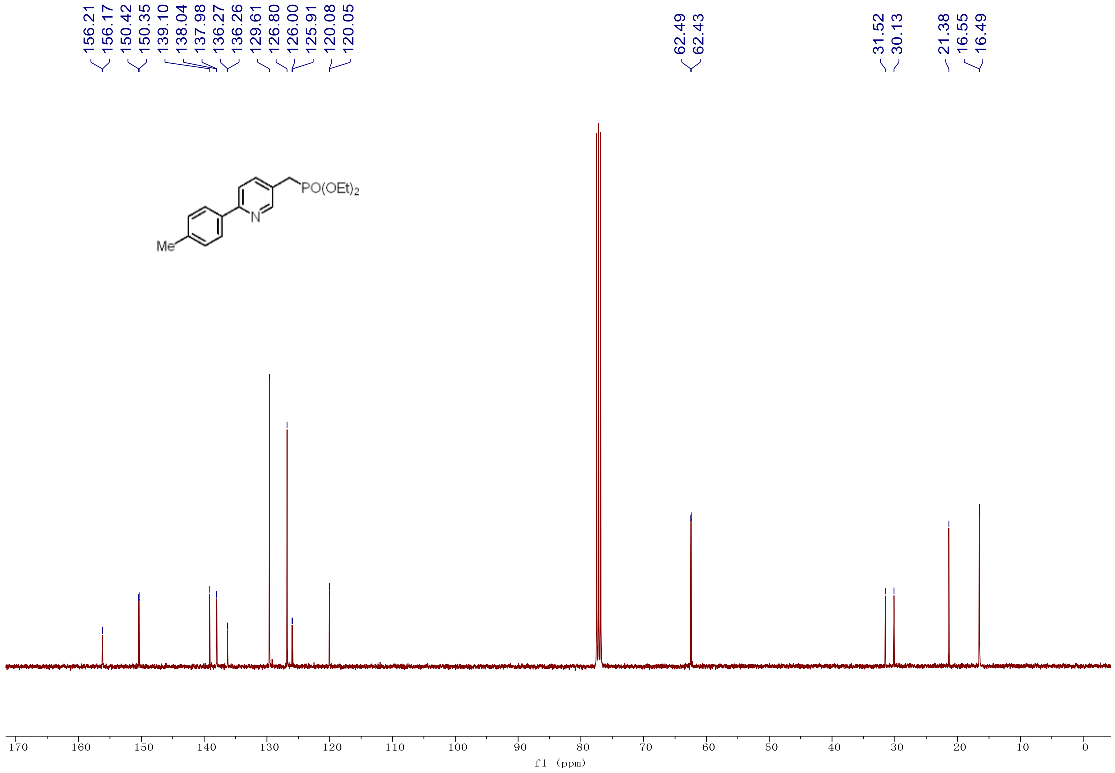
156.21 156.17 150.42 150.35 139.10 138.04 137.98 136.27 136.26 129.61 126.80 126.00 125.91 120.08 120.05
62.49 62.43
31.52 30.13 21.38 16.55 16.49
PO(OEt)2
N
Me
f1 (ppm)

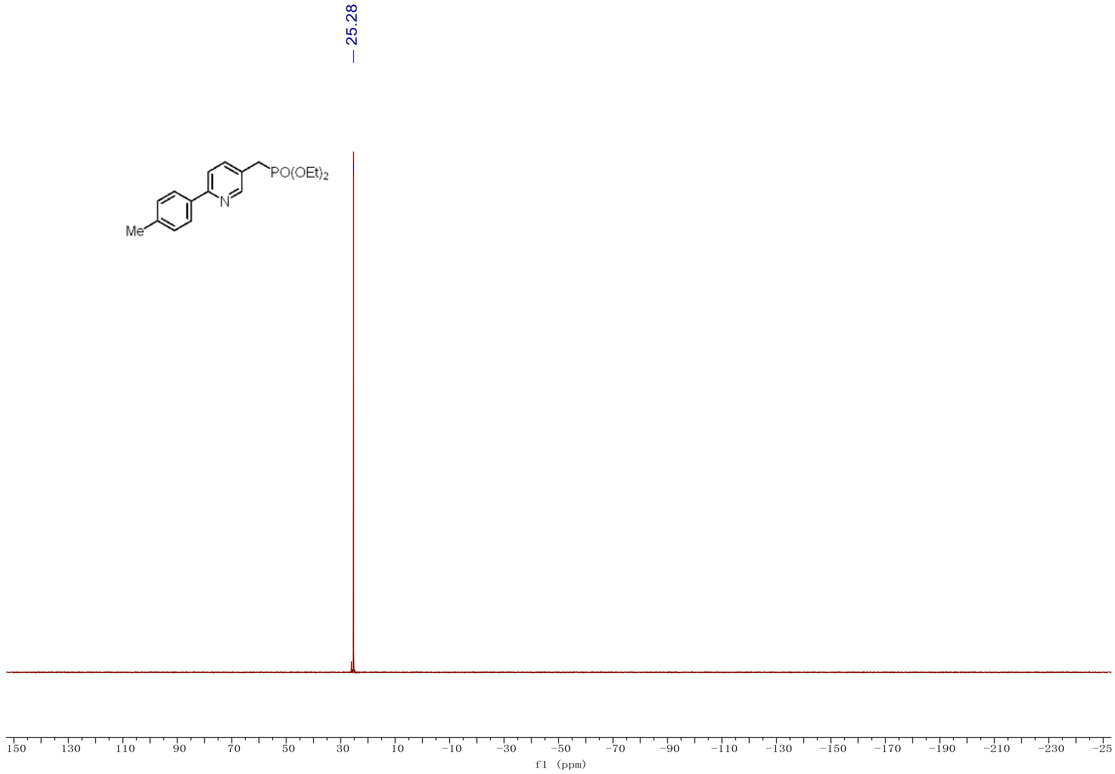
25.28
PO(OEt)2
N
Me
f1 (ppm)

5c

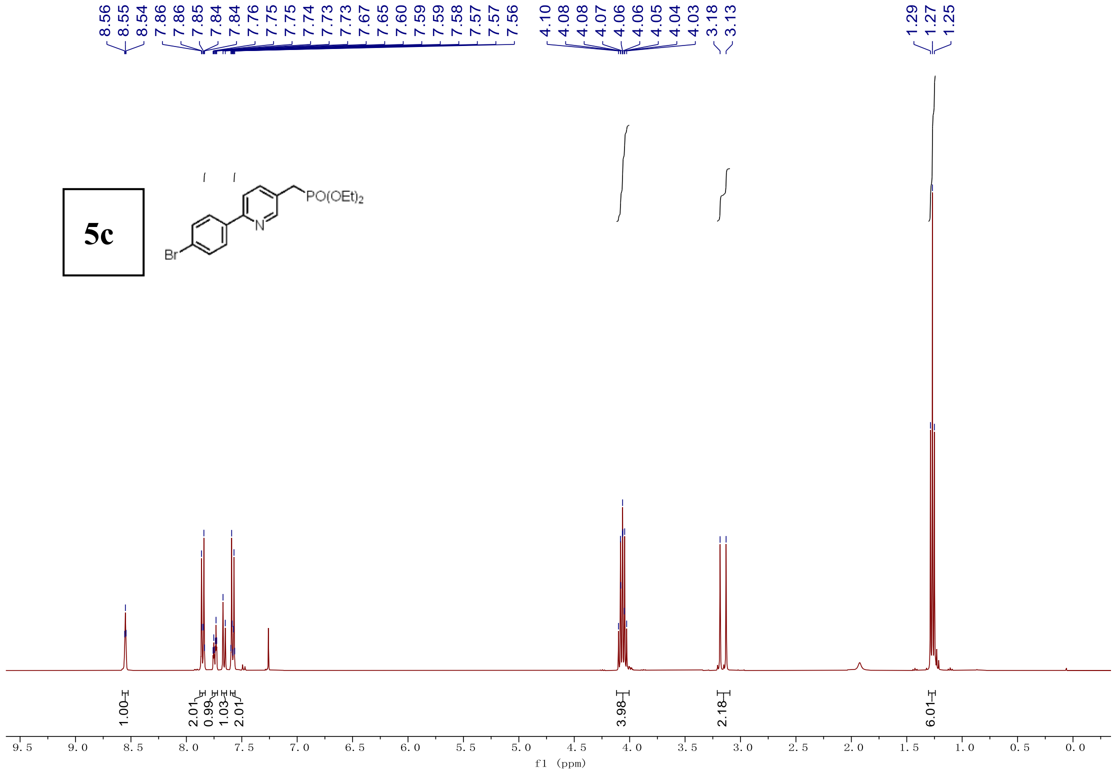
8.56 8.55 8.54 7.86 7.85 7.84 7.84 7.76 7.75 7.75 7.74 7.73 7.73 7.67 7.65 7.60 7.59 7.59 7.58 7.57 7.57 7.56
4.10 4.08 4.07 4.06 4.06 4.05 4.04 4.03 3.18 3.13
1.29 1.27 1.25
PO(OEt)2
N
Br
1.00
2.01
0.99
1.03
2.01
3.98
2.18
6.01
f1 (ppm)

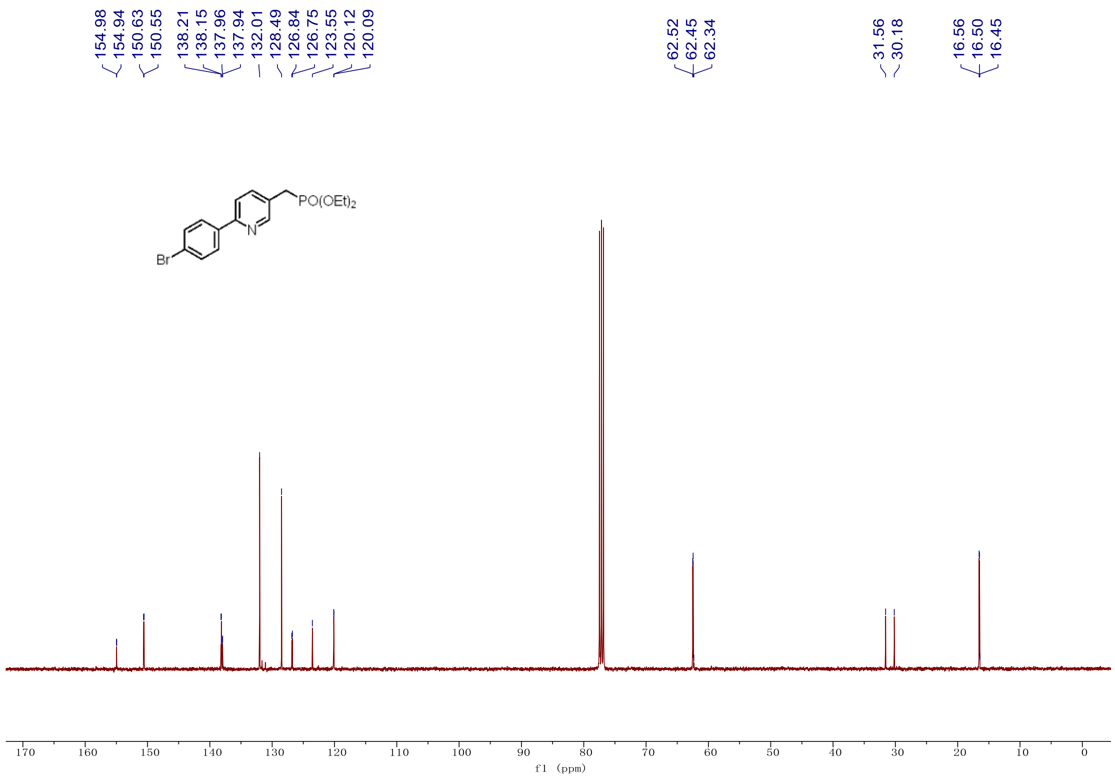
154.98
154.94
150.63
150.55
138.21
138.15
137.96
137.94
132.01
128.49
126.84
126.75
123.55
120.12
120.09
62.52
62.45
62.34
31.56
30.18
16.56
16.50
16.45
PO(OEt)2
N
Br
f1 (ppm)

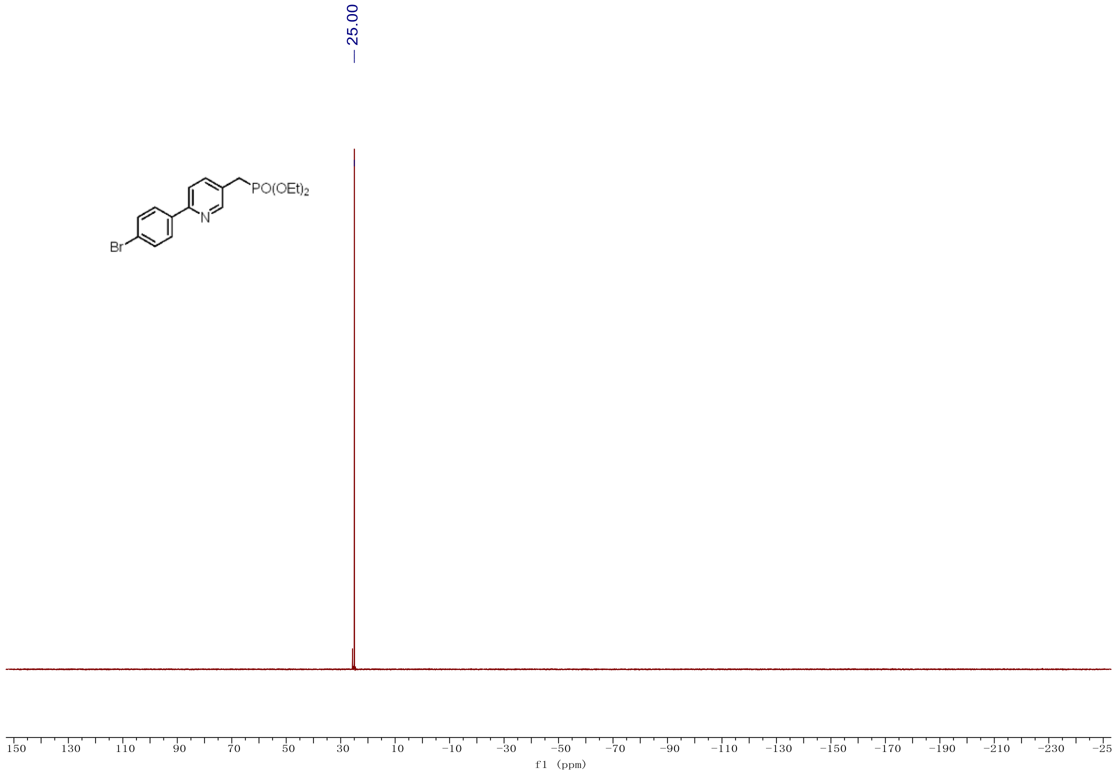
25.00
PO(OEt)2
N
Br
f1 (ppm)

5d

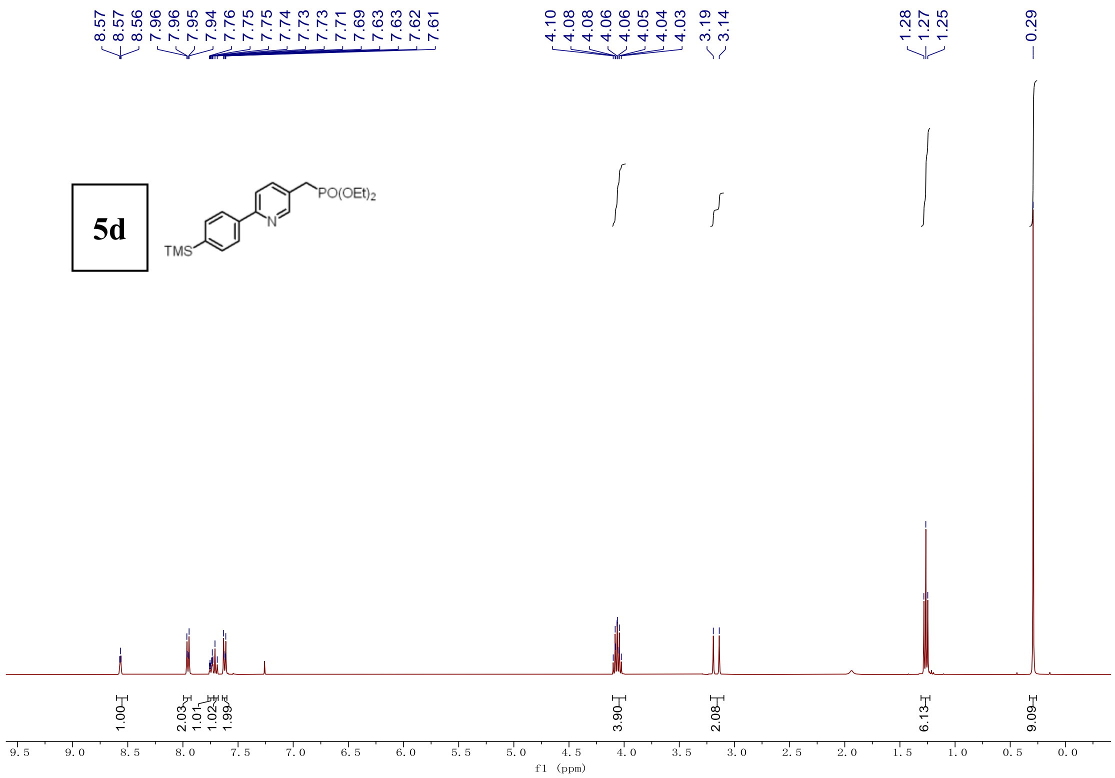
8.57
8.57
8.56
7.96
7.95
7.94
7.76
7.75
7.75
7.74
7.73
7.73
7.71
7.69
7.63
7.63
7.62
7.61
4.10
4.08
4.08
4.06
4.06
4.05
4.04
4.03
3.19
3.14
1.28
1.27
1.25
0.29
PO(OEt)2
TMS
N
1.00
2.03
1.01
1.02
1.99
3.90
2.08
6.13
9.09
f1 (ppm)

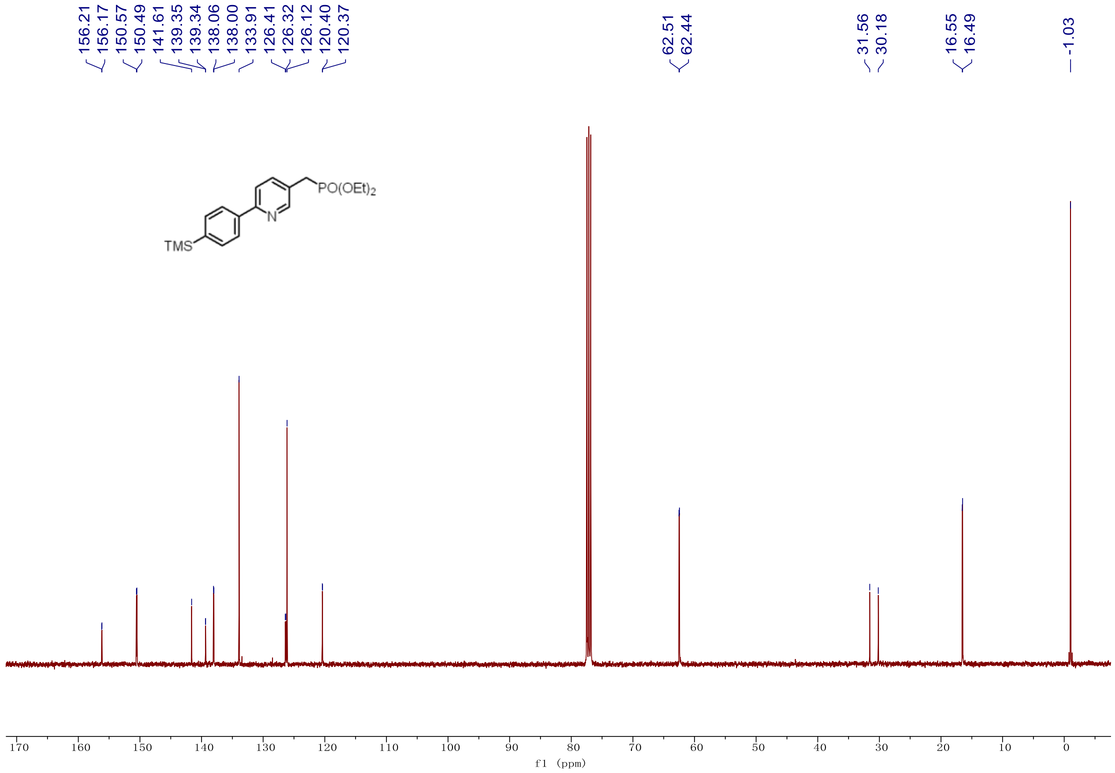
156.21
156.17
150.57
150.49
141.61
139.35
139.34
138.06
138.00
133.91
126.41
126.32
126.12
120.40
120.37
62.51
62.44
31.56
30.18
16.55
16.49
-1.03
PO(OEt)2
TMS
N
f1 (ppm)

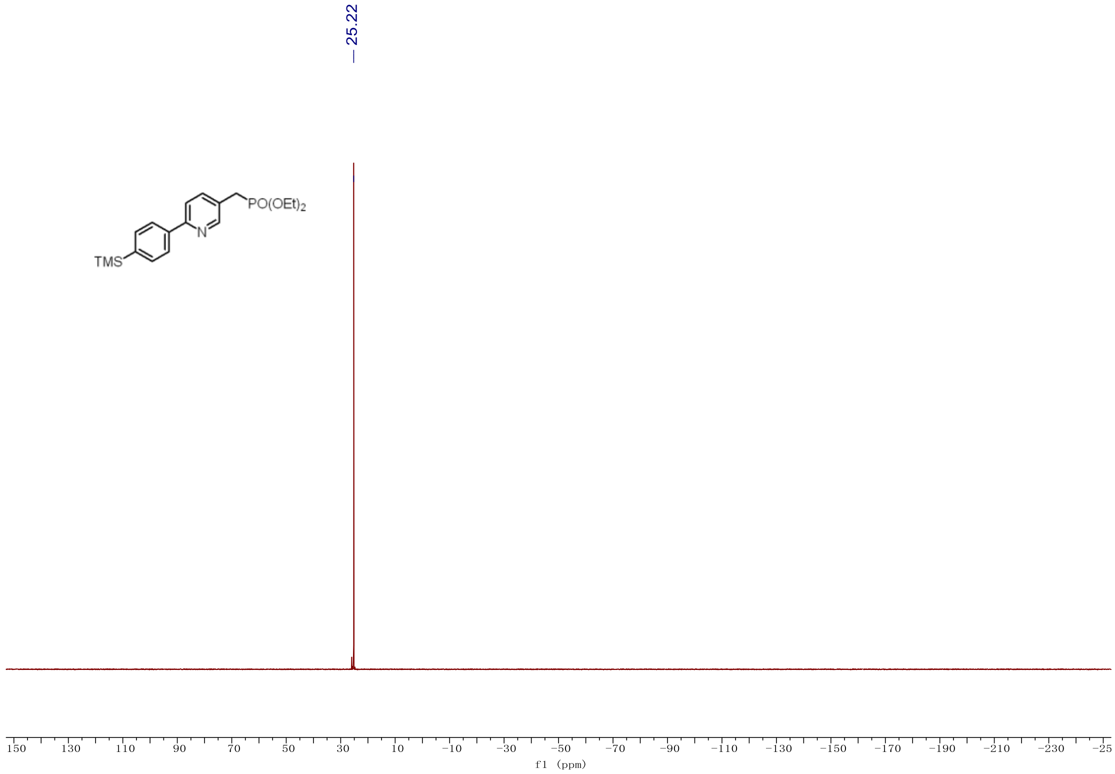

5e

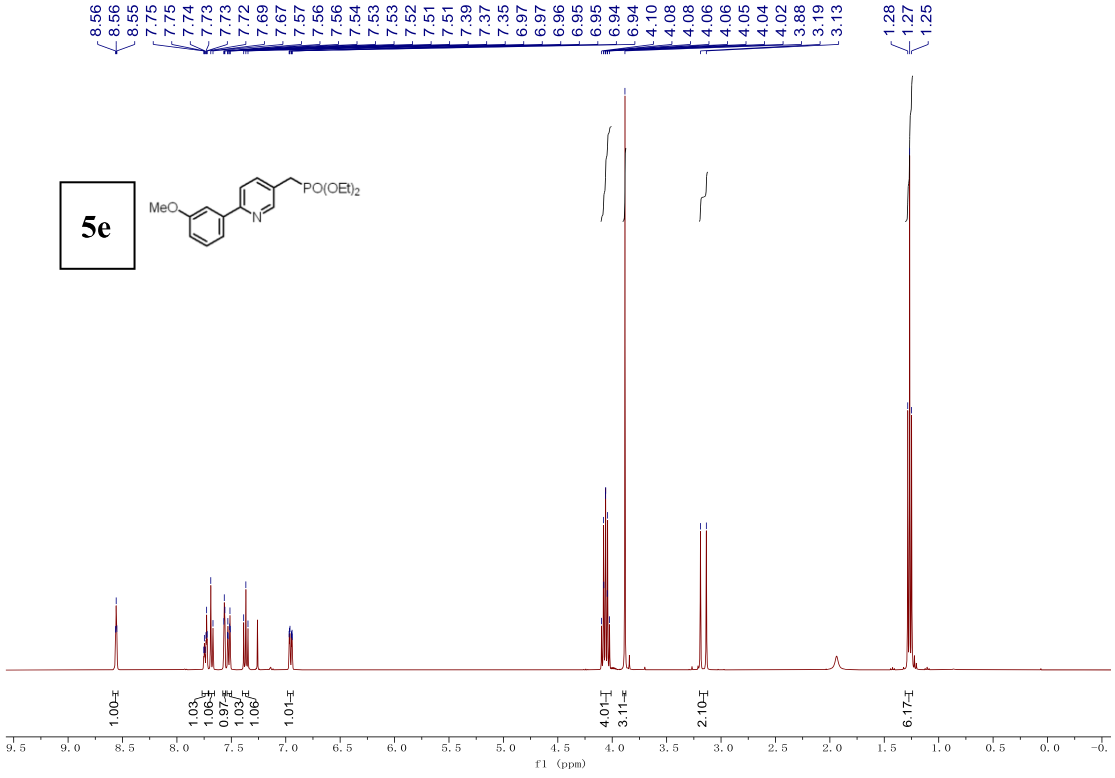

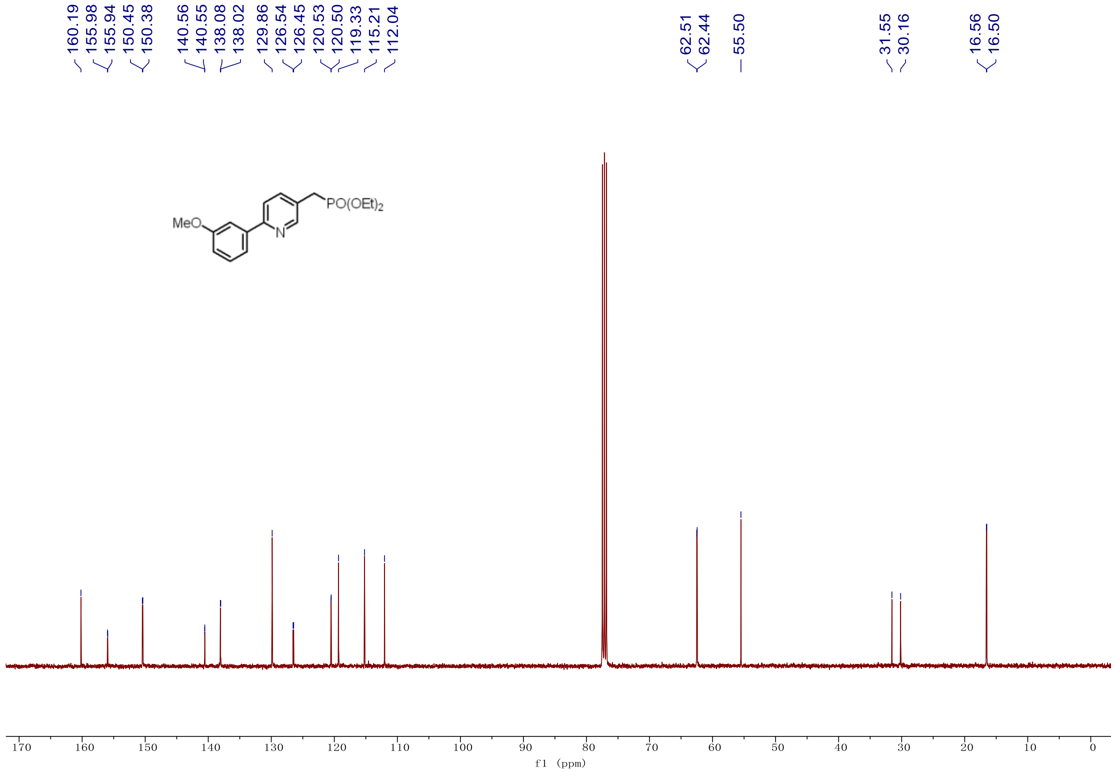
160.19
155.98
155.94
150.45
150.38
140.56
140.55
138.08
138.02
129.86
126.54
126.45
120.53
120.50
119.33
115.21
112.04
62.51
62.44
55.50
31.55
30.16
16.56
16.50
MeO
N
PO(OEt)2
f1 (ppm)

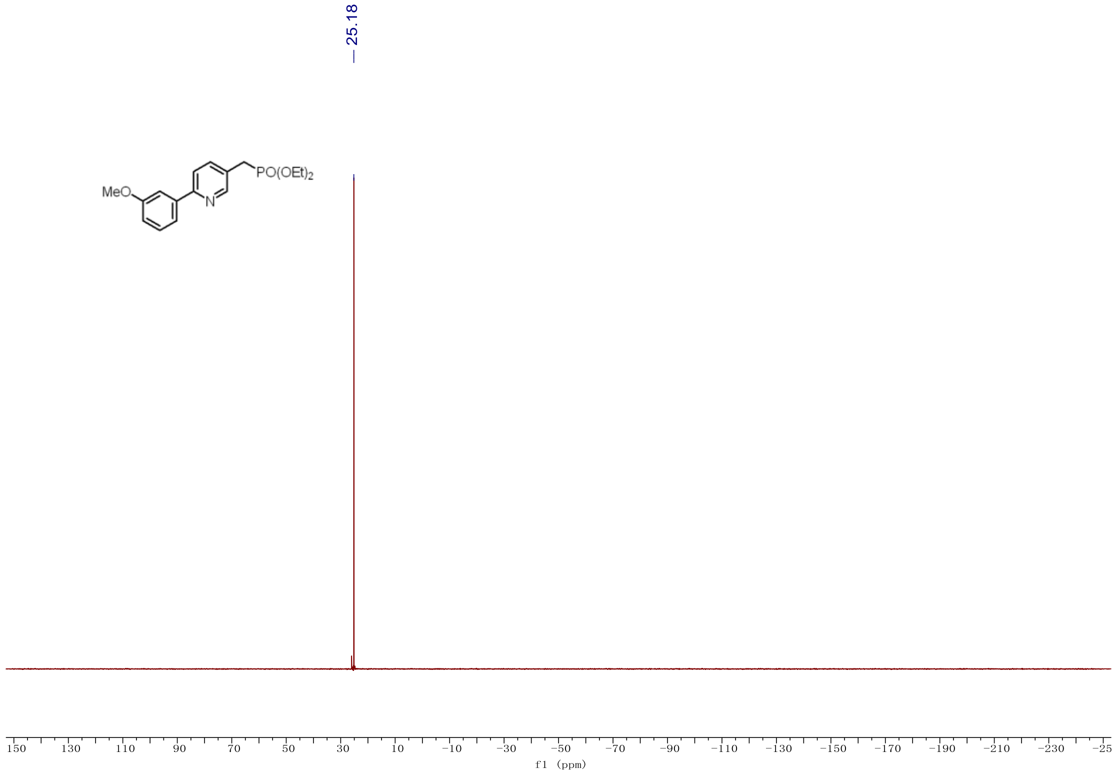
25.18
MeO
N
PO(OEt)2
f1 (ppm)

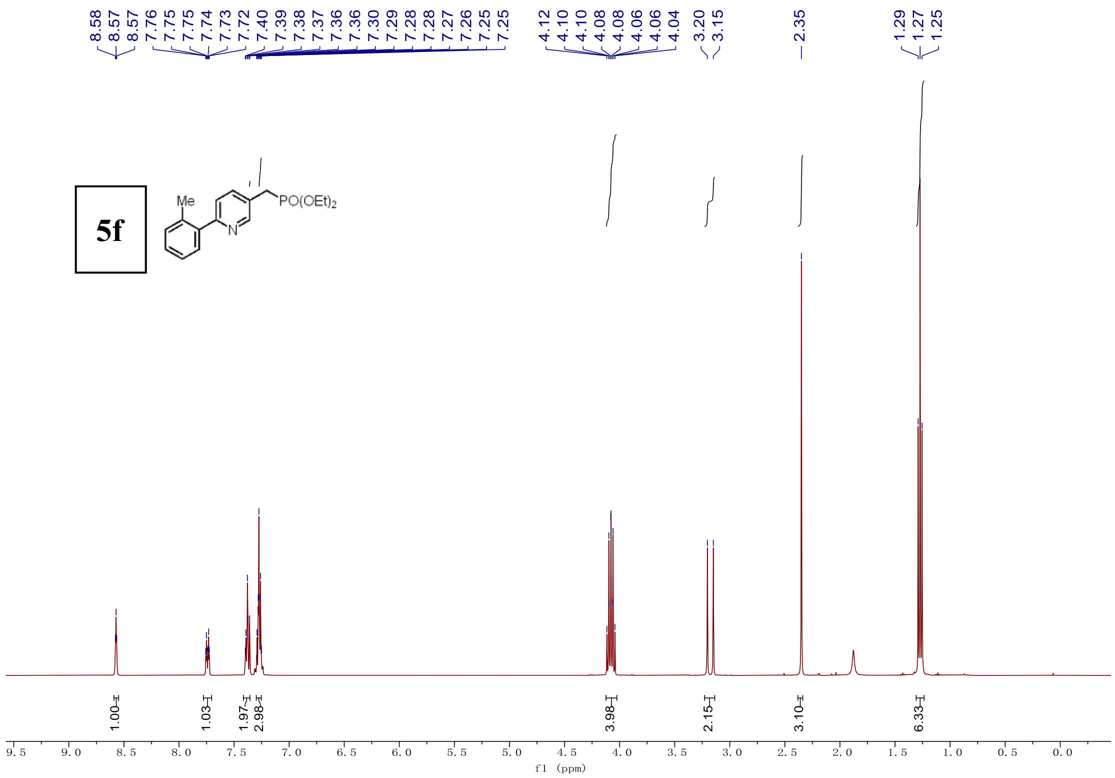
8.58
8.57
8.57
7.76
7.75
7.75
7.74
7.73
7.72
7.40
7.39
7.38
7.37
7.36
7.36
7.30
7.29
7.28
7.28
7.27
7.26
7.25
7.25
4.12
4.10
4.10
4.08
4.08
4.06
4.06
4.04
3.20
3.15
2.35
1.29
1.27
1.25
5f
Me
N
PO(OEt)2
1.00
1.03
1.97
2.98
3.98
2.15
3.10
6.33
f1 (ppm)

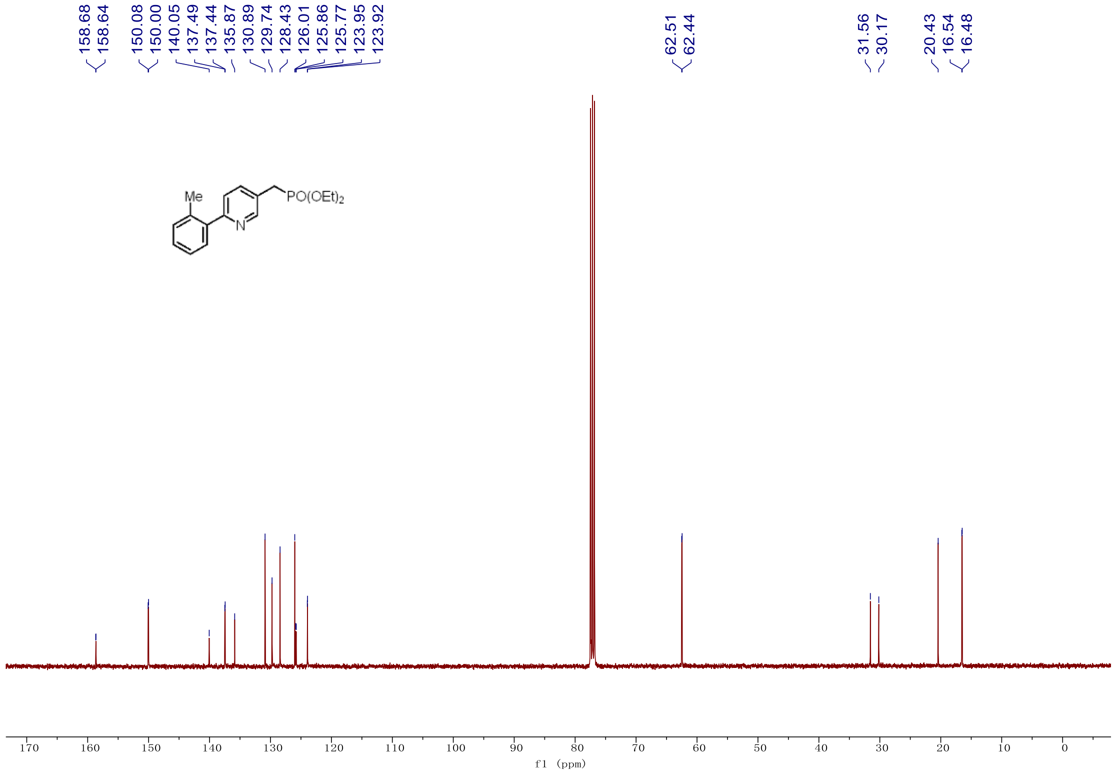
158.68
158.64
150.08
150.00
140.05
137.49
137.44
135.87
130.89
129.74
128.43
126.01
125.86
125.77
123.95
123.92
62.51
62.44
31.56
30.17
20.43
16.54
16.48
Me
N
PO(OEt)2
f1 (ppm)

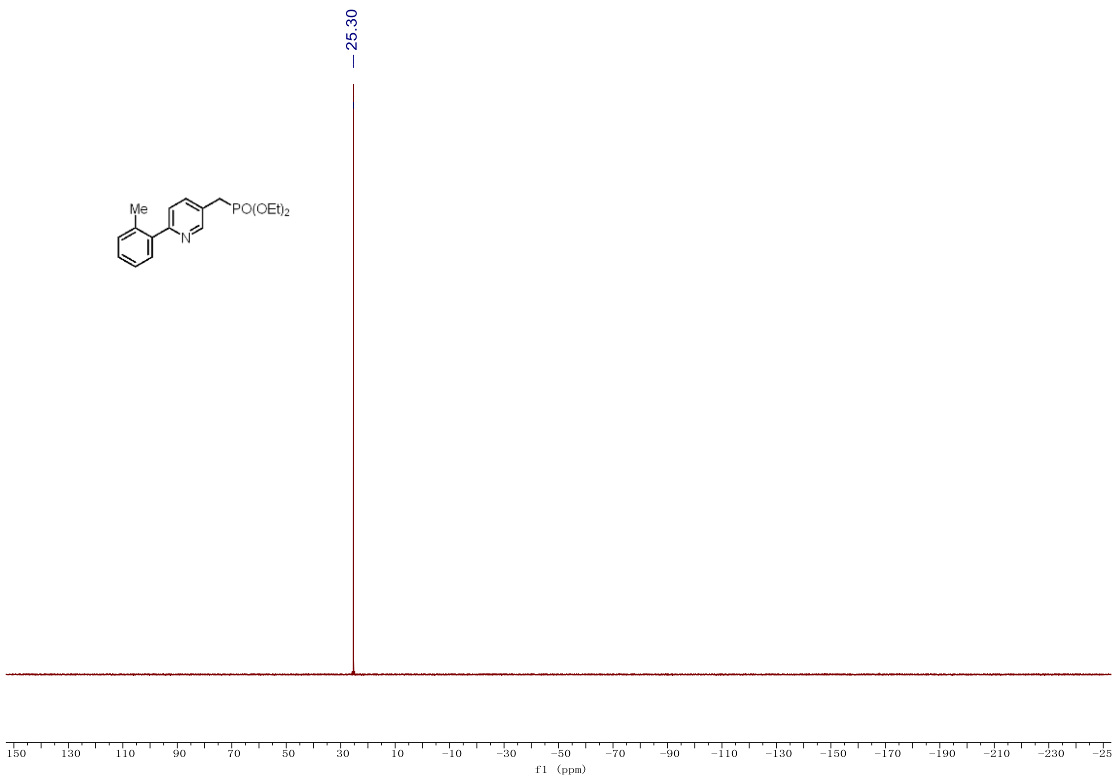

5g

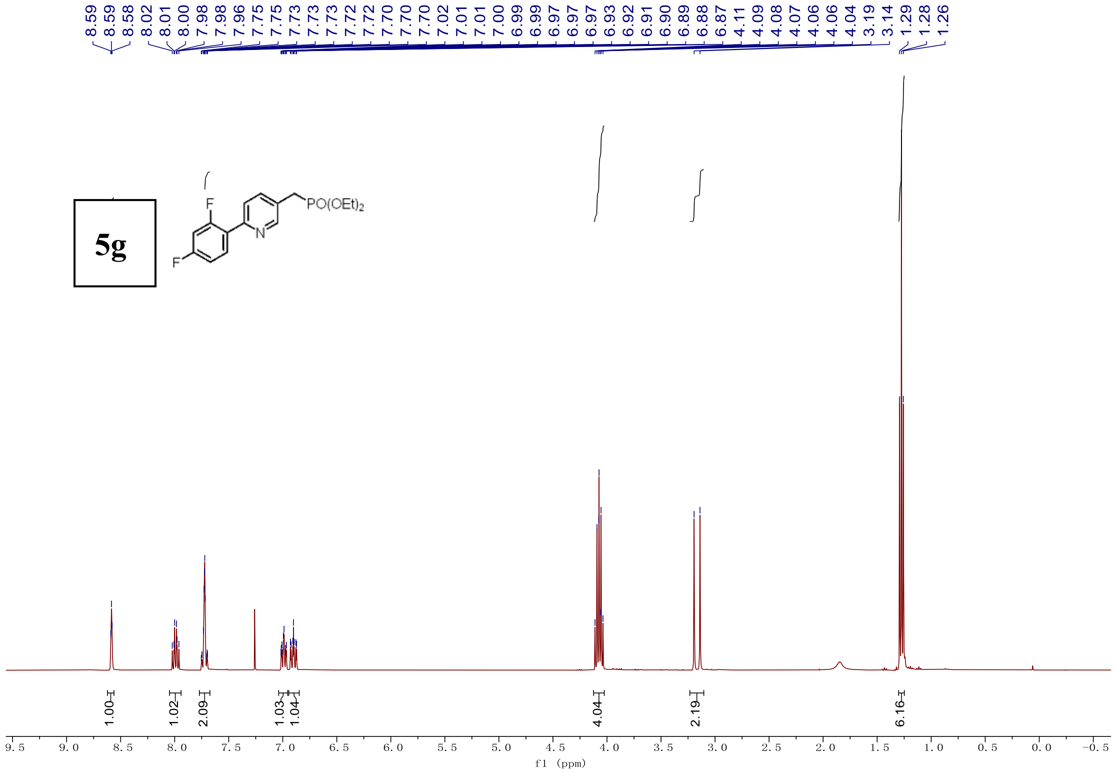

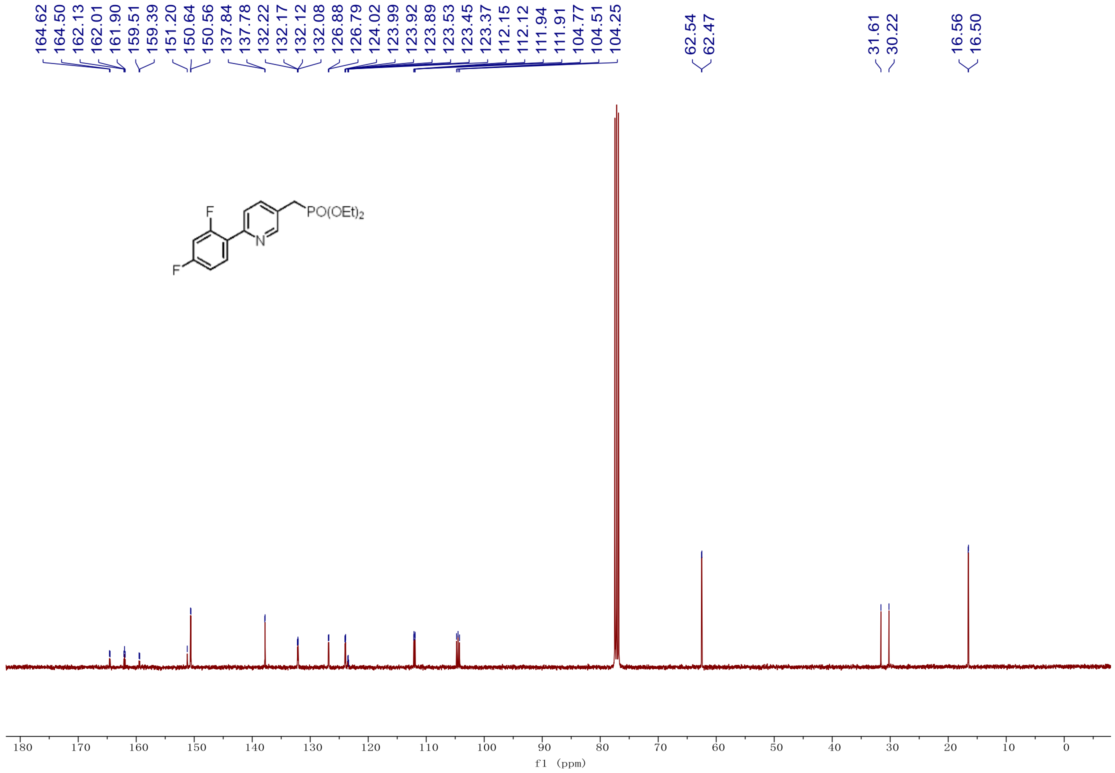
164.62
164.50
162.13
162.01
161.90
159.51
159.39
151.20
150.64
150.56
137.84
137.78
132.22
132.17
132.12
132.08
126.88
126.79
124.02
123.99
123.92
123.89
123.53
123.45
123.37
112.15
112.12
111.94
111.91
104.77
104.51
104.25
62.54
62.47
31.61
30.22
16.56
16.50
F
F
N
PO(OEt)2
f1 (ppm)

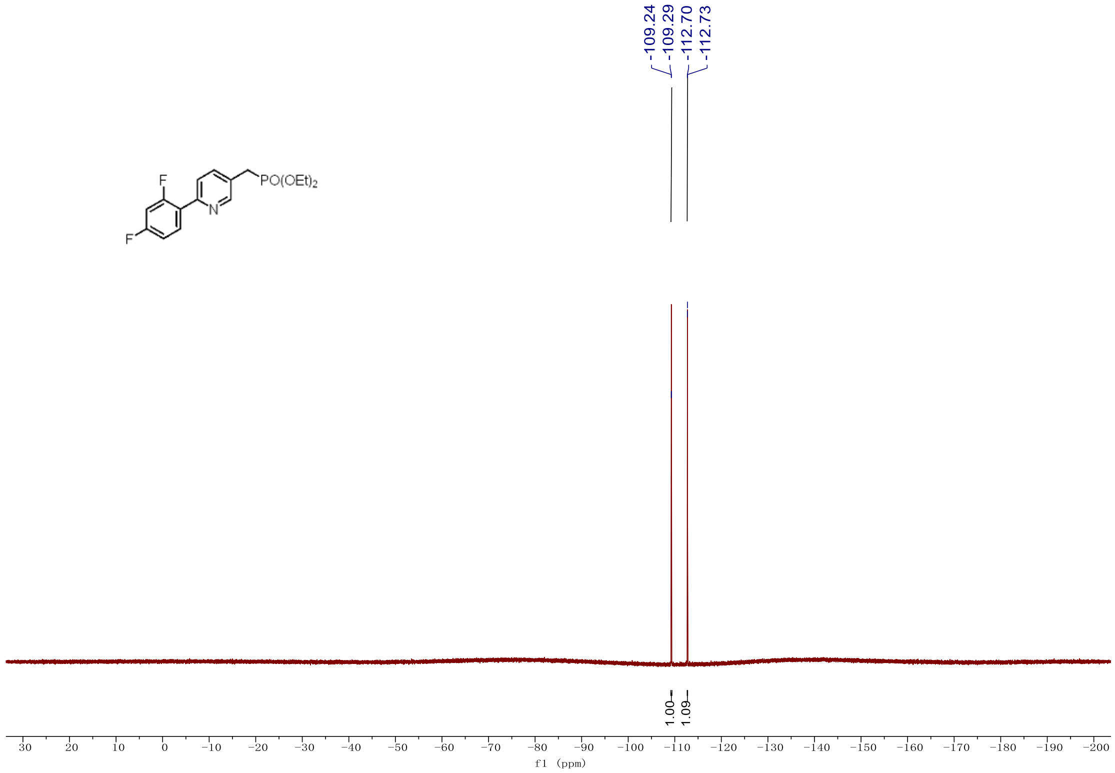
-109.24
-109.29
-112.70
-112.73
F
F
N
PO(OEt)2
1.00
1.09
f1 (ppm)

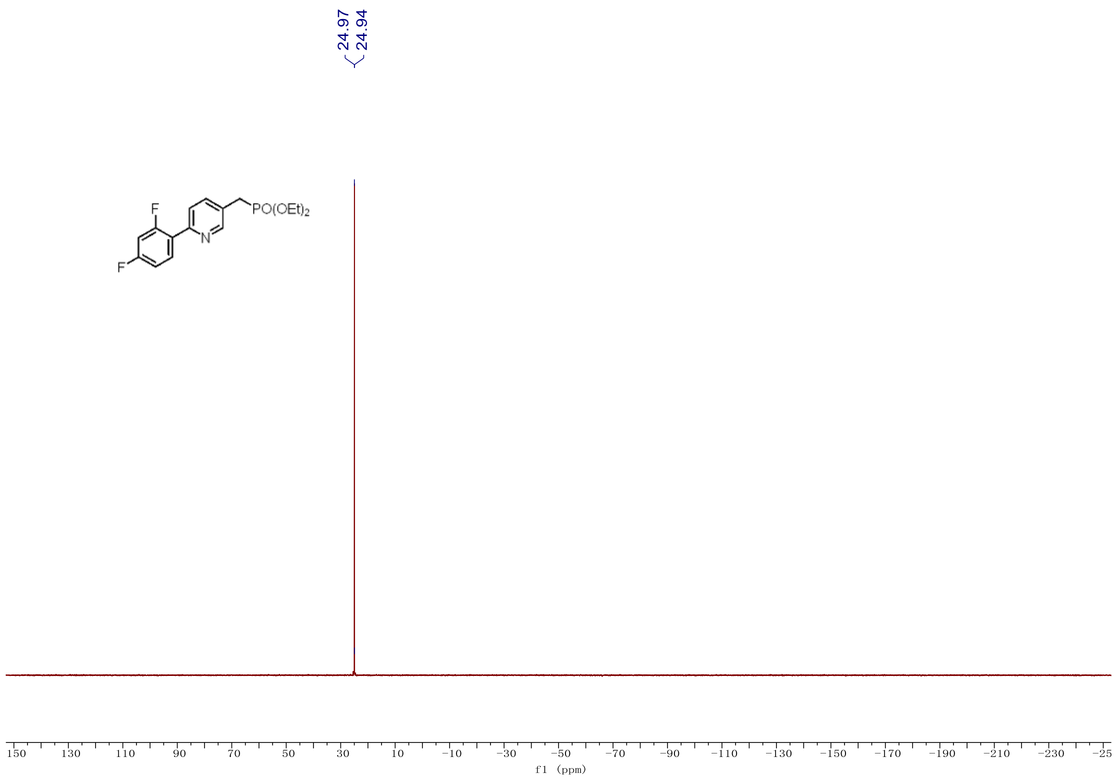
24.97
24.94
PO(OEt)2
F
F
N
150 130 110 90 70 50 30 10 −10 −30 −50 −70 −90 −110 −130 −150 −170 −190 −210 −230 −25
f1 (ppm)

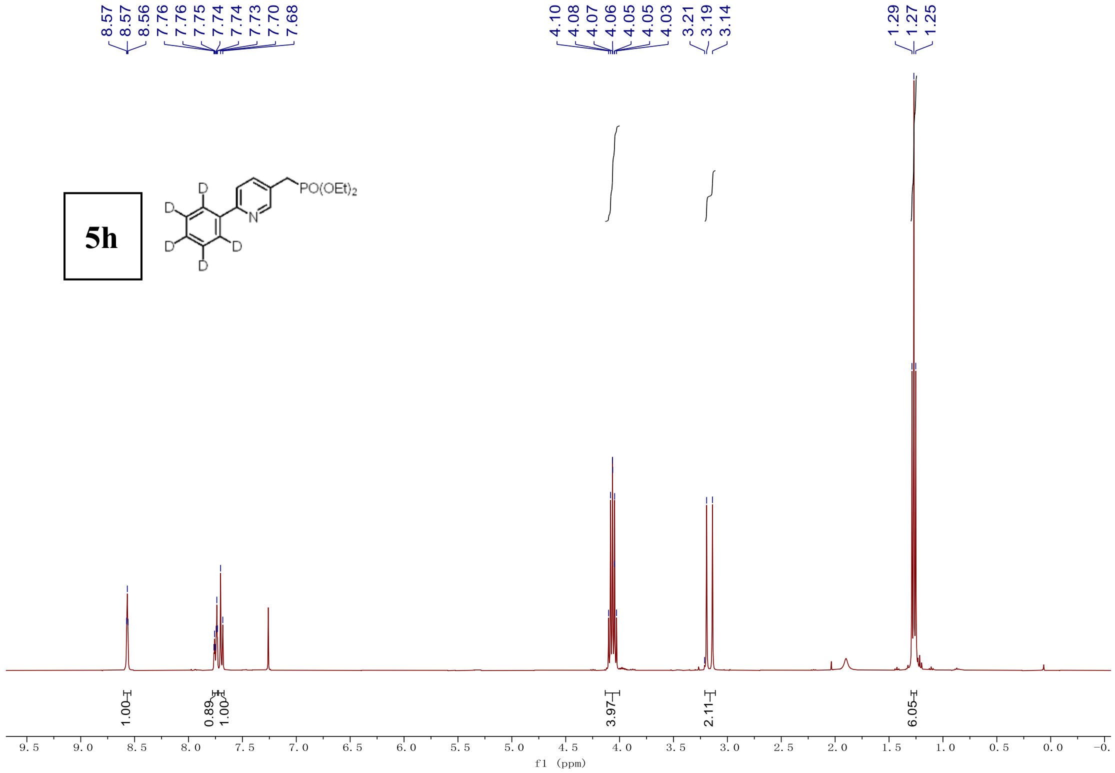
8.57
8.57
8.56
7.76
7.76
7.75
7.74
7.74
7.73
7.70
7.68
4.10
4.08
4.07
4.06
4.05
4.05
4.03
3.21
3.19
3.14
1.29
1.27
1.25
5h
D
D
D
D
D
N
PO(OEt)2
1.00
0.89
1.00
3.97
2.11
6.05
9.5 9.0 8.5 8.0 7.5 7.0 6.5 6.0 5.5 5.0 4.5 4.0 3.5 3.0 2.5 2.0 1.5 1.0 0.5 0.0 −0.
f1 (ppm)

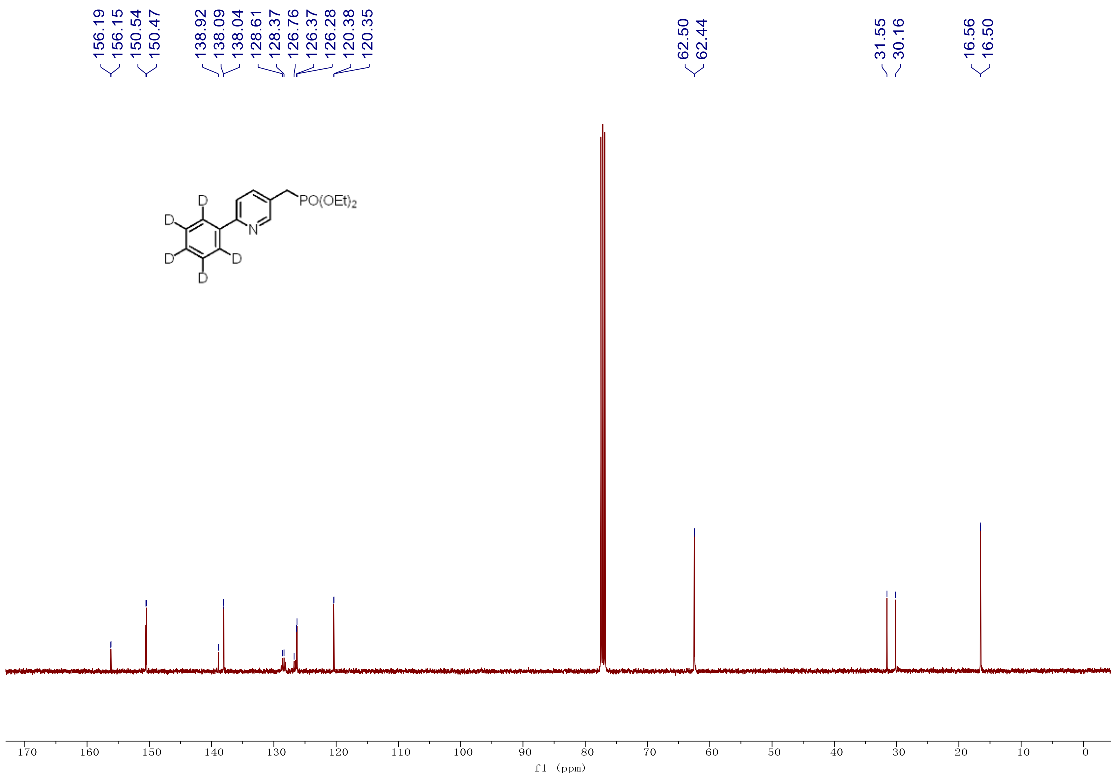
156.19
156.15
150.54
150.47
138.92
138.09
138.04
128.61
128.37
126.76
126.37
126.28
120.38
120.35
62.50
62.44
31.55
30.16
16.56
16.50
PO(OEt)2
D
N
f1 (ppm)

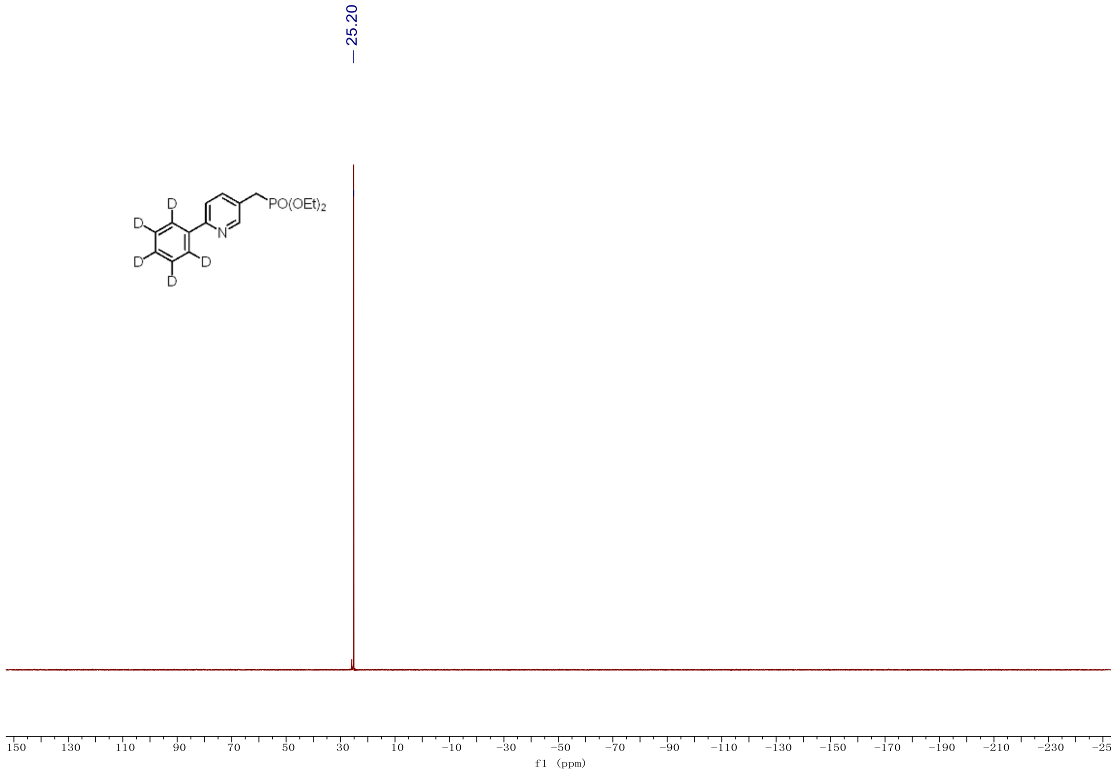
25.20
PO(OEt)2
D
N
f1 (ppm)

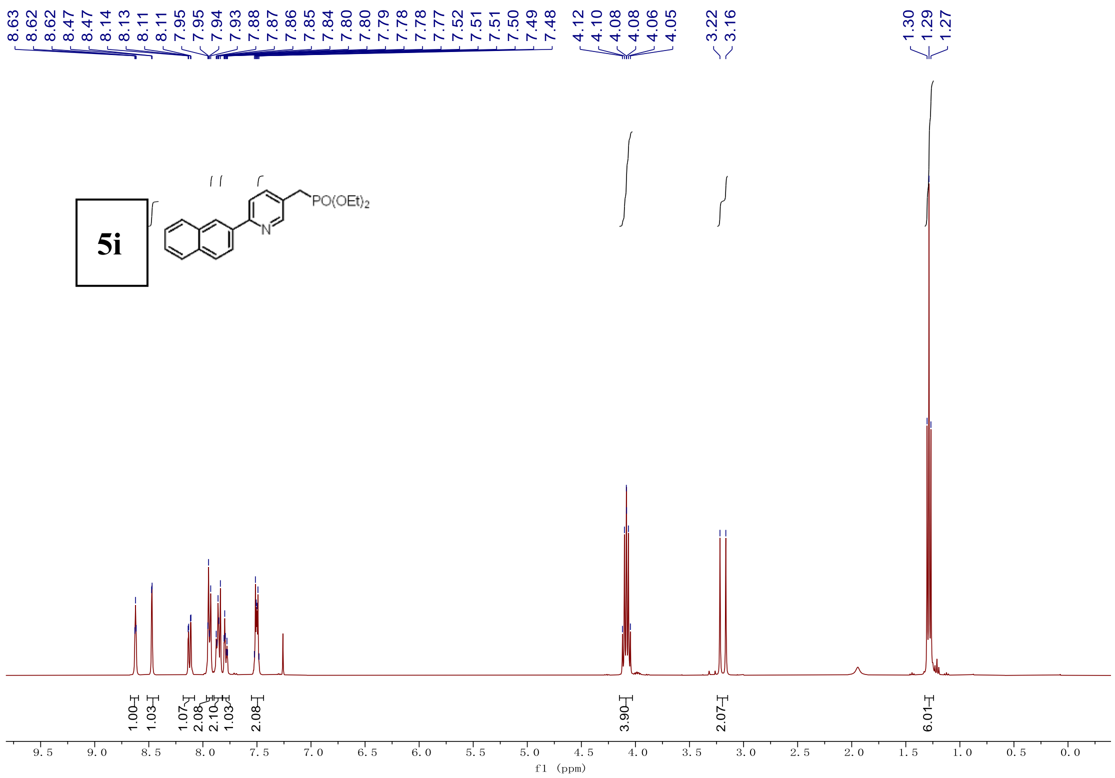
5i
PO(OEt)2
N
8.63 8.62 8.62 8.47 8.47 8.14 8.13 8.11 8.11 7.95 7.95 7.94 7.93 7.88 7.87 7.86 7.85 7.84 7.80 7.79 7.78 7.78 7.77 7.52 7.51 7.51 7.50 7.49 7.48
4.12 4.10 4.08 4.08 4.06 4.05
3.22 3.16
1.30 1.29 1.27
1.00 1.03 1.07 2.08 2.10 1.03 2.08 3.90 2.07 6.01
f1 (ppm)

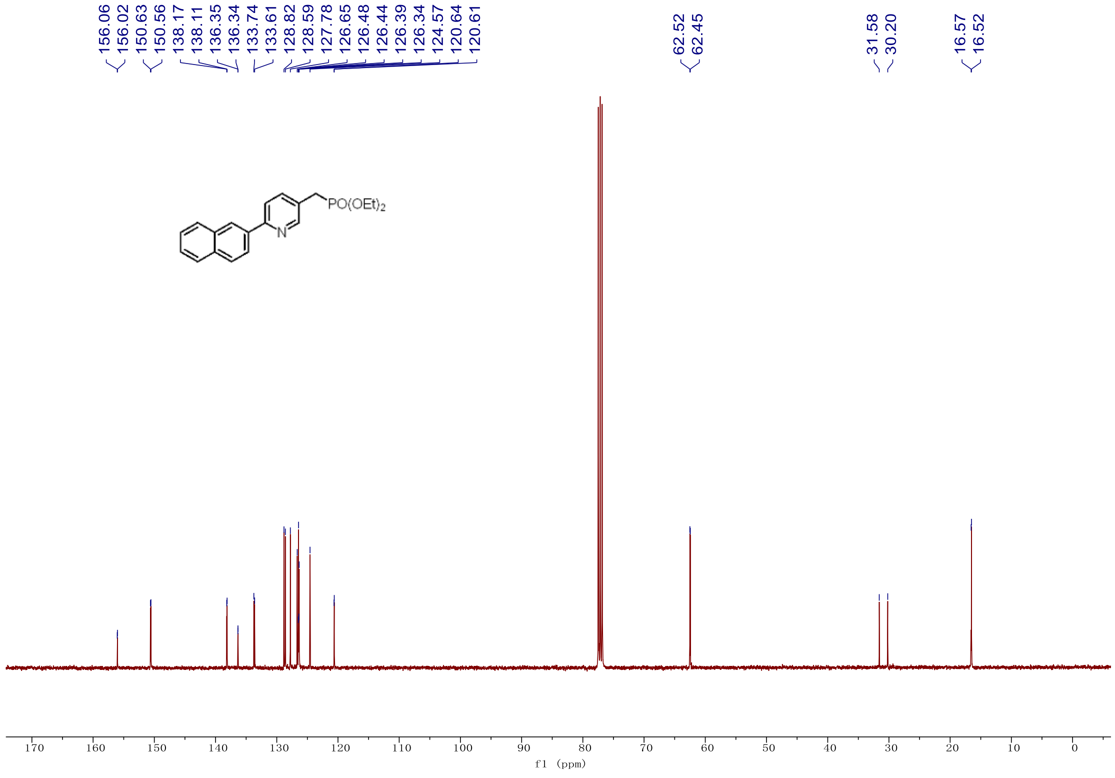
156.06 156.02 150.63 150.56 138.17 138.11 136.35 136.34 133.74 133.61 128.82 128.59 127.78 126.65 126.48 126.44 126.39 126.34 124.57 120.64 120.61
62.52 62.45
31.58 30.20
16.57 16.52
PO(OEt)2
N
f1 (ppm)

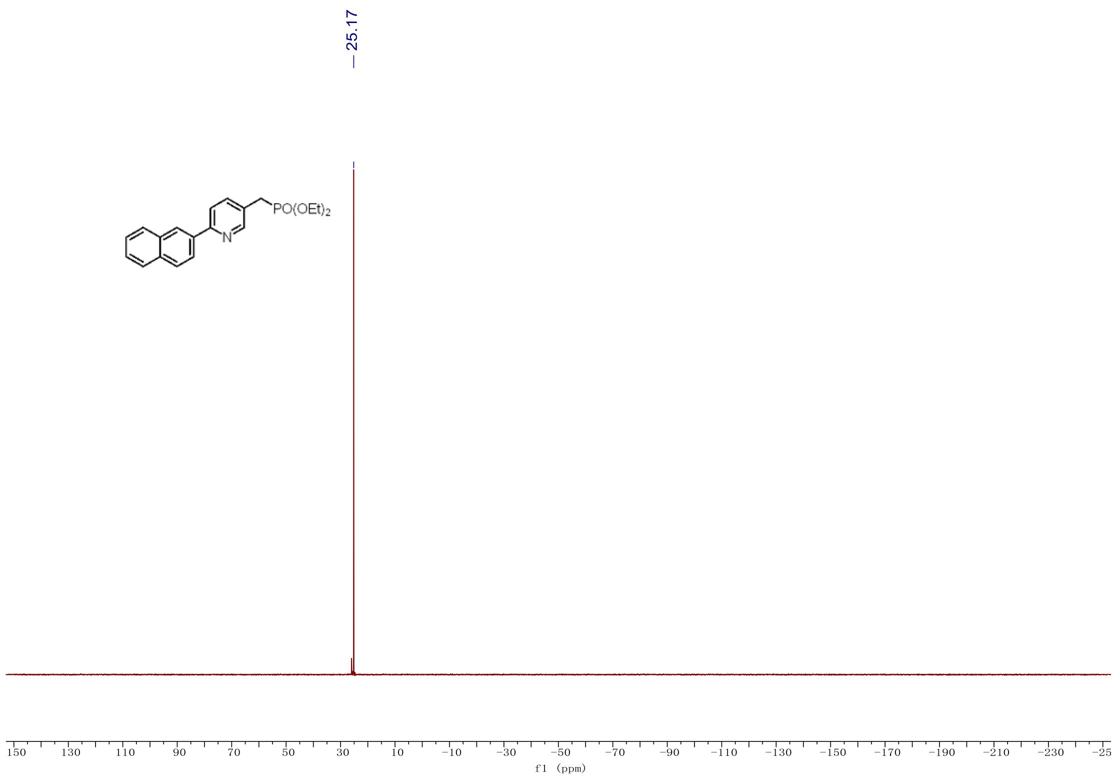

25.17
PO(OEt)2
N
f1 (ppm)

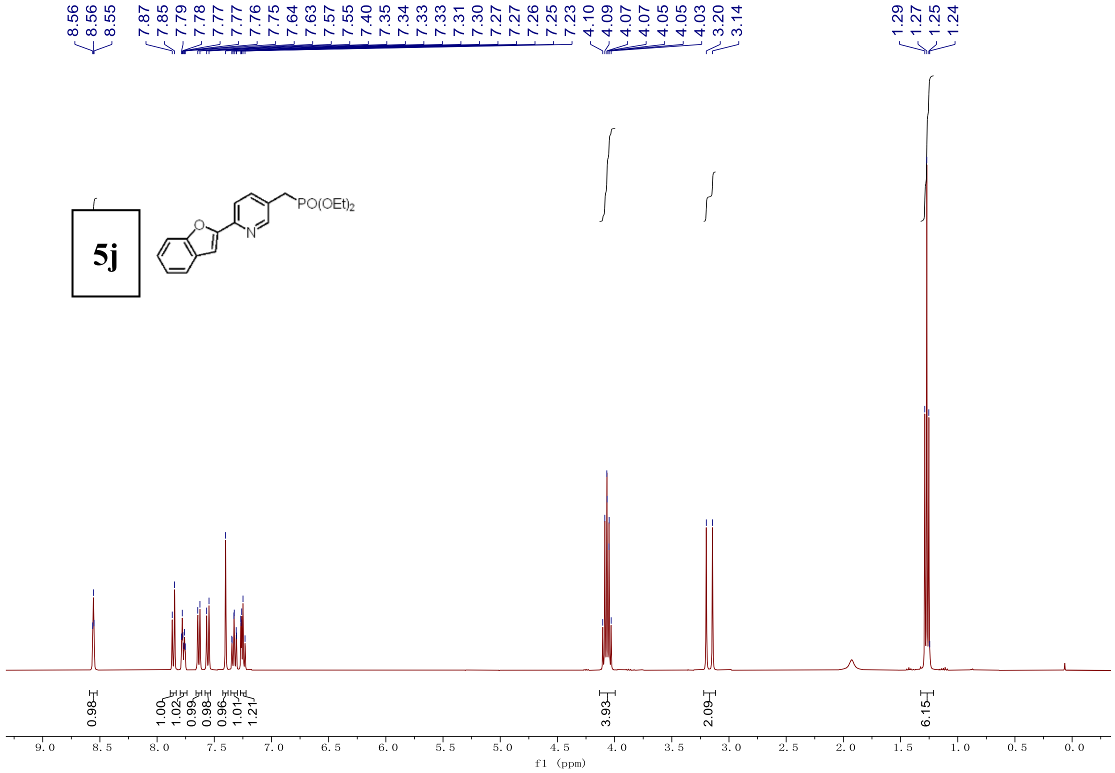

8.56
8.56
8.55
7.87
7.85
7.79
7.78
7.77
7.77
7.76
7.75
7.64
7.63
7.57
7.55
7.40
7.35
7.34
7.33
7.33
7.31
7.30
7.27
7.27
7.26
7.25
7.23
4.10
4.09
4.07
4.07
4.05
4.05
4.03
3.20
3.14
1.29
1.27
1.25
1.24
PO(OEt)2
O
N
0.98
1.00
1.02
0.99
0.98
0.96
1.01
1.21
3.93
2.09
6.15
f1 (ppm)

5j

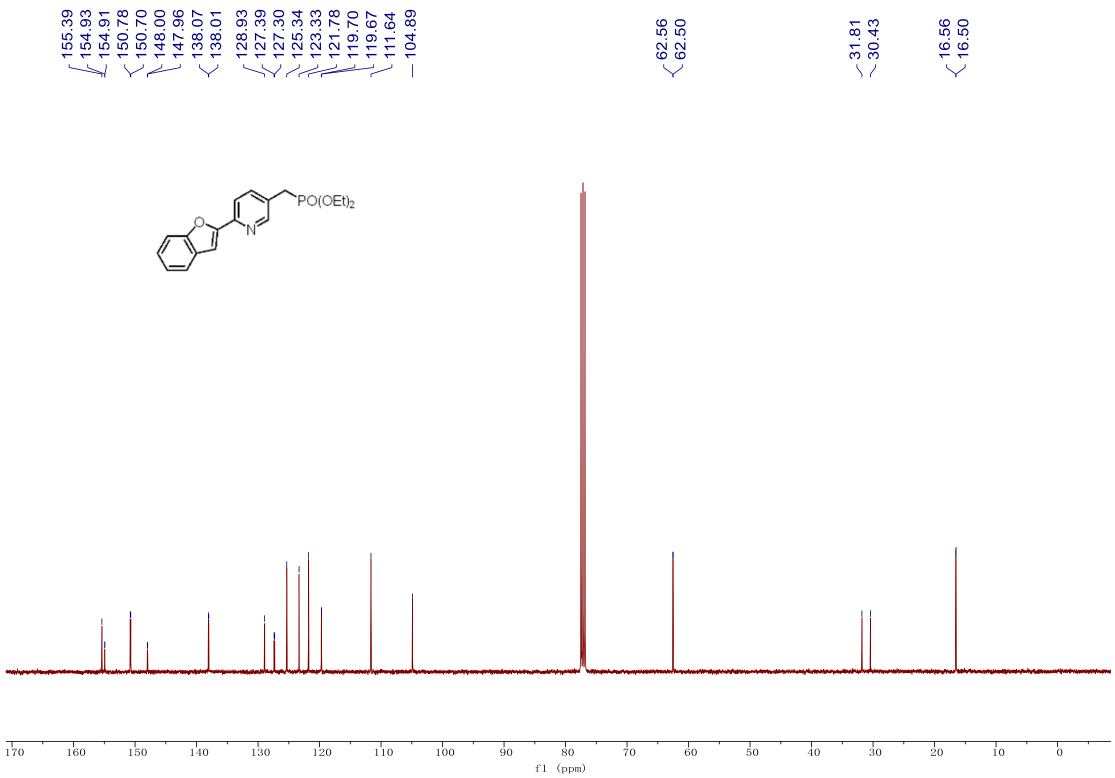
155.39
154.93
154.91
150.78
150.70
148.00
147.96
138.07
138.01
128.93
127.39
127.30
125.34
123.33
121.78
119.70
119.67
111.64
104.89
62.56
62.50
31.81
30.43
16.56
16.50
PO(OEt)2
O
N
f1 (ppm)

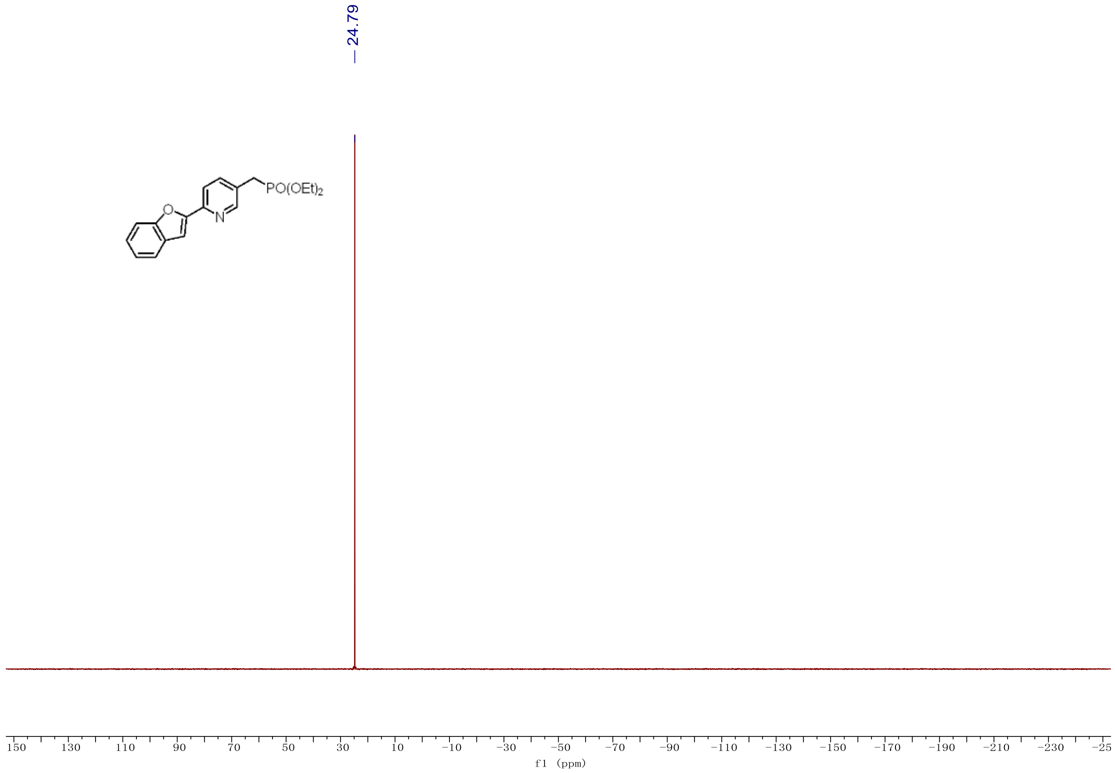
24.79
PO(OEt)2
O
N
f1 (ppm)

5k

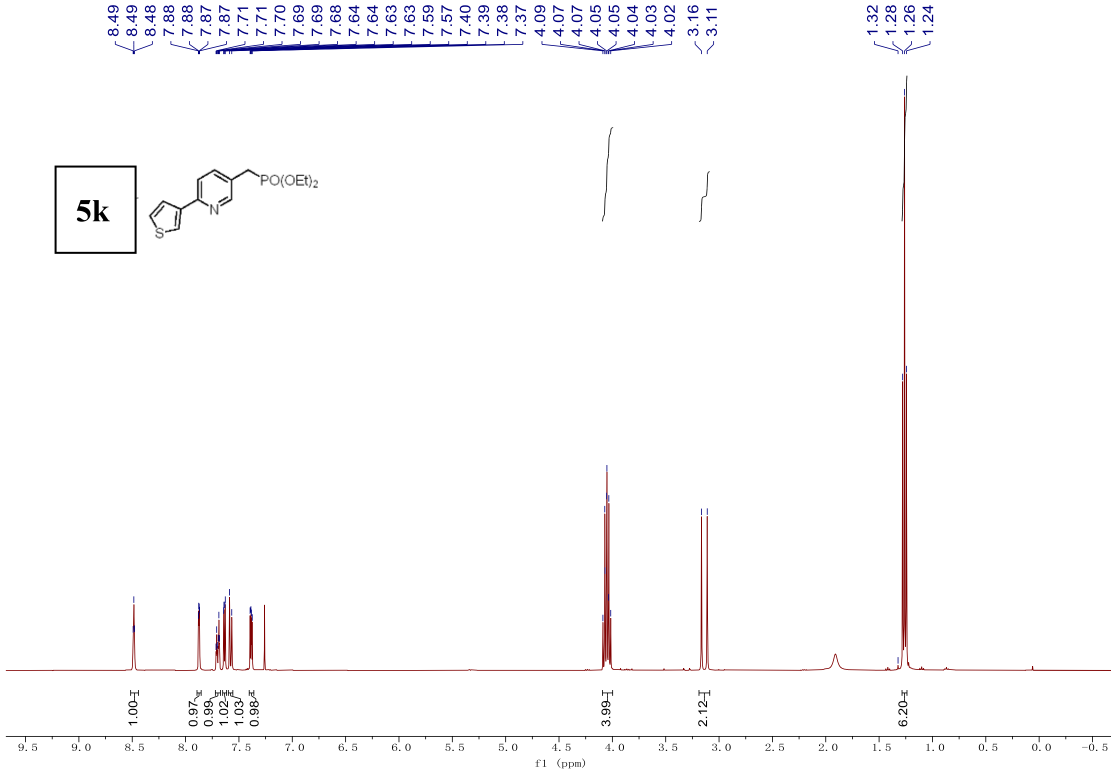
8.49
8.49
8.48
7.88
7.87
7.87
7.71
7.71
7.70
7.69
7.69
7.68
7.64
7.64
7.63
7.63
7.59
7.57
7.40
7.39
7.38
7.37
4.09
4.07
4.07
4.05
4.05
4.04
4.04
4.03
4.02
3.16
3.11
1.32
1.28
1.26
1.24
PO(OEt)2
N
S
1.00
0.97
0.99
1.02
1.03
0.98
3.99
2.12
6.20
f1 (ppm)

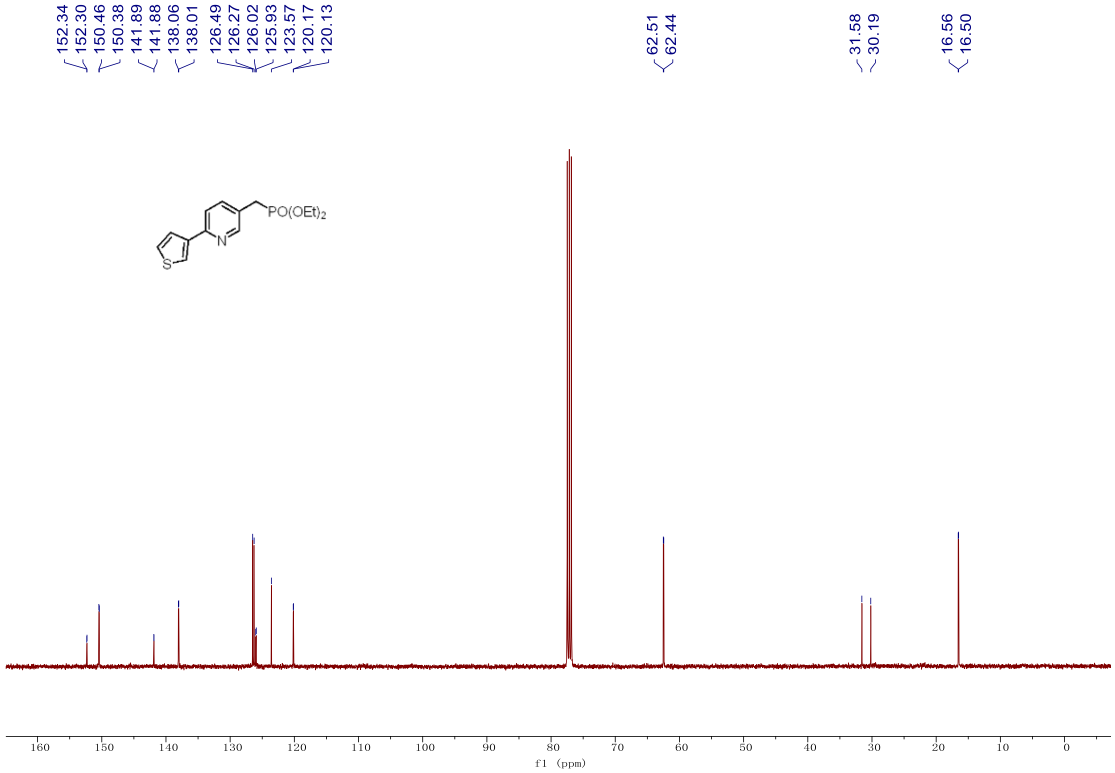
152.34
152.30
150.46
150.38
141.89
141.88
138.06
138.01
126.49
126.27
126.02
125.93
123.57
120.17
120.13
62.51
62.44
31.58
30.19
16.56
16.50
PO(OEt)2
N
S
f1 (ppm)

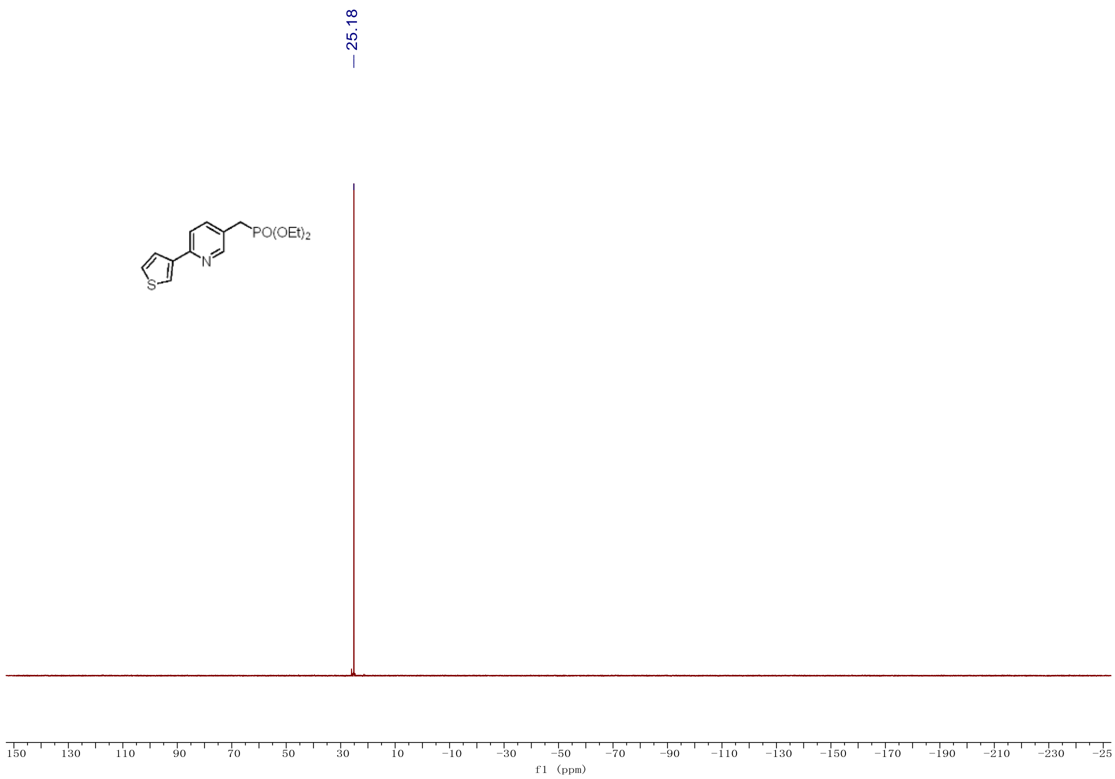
25.18
PO(OEt)2
S
N
f1 (ppm)

5l

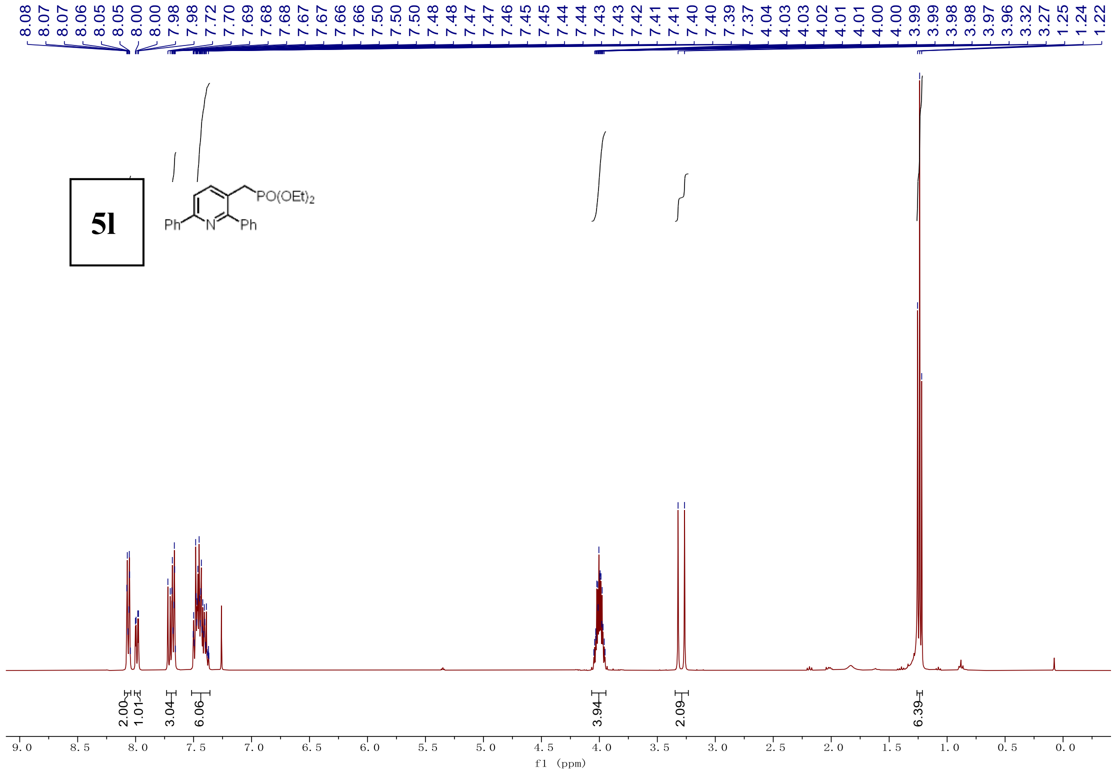
8.08 8.07 8.07 8.06 8.05 8.05 8.00 8.00 7.98 7.98 7.72 7.70 7.69 7.68 7.67 7.67 7.66 7.50 7.50 7.48 7.48 7.47 7.47 7.46 7.45 7.45 7.44 7.44 7.43 7.43 7.42 7.41 7.41 7.40 7.40 7.39 7.37 4.04 4.03 4.03 4.02 4.01 4.01 4.00 4.00 3.99 3.99 3.98 3.98 3.97 3.96 3.32 3.27 1.25 1.24 1.22
PO(OEt)2
Ph
N
Ph
2.00
1.01
3.04
6.06
3.94
2.09
6.39
f1 (ppm)

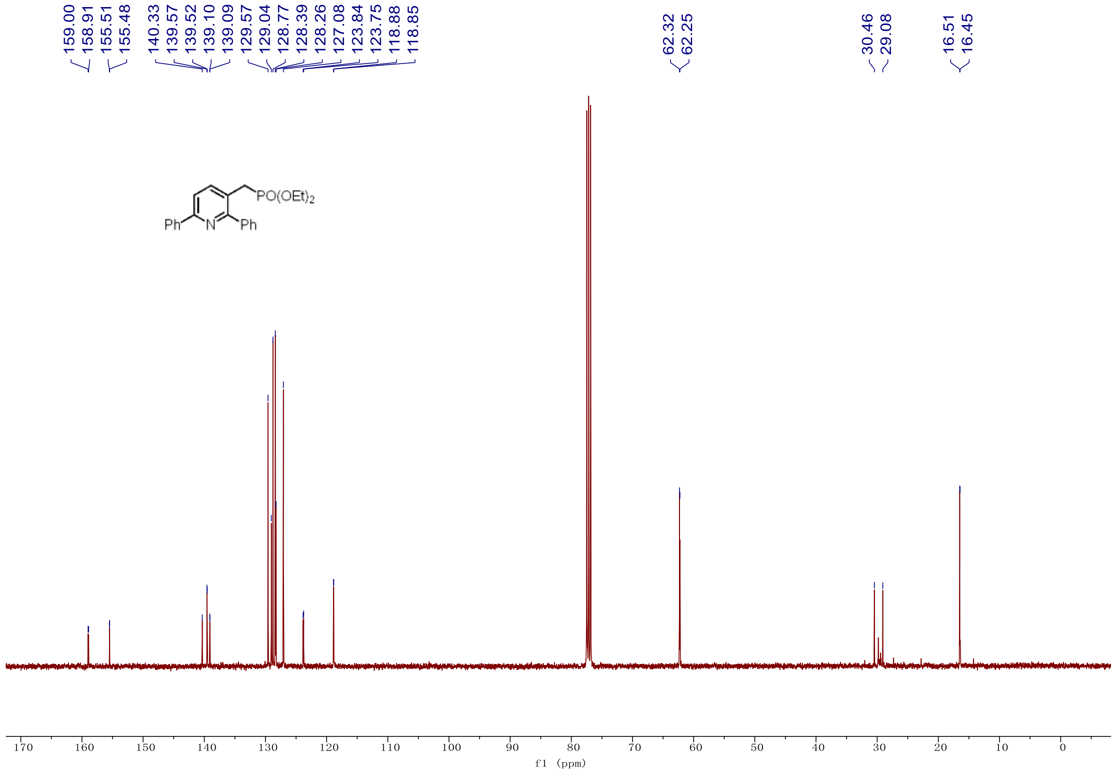
159.00
158.91
155.51
155.48
140.33
139.57
139.52
139.10
139.09
129.57
129.04
128.77
128.39
128.26
127.08
123.84
123.75
118.88
118.85
62.32
62.25
30.46
29.08
16.51
16.45
PO(OEt)2
Ph
N
Ph
f1 (ppm)

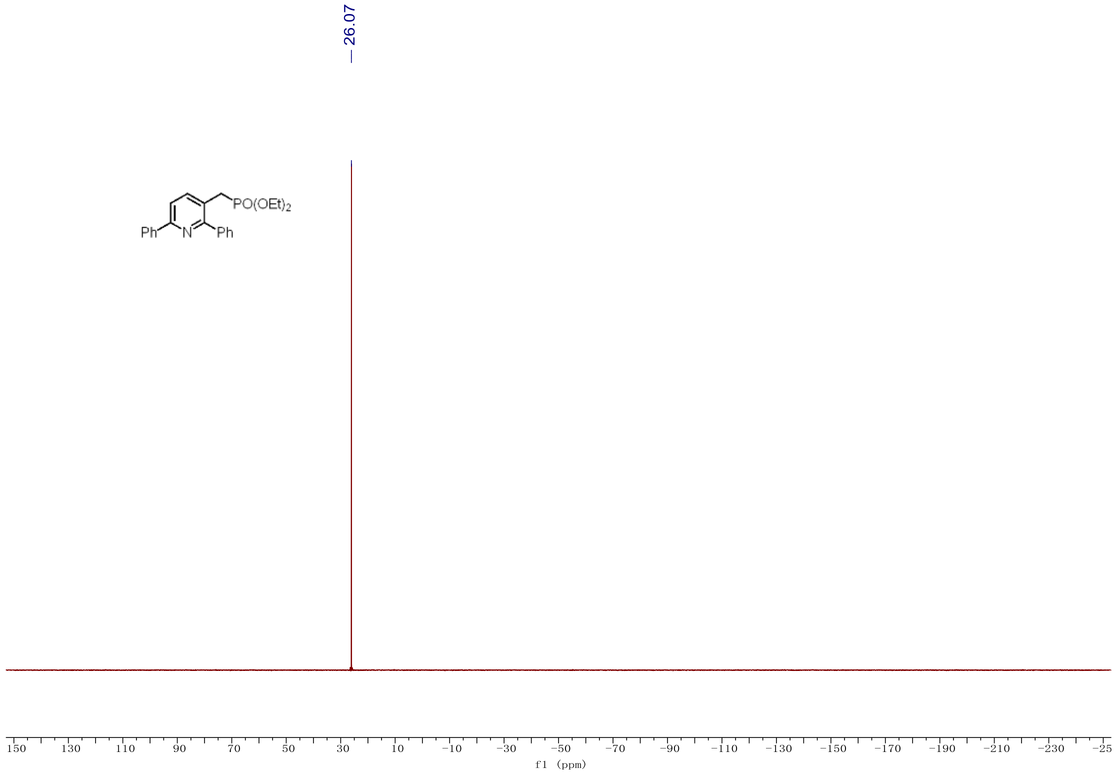
26.07
PO(OEt)2
Ph
N
Ph
f1 (ppm)

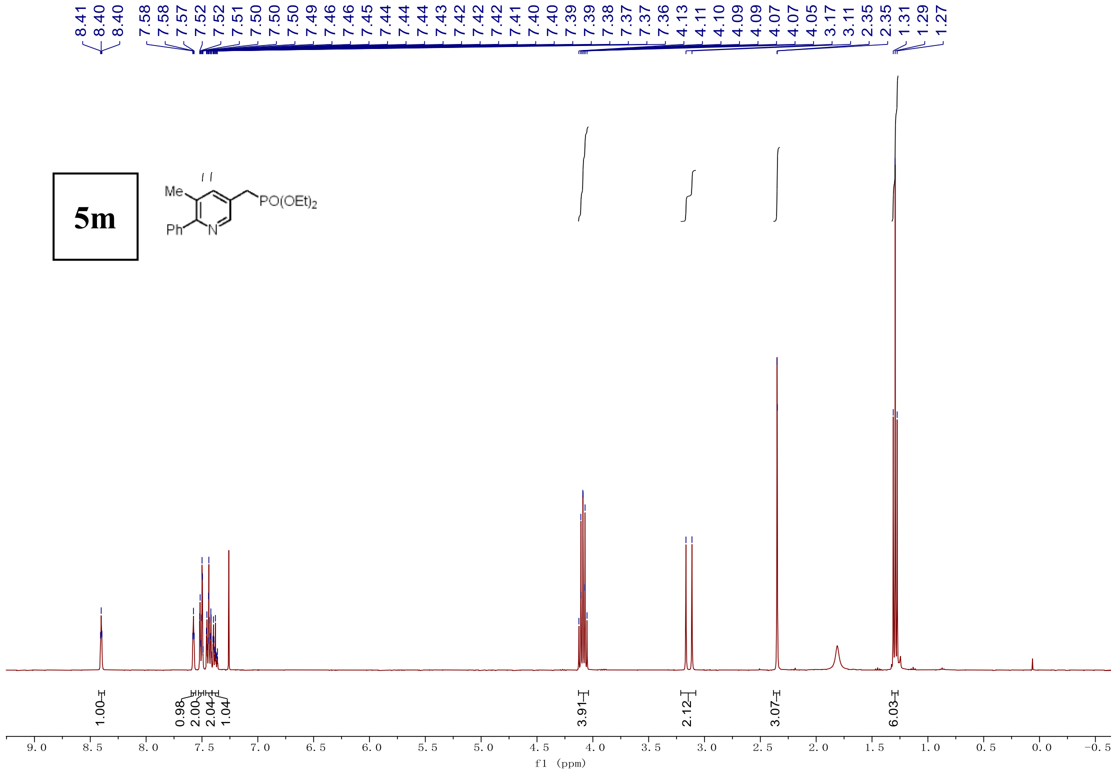
8.41
8.40
8.40
7.58
7.58
7.57
7.52
7.52
7.51
7.50
7.50
7.49
7.46
7.46
7.45
7.44
7.44
7.44
7.43
7.42
7.42
7.42
7.41
7.40
7.39
7.39
7.38
7.37
7.37
7.36
4.13
4.11
4.10
4.09
4.09
4.07
4.07
4.05
3.17
3.11
2.35
2.35
1.31
1.29
1.27
5m
Me
Ph
N
PO(OEt)2
1.00
0.98
2.00
2.04
1.04
3.91
2.12
3.07
6.03
f1 (ppm)

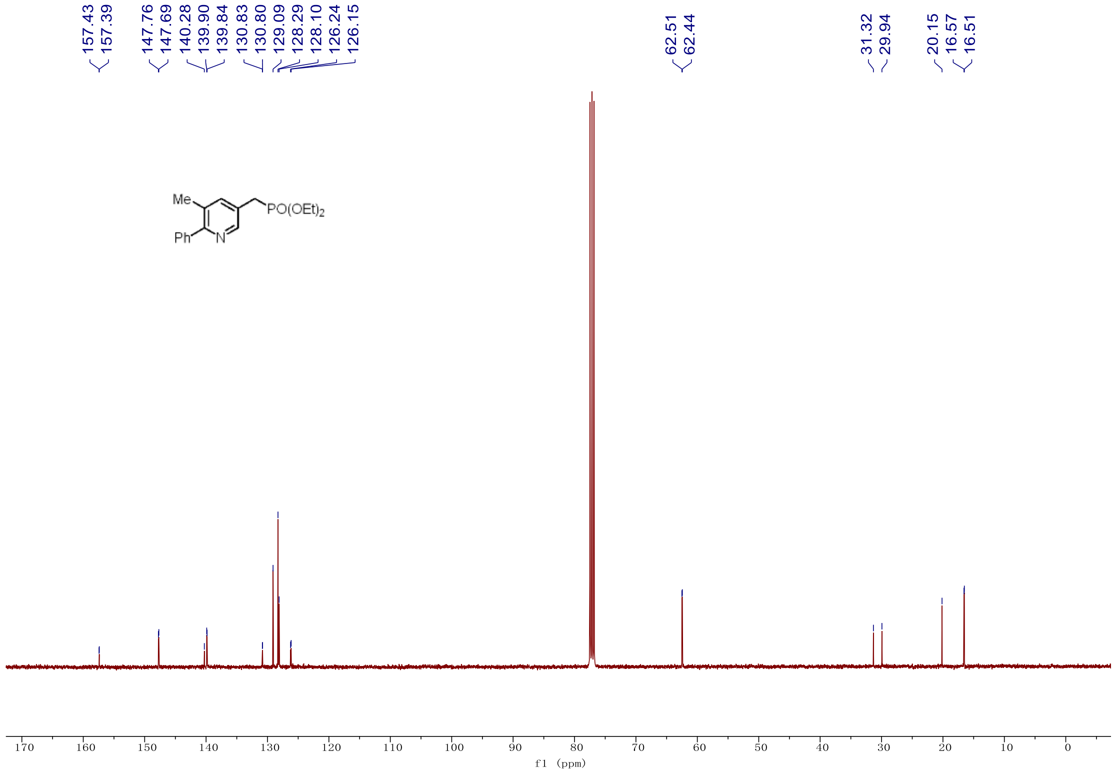
157.43
157.39
147.76
147.69
140.28
139.90
139.84
130.83
130.80
129.09
128.29
128.10
126.24
126.15
62.51
62.44
31.32
29.94
20.15
16.57
16.51
Me
Ph
N
PO(OEt)2
f1 (ppm)

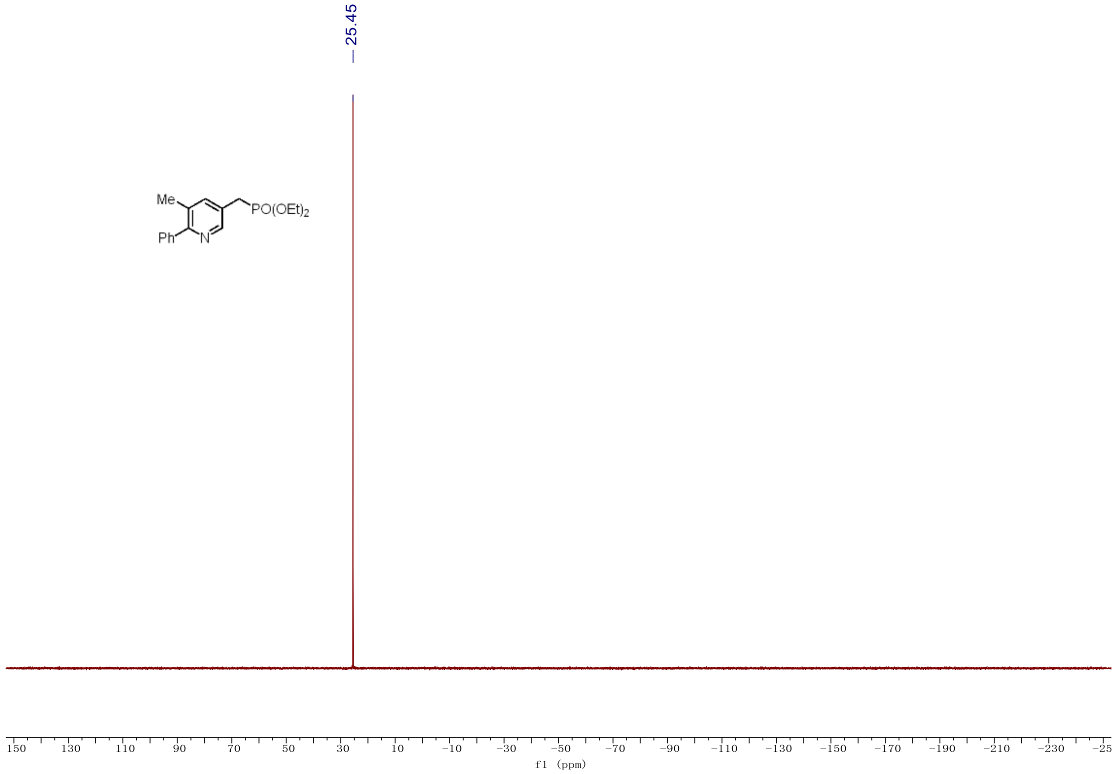
25.45
Me
PO(OEt)2
Ph
N
f1 (ppm)

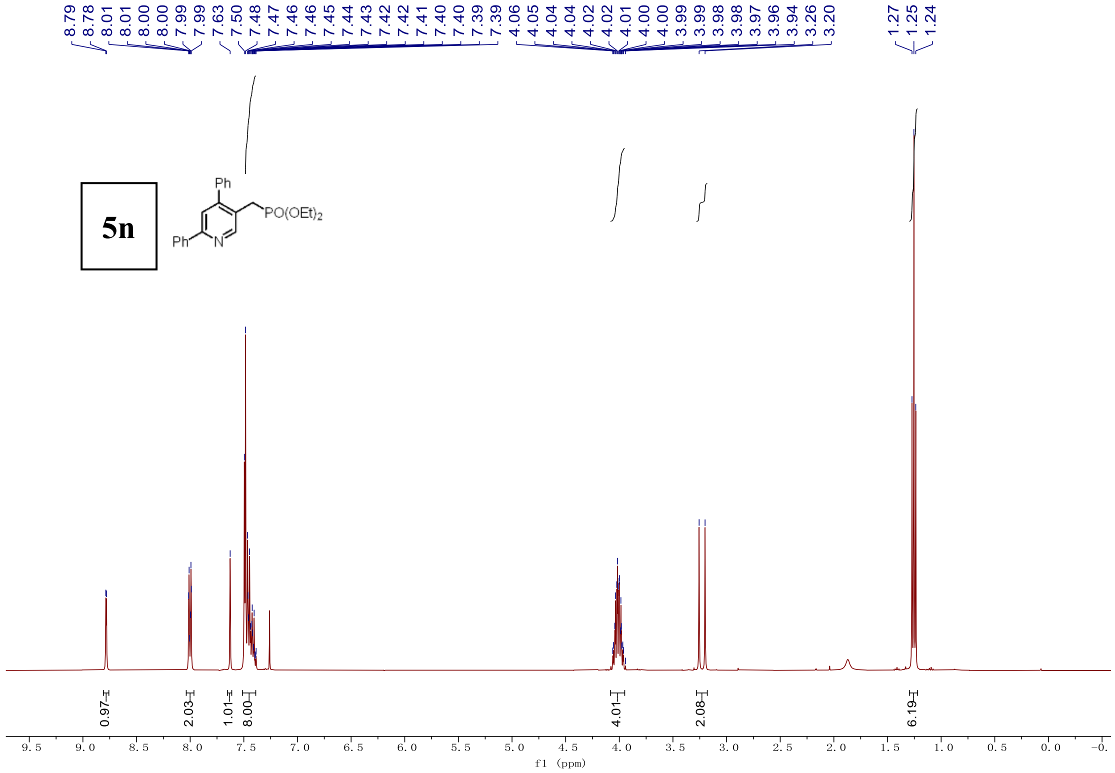
8.79
8.78
8.01
8.01
8.00
8.00
7.99
7.99
7.63
7.50
7.48
7.47
7.46
7.46
7.45
7.44
7.43
7.42
7.42
7.41
7.40
7.40
7.39
7.39
4.06
4.05
4.04
4.04
4.02
4.02
4.01
4.00
4.00
3.99
3.99
3.98
3.98
3.97
3.96
3.94
3.26
3.20
1.27
1.25
1.24
5n
Ph
PO(OEt)2
Ph
N
0.97
2.03
1.01
8.00
4.01
2.08
6.19
f1 (ppm)

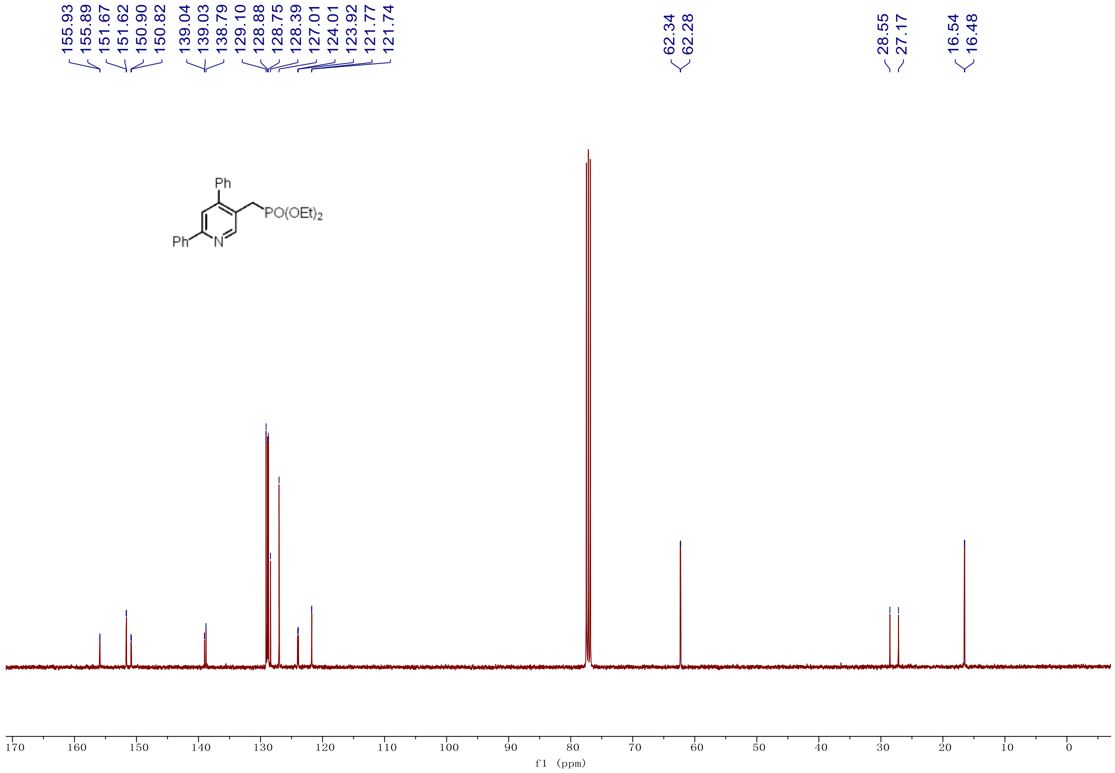
155.93
155.89
151.67
151.62
150.90
150.82
139.04
139.03
138.79
129.10
128.88
128.75
128.39
127.01
124.01
123.92
121.77
121.74
62.34
62.28
28.55
27.17
16.54
16.48
Ph
PO(OEt)2
Ph
N
f1 (ppm)

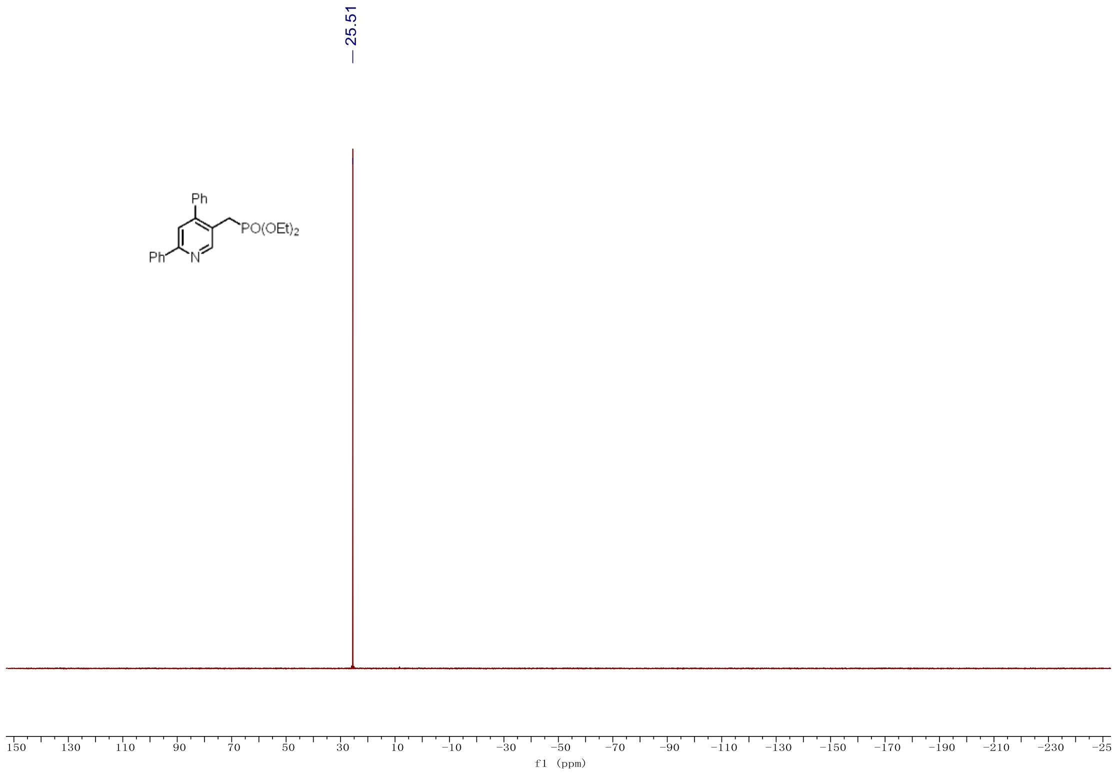
25.51
Ph
PO(OEt)2
Ph
N
f1 (ppm)

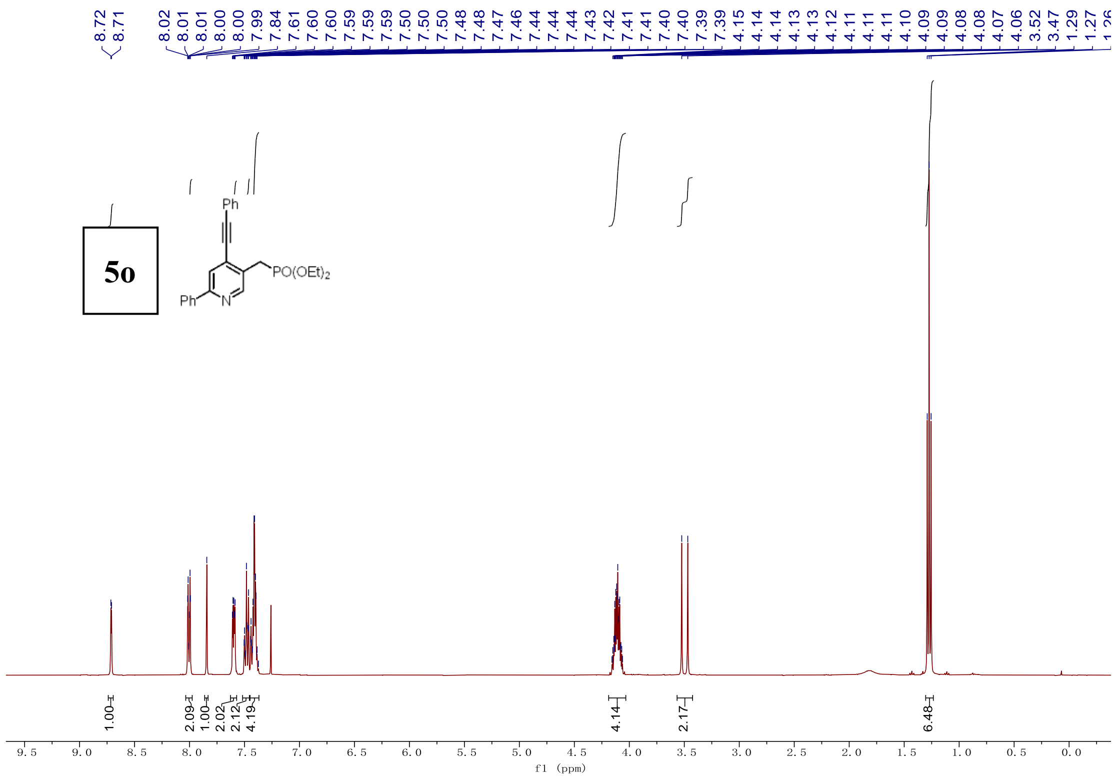
8.72
8.71
8.02
8.01
8.00
8.00
7.99
7.84
7.61
7.60
7.59
7.59
7.59
7.50
7.50
7.48
7.48
7.47
7.46
7.44
7.44
7.44
7.43
7.42
7.41
7.41
7.40
7.40
7.39
4.15
4.14
4.14
4.13
4.13
4.12
4.11
4.11
4.11
4.10
4.09
4.09
4.08
4.08
4.07
4.06
3.52
3.47
1.29
1.27
5o
Ph
PO(OEt)2
Ph
N
1.00
2.09
1.00
2.02
2.12
4.19
4.14
2.17
6.48
f1 (ppm)

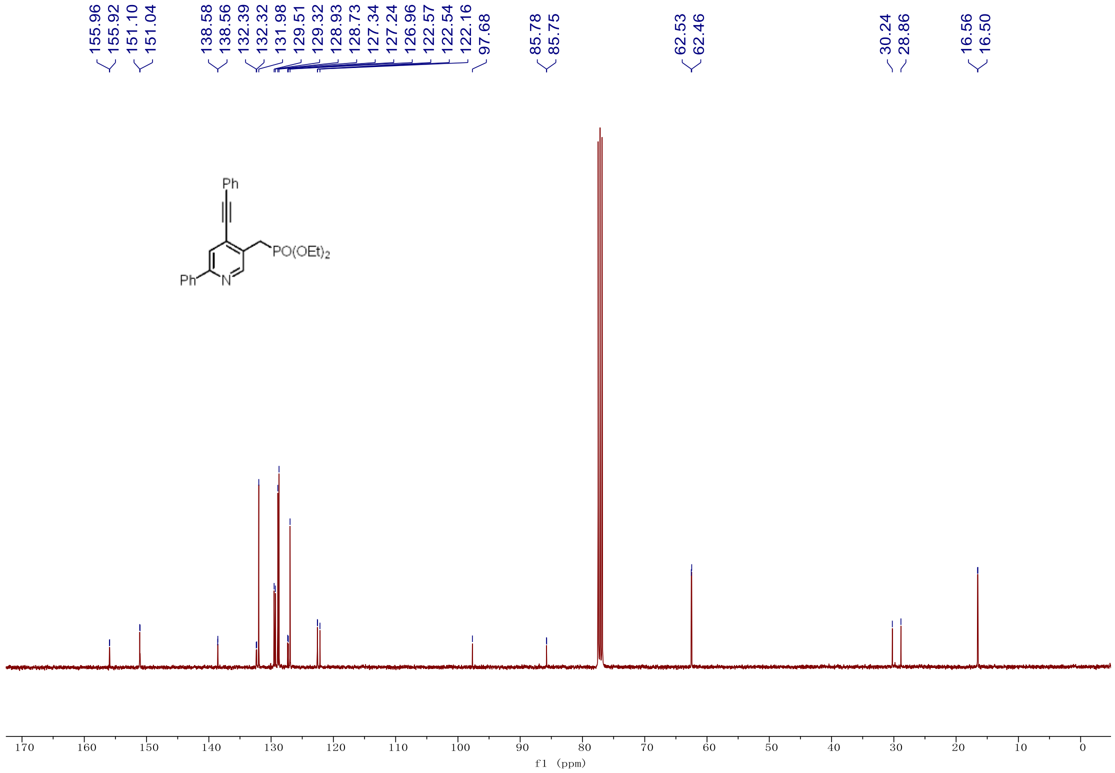
155.96
155.92
151.10
151.04
138.58
138.56
132.39
132.32
131.98
129.51
129.32
128.93
128.73
127.34
127.24
126.96
122.57
122.54
122.16
97.68
85.78
85.75
62.53
62.46
30.24
28.86
16.56
16.50
Ph
PO(OEt)2
Ph
N
f1 (ppm)

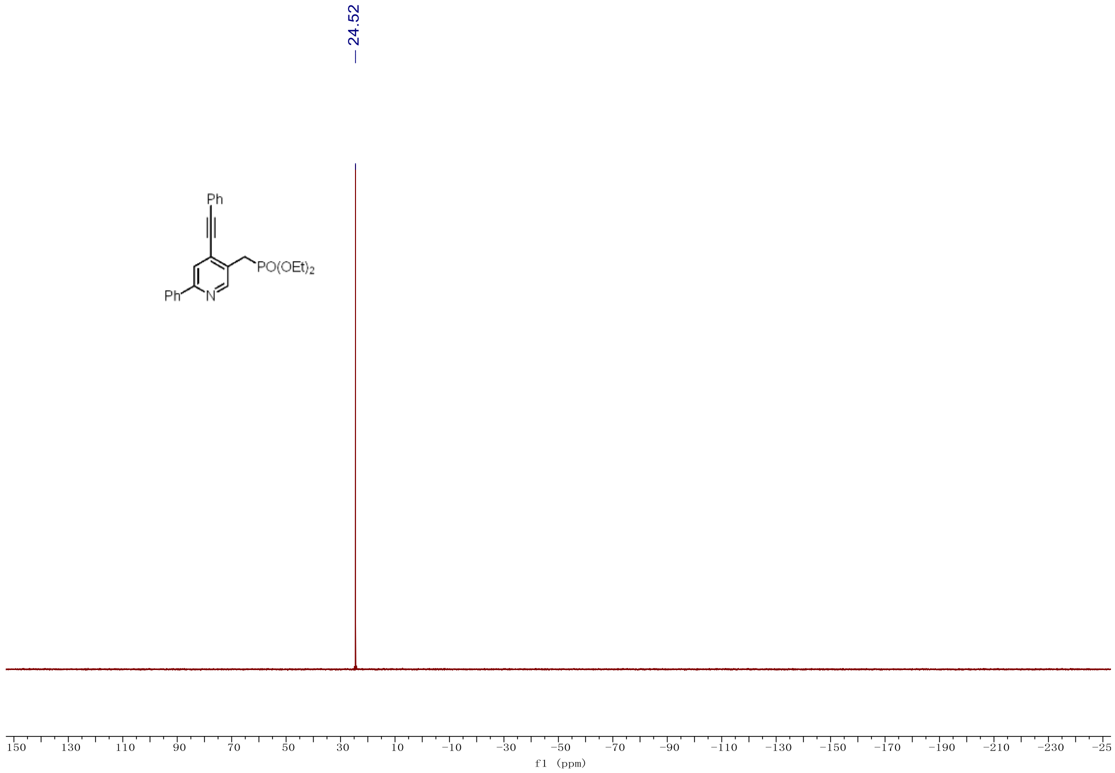
24.52
Ph
PO(OEt)2
Ph
N
f1 (ppm)

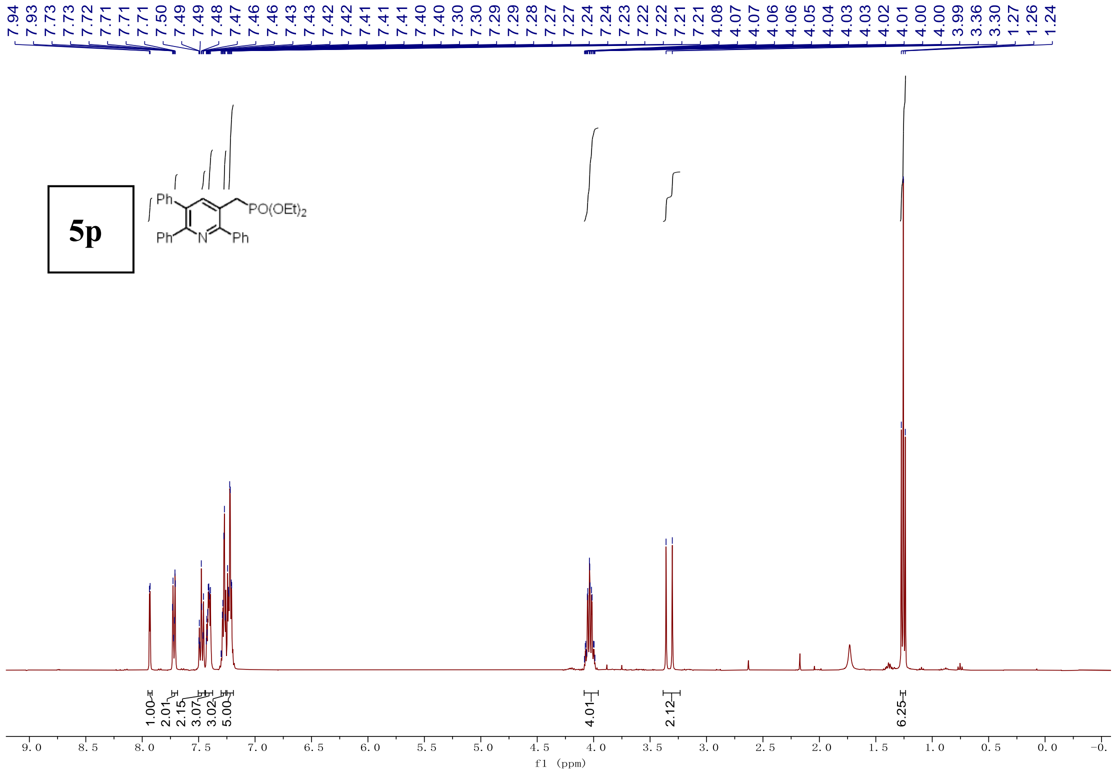
Ph
PO(OEt)2
Ph
N
Ph
1.00
2.01
2.15
3.07
3.02
5.00
4.01
2.12
6.25
f1 (ppm)

5p

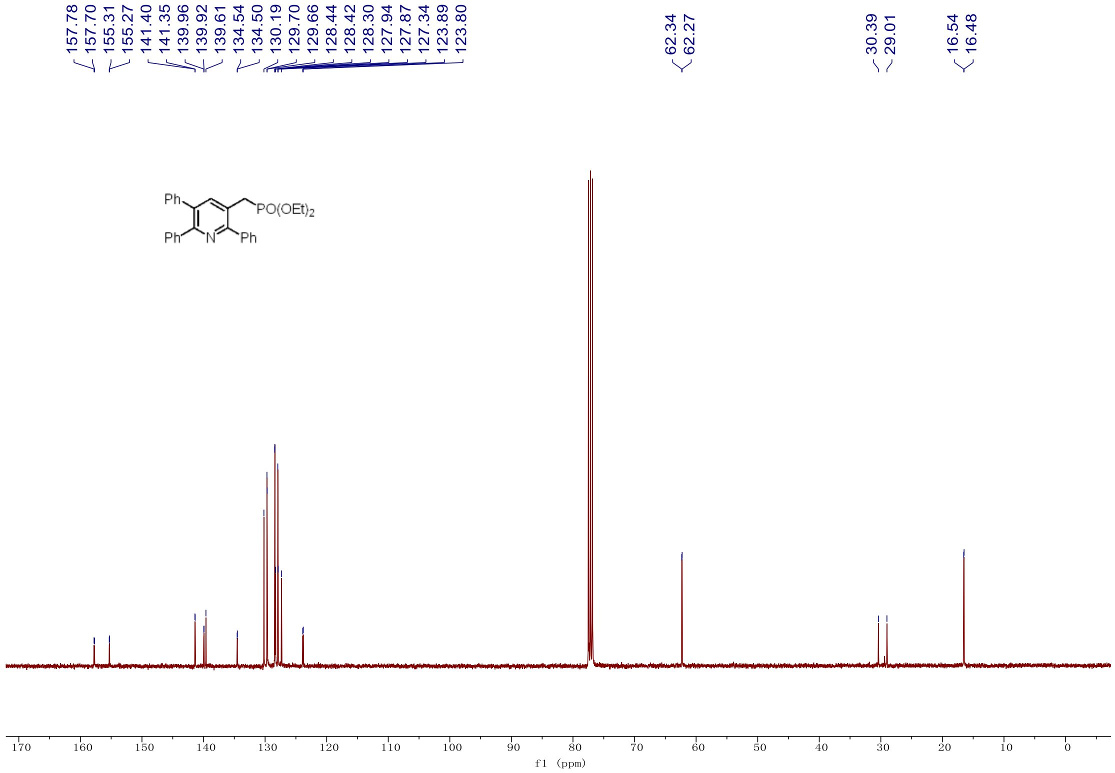
157.78
157.70
155.31
155.27
141.40
141.35
139.96
139.92
139.61
134.54
134.50
130.19
129.70
129.66
128.44
128.42
128.30
127.94
127.87
127.34
123.89
123.80
62.34
62.27
30.39
29.01
16.54
16.48
Ph
PO(OEt)2
Ph
N
Ph
f1 (ppm)

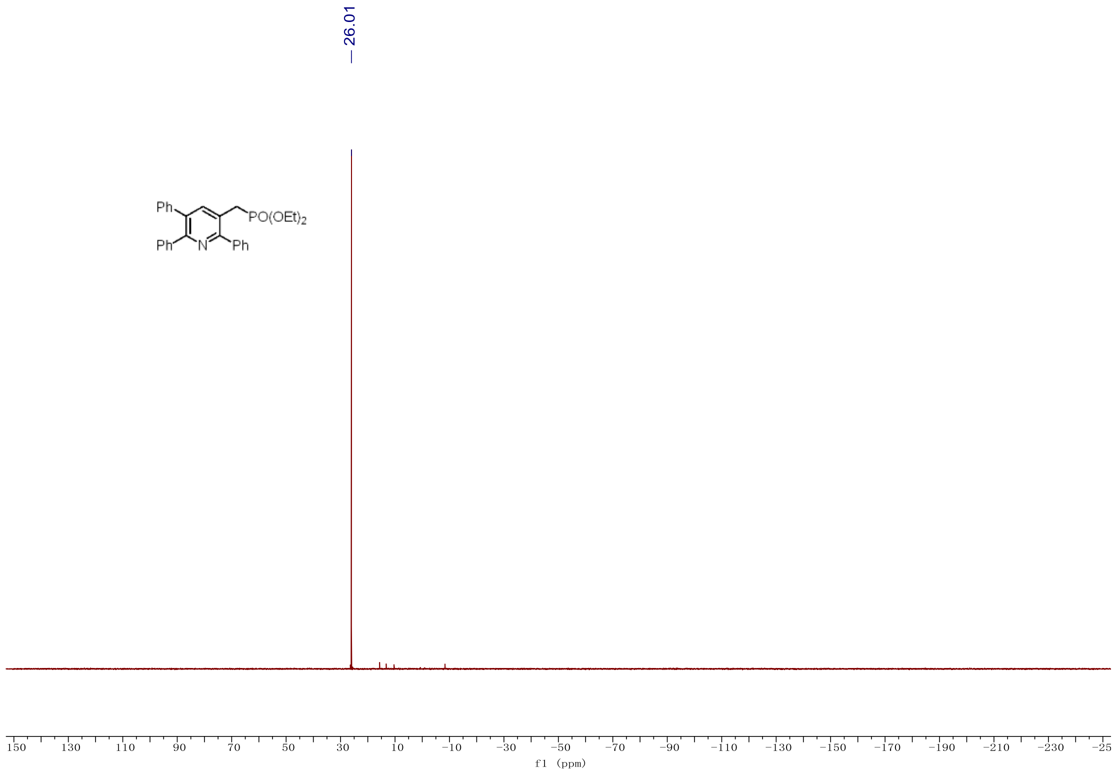
26.01
Ph
PO(OEt)2
Ph
N
Ph
f1 (ppm)

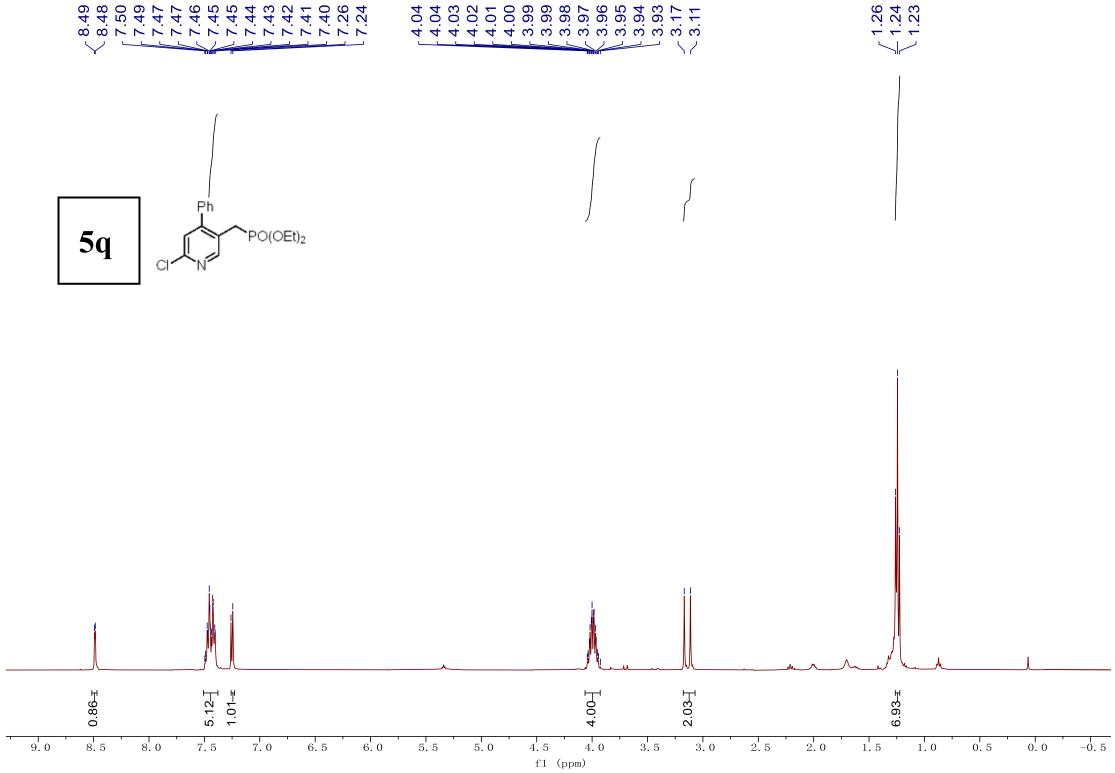
8.49
8.48
7.50
7.49
7.47
7.47
7.46
7.45
7.45
7.44
7.43
7.42
7.41
7.40
7.26
7.24
4.04
4.04
4.03
4.02
4.01
4.00
3.99
3.99
3.98
3.97
3.96
3.95
3.94
3.93
3.17
3.11
1.26
1.24
1.23
5q
Ph
PO(OEt)2
Cl
N
0.86
5.12
1.01
4.00
2.03
6.93
f1 (ppm)

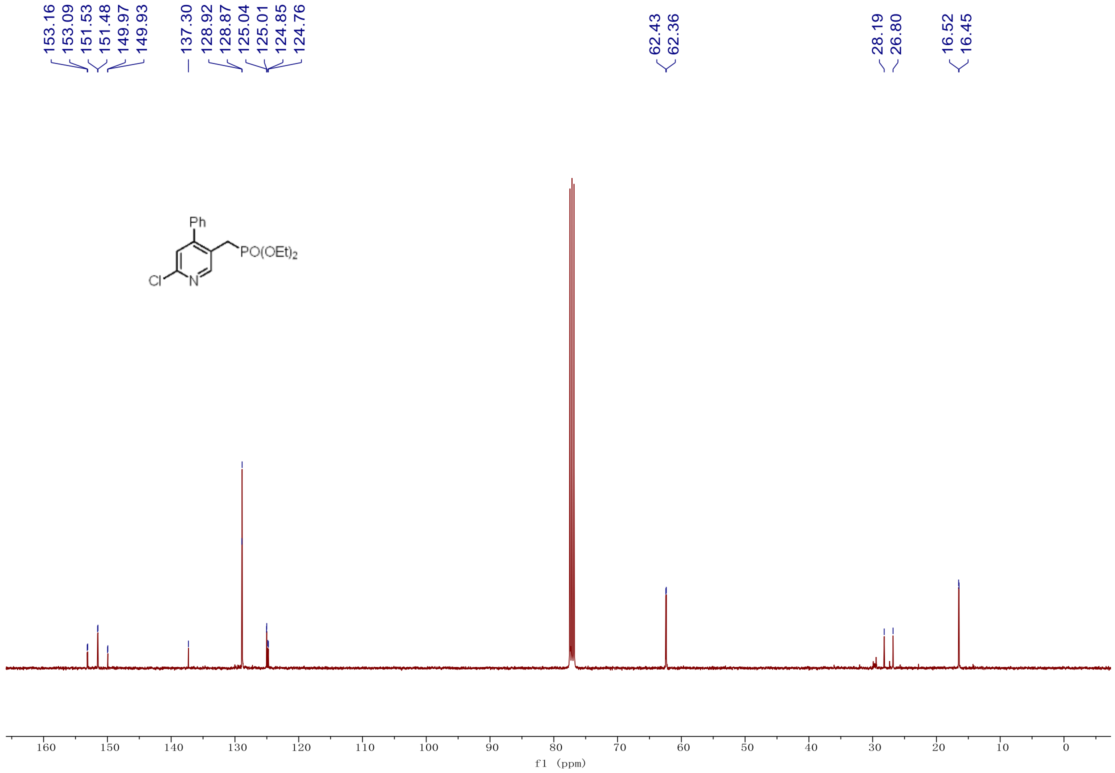
153.16
153.09
151.53
151.48
149.97
149.93
137.30
128.92
128.87
125.04
125.01
124.85
124.76
62.43
62.36
28.19
26.80
16.52
16.45
Ph
PO(OEt)2
Cl
N
f1 (ppm)

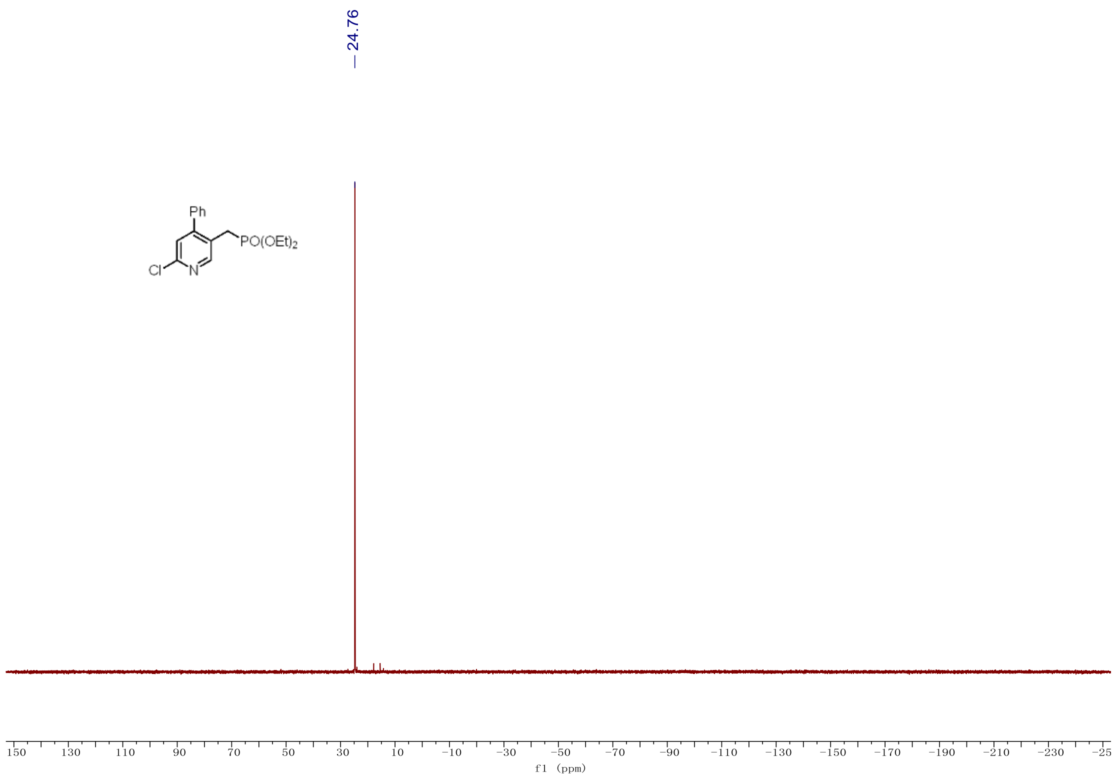
24.76
Ph
PO(OEt)2
Cl
N
f1 (ppm)

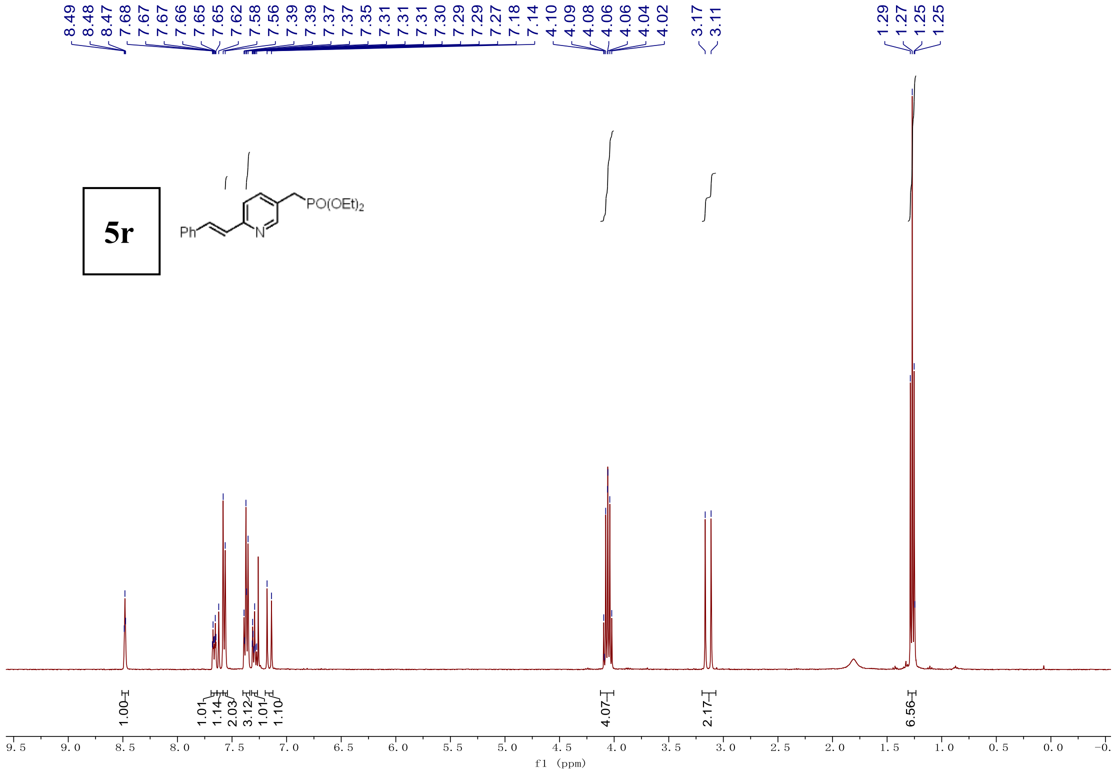
8.49 8.48 8.47 7.68 7.67 7.66 7.65 7.65 7.62 7.58 7.56 7.39 7.39 7.37 7.37 7.35 7.31 7.31 7.30 7.29 7.29 7.27 7.18 7.14 4.10 4.09 4.08 4.06 4.06 4.04 4.02 3.17 3.11 1.29 1.27 1.25 1.25
5r
Ph
N
PO(OEt)2
1.00 1.01 1.14 2.03 3.12 1.01 1.10 4.07 2.17 6.56
f1 (ppm)

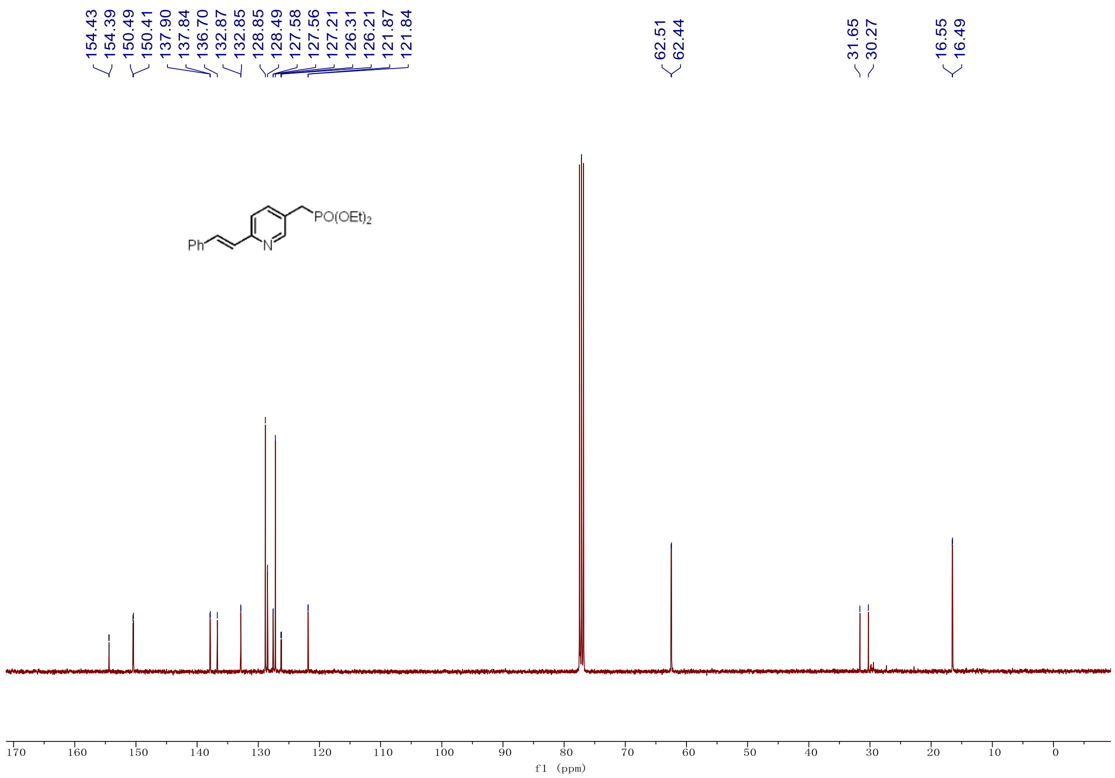
154.43
154.39
150.49
150.41
137.90
137.84
136.70
132.87
132.85
128.85
128.49
127.58
127.56
127.21
126.31
126.21
121.87
121.84
62.51
62.44
31.65
30.27
16.55
16.49
PO(OEt)2
Ph
N
f1 (ppm)

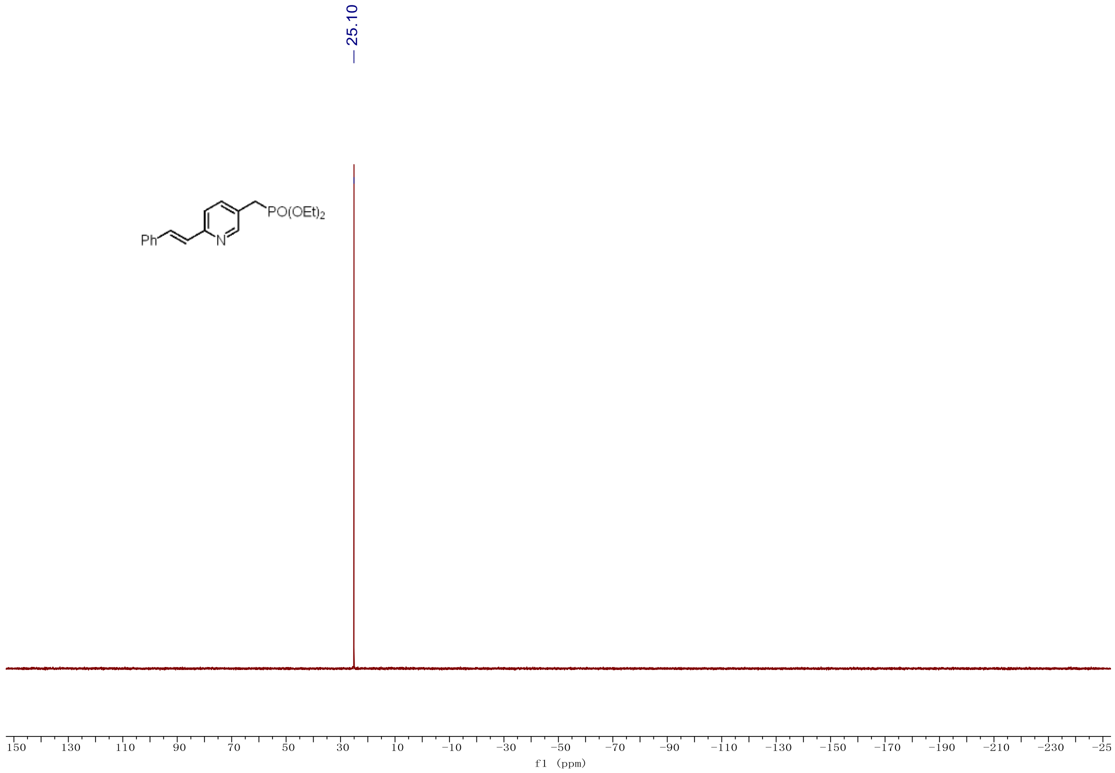
25.10
PO(OEt)2
Ph
N
f1 (ppm)

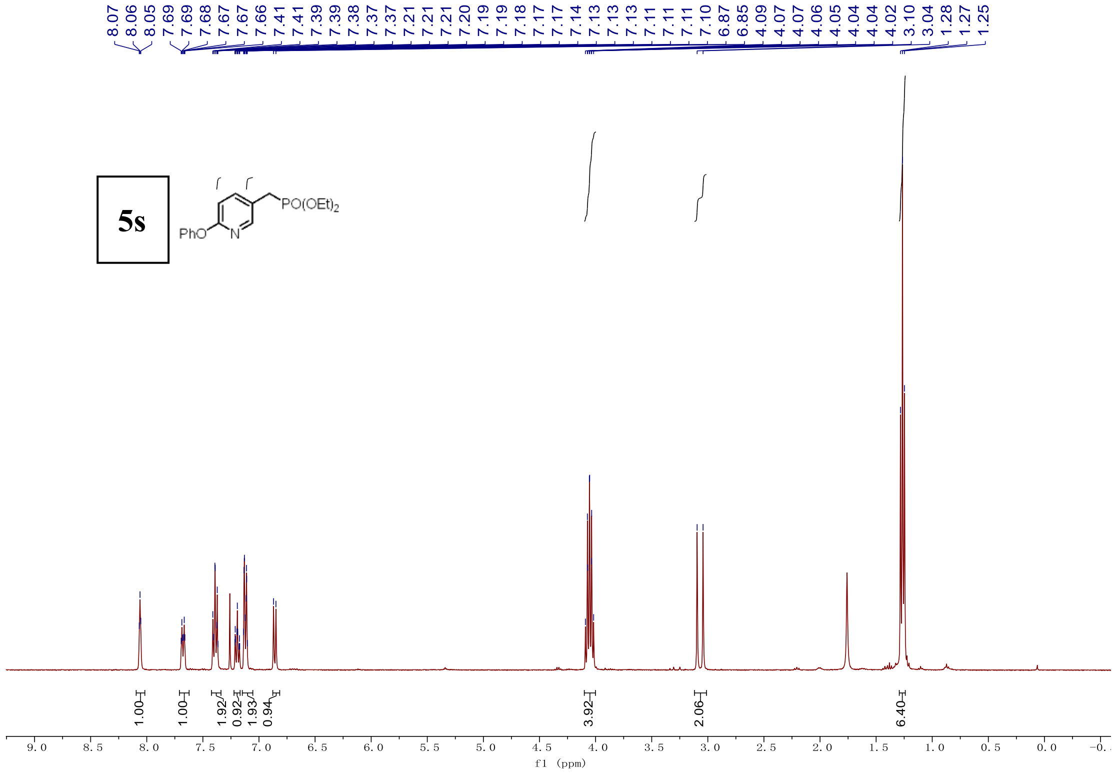
8.07 8.06 8.05 7.69 7.69 7.68 7.67 7.67 7.66 7.41 7.41 7.39 7.39 7.38 7.37 7.37 7.21 7.21 7.21 7.20 7.19 7.19 7.18 7.17 7.17 7.14 7.13 7.13 7.13 7.11 7.11 7.11 7.10 6.87 6.85 4.09 4.07 4.07 4.06 4.05 4.04 4.04 4.02 3.10 3.04 1.28 1.27 1.25
5s
PhO
N
PO(OEt)2
1.00
1.00
1.92
0.92
1.93
0.94
3.92
2.06
6.40
f1 (ppm)

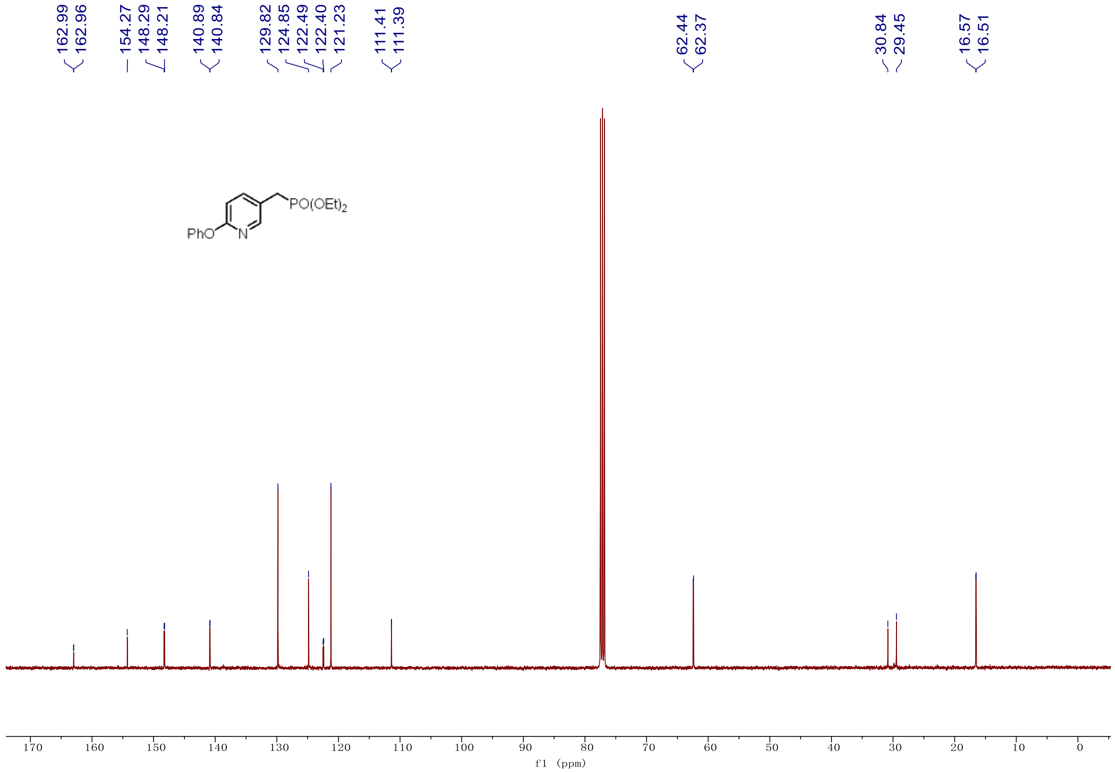
162.99 162.96 154.27 148.29 148.21 140.89 140.84 129.82 124.85 122.49 122.40 121.23 111.41 111.39 62.44 62.37 30.84 29.45 16.57 16.51
PhO
N
PO(OEt)2
f1 (ppm)

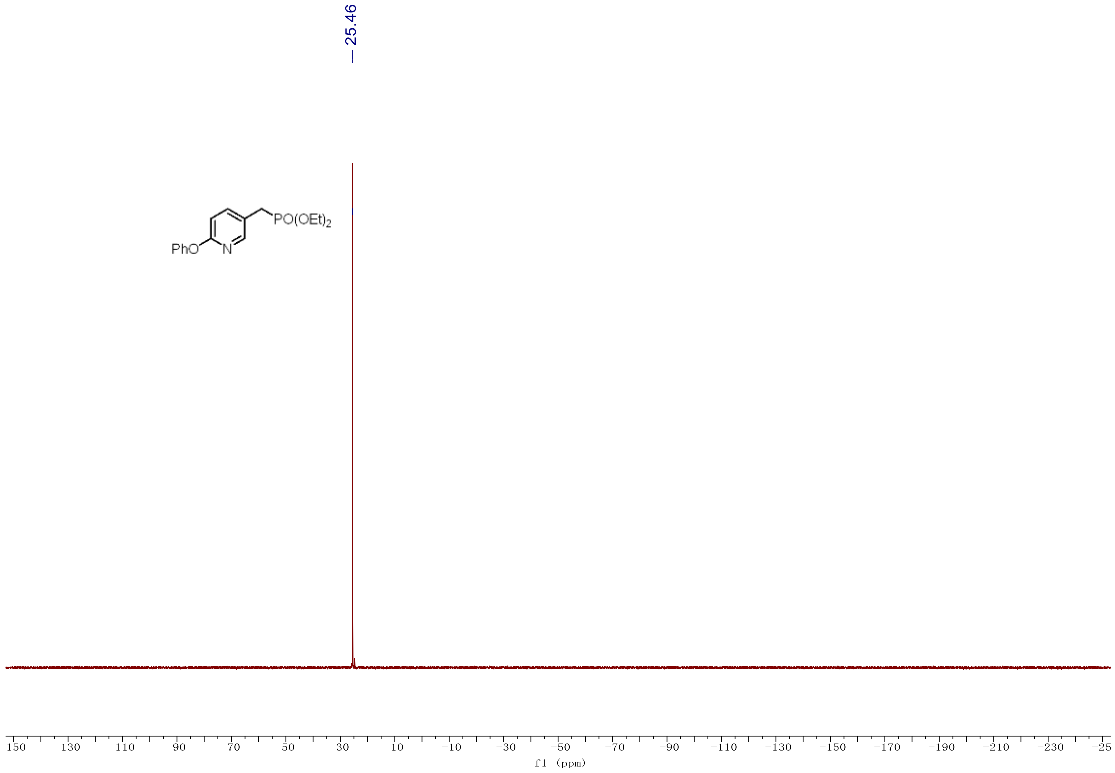
25.46
PO(OEt)2
PhO
N
f1 (ppm)

5t

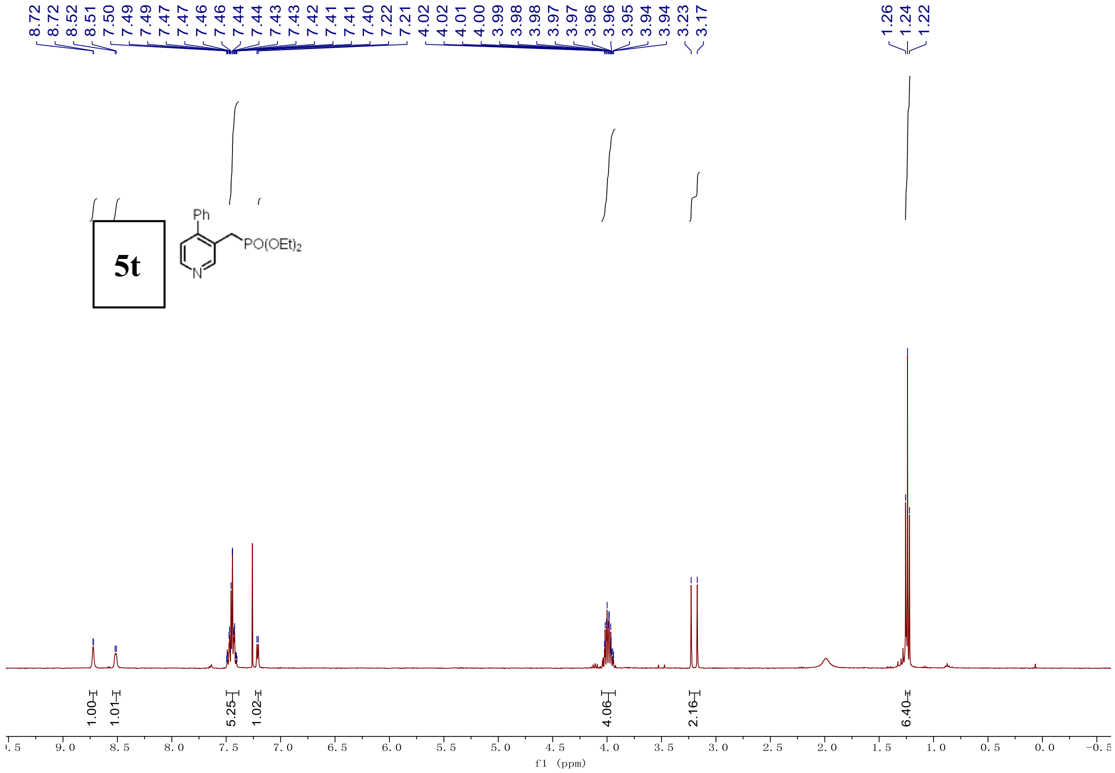
8.72
8.72
8.52
8.51
7.50
7.49
7.49
7.47
7.47
7.46
7.46
7.44
7.44
7.43
7.43
7.42
7.41
7.41
7.40
7.22
7.21
4.02
4.02
4.01
4.00
3.99
3.98
3.98
3.97
3.97
3.96
3.96
3.95
3.94
3.94
3.23
3.17
1.26
1.24
1.22
Ph
PO(OEt)2
N
1.00
1.01
5.25
1.02
4.06
2.16
6.40
f1 (ppm)

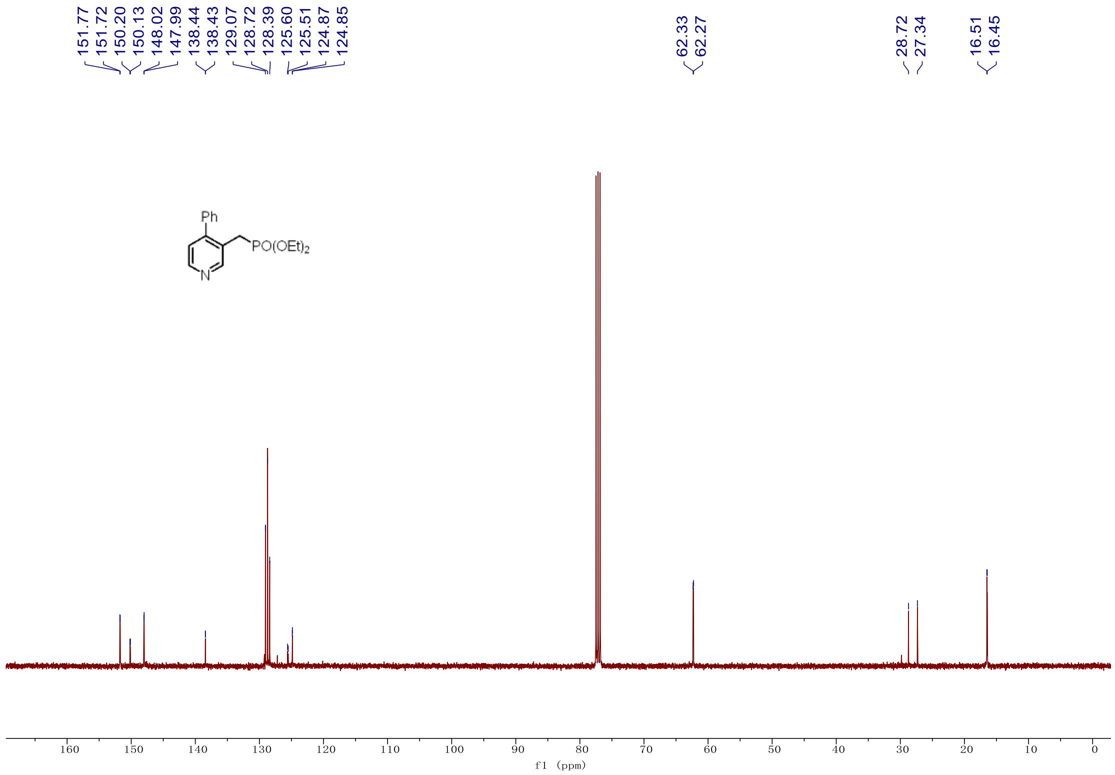
151.77
151.72
150.20
150.13
148.02
147.99
138.44
138.43
129.07
128.72
128.39
125.60
125.51
124.87
124.85
62.33
62.27
28.72
27.34
16.51
16.45
Ph
PO(OEt)2
N
160 150 140 130 120 110 100 90 80 70 60 50 40 30 20 10 0
f1 (ppm)

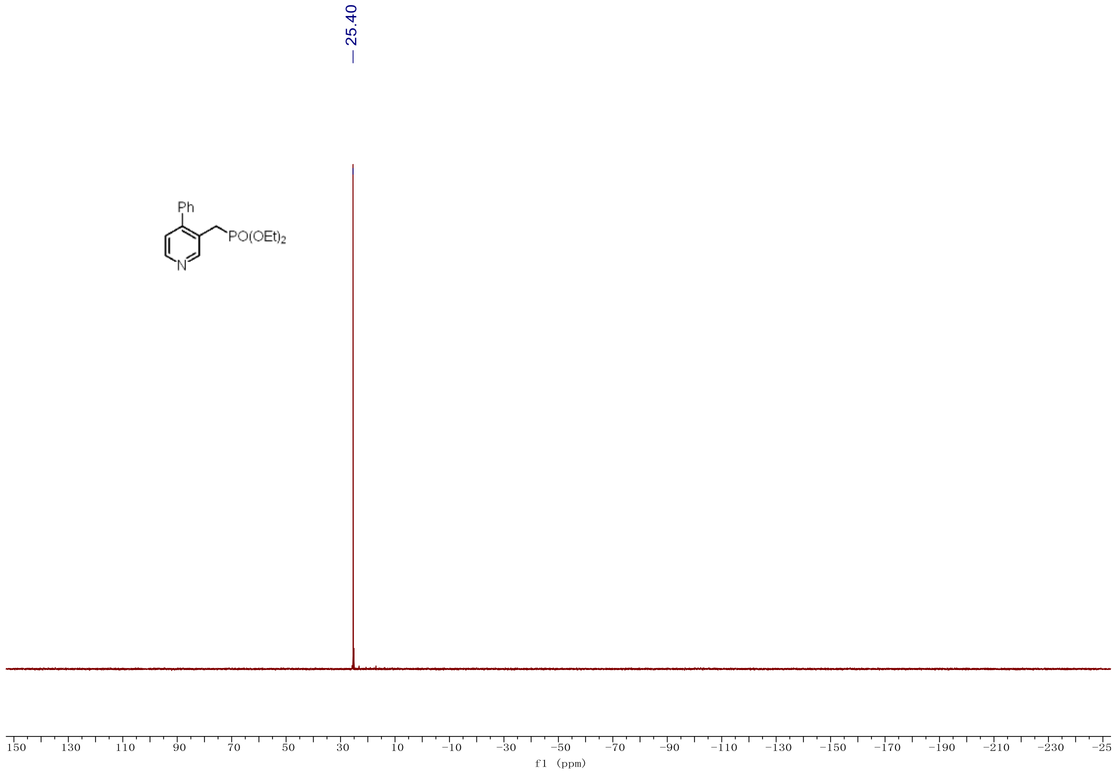
25.40
Ph
PO(OEt)2
N
150 130 110 90 70 50 30 10 −10 −30 −50 −70 −90 −110 −130 −150 −170 −190 −210 −230 −25
f1 (ppm)

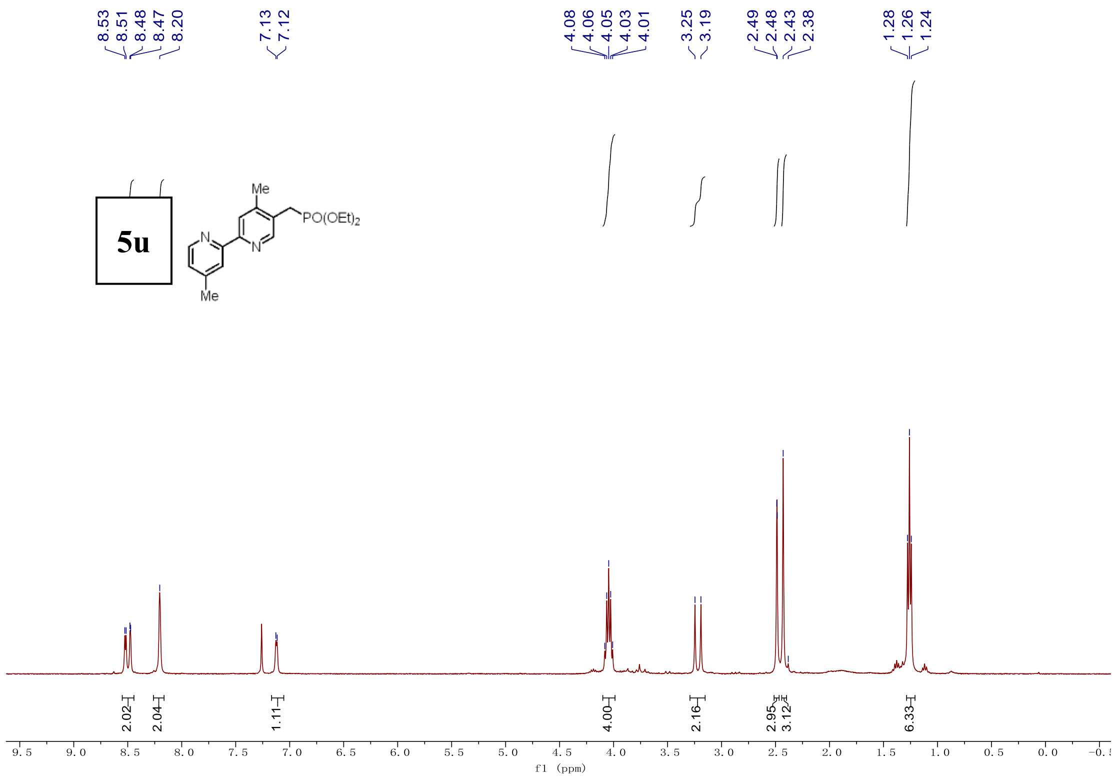
8.53
8.51
8.48
8.47
8.20
7.13
7.12
4.08
4.06
4.05
4.03
4.01
3.25
3.19
2.49
2.48
2.43
2.38
1.28
1.26
1.24
5u
Me
PO(OEt)$_2$
N
N
Me
2.02
2.04
1.11
4.00
2.16
2.95
3.12
6.33
f1 (ppm)

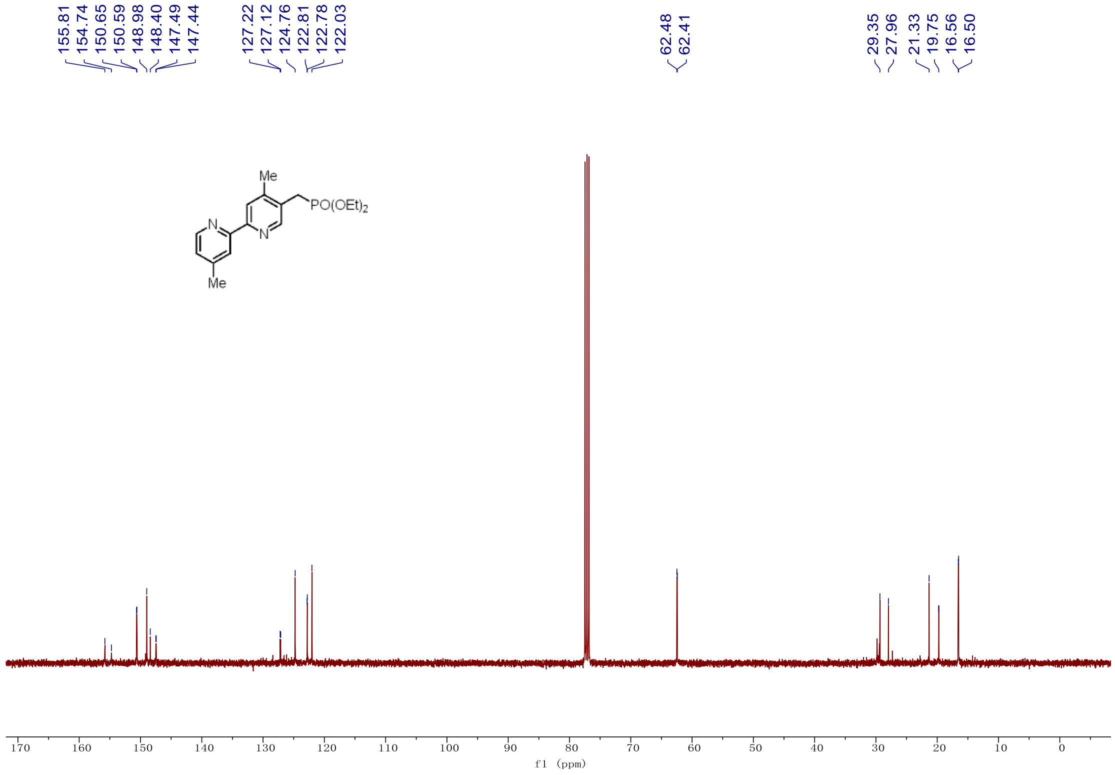
155.81
154.74
150.65
150.59
148.98
148.40
147.49
147.44
127.22
127.12
124.76
122.81
122.78
122.03
62.48
62.41
29.35
27.96
21.33
19.75
16.56
16.50
Me
PO(OEt)$_2$
N
N
Me
f1 (ppm)

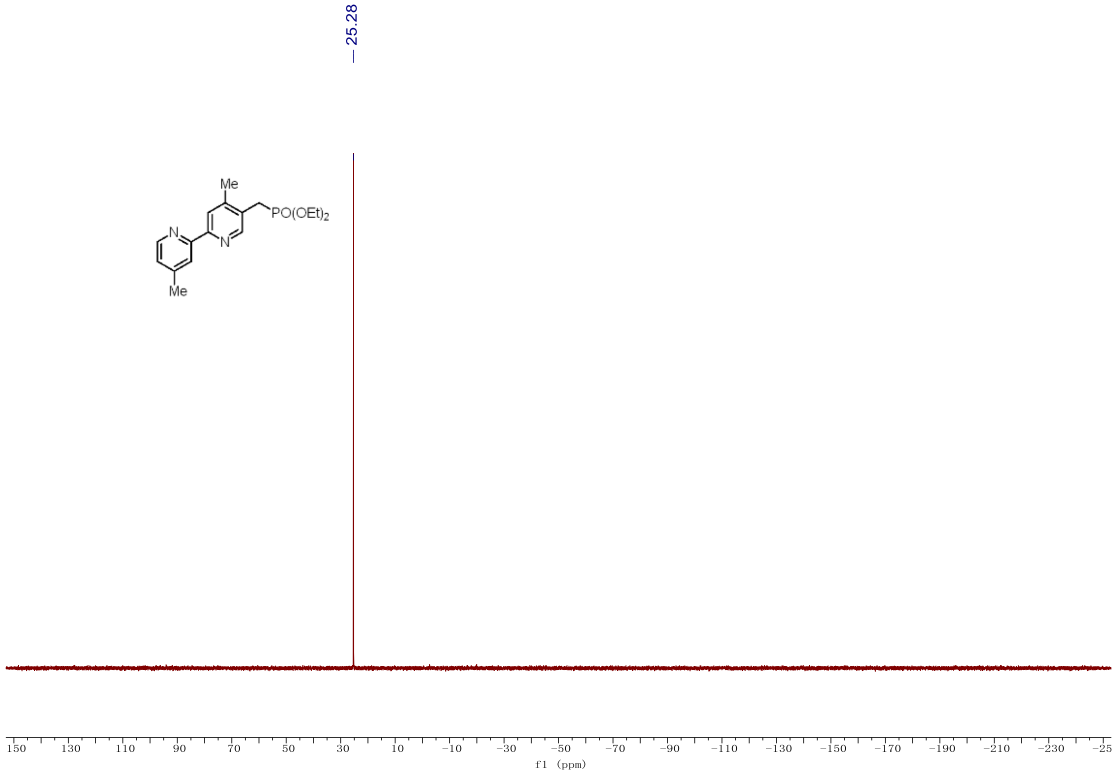

25.28
Me
PO(OEt)2
N
N
Me
f1 (ppm)

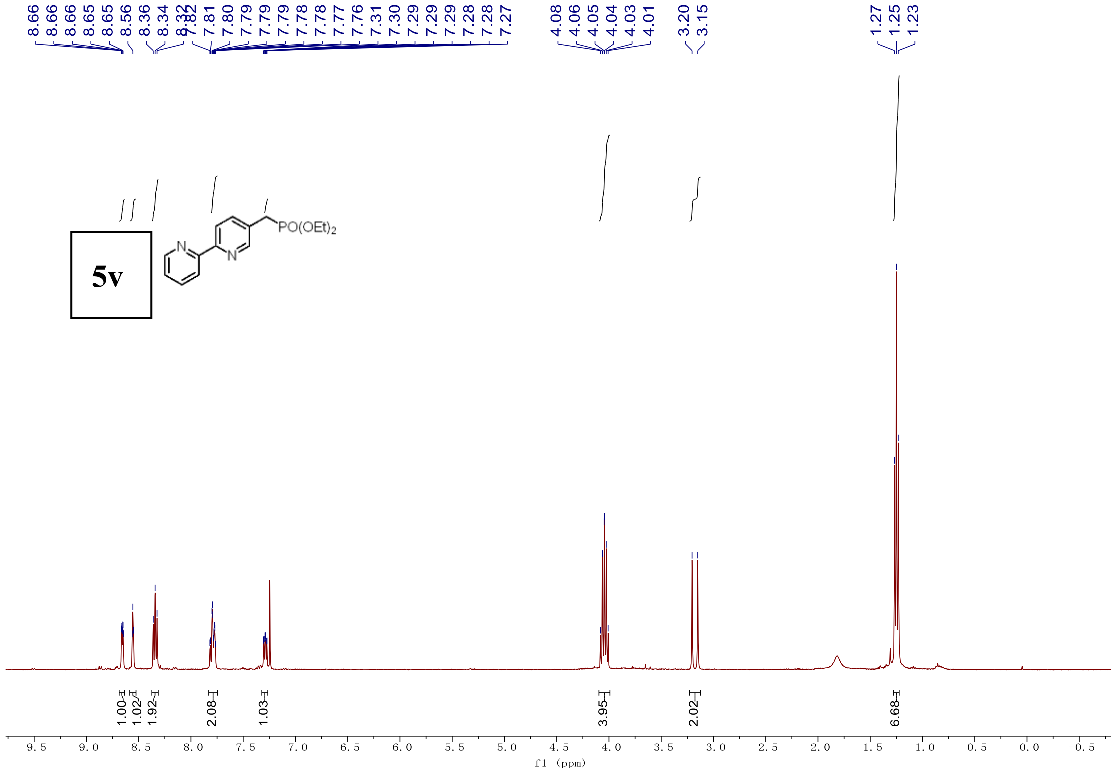

8.66
8.66
8.66
8.65
8.56
8.36
8.34
8.32
7.81
7.80
7.79
7.79
7.78
7.78
7.77
7.76
7.31
7.30
7.29
7.29
7.28
7.28
7.27
4.08
4.06
4.05
4.04
4.03
4.01
3.20
3.15
1.27
1.25
1.23
5v
PO(OEt)2
N
N
1.00
1.02
1.92
2.08
1.03
3.95
2.02
6.68
f1 (ppm)

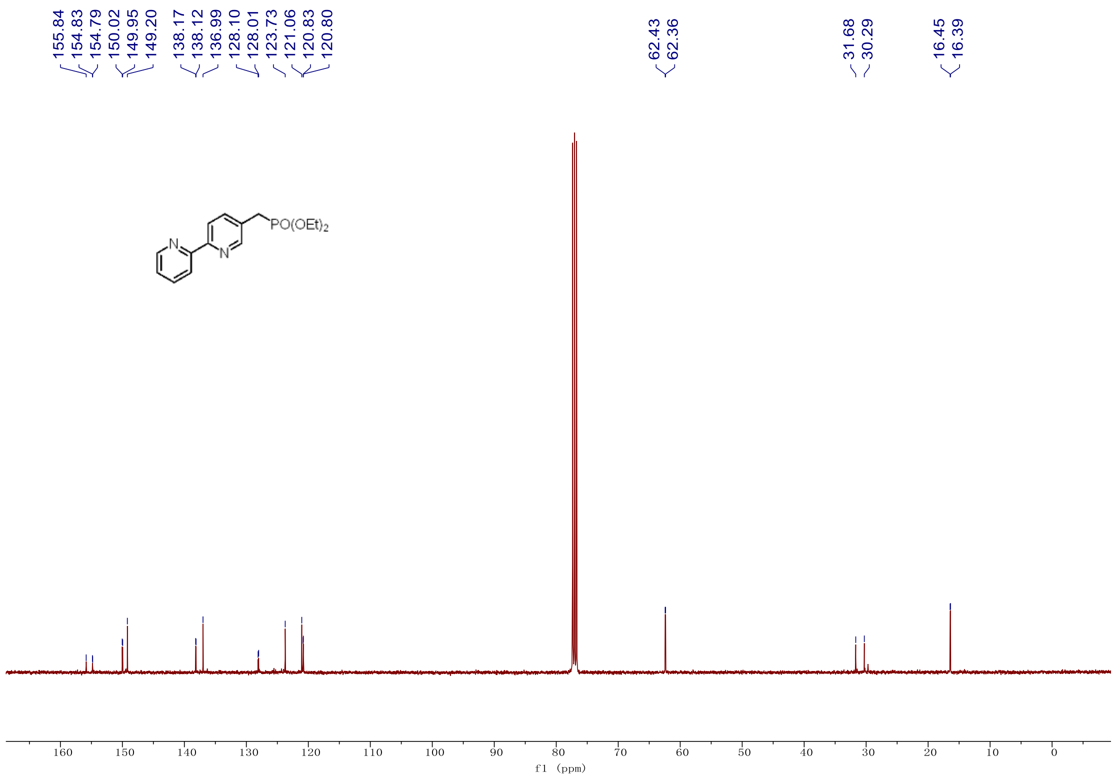
155.84
154.83
154.79
150.02
149.95
149.20
138.17
138.12
136.99
128.10
128.01
123.73
121.06
120.83
120.80
62.43
62.36
31.68
30.29
16.45
16.39
PO(OEt)2
N
N
f1 (ppm)

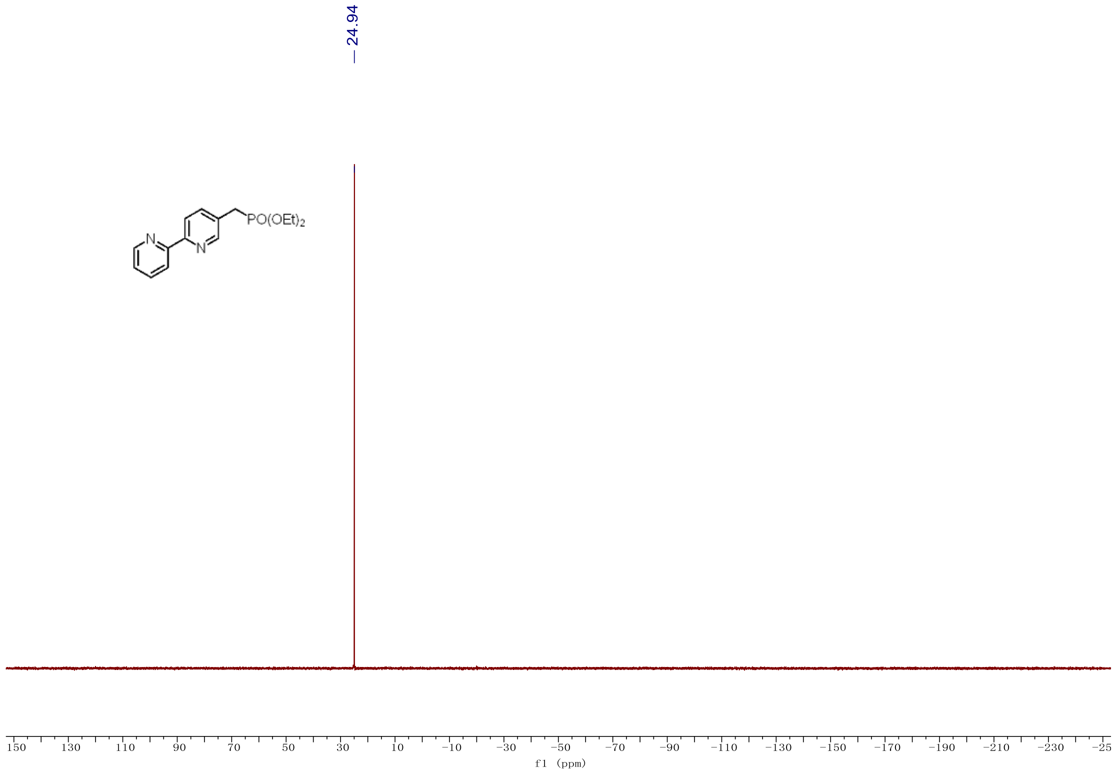
24.94
PO(OEt)2
N
N
f1 (ppm)

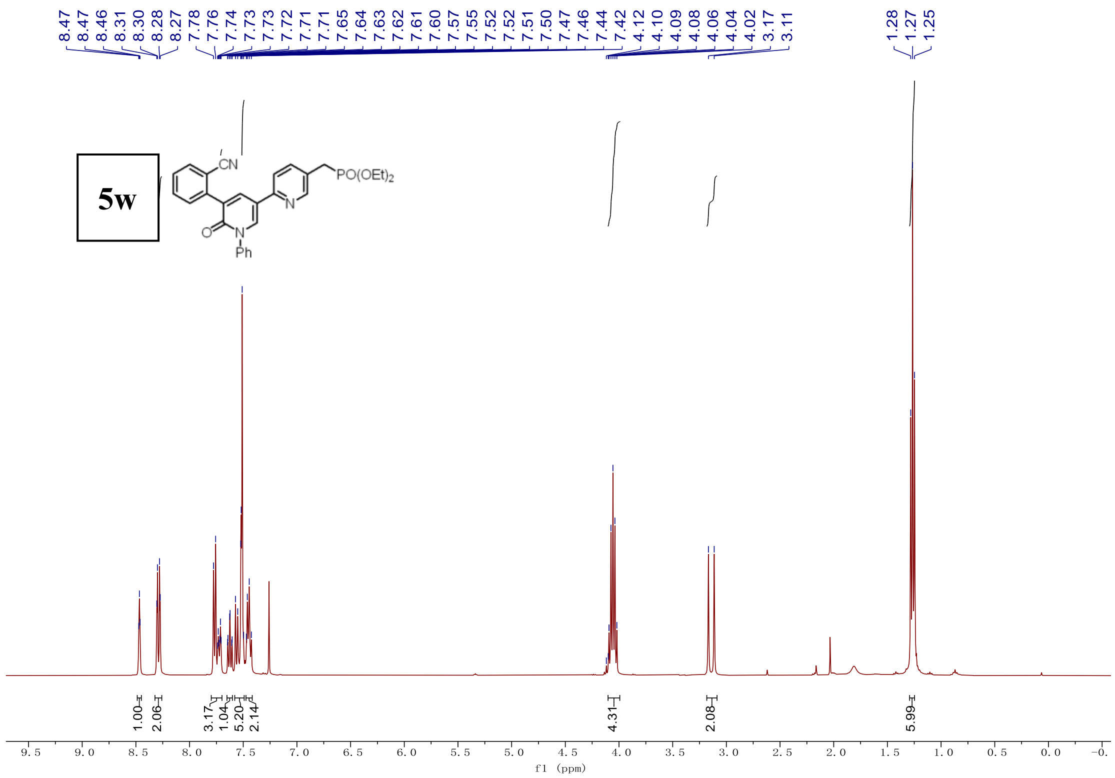
8.47
8.47
8.46
8.31
8.30
8.28
8.27
7.78
7.76
7.74
7.73
7.73
7.72
7.71
7.71
7.65
7.64
7.63
7.62
7.61
7.60
7.57
7.55
7.52
7.52
7.51
7.50
7.47
7.46
7.44
7.42
4.12
4.10
4.09
4.08
4.06
4.04
4.02
3.17
3.11
1.28
1.27
1.25
5w
CN
PO(OEt)2
N
O
N
Ph
1.00
2.06
3.17
1.04
5.20
2.14
4.31
2.08
5.99
f1 (ppm)

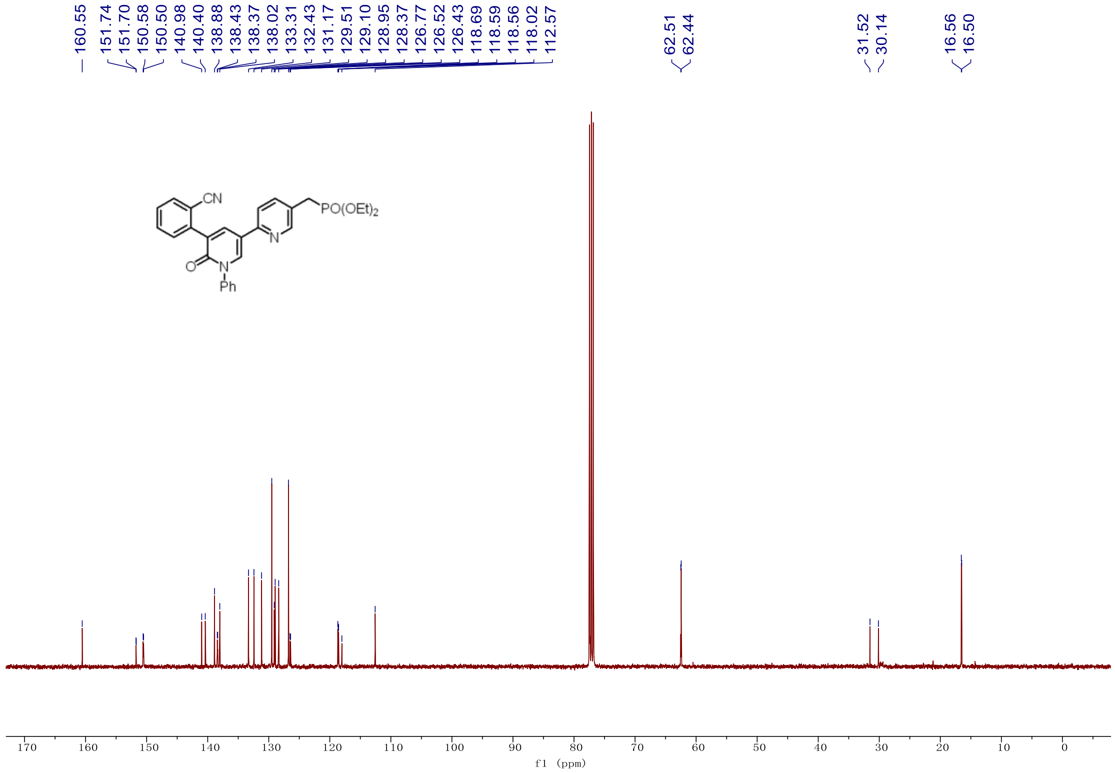
160.55
151.74
151.70
150.58
150.50
140.98
140.40
138.88
138.43
138.37
138.02
133.31
132.43
131.17
129.51
129.10
128.95
128.37
126.77
126.52
126.43
118.69
118.59
118.56
118.02
112.57
62.51
62.44
31.52
30.14
16.56
16.50
CN
PO(OEt)2
N
O
N
Ph
f1 (ppm)

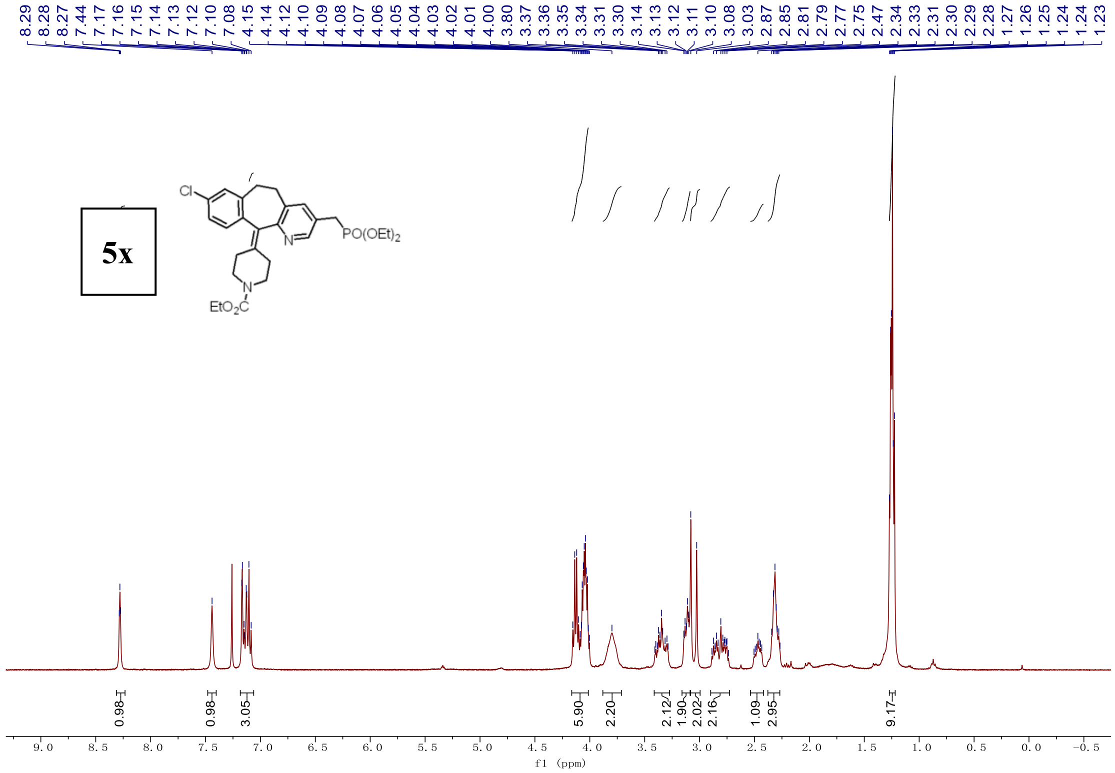
8.29 8.28 8.27 7.44 7.17 7.16 7.15 7.14 7.13 7.12 7.10 7.08 4.15 4.14 4.12 4.10 4.09 4.08 4.07 4.06 4.05 4.04 4.03 4.02 4.01 4.00 3.80 3.37 3.36 3.35 3.34 3.31 3.30 3.14 3.13 3.12 3.11 3.10 3.08 3.03 2.87 2.85 2.81 2.79 2.77 2.75 2.47 2.34 2.33 2.31 2.30 2.29 2.28 1.27 1.26 1.25 1.24 1.24 1.23
5x
Cl
N
PO(OEt)2
N
EtO2C
0.98
0.98
3.05
5.90
2.20
2.12
1.90
2.02
2.16
1.09
2.95
9.17
9.0 8.5 8.0 7.5 7.0 6.5 6.0 5.5 5.0 4.5 4.0 3.5 3.0 2.5 2.0 1.5 1.0 0.5 0.0 -0.5
f1 (ppm)

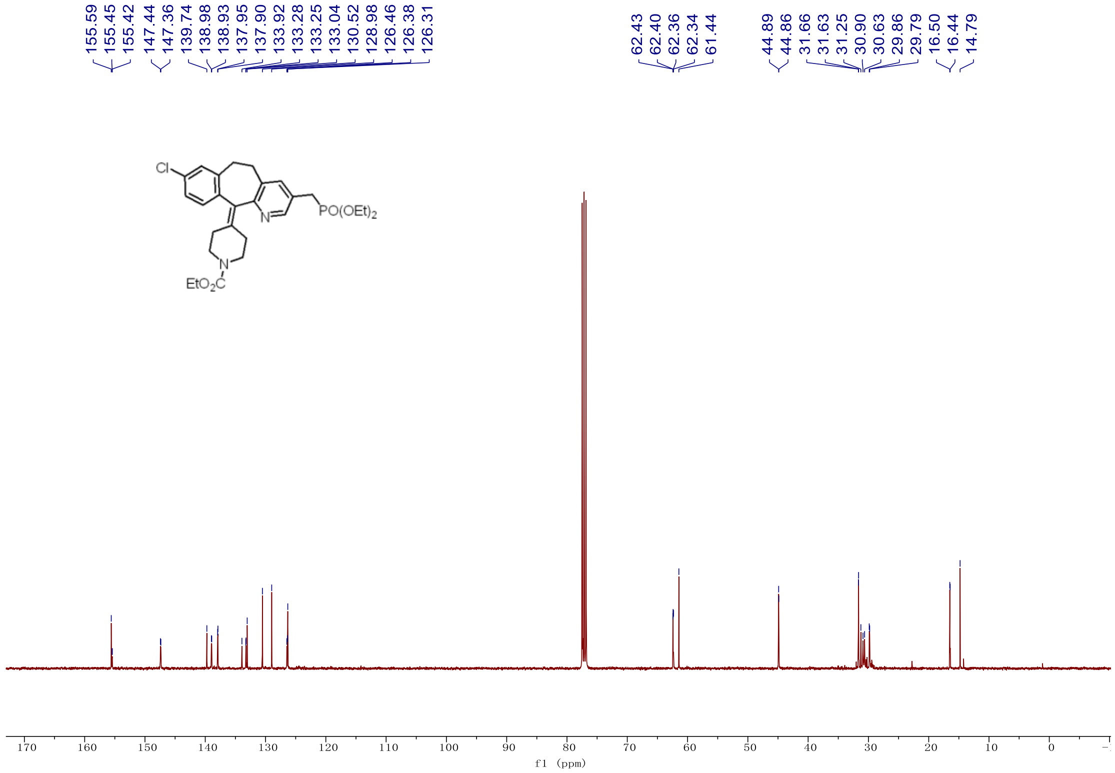
155.59 155.45 155.42 147.44 147.36 139.74 138.98 138.93 137.95 137.90 133.92 133.28 133.25 133.04 130.52 128.98 126.46 126.38 126.31
62.43 62.40 62.36 62.34 61.44
44.89 44.86 31.66 31.63 31.25 30.90 30.63 29.86 29.79 16.50 16.44 14.79
Cl
N
PO(OEt)2
N
EtO2C
170 160 150 140 130 120 110 100 90 80 70 60 50 40 30 20 10 0
f1 (ppm)

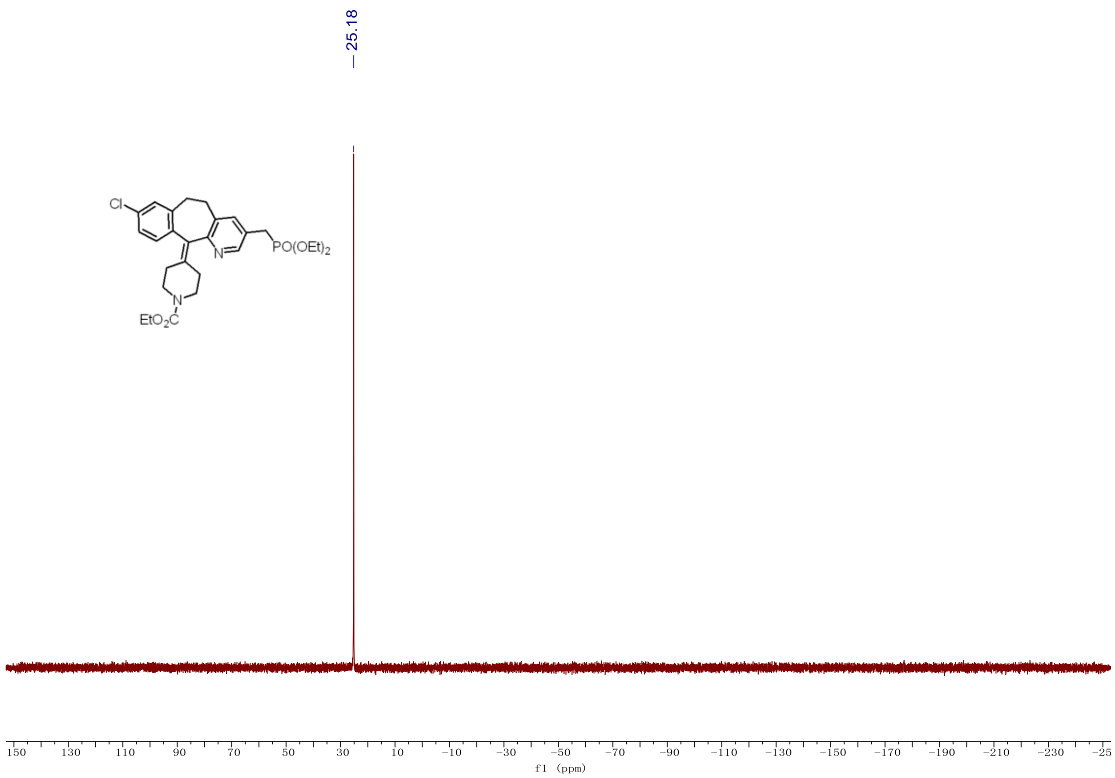

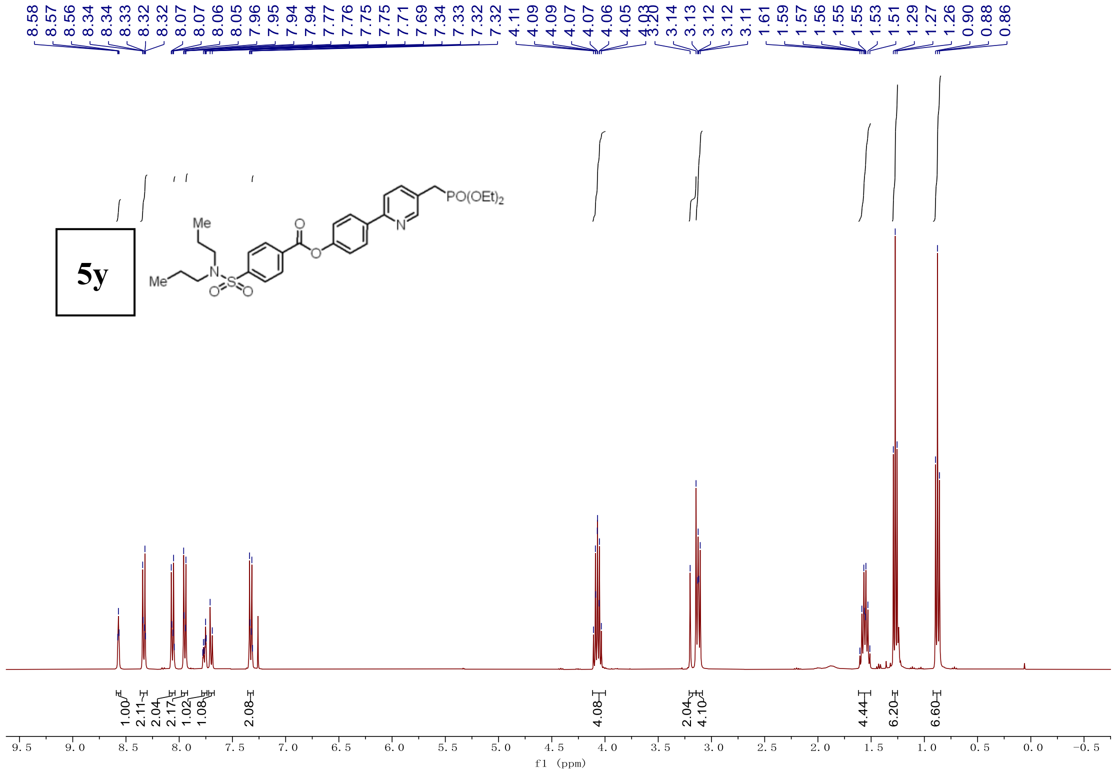

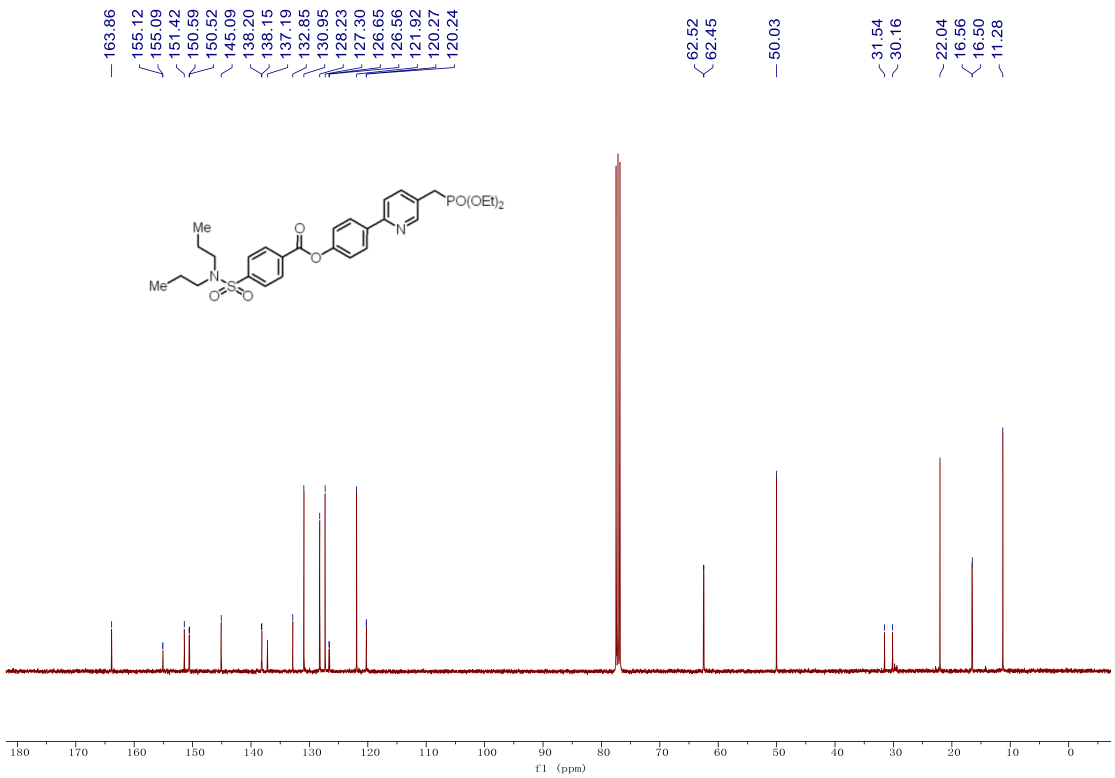
163.86
155.12
155.09
151.42
150.59
150.52
145.09
138.20
138.15
137.19
132.85
130.95
128.23
127.30
126.65
126.56
121.92
120.27
120.24
62.52
62.45
50.03
31.54
30.16
22.04
16.56
16.50
11.28
PO(OEt)2
f1 (ppm)

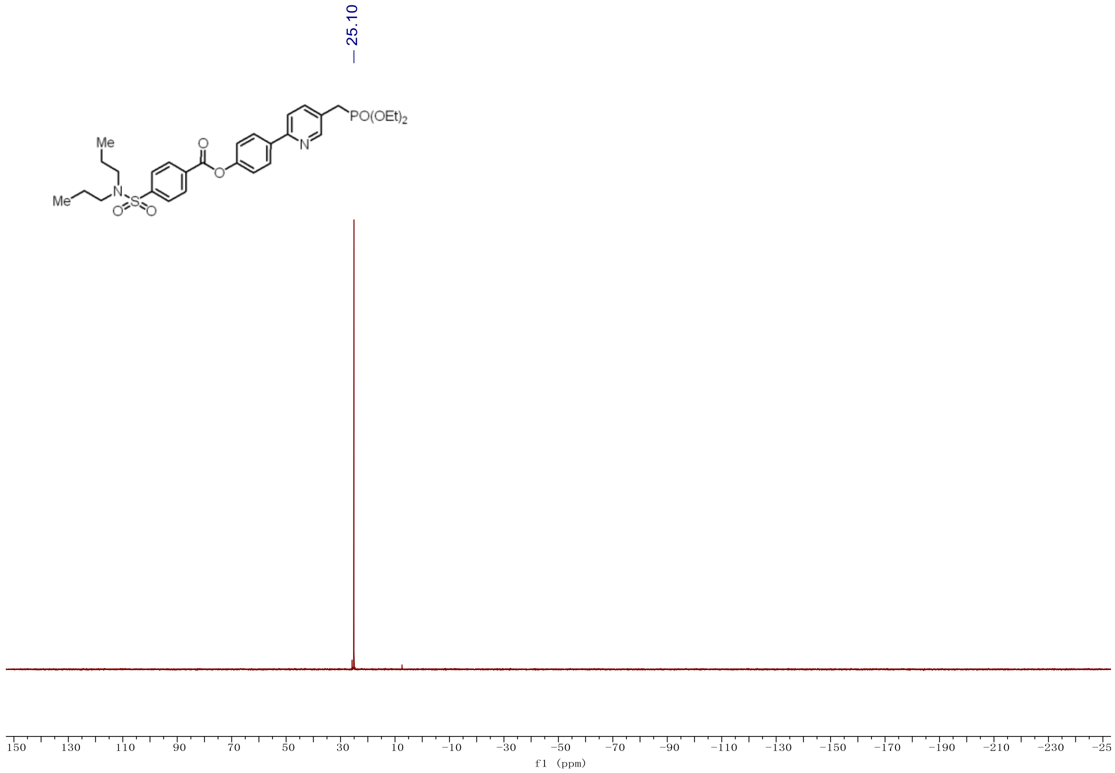
25.10
PO(OEt)2
f1 (ppm)

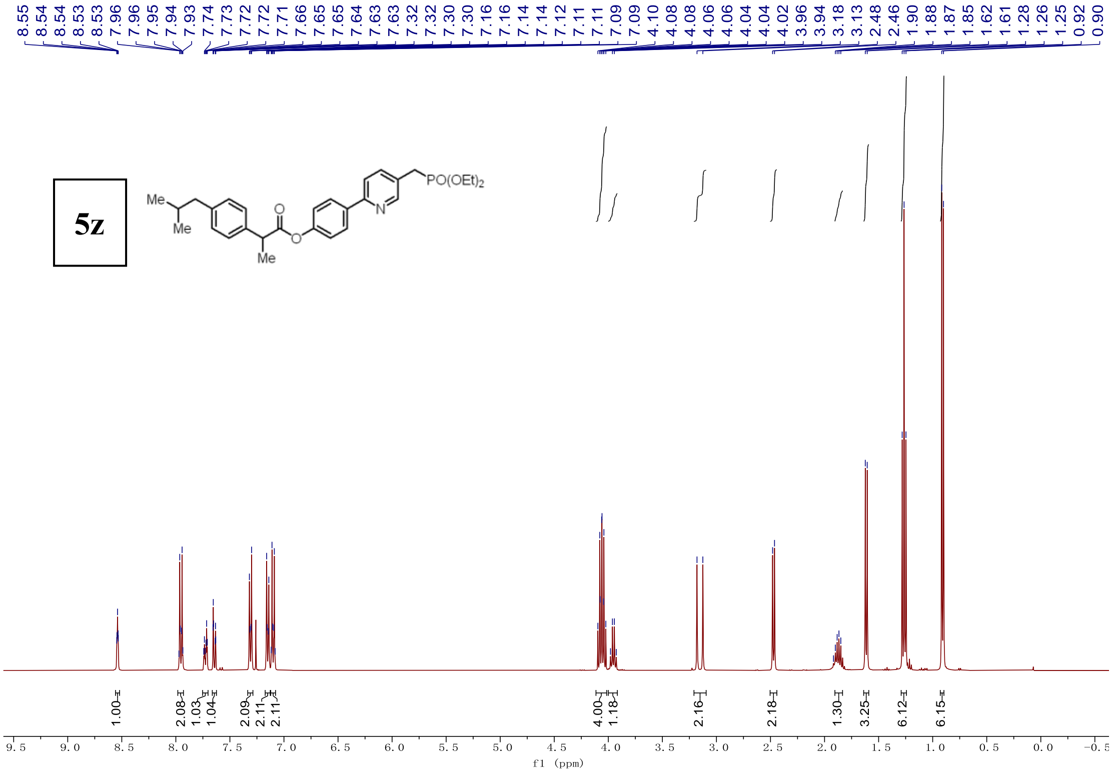
8.55 8.54 8.53 8.53 7.96 7.96 7.95 7.94 7.93 7.74 7.73 7.72 7.72 7.71 7.66 7.65 7.64 7.63 7.63 7.32 7.32 7.30 7.16 7.16 7.14 7.12 7.11 7.11 7.09 4.10 4.08 4.06 4.06 4.04 4.04 4.02 3.96 3.94 3.18 3.13 2.48 2.46 1.90 1.88 1.87 1.85 1.62 1.61 1.28 1.26 1.25 0.92 0.90
5z
Me
Me
O
O
Me
N
PO(OEt)2
1.00
2.08
1.03
1.04
2.09
2.11
2.11
4.00
1.18
2.16
2.18
1.30
3.25
6.12
6.15
f1 (ppm)

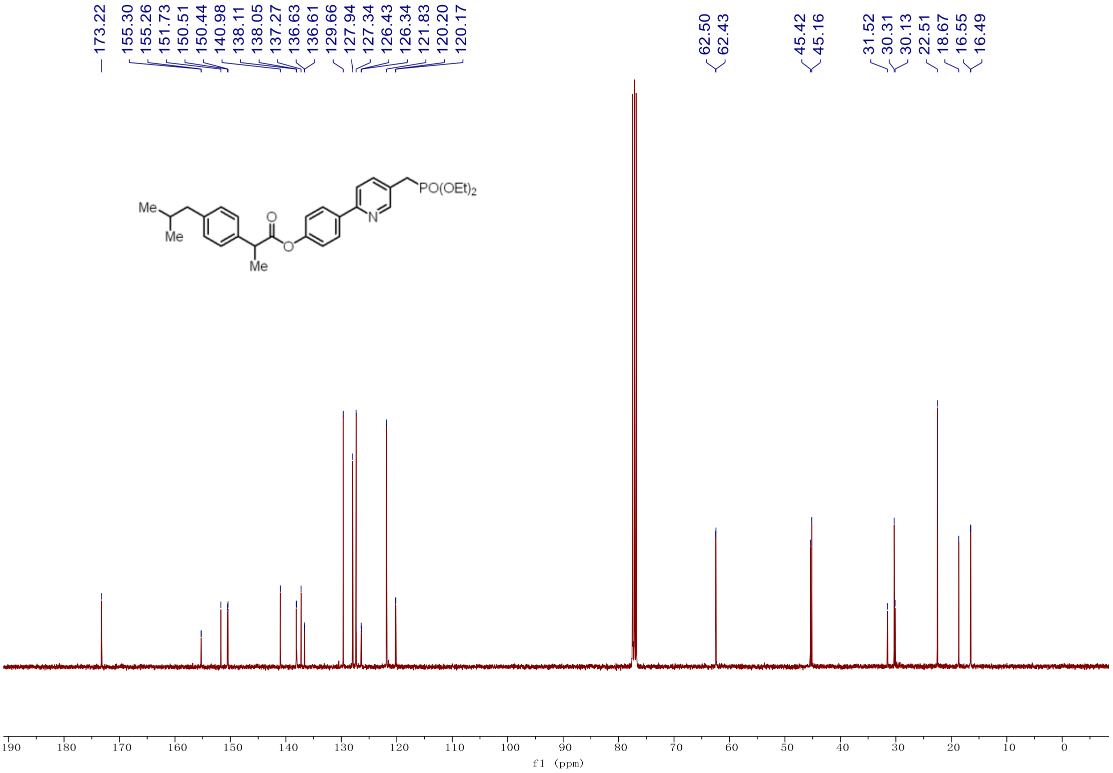
173.22 155.30 155.26 151.73 150.51 150.44 140.98 138.11 138.05 137.27 136.63 136.61 129.66 127.94 127.34 126.43 126.34 121.83 120.20 120.17
62.50 62.43
45.42 45.16
31.52 30.31 30.13 22.51 18.67 16.55 16.49
Me
Me
O
O
Me
N
PO(OEt)2
f1 (ppm)

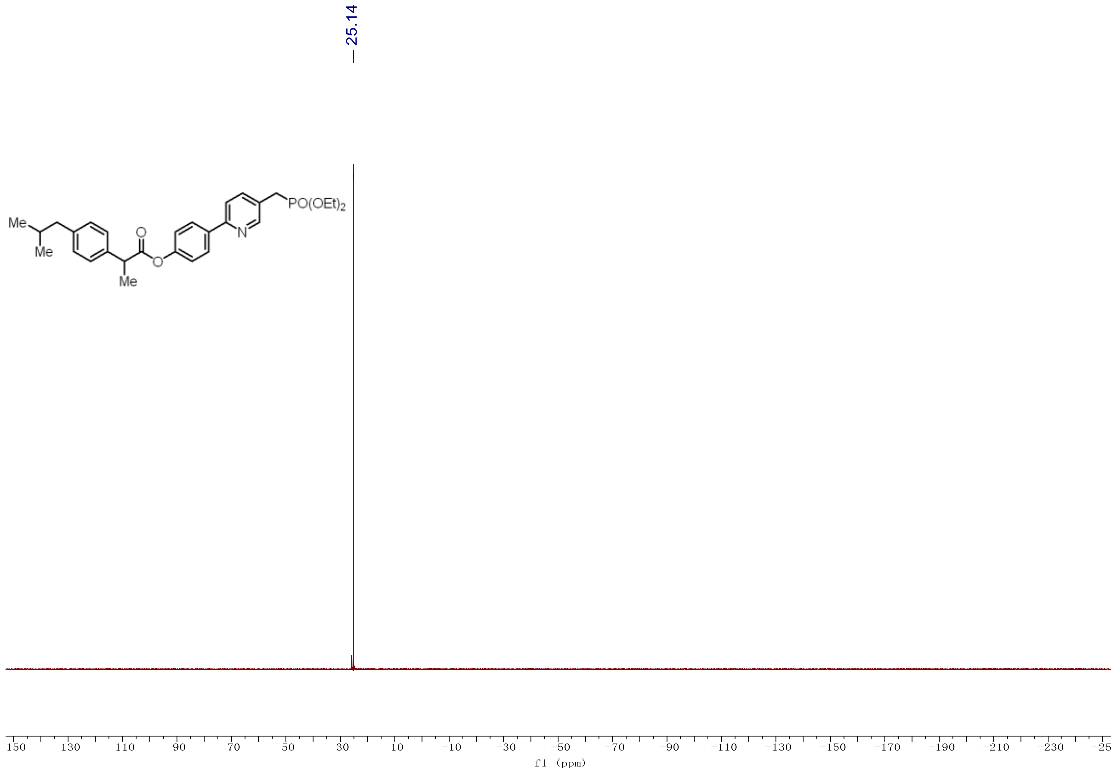
25.14
PO(OEt)2
Me
Me
O
O
Me
N
f1 (ppm)

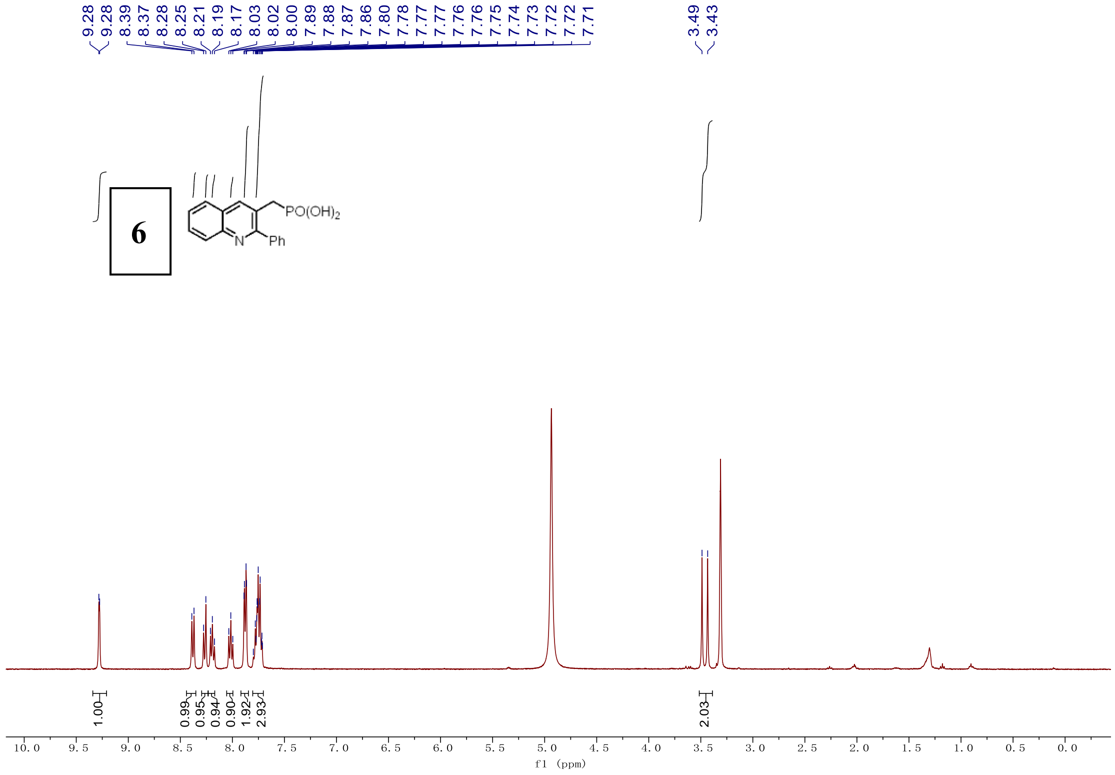
9.28
9.28
8.39
8.37
8.28
8.25
8.21
8.19
8.17
8.03
8.02
8.00
7.89
7.88
7.87
7.86
7.80
7.78
7.77
7.77
7.76
7.76
7.75
7.74
7.73
7.72
7.72
7.71
3.49
3.43
6
PO(OH)2
N
Ph
1.00
0.99
0.95
0.94
0.90
1.92
2.93
2.03
f1 (ppm)

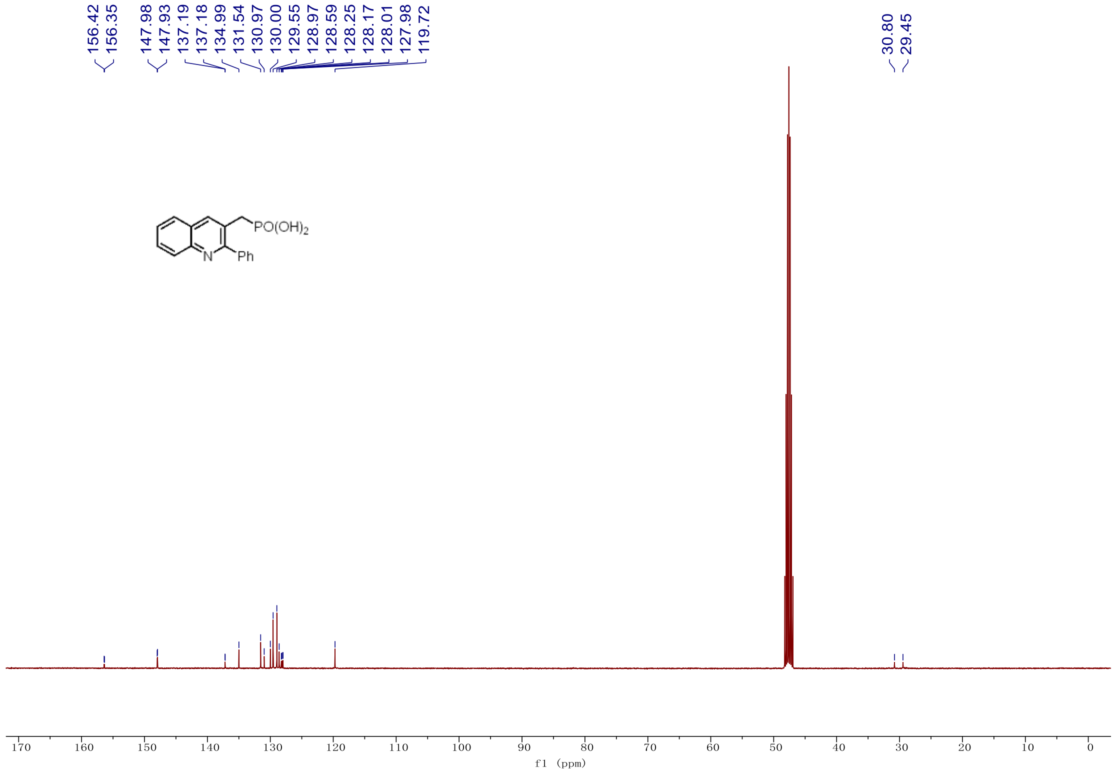
156.42
156.35
147.98
147.93
137.19
137.18
134.99
131.54
130.97
130.00
129.55
128.97
128.59
128.25
128.17
128.01
127.98
119.72
30.80
29.45
PO(OH)2
N
Ph
f1 (ppm)

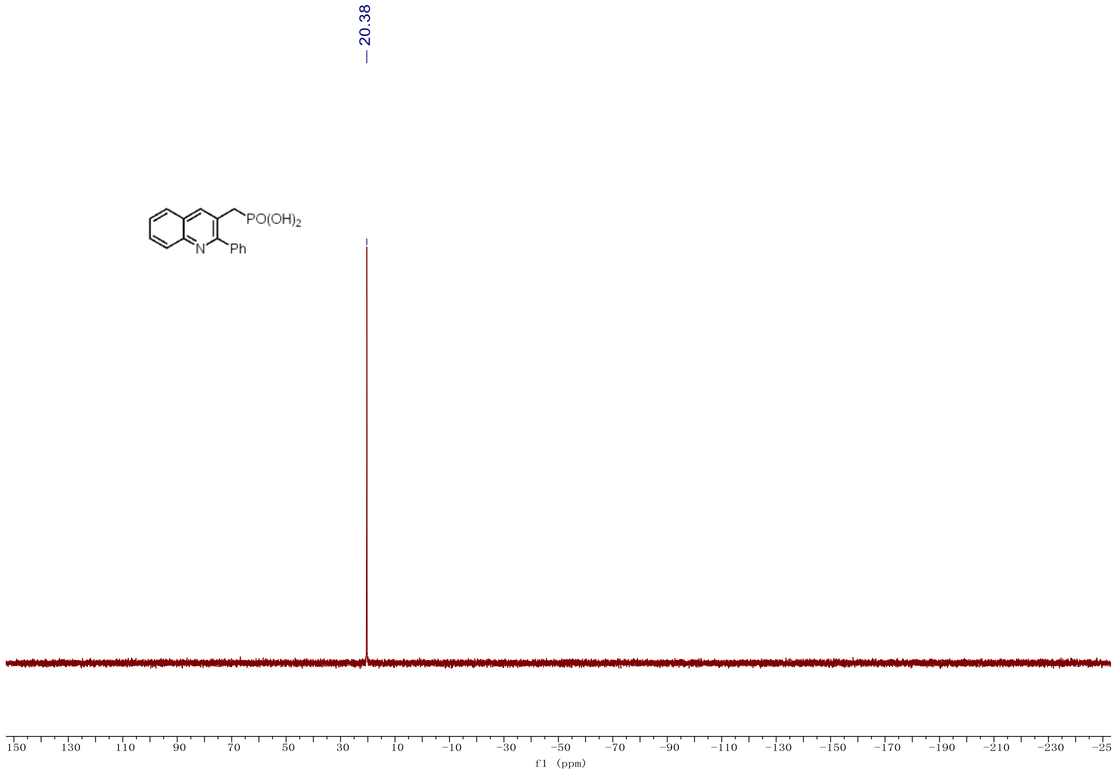
20.38
PO(OH)2
N
Ph
f1 (ppm)

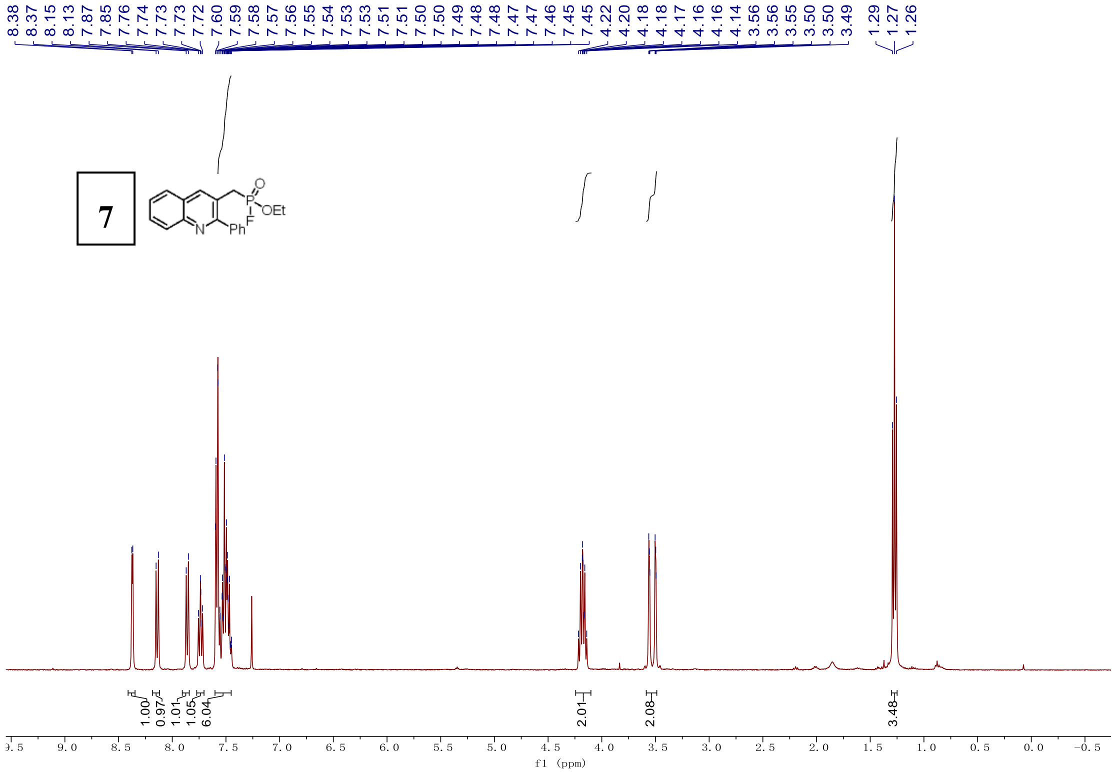
8.38 8.37 8.15 8.13 7.87 7.85 7.76 7.74 7.73 7.73 7.72 7.60 7.59 7.58 7.57 7.56 7.55 7.54 7.53 7.53 7.51 7.51 7.50 7.50 7.49 7.48 7.48 7.47 7.47 7.46 7.45 7.45 4.22 4.20 4.18 4.18 4.17 4.16 4.16 4.14 3.56 3.56 3.55 3.50 3.50 3.49 1.29 1.27 1.26
7
OEt
F
N
Ph
O
P
1.00 0.97 1.01 1.05 6.04
2.01
2.08
3.48
f1 (ppm)

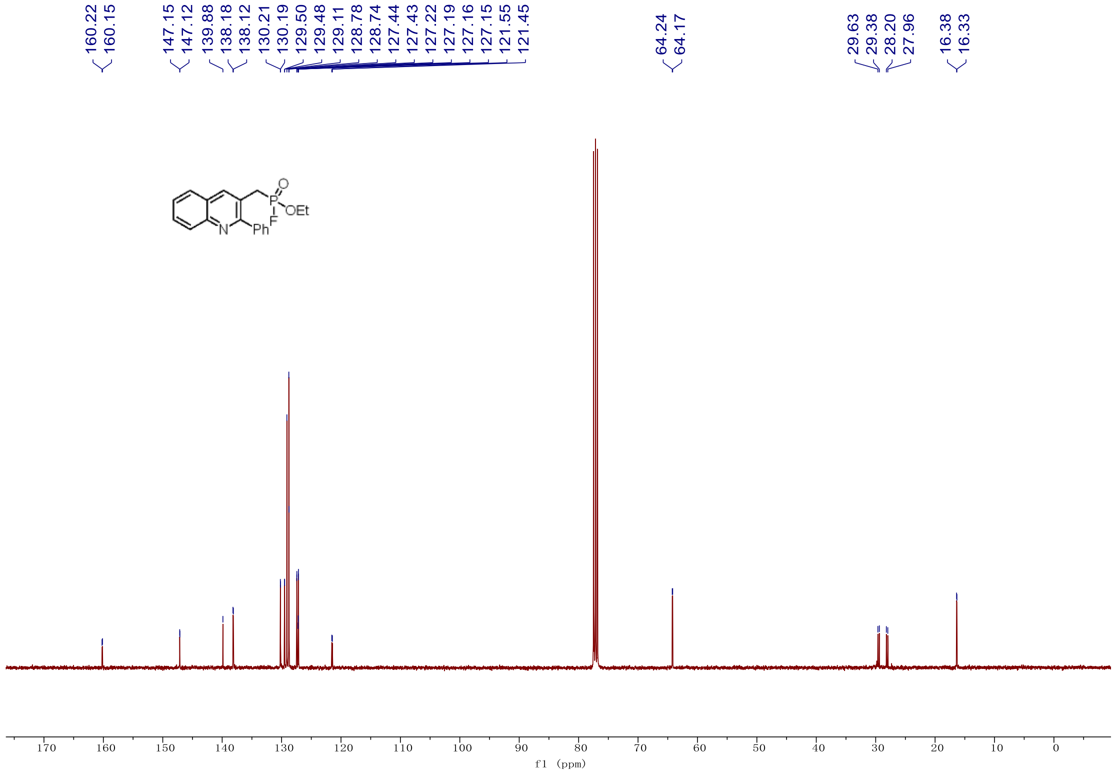
160.22 160.15 147.15 147.12 139.88 138.18 138.12 130.21 130.19 129.50 129.48 129.11 128.78 128.74 127.44 127.43 127.22 127.19 127.16 127.15 121.55 121.45 64.24 64.17 29.63 29.38 28.20 27.96 16.38 16.33
OEt
F
N
Ph
O
P
f1 (ppm)

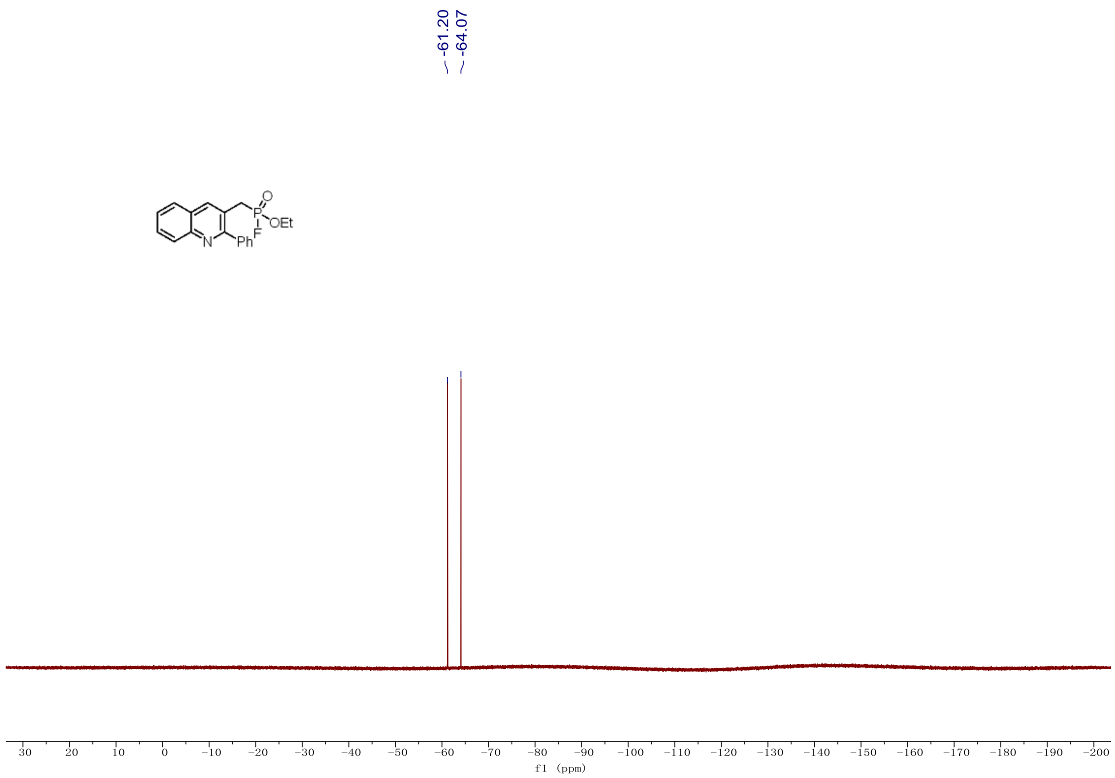
-61.20
-64.07
OEt
F
N
Ph
30 20 10 0 −10 −20 −30 −40 −50 −60 −70 −80 −90 −100 −110 −120 −130 −140 −150 −160 −170 −180 −190 −200
f1 (ppm)

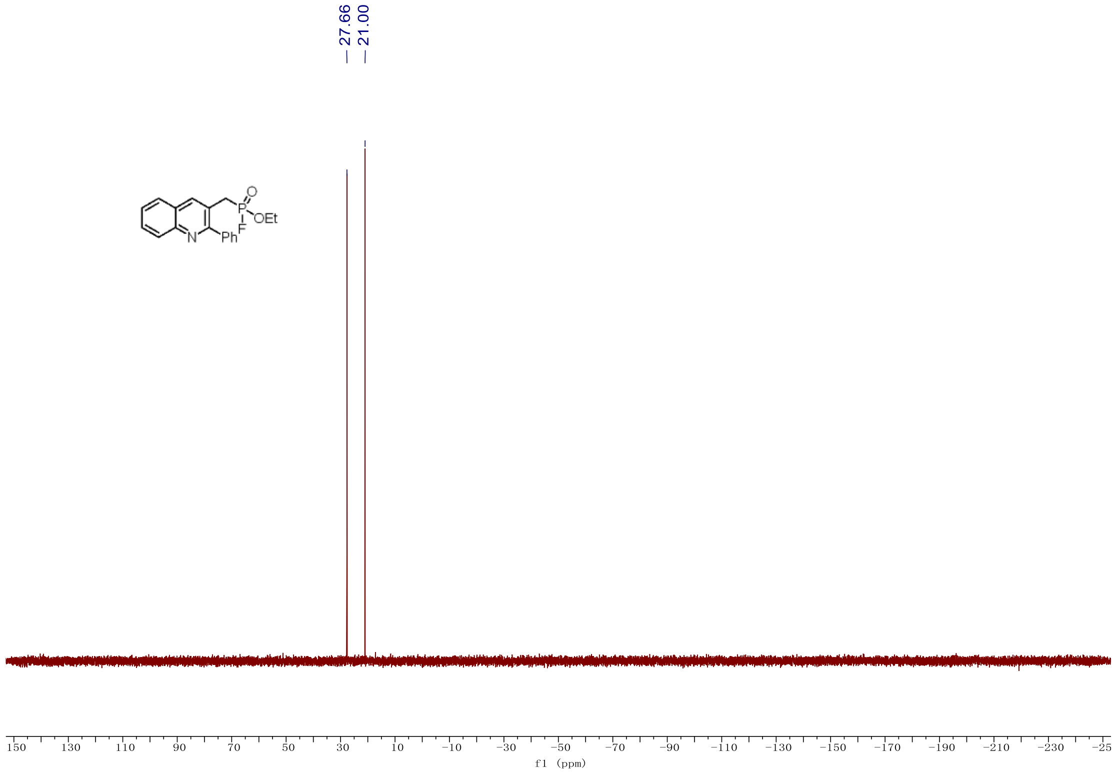
27.66
21.00
OEt
F
N
Ph
150 130 110 90 70 50 30 10 −10 −30 −50 −70 −90 −110 −130 −150 −170 −190 −210 −230
f1 (ppm)

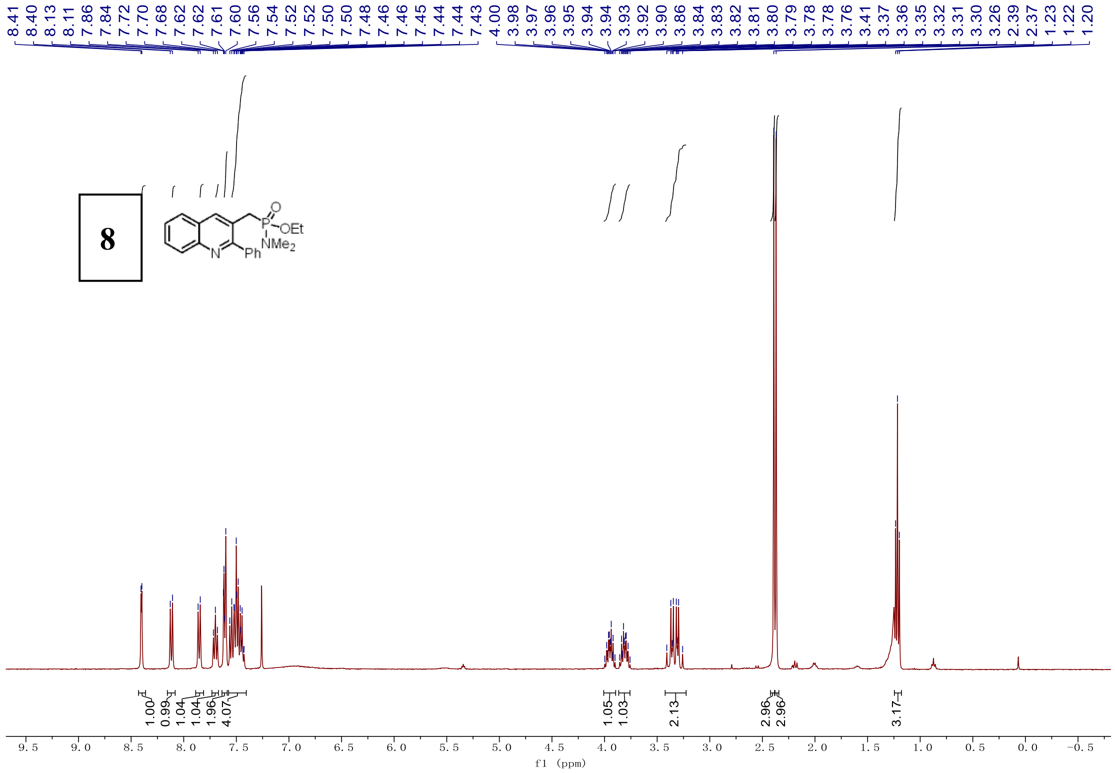
8.41 8.40 8.13 8.11 7.86 7.84 7.72 7.70 7.68 7.62 7.62 7.61 7.60 7.56 7.54 7.52 7.52 7.50 7.50 7.48 7.46 7.46 7.45 7.44 7.44 7.43 4.00 3.98 3.97 3.96 3.95 3.94 3.94 3.93 3.92 3.90 3.86 3.84 3.83 3.82 3.81 3.80 3.79 3.78 3.78 3.76 3.41 3.37 3.36 3.35 3.32 3.31 3.30 3.26 2.39 2.37 1.23 1.22 1.20
8
OEt
NMe2
N
Ph
O
P
1.00 0.99 1.04 1.04 1.96 4.07 1.05 1.03 2.13 2.96 2.96 3.17
f1 (ppm)

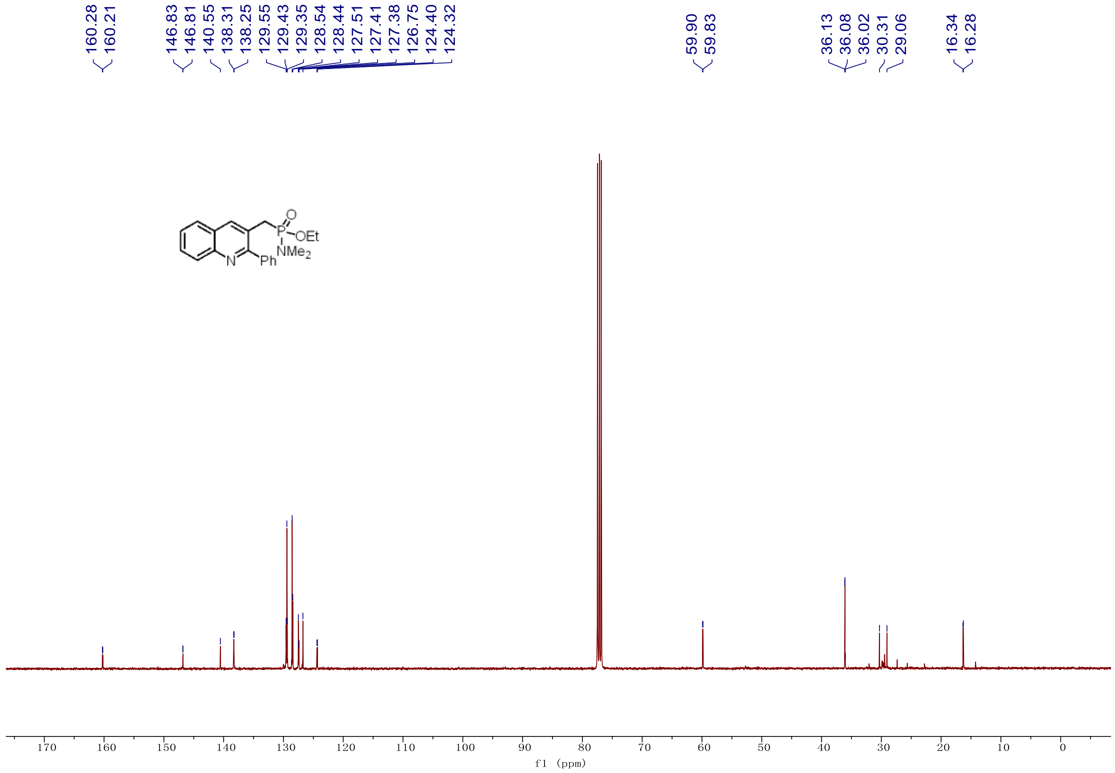
160.28 160.21 146.83 146.81 140.55 138.31 138.25 129.55 129.43 129.35 128.54 128.44 127.51 127.41 127.38 126.75 124.40 124.32 59.90 59.83 36.13 36.08 36.02 30.31 29.06 16.34 16.28
OEt
NMe2
N
Ph
O
P
f1 (ppm)

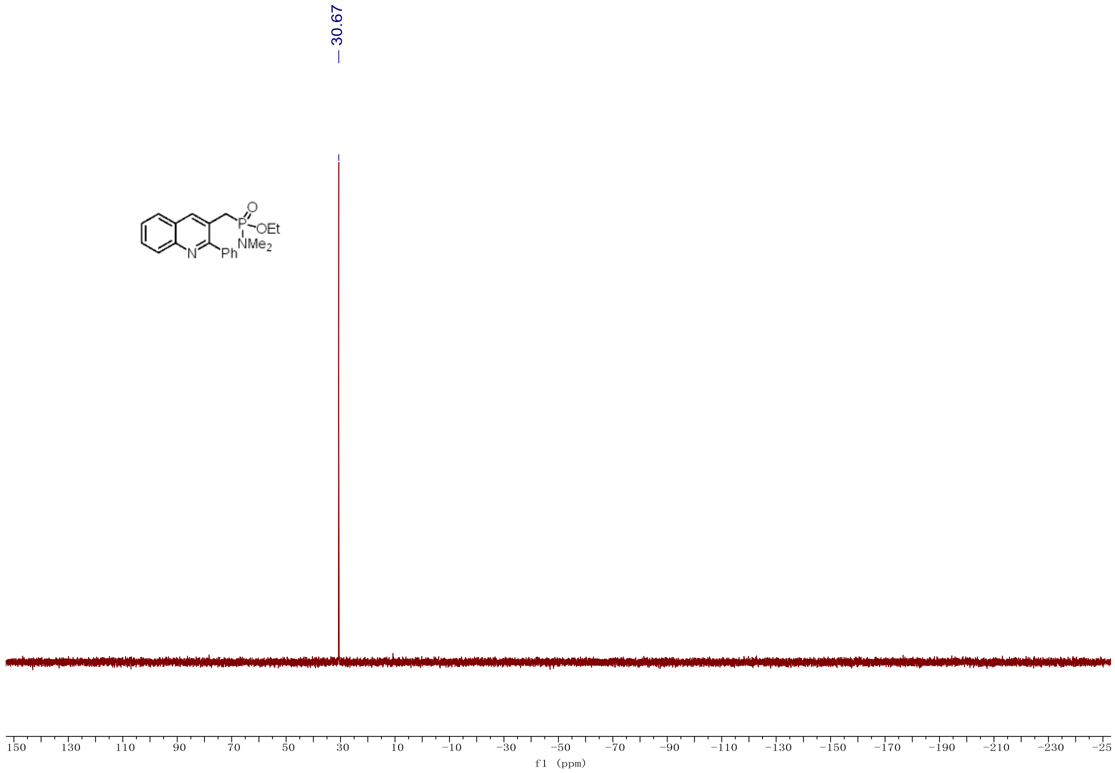

9

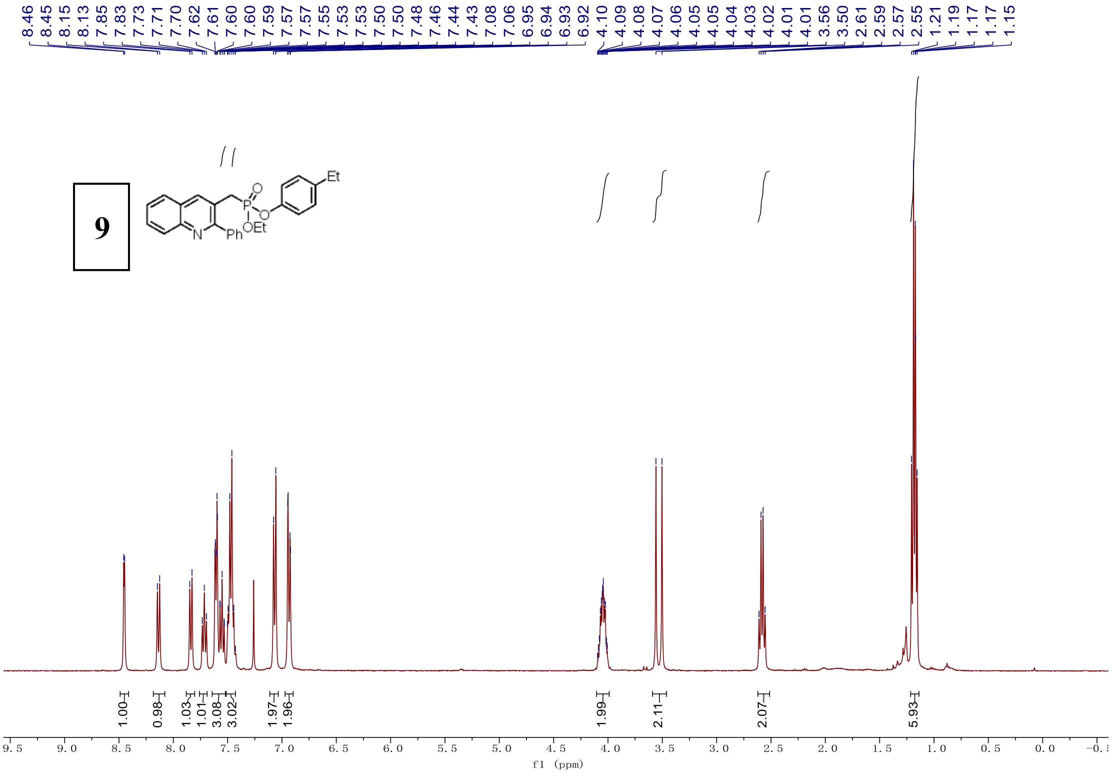

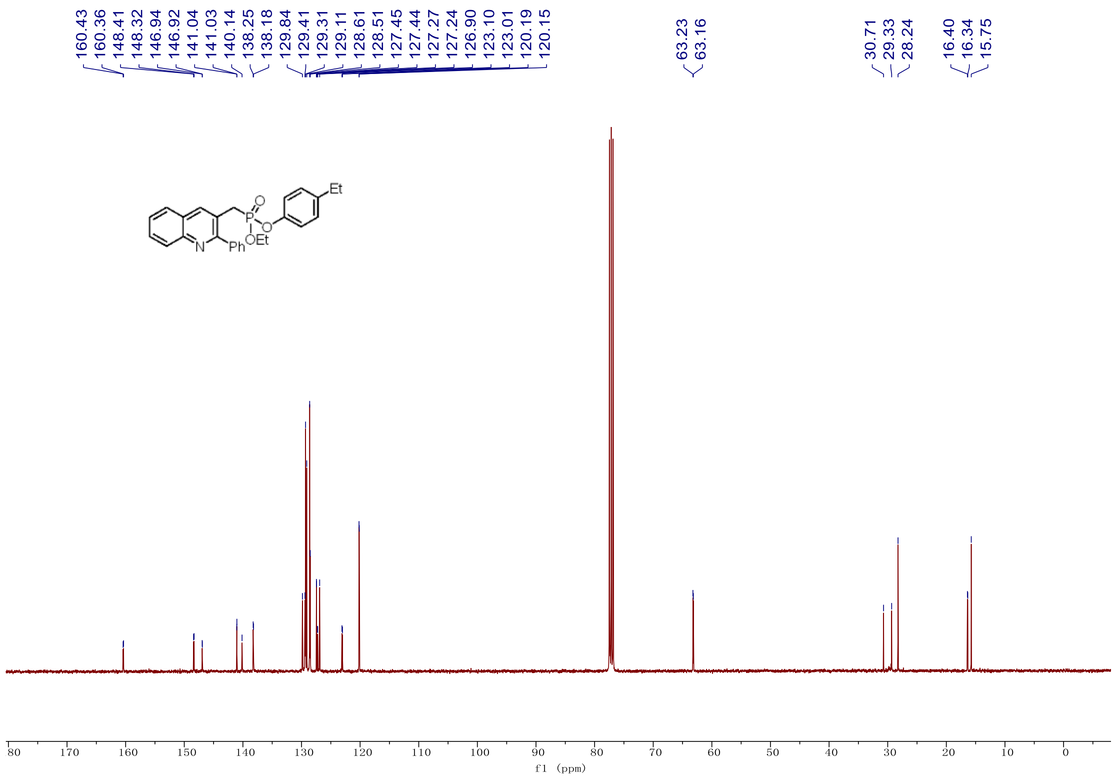
160.43
160.36
148.41
148.32
146.94
146.92
141.04
141.03
140.14
138.25
138.18
129.84
129.41
129.31
129.11
128.61
128.51
127.45
127.44
127.27
127.24
126.90
123.10
123.01
120.19
120.15
63.23
63.16
30.71
29.33
28.24
16.40
16.34
15.75
Et
O
P
O
N
Ph
OEt
f1 (ppm)

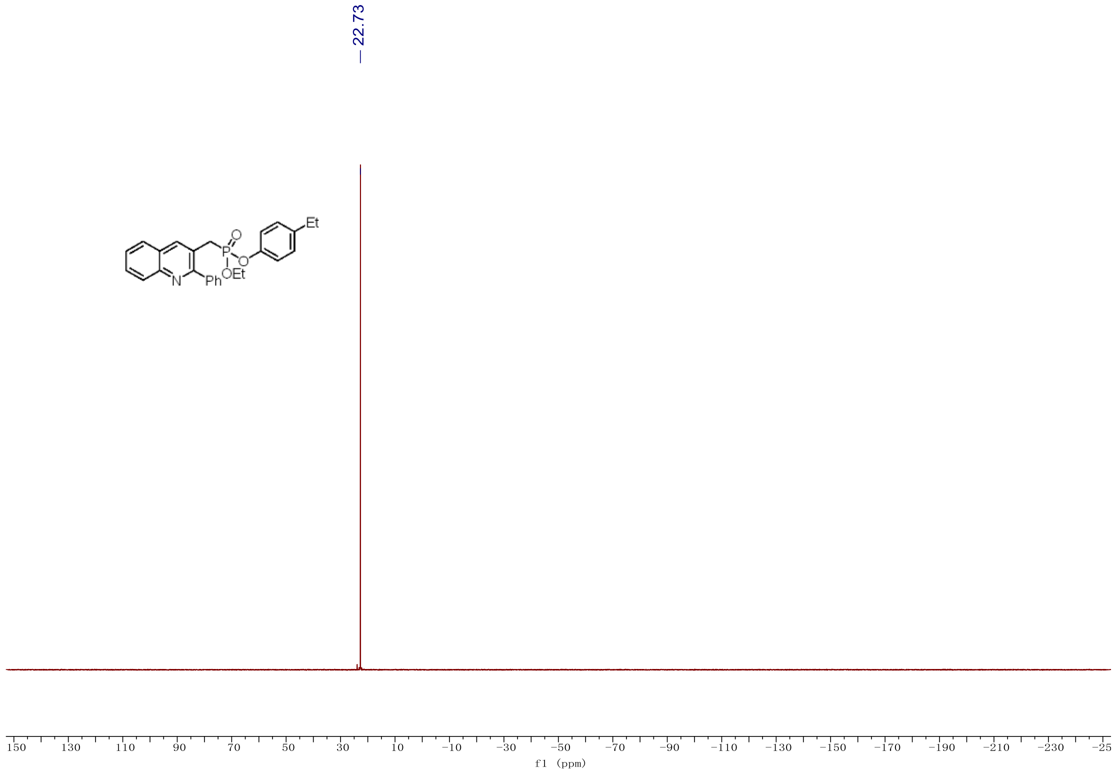
22.73
Et
O
P
O
N
Ph
OEt
f1 (ppm)

10

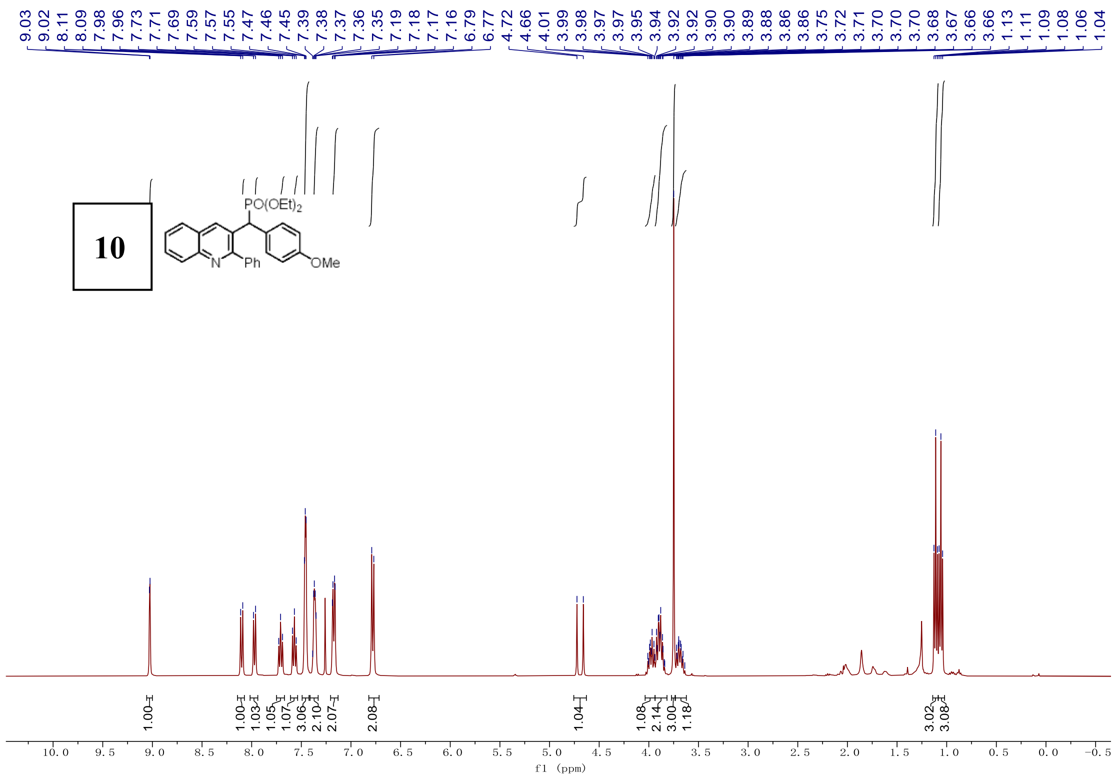
9.03 9.02 8.11 8.09 7.98 7.96 7.73 7.71 7.69 7.59 7.57 7.55 7.47 7.46 7.45 7.39 7.38 7.37 7.36 7.35 7.19 7.18 7.17 7.16 6.79 6.77 4.72 4.66 4.01 3.99 3.98 3.97 3.97 3.95 3.94 3.92 3.92 3.90 3.90 3.89 3.88 3.86 3.75 3.72 3.71 3.70 3.70 3.68 3.67 3.66 1.13 1.11 1.09 1.08 1.06 1.04
PO(OEt)2
N
Ph
OMe
1.00 1.00 1.03 1.05 1.07 3.06 2.10 2.07 2.08 1.04 1.08 2.14 3.00 1.18 3.02 3.08
10.0 9.5 9.0 8.5 8.0 7.5 7.0 6.5 6.0 5.5 5.0 4.5 4.0 3.5 3.0 2.5 2.0 1.5 1.0 0.5 0.0 -0.5
f1 (ppm)

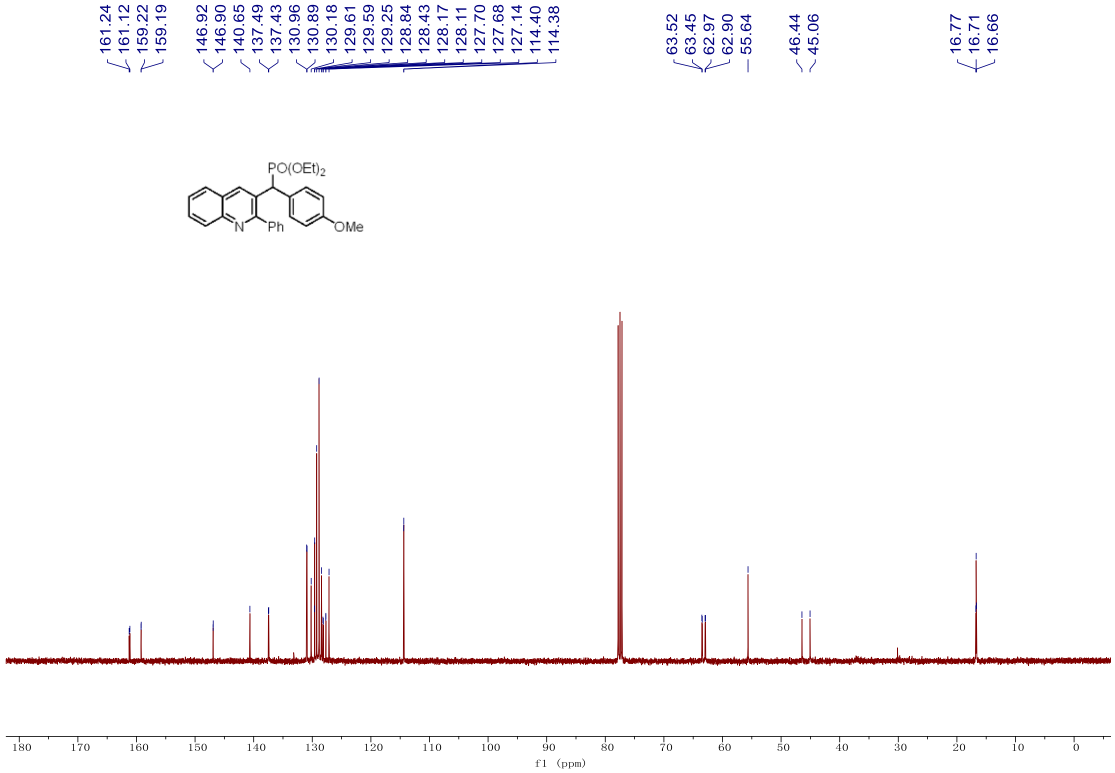
161.24 161.12 159.22 159.19 146.92 146.90 140.65 137.49 137.43 130.96 130.89 130.18 129.61 129.59 129.25 128.84 128.43 128.17 128.11 127.70 127.68 127.14 114.40 114.38 63.52 63.45 62.97 62.90 55.64 46.44 45.06 16.77 16.71 16.66
PO(OEt)2
N
Ph
OMe
180 170 160 150 140 130 120 110 100 90 80 70 60 50 40 30 20 10 0
f1 (ppm)

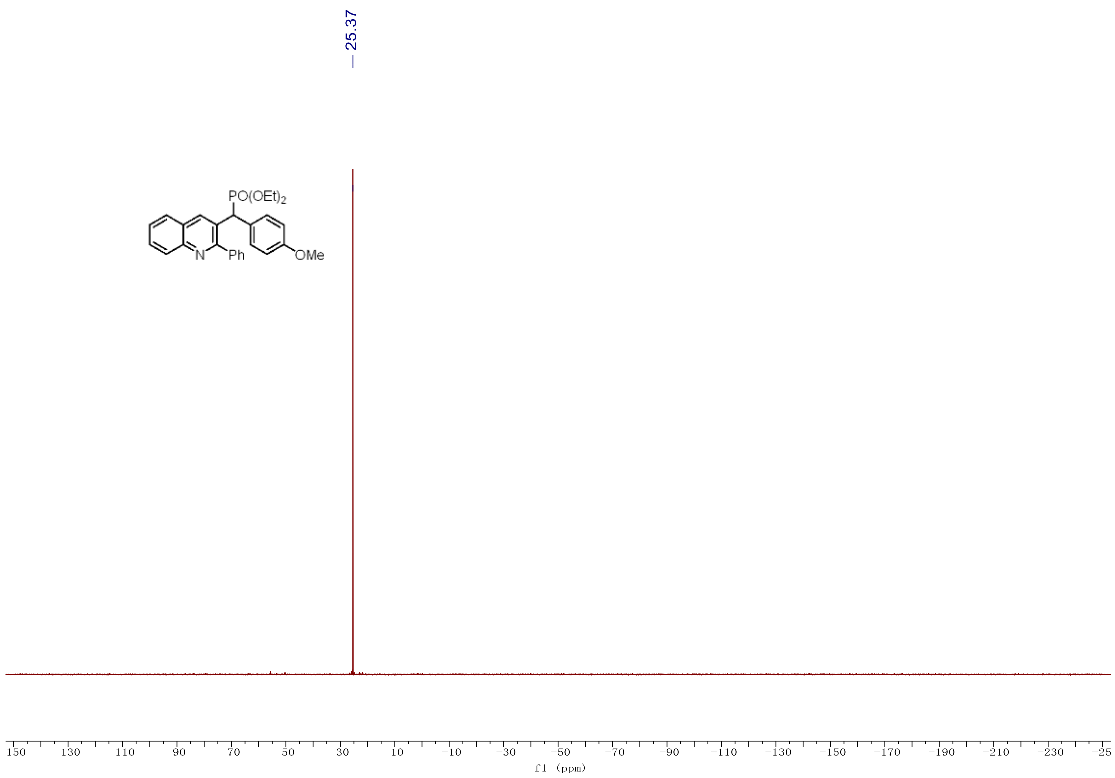

11

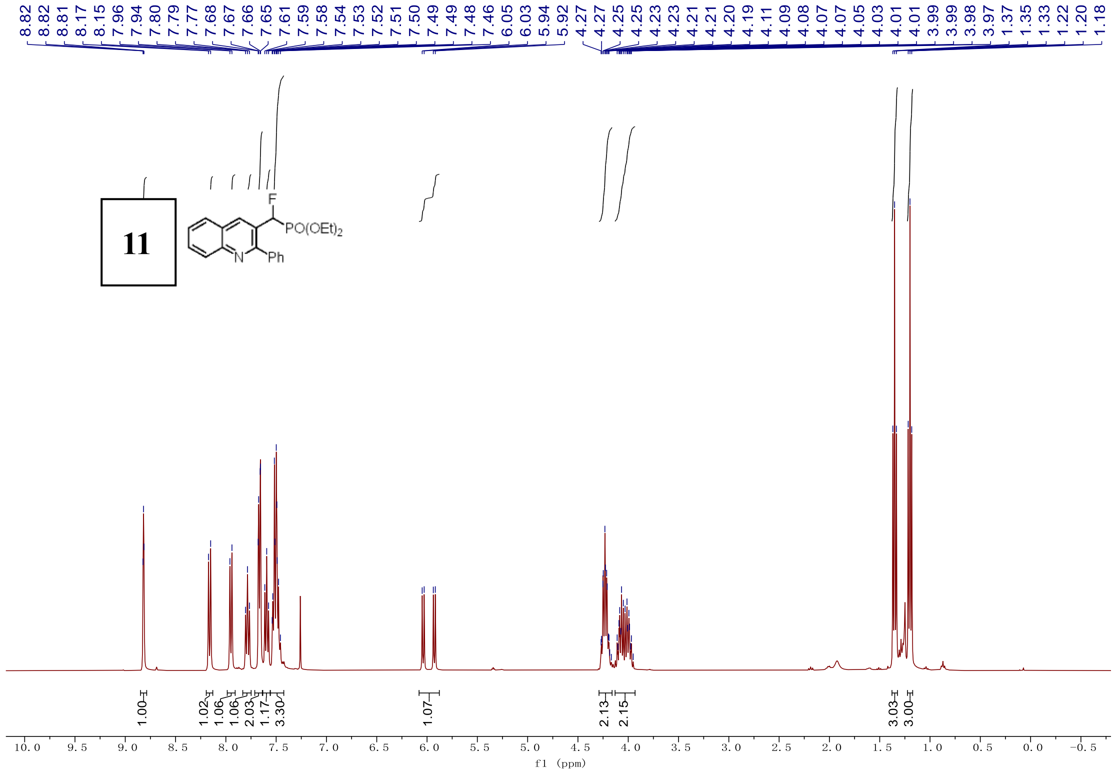

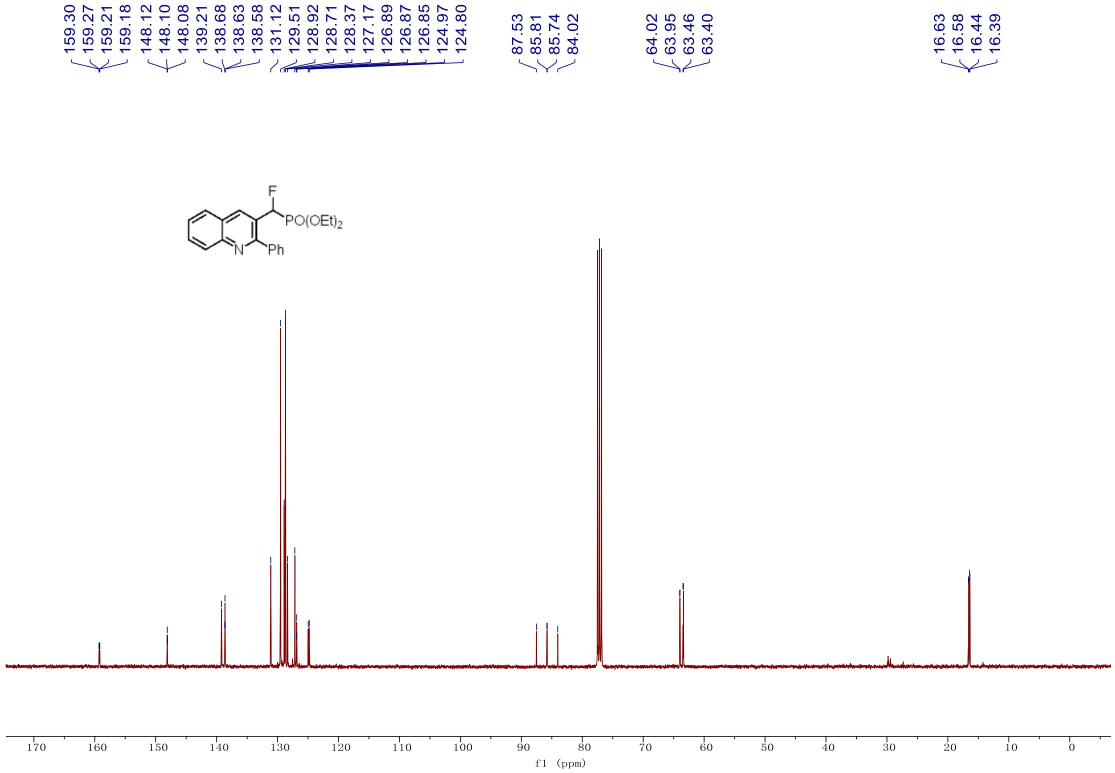
159.30
159.27
159.21
159.18
148.12
148.10
148.08
139.21
138.68
138.63
138.58
131.12
129.51
128.92
128.71
128.37
127.17
126.89
126.87
126.85
124.97
124.80
87.53
85.81
85.74
84.02
64.02
63.95
63.46
63.40
16.63
16.58
16.44
16.39
F
PO(OEt)2
N
Ph
170 160 150 140 130 120 110 100 90 80 70 60 50 40 30 20 10 0
f1 (ppm)

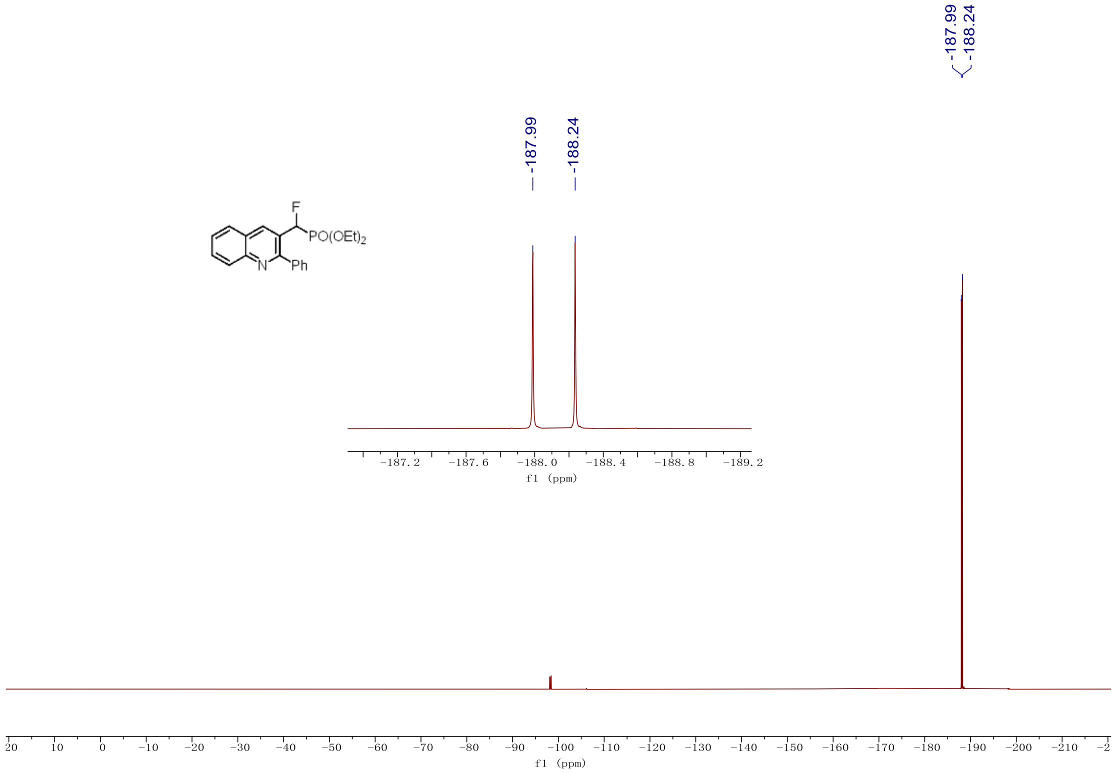
-187.99
-188.24
-187.99
-188.24
F
PO(OEt)2
N
Ph
-187.2 -187.6 -188.0 -188.4 -188.8 -189.2
f1 (ppm)
20 10 0 -10 -20 -30 -40 -50 -60 -70 -80 -90 -100 -110 -120 -130 -140 -150 -160 -170 -180 -190 -200 -210
f1 (ppm)

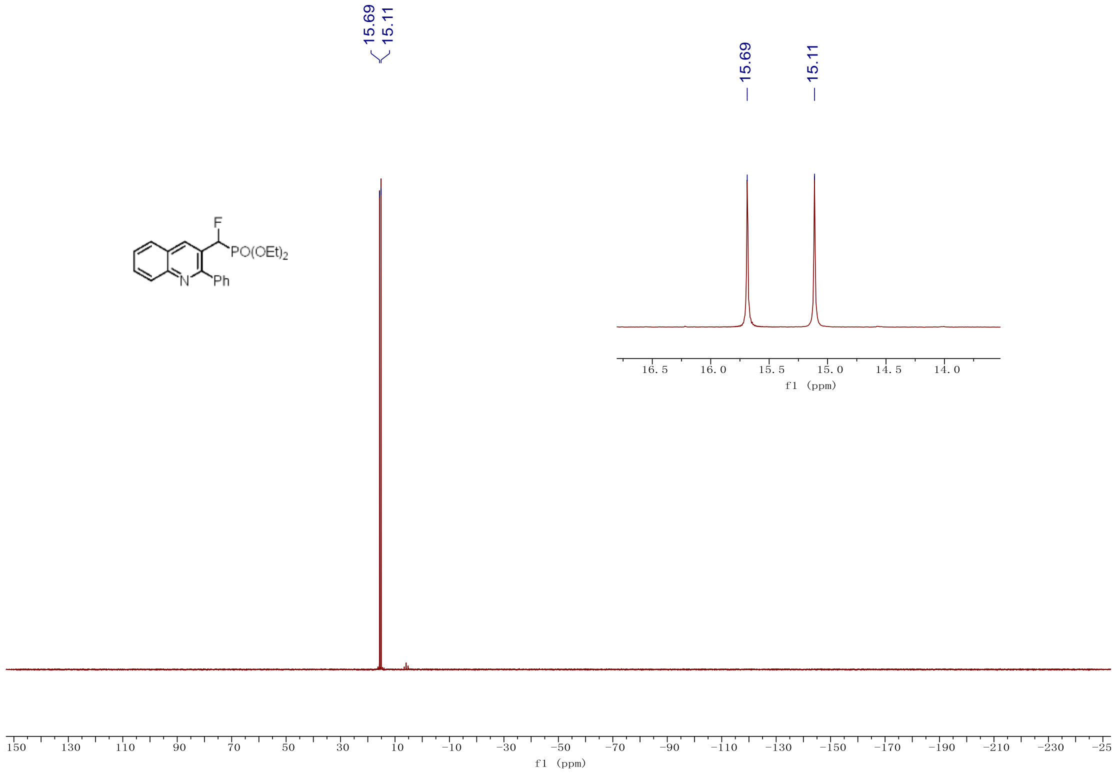
F
PO(OEt)2
N
Ph
15.69
15.11
16.5 16.0 15.5 15.0 14.5 14.0
f1 (ppm)
150 130 110 90 70 50 30 10 −10 −30 −50 −70 −90 −110 −130 −150 −170 −190 −210 −230 −25
f1 (ppm)

12

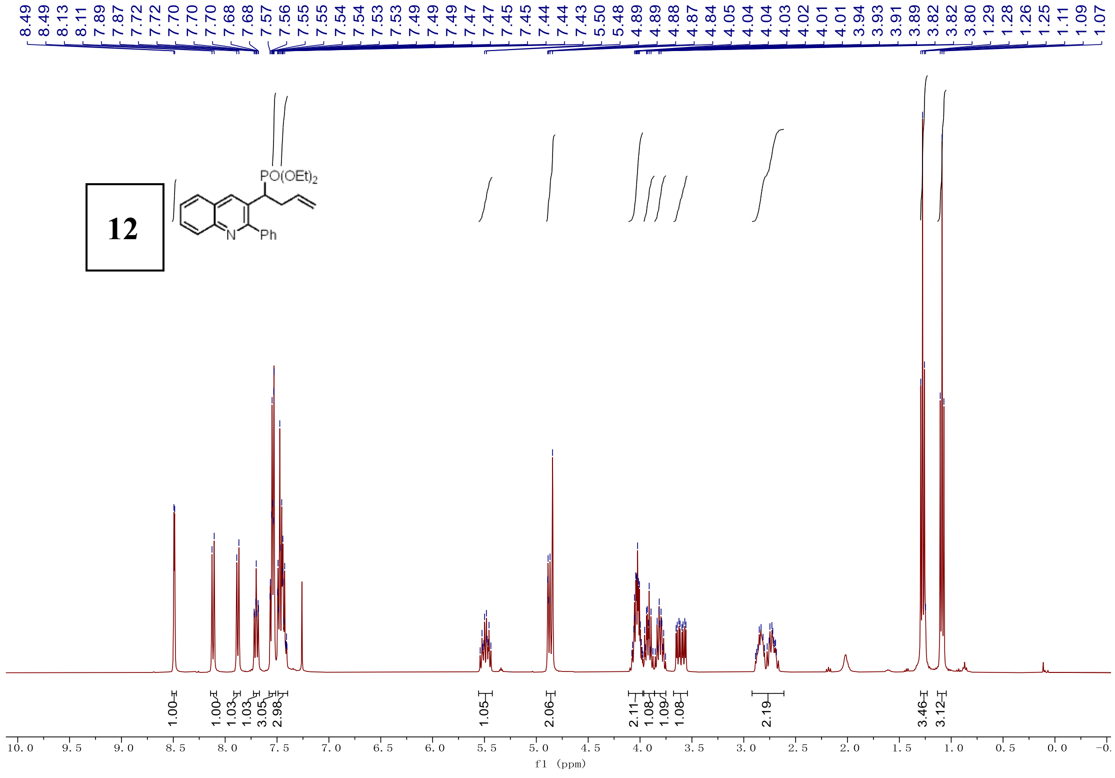
8.49 8.49 8.13 8.11 7.89 7.87 7.72 7.72 7.70 7.70 7.70 7.68 7.68 7.57 7.56 7.55 7.55 7.54 7.53 7.53 7.49 7.49 7.49 7.47 7.47 7.45 7.45 7.44 7.44 7.43 5.50 5.48 4.89 4.89 4.88 4.87 4.84 4.05 4.04 4.04 4.03 4.02 4.01 4.01 3.94 3.93 3.91 3.89 3.82 3.82 3.80 1.29 1.28 1.26 1.25 1.11 1.09 1.07
PO(OEt)2
N
Ph
1.00 1.00 1.03 1.03 3.05 2.98 1.05 2.06 2.11 1.08 1.09 1.08 2.19 3.46 3.12
10.0 9.5 9.0 8.5 8.0 7.5 7.0 6.5 6.0 5.5 5.0 4.5 4.0 3.5 3.0 2.5 2.0 1.5 1.0 0.5 0.0 −0.
f1 (ppm)

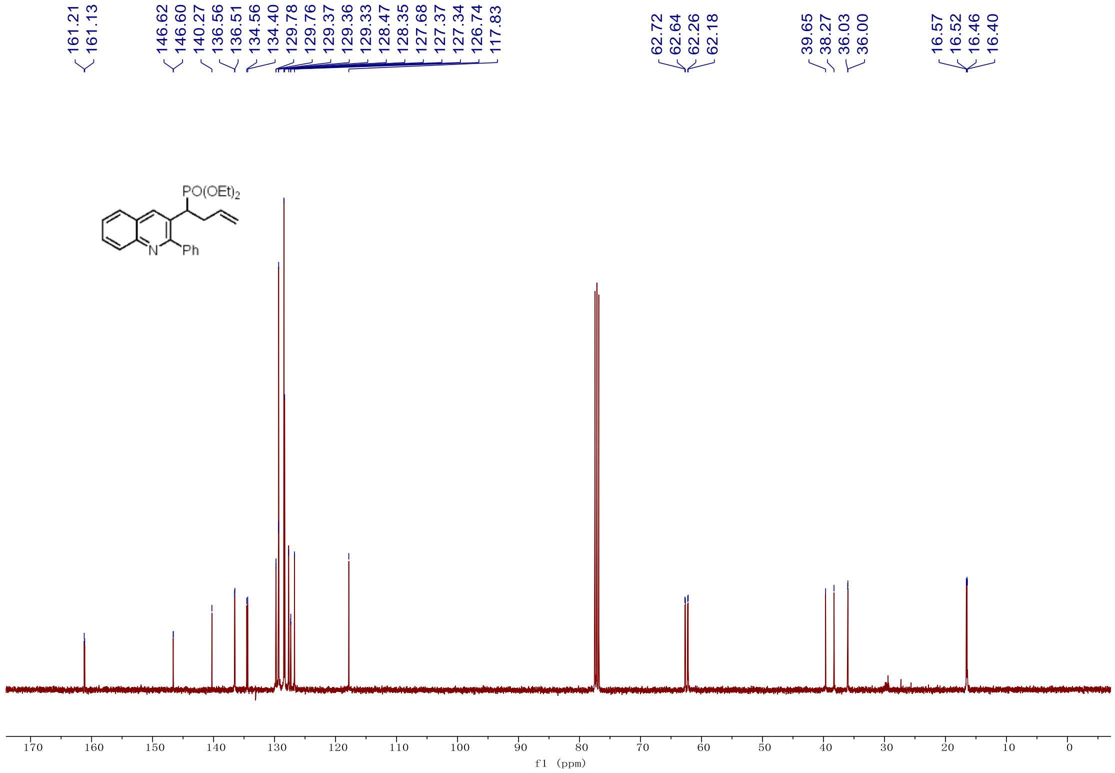
161.21
161.13
146.62
146.60
140.27
136.56
136.51
134.56
134.40
129.78
129.76
129.37
129.36
129.33
128.47
128.35
127.68
127.37
127.34
126.74
117.83
62.72
62.64
62.26
62.18
39.65
38.27
36.03
36.00
16.57
16.52
16.46
16.40
PO(OEt)2
N
Ph
f1 (ppm)

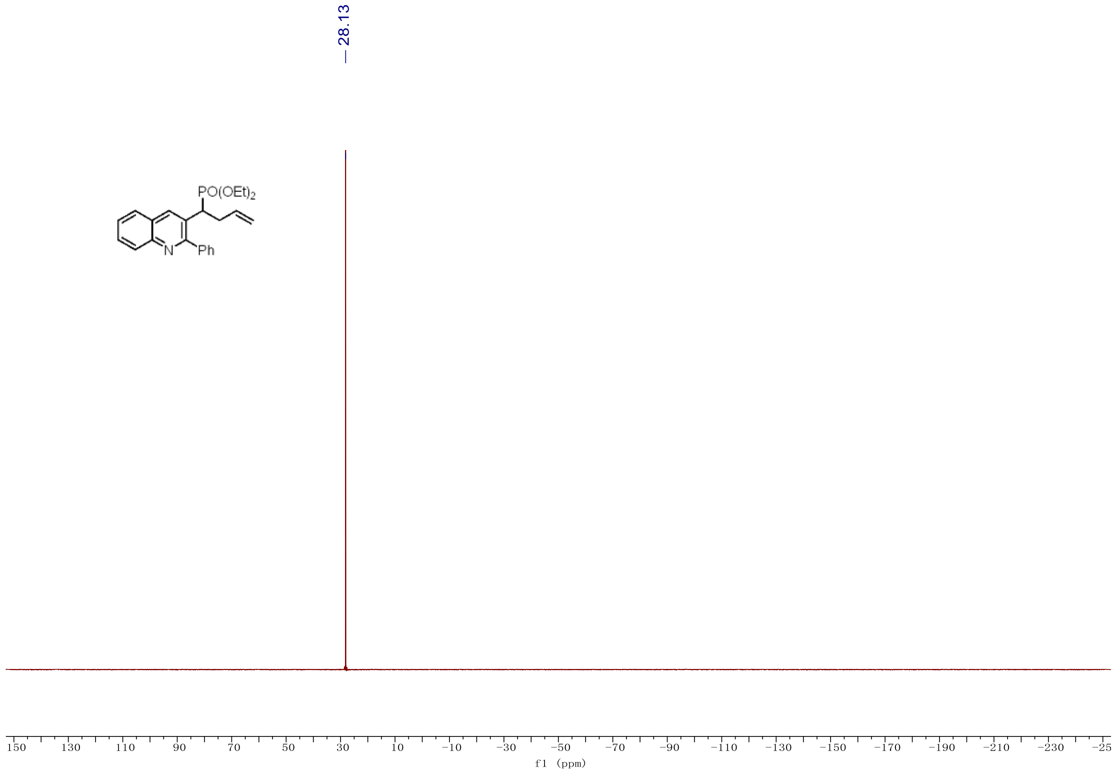
28.13
PO(OEt)2
N
Ph
f1 (ppm)

13

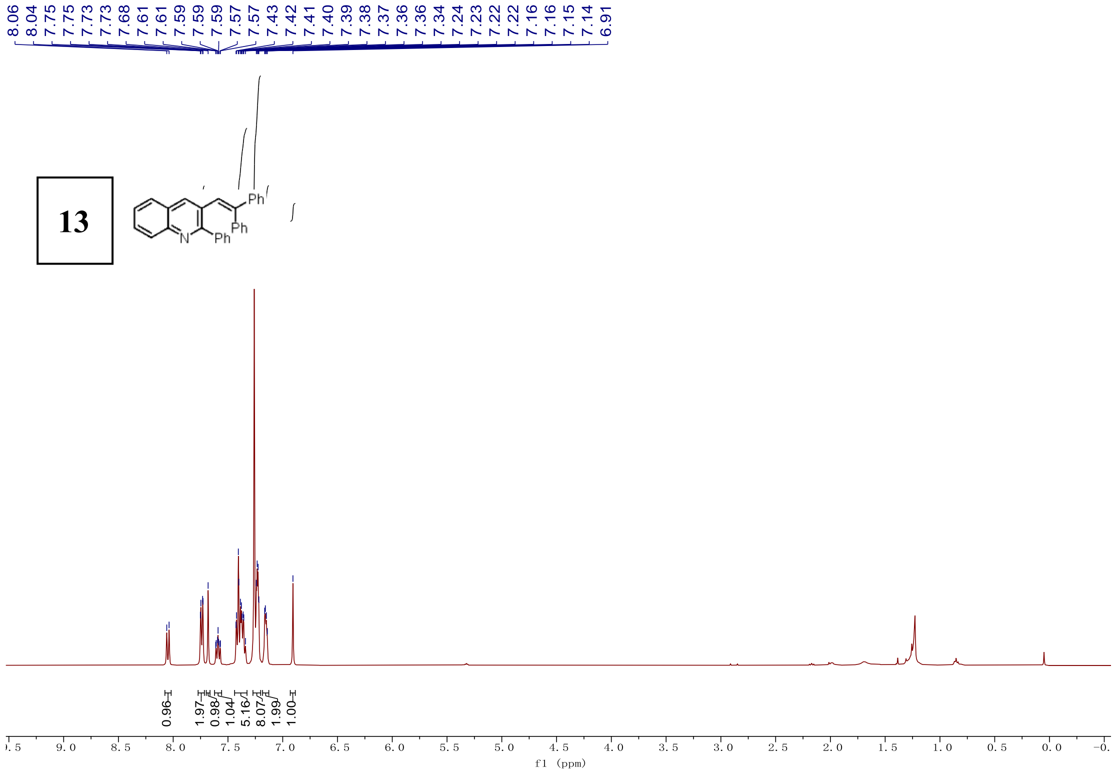
8.06
8.04
7.75
7.75
7.73
7.73
7.68
7.61
7.59
7.59
7.57
7.57
7.43
7.42
7.41
7.40
7.39
7.38
7.37
7.36
7.36
7.34
7.24
7.23
7.22
7.22
7.16
7.15
7.14
6.91
Ph
N
Ph
Ph
0.96
1.97
0.98
1.04
5.16
8.07
1.99
1.00
. 5
9. 0
8. 5
8. 0
7. 5
7. 0
6. 5
6. 0
5. 5
5. 0
4. 5
4. 0
3. 5
3. 0
2. 5
2. 0
1. 5
1. 0
0. 5
0. 0
-0.
f1 (ppm)

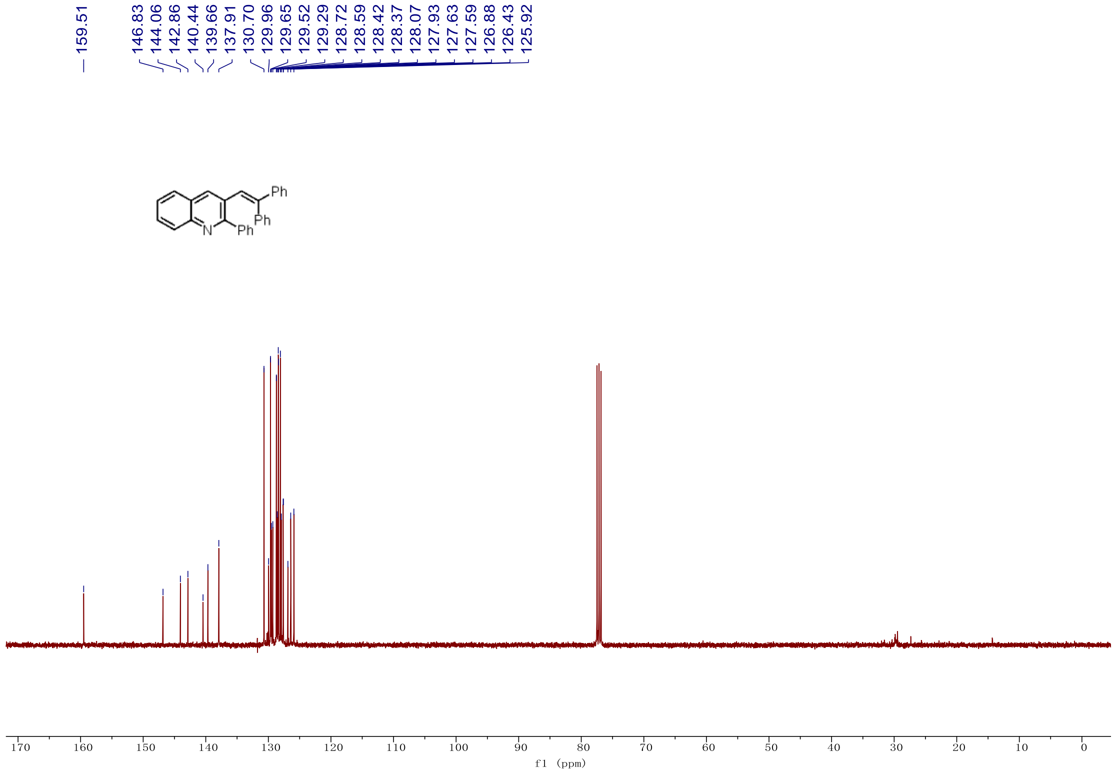
159.51
146.83
144.06
142.86
140.44
139.66
137.91
130.70
129.96
129.65
129.52
129.29
128.72
128.59
128.42
128.37
128.07
127.93
127.63
127.59
126.88
126.43
125.92
Ph
N
Ph
Ph
170
160
150
140
130
120
110
100
90
80
70
60
50
40
30
20
10
0
f1 (ppm)

14

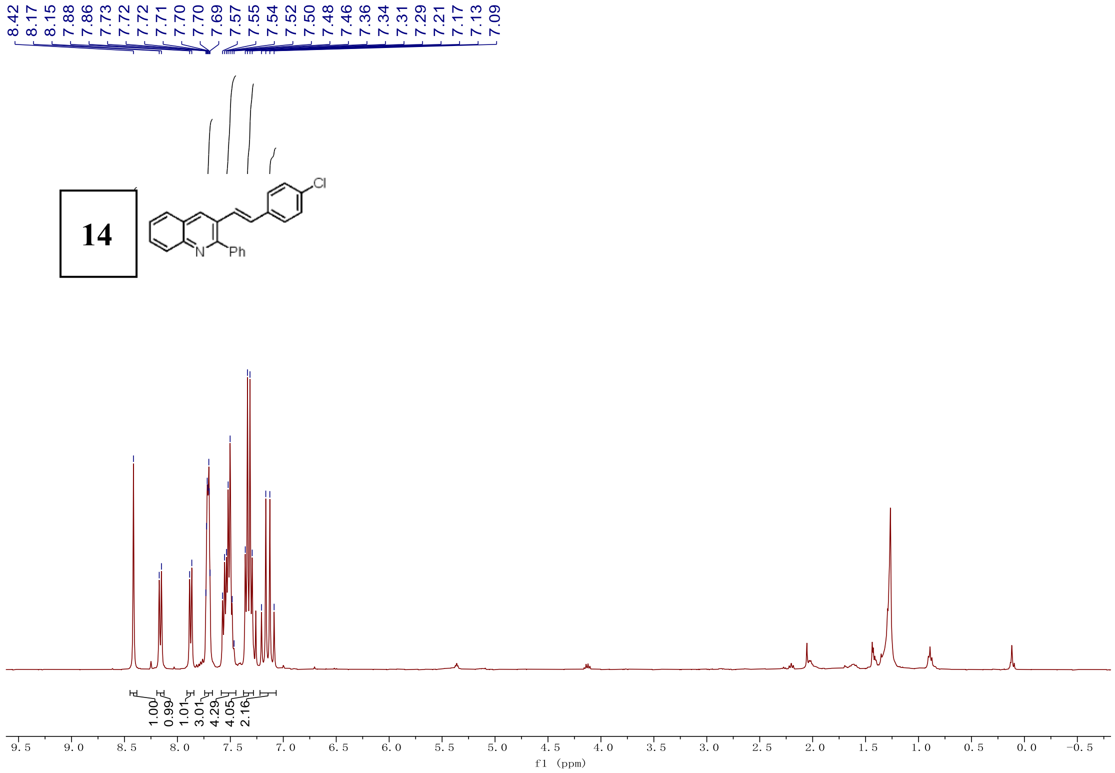
8.42
8.17
8.15
7.88
7.86
7.73
7.72
7.72
7.71
7.70
7.70
7.69
7.57
7.55
7.54
7.52
7.50
7.48
7.46
7.36
7.34
7.31
7.29
7.21
7.17
7.13
7.09
Cl
N
Ph
1.00
0.99
1.01
3.01
4.29
4.05
2.16
f1 (ppm)

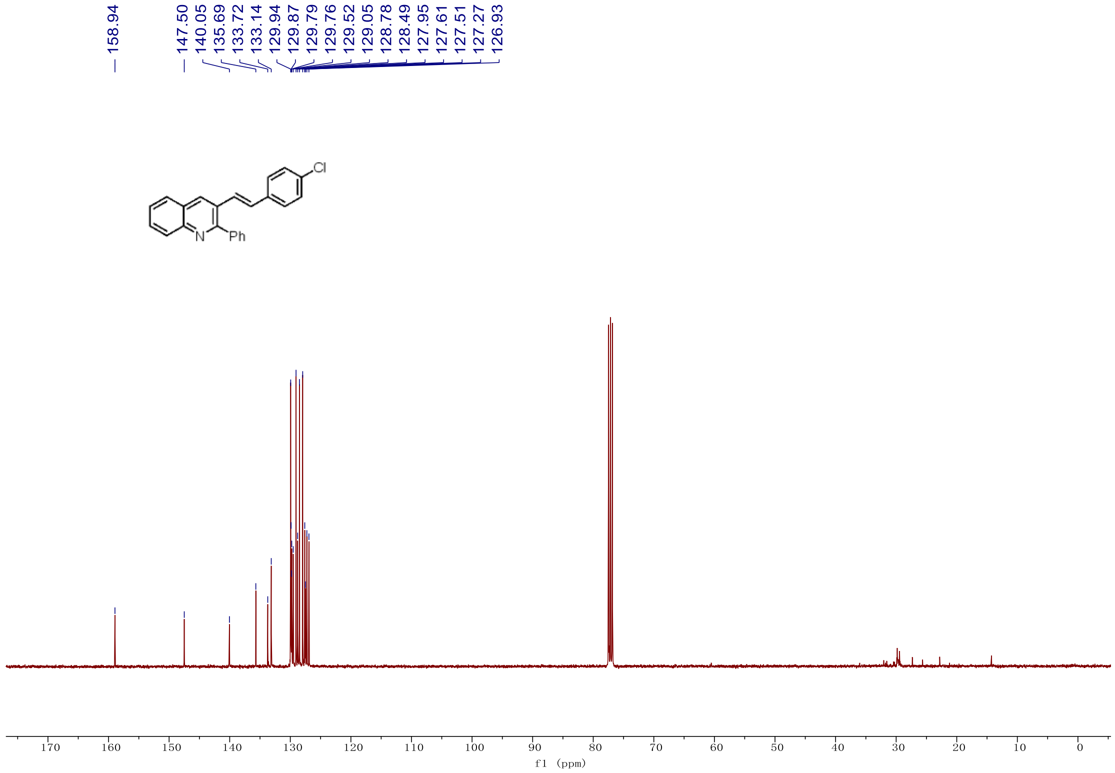
158.94
147.50
140.05
135.69
133.72
133.14
129.94
129.87
129.79
129.76
129.52
129.05
128.78
128.49
127.95
127.61
127.51
127.27
126.93
Cl
N
Ph
f1 (ppm)

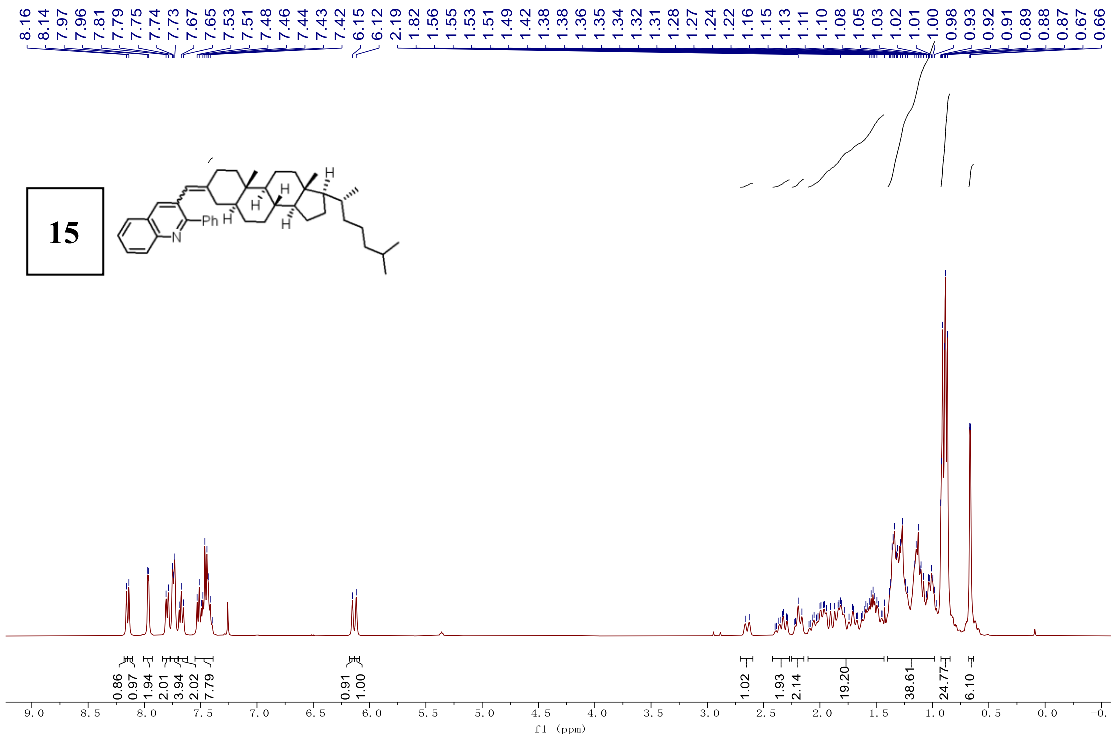
8.16 8.14 7.97 7.96 7.81 7.79 7.75 7.74 7.73 7.67 7.65 7.53 7.51 7.48 7.46 7.44 7.43 7.42 6.15 6.12 2.19 1.82 1.56 1.55 1.53 1.51 1.49 1.42 1.38 1.38 1.36 1.35 1.34 1.32 1.31 1.28 1.27 1.24 1.22 1.16 1.15 1.13 1.11 1.10 1.08 1.05 1.03 1.02 1.01 1.00 0.98 0.93 0.92 0.91 0.89 0.88 0.87 0.67 0.66
15
Ph
N
0.86 0.97 1.94 2.01 3.94 2.02 7.79 0.91 1.00 1.02 1.93 2.14 19.20 38.61 24.77 6.10
9.0 8.5 8.0 7.5 7.0 6.5 6.0 5.5 5.0 4.5 4.0 3.5 3.0 2.5 2.0 1.5 1.0 0.5 0.0 -0.
f1 (ppm)

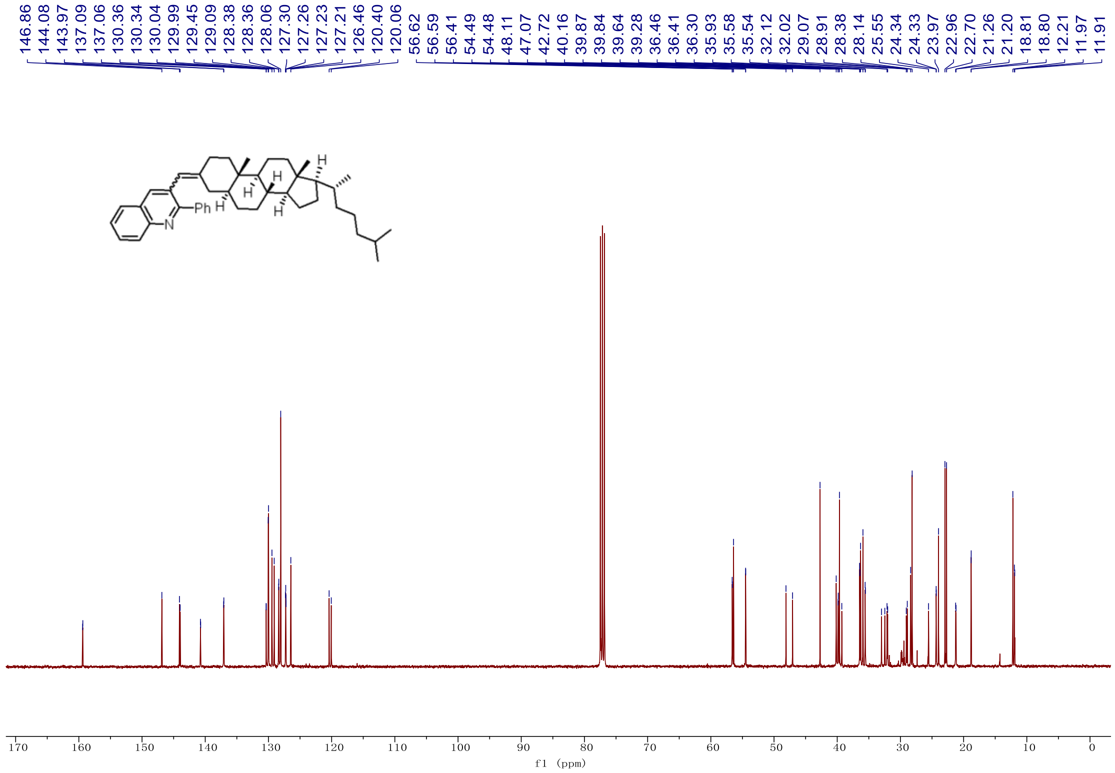
146.86 144.08 143.97 137.09 137.06 130.36 130.34 130.04 129.99 129.45 129.09 128.38 128.36 128.06 127.30 127.26 127.23 127.21 126.46 120.40 120.06 56.62 56.59 56.41 54.49 54.48 48.11 47.07 42.72 40.16 39.87 39.84 39.64 39.28 36.46 36.41 36.30 35.93 35.58 35.54 32.12 32.02 29.07 28.91 28.38 28.14 25.55 24.34 24.33 23.97 22.96 22.70 21.26 21.20 18.81 18.80 12.21 11.97 11.91
Ph
N
170 160 150 140 130 120 110 100 90 80 70 60 50 40 30 20 10 0
f1 (ppm)

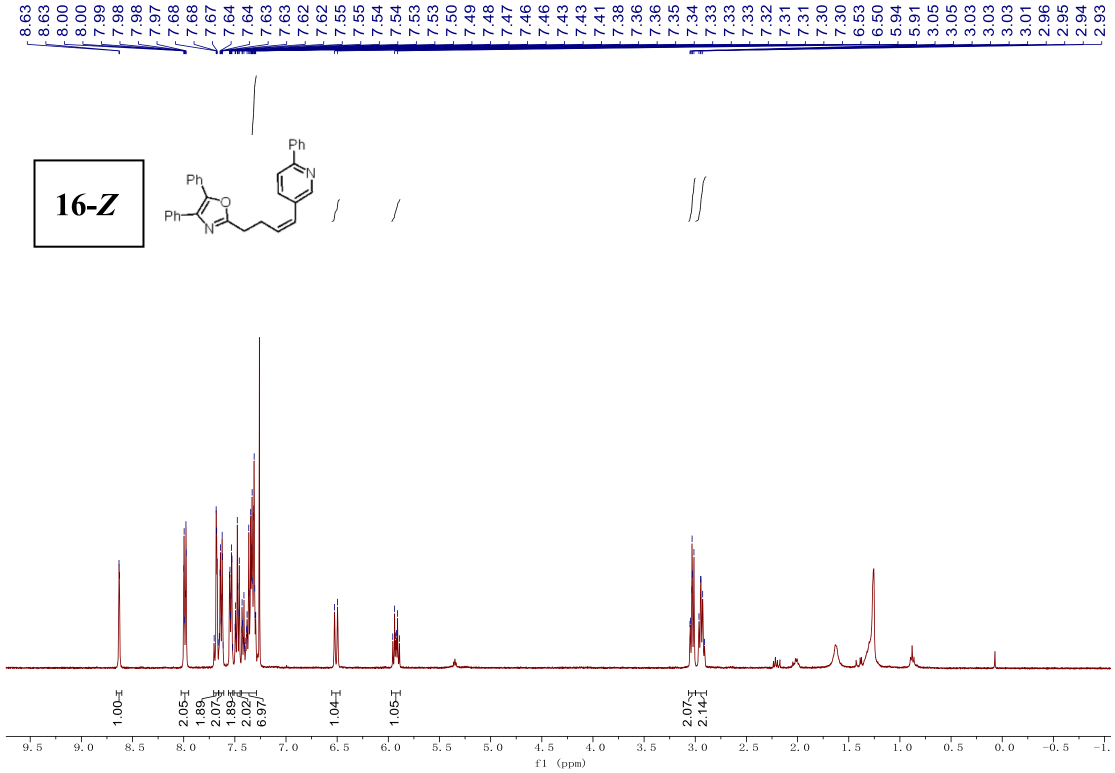
8.63 8.63 8.00 8.00 7.99 7.98 7.98 7.97 7.68 7.68 7.67 7.64 7.64 7.63 7.63 7.62 7.62 7.55 7.55 7.54 7.54 7.53 7.53 7.50 7.49 7.48 7.47 7.46 7.46 7.43 7.43 7.41 7.38 7.36 7.36 7.35 7.34 7.33 7.33 7.33 7.32 7.31 7.31 7.30 7.30 6.53 6.50 5.94 5.91 3.05 3.05 3.03 3.03 3.03 3.01 2.96 2.95 2.94 2.93
16-Z
Ph
Ph
Ph
O
N
N
1.00
2.05
1.89
2.07
1.89
2.02
6.97
1.04
1.05
2.07
2.14
9.5 9.0 8.5 8.0 7.5 7.0 6.5 6.0 5.5 5.0 4.5 4.0 3.5 3.0 2.5 2.0 1.5 1.0 0.5 0.0 -0.5 -1.
f1 (ppm)

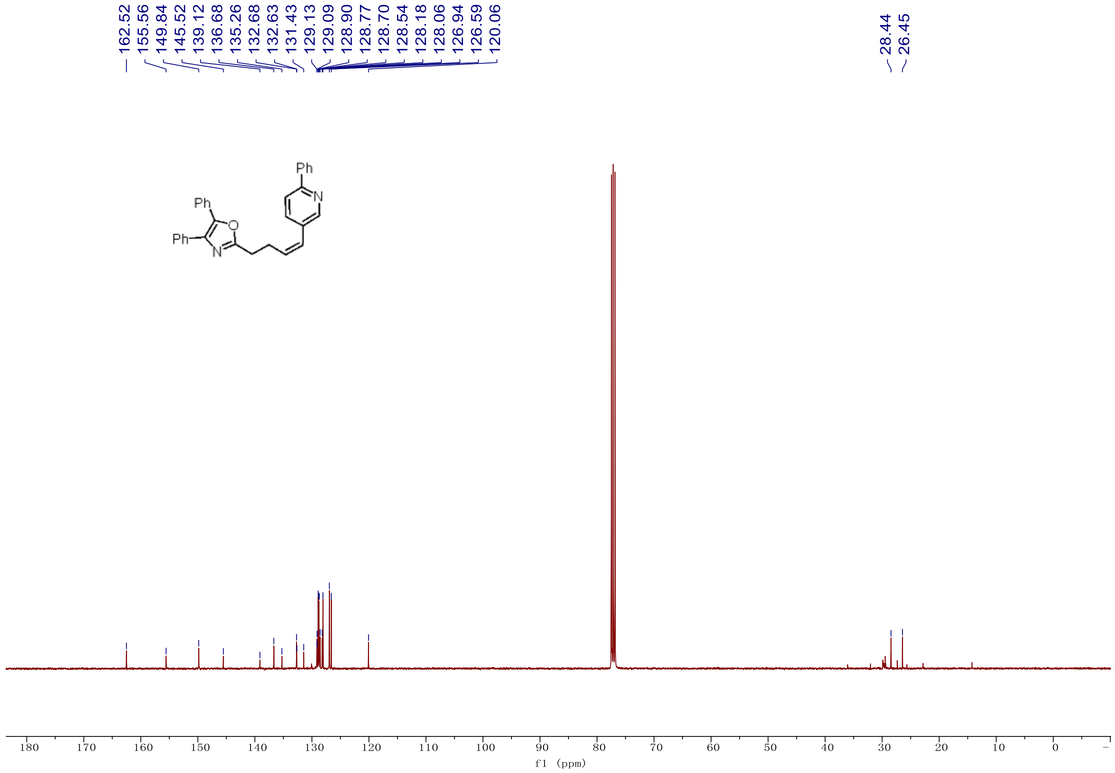
162.52 155.56 149.84 145.52 139.12 136.68 135.26 132.68 132.63 131.43 129.13 129.09 128.90 128.77 128.70 128.54 128.18 128.06 126.94 126.59 120.06
28.44 26.45
Ph
Ph
Ph
O
N
N
180 170 160 150 140 130 120 110 100 90 80 70 60 50 40 30 20 10 0
f1 (ppm)

16-*E*

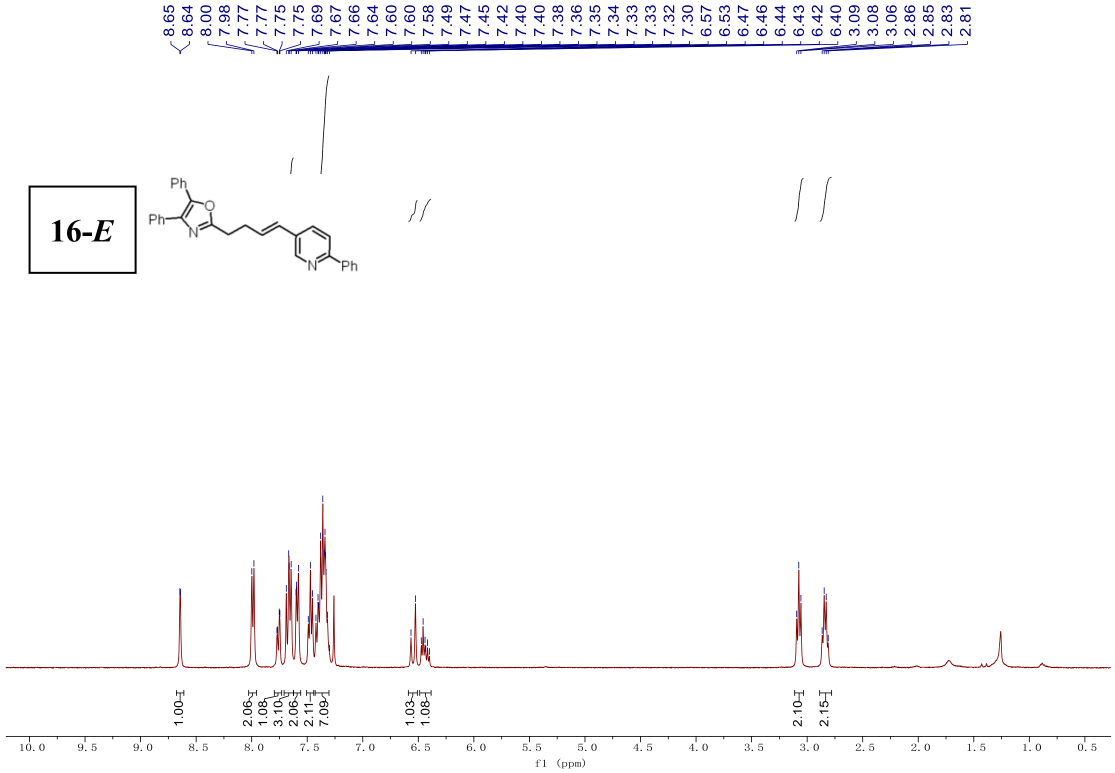
8.65
8.64
8.00
7.98
7.77
7.77
7.75
7.75
7.69
7.67
7.66
7.64
7.60
7.60
7.58
7.49
7.47
7.45
7.42
7.40
7.40
7.38
7.36
7.35
7.34
7.33
7.33
7.32
7.30
6.57
6.53
6.47
6.46
6.44
6.43
6.42
6.40
3.09
3.08
3.06
2.86
2.85
2.83
2.81
Ph
Ph
O
N
N
Ph
1.00
2.06
1.08
3.10
2.06
2.11
7.09
1.03
1.08
2.10
2.15
f1 (ppm)

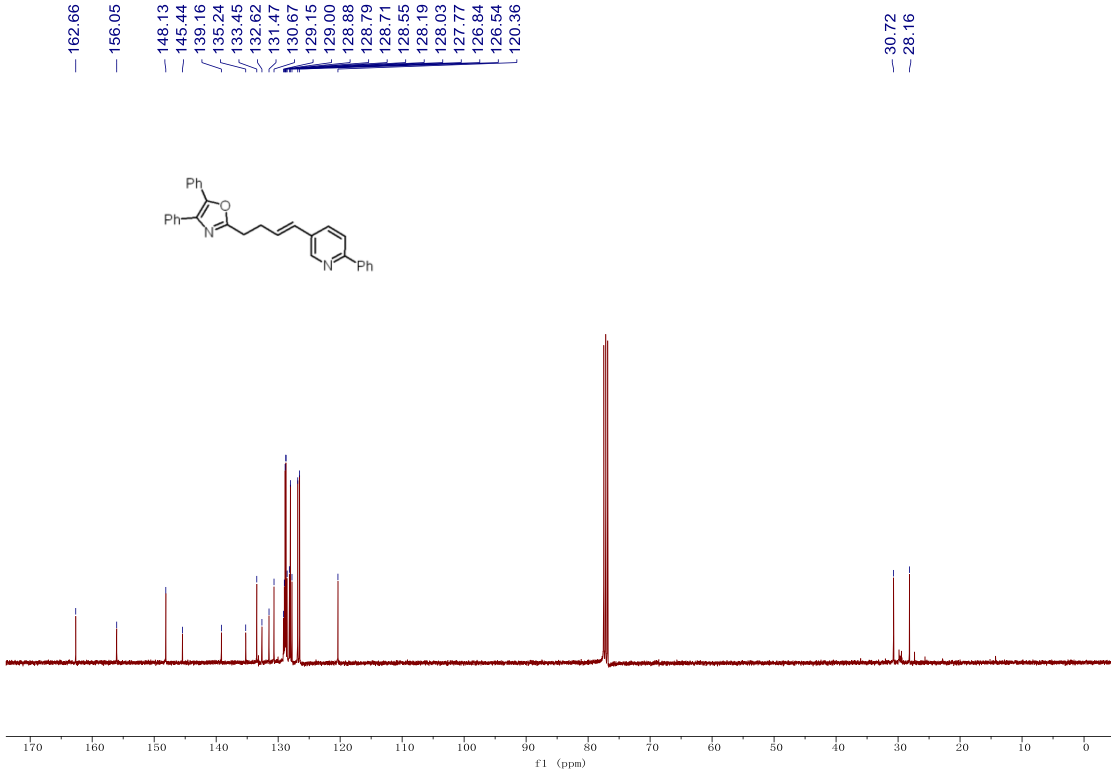
162.66
156.05
148.13
145.44
139.16
135.24
133.45
132.62
131.47
130.67
129.15
129.00
128.88
128.79
128.71
128.55
128.19
128.03
127.77
126.84
126.54
120.36
30.72
28.16
Ph
Ph
O
N
N
Ph
f1 (ppm)

4a

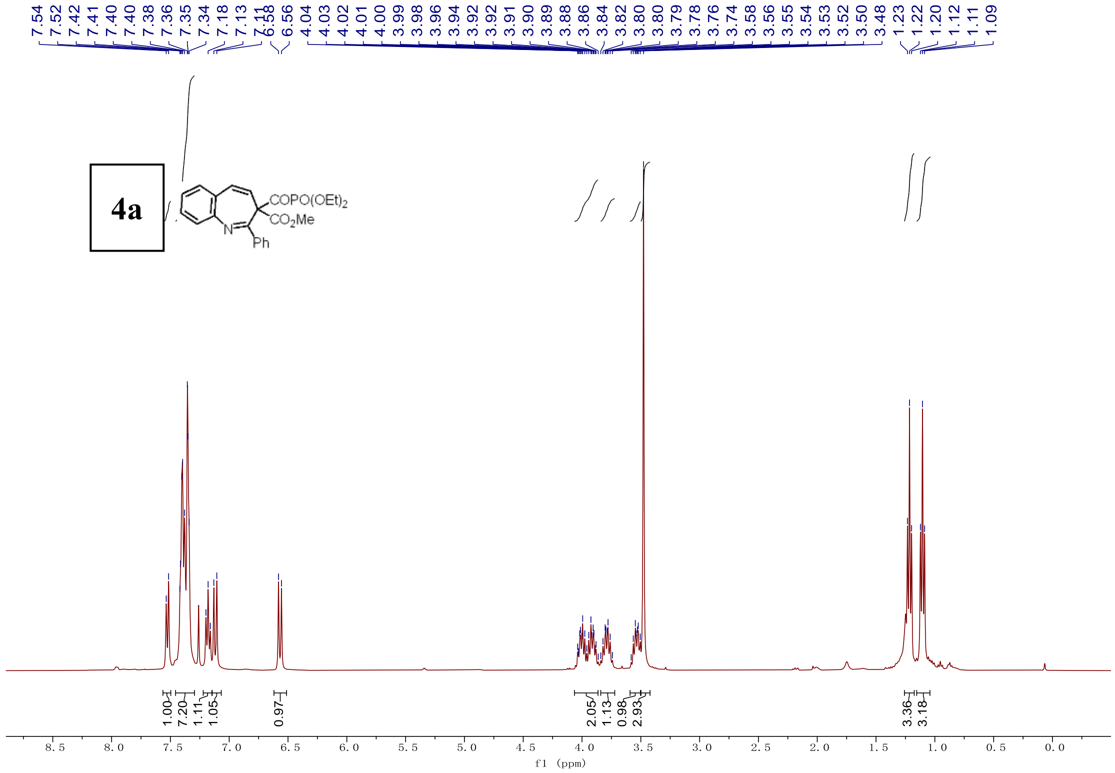
7.54
7.52
7.42
7.41
7.40
7.40
7.38
7.36
7.35
7.34
7.18
7.13
7.11
6.58
6.56
4.04
4.03
4.02
4.01
4.00
3.99
3.98
3.96
3.94
3.92
3.92
3.91
3.90
3.89
3.88
3.86
3.84
3.82
3.80
3.80
3.79
3.78
3.76
3.74
3.58
3.56
3.55
3.54
3.53
3.52
3.50
3.48
1.23
1.22
1.20
1.12
1.11
1.09
COPO(OEt)2
CO2Me
N
Ph
1.00
7.20
1.11
1.05
0.97
2.05
1.13
0.98
2.93
3.36
3.18
f1 (ppm)

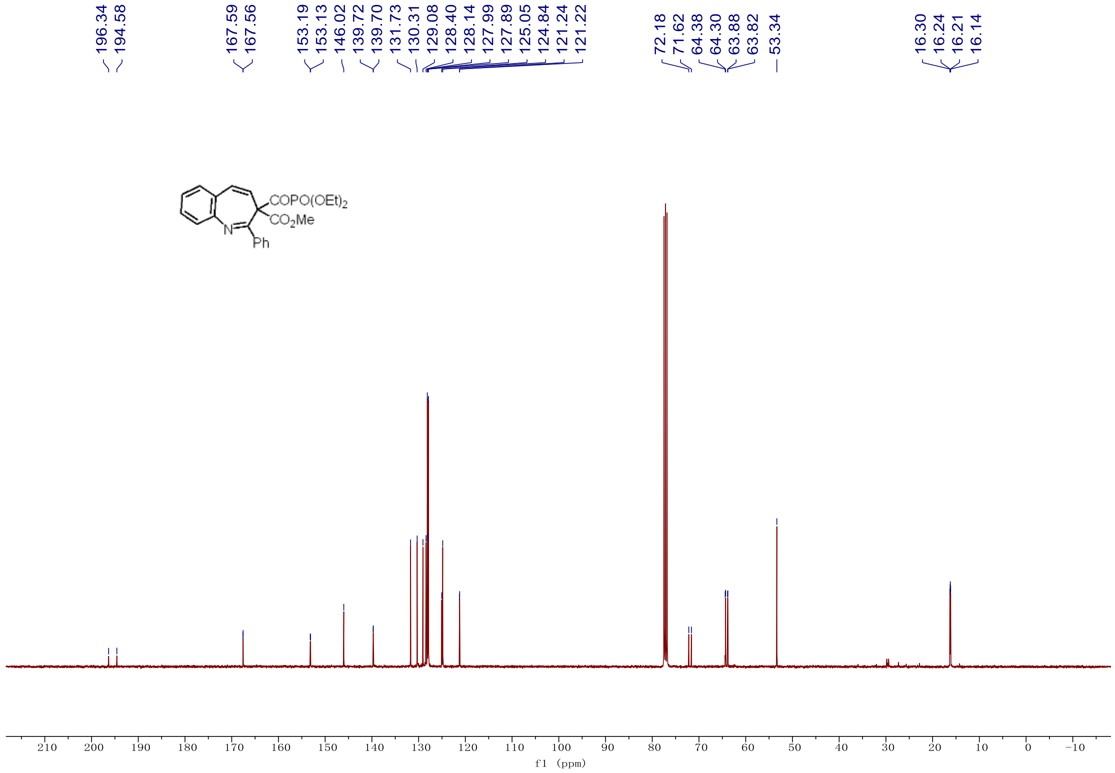
196.34
194.58
167.59
167.56
153.19
153.13
146.02
139.72
139.70
131.73
130.31
129.08
128.40
128.14
127.99
127.89
125.05
124.84
121.24
121.22
72.18
71.62
64.38
64.30
63.88
63.82
53.34
16.30
16.24
16.21
16.14
COPO(OEt)2
CO2Me
N
Ph
f1 (ppm)

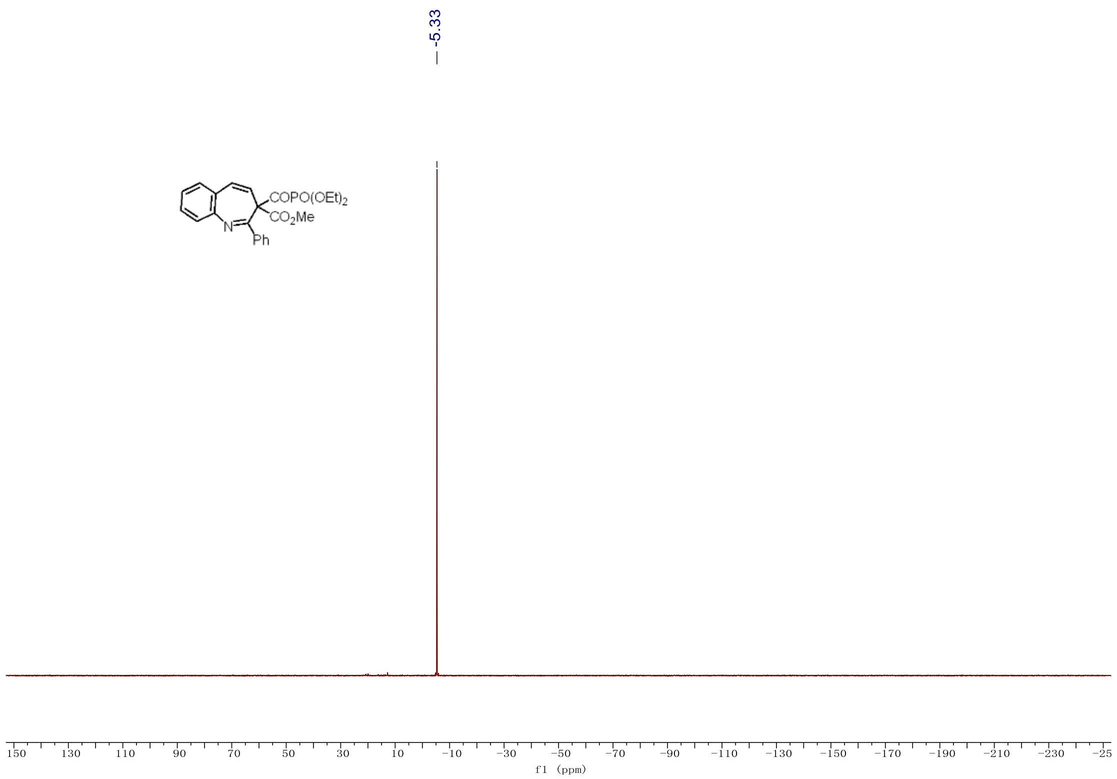
-5.33
COPO(OEt)2
CO2Me
N
Ph
f1 (ppm)

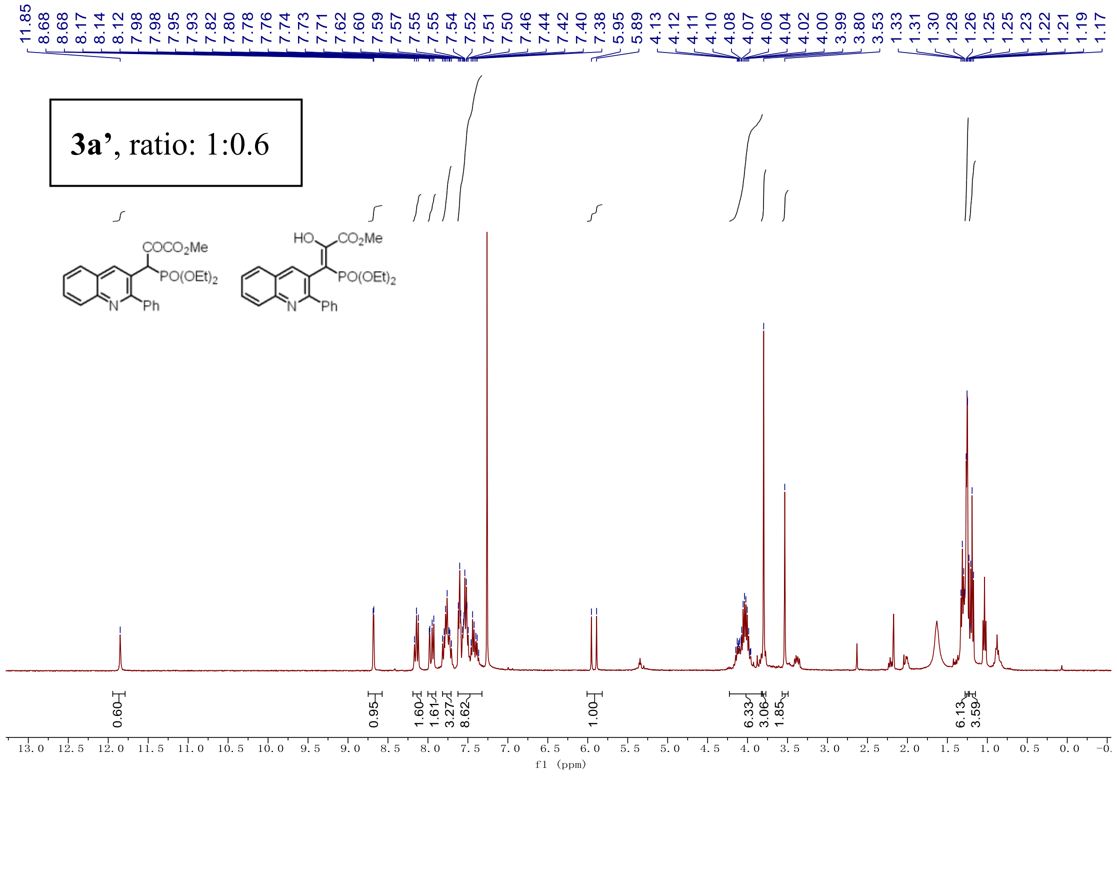
3a', ratio: 1:0.6
COCO2Me
PO(OEt)2
N
Ph
HO
CO2Me
PO(OEt)2
N
Ph
f1 (ppm)

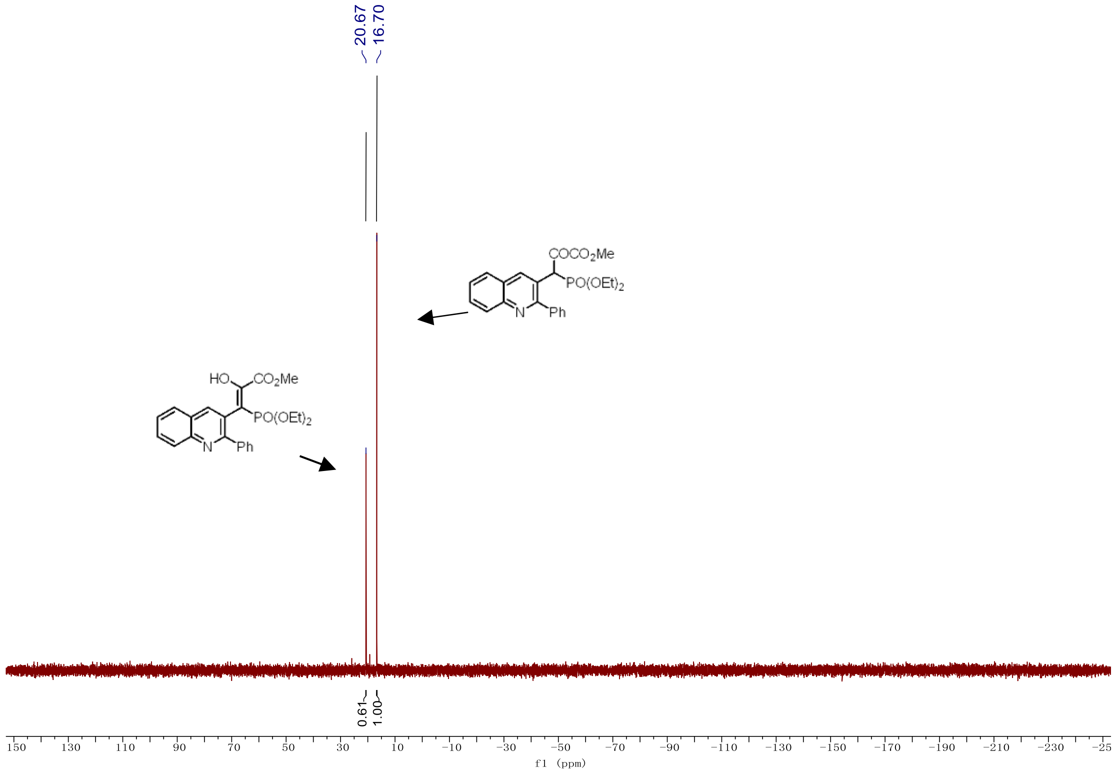
20.67
16.70
COCO2Me
PO(OEt)2
N
Ph
HO
CO2Me
PO(OEt)2
N
Ph
0.61
1.00
f1 (ppm)

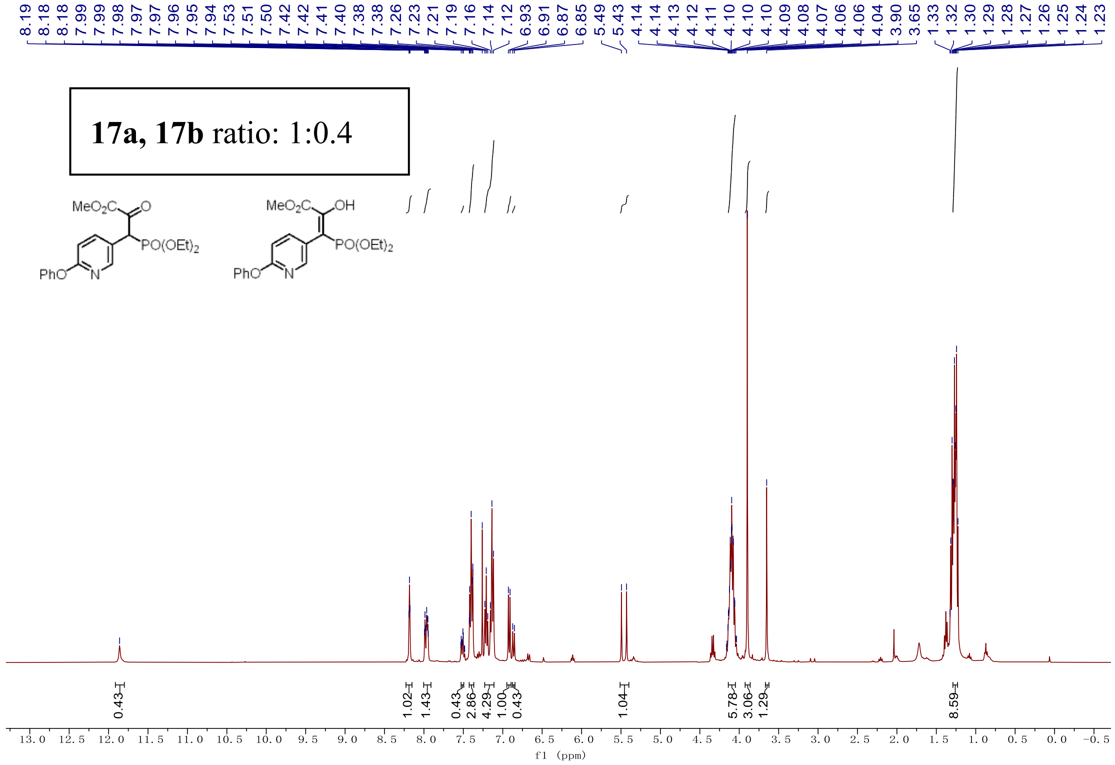
17a, 17b ratio: 1:0.4
MeO2C
O
PO(OEt)2
PhO
N
MeO2C
OH
PO(OEt)2
PhO
N
f1 (ppm)

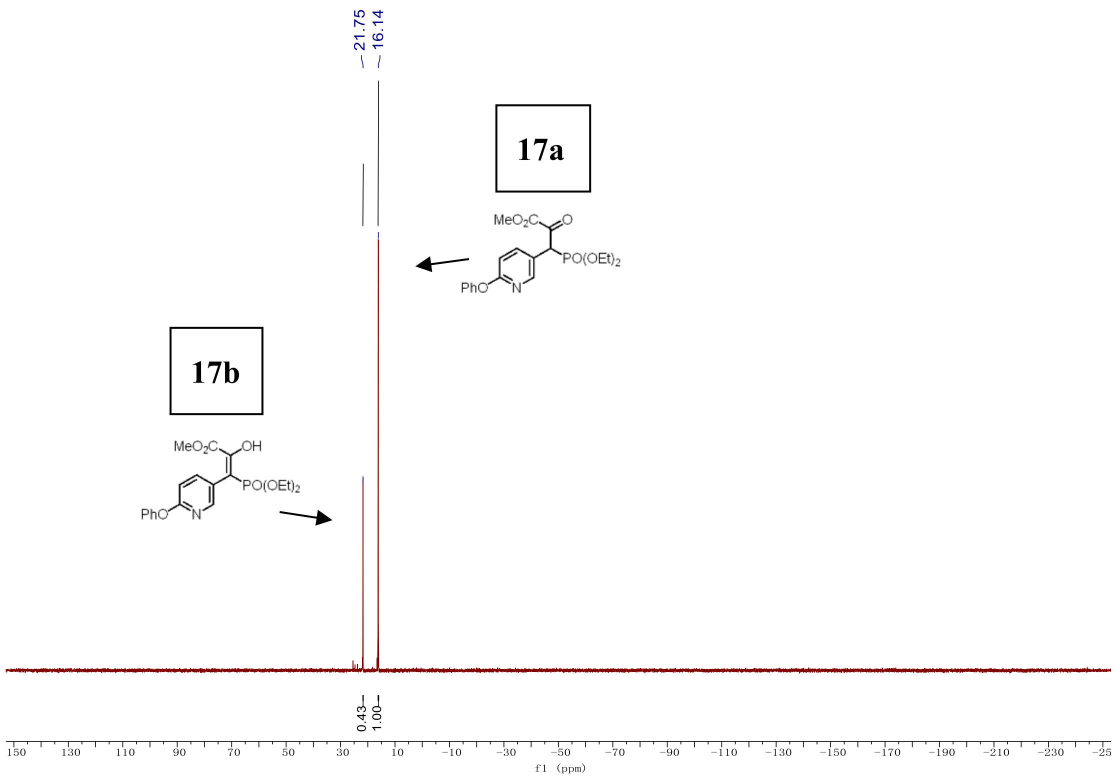
21.75
16.14
17a
MeO_2C
O
$PO(OEt)_2$
PhO
N
17b
MeO_2C
OH
$PO(OEt)_2$
PhO
N
0.43
1.00
f1 (ppm)

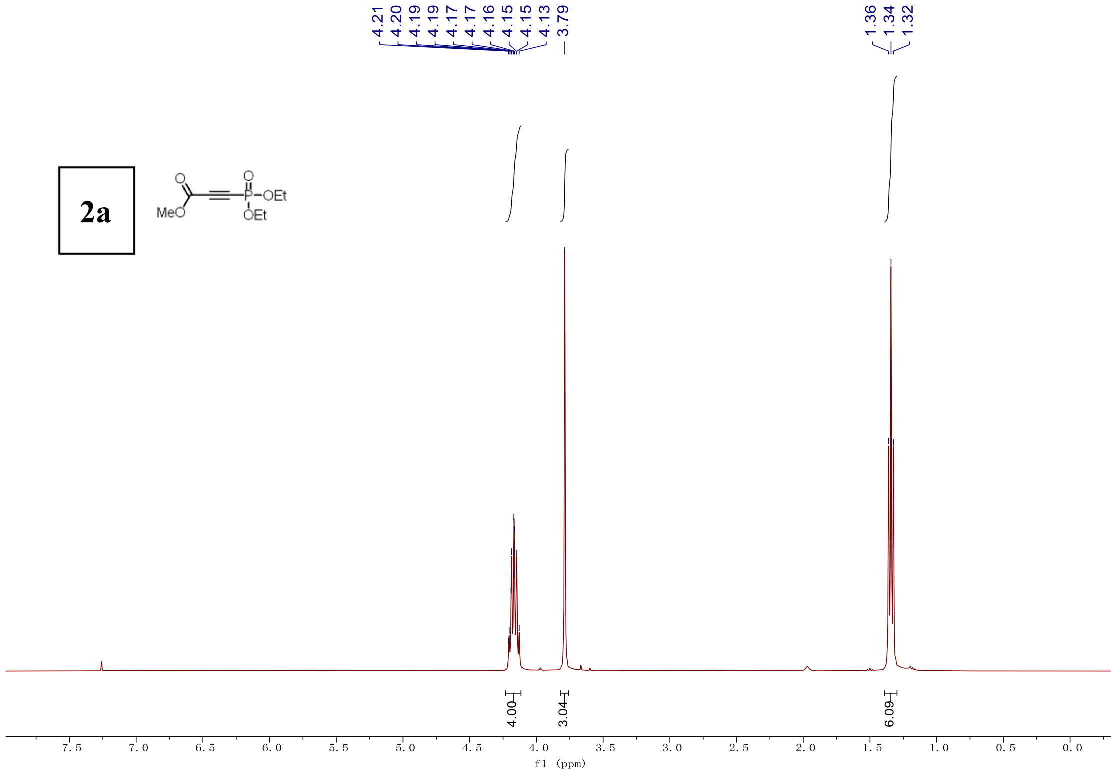
4.21
4.20
4.19
4.19
4.17
4.17
4.16
4.15
4.15
4.13
3.79
1.36
1.34
1.32
2a
O
MeO
O
P
OEt
OEt
4.00
3.04
6.09
f1 (ppm)

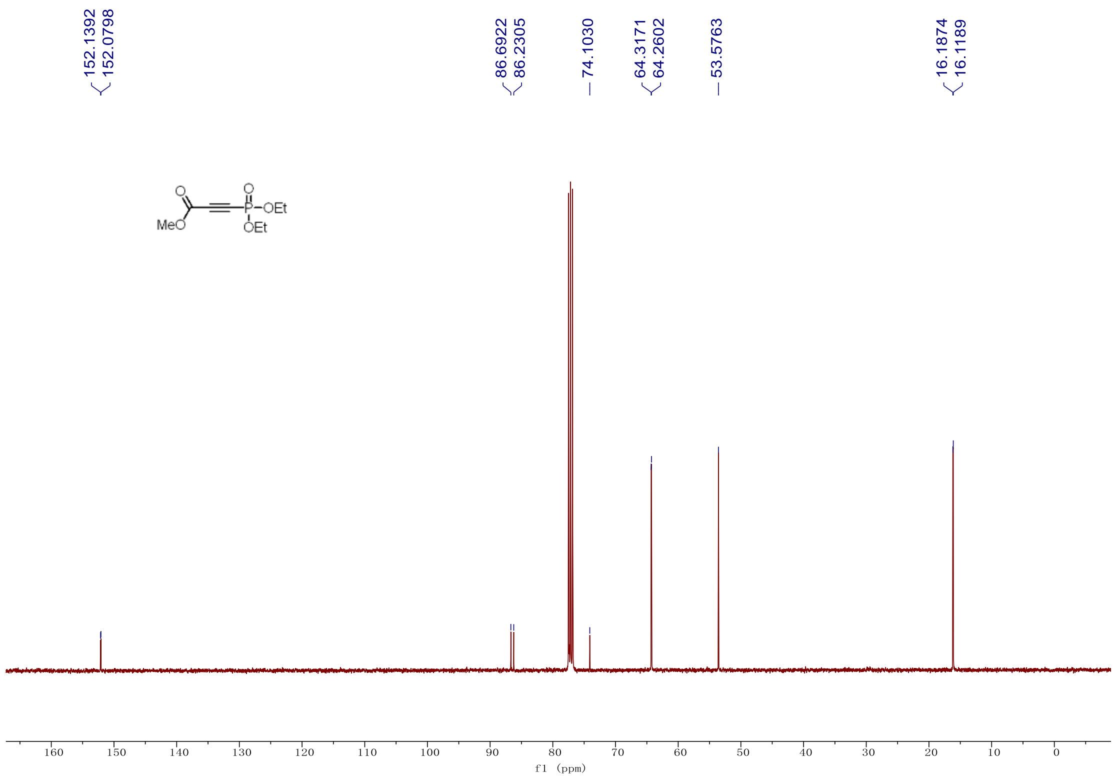
152.1392
152.0798
86.6922
86.2305
74.1030
64.3171
64.2602
53.5763
16.1874
16.1189
MeO
OEt
OEt
f1 (ppm)

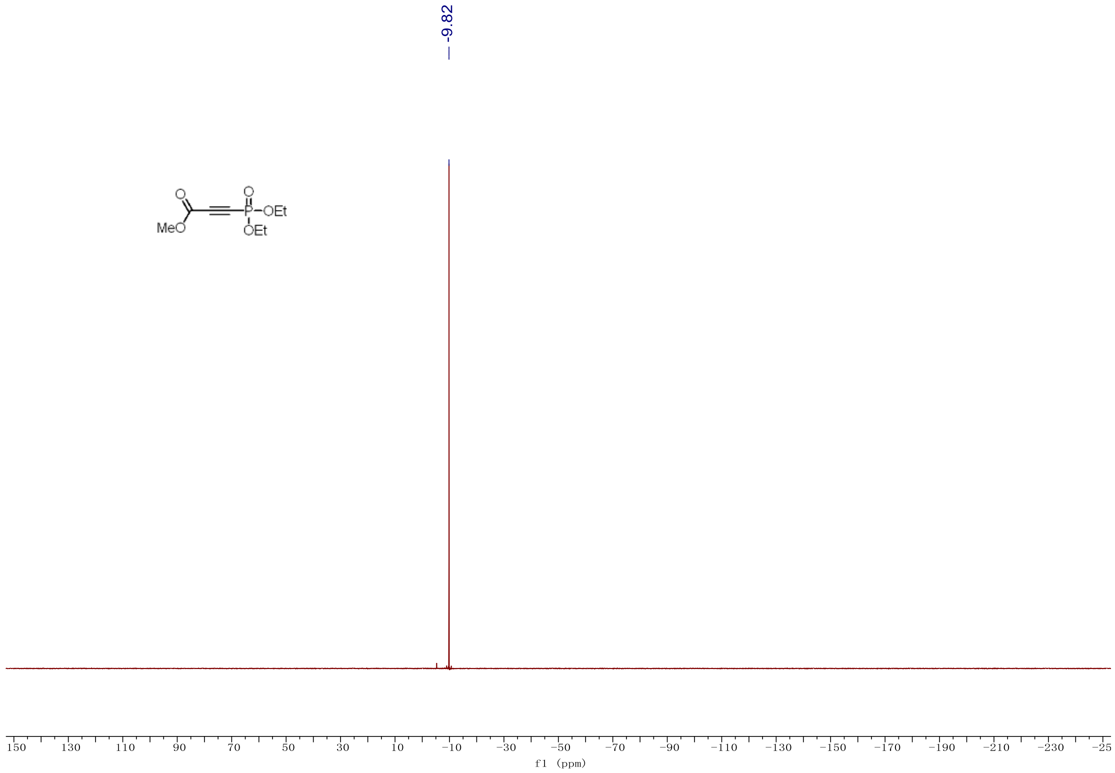
-9.82
MeO
OEt
OEt
f1 (ppm)

2b

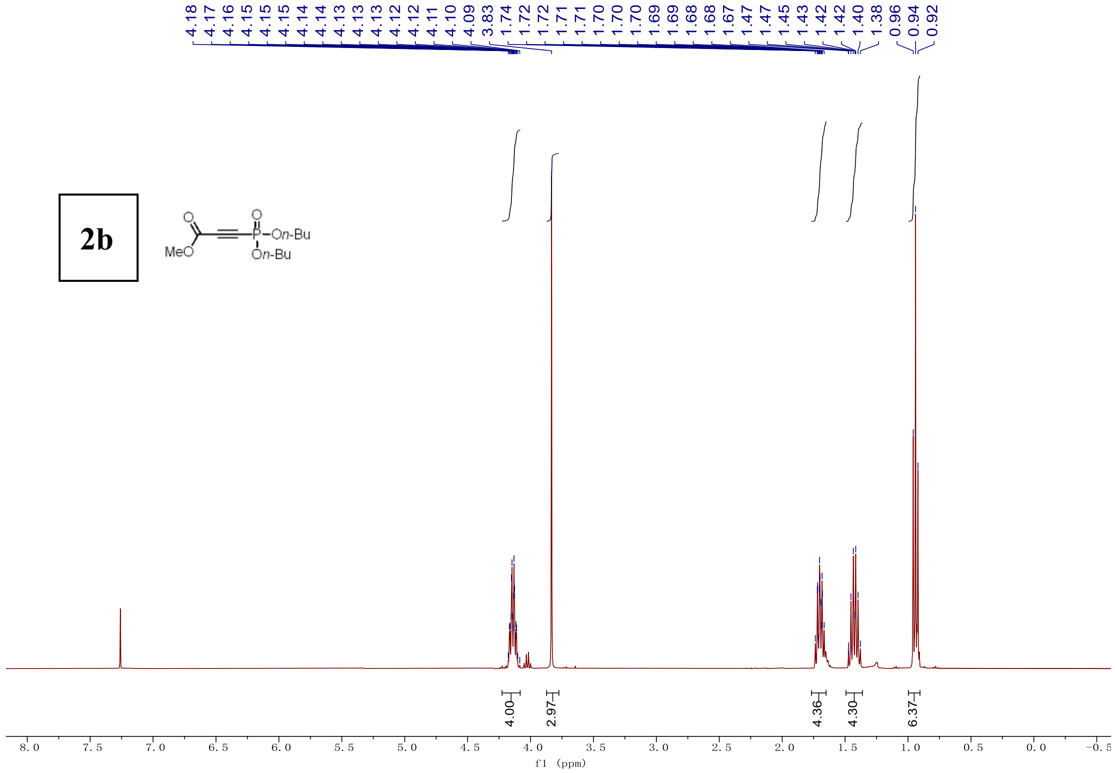
4.18
4.17
4.16
4.15
4.15
4.15
4.14
4.14
4.13
4.13
4.13
4.12
4.12
4.11
4.10
4.09
3.83
1.74
1.72
1.72
1.71
1.71
1.70
1.70
1.70
1.69
1.69
1.68
1.68
1.67
1.47
1.47
1.45
1.43
1.42
1.42
1.40
1.38
0.96
0.94
0.92
MeO
On-Bu
On-Bu
4.00
2.97
4.36
4.30
6.37
f1 (ppm)

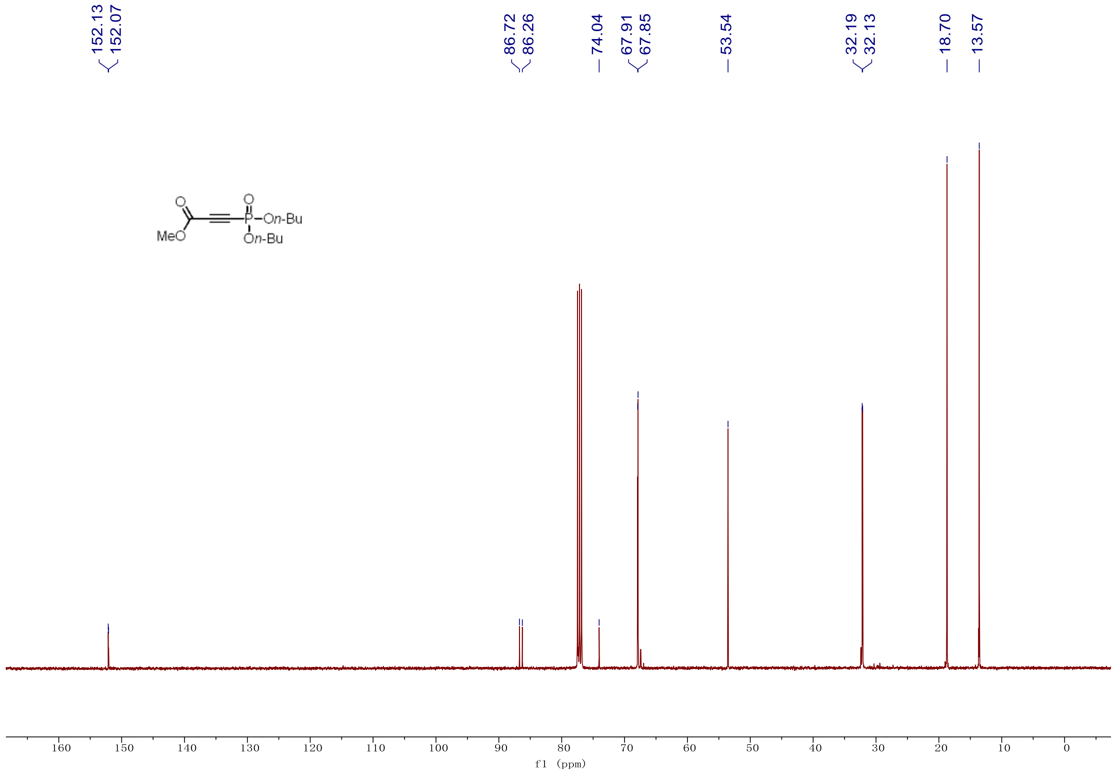
152.13
152.07
86.72
86.26
74.04
67.91
67.85
53.54
32.19
32.13
18.70
13.57
MeO
On-Bu
On-Bu
f1 (ppm)

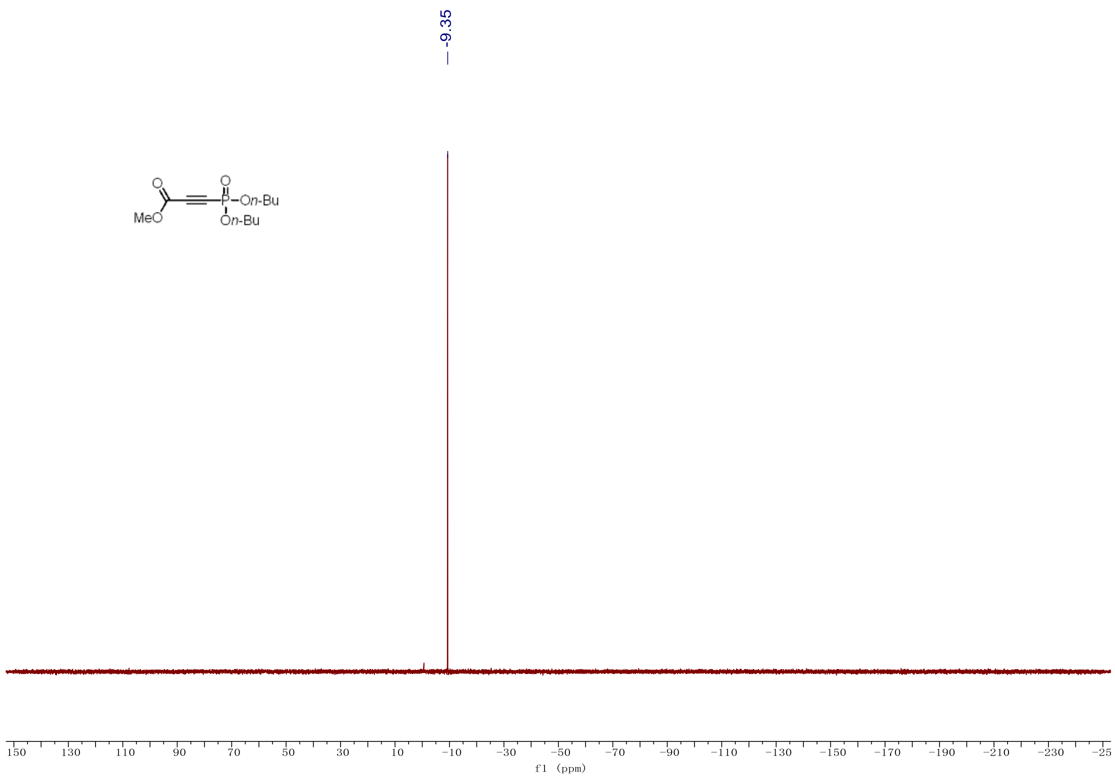

-9.35
On-Bu
On-Bu
MeO
f1 (ppm)

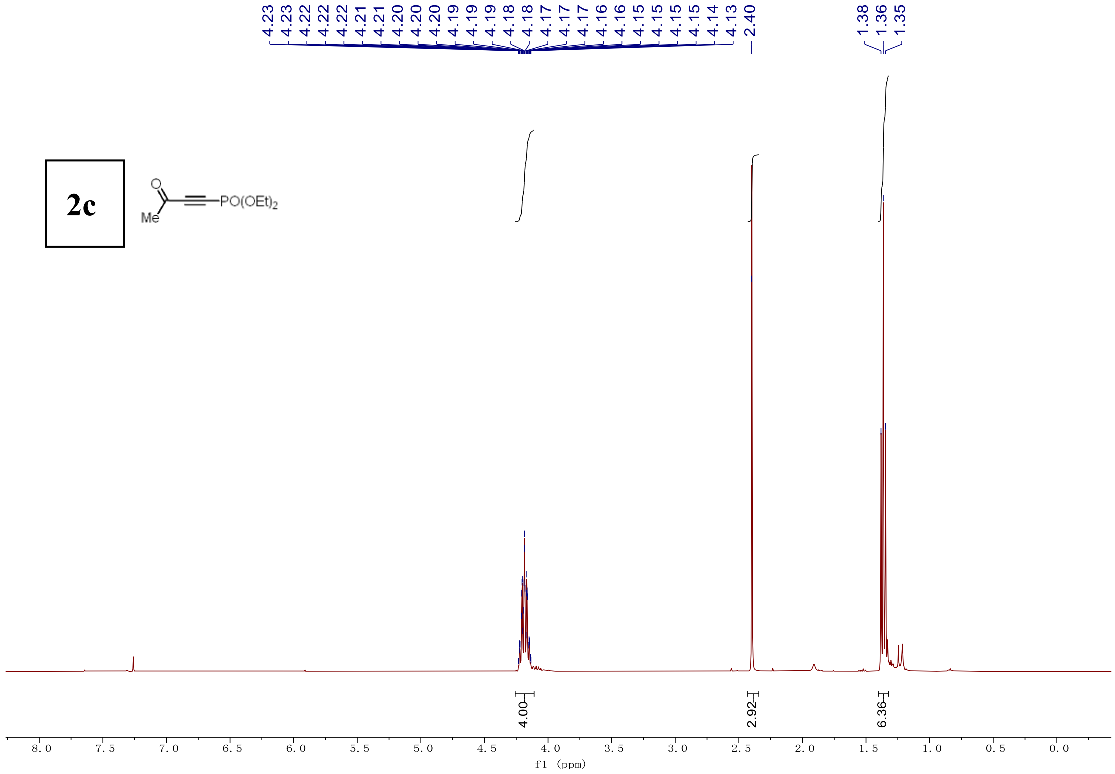

4.23 4.23 4.22 4.22 4.22 4.21 4.21 4.20 4.20 4.20 4.19 4.19 4.19 4.18 4.18 4.17 4.17 4.17 4.16 4.16 4.15 4.15 4.15 4.15 4.14 4.13
2.40
1.38 1.36 1.35
2c
PO(OEt)2
Me
4.00
2.92
6.36
f1 (ppm)

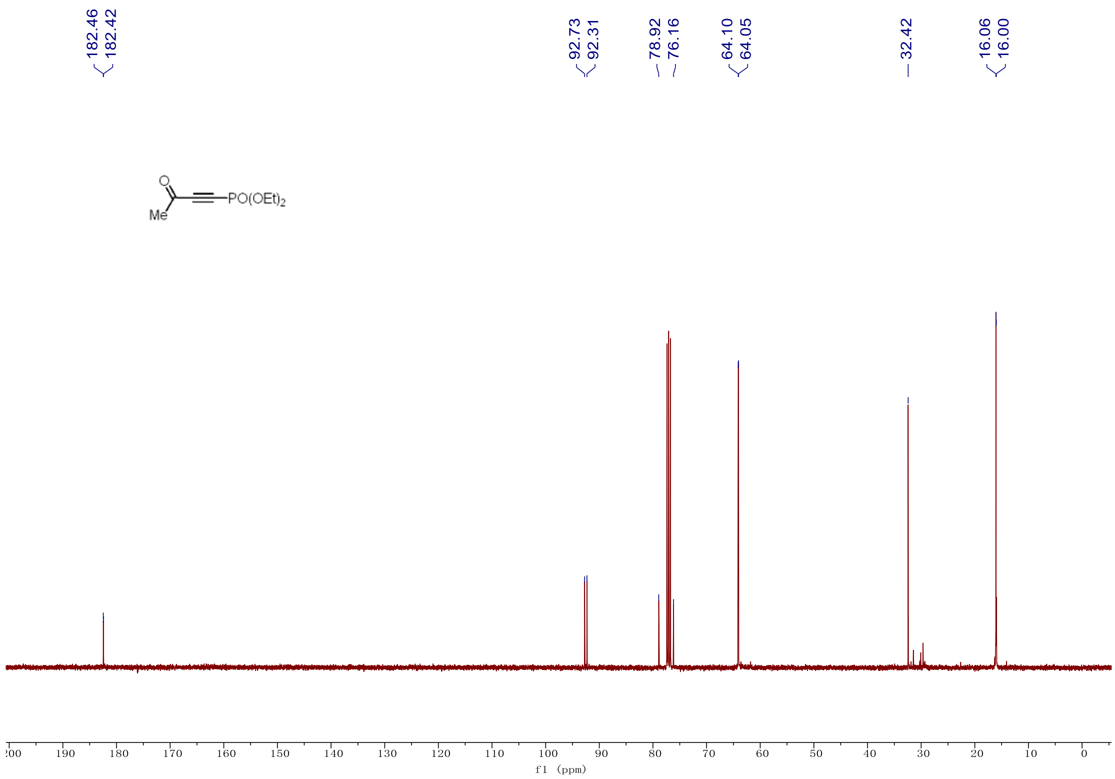
182.46
182.42
92.73
92.31
78.92
76.16
64.10
64.05
32.42
16.06
16.00
PO(OEt)2
Me
O
f1 (ppm)

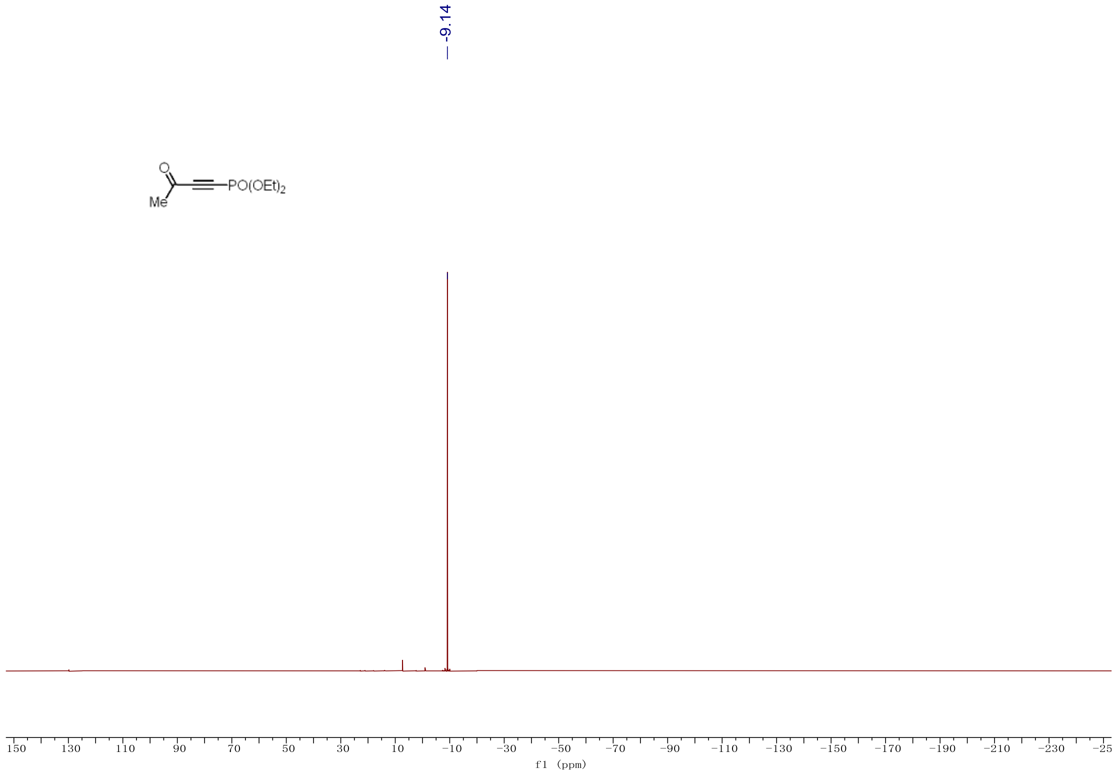
-9.14
PO(OEt)2
Me
O
f1 (ppm)

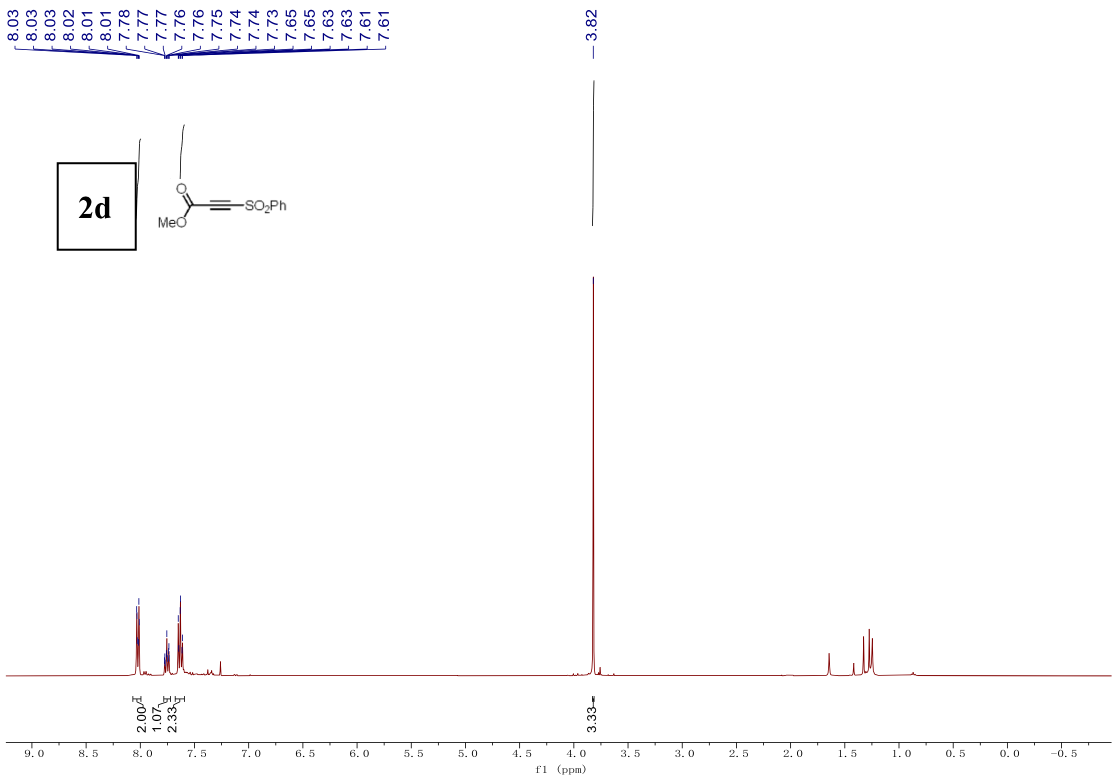
8.03
8.03
8.03
8.02
8.01
8.01
7.78
7.77
7.77
7.76
7.76
7.75
7.74
7.74
7.73
7.65
7.65
7.63
7.63
7.61
7.61
3.82
2d
MeO
O
SO2Ph
2.00
1.07
2.33
3.33
9.0 8.5 8.0 7.5 7.0 6.5 6.0 5.5 5.0 4.5 4.0 3.5 3.0 2.5 2.0 1.5 1.0 0.5 0.0 -0.5
f1 (ppm)

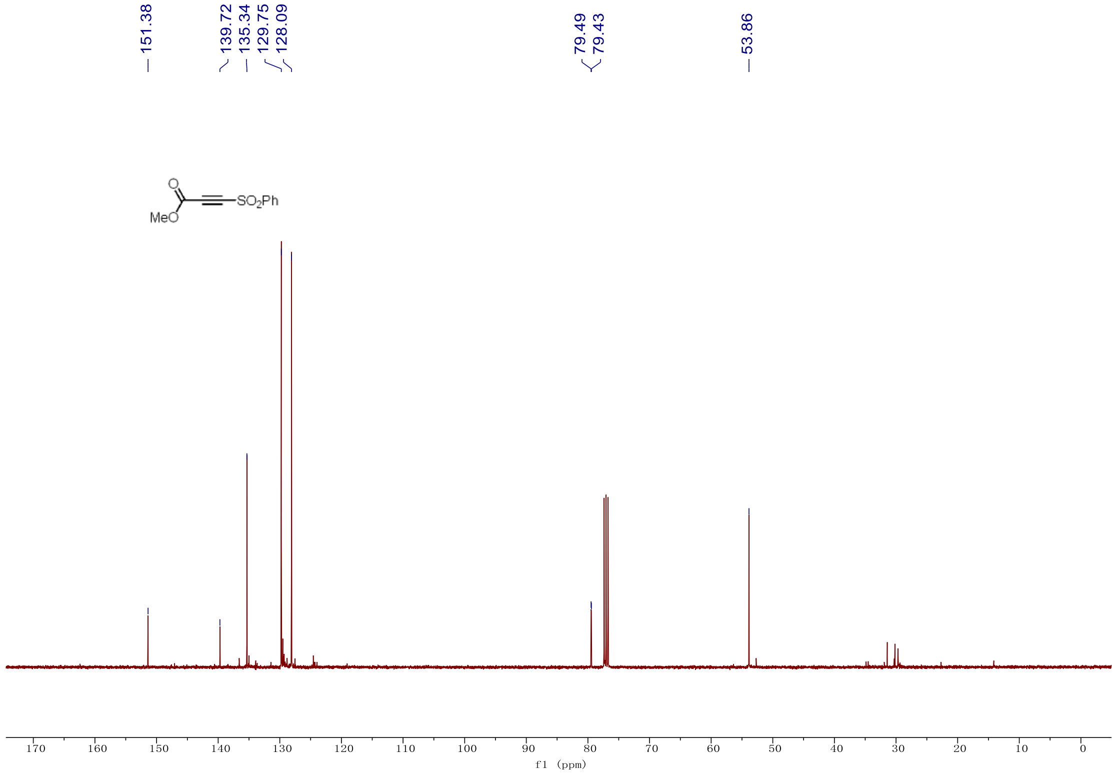
151.38
139.72
135.34
129.75
128.09
79.49
79.43
53.86
MeO
O
SO2Ph
170 160 150 140 130 120 110 100 90 80 70 60 50 40 30 20 10 0
f1 (ppm)
